# NetREm: Network Regression Embeddings reveal cell-type transcription factor coordination for gene regulation

**DOI:** 10.1093/bioadv/vbae206

**Published:** 2024-12-20

**Authors:** Saniya Khullar, Xiang Huang, Raghu Ramesh, John Svaren, Daifeng Wang

**Affiliations:** Waisman Center, University of Wisconsin-Madison, Madison, WI 53705, United States; Department of Biostatistics and Medical Informatics, University of Wisconsin-Madison, Madison, WI 53076, United States; Waisman Center, University of Wisconsin-Madison, Madison, WI 53705, United States; Waisman Center, University of Wisconsin-Madison, Madison, WI 53705, United States; Comparative Biomedical Sciences Training Program, University of Wisconsin-Madison, Madison, WI 53706, United States; Waisman Center, University of Wisconsin-Madison, Madison, WI 53705, United States; Department of Comparative Biosciences, School of Veterinary Medicine, University of Wisconsin-Madison, Madison, WI 53706, United States; Waisman Center, University of Wisconsin-Madison, Madison, WI 53705, United States; Department of Biostatistics and Medical Informatics, University of Wisconsin-Madison, Madison, WI 53076, United States; Department of Computer Sciences, University of Wisconsin-Madison, Madison, WI 53706, United States

## Abstract

**Motivation:**

Transcription factor (TF) coordination plays a key role in gene regulation via direct and/or indirect protein–protein interactions (PPIs) and co-binding to regulatory elements on DNA. Single-cell technologies facilitate gene expression measurement for individual cells and cell-type identification, yet the connection between TF-TF coordination and target gene (TG) regulation of various cell types remains unclear.

**Results:**

To address this, we introduce our innovative computational approach, Network Regression Embeddings (NetREm), to reveal cell-type TF-TF coordination activities for TG regulation. NetREm leverages network-constrained regularization, using prior knowledge of PPIs among TFs, to analyze single-cell gene expression data, uncovering cell-type coordinating TFs and identifying revolutionary TF-TG candidate regulatory network links. NetREm’s performance is validated using simulation studies and benchmarked across several datasets in humans, mice, yeast. Further, we showcase NetREm’s ability to prioritize valid novel human TF-TF coordination links in 9 peripheral blood mononuclear and 42 immune cell sub-types. We apply NetREm to examine cell-type networks in central and peripheral nerve systems (e.g. neuronal, glial, Schwann cells) and in Alzheimer’s disease versus Controls. Top predictions are validated with experimental data from rat, mouse, and human models. Additional functional genomics data helps link genetic variants to our TF-TG regulatory and TF-TF coordination networks.

**Availability and implementation:**

https://github.com/SaniyaKhullar/NetREm.

## 1 Introduction

Transcription factors (TFs) collaborate to activate or repress the expression of their target genes (TGs) by binding to specific DNA sequences known as TF binding sites (TFBSs) on regulatory elements (REs; e.g. enhancers, promoters). These REs are shaped by the combinatorial interactions of multiple TFs, forming transcriptional regulatory modules (TRMs) that govern transcription initiation (Wunderlich and Mirny 2009, Guo and Gifford 2017). TFs often work in concert, either forming stable complexes or enhancing the binding affinity of nearby TFs (Nie *et al.* 2020, Zhao 2023). It is not uncommon for the regulation of one TG to necessitate interactions with 10–15 TFBSs (Bentsen *et al.* 2022). Despite the common occurrence of TF-TF coordination, the complexities underlying this phenomenon are not yet fully understood. The coordinated activity of a few core TFs, among hundreds expressed, defines a cell type’s unique transcriptional landscape and identity (Lee *et al.* 2012, D’Alessio *et al.* 2015). Indeed, the TF binding grammar is complex and context-specific, influenced by proteins occupying shared REs and the chromatin structure.

Gene regulatory networks (GRNs: models of TG regulation by TFs) must incorporate this complex combinatorial coordination (nature: cooperative, antagonistic; type: direct, indirect) among TFs, which is facilitated by the positioning of TFBSs within REs (Ouyang *et al.* 2009, Wang *et al.* 2015a). Historically, classical models of TF cooperativity were predicated on direct protein–protein interactions (PPIs) since many TFs may form complexes [to increase their binding affinity and motif specificity (Sönmezer *et al.* 2021)] to co-regulate TGs (Wang *et al.* 2023). Based on recent studies, it is now understood that TFs may jointly coordinate TG transcription even without direct PPIs (Wang *et al.* 2009, Rao *et al.* 2021). Pioneer TFs, for instance, recognize and bind to their TFBSs even within closed chromatin, inducing local chromatin remodeling (e.g. nucleosome eviction), thereby exposing TFBSs and rendering the DNA more amenable to subsequent binding by settler TFs (Wang *et al.* 2009, Sinha *et al.* 2023). Tethered-binding mechanisms, involving co-activators/co-repressors, allow TFs to regulate TGs cooperatively by recruiting intermediary proteins (e.g. p300/CBP, mediator complexes) (Spitz and Furlong 2012, Ibarra *et al.* 2020). Antagonistic coordination, via sequestration and/or competition for TFBSs, generates cell-state heterogeneity by driving opposing effects on epigenetic programs and TG expression; some TFs bind to DNA only in the presence of cooperating TFs and the absence of antagonistic TFs (Hu *et al.* 2022, Berenson *et al.* 2023).

Despite extensive single-cell data analyses (Wang *et al.* 2018, Mathys *et al.* 2019), the extent of TF-TF coordination in regulating TGs across various cell types remains unclear (Hannenhalli and Levy 2002, Karczewski *et al.* 2011, Ibarra *et al.* 2020, Nie *et al.* 2020, Varjosalo 2022). Understanding this is crucial for dissecting the cell-type GRNs driving cognitive, motor, and behavioral processes, which are often dysregulated in disease states. State-of-the-art (SOTA) GRN-inference tools typically reverse-engineer expression data to find coordinated patterns and gene-gene interactions, uncovering meaningful biological signals from noise (de Campos *et al.* 2019). Such tools create cell-type GRNs using various computational methods on scRNA-seq (single-cell RNA-sequencing) gene expression data, such as: multi-omics integration [scGRNom (Jin *et al.* 2021)], co-expression [GRNBoost2 (Moerman *et al.* 2019)], graphical models and dependency networks [Inferelator (Greenfield *et al.* 2013), MERLIN (Roy *et al.* 2013), SCENIC (Aibar *et al.* 2017), SILGGM (Zhang *et al.* 2018)], information theory [knnDREMI (van Dijk *et al.* 2018), PIDC (Chan *et al.* 2017), Scribe (Qiu *et al.* 2020), RTN (Fletcher *et al.* 2013)], ordinary differential equations [SCODE (Matsumoto *et al.* 2017)], correlation [LEAP (Specht and Li 2017), Pearson *r*], machine learning. Nonetheless, these SOTA tools often overlook the interdependent TF-TF PPI networks (PPINs) that are crucial for TG regulation. Neglecting PPINs may lead these tools to disregard $TF_{i}$-TG pairs with uncorrelated gene expression (Zaborowski and Walther 2020), which may sadly bury true biological relations, as a given $TF_{i}$ could still regulate the TG through complex avenues, often coordinating synergistically or antagonistically with other TFs. Coordinating TFs are often co-expressed (Ouyang *et al.* 2009) and amplify noise in expression profiles (Nagamine *et al.* 2005, Perna *et al.* 2020, Parab *et al.* 2022). Unfortunately, without additional context, these tools might remove truly causal correlated TFs that co-regulate TGs, excluding fundamental aspects of GRN mechanisms (Nicodemus and Malley 2009). Thus, GRN inference tools ought to incorporate various levels of expression regulation, like PPIs for TFs, as they can help reveal these dynamics of TF-TF coordination (Nagamine *et al.* 2005, Perna *et al.* 2020, Parab *et al.* 2022). Tools like BGRMI (Iglesias-Martinez *et al.* 2016) identify relevant TF complexes using PPIs, but ignore inherent PPIN structures and indirect PPIs. RTNduals (Chagas *et al.* 2019) builds a GRN using the RTN tool and then predicts TF co-regulatory behavior solely from expression data, which may not always yield outputs. SCINET (Mohammadi *et al.* 2019) reconstructs cell-type interactomes, while TF-Cluster (Nie *et al.* 2011) identifies coordinated TFs but avoids using prior information; neither tool focuses on TG regulation.

Previous studies have leveraged network-regularized (e.g. graph-regularized, network Lasso) regression models to identify disease-associated genes and networks, incorporating biological information as network metadata (Li and Li 2008, Li and Li 2010, Kim *et al.* 2013, Wang *et al.* 2015b, Dirmeier *et al.* 2018). These models, guided and constrained by prior knowledge, enhance their biological relevance; however, they are not tailored to elucidate TF-TF coordination in TG regulation. There is a growing need for these models to advance, assimilating information from regression data and prior network inputs to generate robust embeddings that may extract significant relationships, offering new insights in biology and beyond (Choy *et al.* 2018, Gharavi *et al.* 2021, Chu *et al.* 2023, Khullar 2024).

To bridge these gaps, we developed Network Regression Embeddings (NetREm), a novel computational framework to infer cell-type TF-TF coordination activities for TG regulation. Building on established network-regularized regression techniques, NetREm integrates multimodal data capturing: intricate aspects of TG regulation [e.g. TF binding profiles, derived TF-TF colocalization, gene expression, chromatin interaction, scATAC-seq (Assay for Transposase-Accessible Chromatin using sequencing) epigenomic markers] and prior network knowledge of interactions among TF predictors (e.g. direct and/or indirect PPIs among TFs). NetREm constructs a robust predictive model for TF-TG regulation (complementary GRN, which is comparable in performance to SOTA GRNs) as well as for TF-TF coordination (at the TG level and the overall cell-type level). Public databases like STRINGdb provide direct and/or indirect, organism-specific, global, undirected, cell-type-agnostic PPIs for >12k organisms (Szklarczyk *et al.* 2023). Despite PPINs having some incorrect PPIs [False Positives (FPs)] and being largely incomplete (Kotlyar *et al.* 2022), it is still helpful to integrate PPINs among TFs (i.e. TF-TF PPINs) as prior biological knowledge (Imoto *et al.* 2003, Mukherjee and Speed 2008, Ghanbari *et al.* 2015, Li and Jackson 2015, McCalla *et al.* 2023); this integration thus improves NetREm’s predicted GRNs from gene expression data. A distinct feature of NetREm is its innovative ability to generate network regression embeddings, which identify and quantify coordination among cell-type TFs for co-regulating individual TGs. In addition, NetREm address the need for not only cell-type-specific annotation of known PPIs but also the discovery of novel PPIs. It provides much-needed insights into TRMs, reveals cell-type-specific and disease-specific TF-TF PPINs, and aids protein interactome studies in uncovering disease gene properties and differential PPIN rewiring (Sevimoglu and Arga 2014, Göös *et al.* 2022). To demonstrate, we benchmark NetREm in various contexts. We also apply NetREm to nervous system (NS) cell types: myelinating (mSCs) versus nonmyelinating (nmSCs) Schwann cells (SCs), as well as in Alzheimer’s disease (AD) versus control states in neurons/glia. NetREm is an open-source tool for general-purpose use.

## 2 Methods

### 2.1 NetREm methodology

#### 2.1.1 Integration of multimodal data and networks in NetREm work-flow

*Preliminary definitions:* TFs (types of proteins) and target genes (TGs, written in *italics*) are represented by their HGNC (HUGO Gene Nomenclature Committee) symbols. TF gene expression is a proxy for TF protein abundance, with the assumption that high gene expression [quantifies messenger RNA (mRNA) transcript abundance] translates to high protein expression in the cell. NetREm can be applied to expression data (sample by gene) at not only the bulk-level but also the single-cell level. Bulk data represents pooled collections of cell lines or tissues, yielding averaged expression profiles. Supplementary File S5 provides more details for each step in our NetREm methodology.

We start with single-cell gene expression data for M samples (individual cells) and T genes in a cell-type; scRNA-seq data is typically high-dimensional (M≪T), sparse, non-negative. We focus on G cell-type TGs, (i.e. ${\left\{{\mathrm{TG}}_{k}\right\}}_{k=1}^{G}$), with expression profiles ${\left\{y_{k}\right\}}_{k=1}^{G}$, respectively, and N potential cell-type TFs, where G≤T, N<T. If all TFs are master regulators, then: T=G+N; else (some TGs are TFs): T<G+N. We ensure that a given TG is not its own candidate TF. NetREm can use prior gene regulatory network (GRN) information that identifies ${\left\{N_{k}\right\}}_{k=1}^{G}\;$respective promising candidate TFs for the set of G TGs where $N_{k}\leq N$ and varies based on the optimal prior GRN TFs selected for the given ${\mathrm{TG}}_{k}$. When prior reference GRN knowledge is absent, ${\mathrm{TG}}_{k}$ (and the other G−1 TGs) will have the same fixed $N_{k}=N$ candidate TFs; it is to be understood that if ${\mathrm{TG}}_{k}$ is also a TF, then its self-TF is excluded, so it has the remaining $N_{k}=N$– 1 candidate TFs.

These steps below are repeated for each of the G TGs. For simplicity, we explain the pipeline for predicting expression levels of a single TG. We use N to represent the number of candidate TFs for this TG, and y for this TG’s true expression across the samples.

*Optional (recommended) prior cell-type GRN information:* Constructing candidate regulatory links from TFs to TGs may improve the quality of NetREm’s solution via initial feature selection of N biologically meaningful TFs tailored for TG, where N≤N. Using diverse information on transcriptional regulation of expression guides the regression (Zaborowski and Walther 2020). Later, we will illustrate how we integrate prior GRNs [initial TF-Regulatory Element (RE)-TG links derived from multi-omics data] in our real-world applications 11 and 12, which are related to Schwann cell (SC) sub-types and Alzheimer’s disease (AD) versus controls in 8 cell types, respectively. When no prior GRN is used: if this TG is a TF, then this TG has N=N-1 candidate TFs; otherwise it has all N TFs as candidates.

*Input PPI network (PPIN, network prior):* Our weighted, undirected, comprehensive PPIN W illuminates biological interactions among proteins (graph or network nodes) that have strong functional association evidence. W inherently captures direct (e.g. complex formation, transient interactions) and/or indirect [e.g. participate in shared processes or bind by intermediate hidden partners (De Las Rivas and Fontanillo 2012)] PPIs. Alas, current PPINs traditionally attribute weight w>0 to all PPIs, even to those with antagonistic, competing roles in pathways (Szklarczyk *et al.* 2023); each edge weight $w_{ij}$ accounts for uncertainty in W and is proportional to the probability that the two connected protein nodes (protein*_i_* and protein*_j_*) interact (i.e. their integrative functional essentiality and biological significance in vital cellular processes) (Li and Liu 2022). While NetREm is equipped to utilize context-specific PPINs (e.g. cell-type-specific and/or tissue-specific PPINs), such data remains relatively scarce and underdeveloped (Kotlyar *et al.* 2022, Kupershmidt *et al.* 2024). Currently, most PPINs are global and organism-specific, as efforts to define refined PPINs for organisms are in their infancy. To navigate these constraints, we calibrated NetREm to effectively harness the wisdom of these global, organism-specific PPIs, maximizing the utility of public datasets. As advancements in PPI mapping generate more granular, context-specific input PPIN datasets, we anticipate that NetREm’s performance will improve.

For the TG, we subset W to obtain $W^{\left(0\right)}$ that: captures known TF-TF PPIs, reflects potential structure/relation background information among TG’s N candidate TF proteins, and is symmetric (i.e. ${\left(W^{\left(0\right)}\right)}^{T}=W^{\left(0\right)}$). A higher $w_{ij}$ value denotes more confidence that $TF_{i}$ and $TF_{j}$ partner, directly and/or indirectly, in processes like regulating DNA chromatin loops of interacting regulatory elements (REs) for TG regulation (Wang *et al.* 2021). Here, $w_{\min}^{\left(0\right)}=\min\left\{w_{ij}^{\left(0\right)}\in W^{\left(0\right)}\right\}\;$is the smallest confidence for known TF-TF links among the N TFs. Nonetheless, some of these N candidate TFs for TG may not be in $W^{\left(0\right)}\;$and edges may be missing for existing TF nodes. We input this potentially partly connected $W^{\left(0\right)}\;$to NetREm.

*NetREm preprocesses the input PPIN* $W^{\left(0\right)}$: To enable NetREm to consider candidate TFs missing from $W^{\left(0\right)}$, ensure numerical stability, and propel the discovery of novel TF-TF links, we need the final, preprocessed TG-specific, TF-TF input PPIN W to include all N TFs and be fully connected. Thus, under-the-hood, NetREm adds an artificial weight $0<\eta <w_{\min}^{\left(0\right)}\;$for missing pairwise edges in $W^{\left(0\right)}$; these novel edges may not exist [i.e. the given pair of TFs truly do not coordinate: True Negatives (TNs)] or are yet to be discovered [i.e. are False Negatives (FNs)]. We obtain our final, undirected, preprocessed fully connected $W\in R^{N\times N}$, which has $\frac{N\left(N-1\right)}{2}\;$unitless PPIs for all N candidate TFs for TG with $w_{ij}>0\;$equal to: $w_{ij}^{\left(0\right)}$ for known and η for artificially added TF-TF links (for *i* ≠ *j*). We do not consider self-loops. Instead, we set $W_{ii}=\frac{d_{i}}{N-1}$ where $TF_{i}$’s degree (connectivity) with the other N-1 TFs is $d_{i}=\sum_{k\neq i}w_{ik}>0$; this representation of $W_{ii}$ is a mathematical treatment that will be used to help solve the network-regularized regression problem defined in the upcoming Step 1.

$$\begin{array}{c} W=\left[\begin{array}{cccc} \frac{\sum_{k=2}^{N}w_{1k}}{N-1} & w_{12} & \ldots & w_{1N}\\ w_{21} & \frac{\sum_{k=1,\;k\neq 2}^{N}w_{2k}}{N-1} & \ldots & w_{2N}\\ \vdots & \vdots & \ddots & \vdots \\ w_{N1} & w_{N2} & \ldots & \frac{\sum_{k=1,\;k\neq N}^{N}w_{Nk}}{N-1} \end{array}\right]\\ {=\left[\begin{array}{cccc} \frac{d_{1}}{N-1} & w_{12} & \ldots & w_{1N}\\ w_{21} & \frac{d_{2}}{N-1} & \ldots & w_{2N}\\ \vdots & \vdots & \ddots & \vdots \\ w_{N1} & w_{N2} & \ldots & \frac{d_{N}}{N-1} \end{array}\right]}_{N\;\times \;N} \end{array}$$

*NetREm preprocesses the input gene expression data:* For the M samples, the $X\in R^{M\times N}$ matrix (where ℝ denotes the set of real numbers) contains expression data for N predictors, while $y\in R^{M}$ is the expression vector for TG. We standardize each column of X: ($X_{ij}\leftarrow \frac{X_{ij}-\mu_{j}}{\sigma_{j}})$ and y: ($y_{i}\leftarrow \frac{y_{i}-\mu_{y}}{\sigma_{y}}$) by respective original means, $\mu_{j}\;\mathrm{and}\;\mu_{y}$, and by standard deviations, $\sigma_{i}\;\mathrm{and}\;\sigma_{y}$, thereby rendering our continuous gene expression data unitless. Then, after this standardization, each TF in X has standardized expression levels with updated values: $\mu_{j}\approx 0$ and $\sigma_{j}\approx 1$ for *j* ∈ {1, … *N*}; similarly, TG now has its expression levels y characterized by $\mu_{y}\approx \;$0 and σy≈ 1. The original pairwise Pearson correlations, r, are preserved. If M≪N, then X suffers from the curse of dimensionality. With technological advances and the advent of large-scale single-cell sequencing studies, we anticipate a boost in M such that M≫N will soon be the norm in gene expression datasets (Cuomo *et al.* 2023, Emani *et al.* 2024, Gupta *et al.* 2024), especially since N≪T (relatively few genes are transcribed and translated to proteins that function as TFs).

*NetREm integrates input data:* NetREm identifies which of these N candidate TFs can predict TG expression values y across the samples, considering PPIs among TFs based on the preprocessed TF-TF PPIN for TG: W. All in all, NetREm can comprehensively integrate multi-omics data and PPINs to discover key TFs and TF-TF coordination events for TG regulation in a cell-type-specific manner.

#### 2.1.2 Step 1: network regularized regression (problem definition)

Given gene expression data with M samples (rows) and N features (columns) represented as $X\in R^{M\times N}$ (e.g. N candidate TF expression values for each M) and response $y\in R^{M}$ (e.g. TG expression for each M), we want to learn a linear predictor $y\approx \;Xc^{*}$ for TG. By imposing prior TF-TF PPIN information as a regularization term, we develop a functional map highlighting connectivity patterns among these N TFs for TG; this typically steers NetREm to favor groups of TFs with greater shared PPIN connectivity over relatively isolated TFs, thereby enhancing its ability to capture biologically relevant PPIs. This is the grouped variable selection property embodied by network-regularized regression models (Li and Liu 2022) like NetREm. Coefficients $c^{*}\in R^{N}\;$represent the importance of TFs for regulating TG and are found by optimizing the following problem with this objective function:

(1) $$c^{*}=\underset{c}{\mathrm{argmin}}f\left(c\right)=\frac{1}{2M}{\left|\left|y-Xc\right|\right|}^{2}+\alpha {\left|\left|c\right|\right|}_{1}+\frac{\beta }{2}\sum_{i=1}^{N}\sum_{j=i}^{N}w_{ij}{\left(\frac{c_{i}}{\sqrt{d_{i}}}-\frac{c_{j}}{\sqrt{d_{j}}}\right)}^{2}\;$$

Our **3 terms** in Equation (1) are unitless and compatible for addition: **#1** (data-fitting): ensures that Xc is close to y, and $\frac{1}{2M}$ is a normalization factor to make it invariant to sample size M. **#2** (sparsity-prior): favors a sparse solution (small number of non-zero $c^{*}$), helping simplify the model (i.e. fewer final TF predictors $N^{*}$) and boost reliability. **#3** (network-prior): penalizes differences between $c^{*}$ of connected pairwise TF nodes, normalized by their respective network centrality d and adjusted for their PPI weights with other candidate TFs in the TG-specific input W. Inspired by (Li and Li 2008), this approach allows for a more equitable representation of TFs, irrespective of their d; this network-oriented variant of the Ridge $L_{2}$ penalty $\sum_{i=1}^{N}c_{i}^{2}$, promotes topology-aware $c^{*\;}$ shrinkage and smoothing for neighboring nodes $TF_{i}$ and $TF_{j}$, with probability proportional to $w_{ij}$. It underscores the principle that strongly connected TFs likely perform shared functions, even if their individual impacts on TG expression (i.e. their $c^{*}$ signs) differ. This recognizes the community structure in existing PPINs that groups proteins with similar biological roles with w>0, not distinguishing between cooperative (+) and antagonistic (−) PPIs (Padi and Quackenbush 2015, Szklarczyk *et al.* 2023). NetREm leverages this refined understanding, offering a comprehensive perspective on the interplay among TFs in W.

We can tune these two hyperparameter knobs: network-constrained prior, β>0, decides the strength of the PPIN regularization penalty (applied 1st: a higher β guides NetREm to prioritize TFs with stronger mutual PPIs by amplifying the punishment for selecting TFs with relatively weaker PPIs in the final model); sparsity prior, α≥0, impacts the Lasso $L_{1}$ penalty (applied 2nd: a higher α encourages NetREm to produce simpler models that are more robust to noise in the expression data). NetREm, a PPIN-aware adaptation of ElasticNet regression, performs automatic variable selection based on both expression and PPIN data, grouping and selecting strongly connected TFs (emphasizing more trustworthy known TF-TF PPI subnetworks) in a spirit akin to ElasticNet. If M≪N, ElasticNet and network-regularized models (like NetREm) may still select $N^{*}$≤N TFs as final in their respective solutions; these approaches address limitations of Lasso regression that may indiscriminately select only 1 TF from a group of highly correlated TFs and only $N^{*}$≤M TFs if M≪N (Zou and Hastie 2005, Li and Li 2008).

#### 2.1.3 Step 2: gene expression embeddings from network regression

Our novel method transforms the original problem into a Lasso regression problem in a new space with cell-type-specific TF-TF interactions. First, we represent the network-prior term in a more compact matrix–vector form as: $\frac{\beta }{2}\sum_{i=1}^{N}\sum_{j=i}^{N}w_{ij}{\left(\frac{c_{i}}{\sqrt{d_{i}}}-\frac{c_{j}}{\sqrt{d_{j}}}\right)}^{2}=\frac{\beta }{2}c^{T}Ac$, where $A=D^{T}\left(W⊙V\right)D=[\begin{array}{cccc} 1 & -w_{12}/\sqrt{d_{1}d_{2}} & \ldots & -w_{1N}/\sqrt{d_{1}d_{N}}\\ -w_{21}/\sqrt{d_{1}d_{2}} & 1 & \ldots & -w_{2N}/\sqrt{d_{2}d_{N}}\\ \vdots & \vdots & \ddots & \vdots \\ -w_{N1}/\sqrt{d_{1}d_{N}} & -w_{N2}/\sqrt{d_{2}d_{N}} & \ldots & 1 \end{array}].\;A,\;D,\;V,\;W$ are all symmetric matrices with dimensions: $R^{N\times N}$. We define $V=N\cdot I-11^{T}$ where $1\;\in R^{N\times 1}$ is a N dimensional all 1 column vector, *I* is the identity matrix (main diagonals: 1, off-diagonals: 0), and W⊙V is the element-wise (⊙) multiplication of W and V (i.e. their Hadamard product). Diagonal matrix $D=\mathrm{diag}\left(1/\sqrt{d}\right)\in R^{N\times N}$ has main diagonal degree-based elements: $1/\sqrt{d_{i}}$ and off-diagonals: 0. V only depends on N (the # of candidate TFs) to obtain its constant values. That is, $V={\left[\begin{array}{cccc} N-1 & -1 & \ldots & -1\\ -1 & N-1 & \ldots & -1\\ \vdots & \vdots & \ddots & \vdots \\ -1 & -1 & \ldots & N-1 \end{array}\right]}_{N\times N}$ and $W⊙V={\left[\begin{array}{cccc} d_{1} & -w_{12} & \ldots & -w_{1N}\\ -w_{21} & d_{2} & \ldots & -w_{2N}\\ \vdots & \vdots & \ddots & \vdots \\ -w_{N1} & -w_{N2} & \ldots & d_{N} \end{array}\right]}_{N\times N}$.

Given that squared term${\;\left(\frac{c_{i}}{\sqrt{d_{i}}}-\frac{c_{j}}{\sqrt{d_{j}}}\right)}^{2}\geq 0$ and $w_{ij}\geq \eta >0$ and β>0, the quadratic form $c^{T}Ac\geq 0$ for any vector of optimal $c^{*}$ and $A=\left[a_{ij}\right]\in R^{N\times N}$ is therefore symmetric and positive semi-definite. Here, $a_{ij}=a_{ji}<0\;$for i≠j and main diagonal $a_{ii}=1\;$for i=1,…,N. A scales each entry ${\left(W⊙V\right)}_{ij}\;$by $1/\sqrt{d_{i}d_{j}}$ so $A_{ij}={\left(W⊙V\right)}_{ij}/\sqrt{d_{i}d_{j}}$. Hence, A captures connectivity and interaction strengths in W in a form suitable for regularized regression. As A is based on a fully connected PPIN (i.e. there are no completely isolated subsets or islands of TF nodes), A is a variant of normalized graph Laplacian matrix L, specifically engineered for mathematical contexts where w and d are crucial. Off-diagonal elements in A are $-1\leq A_{ij}=-w_{ij}/\sqrt{d_{i}d_{j}}<0$ where i≠j; since $d_{i}=\sum_{k\neq i}w_{ik}\geq w_{ij}$ and $d_{j}=\sum_{k\neq j}w_{jk}\geq w_{ji}$, then $\sqrt{d_{i}d_{j}}\geq w_{ij}$. These negative off-diagonal values indicate the respective penalty for dissimilarity between connected nodes since the regularization term aims to minimize differences in characteristics (modeled by $\left|c^{*}\right|$) amongst TFs by smoothing $c^{*}$ across the input PPIN based on network properties and bonds among the candidate TFs.

Using this matrix–vector representation, we reformulate $f\left(c\right)$ in Eq (1) as $f\left(c\right)=\frac{1}{2M}{\left|\left|y-Xc\right|\right|}^{2}+\alpha {\left|\left|c\right|\right|}_{1}+\frac{\beta }{2}c^{T}Ac$. We note that ${\left|\left|y-Xc\right|\right|}^{2}={\left(y-Xc\right)}^{T}\left(y-Xc\right)=y^{T}y-2y^{T}Xc+c^{T}X^{T}Xc$.

$$\mathrm{Thus},\;\;f\left(c\right)=\frac{1}{2M}\left(y^{T}y-2y^{T}Xc+c^{T}X^{T}Xc\right)+\alpha {\left|\left|c\right|\right|}_{1}+\frac{\beta }{2}c^{T}Ac=\frac{1}{2M}c^{T}\left(X^{T}X+\beta MA\right)c\;-\;\frac{1}{M}y^{T}Xc+\;\alpha {\left|\left|c\right|\right|}_{1}+\;\frac{1}{2M}y^{T}y$$

We set $E=\frac{X^{T}X}{M}+\beta A\in R^{N\;\times \;N}$, by dividing $X^{T}X+\beta MA$ by M. E is a symmetric and positive semi-definite matrix since A and Gram matrix $X^{T}X\in R^{N\;\times \;N}$ are both symmetric and positive semi-definite and β,M,N>0. Therefore, *E* consists of two terms: $\frac{X^{T}X}{M}$ and βA. ${\left(X^{T}X\right)}_{ii}=M$, which reflects the sum of squared values of each TF and is indicative of the variance ($\sigma^{2}=1$) scaled by M. Off-diagonals ${\left(X^{T}X\right)}_{ij}$, for i≠j, are sums of products of pairs of different TFs; hence, $\left|{\left(X^{T}X\right)}_{ij}\right|\leq M$ since $\frac{{\left(X^{T}X\right)}_{ij}}{M}$ represents: $\left|r\left(TF_{i},\;TF_{j}\right)\right|\leq 1$ for Pearson correlation r. That is, $\frac{{\left(X^{T}X\right)}_{ij}}{M}\in$ [−1, 1] represents *r* between $TF_{i}$ and $TF_{j}$ because the columns of *X* have been standardized to have zero mean and unit variance in the preprocessing step. This first expression-based term (cell-type-specific data term) for E is: $\frac{X^{T}X}{M}$, represents pairwise r among TFs, has maximum value 1, and is the covariance matrix of X scaled by $\frac{1}{M}.$ The second term (cell-type-independent PPI term): βA is β for main-diagonals and $-\beta \leq {\beta A}_{ij}<0\;(\mathrm{for}\;i\neq j)$ for off-diagonals. In the future, as context-specific PPINs become available, they may be input to NetREm in place of global, organism-specific PPINs to improve the cell-type-specificity of our results. Thus, $E_{ij}=r\left(x_{i},x_{j}\right)-\beta \frac{w_{ij}}{\sqrt{d_{i}d_{j}}}$, which balances r with scholarly PPIN knowledge and expertise$\left(\frac{w_{ij}}{\sqrt{d_{i}d_{j}}}\right)$ for a given β.

(2) $$\begin{array}{c} f(c)=\frac{1}{2M}c^{T}\left(X^{T}X+M\beta A\right)c\;-\;\frac{1}{M}y^{T}Xc+\;\alpha {\left|\left|c\right|\right|}_{1}+\;\frac{1}{2M}y^{T}y\\ \begin{array}{c} =\frac{1}{2N}c^{T}{\tilde{X}}^{T}\tilde{X}c\;-\;\frac{1}{N}{\tilde{y}}^{T}\tilde{X}c+\alpha {\left|\left|c\right|\right|}_{1}+\frac{1}{2M}y^{T}y\;\\ =\frac{1}{2N}{\left|\left|\tilde{y}-\tilde{X}c\right|\right|}^{2}+\alpha {\left|\left|c\right|\right|}_{1}+\frac{1}{2M}y^{T}y\;-\;\frac{1}{2N}{\tilde{y}}^{T}\tilde{y},\; \end{array} \end{array}$$

where $\tilde{X}\in R^{N\times N}$ and is symmetric (i.e. $\tilde{X}={\tilde{X}}^{T}$) and $\tilde{y}\;\in R^{N\;}$satisfies:

(3a) $$\frac{1}{N}{\tilde{X}}^{T}\tilde{X}={\frac{1}{M}X}^{T}X+\beta A$$

 

(3b) $$\frac{1}{N}{\tilde{y}}^{T}\tilde{X}=\frac{1}{M}y^{T}X$$

Finally, we reformulate Equation (2) as a conventional Lasso problem (by omitting the constant term $\frac{1}{2M}y^{T}y-\frac{1}{2N}{\tilde{y}}^{T}\tilde{y}$), which we solve using standard Lasso (α is fixed) or LassoCV [optimal α is selected via cross-validation (CV)] regression solvers (Pedregosa *et al.* 2011):

(4) $$c^{*}=\underset{c}{\mathrm{argmin}}\tilde{f}(c)=\frac{1}{2N}{\left|\left|\tilde{y}-\tilde{X}c\right|\right|}^{2}+\alpha {\left|\left|c\right|\right|}_{1}\;$$

To compute $\tilde{X}\;\mathrm{and}\;\tilde{y}$, we perform a Singular Value Decomposition (SVD) on E expressed as: $E=U\Sigma U^{T}.$ Here, $U\in R^{N\times N}\;$is the matrix of the left singular vectors of E and $\Sigma \;\in R^{N\times N}$ is a diagonal matrix of singular values $S=\left\{s_{1},s_{2},\ldots ,\;s_{N}\right\}$ of E. All N values in S are non-negative and convey information regarding the strength of each corresponding dimension [$s_{\max}=\max\left(S\right)$; $s_{\min}=\min\left(S\right)]$. Then, $E=U\Sigma^{\frac{1}{2}}\Sigma^{\frac{1}{2}}U^{T}={\left(\Sigma^{\frac{1}{2}}U^{T}\right)}^{T}\left(\Sigma^{\frac{1}{2}}U^{T}\right)$. Based on Eq (3a), $E=\frac{1}{N}{\tilde{X}}^{T}\tilde{X}$, so ${NE=\tilde{X}}^{T}\tilde{X}$. Hence, ${\left(\sqrt{N}\Sigma^{\frac{1}{2}}U^{T}\right)}^{T}\left({\sqrt{N}\Sigma }^{\frac{1}{2}}U^{T}\right)={\tilde{X}}^{T}\tilde{X}$. For improved stability, we set small singular values (e.g. $s_{i}<{10^{-6}s}_{\max}$) to 0, resulting in a truncated matrix of singular values, $\Sigma_{\mathrm{trunc}}$; similarly, we adjust the inverse matrix ${\Sigma_{\mathrm{trunc}}}^{-1}$ by setting inverse elements corresponding to small singular values to 0. By substituting ${\Sigma_{\mathrm{trunc}}}^{-\frac{1}{2}}\;$in place of $\Sigma^{-\frac{1}{2}}$, we effectively use truncated SVD, thereby enhancing NetREm’s robustness by excluding contributions from small singular values. Thus, X and y are transformed to a new latent space of gene expression embeddings that incorporate PPIN information: $\tilde{X}\in R^{N\times N}$ and $\tilde{y}\in R^{N}$, respectively, in Equation (4):

$$\begin{array}{c} \tilde{X}=\sqrt{N}{\Sigma_{\mathrm{trunc}}}^{\frac{1}{2}}U^{T}\;\mathrm{where}\;\\ \tilde{X}={\left[\begin{array}{ccc} | & | & \cdots \\ \tilde{X_{1}} & \tilde{X_{2}} & \ldots \\ | & | & \cdots \end{array}\;\begin{array}{c} |\\ \tilde{X_{N}}\\ | \end{array}\right]}_{N\;\times \;N}\;\mathrm{and}\\ \tilde{y}=\frac{\sqrt{N}}{M}{\Sigma_{\mathrm{trunc}}}^{-\frac{1}{2}}U^{T}X^{T}y \end{array}$$

When β = 0, this transformation yields principal components (PCs) of X via SVD on $X^{T}X$, a trivial case without PPIN information being integrated; however, since we require β > 0, $\tilde{X}$ not only reflects the PCs of X but also includes PPIN structure, with βA added to ${\frac{1}{M}X}^{T}X$. Essentially, this creates an “embedding” that encapsulates both gene expression relations and prior PPIN knowledge. Therefore, our comprehensive approach captures data patterns as well as erudition on PPI network relations. The higher β is, the greater the contribution of existing PPIN wisdom will be toward $\tilde{X}$ and $\tilde{y}$. We perform Lasso regression to solve Equation (4) for $\tilde{X}$ and $\tilde{y},$ determining the optimal $c^{*}.$

#### 2.1.4 Output 1: identification of novel cell-type TFs for regulating TG

*TF-TG regulatory network for TG:* Several TFs regulate the transcriptional activity of TG in a cell type at a certain time (Nie *et al.* 2011). Solving the network-regularized regression problem produces a vector of Lasso coefficients $c^{*}\in R^{N}\;$for N TFs predicting the true TG expression y. We focus on $c^{*}\neq$ 0, which represents our $N^{*}$ final predicted TFs for TG out of N candidates, where 0 < $N^{*}\leq N$. NetREm constructs a comprehensive directed TF-TG regulatory network (complementary GRN) of $N^{*}$ edges, weighted by $c^{*}$. Here, $\left|c_{i}^{*}\right|$ indicates the strength of the $TF_{i}$-TG link, measuring $TF_{i}$’s relative importance in regulating TG. $TF_{i}$ with $c_{i}^{*}>0\;$may activate TG and $TF_{j}$ with $c_{j}^{*}<0\;$may repress the transcription and subsequent expression of TG. This tug-of-war between activators and repressors orchestrates TG regulation. Biological complexity enables certain TFs to have dual-function roles, alternating between activation and repression of their given TGs depending on the context and signals (Boyle and Després 2010, Skok Gibbs *et al.* 2022). Given the competitive nature of TF binding site (TFBS) binding, the $N-N^{*}\;$discarded TFs may lose to some of the $N^{*}\;$TFs (i.e. antagonistic relation); nonetheless, we do not speculate on this. Overall, NetREm unearths novel cell-type-specific, coordinating TFs involved in TG regulation, providing a more nuanced view of TF-TG interactions.

We can evaluate our performance in training and testing gene expression data, by comparing our predicted $\hat{y_{\;}}\in {\mathbb{R}}^{N\times 1}$ to the actual target y expression levels, using regression metrics like Mean Square Error (MSE) = $\frac{1}{M}\sum_{v=1}^{M}(\hat{y_{v\;}}-y_{v}{)}^{2}$. To achieve more accurate regulatory links, we integrate multi-omics data like TF-DNA-binding (Dibaeinia and Sinha 2020) in applications 11–12 to predict prior reference GRNs. We identify regulatory elements (REs) for TG and determine candidate TFs that are likely to bind directly to or associate indirectly with TFBSs on these REs. Then, we input these N TG-specific candidate TFs to NetREm for the TG (i.e. the list and number of TFs can differ among TGs). Lastly, we overlay NetREm’s $N^{*}$ TF-TG regulatory links for TG with this prior reference, candidate GRN (initial TF–RE–TG links for N TFs for TG). This annotates our optimal links with epigenomic information and details on REs. Ultimately, we isolate highly-confident final TF-RE-TG links for our final $N^{*}$ TFs for TG.

*Cell-type TF-TG regulatory network (complementary GRN)*: To obtain our cell-type TF-TG regulatory network, we apply NetREm, iteratively, to each of the G TGs and weave together these individual TF-TG links (details: Supplementary File S5). We may narrow down links by retaining TFs with $\left|c^{*}\right|>c_{\min}$ (minimum threshold, default: 0) and TGs meeting specific criteria (e.g. $MSE_{TG}<MSE_{\max}$). Our GRN relates TFs to TGs they regulate, helping explain how cell types establish and maintain cellular identity. We may annotate/validate this network by identifying eSNPs (expression SNPs) that not only have expression quantitative trait loci (eQTL) links to altered TG expression (Coetzee *et al.* 2015) (i.e. eSNP-eTG links) but also impact TF binding of any of the $N^{*}$ TFs for TG; when prior reference GRNs are used, we employ a more stringent approach to ensure that SNPs (Single Nucleotide Polymorphisms) fall in the same REs where TFs are predicted to bind, linking them to TG regulation.

#### 2.1.5 Output 2: direct and/or indirect TF-TF coordination

*TG-specific TF-TF coordination B in the cell-type:* NetREm helps fulfill the need for cell-type-specific proteome analysis by which proteins interact to carry out processes like TG regulation. Existing PPINs aggregate direct and/or indirect PPIs in an organism; this broad approach has limitations, as not all proteins are expressed in every cell or tissue type, and some may be aberrant in diseases (Padi and Quackenbush 2015). To overcome this, there have been efforts to annotate global PPIs at various levels, utilizing various data, including tissue-specific protein expression, cell-line-specific links, phenotype-based studies (Federico and Monti 2021). However, co-expression of TFs need not imply or guarantee that they interact in specific cell or tissue types (Gonzalez-Teran *et al.* 2022). Further, current PPINs do not distinguish between cooperation and antagonism (i.e. links are usually presented as |w|, thus lacking insight into the true signs of w); indeed, there is a lack of knowledge regarding antagonistic relationships among proteins like TFs (Berenson *et al.* 2023). This underscores the necessity for NetREm’s second output that predicts how TFs coordinate with each other to regulate the TG in the cell type.

This output is a weighted and signed TG-specific TF-TF coordination network given by an adjacency matrix of coordination scores B, if there are $N^{*}\geq 2\;$final TFs for TG. These scores are a function f of both embeddings $\tilde{X}$ and the solution $c^{*}$, such that: $B=f\left(\tilde{X},\;c^{*}\right)$. In our framework, $B_{ij}>0$ suggests mutual cooperativity (e.g. co-binding, pioneer-settler TF relations, recruitment) and $B_{ij}<0\;$implies antagonism (e.g. sequestration, competition) between $TF_{i}$ and $TF_{j}\;$for co-regulating TG. Here, we delineate our process for building this B score that denotes the sign and strength of TG-based TF-TF co-regulatory activity.

We use $c^{*}$ to predict the nature of interactions among $N^{*}\;$TFs for TG in a symmetric matrix $C\in R^{N\times N}$. Here, $C_{ij}=\mathrm{sign}\left(c_{i},c_{j}\right)=\left\{1\;\mathrm{if}\;c_{i}^{*}c_{j}^{*}>0;-1\;\mathrm{if}\;c_{i}^{*}c_{j}^{*}<0;\;0\;\mathrm{otherwise}\right\}$ and $C_{ii}=0$. The RTNduals method assesses the coordinated behavior of 2 TFs by analyzing correlation distributions between them and their shared TGs; building on approaches like these, we use C to deduce the relative coordination relations among TFs for TG, acknowledging that TFs may exhibit antagonistic or cooperative interactions depending on the TG and context. If $C_{ij}>0$, both TFs likely cooperate, aiming to either upregulate or downregulate TG expression in unison; their combined, harmonious, synergistic net effect on the TG is stronger than their individual effects. Conversely, if $C_{ij}<0$, both TFs likely act antagonistically, with conflicting influences on TG expression; this activator–repressor antagonism weakens their combined effect compared to their individual impacts, potentially due to partially canceling each other’s activities. When $C_{ij}=0$ (and i≠j), at least 1 of the 2 TFs is not a final TF for TG and we cannot ascertain their potential nature of interaction.

Earlier, we set $E=\frac{X^{T}X}{M}+\beta A$. The first term $\frac{X^{T}X}{M}$, represents the original normalized inner product space from column vectors $x_{1},x_{2},\ldots ,x_{N}$ of X, where $x_{i}\in R^{M}\;$represents $TF_{i}$’s standardized expression levels across M cells. The second term $A=D^{T}\left(W⊙V\right)D$, purely depends on TF-TF PPIN strengths w>0, and can be retrieved from public databases. By Equation$\;\left(3a\right)$, $E=\frac{X^{T}X}{M}+\beta A=\frac{1}{N}{\tilde{X}}^{T}\tilde{X}$; we thus transform X and network-prior PPIN data to $\tilde{X}$ embedding data. This yields a new normalized inner-product space $\frac{1}{N}{\tilde{X}}^{T}\tilde{X}$ that helps depict and encode an aspect of cell-type TF-TF coordination scores for regulating the TG. Since N is a scalar, we use $\left|{\tilde{X}}^{T}\tilde{X}\right|\in R^{N\times N}$. For each $TF_{i}-TF_{j}$ pair, we divide $\left|{\tilde{X_{i}}}^{T}\tilde{X_{j}\;}\right|$, which is proportional to the extent of their potential coordination, by $\left|\left|\tilde{X_{i}}\right|\right|\cdot \left|\left|\tilde{X_{j}}\right|\right|$ to scale it. $\left|\left|\tilde{X_{i}}\right|\right|=\sqrt{\sum_{z=1}^{M}{\left(\tilde{x_{iz}}\right)}^{2}}>0\;$is the Euclidean norm of $TF_{i}$’s embedding $\tilde{X_{i}}.$ This essentially is their cosine similarity (cos) magnitude: $\left|\cos\left(\tilde{X_{i}},\tilde{X_{j}}\right)\right|=\frac{\left|{\tilde{X_{i}}}^{T}\tilde{X_{j}}\right|}{\left|\left|\tilde{X_{i}}\right|\right|\cdot \left|\left|\tilde{X_{j}}\right|\right|}\leq$ 1. To learn coordination scores, we use a coefficient-aware-cos metric: $B_{ij}^{\left(0\right)}=\left|\cos\left(\tilde{X_{i}},\tilde{X_{j}}\;\right)\right|⊙C$ for i≠j and $B_{ii}^{\left(0\right)}\;$= 0. We apply maximum absolute value scaling $\frac{B_{ij}^{\left(0\right)}}{\max\left(\left|B^{\left(0\right)}\right|\right)}$ on this symmetric matrix where $\max\left(\left|B^{\left(0\right)}\right|\right)$ is the maximum magnitude of these $\ell_{N}=\frac{N\left(N-1\right)}{2}$ pairwise scores among these *N* candidate TFs. Our TG-specific TF-TF coordination $B\in R^{N\times N}\;$has: $B_{ij}=\frac{100B_{ij}^{\left(0\right)}}{\max\left(\left|B\right|\right)}$ where $-100\leq B_{ij}\leq 100$ if $TF_{i}$ and $TF_{j}\;$are among $N^{*}$ TFs (i.e. $c_{i}^{*}c_{j}^{*}\neq 0$) where i≠j; else $B_{ij}$ is 0 for the remaining cases. Of the $\ell_{N}$ undirected links in our symmetric B matrix: $\ell_{N^{*}}=\frac{N^{*}\left(N^{*}-1\right)}{2}$ have ≠0 scores; these $\ell_{N^{*}}$ scored links in B form the TF-TF coordination network that is unique to TG, representing interactions among the $N^{*}$ final TFs that are altogether co-regulating TG.

TF pairs with higher $\left|B\right|$ have stronger relative coordination for co-regulating TG. Ultimately, the pairwise TG-specific TF-TF coordination score for any pair of cell-type TFs will be 0 unless both TFs are in the set of $N^{*}$ final predicted co-regulators of TG. NetREm predicts B for known TF-TF PPIs (pairs with $w_{ij}>\eta$), uncovering meaningful, cell-type-specific PPI subnetworks. These documented PPIs have established partnership for orchestrating biological processes. It also predicts B for artificially-added PPI links ($w_{ij}=\;\eta$), flagging (high $\left|B\right|$) promising, novel, potential FN TF-TF links for follow-up investigation. TF-TF coordination can be direct (e.g. forming complexes, tethering) or indirect (e.g. TFs may not interact physically but can modify local chromatin environments, facilitating binding of other TFs) (Srivastava and Mahony 2020). Typically, direct PPIs rely on genome-wide data, indirect (e.g. guilt-by-association) PPIs use genetic interaction data (Wang *et al.* 2009). While studies predict negatomes (proteins unlikely to interact physically) (Jha *et al.* 2022), they offer limited insights on indirect PPIs. Previous studies (Drew *et al.* 2021) highlight the need for approaches to predict indirect PPIs in addition to direct physical PPIs.

*Overall cell-type-specific TF-TF coordination* $\bar{B}:\;$In Supplementary File S5, we explain how we create our weighted, signed $\bar{B}$ network, where $\bar{B_{ij}}$, a signed percentile, is the net, general, overarching $TF_{i}$ – $TF_{j}$ coordination behavior across all of the fixed G TGs (regardless of whether or not they are co-regulated by both TFs) in the given cell-type. That is, for ease of relative comparison and for holistic analysis, for each TF-TF pair, we consider its overall pattern of coordination across all TGs. The G TG-specific ${\left\{B^{k}\right\}}_{k=1}^{G}\;$networks and overall $\bar{B}$ network both contain scores between −100 and 100; negative scores suggest potential antagonistic co-regulatory relations and positive scores assign potential cooperative relations among the TFs, via direct and/or indirect mechanisms. $\bar{B}$ is a function f of ${\left\{B^{k}\right\}}_{k=1}^{G}\;$across all of the G TGs in the cell type; for each pair of coordinating TFs (i.e. TFs that co-regulate at least 1 of the G TGs), $\bar{B}$ has a unique non-zero value and $\left|\bar{B}\right|\;$represents the relative percentile. Hereafter, please note that for simplicity, we may interchangeably refer to our TG-specific TF-TF coordination scores in the cell type as our individual *B* scores (or *B* network; −100 ≤ *B* ≤ 100) and our overall cell-type TF-TF coordination scores as our $\bar{B}$ scores (or $\bar{B}$ network; −100 ≤ $\bar{B}$ ≤ 100).

### 2.2 Real-world datasets and preprocessing

#### 2.2.1 Overview of 12 real-world applications (12 different studies)

We apply NetREm step-by-step for each TG in the cell type for 12 main applications (A) spanning 3 organisms [human (Homo Sapiens): 1–2, 5, 7, 8–12; mouse (Mus Musculus): 3–4; yeast (Saccharomyces Cerevisiae): 6]. These individual TG-specific models (regulatory networks and TF-TF coordination networks) are then used to derive the aggregate, overall, cell-type-specific (or tissue-type-specific) networks for the organism. When we benchmark NetREm across 13 human contexts, we use data from A: 8–9 and all 8 cell types in Control stages from A12. Supplementary File S5 details parameters and evaluation for applications. Supplementary Tables S1 and S2 break down the number of TGs, number of TFs if N is fixed (A1–A10) or metrics if N is variable (A11–A12).

We run NetREm without prior reference GRN knowledge for these 10 A: **1:** Simulated data for human Embryonic Stem cells (hESCs). **2:** Human Hematopoietic Stem cells (HSCs), which are self-renewing and long-living cells in bone marrow that are essential to produce blood cells. **3:** Mouse Embryonic Stem cells (mESCs), which are derived from the inner cell mass of the early embryo and are pluripotent since they can self-renew, develop, specialize, differentiate, and mature into any cell type. **4:** Mouse Dendritic cells (mDCs), which capture and present antigens to other immune cells. **5:** Human Pluripotent Stem cells (hPSCs) that can differentiate into nearly any cell type. **6:** Budding Yeast cells (YCs): covering 11 environmental conditions and 12 genotypes of this eukaryotic fungus. **7:** 9 Peripheral Blood Mononuclear cell (PBMC) sub-types that are part of the immune system. We train NetREm on each of these 9 sub-types with 1,029 TFs (Lambert *et al.* 2018). **8:** Pooled Schwann cells (SCs) across 5 tissues. **9:** 4 primary Central Nervous System (CNS) cell-types: GABA-ergic Inhibitory neurons (InNs), Glutamatergic Excitatory neurons (ExNs), Oligodendrocytes (Oligo), Microglia (Mic). **10:** 62 immune cell sub-types. We run A10 with 125 relevant TFs tested by (Berenson *et al.* 2023). In these 10 applications, we fix our *N* candidate TFs to be the same across all our *G* respective TGs: the N potential TFs for the given cell type. If the TG is also considered to encode a potential cell-type TF in N, we then remove it from the set for the TG, so this TG will have the remaining N=N-1 TFs as its candidate input TFs for NetREm (i.e. its *X* will lack the column for that given self-TF to prevent the TG from being its own predictor); it follows that its input PPIN among the predictors will be a subset of the PPIN used for the other TFs (i.e. we drop links for that TF from the input PPIN). Thus, for A1-10, we simply run NetREm using gene expression data and a PPI network among the N TF predictors, where *X* ∈ R^*M* × N^ and *W* ∈ R^N × N^ are held static for all TGs in the application except for those that also function as TFs (in those cases, *X* ∈ R^*M* × (N–1)^ and *W* ∈ R^(N–1) × (N–1)^). In A1–6, we use gold standards to hone our *G* TGs and N relevant TFs, so that our TF-TG links are comparable with the current ground truth; thus, we train NetREm models for each of these *G* TGs using these *N* TFs as candidates, where both sets of TGs and TFs are found in the expression data as well as the ground truth data. In A8–9, we run NetREm with all genes as TGs and all cell-type TFs as candidate TFs.

We run NetREm on two applications in humans using prior reference GRNs from multi-omics data to define a custom set of N highly probable, candidate TFs for each TG (N differs across TGs): **11:** Myelinating (mSCs) and nonmyelinating (nmSCs) human Schwann cells in the peripheral nervous system (PNS). **12:** Alzheimer’s disease (AD) and Control stages in humans for eight cell-types in the CNS. Four glial cells: Astrocytes (Astro), Oligo, Oligo Progenitor cells (OPCs or Oligodendrocyte Precursor cells); Mic; two neuronal cells: InNs, ExNs; two vascular and blood–brain barrier (BBB) cells: Pericytes, Endothelial BBB (Endo. BBB) cells. Therefore, for each context in A11–12, we derive a prior reference GRN of initial TF-RE-TG links and run NetREm for each TG, utilizing the TFs identified in this GRN as its *N* ≤ N TG-specific candidate regulators. Accordingly, for each TG, NetREm’s input matrices *X* ∈ R*^M × N^* and *W* ∈ R*^N × N^* are adjusted, dynamically, to reflect its *N* candidate TFs (since *N* may vary across TGs).

#### 2.2.2 Single-cell gene expression data

Supplementary File S5 provides preprocessing details for our 12 applications A. In A1, 11–12 we use 70% of data for training, 30% for testing. **1:** We randomly select 1,250 TGs and corresponding TFs from a weighted and signed (+: activates; −: represses) ground truth GRN atlas from TF induction analysis (Sharov *et al.* 2022). This results in N = 207 TFs and 5,050 GRN links that we input to the SERGIO tool (Dibaeinia and Sinha 2020) to simulate realistic single-cell data for 100 cells and 1,000 cells with 1,442 genes in total. When running SERGIO, we vary the noise parameter (30, 60, 90)%, thus retrieving 3 different synthetic expression datasets for a given number of cells. Hence, we have 6 datasets. **2:** We use Buenrostro *et al.* (2018). **3:** We use (Tran *et al.* 2019) that reprograms mouse embryonic fibroblasts to embryonic-like induced pluripotent stem cells. In A4–6, we use McCalla *et al.* (2023) for the final, normalized preprocessed data for the following original datasets: **4:**  Shalek *et al.* (2014) for >1.7k primary bone marrow DCs. **5:**  Han *et al.* (2018) for 5,520 hPSCs. **6:**  Jackson *et al.* (2020) for 17,396 cells in budding yeast. **7:** Public healthy donor data on 2.7k PBMCs (Satija 2024). **8:** Genotype-Tissue Expression (GTEx) (Eraslan *et al.* 2022). **9:**  Jin *et al.* (2021) for preprocessed postmortem nuclear transcriptomic tissue expression in the human frontal cortex (Lake *et al.* 2018). **10:**  The Tabula Sapiens Consortium (2022) Atlas for >264k human immune cells pooled across 25 tissues. **11:**  Avraham *et al.* (2022) for mSCs/nmSCs in Dorsal Root Ganglion (DRG) L4,5 regions from 5 donors. **12:** Preprocessed data (Gupta *et al.* 2022a) for 24 AD and 24 healthy humans based on 80,660 droplet-based single-nucleus prefrontal cortex brain transcriptomes (Mathys *et al.* 2019).

#### 2.2.3 Prior GRN reference information on TG regulation

In A11–12, we employ multi-omics and epigenetic data on open scATAC-seq chromatin regions mapped to TGs (i.e. peak-to-TG links) to identify potential interacting REs for TGs. We map sequence-specific TF motifs to REs, using Position Weight Matrix (PWM) databases to predict TFBSs, forming a motif-based GRN (comprises direct TF-RE-TG candidate links). We prune this motif-based TF list based on relative TF gene expression levels (proxy for TF protein abundance levels) and motif matching scores. To capture overlooked TFs, we augment our pruned TF list by adding TFs with not only known PPIs but also predictions of TF-TF colocalization, protein complexes, and/or Gene Ontology Molecular Function (GOMF) similarity (Wu *et al.* 2021). Our adaptation addresses limitations of GRN-inference tools that rely solely on accessible motif matches, potentially missing causal TFs for TGs (Zhang *et al.* 2023); as ≈10% of the ≈1.6k human TFs lack motif data and are traditionally excluded, using PPIs during GRN inference is recommended to help incorporate these missing TFs (Badia-I-Mompel *et al.* 2023). Augmented TFs may bind to TFBSs directly (weak signals) or indirectly (associate with DNA-binding TFs) (Gordân *et al.* 2009, Sloan *et al.* 2016). Our final prior, reference, candidate GRNs comprise these initial TF-RE-TG links. We input the N biologically promising TG-specific candidate corresponding TFs to NetREm for the given TG; NetREm will ultimately select its optimal subset of TF-TG links (i.e. its final predicted GRN) from this prior GRN. These subjective steps are detailed in Supplementary File S5. In general, various creative approaches can assist us with inferring prior reference GRNs (initial candidate: TF-RE-TG links or simple TF-TG links), depending on the multi-omics data available for our desired application. For instance, another option could be to first run an alternative GRN method (using the expression data and other relevant data) as a first pass to predict links and define a prior GRN as input for NetREm; second, we can then apply NetREm for each TG, in our application, using the expression data for these TG-specific candidate TFs (regulatory knowledge gleaned from the prior GRN) as our input features.

#### 2.2.4 Protein–protein interaction networks

We use organism-specific input PPINs derived from publicly available databases and remove any self-loops. In Supplementary File S5, we explain the resources and preprocessing we use to build our final PPINs.

*Final Comprehensive (Partly Connected) PPINs* W: To construct the input PPINs for mice and yeast, we employ STRINGdb (Szklarczyk *et al.* 2023) for all interactions (reflecting evidence from co-expression, co-localization, evolutionary conservation, shared pathways, experimental validation, and beyond). For our human applications, we create our main, comprehensive NetREm input PPIN (i.e. “Main NetREm PPIN”) by pooling PPIs, curated by gurus in the field, from several global human resources, including the entire STRING version 12 (v12) all database. Specifically, STRING enables us to build weighted PPINs that include both direct (e.g. physical binding, complex co-existence) and indirect (e.g. metabolic/signaling pathways) interactions for the organism. We leverage STRING’s full network of scored links to incorporate multiple lines of evidence for each known and/or predicted PPI, represented as combined scores. To cap our edge weights w at 1, we scale these combined scores by dividing by them by the maximum combined score in the PPIN.

For comparative analysis of NetREm across 13 human contexts, we use many supplementary human PPINs (that have been thoughtfully created using different criteria and resources) that are weighted [e.g. HIPPIE (Human Integrated PPI Reference) (Alanis-Lobato *et al.* 2017), hu.MAP (Human Protein Complex Map) 2.0 (Drew *et al.* 2021), Max SAINT (Significance Analysis of INTeractome) Score (Göös *et al.* 2022)] or dummy [Barabasi (Morselli Gysi *et al.* 2021), Integrated Interactions Database (IID) (Kotlyar *et al.* 2022) filtered for nervous system-related PPIs, Final Interactome (Kosoglu *et al.* 2024), HuRI_HI-union (Human Reference Protein Interactome Mapping Project) (Luck *et al.* 2020), Multiscale Interactome (Ruiz *et al.* 2021)]. We assign w = 1 to all PPIs in any dummy PPINs as those provide no information on weights.

Overall, for each PPIN, we apply a minimum threshold to ensure that only interactions with meaningful confidence (0.01 <w≤1) are retained in our final, corresponding PPIN W. At present, our PPINs (except for the Contextual IID) are global (organism-based, lacking cell-type and tissue specificity), reflecting the fledgling stage of efforts to develop more refined, context-specific networks for organisms (Kotlyar *et al.* 2022, Kupershmidt *et al.* 2024). We are hopeful that in coming years, weighted, context-specific PPINs will become the norm rather than the exception, allowing NetREm to increasingly leverage them as prior knowledge input for enhanced performance.

*Final Fully Connected TG-specific TF-TF PPIN* W: For each TG (of G TGs in a context), we filter the respective W to only keep its N candidate TFs to yield a potentially partly connected $W^{\left(0\right)}\;$that we input to NetREm as known TF-TF links among these candidates. Internally, NetREm assigns an artificial positive weight η = 0.01 (that we set based on our preferences) to missing pairwise edges in $W^{\left(0\right)}$, building a comprehensive preprocessed, TG-specific W (used for subsequent steps in the pipeline) where for all *N* potential TFs: 0.01 $\leq w_{ij}\leq$1 for i≠j.

### 2.3 Data access

Please note that we implement NetREm as an open-source software package with details in Supplementary File S5. Supplementary Table S3 lists the resources that we used. We use human hg38 and rat rn5 reference genomes. Metrics for NetREm’s final cell-type networks in A11 (human SC subtypes: mSCs versus nmSCs) and A12 (AD versus control across 8 neuronal/glial cell types) are available in Supplementary Table S4 and visualized in the associated panel: Supplementary Fig. S1A and B.

## 3 Results

### 3.1 Overview of NetREm

**Figure 1.**
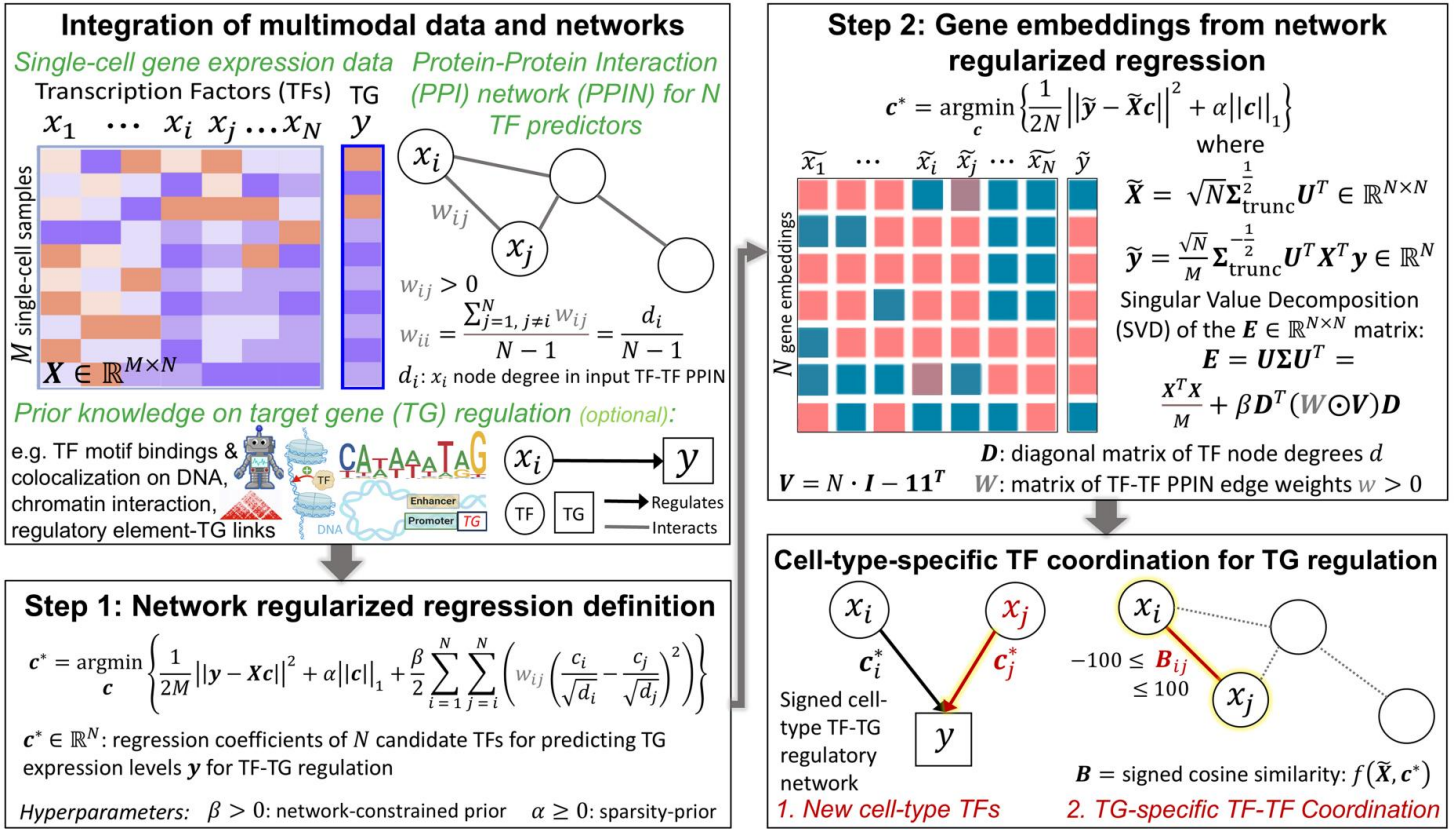
Overview of Network Regression Embeddings (NetREm): a multi-step, multi-omics computational framework to construct comprehensive cell-type-specific TF-TG regulatory and TF-TF coordination $\bar{B}\;$networks. We apply this pipeline for each TG in a cell-type. NetREm integrates multimodal data: gene expression ($X\in R^{M\times N}$, $y\in R^{M}$) for M cells (samples), direct/indirect TF-TF PPIN $W^{\left(0\right)}\;$with non-negative weights, optional prior GRN information (e.g. initial TF-RE-TG links). This prior candidate GRN helps select only relevant TF predictors for TG (from multi-omics data) to reduce the dimensionality of X: M (rows), N candidate TFs (columns). X and y are standardized (mean μ: 0, standard deviation σ: 1) across cells for TFs and TG. Goal: identify TFs, out of N candidates, whose expressions best predict TG expression y. **Step 1** sets up a PPIN-regularized regression problem (preprocessing $W^{\left(0\right)}\;$with artificially added minimum edges to ensure that our final W is fully connected for N TFs) to identify optimal TFs for this TG, guided by network β and sparsity α hyperparameters. **Step 2** solves this, transforming these inputs to latent space gene expression embeddings ($\tilde{X}\in R^{N\times N}$, $\tilde{y}\in R^{N}$) by performing SVD on the $E=\frac{X^{T}X}{M}+\beta A\;$matrix ($A=D^{T}\left(W⊙V\right)D$). E combines gene expression relations and PPIN information. ($\tilde{X},\;\tilde{y}$) undergo Lasso regression (via model-type: Lasso (α is given) or LassoCV [α chosen by cross-validation (CV)]; default: no *y*-intercept term) to predict optimal coefficients $c^{*}\in R^{N}$ for the regulation of TG. NetREm outputs 2 networks capturing different aspects of TG regulation that can be integrated. *#1:* links optimal TFs to TG by $c^{*}$ (> 0: activator, < 0: repressor), in a directed TF-TG regulatory network, a complementary GRN. This likely uncovers novel cell-type TFs like $x_{j}$ (that is, $TF_{j}$) and reflects the underlying biology of coordination among TFs. If we input a prior GRN, we may use it to help annotate our TF-TG links with biological metadata (e.g. TF-RE-TG network). *#2*: TG-specific undirected TF-TF coordination network B predicts indirect/direct relations among TFs to regulate TG. $TF_{i}-TF_{j}$ coordination $B_{ij}$ shows cooperative (>0 if both are co-activators or co-repressors) or antagonistic: (<0 if 1 TF is a repressor and the other TF is an activator) co-regulation of TG. B is a matrix of $c^{*}$-aware cosine similarity scores (Section 2), a function of $\tilde{X}\;$and $c^{*}$. NetREm thus identifies novel coordination among cell-type TFs for co-regulating TG. Results are stitched together across runs for all G TGs in the cell type to obtain final cell-type-specific outputs.

We have developed NetREm as a robust, multi-omics, computational approach to build and integrate networks of TF-to-TG regulation and TF-TF coordination in a cell-type-specific manner (Fig. 1). Network regularization-based approaches like NetREm are useful in applications where predictors form subnetworks to influence the outcome (Li and Liu 2022). NetREm uses single-cell or bulk gene expression data and a comprehensive PPIN ($W^{\left(0\right)}$) that captures verified direct and/or indirect functional associations among TFs. While the current landscape primarily supports the use of global, organism-specific PPINs, NetREm is positioned to evolve alongside the development of cell-type or context-specific interaction data, leveraging these burgeoning advancements to enhance predictive capabilities. Providing a prior, reference candidate GRN [e.g. initial TF-regulatory element (RE)-TG links] to NetREm is optional but is recommended as it improves the biological relevance of outputs with its initial feature selection; elected TFs in this prior GRN are candidate regulators for the TG and NetREm identifies the subset of these initial links that are predictive of TG expression. NetREm integrates this multimodal data, applying a 2-step optimization process for each of the G TGs individually: **1.** It formulates a network-regularized regression problem, using the input PPIN as a priori info, to sift through N candidate TF proteins, to find those most likely to co-regulate the given TG. To do this, NetREm transforms the input $W^{\left(0\right)}\;$to a fully connected TF-TF PPIN W comprising not only known but also artificially added links (with minimum positive weight $w_{\min}=\eta$) for the N candidate TFs for this TG. **2.** To solve this regression problem, NetREm employs Singular Value Decomposition (SVD) to create network regression embeddings, which are used in Lasso regression to predict TG expression. In the process, NetREm learns not only key TF regulators (coefficients $c^{*}$ that describe the TF-TG regulatory links; potential regulatory roles for $TF_{i}$: $c_{i}^{*}>0$ → activator, $c_{i}^{*}<0$ → repressor) but also a TG-specific cell-type TF-TF coordination network −100 ≤ B≤ 100, predicting cooperative (B > 0) and/or antagonistic (B < 0) TF-TF relations for regulating TG. NetREm also annotates known PPIs, uncovering confident cell-type-specific TF-TF PPI subnetworks involved in TG regulation. By integrating foundational genomic PPIs, NetREm uses transfer learning to discover unanticipated, biologically significant TF-TG links and novel functional TF-TF relations for future investigation. Thus, there are G individual: B networks and TF-TG networks for the G respective TGs that can be woven together. Aggregating these outputs across all G TGs, yields these 2 weighted, signed, cell-type-specific networks: a directed TF-TG regulatory network [that is a complementary state-of-the-art (SOTA) GRN] and an undirected overall TF-TF coordination (−100$\leq \bar{B}\leq$100) network. These individual (TG-based) and aggregate (overall) outputs support downstream analyses; for instance, we may link noncoding SNPs to potential regulatory roles via eQTL SNP-TG (i.e. eSNP-eTG) associations in our networks.

We demonstrate NetREm’s versatility across diverse real-world scenarios, by benchmarking our TF-TG regulatory networks against established gold standard GRN models in humans [Embryonic Stem cells (hESCs), Hematopoietic Stem cells (HSCs), Pluripotent Stem cells (hPSCs)], mice [ESCs (mESCs), Dendritic cells (mDCs)], budding Yeast cells (YCs). We use diverse techniques to assess $\bar{B}$ across these settings and additional human contexts: 9 PBMCs, pooled SCs, 62 pooled immune cell sub-types, 4 primary CNS cell types. Further, we highlight NetREm’s use of prior input reference GRNs, derived from multi-omics data, for specific contexts (cell types, diseases) in two human applications: **1.** myelinating (mSCs) versus nonmyelinating (nmSCs) SC sub-types; **2.** AD versus Controls in 8 neuron/glia cell types. The resulting TF-TG networks, integrated with TF-RE-TG annotations (Section 2), yield enhanced, context-specific TF-RE-TG regulatory and TF-TF coordination networks.

### 3.2 Simulation study

**Figure 2.**
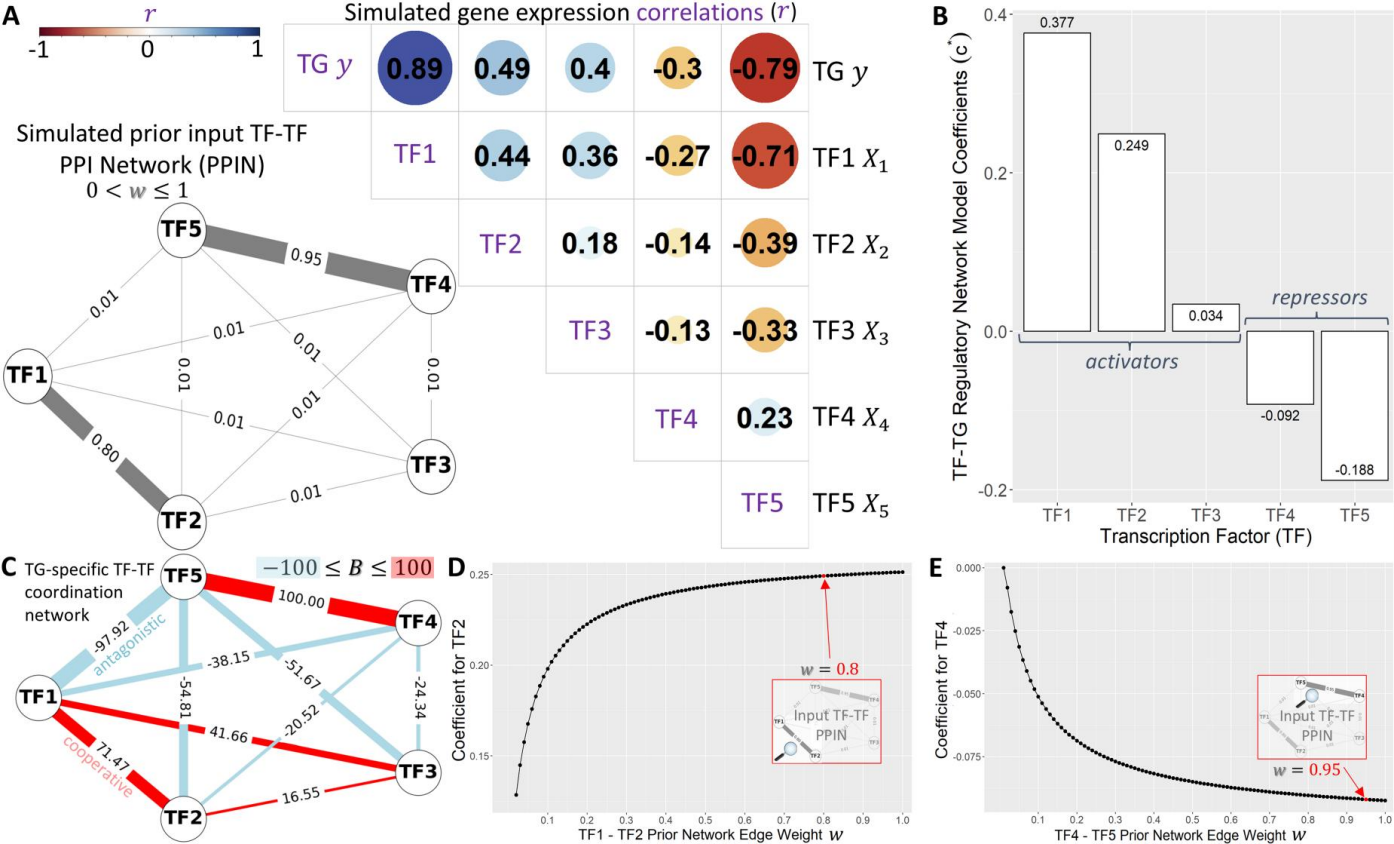
Simulation study of five TFs, one TG. (A) Bottom left: prior, preprocessed, fully connected undirected TF-TF PPIN W with default weights (η = 0.01) added; strong experimentally verified connections: $TF_{1}$-$TF_{2}$ (0.8); $TF_{4}$-$TF_{5}$ (0.95). Top right: Pearson correlation r matrix in training data: r(TF, TG)≈ [0.9, 0.5, 0.4, −0.3, −0.8]. Dot sizes are based on $\left|r\right|$ and colored based on whether r is + or −. (B) Coefficients $c^{*}$ for TFs in TF-TG regulatory network, based on NetREm(β =1, α = 0.1, no y-intercept term). Potential activators ($c^{*}>0$): $TF_{1}$ to $TF_{3}$. Repressors ($c^{*}<0$): $TF_{4}$ and $TF_{5}$. (C) TG-specific TF-TF coordination network (scores: B) colored based on whether the indirect and/or direct links are predicted as antagonistic (−; activator–repressor links) or cooperative (+; links between co-activators or co-repressors). Functionally validated coordination: $TF_{1}$-$TF_{2}$ and $TF_{4}$-$TF_{5}$; others are novel. (D) Effects of varying $TF_{1}$-$TF_{2}\;w$ in the original TF-TF input PPIN, from 0.01 to 1 in 0.01 increments, holding all else fixed. Respective $TF_{2}\;c^{*}$ increases monotonically in an arc shape from 0.106 to 0.251 as w increases. (E) Similar sensitivity analysis shows $TF_{4}$’s $\left|c^{*}\right|$ increases, becoming more negative from 0 to −9.2e−2, as $TF_{4}$-$TF_{5}\;$edge weight in TF-TF input PPIN is perturbed.

We test NetREm on simulated single-cell gene expression data for 10,000 cells as a proof-of-concept, where X (matrix of real value expression levels for N = 5 candidate TF predictors: $R^{10,000\;\text{samples}\;\times 5\;\text{features}\;}$) and y (TG expression vector: $R^{10,000\;\text{samples}\;\times 1}$) are drawn from a normal distribution with dropouts to achieve ≈40% sparsity, mimicking single-cell data (Supplementary Fig. S2A). Training data (70% of the original data; M: 7,000 cells) is used to standardize the data ($X_{\mathrm{train}}\in R^{7,000\times 5},\;y_{\mathrm{train}}\in R^{7,000\times 1},\;X_{\mathrm{test}}\in R^{3,000\times 5},y_{\mathrm{test}}\in R^{3,000\times 1}$) so that each variable has: mean μ = 0, standard deviation σ = 1. In both training and testing data, TFs 1–5 have respective expression Pearson correlations with y represented by: $r\left(TF,\;TG\right)\approx$ [0.9, 0.5, 0.4, −0.3, −0.8] (Fig. 2A, Supplementary Fig. S2B and C). Our PPIN of known direct and/or indirect PPIs among TFs has strong weights w for $TF_{1}-TF_{2}$ (0.8), $TF_{4}-TF_{5}$ (0.95). NetREm(β = 1, α = 0.1) sets w to η= 0.01 for missing PPIs (making our input PPIN fully connected) and predicts y based on X, subject to the PPIN constraint. It outputs two networks: TF-TG regulation ($c^{*}$ values) relates expression levels of TFs to TG expression (Fig. 2B) and TG-specific TF-TF coordination (B scores) (Fig. 2C) predicts the potential behavior among TFs to regulate this TG. TFs with lower ranks have higher $\left|c^{*}\right|\;$and are more important regulators of TG.

We compare NetREm to four default Python Scikit-Learn (Pedregosa *et al.* 2011) benchmark regression models (BRMs) fit with no y-intercept term: Linear Regression and three regularization ones (ElasticNetCV, LassoCV, RidgeCV). Absolute values of $c^{*}\;$for $TF_{2}$ and $TF_{4}$ are significantly higher in our NetREm model (Supplementary Fig. S2D, Supplementary Table S5: *P* < 2e−16), highlighting NetREm’s grouped variable selection property that prioritizes them due to their strong corresponding PPIs with $TF_{1}\;$and $TF_{5}\;$that both have expression levels strongly correlating with y (Li and Li 2008). BRMs favor $TF_{3}$ over $TF_{4}$ (since $TF_{3}\;$has a greater magnitude of correlation, $\left|r\right|$, with y), which is a trend also observed in the GRN unveiled by the RTN tool. Unlike these five models, NetREm prioritizes $TF_{4}$ over $TF_{3}\;$since $TF_{4}$ strongly interacts with $TF_{5}$, while $TF_{3}$ has weak PPIs with other TFs. Sensitivity analysis (Fig. 2D and E) for fixed α and β confirms $\left|c^{*}\right|\;$for $TF_{2}$ and $TF_{4}\;$increase as their respective PPIs with $TF_{1}$ and $TF_{5}$ strengthen; for reference, the red dots in the plots reflect the actual TF-TF input PPIN w that is selected for our main simulation. NetREm’s test MSE increases from 0.15 to 0.22 as β increases from 0.01 to 1 (Supplementary Fig. S2E). Test MSEs for the 4 BRMs ≈ 0.14, which we can also achieve with NetREm(β = 0.01, α subsequently determined by LassoCV); nonetheless, studies (Li and Liu 2022) emphasize obtaining more interpretable and context-driven features by incorporating prior network knowledge into models, even if accuracy is sacrificed or these prior networks (of relationships among predictors) are imperfect/faulty. Supplementary Figure S2F illustrates changes in B as β increases from 0.01 to 2.

One of NetREm’s salient characteristics is its ability to explicitly model and integrate PPIN structures/relations among TFs. By doing so, NetREm identifies key TFs for predicting y, which may not exhibit strong r with y individually. BRMs may succumb to the pitfalls of expression data that often contains noise of both direct and indirect TF-TG interactions (Escorcia-Rodríguez *et al.* 2023). Integrating the sage PPI expertise, from leading gurus in the field, helps NetREm prioritize essential but less prominent TFs for TG like $TF_{4}\;$(Supplementary Fig. S2G) that may instead form more complex regulatory control mechanisms by coordinating with other TFs (Zaborowski and Walther 2020).

NetREm also generates a coordination network among TFs that co-regulate TG, a capability beyond the scope of BRMs. Instead, BRMs primarily focus on prediction accuracy by selecting $c^{*}$ to best represent each TF’s impact on y, typically treating TFs as “lone wolves,” neglecting the TF-TF interdependencies that are vital to GRNs. Our B network shows that $TF_{1}$ and $TF_{2}\;$cooperate with $TF_{3}$ weakly to increase y, and $TF_{4}$ and $TF_{5}\;$collaborate to decrease it; $TF_{1}$-$TF_{2}$ and $TF_{4}$-$TF_{5}$ links are highly confident (known links in the input PPIN). NetREm predicts novel direct/indirect links like $TF_{1}$-$TF_{5}$ activator-repressor antagonism: B = −97.9. $TF_{3}$ has weaker relations with $TF_{1}\;$than $TF_{2}\;$does (41.7 versus 71.5) and the smallest regulatory role: $\left|c^{*}\right|\;$= 3.4e−2.

Our toy simulation data with a few TFs and 1 TG intuitively explains NetREm and its advantages. We detail more simulations in Supplementary Fig. S3A–C and Supplementary Tables S6 and S7 in Supplementary Methods in Supplementary File S5. We find that: our results are stable and consistent for various expression sparsity levels and M≪N cases (e.g. Supplementary Fig. S4A–I provides a detailed adaptation: M = 6, N = 5), excess β over-constrains NetREm causing its predictions to suffer (bias-variance trade-offs), NetREm has more robust $c^{*}$ estimates (is less variable) and more accurate TF role assignments, and can capture complex TF-TF PPIs. Next, we benchmark NetREm in real-world settings: many TFs and TGs. We apply it step-by-step for each TG in a cell-type to learn B, TF-TG regulation, overall TF-TF coordination $\bar{B}$.

### 3.3 Benchmarking NetREm with No Prior GRN Information

In this section, we assess how effectively NetREm predicts cell-type: TF-TG regulation and TF-TF coordination (TG-specific: B, overall: $\bar{B}$) networks in real-world scenarios lacking prior, candidate GRN information. All TGs have the same N candidate TFs, where we set N = N cell-type TFs; for TGs that are TFs, N is reduced by 1 since that respective TF is omitted (i.e. N = N−1). We anticipate that incorporating prior GRNs derived from multi-omics data may only enhance our performance by providing a tailored, context-specific list of promising TFs for each TG. Here, we benchmark NetREm’s output networks across several mouse, yeast, and human datasets. Moreover, we analyze NetREm’s sensitivity and robustness to changes and evolutions in the input PPIN. Please see Supplementary File S5 for more details.

#### 3.3.1 Evaluating TF-TG regulatory links

To evaluate our networks holistically, we compare NetREm with 4 BRMs in terms of predicted signed ($c^{*}:\;$+/−) TF-TG regulatory links. For this, we use the SERGIO tool to generate 6 realistic datasets (M = 70 or 700 cells in the training data; noise %: 30, 60, and 90) for 1,250 TGs and N=N = 207 hESC TFs, simulated based on a signed ground truth atlas GRN in hESCs. These six synthetic datasets enable us to evaluate performance based on M-to-N ratio and noise that may obscure true signals. These models (except GRNBoost2) use $c^{*}\;$to assign TF roles (activator, repressor); NetREm uses $c^{*}$ to also infer the nature (cooperative, antagonistic) of coordination among TFs for regulating TGs. Overall, NetREm achieves the highest precision in predicting signed-TF-TG links, suggesting its stronger reliability in identifying true TF-TG links [True Positives (TPs)], assigning accurate TF roles, prioritizing promising links with a reduced risk of False Positives (FPs) (Supplementary Fig. S5A–E). When fit with LassoCV (to optimize α given β), NetREm’s TF-TG accuracy is superior to that of GRNBoost2 and BRMs, underscoring its robustness to noise. GRNBoost2 has the lowest accuracy even with complete data usage (M = 100 or 1,000 cells).

NetREm enhances robustness through PPIN regularization. To illustrate this, we benchmark NetREm on real-world single-cell gene expression data in HSCs, hPSCs, mESCs, mDCs, YCs (Supplementary Figs S6A–I and S7A–C; Supplementary Tables S8–S14). These datasets are noisy, sparse, high-dimensional (N≫M) and reflect the inherent complexity of eukaryotic gene regulation. We evaluate our inferred TF-TG links against respective gold standard validation GRN TF-TG links that lack $c^{*}$ sign information. Our findings corroborate that NetREm has higher sensitivity for identifying relevant biomarkers, as it incorporates biological information, but has lower specificity compared to Lasso and ElasticNet models (Li and Li 2008, Shojaie and Michailidis 2009). Tuning hyperparameters β and/or α allows for customized optimization and adjustment of NetREm’s results; for instance, increasing α may: reduce sensitivity, increase specificity.

Without the use of prior PPI information that NetREm incorporates, predicting TF-TG links using single-cell expression data alone can be problematic. This expression data often fails to accurately represent expression distributions (Nguyen *et al.* 2020). It is further challenging to identify TFs that truly coordinate solely based on similar expression correlations (Hoefsloot *et al.* 2008; de Campos *et al.* 2019). Correlated TFs may either coordinate to causally co-regulate TGs (Ouyang *et al.* 2009, Wang *et al.* 2016, Ahsendorf *et al.* 2017) or merely be spuriously co-associated. Linear Regression and Ridge regression tend to retain correlated TFs, Lasso regression often drops them (potentially omitting co-regulating TFs), ElasticNet regression balances Ridge and Lasso regression models. Thus, BRMs may struggle to identify functionally coordinated TFs for TG regulation (Nie *et al.* 2011), compromising the integrity of their TF-TG regulatory networks (Nicodemus and Malley 2009, Roy *et al.* 2020). On the other hand, NetREm considers not only TF-TF expression correlations but also known information on TF-TF PPIs, helping account for noise in expression data due to TF-TF coordination (Parab *et al.* 2022). NetREm can thus uncover TF-TF interactions for TG regulation, discerning each TF’s influence with superior generalizability and consistency, capturing intricate relations that BRMs cannot.

NetREm’s optimization incorporates PPIN structures among TFs to encourage the selection of strongly grouped TFs that are involved in known, biological PPIs. We highlight this grouped variable selection property (Li and Li 2008) in HSCs where we run NetREm and benchmark models for 10,588 TGs and N = 178 TFs (Supplementary Table S8). RTN falters, yielding a GRN of just 152 links (of which 16 are True Positives (TPs)) for only 16 TGs; RTNduals, which uses RTN to infer its GRN, cannot predict TF-TF coordination links in HSCs. ElasticNetCV and LassoCV are limited in identifying TF-TG links for TGs. For instance, both models notably miss the regulation of *ATF2*, a gene that translates to a pivotal TF in stem cells (Ju *et al.* 2023). On the other end, Linear Regression and RidgeCV predict that *ATF2* is regulated by all 177 possible TFs, illustrating their alarming potential for FPs. However, for β = 10, NetREm uncovers 8 final TFs for regulating *ATF2* where all but WHSC1 are substantiated by gold standards (Zhang *et al.* 2023). We note the same for TGs including *BRD2, RNF167, DUSP2*. NetREm flags groups of verified and novel coordinating TFs connected along biologically meaningful, cell-type PPIs for these four TGs (Supplementary Fig. S6A–I) and beyond! For instance, TFAP4, 1 of our 10 novel TFs for *RNF167*, is involved in adipogenesis and negative regulation of cell population proliferation with 3 of 17 substantiated final TFs. These examples not only validate NetREm’s applicability to complex biological systems but also show, directly, its ability to effectively identify accurate TF groupings from a large pool of candidate TFs for enhanced GRN inference. In fact, NetREm’s TF-TG regulatory networks may be considered complementary to those prophesied by existing SOTA cell-type GRN tools.

**Figure 3.**
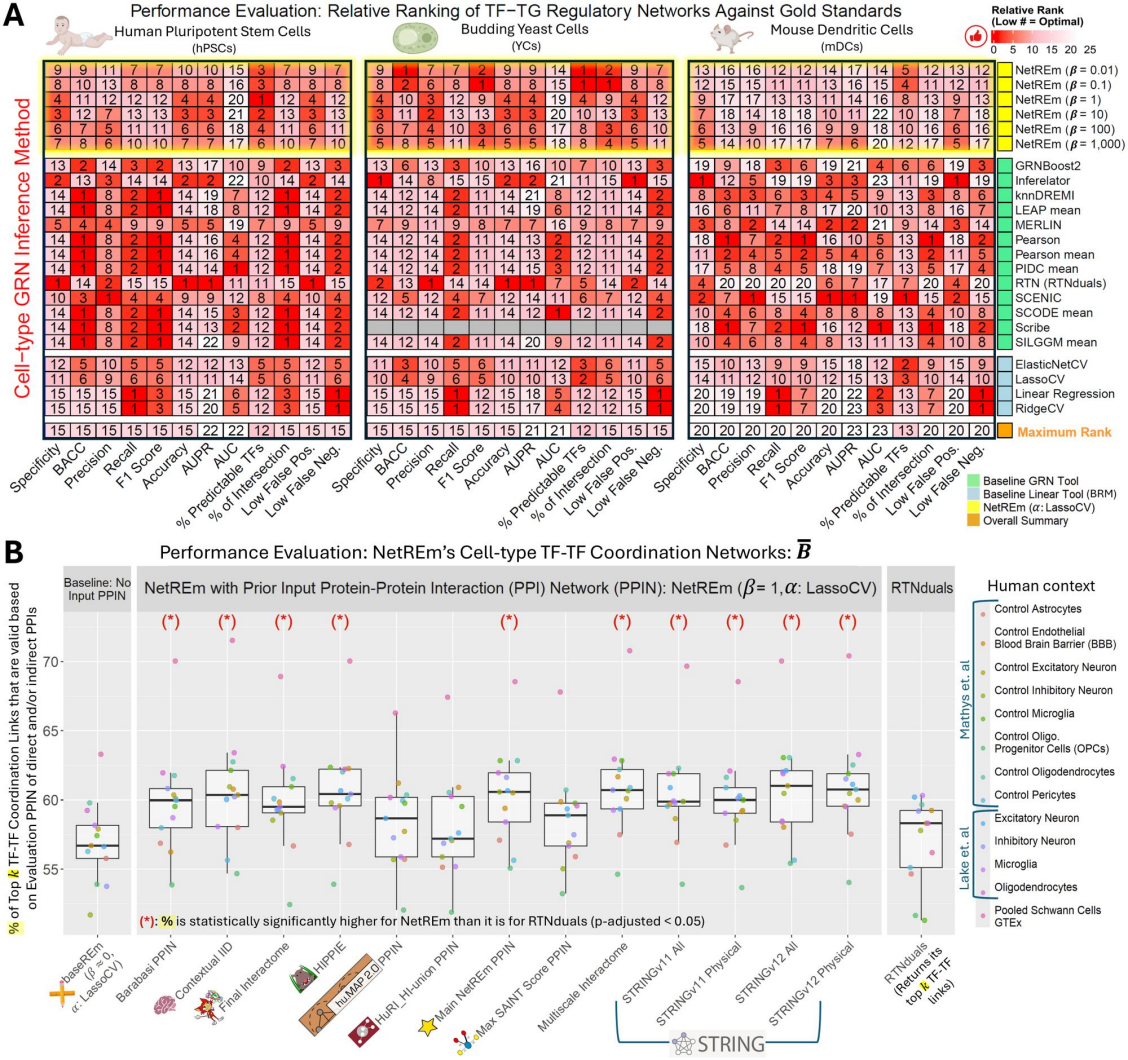
Benchmarking NetREm’s cell-type TF-TG regulatory networks and TF-TF coordination networks across datasets in the absence of prior gene regulatory network (GRN) knowledge. (A) Comparing the performance of NetREm’s complementary cell-type GRNs against those of BRMs and state-of-the-art (SOTA) GRN inference tools for three applications spanning three organisms: hPSCs, YCs, mDCs. Several NetREm (α subsequently determined by LassoCV) models are run for different β. Various metrics are used to evaluate the GRNs, including: balanced accuracy (BACC), Area Under the Precision Recall curve (AUPR), AUC [Area Under the ROC (Receiver operating characteristic) curve]. Lower rank values: more optimal TF-TG GRN metrics. Bottom row: maximum ranking for that application and that metric, given that some tools may tie with others. Across applications and metrics, no method singularly outperforms all the others. This illustrates that NetREm’s TF-TG regulatory networks may be considered complementary GRNs. Tuning NetREm’s β yields different performance results. (B) Comparative boxplots paired with underlying dot plots (% values for each context for a given model) of the performance of predicted cell-type TF-TF coordination networks $\bar{B}$ for 13 human contexts in terms of predicting valid TF-TF links based on an Evaluation PPIN. For each context, RTNduals outputs its top k TF-TF links and the top k number of links (high $\left|\bar{B}\right|$) are extracted, respectively, from each of the NetREm (α: LassoCV) models to facilitate ease of comparison; the % of k links that are valid based on an Evaluation PPIN are then returned. Left: baseline model of NetREm run in the absence of an input PPIN (i.e. baseREm: positive β  ≈ 0). Right: RTNduals tool used for benchmarking. Middle: 13 NetREm (β = 1) models for different input PPI networks. 10 of 13 NetREm models with a (*) on top predict TF-TF coordination links that have a statistically significantly higher % of valid links across the 13 contexts when compared with RTNduals.

NetREm’s performance as a complementary SOTA GRN inference tool is validated through comprehensive benchmarking across diverse biological applications. For this analysis, we utilize the corresponding input (normalized expression data), validation (ground truth GRN), and predictions (SOTA cell-type GRNs) from a study (McCalla *et al.* 2023) that benchmarked GRN methods in various contexts; we focus on mDCs, hPSCs, and YCs. In all three applications, our five NetREm models (tuning β ∈ {0.01; 0.1; 1; 10; 100; 1,000}, α: LassoCV) demonstrate competitive performance against SOTA GRNs (Inferelator; knnDREMI; mean: LEAP, Pearson, PIDC, SILGGM, SCODE; MERLIN; Pearson; SCENIC; Scribe, RTN, GRNBoost2) and BRMs across various metrics (Supplementary Fig. S7A–C, Supplementary Tables S10–S14). In Fig. 3A, for each metric, we rank models relative to each other, where smaller ranks indicate superior outcomes. For example, in YCs, various NetREm models achieve the top rank (#1) in balanced accuracy (BACC), F1 score (harmonic mean of precision and recall), % of the intersection (PerInt), % of predictable TFs (a fine-grained measure of accuracy for individual TF target sets). Many studies (Pratapa *et al.* 2020, Seçilmiş *et al.* 2022, Alawad *et al.* 2023, Sha *et al.* 2024, Skok Gibbs *et al.* 2024) primarily emphasize AUPR (Area Under the Precision Recall Curve) for evaluating the global accuracy of inferred GRNs, due to the sparsity of true GRNs and the inherent class imbalance [predominantly negative, non-regulatory pairs (Liu *et al.* 2015)]. All 5 NetREm models consistently attain high performance in AUPR, specificity, accuracy, as well as low FPs, underscoring their strong ability to identify true regulatory links. While no single method universally surpasses others, our NetREm method is adaptable with strong performance across diverse metrics and biological contexts, illustrating its effectiveness and versatility. Importantly, NetREm uniquely infers TF-TF coordination networks, both TG-specific (B) and overall ($\bar{B}$), capabilities that are absent in other methods; while RTNduals also attempts TF-TF link prediction, it often fails to output $\bar{B}\;$links for some datasets (e.g. PBMCs, HSCs) and it does not infer B links (e.g. it cannot predict coordination specific to TG in Fig. 2).

#### 3.3.2 Evaluating context-specific TF-TF coordination networks B for individual TGs

We evaluate NetREm’s predictions of TF-TF coordination, B, as either cooperative or antagonistic in co-regulating their individual TGs (Supplementary Fig. S8A and B). TFs may exhibit moonlighting roles, with their PPIs (direct/indirect) and functions varying based on context (Drew *et al.* 2021) and specific TGs they co-regulate. NetREm predicts these individual coordination networks for each of the G TGs in the cell type for the provided context. To assess NetREm’s predictions, we obtain experimental findings for 11 cytokine TGs and 125 TFs derived from (Berenson *et al.* 2023), which uncovered cooperative/antagonistic TF-TF interactions by performing pY1H (paired yeast one-hybrid) assays to detect direct DNA binding to REs in immune-related contexts. We run NetREm across human immune cell sub-types from the Tabula Sapiens Atlas for these TFs and TGs, identifying 167 unique TF-TF links (94 cooperative, 73 antagonistic) involved in TG regulation [i.e. ($TF_{i}$-$TF_{j}$)-TG links] that are verified; this totals 361 unique interactions across 42 sub-types. For example, we accurately predict both cooperative interactions (FOS-JUN, FOS-FOSL2) regulating *IL6* in granulocytes [inflammatory white blood cells (WBCs) that respond to infection] and monocytes (type of WBC that differentiates into macrophages or dendritic cells), as well as three out of five antagonistic PPIs regulating *IL1B* in Dn4 thymocytes (precursors of T-cells in the thymus). In addition, we predict five of eight true TF-TF PPIs (one antagonistic, four cooperative) for regulating *CCL5* in double positive alpha-beta thymocytes (express both CD4 and CD8, crucial for T-cell development). We show the TG-specific nature of TF-TF PPIs, such as how ATF2-FOSL2 cooperate to co-regulate *TNF* but are antagonistic co-regulators of both *CXCL8* and *IL1B*. Furthermore, NetREm uncovers novel PPIs, such as the antagonism between MAFF and MBD3 to co-regulate *IL10* in CD8+ memory T-cells. These results underscore NetREm’s unprecedented ability to predict both established and novel cooperative and antagonistic TF-TF mechanisms, at the cell sub-type-level, for co-regulating individual TGs. With the dawn of advancing research, as the accuracy and coverage of PPIs expand, the benefits of using NetREm—with PPIs among TF predictors as prior knowledge—to demystify TF-TF coordination activities for gene regulation (not only for the overall cell/tissue type in a certain health condition but also for individual TGs within that context) are poised to only increase.

#### 3.3.3 Evaluating overall context-specific cell-type TF-TF coordination networks $\bar{B}$

We evaluate the performance of our $\bar{B}$ in mESCs, mDCs, and PBMCs using the STRINGdb Version 11 (V11) PPIN as input (proxy for outdated information on direct/indirect PPIs), categorizing TF-TF pairs into four groups based on their status in the V11 and the updated STRINGdb V12 PPINs: absent in both, present in both (TPs), removed in V12 (FPs), discovered in V12 (FNs). Top TF-TF links have higher $\left|\bar{B}\right|$. Welch 1-sided *t*-tests (*P*-adjusted < 0.05) compare $\left|\bar{B}\right|$ values across these four groups (Supplementary Tables S15–S20, Supplementary Figs S9A–D, S10A and B, and S11A–C). For instance, in mESCs, NetREm often reflects known PPIs, flags FPs to remove, and uncovers biological truths, nominating promising candidate PPIs for follow-ups. Overall, NetREm prioritizes known TPs and has potential to flag future TF-TF PPIs (FNs) that are currently unknown.

Further, we gauge whether NetREm can help contribute to the detection of cell-type-specific TF-TF PPIs, an ongoing challenge since most existing PPINs are global and organism-specific and are not cell-type-specific. We use the contextual IID that annotates known human PPIs for ≈243 biological terms. Primarily, this IID helps us validate if relatively strong coordination links (i.e. $\left|\bar{B}\right|\;$≥ 85) across applications tend to reflect context-specific annotation terms. Despite the IID not being cell-type-specific, it is still a helpful resource to show that top links in Microglia (Mic) and pooled SCs (Eraslan *et al.* 2022), are biologically relevant, enriched for nervous system (NS)-related terms (Supplementary Fig. S12A). Similarly, we find that strong TF-TF links in immune PBMC sub-types associate with immune-related terms; these links also typically comprise TFs that are experimentally supported markers for the relevant immune cell-types based on CellMarker 2.0 (Hu *et al.* 2023) (Supplementary Fig. S12B). Currently, there is no direct resource to help guarantee that top links belong to cell-type-specific PPINs. However, we can tentatively extrapolate that if NetREm’s links are context-specific and/or involve cell-marker TFs, they might also be indicative of cell-type specificity. This assumption positions NetREm as a potential pioneer in identifying cell-type-specific TF-TF PPIs, exploring new frontiers in the field.

Moreover, we benchmark NetREm’s performance against that of RTNduals. Both tools recognize that each TG in a GRN may link to many regulators based on direct and/or indirect interactions among TFs. We compare NetREm(β=1, α: LassoCV) run with 13 different human input PPINs (our 13 $\mathrm{NetRE}m_{\beta =1}$ models) against RTNduals, using the same given expression data and corresponding list of N context-based TFs for each of the 13 human contexts where RTNduals yields its k respective TF-TF links. We select our top k links (high $\left|\bar{B}\right|$) for our models. To determine the validity of the top predicted TF-TF links, we use a comprehensive Evaluation PPIN (Supplementary File S5) of direct and/or indirect PPIs as our current improvised gold standard. Ten $\mathrm{NetRE}m_{\beta =1}\;$models output a statistically significantly higher % of valid links (Fig. 3B), outperforming RTNduals in ≥10 of 13 contexts (Supplementary Table S21). We further motivate the role of PPINs as prior knowledge, showing how baseREm (NetREm (positive β≈0, α: LassoCV) yields poorer results as it lacks wisdom regarding any PPIs among predictors. On average (across the 13 contexts), all 13 $\mathrm{NetRE}m_{\beta =1}\;$models have fewer poor results (links that are not in the Evaluation PPIN: FPs and/or unknown), learning a greater % of actual PPIs compared to RTNduals and baseREm (Supplementary Fig. S13A and B); this further underscores NetREm’s unparalleled predictive prowess in prioritizing TPs and leveraging historical PPIs to forecast and discover actual PPIs that are not yet known in the existing input PPIN. This is encouraging as PPINs are largely incomplete: only a small fraction of ≈130,000–650,000 potential human PPIs have been identified in experiments (Stumpf *et al.* 2008, Venkatesan *et al.* 2009, Sevimoglu and Arga 2014) that may include FPs (Yu *et al.* 2020). A strength of NetREm is that it not only weights known PPIs, but also estimates coordination when it may not be possible to observe PPIs.

#### 3.3.4 Evaluating the robustness of NetREm’s context-based output for various input PPINs

Our comparative analyses suggest that for a fixed and meaningful β, NetREm’s overall outputs are robust to variations in the PPIN that is input. To demonstrate this, we use these 13 $\mathrm{NetRE}m_{\beta =1}\;$models with baseREm as a reference. These 13 input PPINs are culled from different resources and exhibit a range of diversity in their TF-TF PPIs, with average pairwise Spearman ρ correlations from 2.45e−5 to 0.85 (average ρ = 0.32) (Supplementary Fig. S14A). We conduct 91 pairwise comparisons among these 14 models, examining the similarity of their corresponding output [TF-TG (Supplementary Fig. S14B–D) and $\bar{B}$ (Supplementary Fig. S14E–G)] networks across the 13 human contexts.

### 3.4 Gene regulatory links between TFs and TGs in human mSCs and nmSCs

With emerging new single-cell epigenomic data from many human tissues, we can now model GRNs in novel contexts. We apply NetREm to analyze SCs, which play a pivotal role in maintaining, regenerating, myelinating, and supporting PNS neurons. SCs are derived from the neural crest and exhibit tremendous flexibility in myelination as well as in several other tissues; they also function as terminally differentiated cells that can reverse differentiation after nerve injury to aid nerve regeneration (Ma and Svaren 2018). Single-cell rodent NS studies reveal substantial diversity in SC differentiation status (Gerber *et al.* 2021, Yim *et al.* 2022). Two main SC phenotypes (or sub-types) are: (i) mSCs associated with larger diameter axons (>1 micron); (ii) nmSCs that wrap a bundle of smaller diameter axons (typically sensory axons), i.e. a Remak bundle. While mSCs envelop axons in myelin sheaths to enhance conduction speed, nmSCs support sensory axon function/interactions without forming myelin, contributing to overall nerve integrity.

Uncovering TF-TG regulatory mechanisms modulating cellular processes in mSCs and nmSCs may help us understand and treat debilitating nerve injuries, demyelinating disorders, hereditary neuropathies. Mutations affecting SC function are the most prevalent cause of demyelinating genetic neuropathy Charcot–Marie–Tooth disease (CMT) (Tao *et al.* 2019) and some affect major transcriptional TFs of SC differentiation like EGR2 and SOX10 (Srinivasan *et al.* 2012, Fröb and Wegner 2022). Both TFs are co-expressed in myelinating SCs, colocalizing at several core myelin regulatory elements (REs) (Jones *et al.* 2007, Poitelon *et al.* 2016). In fact, mutations in EGR2 may disrupt cooperative SOX10 binding to its TFBSs (LeBlanc *et al.* 2006) and its subsequent regulation of its TGs. SC regulation also involves TEAD1 and other Hippo regulators to govern shared TGs and orchestrate PNS myelination (Srinivasan *et al.* 2012, Lopez-Anido *et al.* 2016). Since no TFs are exclusively expressed by SCs, uncovering the TF-TF coordination networks crucial for SC lineage maturation and differentiation into mSCs or nmSCs also holds significance (Ma and Svaren 2018). However, distinct transcriptional regulatory networks coordinating SC function and underlying states and cell fates require a variety of TFs beyond EGR2 and SOX10 (Hung *et al.* 2015).

In response, we apply NetREm to each TG in mSCs and nmSCs using single-cell data for the human DRG. We derive prior GRNs using multi-omics data (Section 2), such as: scATAC-seq data on accessible DNA regions for the cell type, cis-candidate REs (cCREs) of chromatin interactions for the cell type, eQTL data, gene expression data, PPIs, TF binding profiles. To construct these initial candidate prior reference GRNs, we annotate open chromatin regions in adult human SCs with known RE peak-to-TG links (Zhang *et al.* 2021) and use motif-based analysis to predict TFs that may associate with these REs. By removing lowly expressed TFs in the corresponding training expression data, we create tailored prior mSC and nmSC GRNs of initial TF-RE-TG links. For each TG in an SC sub-type, we input its N TG-specific selected candidate TFs (from its respective prior GRN) to NetREm. In total, NetREm outputs 183,242 mSC and 277,541 nmSC total TF-TG links (Supplementary File S1) comprising: 221 TFs and 8,950 TGs in mSCs; 228 TFs and 5,207 TGs in nmSCs. Both SC sub-types share: 33,806 TF-TG links, 27,037 c*-signed-TF-TG links, 3,841 TGs, and 197 TFs. TF EGR2 is mSC-specific (Balakrishnan *et al.* 2020). We enhance our regulatory networks by overlaying them with prior GRN annotations, resulting in our finalized TF-RE-TG regulatory networks.

**Figure 4.**
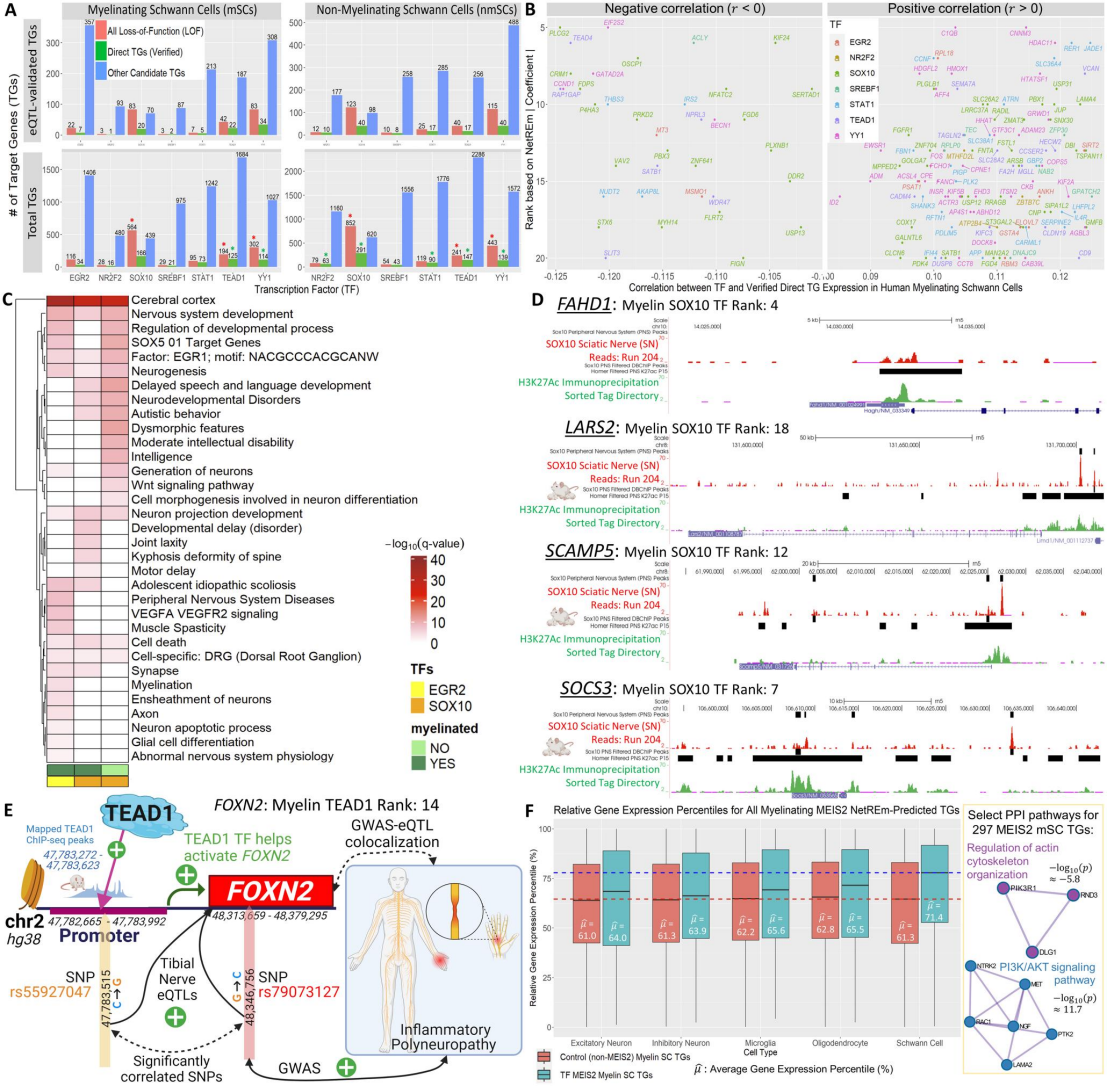
Gene regulatory links between TFs and TGs in human mSCs & nmSCs. (A) Bar plot categorizes TGs for core TFs in mSCs and nmSCs. Top: eTGs with eQTL validation. Bottom: original counts with star (*) for over-enriched TGs based on a hypergeometric test of overlap significance (*P*-adjusted < 5%). (B) Points: Direct TGs. *X*-axis: r between core SC TF and direct TG in train data. *Y*-axis: rank of TF for regulating the given TG in mSCs, where smaller ranks imply greater $\left|c^{*}\right|,\;$i.e. stronger relations. For simplicity, we show results where the TF is the top 5–20 for its direct TG. (C) Hierarchically clustered, select gene enrichments for all EGR2 mSC, SOX10 mSC, and SOX10 nmSC TGs; values: $-\log_{10}\left(q\right)$. (D) Rat epigenome tracks for four novel mSC SOX10-activated TGs with SOX10 as a top predictor TF. Tracks: rat sciatic nerve ChIP-seq peaks, SOX10 PNS peaks, histone modifications associated with enhancers (H3K27ac immunoprecipitation sorted tags). SOX10 is predicted to bind to REs in humans (based on prior input mSC GRN) to help activate these novel TGs. (E) Tibial Nerve eSNP rs55927047 [chromosome (chr) 2: 47,783,515 DNA change: C to G] in human *FOXN2* promoter (overlaps with orthologous TEAD1 rat ChIP-seq binding peaks) may strongly boost TEAD1 binding affinity to this *FOXN2* promoter to activate ($c^{*}$ > 0) *FOXN2*, a GWAS-eQTL colocalized biomarker for inflammatory polyneuropathy. *FOXN2* is a novel TEAD1 TG with support (via liftover analysis from rat to human reference genomes): TEAD1 rat ChIP-seq binding within 100 kilobases (kb) of its Transcription Start Site (TSS). (F) Left: Boxplots compare relative expression percentiles for all MEIS2 mSC TGs in GTEx pooled SCs with those in four CNS cell types. Median percentile for MEIS2 mSC TGs in SCs overall is 71.4 versus ≤ 61.3 in controls (non-TGs for MEIS2 in mSCs). Right: enriched PPI pathways for MEIS2 mSC TG proteins, which are important in SCs.

We examine NetREm’s results for eight core SC TFs that have validation [genome binding and loss-of-function (LOF)] data from rodent SCs: EGR2, NR2F2, RXRG, SOX10, SREBF1, STAT1, TEAD1, YY1. LOF TGs, whose expression varies upon TF knockdown, show their direct or indirect dependency on the TF (Nie *et al.* 2020). Valid direct TGs are a subset of LOF TGs with ChIP-seq (Chromatin Immunoprecipitation Sequencing) evidence of nearby TF binding, suggesting direct regulation by the TF (Titsias *et al.* 2012); this dual confirmation enhances our confidence in these direct TGs (Badia-I-Mompel *et al.* 2023). We lack LOF data for RXRG. In Fig. 4A, we provide counts of NetREm-predicted TGs: direct, all LOF, and novel candidates. We also report on eTGs with strong Tibial Nerve eQTL support (The GTEX Consortium 2020) based on mapped SNP-TF-RE-TG predictions: instances where an eSNP strongly alters TF binding to a RE, influencing TG expression. For example, we predict 2,015 YY1 TGs in nmSCs (139 direct; 304 LOF only; 1,572 novel), where LOF and direct TGs are significant (hypergeometric adjusted *P* < 0.05); of these, 40 direct (28.8%), 75 LOF only (24.7%), and 488 novel (31%) YY1 TGs are eTGs (expression TGs: based on eQTL support). In both SC sub-types, our final TF-TG predictions for all LOF TGs and direct TGs have higher sensitivity and F1 scores compared to LassoCV and ElasticNetCV networks, and higher accuracy and specificity than GRNBoost2, Linear Regression, and RidgeCV TF-TG regulatory networks (Supplementary Fig. S15).

We explore cases where NetREm accurately identifies a core SC TF as the top predictor (low rank) for its direct TG, even when training expression data shows a low r(TF, TG) (Fig. 4B). This is important as studies observe that in eukaryotes (unlike in prokaryotes), correlations r and mutual information among TFs and known TGs are not much higher than those between TFs and non-TGs (Phillips 2008, Zaborowski and Walther 2020, Escorcia-Rodríguez *et al.* 2023). For example, SOX10 weakly correlates with *SIPA1L2* [candidate modifier TG for CMT type 1A disease (Tao *et al.* 2019)] but is predicted to have rank 17. Despite its relatively weak r = −0.14 with *MBP* (myelin basic protein), a major constituent of myelin sheaths, STAT1 is its top 10 ranked TF. Although *APP* (Amyloid-beta precursor protein) exhibits a higher r with STAT1 compared to Fibrillin-1 gene *FBN1* (9.7e−2 versus 10.8e−2), NetREm ranks STAT1 higher as an activator for *FBN1* than for *APP* (13 versus 20). This aligns with findings of STAT1 LOF impacts of −0.61 for *FBN1* and −0.51 for *APP* (Xu *et al.* 2023).

NetREm reveals biologically relevant signals, identifying novel TGs for TFs. RXRG’s high regulatory activity in nmSCs (Supplementary Table S22) is consistent with rodent/human studies (Gerber *et al.* 2021). All TGs across three groups (EGR2 mSC, SOX10 mSC and nmSC) are enriched for PNS-related terms (Fig. 4C). We find 2,316 SOX10 TGs (159 shared; 428 direct; 1,314 LOFs overall) with 29 direct and 103 LOFs overall in both SC sub-types. Figure 4D shows rat nerve epigenome tracks for four novel SOX10 candidate mSC TGs (neither LOF nor direct TGs of SOX10: *FAHD1, LARS2, SCAMP5, SOCS3*) with strong SOX10 binding to SC regulatory regions in open chromatin (Lopez-Anido *et al.* 2015). We show corresponding data on accessible chromatin in adult SCs and SOX10 binding regions in humans for these four TGs in Supplementary Fig. S16A–D. In Fig. 4E, SNP rs55927047 enhances TEAD1 binding to its TFBS on *FOXN2*’s promoter to help activate transcription of *FOXN2* (Forkhead Box N2) in adult mSCs. Orthologous rat nerve TEAD1 ChIP-seq peaks also overlap with this promoter region. This eSNP correlates strongly [is in linkage disequilibrium (LD)] with SNP rs79073127 that links to higher inflammatory polyneuropathy risk in the Pan UK Biobank Genome-wide Association Study (GWAS) database (Turley *et al.*). *FOXN2* GWAS-eQTL colocalizes for this condition, with 75% probability (Wallace 2020). Both SNPs correlate with increased *FOXN2* expression.

We highlight additional TFs in SCs, underscoring their significant roles despite limited validation. Notably, our 297 mSC TGs for MEIS2 [core TF in DRG sensory neurons (Roussel *et al.* 2022)] are prominent in PPI pathways like: PI3K-Akt signaling [crucial for PNS myelination (Ishii *et al.* 2021)], actin cytoskeleton organization [essential for PNS regeneration (Wang *et al.* 2018)] (Fig. 4F). In comparison, these TGs have higher expression in pooled SCs than in any of the four CNS cell types (1-sided t, *P*-adj < 2e−6), a pattern absent in controls. Supplementary Figure S17 showcases principal hub TFs like EGR1 (485 nmSC eTGs) and RXRA (971 eTGs), which regulate the most eQTL-validated eTGs in SCs.

### 3.5 Coordination among TFs for gene regulation in human mSCs and nmSCs

**Figure 5.**
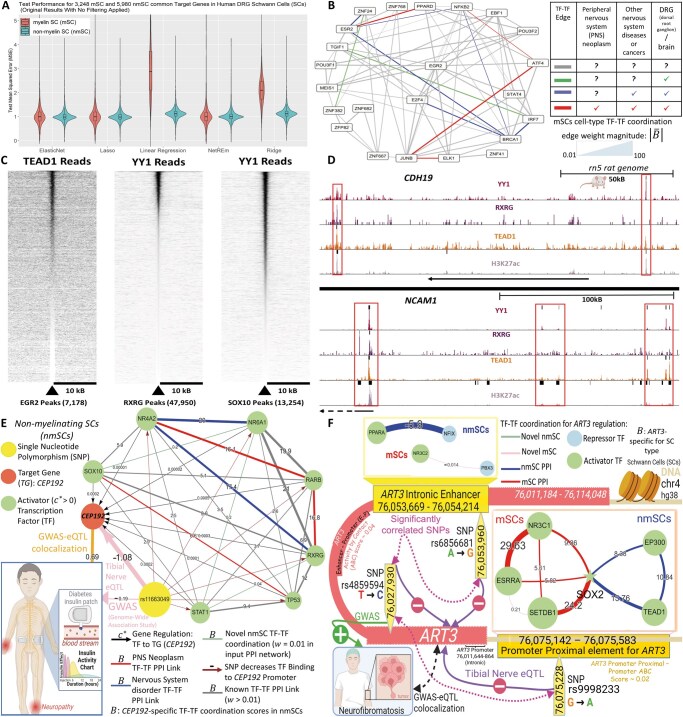
Coordination among TFs for gene regulation in human mSCs & nmSCs. (A) Density boxplots: NetREm outperforms linear regression and RidgeCV in both SCs, with lower test MSEs. NetREm predicts links for more TGs than ElasticNet and Lasso do. For mSCs, NetREm achieves median MSE: 0.95; nmSC: 1.1. (B) Input PPI subnetwork for only known TF-TF PPIs among 23 of 24 mSC-specific TFs; artificial (novel) links excluded. Edges: cell-type TF-TF cooperation ($\bar{B}$) across TGs in mSCs since $\bar{B}$ > 0 for all. High $\bar{B}\;$= stronger cooperation. Edges are annotated based on the Contextual IID. (C) Cut&Run heatmaps show overlaps of TEAD1 centered on EGR2 peaks, YY1 C&R centered on RXRG peaks, YY1 C&R centered on SOX10 peaks. (D) YY1, RXRG, and TEAD1 C&R tracks along with ChIP-seq H3K27ac tracks from rat S16 SC line. Boxes highlight enhancer regions where TFs colocalize across TGs: *CDH19* (top), *NCAM1* (bottom). Both TGs are impacted by Tibial Nerve eSNPs altering regulatory TF binding at their loci. (E) *CEP192* in nmSCs GWAS-eQTL colocalizes with low risk of polyneuropathy in diabetes. Tibial Nerve eSNP associates with lower risk of this condition and lower *CEP192* expression by potentially strongly disrupting bindings of six activators to *CEP192* promoter. SOX10 cooperates with them. *CEP192*’s B links are colored based on the Contextual IID: novel (not found in input PPI network); known TF-TF PPIs: PNS Neoplasms, NS disorders, neither. (F) Tibial Nerve eSNPs potentially influence the regulation of GWAS-eQTL colocalized neurofibromatosis TG *ART3* through activators and repressors that form distinct TRMs (transcriptional regulatory modules) in mSCs and nmSCs along interacting REs (3D DNA loop) in open chromatin. *ART3*’s promoter has Activity by Contact (ABC) scores (Zhang *et al.* 2021) of ≈0.04 with the intronic enhancer, ≈0.02 with the proximal promoter regulatory element (RE). TF-TF B edge widths are shown relative to other TF-TF B links in that given *ART3* RE (i.e. intronic enhancer, promoter proximal).

Our test MSEs are significantly lower than those of Linear and Ridge BRMs (Fig. 5A, Supplementary Table S23). NetREm predicts SC-type-specific coordination B for each TG. It also outputs 22,809 mSC and 24,795 nmSC non-zero direct/indirect TF-TF coordination $\bar{B}$ links (Supplementary File S2). Notably, top IID contexts for strong mSC and/or nmSC $\left|\bar{B}\right|\;$relate to the DRG and brain. Figure 5B shows 23 of 24 mSC-specific TFs in a mSC $\bar{B}$ network of 77 known PPIs (w > 0.01), where novel links are excluded for visual simplicity (corresponding figure for nmSCs: Supplementary Fig. S18A); POU3F1-EGR2 mSC cooperativity is extremely strong ($\bar{B}$ = 99.14, i.e. 99.14 percentile for mSCs), JUNB-ATF4 ($\bar{B}$ = 96.98) interact in PNS neoplasms like Schwannomas. BNC2’s regulatory activity in nmSCs may be attributed to the absence of its predicted repressor EGR2, which is ranked 10 of 20 mSC TFs for *BNC2*.

NetREm discovers and prioritizes novel TF-TF coordination links that are promising. 48 of our novel links in nmSCs (37 are also in mSCs), comprising 30 TFs, are validated by strong SAINT scores of physical TF-TF binding in recent BioID (proximity-dependent biotin identification) and/or AP-MS (Affinity Purification-Mass Spectroscopy) human experiments (Göös *et al.* 2022) (Supplementary Fig. S18B). RXRG, STAT1, TEAD1, and YY1 co-regulate 174 TGs in nmSCs and *SETD2* in mSCs (Supplementary Fig. S18C), suggesting their preferential coordination in nmSCs. RXRG’s PPI links with STAT1, TEAD1, and YY1 are unknown in our main input PPIN (Supplementary Fig. S19A). RXRG strongly positively correlates with TEAD1 and YY1 (r = 0.5, 0.7) in nmSCs, but negatively in mSCs (r = −0.9, −0.7) in mouse sciatic nerves, correspondingly (Gerber *et al.* 2021). STAT1 has 591 [Jaccard Similarity (JS) score: 0.21], YY1 has 599 (JS: 0.20), TEAD1 has 843 (JS: 0.26, significant) co-regulated nmSC TGs with RXRG (Supplementary Fig. S19B and C). In nmSCs, RXRG and YY1 share 94 eSNPs, 95 eTGs, 88 eQTLs compared, respectively, with 28, 54, 24 for RXRG and STAT1 [cooperate for 704 TGs (Supplementary Fig. S19D), antagonistic for 104 TGs (Supplementary Fig. S19E)]; RXRG-cooperation $\bar{B}$ is: TEAD1 (16.3), STAT1 (23.5), YY1 (24.1) (Supplementary Fig. S19F).

To independently test our predicted TF-TF coordination for these core SC TFs, we use binding data from rat SCs in the PNS, derived from ChIP-seq analysis of the active enhancer H3K27ac histone mark and ChIP-seq/Cut&Run read density assay (C&R) data of TF binding in nerve or S16 SC cell lines. Of 15,864 ChIP-Seq H3K27 enhancer peaks shared between PNS and S16 lines, RXRG shares 3,450 and 2,017 peaks with YY1 and TEAD1 (Supplementary Fig. S19G). 43.9% of RXRG peaks have YY1 binding (most colocalization among our core SC TFs) and 25.7% of RXRG peaks have TEAD1 binding (Supplementary Fig. S19H). Conversely, 24% of TEAD1 peaks and 28% of YY1 peaks have RXRG binding. C&R helps determine the extent of colocalized binding along REs. TEAD1 C&R centered across EGR2 peaks reveals that TEAD1 colocalizes at ≈40% of EGR2 TFBSs (Fig. 5C), supporting predicted EGR2-TEAD1 coordination in mSCs. YY1 C&R reads overlap ≈70% when centered on RXRG peaks and SOX10 peaks. Both *CDH19* and *NCAM1* are established marker genes for SCs (Hu *et al.* 2023). *CDH19* is preferentially expressed in nmSCs (Stratton *et al.* 2017) and has RXRG ChIP-seq binding nearby. Tibial Nerve eQTL SNP rs17799413 may be associated with lower *CDH19* expression (slope: 2.4e−1), strongly altering binding of TEAD1 in both SC sub-types and 7 core TFs (e.g. RXRG, YY1) in nmSCs. C&R sequencing unearths TEAD1 and RXRG binding peaks that are 40 kb upstream of *CDH19*’s Transcription Start Site (TSS) (Fig. 5D). *NCAM1*, a YY1 LOF and SOX10 direct TG, codes for a neural adhesion molecule preferentially expressed in nmSCs (Martini and Schachner 1986) and upregulated in the brain after lung tumor metastasis (Wang *et al.* 2022a). Our network predicts regulation of *NCAM1* by TEAD1, YY1, and RXRG in nmSCs. Rs10749999 associates with higher *NCAM1* expression (slope: 9e−2) and may boost YY1 and TEAD1 binding in nmSCs. Active SC enhancers ≈130 and 200 kb upstream of *NCAM1*’s TSS have TEAD1 and YY1 binding. *NCAM1* has TEAD1, RXRG, and YY1 binding at a promoter surrounding its gene locus. Rat H3K27Ac and TF ChIP-seq data shows that RXRG co-regulates: 555 TGs with TEAD1, 352 TGs with YY1, and 27 of 91 mapped STAT1 TGs. This further supports our predicted coordination by these 4 TFs, showcasing NetREm’s prowess in identifying novel colocalizing TFs in the absence of current evidence of direct binding interactions from high-throughput studies of PPIs.

Predicting causal pathways linking genotype to phenotype remains a challenge (Liu *et al.* 2022, Chandrashekar *et al.* 2023). Computational methods can help decipher the functional impacts of SNPs on PPIs, aiding in uncovering disease risk genes for targeted precision therapies. Most noncoding, disease-associated SNPs alter human PPIs rather than protein properties like folding or stability (Sahni *et al.* 2015, Chen *et al.* 2018, Kwok-Shing *et al.* 2018, Cheng *et al.* 2021). Integrating TF-TG regulatory ($c^{*}$) and B networks with noncoding eSNP rs11663049 sheds insights on how PPIs associate with this phenotype: polyneuropathy in diabetes (Fig. 5E). Dysregulated mitotic checkpoint regulators may lead to abnormal insulin signaling in diabetes (Choi *et al.* 2016). *CEP192* helps form mitotic spindles (Joukov *et al.* 2010) and colocalizes for this phenotype with 69% probability. The eSNP reduces the risk of this phenotype and is tied to lower *CEP192* expression, potentially by strongly decreasing binding of 6 activator TFs to *CEP192*’s promoter in nmSCs. In fact, *CEP192* is a TG that is co-regulated by both RXRG and STAT1 TFs. RXRG-NR4A2 and NR4A2-THRB associate with PNS neoplasms. SOX10, with its dynamic, cell-type-specific cooperation, works with these TFs; in fact, SOX family TFs achieve cell-type-specificity via partner TFs that facilitate TG regulation by binding to nearby SOX TFBSs (Stevanovic *et al.* 2021). Overall, we find that SOX10-NFIA cooperate in nmSCs ($\bar{B}$ = 81.3) but display antagonism when influencing glial lineage diversification (Glasgow *et al.* 2014) in mSCs ($\bar{B}$ = −78.7).

To illustrate differences in TG-specific *B* coordination networks between SC sub-types, even for shared TGs, we examine TG *ART3* (ADP-Ribosyltransferase 3) that colocalizes with neurofibromatosis (NF: characterized by the formation of NS-related tumors) with a 68% probability (Fig. 5F). We predict an interacting DNA chromatin loop (Zhang *et al.* 2021) of REs in open chromatin in adult SCs, featuring differing B between mSCs and nmSCs, involving strong repressors and activators (Sharov *et al.* 2022) to regulate *ART3*. Three eSNPs link to lower *ART3* expression: NF-associated rs4859594 correlates with two regulatory SNPs (rs6856681: located in a predicted intronic enhancer; rs9998233: in a promoter proximal RE) that may disrupt coordination networks by strongly decreasing activator binding to *ART3’s* REs, while strongly increasing it for repressors. Both SC sub-types have SOX2 as a common TF; it is a core regulator of SC myelination and myelinating disorders, and is a super pioneer TF associated with cancer cell proliferation and survival (Benedetti *et al.* 2022). SOX2 coordinates with TFs at a proximal promoter regulatory region for *ART3*, eagerly cooperating with: TEAD1 (B = 15.76) in nmSCs, SETDB1 (B = 24.2) in mSCs. PPARA-NFIX (nmSCs) and NR3C2-PBX3 (mSCs) are antagonistic TF-TF relations on the enhancer.

### 3.6 Prediction and comparative analysis of cell-type coordination among TFs for gene regulation across neuronal and glial cell types in AD

Cell-type TF-TF coordination networks $\bar{B}$ are crucial for neuronal functions like neurotransmission and synapse plasticity, and are disrupted in Alzheimer’s disease (AD), leading to memory loss, neuroinflammation, cognitive decline (Mathew *et al.* 2022). Understanding how altered $\bar{B}$ impacts TG expression in AD is essential for identifying master regulators and developing targeted therapies (Wang *et al.* 2016). Signaling PPIs associated with dementia symptoms highlight the potential of targeting these altered PPIs (Mao *et al.* 2020) to delay AD progression (Vargas *et al.* 2018, Gupta *et al.* 2022b). In response, we integrate multi-omics data to construct 16 context-specific prior candidate GRNs (initial TF-RE-TG links) for 8 cell types in AD and in Controls. For each TG in a context, NetREm uses the N TG-specific candidate TFs from the respective prior GRN as input features, based on TFs that may associate with its REs to regulate it (Section 2). Ultimately, NetREm generates 16 corresponding final TF-RE-TG (TF-TG links: Supplementary File S3) and $\bar{B}$ (Supplementary File S4, word cloud of top cell-type TF-TF links: Supplementary Fig. S20A) networks. Reverse engineering changes in these regulatory and coordination networks across cell types may help illuminate molecular drivers of AD.

We evaluate the biological network properties of our predicted TF-TG regulatory networks by comparing NetREm with scNET (Gupta *et al.* 2022a), which applies the scGRNom tool to generate TF-RE-TG networks on the same preprocessed expression data for four out of eight cell types in AD and in Controls. Across all eight contexts, NetREm demonstrates superior scale-free topology (Supplementary Table S24), with power law degree exponent (γ) nearer to the ideal 2–3 range and higher coefficient of determination (*R*^2^) that is closer to 1 (Broido and Clauset 2019, Chen *et al.* 2023). To assess NetREm’s biological relevance and predictive accuracy, we use the high-resolution brainSCOPE atlas (Emani *et al.* 2024) as a proxy for signed ground truth cell-type-specific GRNs in Controls. We compare TF- $c^{*}$ sign-TG regulatory links predicted by NetREm and scNET in Control Mic and Oligodendrocytes (Oligo), carefully filtering the respective networks for proper comparison (details: Supplementary File S5). NetREm outperforms scNET with 59.6% and 23.3% higher JS scores in Mic and Oligo, respectively, and achieves better signed average AUPR metrics in both Mic (0.687 versus 0.645) and Oligo (0.713 versus 0.648) (Supplementary Table S25). Our comparative analyses portray NetREm’s strengths in predicting meaningful TF-TG transcriptional regulatory interactions across different cell types and disease contexts.

**Figure 6.**
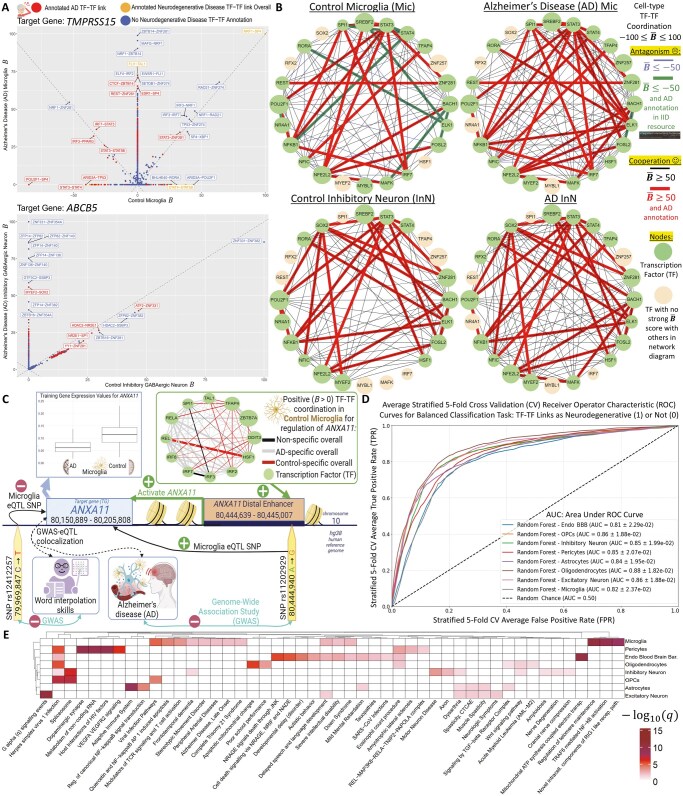
Prediction and comparative analysis of cell-type coordination among TFs for TG regulation across neuronal and glial cell types in AD. (A) Coordination scores B for 2 AD risk TGs, *TMPRSS15* for Mic and *ABCB5* for InNs, in Control versus AD. We color points to highlight TF-TF links annotated by the Contextual IID for AD or other neurodegenerative diseases. The default points have no current known annotation for neurodegeneration. (B) Circular network diagrams visualize strong cell-type direct/indirect TF-TF coordination in AD/Control Mic and InNs, focusing on TFs that exhibit potentially strong: cooperation (50 $\leq \bar{B}$  ≤100) or antagonism (−100 $\leq \bar{B}$  ≤-50). Thick edges are AD-related in the IID. We use a lighter color to show TFs not expressed or lacking strong links with other TFs in this visual. (C) 12 TFs that may cooperate to activate *ANXA11* based on NetREm’s final TF-RE-TG regulatory network in Control Mic (higher expression for *ANXA11* than in AD). Widths for TF-TF links are B scores for *ANXA11* regulation in Controls and colors are based on the statistical significance of TF-TF coordination links across all TGs in both Mic networks. There are 3 colors for the TF-TF links based on if they are statistically significant (higher in AD or higher in Controls) or not. A SNP correlating with lower AD susceptibility, increases binding of this TF-TF network to an enhancer, and links to higher *ANXA11* expression. Further, a risk SNP for reduced cognitive ability (poor word interpolation skills) links to lower *ANXA11* expression. (D) Average stratified 5-fold CV (Cross Validation) ROC (receiver operating characteristic) curves for RF models predicting TF-TF links annotated for neurodegenerative diseases (class 1) or not (class 0) for balanced classes. (E) Enrichment analysis for top 500 optimal genes identified by RF for each of 8 cell types. Hierarchical clustering is performed on rows (cell types) and columns (terms); $-\log_{10}\left(q\right)$ values are shown.

We explore TG-specific coordination B in Control versus AD stages for two AD risk genes (Jia *et al.* 2021, Bossaerts *et al.* 2022) (Fig. 6A): *ABCB5* in Inhibitory Neurons (InNs) (*t*-test statistic: 22.6, *P*-adj < 4.5e−112) and *TMPRSS15* in Mic (*t*: 36.1, *P*-adj < 4.8e−283); both TGs show notable increases in B in AD versus baseline Controls for the respective cell type. Some TF-TF pairs strongly cooperate (i.e. 50 ≤B≤100) exclusively in AD, such as: ZBTB14-ZNF281, FLI1-TAL1 for *TMPRSS15*; ZNF331-ZNF354A, MYEF2-SOX2 for *ABCB5*. Known AD-annotated links IRF7-STAT3 and STAT3-STAT5B have strong antagonism (i.e. −100 ≤B≤-50) in Controls but cooperate in AD to co-regulate *TMPRSS15*. Figure 6B compares strong $\bar{B}$ among select TFs between conditions for Mic and InNs. IRF7-STAT3 is in Mic, MYEF2-SOX2 is in InNs only. AD link STAT3-STAT4 is antagonistic in Controls for *TMPRSS15* in Mic and in Control Mic overall (but is cooperative for the other three networks). RORA-ELK1 is cooperative in all three networks but is antagonistic in Control Mic. FOSL2-BACH1 is antagonistic in Mic but cooperative in InNs. Indeed, RORA activity increases in InNs and Mic in AD (Acquaah-Mensah *et al.* 2015). AD-annotated links in AD InNs/Mic include ELK1-SPI1 and ELK1-STAT3.

Figure 6C presents a multifaceted network weaving together TF-TG links, coordination, phenotypes, SNPs. TF-TF coordination is discernibly stronger and positive in Control Mic (1-sided *t*-test), pointing to potentially disrupted cooperativity during AD. Our attention is drawn to the regulation of *ANXA11*, a critical player in diverse functions (e.g. apoptosis, neutrophil function) and signaling pathways (e.g. MAPK, P53) (Mirsaeidi *et al.* 2016). Mutations in *ANXA11* are correlated with NS diseases (Wang *et al.* 2022b) and high risk of inflammatory conditions like sarcoidosis (Smith *et al.* 2017). Our previous work (Khullar and Wang 2023, Khullar 2024) identifies *ANXA11* as 1 of 36 AD-Covid risk candidate neuroinflammatory biomarker genes (Zhang *et al.* 2024) and assigns *ANXA11* to a Control gene co-expression module in the Hippocampus brain region. NetREm offers nuanced insights on noncoding SNPs associated with a reduced likelihood of AD (rs11202929) and with poor cognitive function (rs12412257), which are linked to greater and lesser *ANXA11* expression, respectively, in Mic (resident CNS macrophage immune cells). Rs12412257 associates with reduced word interpolation ability, a measure of fluid intelligence and reasoning and a prognostic marker of AD (Eyigoz *et al.* 2020). Rs11202929 is protective against AD (GWAS slope < 0) and may enhance the binding affinity of 12 cooperating activators to TFBSs on *ANXA11's* enhancer in open chromatin in adult Mic. SPI1 is a core Mic TF for AD genes (Rustenhoven *et al.* 2018). *ANXA11* has higher Mic expression in controls than in AD [*t*-test *P*-adj = 2.6e−68, $\log_{2}$(Fold Change) of (Control/AD) = 0.78; 668 AD, 676 controls]. Our significant GWAS-eQTL colocalization analyses reveals that *ANXA11* expression positively associates with lower AD risk and with better word interpolation ability. All in all, NetREm provides a powerful framework to deepen our understanding of complex GRNs and their implications across a spectrum of health conditions.

We further evaluate our $\bar{B}$ networks. Supplementary Figure S20B compares our 16 $\bar{B}$ networks for 216 novel links that are validated in recent physical human experiments (Göös *et al.* 2022). In control Astrocytes, ExNs, InNs, and Oligo, we find correlation r≈ {0.63, 0.54, 0.45, 0.50}, respectively, between the JS of ChIP-seq peak overlaps [proxy metric for cooperativity (Yu *et al.* 2015)] and our $\bar{B}$ scores for 5 TFs with ENCODE3 TF cluster data (Sloan *et al.* 2016) in neural cells (e.g. neurons, glial cells) (Supplementary Fig. S20A–C).

In addition, for each cell type, we build default Random Forest (RF) (Fig. 6D) machine learning models (Pedregosa *et al.* 2011) to detect TGs with altered B from Controls to AD that may predict TF-TF links annotated (in Contextual IID) with neurodegenerative disease (class 1) or not (class 0). Input data consists of changes in B across TGs from Control to AD, for the given cell-type. To tackle this positive unlabeled learning problem (Yang *et al.* 2014), we undersample class 0 so that each cell type has an equal count of TF-TF links in both classes. We evaluate performance via stratified 5-fold Cross Validation (CV) noting that all 8 of the RF cell-type models have an average area under Receiver-Operator Characteristic (ROC) curve (AUC) ≥ 0.81. Across our models, the top 500 RF TGs (highest feature importance) are enriched for neurodegeneration, cell-type functions, immunity, intellectual disabilities, tauopathy (Fig. 6E). OPCs and InNs have the highest overlap (35 top TGs in common); disrupted InN signaling to OPCs may diminish myelination and CNS interneuron activity, and severely impair prefrontal cortical network functions and social cognitive behavior (Fang *et al.* 2022) (Supplementary Table S26).

## 4 Discussion

In this paper, we present NetREm, a computational multi-omics-based approach that employs network-regularized regression on single-cell gene expression data to predict cell-type coordination among TFs for TG regulation. NetREm addresses a major challenge in traditional studies of cell-type GRNs. Gene expression data, often nascent, sparse and noisy, fails to capture crucial GRN mechanisms such as TF binding to DNA, coordination among TFs/cofactors, and DNA accessibility, and it typically provides weak signals for distinguishing TP from FP TF-TG links (Badia-I-Mompel *et al.* 2023, Kim *et al.* 2023). Sole reliance on expression data for GRN inference is therefore woefully inadequate, and perhaps even futile, often leading to unstable and inaccurate results.

Explicitly modeling direct and indirect TF-TF interactions can enhance GRN inference, enabling the discovery of novel TF-TG links and key cell-type TFs (Skok Gibbs *et al.* 2022). Functionally related TF predictors, like neighbors in scale-free feature networks (e.g. TF-TF PPINs), can coordinate synergistically or antagonistically in biological processes like TG regulation (Kong and Yu 2018). Nonetheless, traditional methods oft miss such complex dynamics of TF-TF PPIs involved in GRNs.

SOTA GRN tools like SCENIC indirectly hint at TF-TF interactions by analyzing TFs that co-regulate multiple TGs. However, such tools primarily focus on TFs with strong motif binding, excluding other prior information. For instance, in our comparative analysis, SCENIC identifies many TFs in SCs (nmSCs: 640, mSCs: 522 TFs) but overlooks TEAD1 in both SC sub-types, instead detecting three other TEAD family TFs. TFs like TEAD1, which exhibit relatively weak motif-binding signals, are drowned out. In contrast, NetREm, which incorporates TF-TF PPINs, effectively captures essential GRN relations for core SC regulator TFs like TEAD1. This underscores the importance of integrating comprehensive prior insight like PPINs, in GRN predictions from expression regression, a capability that NetREm successfully implements (Dibaeinia and Sinha 2020).

NetREm reveals cell-type coordination among TFs, $\bar{B}$, with some network links mediated by physical and others by indirect (e.g. pioneer/settler models show TFBSs are often >50 bp apart in REs) PPIs. By weighing known direct and/or indirect PPIs in the context of TG regulation, NetREm helps characterize existing PPINs at a cell-type level, a current challenge in biology (Johnson *et al.* 2021, Hsu *et al.* 2022, Murtaza *et al.* 2022). It also helps address the link prediction problem (Singh and Vig 2017), flagging undiscovered PPIs for follow-ups. The lack of cell-type PPI annotations, while a challenge (Yu *et al.* 2023), offers NetREm an opportunity to contribute to ongoing efforts (Kupershmidt *et al.* 2024) to broaden our understanding of PPIs and protein dynamics with its dual capacity to annotate known TF-TF PPIs and discover novel cell-type-specific ones.

NetREm predicts unprecedented overall cell-type $\bar{B}$ networks across various conditions, including human, mouse, and yeast contexts, even in the absence of prior, reference GRN information. Benchmarking shows that our TF-TG regulatory networks not only perform competitively with SOTA GRNs but also uncover novel cell-type TFs that co-regulate TGs; moreover, our $\bar{B}$ networks effectively prioritize TP and FN TF-TF links. Additionally, NetREm predicts unrivaled TG-specific B for cell types. With ongoing efforts in PPI prediction, validation, and annotation, we are optimistic that more context-specific PPINs will become accessible, allowing NetREm to leverage these improvements and further enhance the quality of our outputs.

Disrupted cell-type PPIs are critical in neurobiological disorders, since PPIs mediate neuronal functions (Mathew *et al.* 2022) and beyond. We integrate multi-omics data, capturing various levels of TG regulation (e.g. scRNA-seq, scATAC-seq), to learn prior, reference GRNs for nervous system (NS) cell types for our SC and AD human applications. Detecting these candidate GRN TFs for TGs aids NetREm in inferring biologically significant cell-type TF-TG links (Zaborowski and Walther 2020, Zhang *et al.* 2023). Aligning NetREm’s TF-TG links with these prior reference GRNs helps us deduce TF-RE-TG links. NetREm uncovers novel TF-TF crosstalk for TG regulation in SCs and during AD in neurons/glia. We also demonstrate how our networks may facilitate identification of novel biomarker TGs for diseases. To this end, we obtain TF-TF links annotated with neurodegenerative diseases. Then, we compute changes in cell-type TF-TF coordination network embedding values between Control and AD across TGs as input to RF models that identify candidate cell-type biomarker TGs for neurodegeneration.

Insights derived from NetREm may contribute to advancing targeted therapies and regenerative medicine. We apply our predicted regulatory and coordination networks to trace how noncoding eSNPs may alter co-regulatory dynamics among TFs, potentially altering the expression of disease-associated eTGs. We present examples of GWAS-eQTL colocalized disease risk SNPs altering TRMs (TF-TF coordination subnetworks) and TF-TG regulatory networks associated with disease-associated TGs. To validate top findings, we use available functional genomic data in humans, rats, or mice (e.g. eQTLs, GWAS, Cut&Run sequencing, ChIP-seq data, knockout studies).

NetREm expands upon previous work in network regularized regression by learning and generating embeddings using SVD. In the future, we can incorporate nonlinear dimensionality reduction into NetREm to capture nonlinear patterns and regularize latent representations with prior information. We may see if any of our N* final TFs for a given TG form homodimers (e.g. $TF_{i}-TF_{i}$) or adapt NetREm to account for this. In addition, NetREm can integrate other information, like signaling pathways, to learn TF-TF coordination for TG regulation. Beyond expression regression, we can extend NetREm to other emerging single-cell omics like scATAC-seq data to explore TF and chromatin interactions in open regions. In fact, Supplementary Table S27 shows potential extrapolations of the NetREm framework to help tackle several other biological problems (e.g. modeling epistatic interactions among SNPs that influence complex traits/disease). In conclusion, NetREm extends to any discipline where predictors exhibit a network structure that informs the continuous outcome.

## Supplementary Material

vbae206_Supplementary_Data

## Data Availability

The data underlying this article are available in the article and in its online supplementary material.
