# Supplementary material for: NetREm: Network Regression Embeddings reveal cell-type transcription factor coordination for gene regulation
Source: Bioinform Adv. 2024 Dec 20;5(1):vbae206. doi: 10.1093/bioadv/vbae206 (PMC12011367; doi:10.1093/bioadv/vbae206)
Supplement: vbae206_Supplementary_Data [file vbae206_supplementary_data.zip › cb04a_Supplementary Materials.zip!/Supplementary Materials/Supplementary File 5.pdf]

Supplementary materials for:

NetREm Network Regression Embeddings reveal cell-type transcription factor coordination for gene regulation

Saniya Khullar[1,2], Xiang Huang[1], Raghu Ramesh[1,4], John Svaren[1,5], Daifeng Wang[1,2,3,*]

Table of Contents

Table of Contents 1

Supplementary Figures 7

Supplementary Figure 1 – Metrics for NetREm's cell-type Transcription Factor (TF) – Target Gene (TG) Gene Regulatory Networks (GRNs) and TF-TF coordination networks for real-world human applications 8

Supplementary Figure 2 – Simulation Study 9

Supplementary Figure 3 – Analysis of NetREm's performance based on 1,000 simulations 12

Supplementary Figure 4 – Under-the-hood: Simulation Study adapted for a case where predictors $N > M$ cells (samples). 14

Supplementary Figure 5 – Evaluating TF-TG Regulatory Networks in simulated Human Embryonic Stem Cells (hESCs) 17

Supplementary Figure 6 – Highlighting NetREm's Grouped TF Selection Property (prioritizing TF-TF relations in PPIN) using 4 Target Genes (TGs) in human Hematopoietic Stem Cells (HSCs) 19

Supplementary Figure 7 – Analyzing NetREm's Predicted TF-TG Regulatory Links in Normalized: Mouse Dendritic Cells (mDCs), Human Pluripotent Stem Cells (hPSCs), and Budding Yeast Cells (YCs). 22

Supplementary Figure 8 – Validating TG-Specific TF-TF Coordination Links in Human Immune Cell Subtypes Using Experimental Data on Paired Yeast One-Hybrid (pY1H) Assays 26

Supplementary Figure 9 – Analyzing NetREm's ability to prioritize and predict future Protein-Protein Interaction (PPI) Links in Mouse Embryonic Stem Cells (mESCs). 27

Supplementary Figure 10 – Analyzing NetREm's ability to prioritize and predict future Protein-Protein Interaction (PPI) Links in Mouse Dendritic Cells (mDCs). 29

Supplementary Figure 11 – NetREm TF-TF Coordination Performance for 9 different Human Immune Cell Types: PBMCs: Peripheral Blood Mononuclear Cells. 30

Supplementary Figure 12 – Evaluating the Context-Specificity of Annotations for Top TF-TF Coordination Links..... 34

Supplementary Figure 13 – Benchmark: Compare predicted TF-TF Links for RTNduals versus NetREm for various input PPI Networks 36

Supplementary Figure 14 – Evaluating the Robustness of NetREm's TF-TG Regulatory Networks and TF-TF Coordination Networks across 13 Human Contexts for Different Input PPI Networks (PPINs) 43

Supplementary Figure 15 – NetREm TF-TG regulatory link performance metrics for 7 core Schwann cell (SC) Transcription Factors (TFs) 44

Supplementary Figure 16 – Open Chromatin in human SCs and SOX10 predicted binding regions for 4novel SOX10-predicted TGs in human mSCs 45

Supplementary Figure 17 – Comparative barplot showing the top 14 Transcription Factors (TFs) with the highest # of eQTL-validated Target Genes (TGs) overall across myelinating (mSCs) and non-myelinating (nmSCs) SCs. 46

Supplementary Figure 18 – TF-TF Interactions and TF-TG Regulatory Networks in human nmSCs 47

Supplementary Figure 19 – Analyzing NetREm TF-TF predictions and validations for 8 core TFs in SCs 49

Supplementary Figure 20 – Comparing Coordination Scores Across Glial/Neuronal Cell-Types and Conditions: Information Content Word Cloud representations of Cell-type TF-TF Coordination Network Links $-100 \leq B \leq 100$ and Comparison of Novel TF-TF Links with Physical Binding Support 51

Supplementary Figure 21 – Analyzing NetREm TF-TF predictions and validations for Control Excitatory and Inhibitory Neurons based on Neural Cells for 5 TFs: CTCF, EP300, MXI1, RAD21, SMC3 52

Supplementary Tables 53

Supplementary Table S1 53

Supplementary Table S2 54

Supplementary Table S3 56

Supplementary Table S4 58

Supplementary Table S5 59

Supplementary Table S6 60

Supplementary Table S7 60

Supplementary Table S8 61

Supplementary Table S9 61

Supplementary Table S10 62

Supplementary Table S11 63

Supplementary Table S12 64

Supplementary Table S13 65

Supplementary Table S14 65

Supplementary Table S15 67

Supplementary Table S16 68

Supplementary Table S17 68

Supplementary Table S18 69

Supplementary Table S19 71

Supplementary Table S20 72

Supplementary Table S21 73

Supplementary Table S22 74

Supplementary Table S23 74

Supplementary Table S24 75

Supplementary Table S25 76

Supplementary Table S26 77
Supplementary Table S27 77
Supplementary Methods **79**
Section §1: Mathematical Methods Work for NetREm: 79
Pre-processing gene expression data: 79
§1.1 Integrating multimodal data & networks in NetREm workflow 79
§1.2 Step 1: Network regularized regression (Problem Definition) 79
§1.3 Step 2: Gene expression embeddings from network regression 79
§1.4 Output 1: Identification of novel cell-type TFs for regulating TG 82
§1.5 Output 2: Cell-type TF-TF coordination (direct/indirect interactions) 84
Section §2: Simulation Study: 85
§2.1 Biological Interpretation of Main Simulation in Figure 2: 86
§2.2 Simulation with various sparsity levels: 86
§2.3 Simulation with various M and N 86
§2.4 Testing Robustness 87
§2.5 Benchmarking β 87
Section §3: Introducing the 12 main applications of NetREm 88
§3.1 Diving into the 12 main applications of NetREm 88
§3.2 Example outputs by NetREm for a case-control (treatment versus control application) 89
Section §4: Single-Cell Gene Expression Data for 11 Main Applications (Apps) 90
§4.1: human Embryonic Stem Cells (hESCs): simulated 90
§4.2: human Hematopoietic Stem Cells (HSCs) 90
§4.3: mouse Embryonic Stem Cells (mESCs) 91
§4.4: mouse Dendritic Cells (mDCs) 91
§4.5: human pluripotent stem cells (hPSCs) 91
§4.6: budding yeast cells (YCs) 91
§4.7: human Peripheral Blood Mononuclear cells (PBMCs) 92
§4.8: Genotype-Tissue Expression (GTEx) Data for Schwann Cells 92
§4.9: Gene Expression data for 4 Neuronal/Glial Cells 92
§4.10: Human Immune Cell Sub-types 92
§4.11: Human myelinating (mSCs) and non-myelinating (nmSCs) Schwann cells (SCs) 93
§4.12: Alzheimer's disease (AD) vs. Controls in 8 Central Nervous System (CNS) Cell-Types 93
Section §5: Overview of Building out Final Comprehensive Protein-Protein Interaction Networks (PPINs): $\mathbb{W}$ 93
§5.1 Final Comprehensive PPI Networks (PPINs) in Humans 94
§5.2 Final Comprehensive PPI Networks (PPINs) in Mice 100

§5.3 Final Comprehensive PPI Networks (PPINs) in Yeast 101
§5.4 Rules for handling weighted versus binary (dummy) PPI Networks 101
§5.5 Overview of Input PPI Network Used for Different Analyses 101
Section §6: Overview of Building out Fully-Connected Comprehensive TF-TF Input PPINs for NetREm: W 103
§6.1 Fully-Connected Comprehensive TF-TF PPI Networks (PPINs) in Humans 104
§6.2 Fully-Connected Comprehensive TF-TF PPI Networks (PPINs) in Mouse 105
§6.3 Fully-Connected Comprehensive TF-TF PPI Networks (PPINs) in Yeast 105
Section §7: Prior Knowledge on Gene Regulation in Applications 10 and 11 105
Prior gene regulatory network (GRN) knowledge on gene regulation (overview) 105
Goal: Deriving Prior Gene Regulatory Network (GRN) for each Target Gene (TG) 107
§7.0 Organizing Input Data 107
§7.1 Constructing Motif-based Gene Regulatory Networks (GRNs) 109
§7.2 Pruning TFs down based on expression levels 111
§7.3 Pruning TFs down based on motif-score criteria 112
§7.4 TF Augmentation Step: 113
Section §8 NetREm Parameters for Applications 115
§8.1 NetREm in benchmarking applications with no prior GRN information 115
§8.2 NetREm with prior GRN information for TG-specific TFs 117
Section §9: General Overview of Benchmarking NetREm's Outputs 117
§9.1: Cell-type-specific TF-TG regulatory networks (output 1) 118
§9.2: TG-specific TF-TF Coordination networks for the cell-type (output 2) 120
§9.3: Overall cell-type-specific TF-TF coordination networks (output 2) 120
§9.4: Robustness of NetREm's aggregate context-specific outputs to changes in the input PPIN networks 120
Section §10: NetREm analysis for 13 different human contexts (in the Nervous System) 121
§10.1 Building the Evaluation PPI Network 121
§10.2 Gathering Input PPI Networks 122
§10.3 Defining Similarity for the Input PPI Networks 124
§10.4 Datasets used from the following studies/applications for the 13 Human Contexts 125
§10.5 Parameters used for 13 NetREm Models: NetREm$\beta = 1$ 129
§10.6 Baseline model used (baseREm) 129
§10.7 Comparison with RTNduals 130
§10.8 Pairwise comparisons for the Input PPI networks 130
§10.9 Analyzing the Robustness of NetREm's TF-TG Regulatory Networks 131
§10.10 Analyzing the Robustness of NetREm's Context-Specific TF-TF Coordination Networks 132
Section §11: Benchmarking for NetREm with No Prior GRN Knowledge 134

§11.1: Cell-type-specific TF-TG regulatory networks (output 1) 135
§11.2: Cell-type-specific TF-TF coordination scores B (output 2) 135
§11.3: SCENIC applied to human myelinating (mSCs) and non-myelinating (nmSCs) Schwann cells (SCs) in Dorsal Root Ganglion (DRG): 136
Section §12: Simulated human embryonic stem cells (hESCs) 137
§12.1: Motivation for using the SERGIO tool 137
§12.2: Signed ground truth gene regulatory network (GRN) for hESCs: 137
§12.3: Using the SERGIO Tool to Generate hESC gene expression data based on input ground truth GRN: 138
§12.4: Corresponding human input protein-protein interaction (PPI) network (PPIN): 139
Section §13: Overview of PBMC Data 140
§13.1: Cell classes in Cell Marker 2.0 140
§13.2: PBMC Sub-cell-type groupings: 141
Section §14: Evaluating PPI Predictions 142
§14.1 Validate TF-TF Coordination Link Prediction Scores for True Signals based on new PPINs 142
§14.2 Comparing NetREm Predictions with RTNduals and baseREm across 13 human contexts using an Evaluation PPIN 144
§14.3 Integrated Interactions Database (IID) for Contextual PPI Annotations 144
§14.4 Using Cut&Run data and ChIP-seq data to predict TF-TF colocalization and coordination 145
Section §15: Alzheimer's disease versus Controls: Additional Information for Application 12 145
§15.1 Comparing NetREm with scNET 145
§15.2 Evaluating if Predicted TF-TG Regulatory Networks Exhibit Scale-Free Topology 146
§15.3 Utilizing the brainSCOPE resource as a ground truth GRN in Control Microglia and Oligodendrocytes 146
§15.4 Comparing Jaccard Similarity of Signed TF-TG Regulatory Links between NetREm and scNET 146
§15.5 Signed Weighted AUPR Calculation for Regulatory Networks 149
§15.6 Neural Cell TF-clusters ChIP-seq tracks 150
Section §16: Gold Standard TG-Specific TF-TF Coordination 150
§16.1: Ground truth experimental data on TF-TF coordination for TG regulation 151
§16.2: Running NetREm on 62 filtered gene expression datasets for 62 immune cell subtypes 151
Section §17: Gold Standard Gene Regulatory Networks for the Main Applications 152
§17.1: human Embryonic Stem Cells (hESCs) 152
§17.2: human Hematopoietic Stem Cells (HSCs) 152
§17.3: mouse Embryonic Stem Cells (mESCs) 153
§17.4: mouse Dendritic Cells (mDCs) 153
§17.5: human pluripotent stem cells (hPSCs) 153
§17.6: budding yeast cells (YCs) 153
§17.7: Human myelinating (mSCs) and non-myelinating (nmSCs) Schwann cells (SCs) 153

§17.8: Alzheimer's disease (AD) vs. Controls in Central Nervous System (CNS) Cell-Types 154
Section §18: Training Comparative Methods for TF-TG Regulation (i.e. GRN Inference) 154
§18.1: The 4 Benchmark Regression Models (BRMs) 154
§18.2: GRNBoost2 154
§18.3: RTN (used by RTNduals) 154
§18.4: SCENIC (for mSCs and nmSCs) 155
§18.5: Output State-of-the-art (SOTA) cell-type GRNs from a benchmarking study 155
§18.6: scNET (application of the scGRNom tool) 157
§18.7: Overview of different types of models utilized 158
Section §19: RTNduals: Comparative Method for TF-TF Coordination 158
§19.1: Comparative Analysis Approach 159
§19.2: RTNduals method 160
§19.3: RTNduals may fail to yield results 160
§19.4: Applying NetREm and RTNduals for each context 161
Section §20: TF-TG Regulatory Network Evaluation using Gold Standard Networks 161
§20.1: Confusion matrix: 161
§20.2: Evaluation metrics: 162
§20.3: Area Under the Receiver Operating Characteristic Curve (AUC) and the Area Under the Precision-Recall Curve (AUPR) when Ground Truth Network has no weights: 163
§20.4: Calculating the % of Predictable TFs 164
Section §21: Additional Biological Resources for TF-TG Regulatory Network Evaluation & Downstream Analysis 165
§21.1 Expression quantitative trait loci (eQTL)-based validation 165
§21.2 GWAS-eQTL colocalization analysis 166
§21.3 Gene Enrichment Analysis 166
Section §22: Myelinating vs. Non-myelinating Human Schwann cells (Application 10): Additional details 166
§22.1 Experimental validation data for Schwann cells (SCs) 166
§22.2 Relative gene expression percentile analysis 169
§22.3 Hypergeometric test of TF-TG regulatory network overlaps with known TGs for TFs in SCs 169
§22.4: Validation data: Sciatic Nerve Atlas Data for Schwann Cells in Mice 170
Section §23: Software Implementation of NetREm 170
§23.1: Handling input gene expression data 170
§23.2: Running netrem and netremCV functions 171
§23.3: ElasticNetREm implementation: elasticnetrem function 172
§23.4: Handling directed input prior networks 172
§23.5: Running NetREm for a cell-type (many target genes) 172
Section §24: References 173

Supplementary Figures

A)

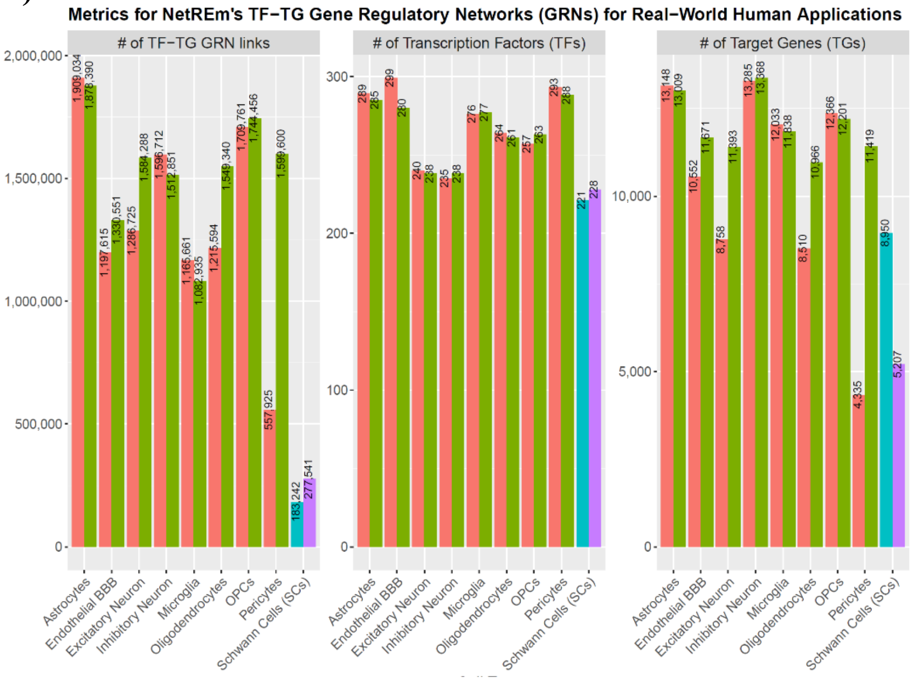

B)

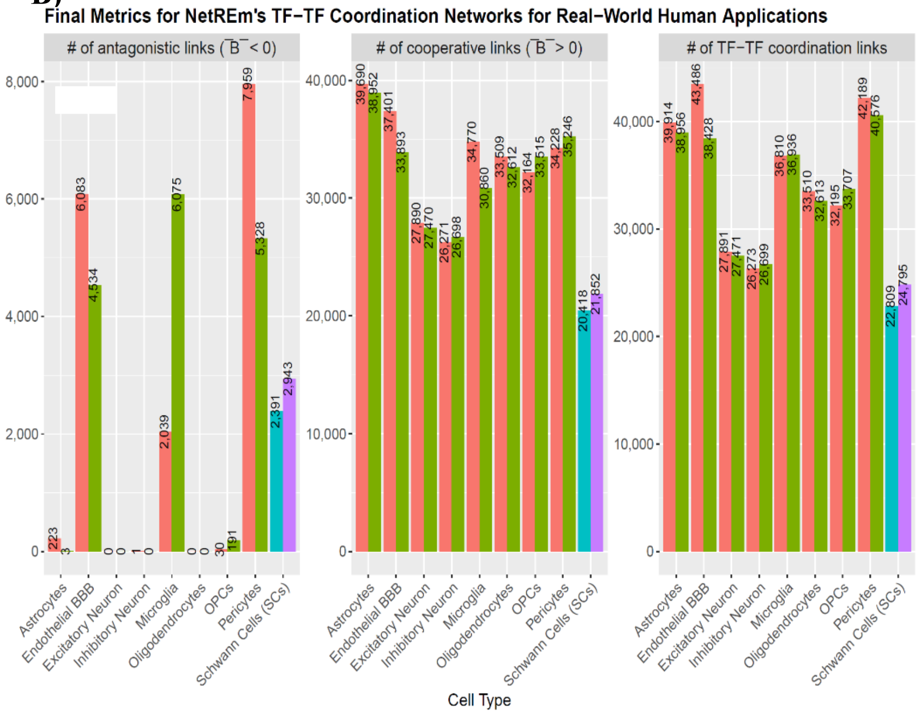

Supplementary Figure 1 – Metrics for NetREm's cell-type Transcription Factor (TF) – Target Gene (TG) Gene Regulatory Networks (GRNs) and TF-TF coordination networks for real-world human applications

We ran NetREm with prior GRN knowledge input: initial TF-regulatory element-TG links → each TG has a customized list of $N \leq \mathcal{N}$ TFs for 2 real-world applications:

- 2 Schwann cell (SC) sub-types: myelinating (mSCs) vs. non-myelinating (nmSCs)
- Alzheimer's disease (AD) vs. Controls: 8 different neuronal/glial cell-types

Please note that the corresponding **Table S4** provides details on these metrics. **A)** Output 1: Cell-type TF-TG GRN regulatory links. **B)** Output 2: Cell-type TF-TF coordination links ($\bar{B} \neq 0$)

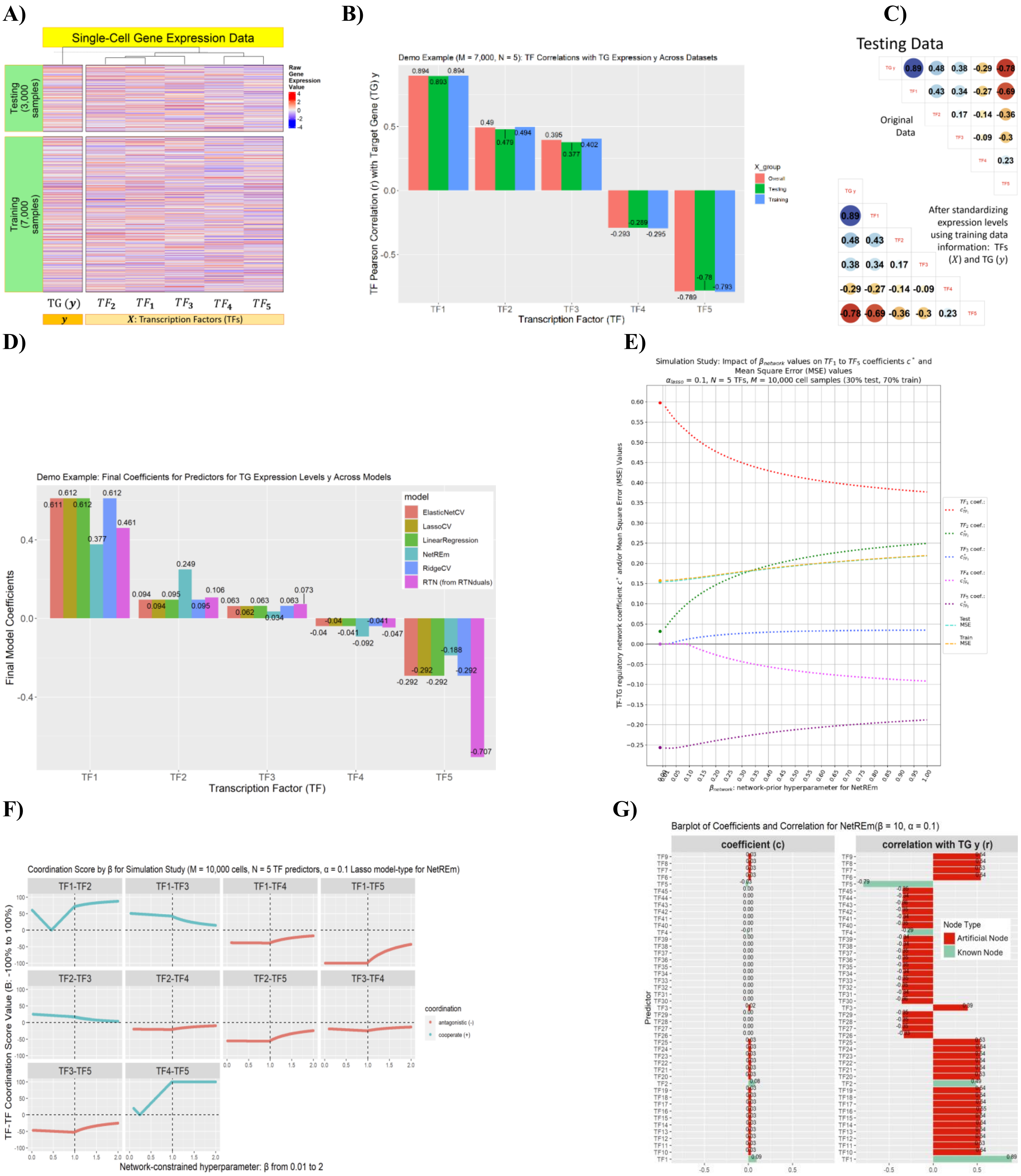

Supplementary Figure 2 – Simulation Study

While the demonstration example illustrates how NetREm can be applied to solve a range of problems and applications in various fields, our focus is on applying NetREm in the field of biology (to uncover cell-type-specific interactions among Transcription Factors (TFs) linked to regulation of respective target genes (TGs)). Here, we elaborate on **Fig. 2** and

provide our framework for analyzing NetREm outputs when building our TF-TG regulatory networks (complementary GRNs) and TF-TF coordination networks. We have 5 predictors (TFs, X represents respective expression levels) and 1 response variable (TG with expression y).

A) Heatmap of single-cell gene expression data: 10,000 cell samples prior to standardizing it based on training data. This data incorporates ~40% sparsity (i.e. 0 values) for each of the 6 variables $(TF_1, TF_2, TF_3, TF_4, TF_5, y)$ to resemble current realities of single-cell gene expression data. The 5 predictors in X cluster hierarchically (dendrogram provided above plot), such that TFs 1 and 2 cluster together (then with TF_3) and TFs 4 and 5 cluster together based on their gene expression profiles and expression dynamics. Here, the # of cell samples used to train models is 70% of 10,000, which is 7,000 cells, so M = 7,000. Since $N = 5$, this is a $N \ll M$ scenario.

B) Comparing actual versus expected correlations between predictors and TG after splitting data: 70% for training and 30% for testing. We hold $cor(TF, TG) \approx$ [0.9, 0.5, 0.4, -0.3, -0.8] in the data overall.

C) Pearson correlation matrix among TFs and TG in test data. Dot sizes represent magnitude, colors indicate positive (blue) or negative (red) correlations. These correlations are ~same before and after standardizing X and y. The matrix at the top right corresponds to original correlations in test data, while the matrix at bottom left corresponds to test data correlations after we standardize TFs (expression: X) and TG (expression y) based on insights from training data (e.g. StandardScalar from training X and training y data).

D) Comparing final coefficients c^* predicted by NetREm versus 4 other benchmark models (BRMs: LassoCV, Linear Regression, ElasticNet CV, Ridge CV) and RTNduals (using the underlying RTN method). We note that we would not be able to retrieve the TF-TF links predicted by RTNduals (that uses the RTN method) since RTNduals returns an error that no partial regulon (i.e. TF) has minimum ≥15. Further, we note that RTN does not return any error metrics (e.g. Mean Square Error (MSE)) for the predictions for individual target genes like TG.

E) This lineplot explores effects without network regularization penalty ($\beta = 0$) and with network regularization penalty ($0.01 \le \beta \le 1$). That is, this visualizes model evaluation results (mean square error (MSE)) on train and test data as a function of β. Moreover, it traces impacts of β on the coefficients for 5 TFs, holding all else constant (i.e. $\alpha = 0.1$). As β ↑: train and test MSE values increase, magnitude of coefficients for TF_1 and TF_5 decrease, magnitudes of coefficients for TF_2 and TF_4 increase much greater than for TF_3. NetREm(β = 0.01, LassoCV α) achieves test MSE of ≈0.14.

F) For the main simulation study in **Fig. 2**, we vary β from 0.01 to 2 and analyze changes in TF-TF coordination scores, when we use the Lasso NetREm model type with $\alpha = 0.1$. Cooperative (+) relations are in red, antagonistic (-) relationships are in blue. The larger β is: the more confidence we have in the input PPIN (that is, we want the input PPIN to then carry more weight in terms of deciding outcomes by grouping predictors).

G) Barplot of coefficients and correlation for NetREm(β = 10, α = 0.1). We expand the main example in **Fig. 2A** to add 20 more TFs with r = 0.55 with TG, and 20 more TFs with r = -0.35 with TG. Correlations (right plot) are for the training data. We use the **Fig. 2A** network of strong known $TF_4 - TF_5$ links and $TF_1 - TF_2$ links (known nodes with PPIs are TF_1, TF_2, TF_4, TF_5) and the rest are artificial nodes and edges (weight = 0.01). So, we have $N = 45$ candidate TFs in this case. We make $\beta = 10$ (and retain $\alpha = 0.1$) and run NetREm for the 7,000 training cells. We illustrate the grouping variable selection property of NetREm, and how TF_1, TF_2, and TF_5 tend to have meaningful coefficients $|c^*| \gg 0$. This is important, since we have added 20 TFs that have a stronger correlation (r = 0.55) with TG y expression levels than TF_2 does and those TFs all still result in lower $|c^*|$ than TF_2 does. Further, TF_4 (has the smallest expression $|r| \approx 0.29$ with y) is 1 of the $N^* = 25$ TFs (of the 45 TFs) that have a non-zero coefficient in c^*. If we use Lasso regression with $\alpha = 0.1$ and no y-intercept (not shown for visual simplicity), we would get $N^* = 23$ TFs, where we lose TF_4 (as well as TF_3); that is, TF_4 is eliminated sheerly due to its low correlation with TG expression y. This shows how NetREm can prioritize TF_4 based on its strong known PPI connection with TF_5. That is, a TF (like TF4) that is lowly correlated with gene expression of its true TG, may need help so that it is properly detected; integrating a PPI network can help include these TFs that may be missing or not prioritized.

A)

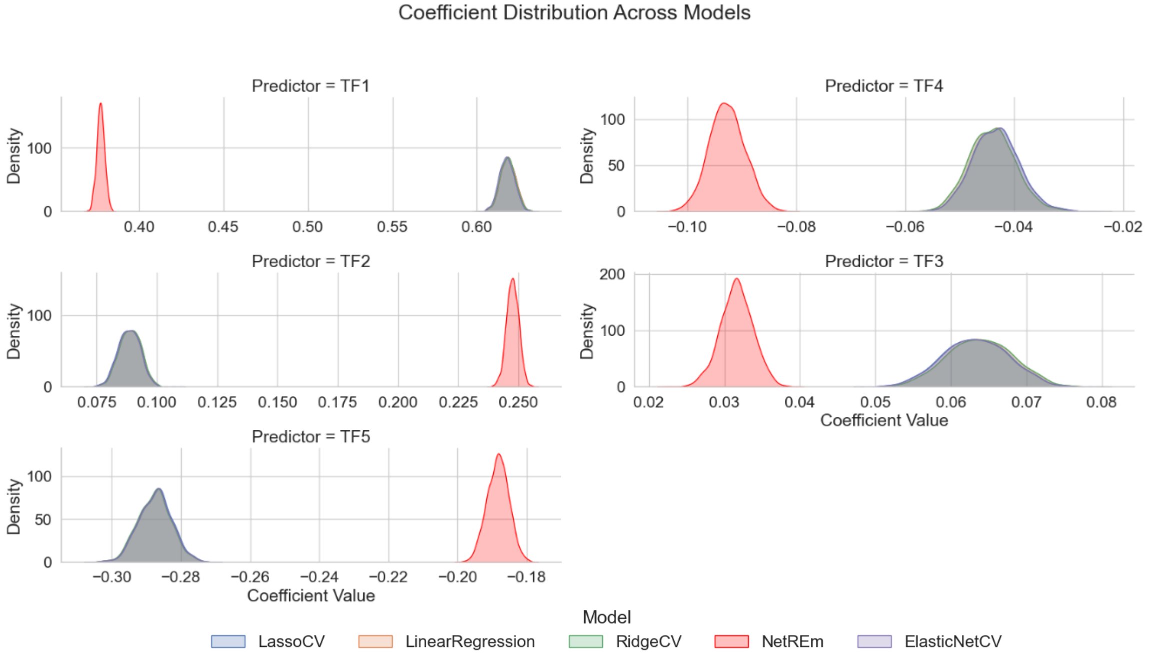

Coefficient Distribution Across Models
Predictor = TF1
Predictor = TF4
Predictor = TF2
Predictor = TF3
Predictor = TF5
Density
Coefficient Value
Model
LassoCV
LinearRegression
RidgeCV
NetREm
ElasticNetCV

B)

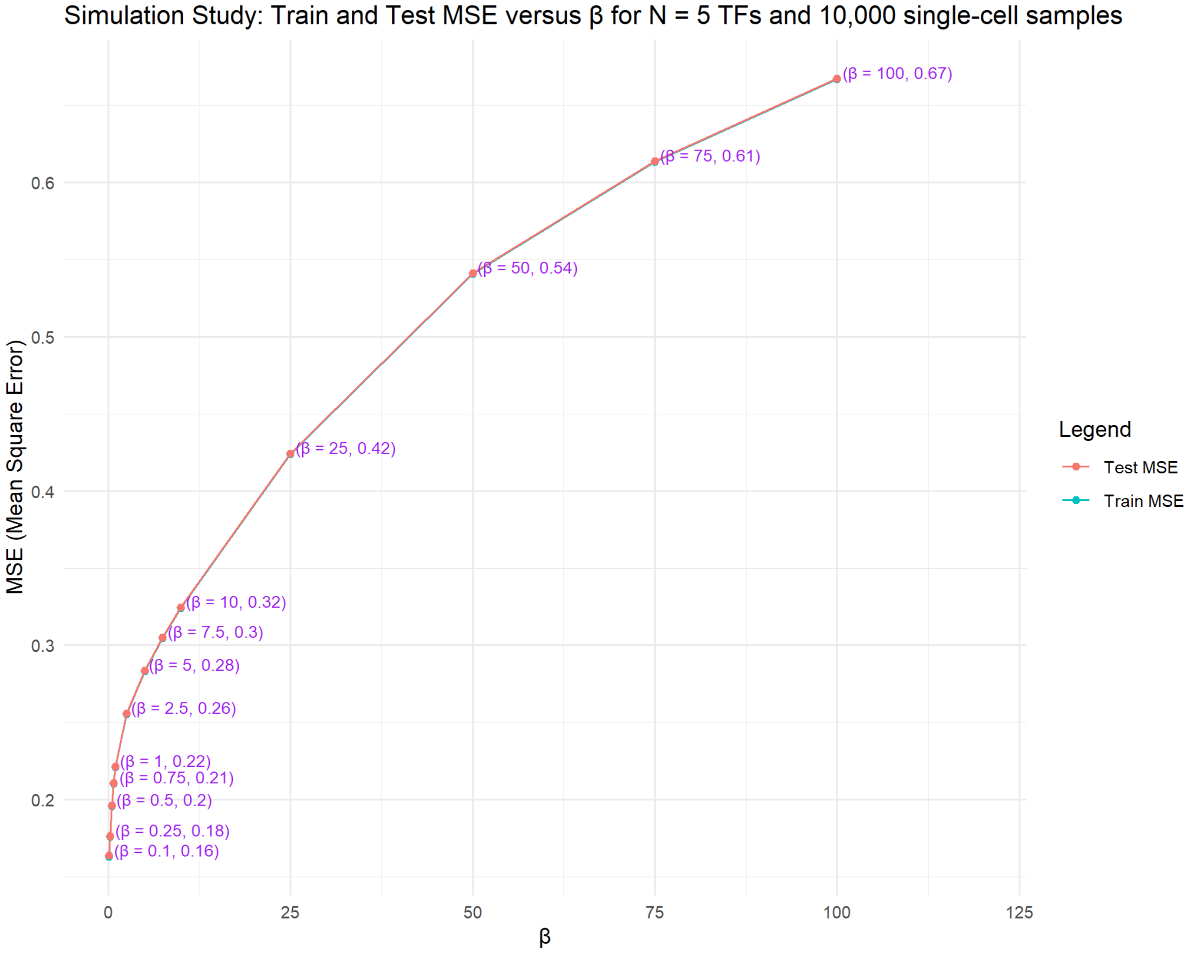

Simulation Study: Train and Test MSE versus β for N = 5 TFs and 10,000 single-cell samples
(β = 100, 0.67)
(β = 75, 0.61)
(β = 50, 0.54)
(β = 25, 0.42)
(β = 10, 0.32)
(β = 7.5, 0.3)
(β = 5, 0.28)
(β = 2.5, 0.26)
(β = 1, 0.22)
(β = 0.75, 0.21)
(β = 0.5, 0.2)
(β = 0.25, 0.18)
(β = 0.1, 0.16)
MSE (Mean Square Error)
β
Legend
Test MSE
Train MSE

C)

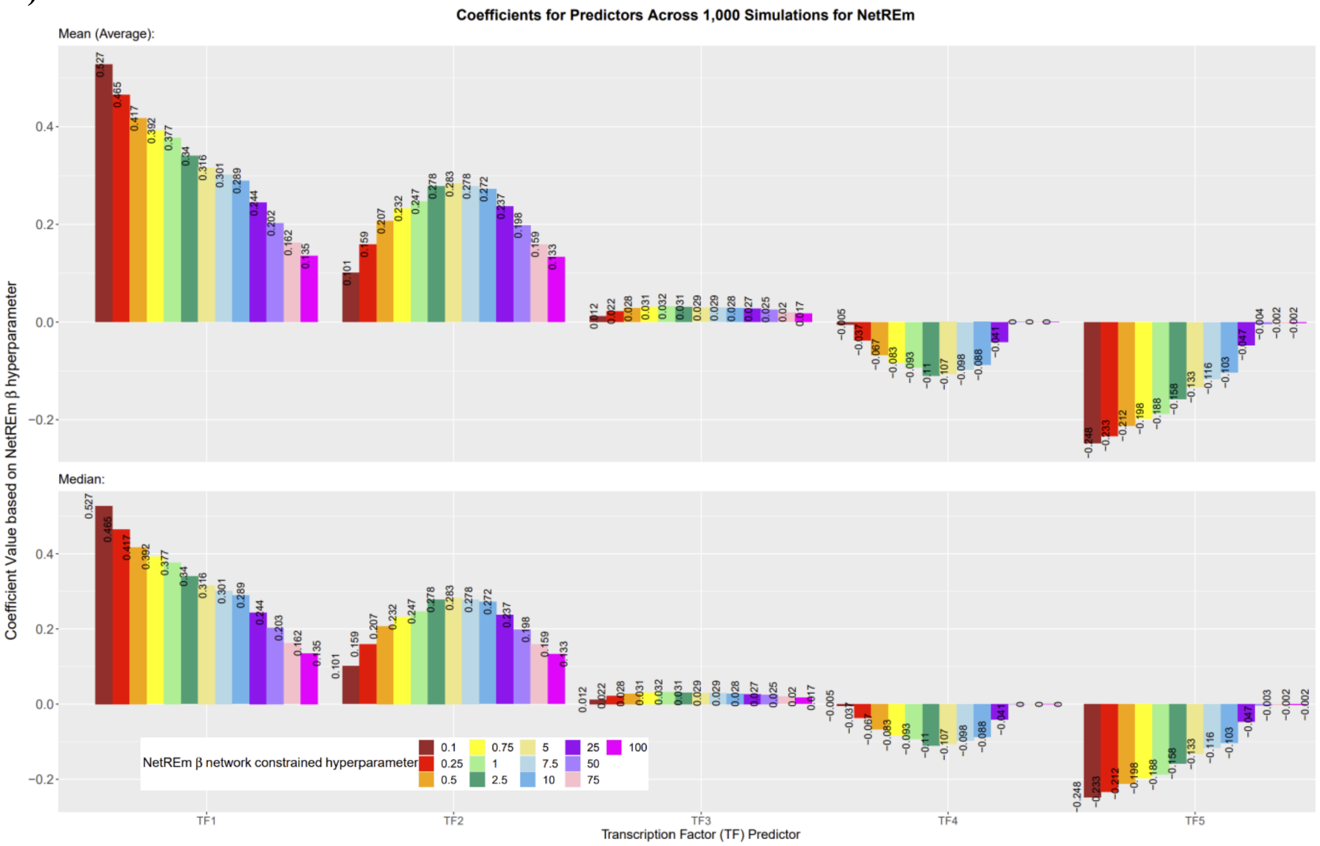

Supplementary Figure 3 – Analysis of NetREm's performance based on 1,000 simulations

A) Density distribution of coefficients for the 5 TFs across NetREm and the 4 benchmark models (LassoCV, LinearRegression, RidgeCV, ElasticNetCV) for 1,000 simulations with the same $cor(TF, TG)$ and prior biological network. In particular, coefficients for TF_1, TF_2, TF_5 typically display exceptionally low standard deviation (std), underscoring NetREm's stability and consistent inference of minimal influence in the model. The coefficient density distribution plot highlights narrower and more peaked distributions of NetREm's coefficients, indicating reduced variability, reduced spread, and enhanced stability across simulations. Overall, NetREm enhances robustness via network constraint regularization, proving particularly advantageous in high-dimensional data prone to multicollinearity and overfitting. NetREm's density distributions of c^* in **Fig. S3A** are more peaked. NetREm's ability to incorporate comprehensive prior data, such as PPINs, into GRN predictions from expression data is a significant advantage of NetREm over these traditional BRMs, setting it apart from the pack.

B) Line plot of average training and testing Mean Square Error (MSE) values across the 1,000 simulations, for 13 different NetREm models on the same input data (benchmarking β); each model is based on a varied network constrained hyperparameter β. Here, as β increases, the MSE values tend to increase monotonically.

C) Comparative barplots of the average and median coefficients for the 5 TF predictors across the 1,000 simulations for the 13 NetREm models (varying network constrained hyperparameter β). That is, this is additional analysis based on the benchmark β results in **Fig. S3B**. We note that for $\beta > 5$, the network constraint is too harsh. This excessive network regularization penalty can be detrimental, leading to models overly constrained by the predefined network structure as the magnitude of coefficients tends to decrease. In particular, the magnitude of coefficients for TF_2 and TF_4 is a U-shape, which increases for $\beta \in \{0.1, 0.25, 0.5, 0.75, 1, 2.5, 5, 7.5, 10, 25, 50, 75, 100\}$ and starts decreasing for $\beta \in \{7.5, 10, 25, 50, 75, 100\}$. By seamlessly blending predictive precision with structural intelligibility, NetREm stands out for its superior consistency, potential to unveil complex data structures, and computational efficacy.

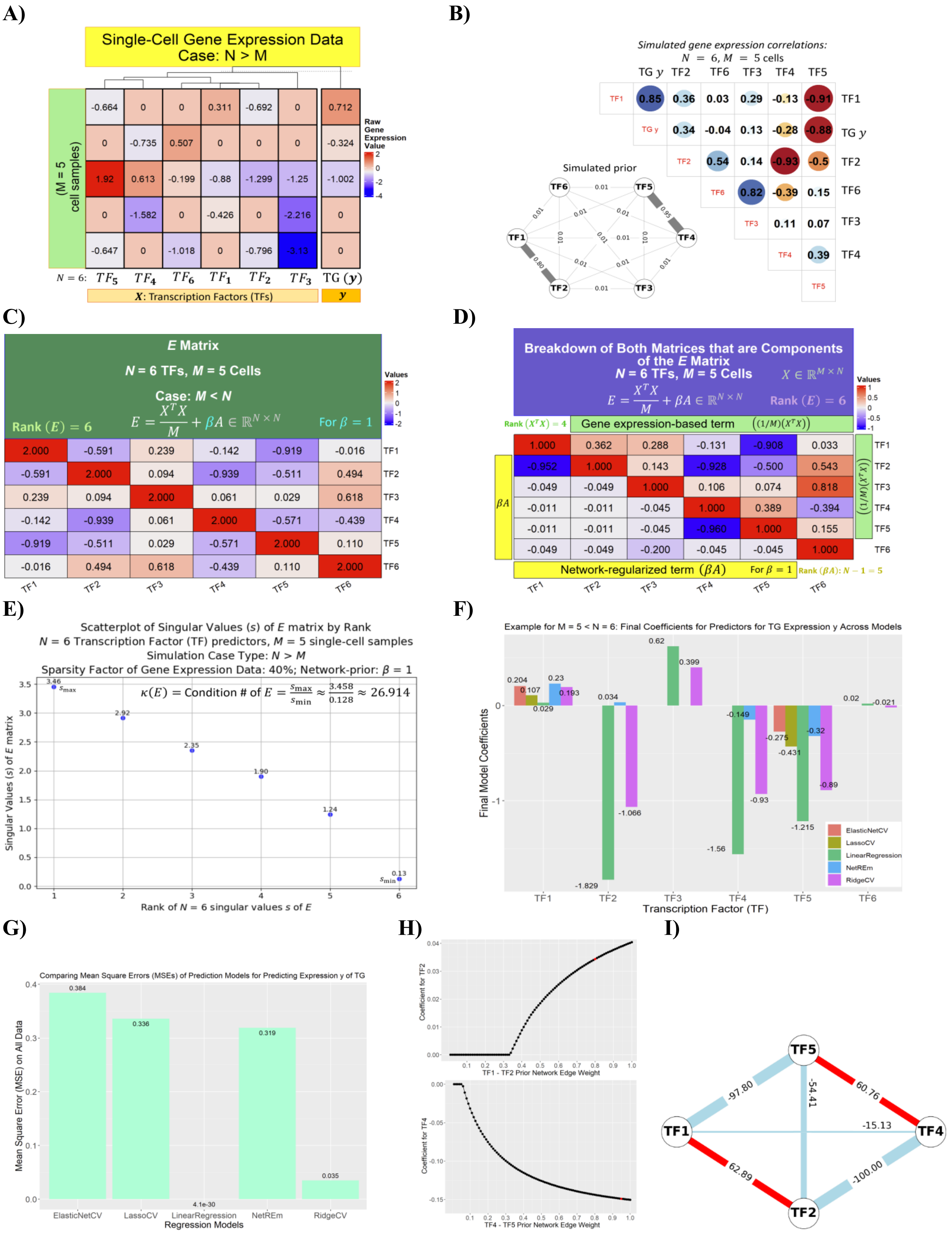
A)
Single-Cell Gene Expression Data
Case: N > M
(M = 5 cell samples)
Raw Gene Expression Value
N = 6:
X: Transcription Factors (TFs)
y
B)
Simulated gene expression correlations:
N = 6, M = 5 cells
Simulated prior
C)
E Matrix
N = 6 TFs, M = 5 Cells
Case: M < N
Rank (E) = 6
For β = 1
Values
D)
Breakdown of Both Matrices that are Components of the E Matrix
N = 6 TFs, M = 5 Cells
Rank (E) = 6
Gene expression-based term
Network-regularized term (βA)
For β = 1
Values
E)
Scatterplot of Singular Values (s) of E matrix by Rank
N = 6 Transcription Factor (TF) predictors, M = 5 single-cell samples
Simulation Case Type: N > M
Sparsity Factor of Gene Expression Data: 40%; Network-prior: β = 1
Singular Values (s) of E matrix
Rank of N = 6 singular values s of E
F)
Example for M = 5 < N = 6: Final Coefficients for Predictors for TG Expression y Across Models
Final Model Coefficients
Transcription Factor (TF)
ElasticNetCV
LassoCV
LinearRegression
NetREm
RidgeCV
G)
Comparing Mean Square Errors (MSEs) of Prediction Models for Predicting Expression y of TG
Mean Square Error (MSE) on All Data
Regression Models
ElasticNetCV
LassoCV
LinearRegression
NetREm
RidgeCV
H)
Coefficient for TF2
TF1 - TF2 Prior Network Edge Weight
Coefficient for TF4
TF4 - TF5 Prior Network Edge Weight
I)
TF5
TF1
TF4
TF2
-97.80
60.76
-54.41
-15.13
62.89
-100.00

Supplementary Figure 4 – Under-the-hood: Simulation Study adapted for a case where predictors $N > M$ cells (samples).

Please note that this figure details the mechanics and mathematics of NetREm, under-the-hood. We have 6 TF predictors $[TF_1, TF_2, TF_3, TF_4, TF_5, TF_6]$ for TG and $M = 5$ single cell samples and use all data for training. These 6 TFs have respective expression levels $X \in \mathbb{R}^{5 \times 6}$. We design this adaptation envisioning TF_6 should definitely not be considered a true TF regulator of TG (i.e. candidate TF_6 should not be selected as a final TF for TG). TG has expression levels $y = [y_1 \quad y_2 \quad y_3 \quad y_4 \quad y_5]^T$.

A) We ensure 40% sparsity for each variable, so 2 cells out of 5 for each TF are 0. These are expression values prior to standardization. Ultimately, we standardize this expression data so that each variable has a mean of 0 and standard deviation of 1.

B) Bottom left: prior biological network with default edge weights (0.1), with stronger experimentally-verified connections for TF_1- TF_2 (0.8) and TF_4- TF_5 (0.95). Orphaned TF_3 and TF_6 nodes are added in with artificial pairwise edges. The top right presents a correlation r matrix among TFs and TG in training expression data where: $cor(TF, TG) \approx$ [0.85, 0.36, 0.29, -0.13, -0.91, 0.03] for TF_1 to TF_6. Dot sizes represent magnitude, colors indicate $r > 0$ (blue) or $r < 0$ (red).

C) To solve the network-regularized regression problem, NetREm creates a symmetric, positive semi-definite matrix $E = \frac{X^TX}{M} + \beta A \in \mathbb{R}^{N \times N}$ by summing expression- ($\frac{1}{M}X^TX$) and PPIN- (βA) -based matrices. Since we select $\beta = 1$, we have $E = \frac{X^TX}{M} + A$. We display values of E, noting E is of full rank since it is 6 and rank(E) can be a max of $\min(\text{rows} = 6,\ \text{columns} = 6) = 6$.

D) Individual components of E. The 1st term (Gene expression-based term $\frac{X^TX}{M}$), is on top right (above main diagonal of 1). This matrix always has main diagonal of 1 since expression data is standardized. Gram matrix X^TX has a rank of 4 in this case, and thus is not full rank since max rank that $X \in \mathbb{R}^{5 \times 6}$ can be $\min(M = 5,\ N = 6) = 5$. The 2nd term (network-regularized term βA) is on bottom left (below main diagonal). When $\beta = 1$, this 2nd term also has a main diagonal of 1. Since A is a variant of the Graph Laplacian matrix, it has rank $N - 1$, which is 6 – 1 = 5. Hence, main diagonal of E is 2. Ultimately, both terms help E achieve a full rank, which we show here. That is, rank$(X^TX) = 4$ is deficient as it is not 5 and rank$(\beta A) = N - 1 = 5$.

E) NetREm performs Singular Value Decomposition (SVD) on E to uncover 6 positive singular values: $\mathcal{S} = \{s_1, s_2, s_3, s_4, s_5, s_6\}$). We find $s_{min} = 0.1284$, $s_{max} = 3.4567$, $\frac{s_{min}}{s_{max}} \approx 0.03716$, and condition # of $E = \kappa(E) = \frac{s_{max}}{s_{min}} = 26.9136 \ll 1e6$, so E is well-conditioned. NetREm may help improve rank deficiency, especially for $N > M$. E has full rank of 6 = N. Results are stable.

Fig. S4F-I focus on outputs of NetREm run with hyperparameters: network regularization $\beta = 1$, sparsity $\alpha = 0.1$.

F) Final coefficients c^* for each TF predictor for regulating TG in TF-TG regulatory network. NetREm coefficients compared to those from the other 4 benchmark models. NetREm and BRMs learn correct c^* signs for TF_1 and TF_5. Only NetREm accurately learns TF_2's $c^* > 0$.

G) Mean square error (MSE) values for 5 models. While ElasticNetCV and LassoCV also remove poorly connected TF_3 and TF_5, they also eliminate TF_4 and have higher MSE. **H)** Top: Effects of varying TF_1-TF_2 edge weight w in original biological prior network, from 0.01 to 1 in 0.01 increments, holding all other parameters fixed (e.g. Lasso model for NetREm: $\alpha = 0.1$). Respective TF_2 c^* increases monotonically in arc shape. Red dot: selected w used for simulated prior PPIN. Bottom: Similar sensitivity analysis for TF_4 and TF_5 shows TF_4 $|c^*|$ increases, becoming more negative from 0, as the TF_4-TF_5 w is perturbed. Results are consistent with **Fig. 2D-E** for the main simulation.

I) TF-TF coordination network for the TG: $-100 \leq B \leq 100$. Red: cooperative ($B > 0$) interactions. Blue: antagonistic ($B < 0$) interactions between the TFs. Activators TF_1 and TF_2 cooperate and repressors TF_4 and TF_5 cooperate, and other interactions among the 4 final TFs are antagonistic.

A) SERGIO-simulated Data: 1,000 Cells: M = 700 cells (training data)

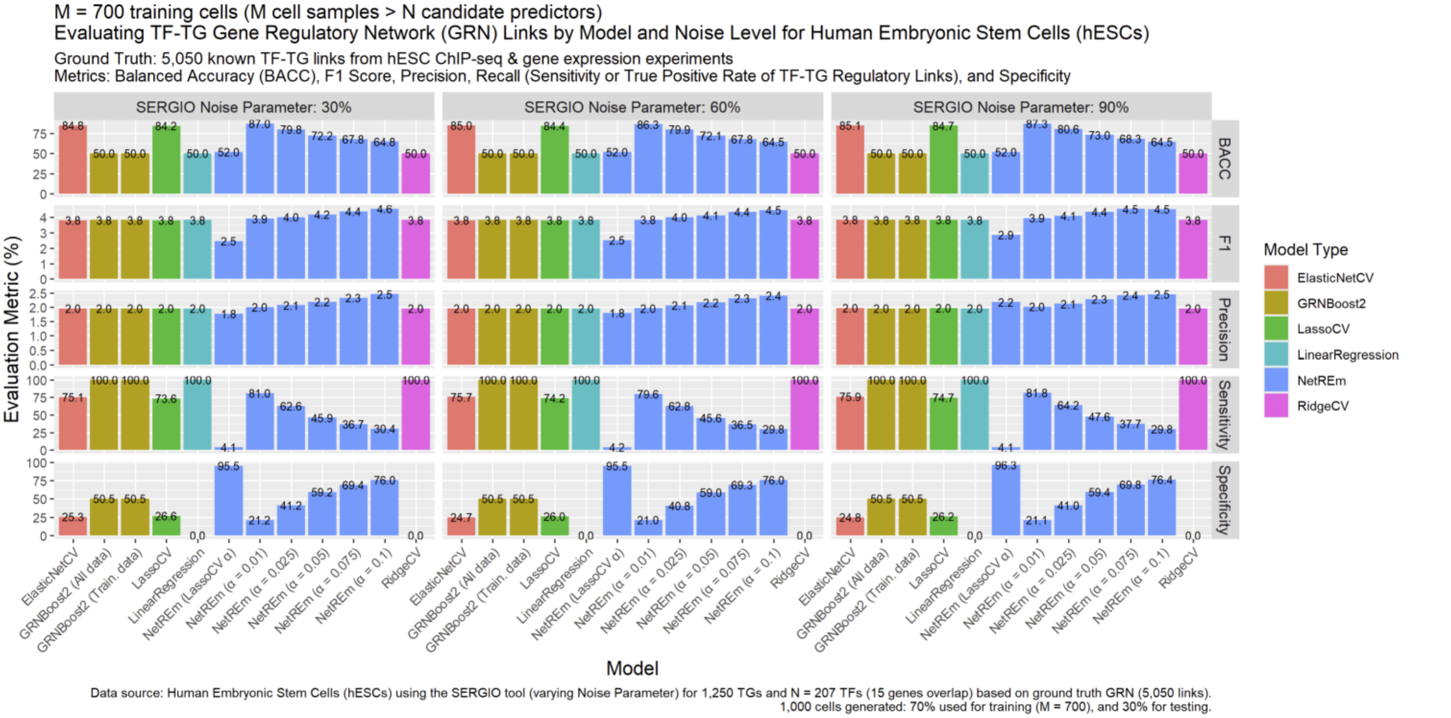

B) SERGIO-simulated Data: 100 Cells: M = 70 cells (train), N = 207 TF predictors (206 when TG is a TF)

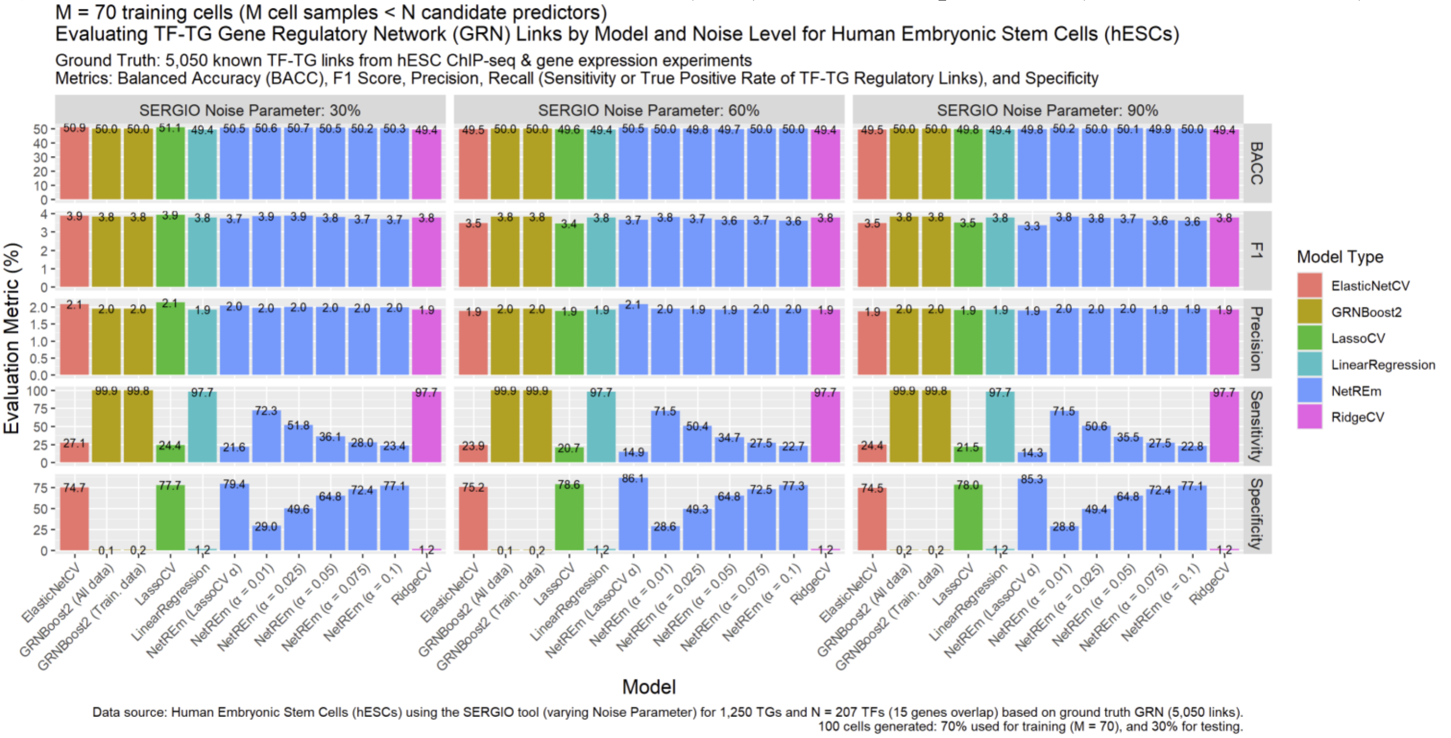

C)

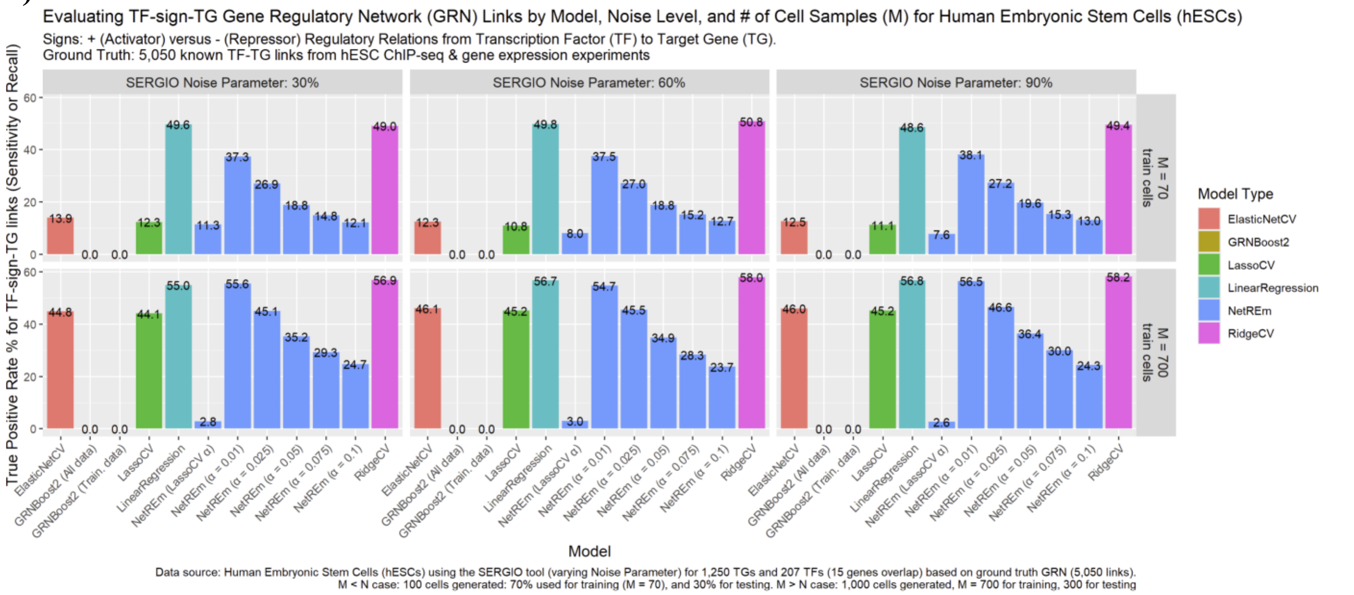

Evaluating TF-sign-TG Gene Regulatory Network (GRN) Links by Model, Noise Level, and # of Cell Samples (M) for Human Embryonic Stem Cells (hESCs)
Signs: + (Activator) versus - (Repressor) Regulatory Relations from Transcription Factor (TF) to Target Gene (TG).
Ground Truth: 5,050 known TF-TG links from hESC ChIP-seq & gene expression experiments
SERGIO Noise Parameter: 30%
SERGIO Noise Parameter: 60%
SERGIO Noise Parameter: 90%
M = 70 train cells
M = 700 train cells
True Positive Rate % for TF-sign-TG links (Sensitivity or Recall)
Model Type
ElasticNetCV
GRNBoost2
LassoCV
LinearRegression
NetREm
RidgeCV
Model
Data source: Human Embryonic Stem Cells (hESCs) using the SERGIO tool (varying Noise Parameter) for 1,250 TGs and 207 TFs (15 genes overlap) based on ground truth GRN (5,050 links).
M < N case: 100 cells generated: 70% used for training (M = 70), and 30% for testing. M > N case: 1,000 cells generated, M = 700 for training, 300 for testing

D)

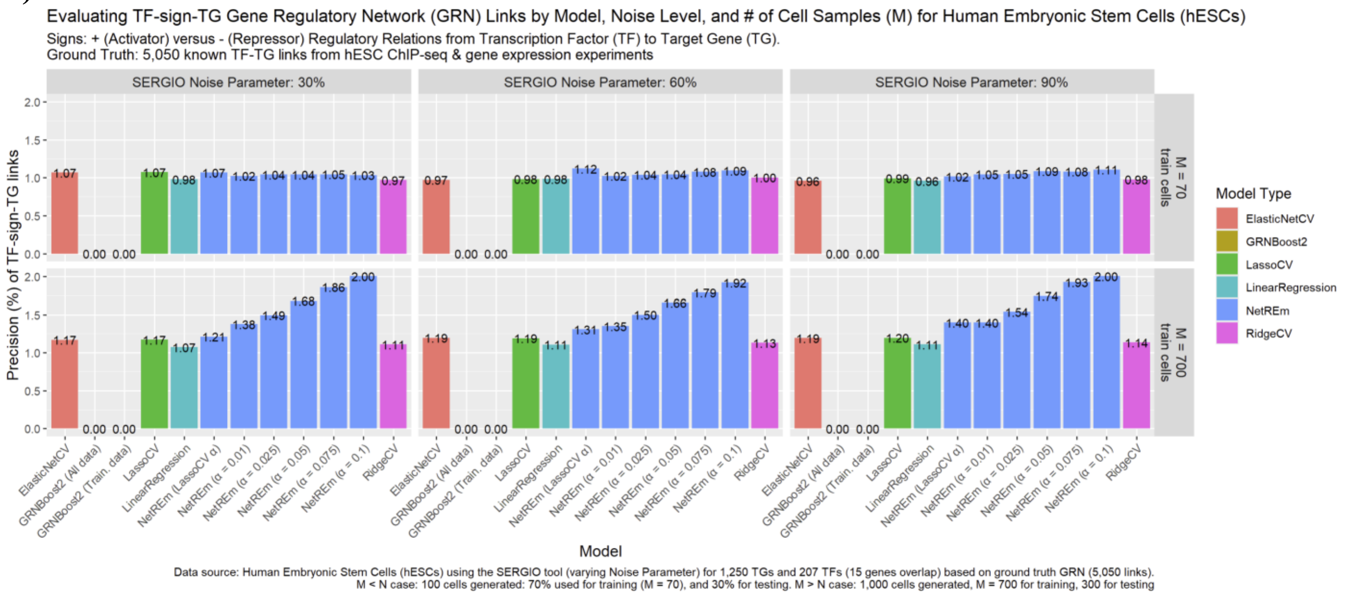

Evaluating TF-sign-TG Gene Regulatory Network (GRN) Links by Model, Noise Level, and # of Cell Samples (M) for Human Embryonic Stem Cells (hESCs)
Signs: + (Activator) versus - (Repressor) Regulatory Relations from Transcription Factor (TF) to Target Gene (TG).
Ground Truth: 5,050 known TF-TG links from hESC ChIP-seq & gene expression experiments
SERGIO Noise Parameter: 30%
SERGIO Noise Parameter: 60%
SERGIO Noise Parameter: 90%
M = 70 train cells
M = 700 train cells
Precision (%) of TF-sign-TG links
Model Type
ElasticNetCV
GRNBoost2
LassoCV
LinearRegression
NetREm
RidgeCV
Model
Data source: Human Embryonic Stem Cells (hESCs) using the SERGIO tool (varying Noise Parameter) for 1,250 TGs and 207 TFs (15 genes overlap) based on ground truth GRN (5,050 links).
M < N case: 100 cells generated: 70% used for training (M = 70), and 30% for testing. M > N case: 1,000 cells generated, M = 700 for training, 300 for testing

E)

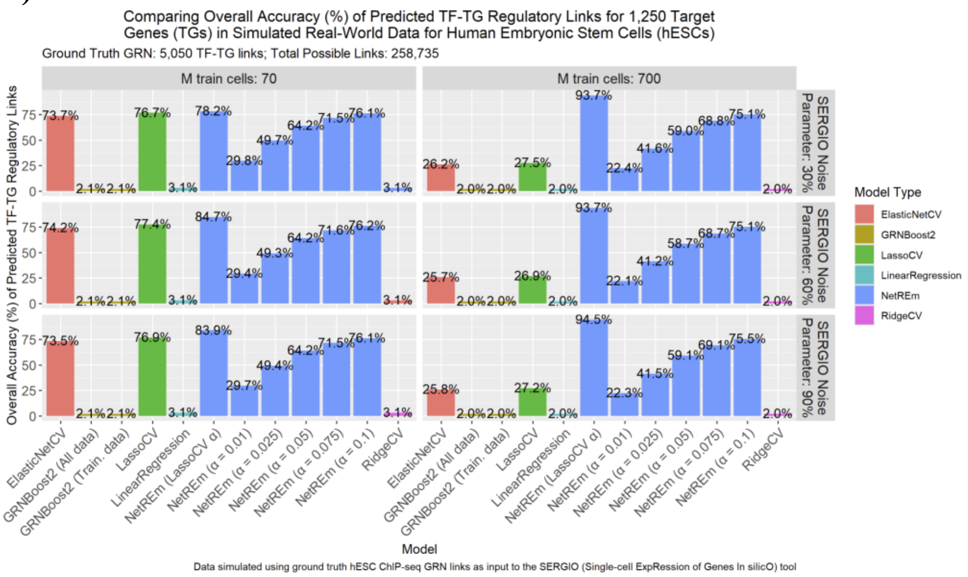

Comparing Overall Accuracy (%) of Predicted TF-TG Regulatory Links for 1,250 Target Genes (TGs) in Simulated Real-World Data for Human Embryonic Stem Cells (hESCs)
Ground Truth GRN: 5,050 TF-TG links; Total Possible Links: 258,735
M train cells: 70
M train cells: 700
SERGIO Noise Parameter: 30%
SERGIO Noise Parameter: 60%
SERGIO Noise Parameter: 90%
Overall Accuracy (%) of Predicted TF-TG Regulatory Links
Model Type
ElasticNetCV
GRNBoost2
LassoCV
LinearRegression
NetREm
RidgeCV
Model
Data simulated using ground truth hESC ChIP-seq GRN links as input to the SERGIO (Single-cell ExpRession of Genes In silicO) tool

Supplementary Figure 5 – Evaluating TF-TG Regulatory Networks in simulated Human Embryonic Stem Cells (hESCs)

We benchmark NetREm's performance for constructing TF-TG regulatory links for 1,250 TGs in hESCs across each of the 6 SERGIO-simulated datasets, which vary based on noise parameter (30%, 60%, 90%) and training cells M (70 versus 700) where 70% of data is used for training. SERGIO incorporates TF-TG regulatory interactions (e.g. stochastic nature of transcription, regulation of TFs by many TFs) found in true data. Data simulated by SERGIO is comparable to experimental data generated by Illumina HiSeq2000, Drop-seq, Illumina 10X chromium, Smart-seq. We input the comprehensive human PPIN of 21,321 edges comprising 10,777 known links and 10,544 artificial links (η = 0.01) for these 207 TFs. For each of the 15 TFs that are also TGs, we remove respective TF and its associated edges from input PPIN and note $N = 206$ for them; $N = \mathcal{N}$= 207 for remaining 1,235 TGs. We run 6 NetREm models with $\beta = 1$, varying α (0.01, 0.025, 0.05, 0.075, 0.1, LassoCV). As α increases, # of discovered TF-TG links decreases as expected, given stricter α leads to sparser results (i.e. fewer $c^* \neq 0$ across TGs).

For the TF-sign-TG regulatory link comparison in hESCs, we cannot consider GRNBoost2 as that only returns relative importance scores for TF-TG scores and does not provide any signed coefficient values; instead, we will consider NetREm and the 4 benchmark models (ElasticNetCV, LassoCV, Linear Regression, and RidgeCV). For HSCs and mESCs, we have TF-TG regulatory links but do not have more information regarding the potential role of the TF in terms of the TG expression. Thus, our TP predictions are simply predicted TF-TG regulatory links that are found in the gold standard GRN networks. We also analyze TPs in hESCs in terms of TF-TG regulatory links, ignoring the coefficient c^* signs. When assessing the TF-TG regulatory links, we compare NetREm with the 5 benchmark models (including GRNBoost2).

A) Focuses on 5 metrics (Balanced Accuracy (BACC), F1 scores, Precision, Sensitivity, Specificity) of SERGIO-simulated data where $N < M = 700$ cells in train data. As α increases, specificity, precision, and F1 scores improve; balanced accuracy (BACC) and sensitivity decline, particularly for $M = 700$. For 90% noise, NetREm models maintain or exceed a precision of 2 to 2.5% compared to benchmarks ≈2%.
B) 5 metrics for data where $N > M = 70$ cells in train data. When $M = 70$, increased α results in higher specificity but lower sensitivity and general decreases in F1
C) True positive (TP) of TF-sign-TG links where sign ($c^* > 0$: activator, $c^* < 0$: repressor) matters. Comparing results across noise for 2 values of M. We compare NetREm with BRMs in terms of signed TF-TG links, focusing on predicting c^* signs (+: activate, -: repress).
D) Focuses on Precision of TF-sign-TG regulatory links.
E) Comparing accuracy (TPs + True Negatives (TNs))/(Total predictions).

A)

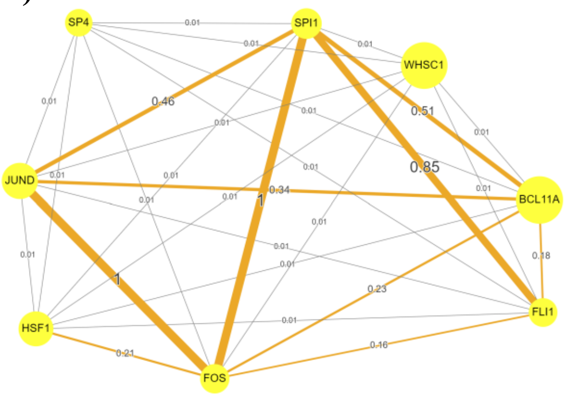

B)

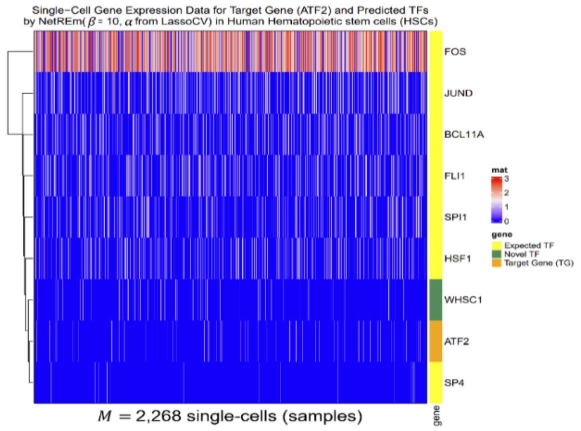

C)

Pairwise correlations (r) Among TFs and the Target Gene (TG) ATF2 in Human Hematopoietic Stem Cells

	BCL11A	ATF2	WHSC1	SP4	HSF1	SPI1	FLI1	FOS	JUND
BCL11A		0.004	-0.047	-0.006	0.009	0.009	-0.027	-0.027	-0.047
ATF2	0.004		0.002	0.009	-0.032	0.020	0.025	0.004	0.016
WHSC1	-0.047	0.002		-0.017	0.016	-0.008	0.013	0.003	-0.004
SP4	-0.006	0.009	-0.017		0.050	0.047	-0.020	0.016	-0.019
HSF1	0.009	-0.032	0.016	0.050		0.023	-0.005	0.011	-0.042
SPI1	0.009	0.020	-0.008	0.047	0.023		0.022	-0.003	-0.008
FLI1	-0.027	0.025	0.013	-0.020	-0.005	0.022		0.031	-0.008
FOS	-0.027	0.004	0.003	0.016	0.011	-0.003	0.031		0.045
JUND	-0.047	0.016	-0.004	-0.019	-0.042	-0.008	-0.008	0.045	

D)

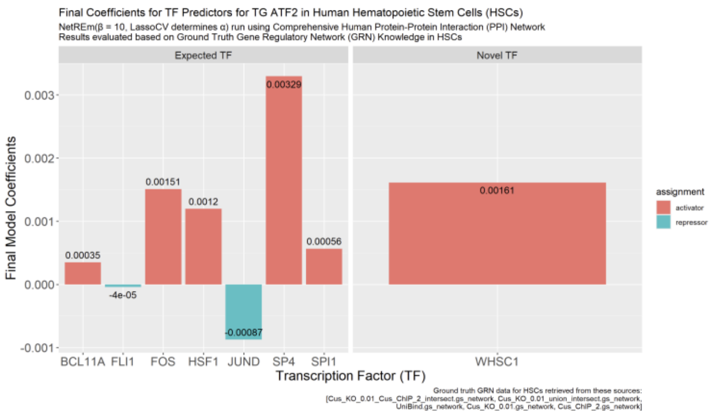

E)

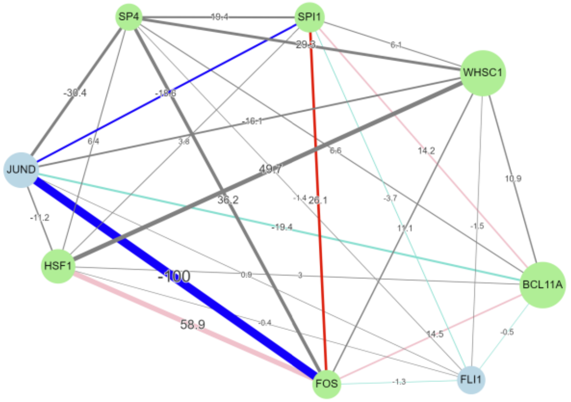

F)

GO	Description	Log10(P)
WP2849	Hematopoietic stem cell differentiation	-7.6
hsa04380	Osteoclast differentiation	-6.5
GO:0030099	myeloid cell differentiation	-5.5

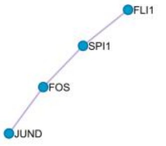

G)

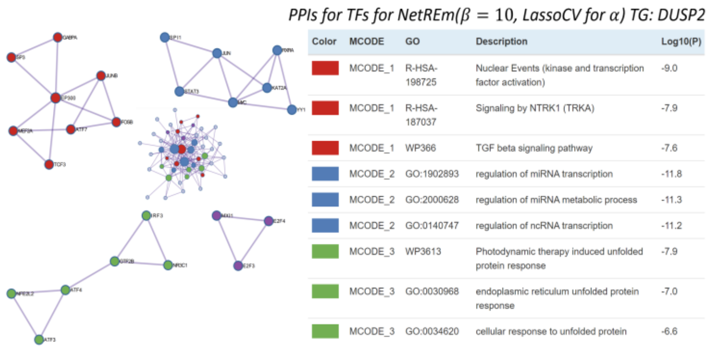

H)

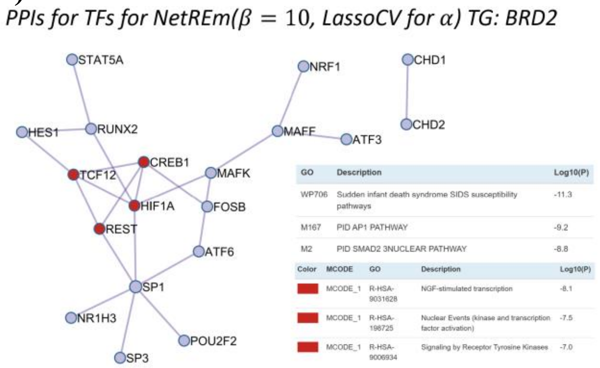

I)

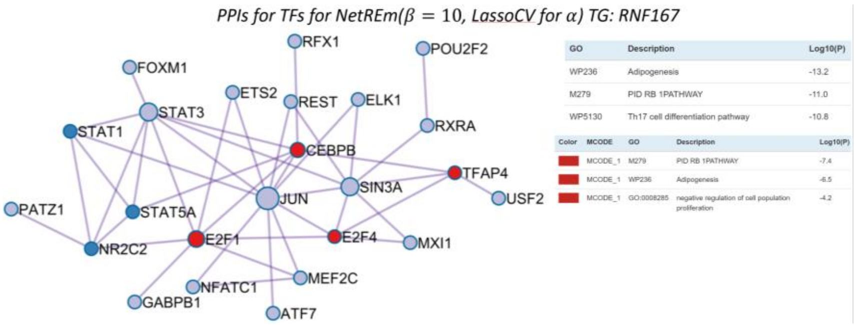

Supplementary Figure 6 – Highlighting NetREm's Grouped TF Selection Property (prioritizing TF-TF relations in PPIN) using 4 Target Genes (TGs) in human Hematopoietic Stem Cells (HSCs)

We highlight NetREm's potential to flag groups of coordinating TFs connected along biologically meaningful, cell-type-specific PPIs(Li and Li 2008). Novel TFs are those that are not in the ground truth GRN for that TG in HSCs. Please note: **Fig. S6A-F** focus on NetREm's results for *ATF2*: NetREm($\beta = 10$, LassoCV to identify optimal α) identifies $N^* = 8$ final TFs for *ATF2* regulation in HSCs, and 7 of 8 (excluding WHSC1) are substantiated in ground truth valid GRNs (Zhang et al. 2023) for *ATF2* in HSCs.

A) Among these 8 TFs there are strong known PPIs between FOS-SPI1 and FOS-JUND (w = 1) and beyond. Here, TFs are yellow nodes and orange links (with w) are comprehensive input PPI network (PPIN) weights between TFs. Grey links are artificial (default edge w: $\eta = 0.01$). SP4 and WHSC1 lack documented PPIs in this network. NetREm performs grouped variable selection of TFs based on a fully-connected input PPIN of known PPIs and artificially-added weak links.

B) Gene expression levels for SP4 and WHSC1 hierarchically cluster closest to those of *ATF2* in HSCs (based on dendrogram on left), which may explain their inclusion as final TFs.

C) Pearson Correlations (r) among 8 TFs and TG *ATF2* in HSCs across 2,268 cells.

D) NetREm's TF-TG regulatory network for *ATF2* where coefficients $c^* > 0$ for activator TFs and $c^* < 0$ for repressor TFs. Literature supports FLI1 (core TF for hematopoiesis) and JUND may act as repressors (Chen et al. 2008; Liu et al. 2019) and others as activators.

E) NetREm outputs *ATF2*-specific TF-TF coordination network for the 8 TFs. Red = + relationship (cooperative) and found in input PPIN and PPI is associated with immune-related conditions in contextual Integrated Interactions Database (IID) (Kotlyar et. al 2022). Pink = + relationship (cooperative) and known PPI. Blue = - relationship (antagonistic) and known PPI (in input PPIn) and associated with immune-related conditions in contextual IID. Turquoise = - relationship (antagonistic) and found in input PPIN. Grey = Novel relationship (not found in the comprehensive input PPIN). Contextual IID corroborates antagonistic JUND-FOS and JUND-SPI and cooperative SPI1-FOS links are immune-related (e.g. bone marrow, lymph nodes, synovial macrophages, leukemia, multiple myeloma, lymphoma, immune system cancer, bone inflammation disease, myeloid leukemia, bone marrow cancer, myeloid neoplasm). NetREm uncovers potential antagonism between SPI1 and FLI1; SPI1 has an inhibitory effect on FLI1-induced erythroid progenitor dedifferentiation (Vecchiarelli-Federico et al. 2017).

F) Metascape (Zhou et al. 2019) shows sequential JUND-FOS-SPI1- FLI1 PPIs are strongly enriched for roles in HSC differentiation among others.

Fig. S6G-I focus on enriched PPIs among final TFs for other target genes (TGs): *DUSP2, BRD2, RNF167*. These are examples of NetREm uncovering meaningful subnetworks of PPIs among predictors for 3 other TGs (*BRD2*: 13 of 21 TFs are validated, *RNF167*: 17 of 27 TFs are validated, *DUSP2*: 25 of 47 TFs are validated) that are missing in ElasticNet and Lasso models and predicted to have all candidate TFs by Ridge and Linear regression models.

G) Metascape-enriched PPIs among the 47 predicted TFs for *DUSP2*. Novel TFs: ATF4, ATF5, CLOCK, E2F3, ERG, FOSB, GTF2B, IRF3, JUN, KAT2A, KLF13, NFE2, NFE2L2, NFE2L3, NFYB, NR2F6, NR3C1, REL, RELB, SKIL, STAT2, STAT3. In *DUSP2*, FOSB is involved in the TGF beta signaling pathway with 7 substantiated TFs, ATF4 and NFE2L2 involved in cellular response to unfolded protein with 5 substantiated TFs, and JUN, KAT2A, STAT3 are involved in regulating miRNA and ncRNA transcription and metabolic processes with other substantiated TFs.

H) Metascape-enriched PPIs among 21 predicted TFs for *BRD2*. Novel TFs: ATF6, E4F1, FOSB, HES1, HIF1A, NR1H3, RUNX2, SPIB. HIF1A is involved in enriched PPIs with 3 substantiated TFs.

I) Novel TFs for *RNF167*: ARID5B, ATF7, MEF2C, NFE2L3, NR2C2, PATZ1, RFX1, STAT1, STAT3, TFAP4. Novel TF TFAP4, is involved in Adipogenesis (creating adipocyte stem cells) and negative regulation of cell population prolifer

ation with 3 substantiated TFs: E2F1, CEBPB, E2F4. Overall, NetREm can identify biologically-enriched PPI-subnetworks for TGs. Thus, NetREm has superior potential in revealing intricate regulatory networks for TGs.

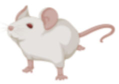

A)

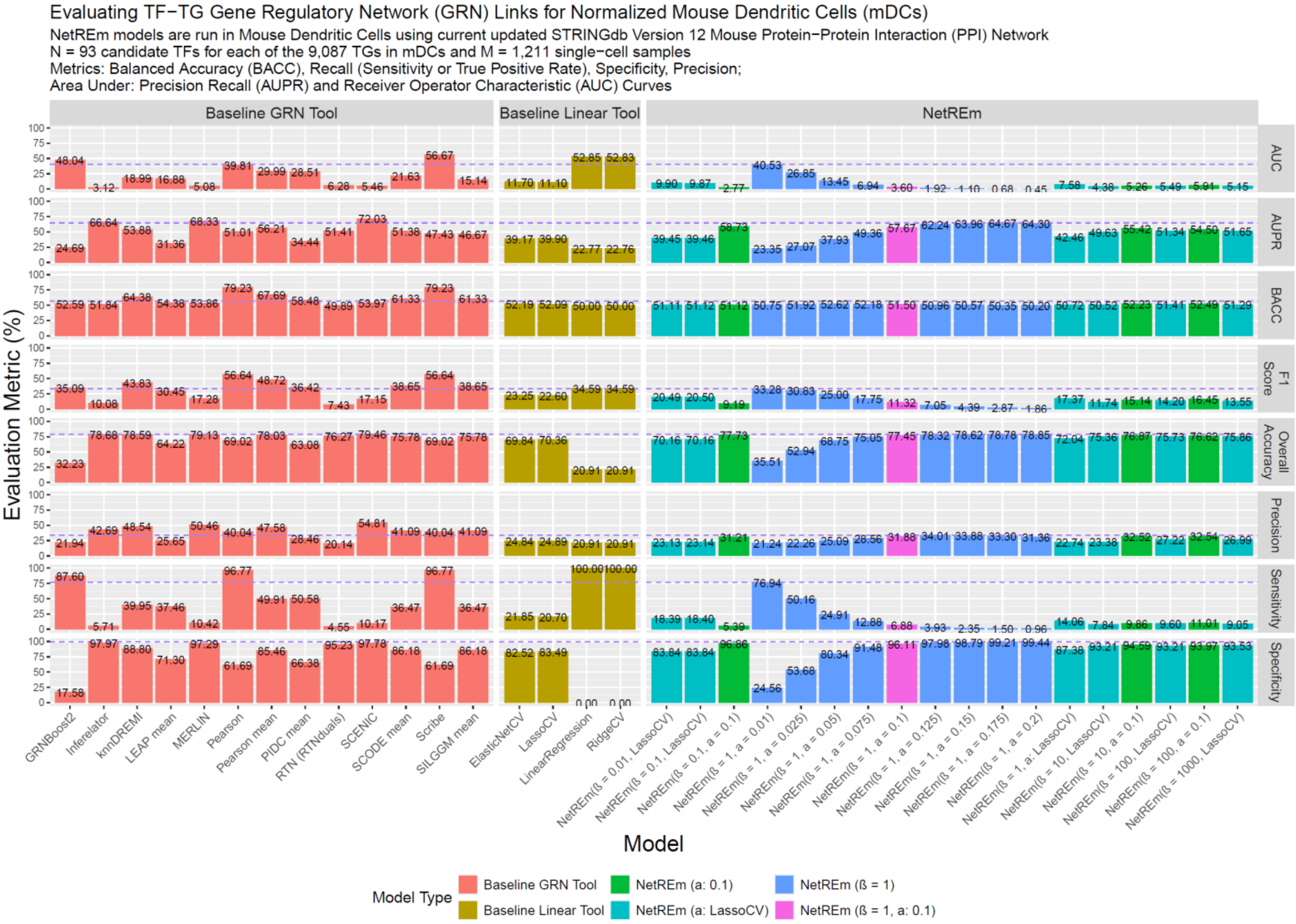

B)

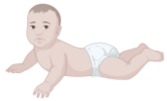

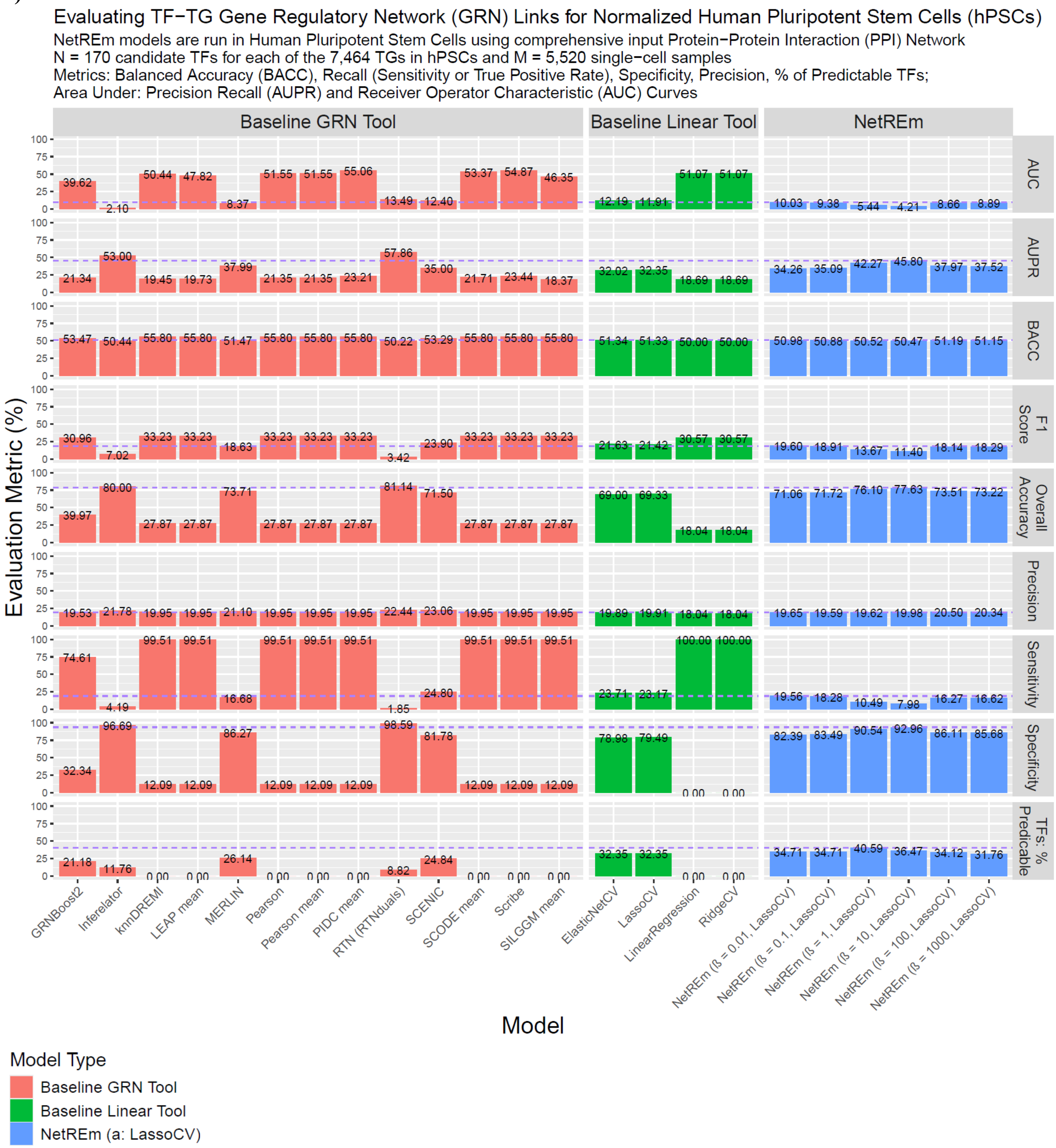
Evaluating TF−TG Gene Regulatory Network (GRN) Links for Normalized Human Pluripotent Stem Cells (hPSCs)
NetREm models are run in Human Pluripotent Stem Cells using comprehensive input Protein−Protein Interaction (PPI) Network
N = 170 candidate TFs for each of the 7,464 TGs in hPSCs and M = 5,520 single−cell samples
Metrics: Balanced Accuracy (BACC), Recall (Sensitivity or True Positive Rate), Specificity, Precision, % of Predictable TFs;
Area Under: Precision Recall (AUPR) and Receiver Operator Characteristic (AUC) Curves
Baseline GRN Tool
Baseline Linear Tool
NetREm
AUC
AUPR
BACC
F1 Score
Overall Accuracy
Precision
Sensitivity
Specificity
TFs: % Predicable
Evaluation Metric (%)
GRNBoost2
Inferelator
knnDREMI
LEAP mean
MERLIN
Pearson
Pearson mean
PIDC mean
RTN (RTNduals)
SCENIC
SCODE mean
Scribe
SILGGM mean
ElasticNetCV
LassoCV
LinearRegression
RidgeCV
NetREm (ß = 0.01, LassoCV)
NetREm (ß = 0.1, LassoCV)
NetREm (ß = 1, LassoCV)
NetREm (ß = 10, LassoCV)
NetREm (ß = 100, LassoCV)
NetREm (ß = 1000, LassoCV)
Model
Model Type
Baseline GRN Tool
Baseline Linear Tool
NetREm (a: LassoCV)

C)

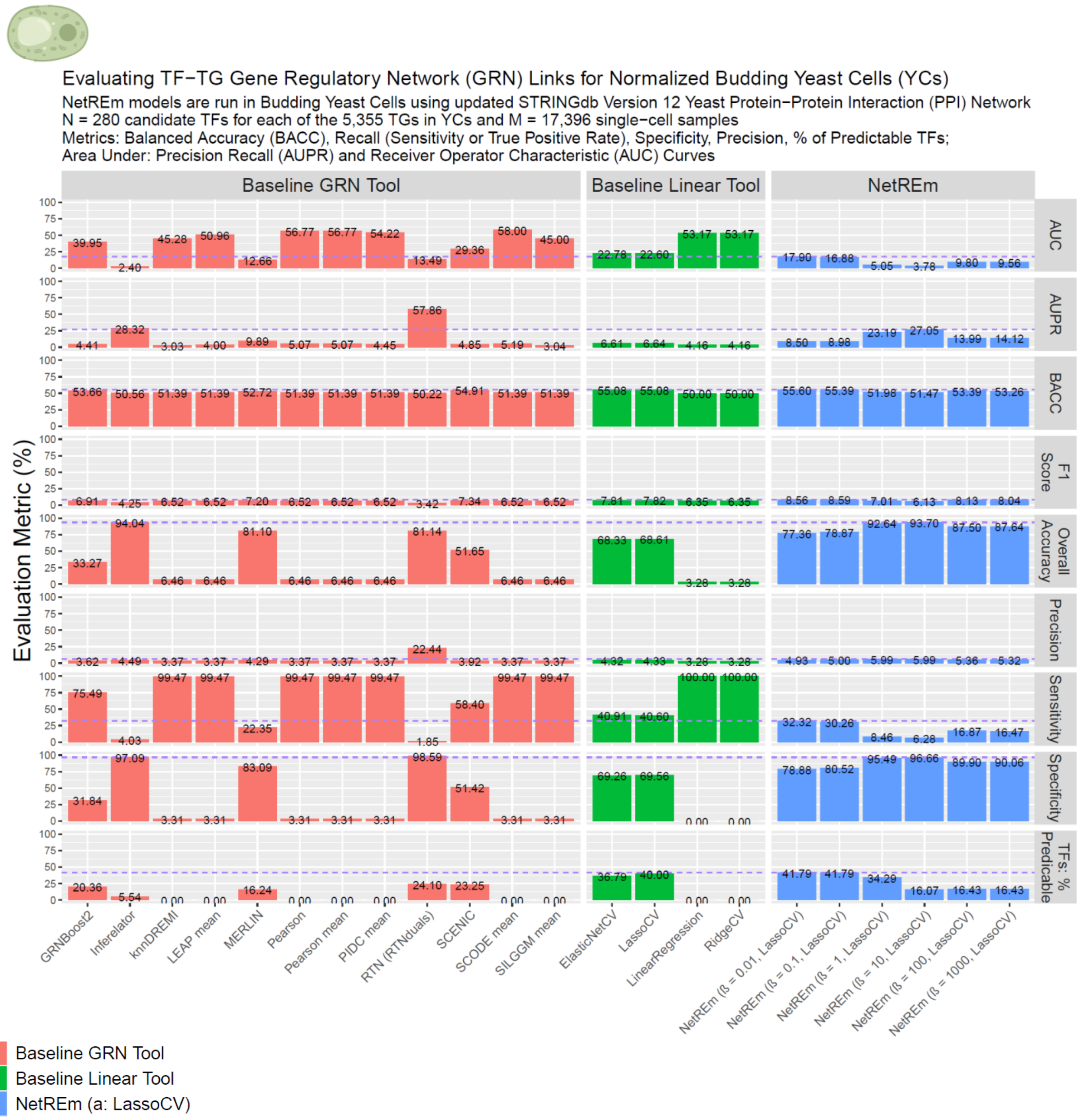

Supplementary Figure 7 – Analyzing NetREm's Predicted TF-TG Regulatory Links in Normalized: Mouse Dendritic Cells (mDCs), Human Pluripotent Stem Cells (hPSCs), and Budding Yeast Cells (YCs).

We analyze NetREm's TF-TG regulatory network performance against known gold standard TF-TG regulatory networks (provided by (McCalla et al. 2023)) in 3 contexts (3 datasets) spanning 3 organisms. This analysis is done in the absence of prior gene regulatory network (GRN) reference information; thus, for all TGs, we have the same list of N candidate TFs (except if the TG is a TF, we simply remove that TF from the list). For comparison, we use state-of-the-art (SOTA) cell-type GRNs predicted by a benchmarking study (McCalla et al. 2023) for these normalized datasets that are predicted based on the following methods: graphical models and dependency networks (Inferelator (Greenfield et. al 2013), MERLIN (Roy et al. 2013), SCENIC (Aibar et. al 2017), SILGGM (Zhang et al. 2018) mean), information theory (knnDREMI (van Dijk et. al 2018), PIDC (Chan et al. 2017) mean, Scribe (Qiu et al. 2020)), ordinary differential equations (SCODE (Matsumoto et al. 2017) mean), correlation r (LEAP (Specht and Li 2017) mean, Pearson, Pearson

mean). Furthermore, the benchmarking study (which we use to obtain our normalized input data, ground truth GRN, and most of the SOTA cell-type GRNs) noted that while these methods tend to be comparable to one another, most of the cell-type GRN methods had a modest recovery of experimentally-derived interactions based on AUPR curve metrics, and adding prior information and estimating the activity of TFs helps improve the performance of cell-type GRN methods like Inferelator and MERLIN. Inferelator, MERLIN, and RTN GRN inference methods have been designed for the older bulk RNA-seq datasets but can be applied for scRNA-seq data; the other SOTA GRNs have been developed specifically for scRNA-seq data. We also predict SOTA GRNs using RTN (an information theoretic method that uses Mutual Information to infer its GRN; it is also used by RTNduals in its downstream analysis) and co-expression based method GRNBoost2 (Moerman et al. 2019). These SOTA GRNs are the Baseline GRN tools on the leftmost panel of these 3 subfigures. In the middle are evaluation results for our 4 Benchmark Regression Models (BRMs) that are applied using the sklearn Python package with no y-intercept: ElasticNetCV, LassoCV, Linear Regression, RidgeCV. On the right are our NetREm models. For all 3 organisms, we train 5 different NetREm models, where network regularization hyperparameter $\beta \in \{0.01; 0.1; 1; 10; 100; 1{,}000\}$ and sparsity-prior hyperparameter α is subsequently optimized by LassoCV. By default, we do not use any y-intercept. In all of our subfigures, for each respective metric we use a horizontal purple dashed line to denote the maximum value of any of the NetREm models for ease of comparison. Please note that the following metrics that we consider are: AUC (area under the receiver operator characteristic (ROC) curve), AUPR (area under the precision-recall curve (AUPR), BACC (balanced accuracy), F1 Score, Overall accuracy, precision, sensitivity (Recall or True Positive Rate), specificity, and % of predictable TFs. All in all, no single method consistently outperforms all other models across all the evaluation metrics for these different datasets and organisms. Based on our performance across these contexts, we note that we may consider NetREm's TF-TG regulatory networks as complementary GRNs. We even note strong or relative performance of NetREm with Inferelator and MERLIN methods that utilize prior knowledge. Overall, NetREm does exhibit strong or comparable performance to SOTA cell-type GRN methods across various metrics. Hence, we may refer to NetREm's TF-TG regulatory networks as complementary GRNs. The main advantage of NetREm is that it additionally predicts TF-TF coordination for TG regulation at not only the overall level (e.g. $\bar{B}$) but also for individual TGs (e.g. TG-specific B). RTNduals may fail to predict TF-TF coordination links for the cell-type and cannot predict this network for individual TGs.

A) Evaluation results for mDCs. We note that we test out results for additional NetREm runs where we fix both β and α.

B) Evaluation results for hPSCs.

C) Evaluation results for YCs. Please note that predictions for the Scribe method were not provided in YCs.

A)

True Target Gene (TG)-Specific TF-TF Coordination Links *B* Discoveries That Are Uncovered by NetREm in Immune Cell-types

Breakdown of the 361 Total Links: 167 Verified Coordinating TF-TF Links for TGs Across 42 Immune Cell Sub-types in Humans (97 unique TF-TF links overall)

Percent (%) of True TF-TF Coordination *B* Links for Target Genes (TGs)

Human Immune System Cell Sub-type	Input Data: # of pooled tissues	Target Gene (TG)										
		CCL2	CCL5	CSF2	CXCL8	IL10	IL18	IL1B	IL23A	IL32	IL6	TNF
CD8b-Positive NKT Cell	1			3.8462								
CD8-Positive, Alpha-Beta Cytokine Secreting Effector T Cell	1									3.704		
CD8-Positive, Alpha-Beta Cytotoxic T Cell	2		50	1.9231						18.52		
CD8-Positive, Alpha-Beta T Cell	2									3.704		
CD8-Positive Alpha-Beta T Cell	16									3.704		
Classical Monocyte	4				4.166667			14.29				
Dendritic Cell	3		25		1.388889		1.8182			7.407		
DN1 Thymic Pro-T Cell	1									3.704		
DN4 Thymocyte	1		12.5	5.7692	15.27778		7.2727	42.86	8.04598	22.22		
Double-Positive, Alpha-Beta Thymocyte	1		62.5		15.27778	12.5	14.545		16.092	11.11		21.05
Erythrocyte	9				4.166667		1.8182		1.14943			
Erythroid Progenitor	3				5.555556		9.0909	14.29				
Granulocyte	2				4.166667			14.29	1.14943		100	
Hematopoietic Stem Cell	4				1.388889		1.8182		1.14943			
Immature Natural Killer Cell	1						1.8182		14.9425			5.263
Immune Cell	2				5.555556		3.6364	14.29		14.81		
Innate Lymphoid Cell	3						1.8182					
Intermediate Monocyte	3				5.555556			14.29				
Langerhans Cell	1	3.774			5.555556		3.6364		19.5402	7.407		
Leukocyte	1			3.8462					1.14943	11.11		
Liver Dendritic Cell	1		37.5				7.2727	14.29		7.407		
Macrophage	24	1.887			9.722222			14.29				5.263
Mast Cell	14						1.8182					
Mature Conventional Dendritic Cell	1	15.09	12.5		11.11111		10.909	14.29		18.52		
Mature NKT Cell	4					25			2.29885	7.407		
Mesenchymal Stem Cell	1	1.887										
Microglial Cell	1				6.944444		1.8182	14.29	1.14943			5.263
Monocyte	10	7.547			9.722222						100	
Myeloid Cell	2				2.777778		3.6364					
Myeloid Dendritic Cell	1						16.364					
Myeloid Progenitor	2				6.944444		5.4545		1.14943			
Naive Regulatory T Cell	1									7.407		
NAMPT Neutrophil	2				6.944444							
Neutrophil	12				2.777778							
NKT Cell	2		25							3.704		
Plasma Cell	20		25		2.777778		1.8182			29.63		
Plasmablast	2				6.944444							
Plasmacytoid Dendritic Cell	5				8.333333	12.5	1.8182		5.74713	3.704	50	
Platelet	2						1.8182	28.57		7.407		36.84
Regulatory T Cell	3									11.11		
T Follicular Helper Cell	1		12.5							3.704		
Type I NKT Cell	3									3.704		

# of True Antagonistic ($B < 0$) TF-TF Coordination Links Uncovered

Human Immune System Cell Sub-type	Target Gene (TG)									
	CCL2	CCL5	CSF2	CXCL8	IL10	IL18	IL1B	IL23A	IL32	TNF
CD8b-Positive NKT Cell										
CD8-Positive, Alpha-Beta Cytokine Secreting Effector T Cell										
CD8-Positive, Alpha-Beta Cytotoxic T Cell		1								
CD8-Positive, Alpha-Beta T Cell										
CD8-Positive Alpha-Beta T Cell										
Classical Monocyte										
Dendritic Cell		1				1			1	
DN1 Thymic Pro-T Cell										
DN4 Thymocyte			2	6		3	3	1	2	
Double-Positive, Alpha-Beta Thymocyte		1		6	1	2		6		4
Erythrocyte										
Erythroid Progenitor						1				
Granulocyte										
Hematopoietic Stem Cell						1				
Immature Natural Killer Cell						1		9		1
Immune Cell				1		2				
Innate Lymphoid Cell										
Intermediate Monocyte										
Langerhans Cell	2			3		1				
Leukocyte										
Liver Dendritic Cell		2				2			2	
Macrophage										
Mast Cell										
Mature Conventional Dendritic Cell	4	1		3		5			2	
Mature NKT Cell									2	
Mesenchymal Stem Cell										
Microglial Cell				1		1				
Monocyte										
Myeloid Cell						1				
Myeloid Dendritic Cell						7				
Myeloid Progenitor						1				
Naive Regulatory T Cell									1	
NAMPT Neutrophil										
Neutrophil										
NKT Cell										
Plasma Cell										
Plasmablast				4						
Plasmacytoid Dendritic Cell				5						
Platelet										
Regulatory T Cell									2	
T Follicular Helper Cell										
Type I NKT Cell										

# of True Cooperative ($B > 0$) TF-TF Coordination Links Uncovered

Human Immune System Cell Sub-type	Target Gene (TG)										
	CCL2	CCL5	CSF2	CXCL8	IL10	IL18	IL1B	IL23A	IL32	IL6	TNF
CD8b-Positive NKT Cell			2								
CD8-Positive, Alpha-Beta Cytokine Secreting Effector T Cell									1		
CD8-Positive, Alpha-Beta Cytotoxic T Cell		3	1						5		
CD8-Positive, Alpha-Beta T Cell									1		
CD8-Positive Alpha-Beta T Cell									1		
Classical Monocyte				3			1				
Dendritic Cell		1		1					1		
DN1 Thymic Pro-T Cell									1		
DN4 Thymocyte		1	1	5		1		6	4		
Double-Positive, Alpha-Beta Thymocyte		4		5		6		8	3		
Erythrocyte				3		1		1			
Erythroid Progenitor				4		4	1				
Granulocyte				3			1	1		2	
Hematopoietic Stem Cell				1				1			
Immature Natural Killer Cell								4			
Immune Cell				3			1		4		
Innate Lymphoid Cell						1					
Intermediate Monocyte				4			1				
Langerhans Cell				1		1		17	2		
Leukocyte			2					1	3		
Liver Dendritic Cell		1				2	1				
Macrophage	1			7			1				1
Mast Cell						1					
Mature Conventional Dendritic Cell	4			5		1	1		3		
Mature NKT Cell					2			2			
Mesenchymal Stem Cell	1										
Microglial Cell				4			1	1			1
Monocyte	4			7						2	
Myeloid Cell				2		1					
Myeloid Dendritic Cell						2					
Myeloid Progenitor				5		2		1			
Naive Regulatory T Cell									1		
NAMPT Neutrophil				5							
Neutrophil				2							
NKT Cell		2							1		
Plasma Cell		2		2		1			8		
Plasmablast				1							
Plasmacytoid Dendritic Cell				1	1	1		5	1	1	
Platelet						1	2		2		7
Regulatory T Cell									1		
T Follicular Helper Cell		1							1		
Type I NKT Cell									1		

B)

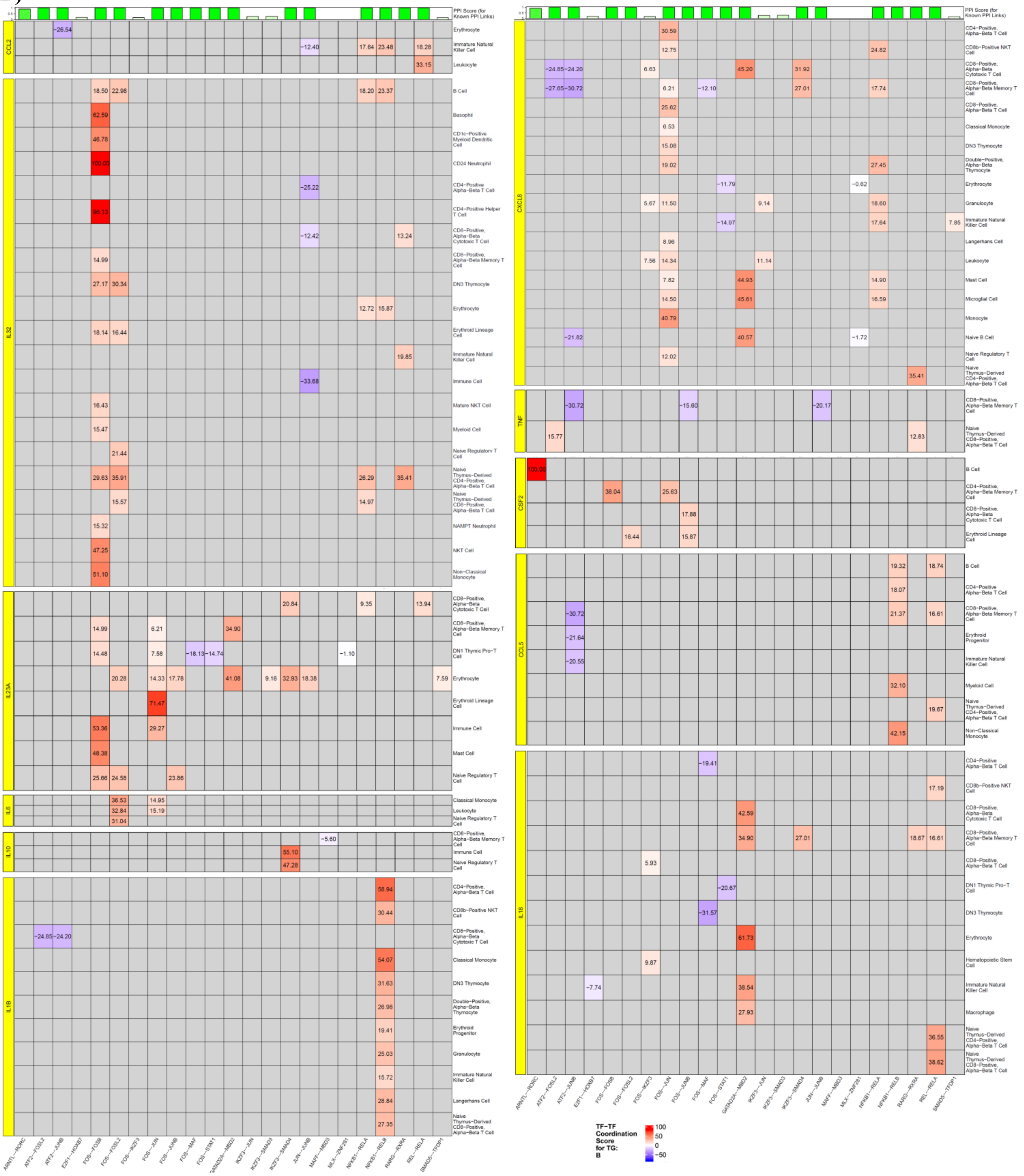
PPI Score (for Known PPI Links)
TF–TF Coordination Score for TG: B
100
50
0
−50

Supplementary Figure 8 – Validating TG-Specific TF-TF Coordination Links in Human Immune Cell Subtypes Using Experimental Data on Paired Yeast One-Hybrid (pY1H) Assays

Please note that we showcase NetREm's unprecedented ability to identify TG-specific TF-TF coordination links B for diverse contexts. For each TG, NetREm predicts TF-TG regulatory links (that can be stitched up into the cell-type TF-TG regulatory network) and TF-TF coordination ($-100 \leq B \leq 100$, which is aggregated to form the overall cell-type TF-TF coordination network $-100 \leq \bar{B} \leq 100$). In general, it is easier to validate cell-type $\bar{B}$ links than it is to validate TG-specific coordination at the cell-type level since B is more granular; nonetheless, it is still quite difficult to even validate $\bar{B}$ (but we focus on those challenges in later figures and tables). In this case, we try to validate NetREm's TG-specific TF-TF coordination links B and present an example of how this may be done (as the datasets for this validation become increasingly available in the future). We note that these TG-specific coordination networks are unprecedented and that NetREm is a pioneer method in this respect. To do this, we utilize experimental findings from a recent study that employed Paired Yeast One-Hybrid (pY1H) experimental Assays to detect DNA-binding cooperativity and antagonism across human TFs. This study focused especially on the immune system in humans and on a few cytokine TGs and found relationships among 137 TFs that they reported. Here, we run NetREm(β = 100, α = 0.1) for TGs (e.g. CCL2, CCL5, CSF2, CXCL8, IL10, IL18, IL1B, IL23A, IL32, IL6, TNF) on 62 immune cell subtypes provided by the Tabula Sapiens atlas (264,824 cells pooled from various tissues) using the Main NetREm Input PPIN for 125 TFs (out of the 137 TFs that are found in the expression data). Please note that this ground truth data lacks cell-type and sub-cell-type specificity. We focus on presenting examples of TFs co-regulating the TGs where the nature of TF-TF coordination (e.g. cooperation or antagonism) is also correct. That is, we highlight $(\mathrm{TF}_i - \mathrm{TF}_j)$ $-$TG links where both TFs co-regulate TG and the nature of their interaction (cooperation or antagonism) is correct.

A) We obtain results for 11 TGs across 42 immune cell subtypes. Please note that we present the metrics for the 97 different TF-TF links that we uncover. There are 167 unique TF-TF to TG links (regardless of cell-type) that are experimentally verified (correct assignment of TG and of TF-TF interaction). These 167 $(\mathrm{TF}_i - \mathrm{TF}_j)$ $-$TG links comprise 94 cooperative PPIs and 73 antagonistic PPIs. Altogether (across the 42 immune cell subtypes), these are 361 unique interactions. For instance, we correctly predict: both cooperative links (FOS-JUN, FOS-FOSL2) for regulating *IL6* in granulocytes and monocytes, 3 of 5 antagonistic links for regulating *IL1B* in Dn4 Thymocytes, 5 of 8 true TF-TF links (1 of 3 antagonistic, 4 of 5 cooperative) for regulating *CCL5* in double-positive, alpha-beta thymocytes. This table provides additional metrics for these 97 links for these 11 TGs across the 42 immune cell subtypes.

B) Please note that we focus on 25 (out of the 97) different TF-TF interactions and show the valid (TF_i- TF_j)-TG predictions for coordination B: accurate predictions that both TFs co-regulate and their nature of coordination (e.g. cooperative or antagonistic). We show the TG-specific TF-TF coordination values for their respective TGs across different immune cell subtypes. The x-axis has these 25 different TF-TF coordination interactions. We split results into 2 columns for visual simplicity. The heatmap is split up separately for each of the 11 TGs (mentioned by the yellow annotation bars on the left). The y-axis has the relevant cell-types. And the values are the TG-specific TF-TF coordination B, which are blue for negative values and red for positive values as B goes from -100 (antagonism) to 100 (cooperation). We show the TG-specific nature of TF-TF interactions, such as JUN-JUNB cooperativity to coregulate *IL23A* but antagonism to co-regulate *TNF*, *IL32*, *CCL2*. Similarly, FOS-JUNB are antagonistic co-regulators of TNF but cooperative co-regulators of *IL23A* and *CSF2*. Another example is how ATF2 and FOSL2 cooperate to co-regulate TNF but are antagonistic when co-regulating *CXCL8* and *IL1B* across immune cell subtypes. Furthermore, we note that NetREm uncovers novel PPIs, such as MAFF and MBD3 (that have an input PPI score of 0 in the Main NetREm PPIN as shown by the green bar plot above the heatmaps) antagonistically co-regulating *IL10* in CD8-positive, alpha-beta memory T cells.

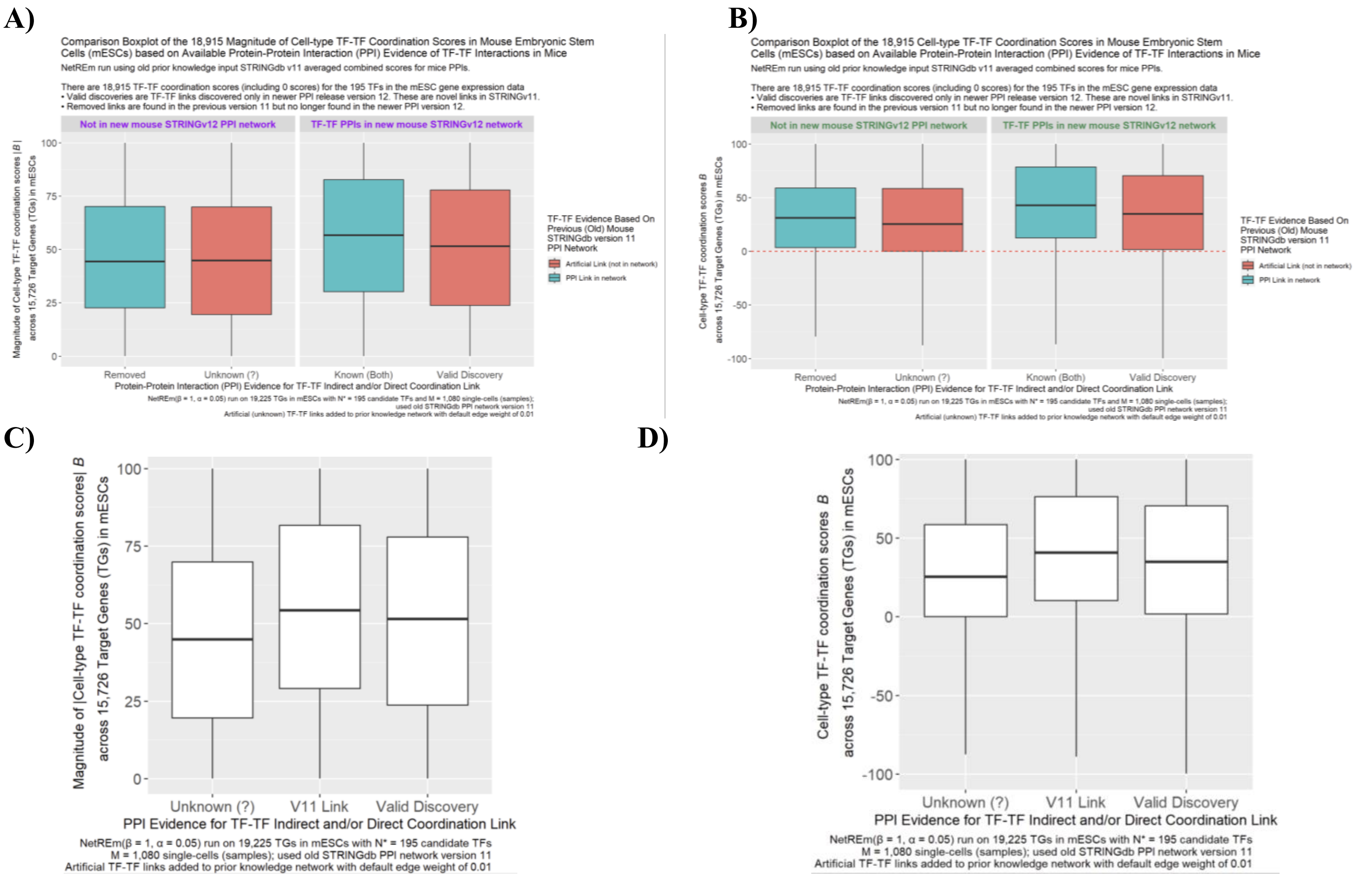

Supplementary Figure 9 – Analyzing NetREm's ability to prioritize and predict future Protein-Protein Interaction (PPI) Links in Mouse Embryonic Stem Cells (mESCs).

For mESCs, the fully-connected TF-TF PPIN in V11 contains 5,044 known and 13,871 artificial edges among 195 candidate TFs; V12 contains 6,856 known, 12,059 artificial edges; 25 singleton TFs in V11 PPIN have known PPIs in V12, indicating evolution from V11 to V12. 4 comparison boxplots of 18,915 Cell-type TF-TF coordination scores ($\bar{B}$) in mESCs based on older STRING PPIN V11 as input to NetREm. STRINGdb considers direct as well as indirect PPIs (i.e. functional associations) among TFs. Outliers are removed from the boxplot visuals. Input: v11 mouse PPIN. Evaluation: updated v12 mouse PPIN. These 4 panels analyze results of NetREm($\beta = 1, \alpha = 0.05$) on 19,225 TGs in mESCs (each have single-cell gene expression measures $M = 1{,}080$ cells) using same $N^* = 195$ candidate TFs for each TG; when the TG is also a TF, that TF is removed and NetREm is run using 194 TGs. Missing pairwise TF-TF links are added to the input network (with default edge weight $\eta = 0.01$) to make the network fully-connected. We ran NetREm with older STRING v11 PPIN as a proxy for older info (v12 PPIN used to gauge NetREm's ability to detect future links).

- *Valid discoveries* are found only in the updated STRING db v12 and were unknown in older STRING v11 (input); such discoveries show NetREm's ability to learn future indirect/direct TF-TF PPIs that may be currently unknown.
- *Unknown links* have not been mentioned in both networks (i.e. STRING v11 and STRING v12 do not mention them):
  - such links could nonetheless be *future discoveries* (False Negatives FNs) or truly True Negatives (TNs: no meaningful direct/indirect interaction truly exists between them in biology).
  - *V11 links* are found in STRING v11: these links may still be in V12 networks: *Known (both)* or may have been *False Positives (FPs)* if they are no longer found in the newer STRING v12 network: *Removed*).

A-B focuses on the distribution of TF-TF coordination scores across these 4 potential groups: Removed, Unknown, Known (Both), and Valid Discovery.

A) focuses on magnitude of $\bar{B}$: $0 \le |\bar{B}| \le 100$.

B) original mESC TF-TF coordination scores: $-100 \le \bar{B} \le 100$.

C-D collapses the Known (both) and Removed links into V11 category and focuses on distribution of TF-TF coordination scores across these 3 groups: Unknown, V11 links, Valid Discovery.
C) focuses on the magnitude: $0 \leq |\bar{B}| \leq 100$. **D)** presents the original: $-100 \leq \bar{B} \leq 100$. The # of edges: 18,915 is based on: $(1/2)\ (N)(N-1)$. Please note: associated Statistical Tests for this analysis can be found in **Supp. Tables S15-16.**

A)

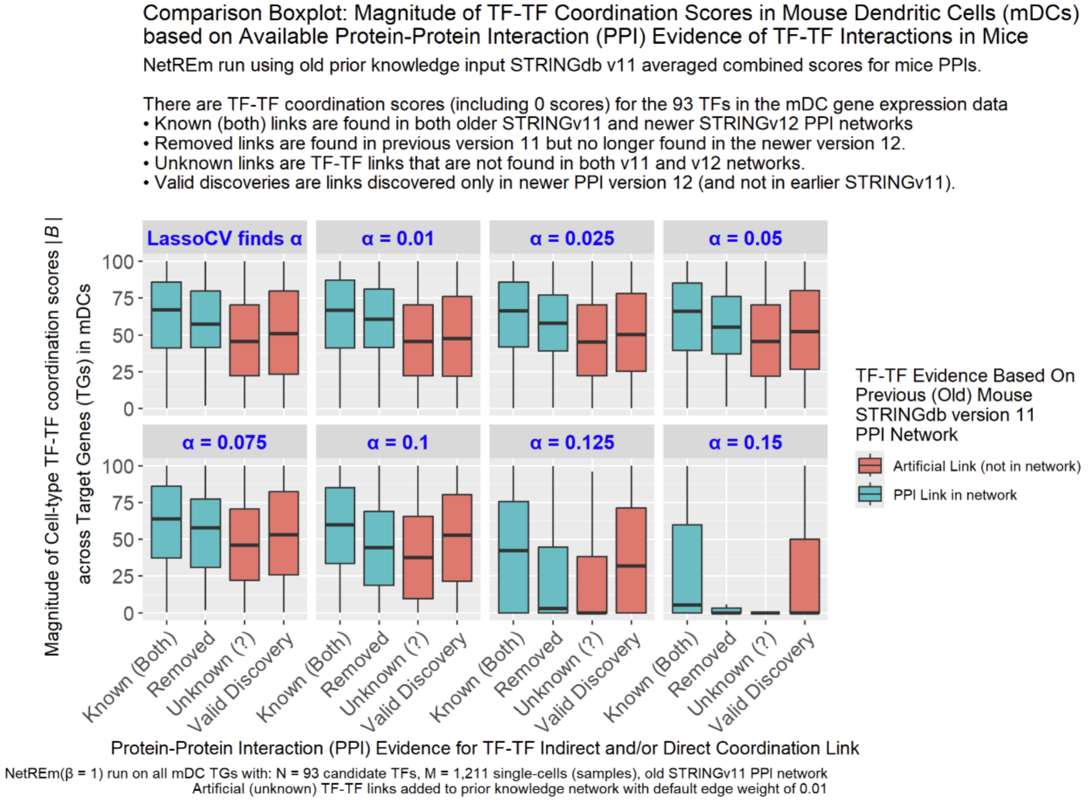

B)

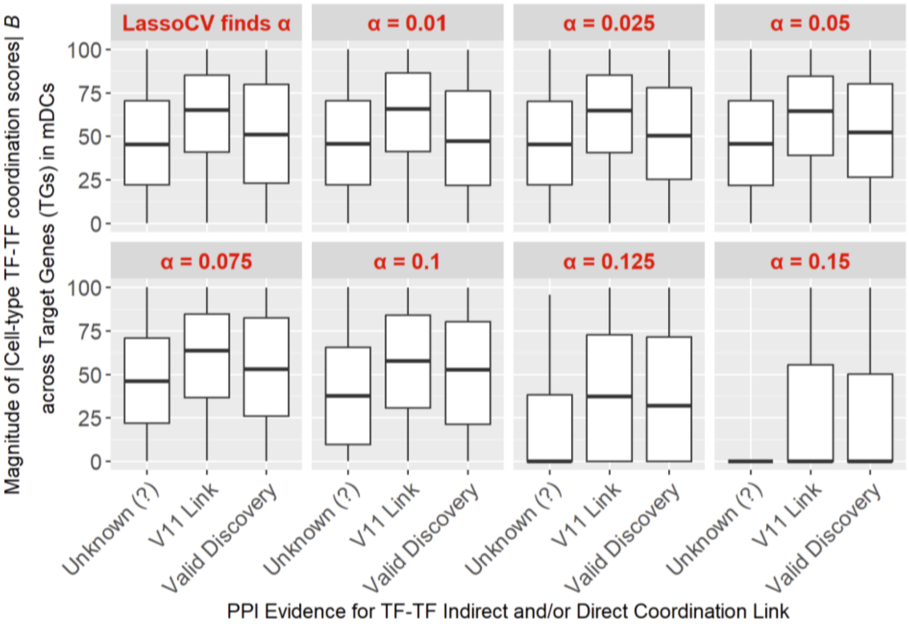

Supplementary Figure 10 – Analyzing NetREm's ability to prioritize and predict future Protein-Protein Interaction (PPI) Links in Mouse Dendritic Cells (mDCs).

This is similar to **Fig. S9**, but we are focusing on mDCs and on visualizing impacts for different sparsity prior hyperparameter α values. Please see reference **Table S15** (adapted for mESCs) and **Tables S17-18**.

A)

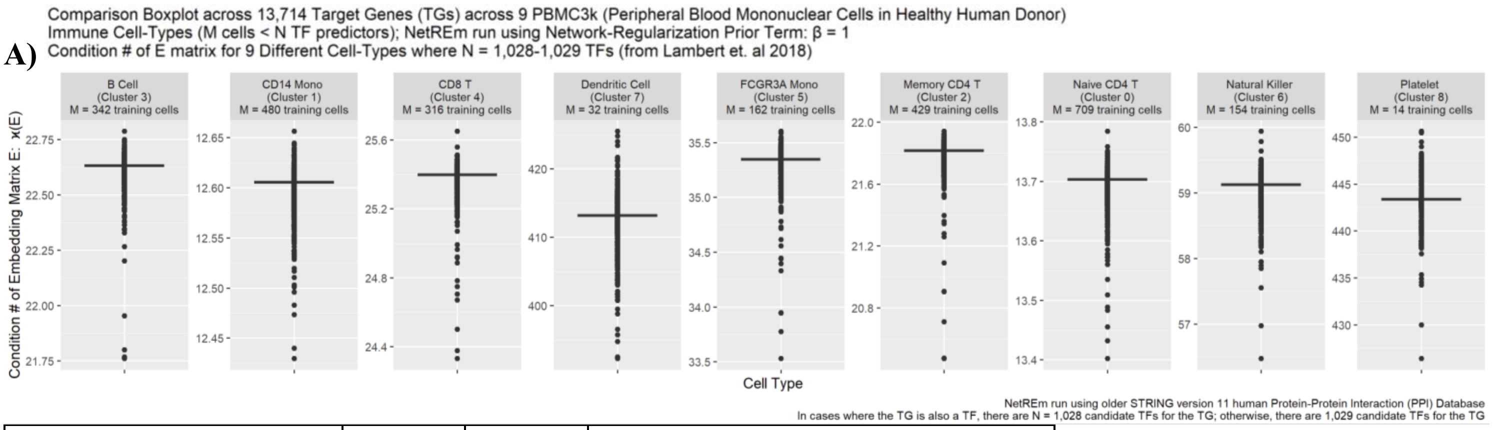

Cell-type	*N*	*M*	Maximum Condition # across TGs
PBMC Naive_CD4_T-C0	1,029	709	13.8
PBMC CD14_Mono_C1	1,029	480	12.7
PBMC Memory_CD4_T-C2	1,029	429	21.9
PBMC B_C3	1,029	342	22.8
PBMC CD8_T_C4	1,029	316	25.6
PBMC FCGR3A_Mono_C5	1,029	162	35.6
PBMC Natural_Killer_C6	1,029	154	59.9
PBMC Dendritic_Cell_C7	1,029	32	425
PBMC Platelet_C8	1,029	14	451

B)

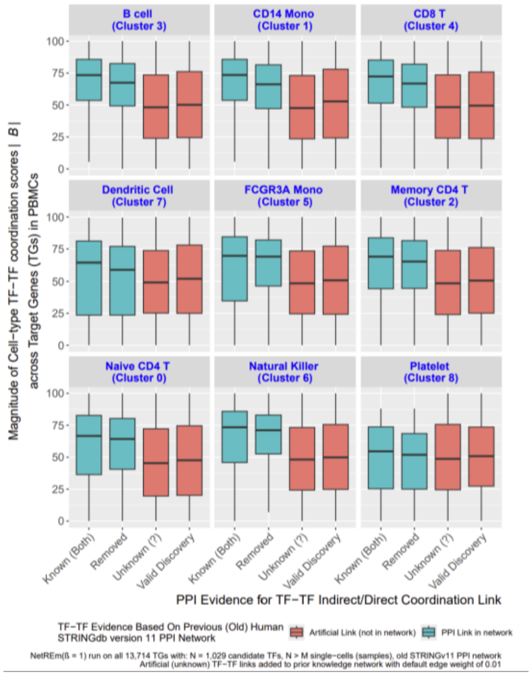

C)

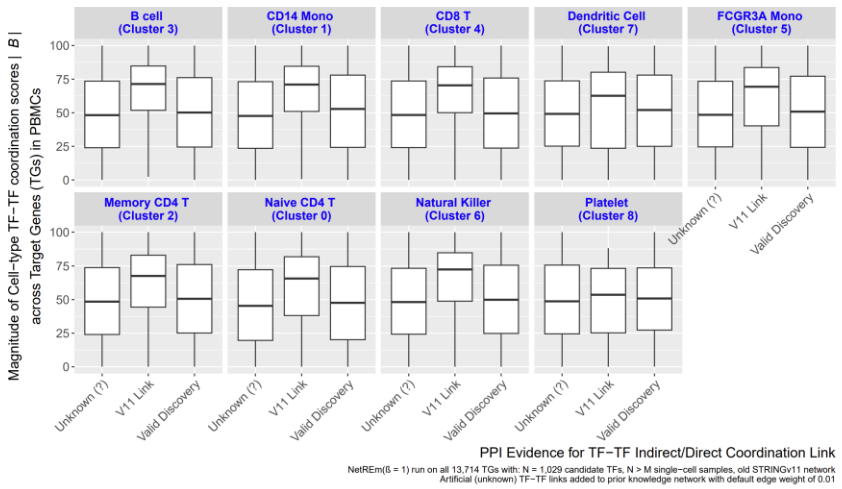

Supplementary Figure 11 – NetREm TF-TF Coordination Performance for 9 different Human Immune Cell Types: PBMCs: Peripheral Blood Mononuclear Cells.

For PBMCs, we intersect the 13,714 TGs with (Lambert et al. 2018) to uncover 1,029 candidate TFs, which we say is $\mathcal{N}$. Then, for most TGs, $N = \mathcal{N} = 1{,}029$ and if the TG is also 1 of the 1,029 TFs, then $N = \mathcal{N} - 1 = 1{,}028$ candidate TFs. We run NetREm using the older version 11 STRINGdb PPI network.

A) Comparison boxplot across 13,714 target genes across 9 PBMC Immune cell-types (M cells < N = 1,029 candidate TFs). NetREm is run using $\beta = 1$ and LassoCV to optimize α. We can see the condition #s over the 13,714 TGs for each of these 9 types. Please note the max condition # of the E matrix is: $\max(\kappa(E))$ = Naive CD4 T (Cluster 0): 13.8, CD14

Mono (Cluster 1): 12.7, Memory CD4 T (Cluster 2): 21.9, B (Cluster 3): 22.8, CD8 T (Cluster 4): 25.6, FCGR3A Mono (Cluster 5): 35.6, Natural Killer (Cluster 6): 59.9, Dendritic Cell (Cluster 7): 425, Platelet (Cluster 8): 451. Since $\max(\kappa(E)) \ll 1e6$, all cells are very well-conditioned and therefore are numerically stable. In particular, the $M = 14$ versus N = 1,029 case for Cluster 8 (Platelets) illustrates this, given that $M \ll N$.

B) Comparison Boxplot: Magnitude of TF-TF coordination scores in 9 human immune system cell-types based on available protein-protein interaction (PPI) evidence. This is an application in Peripheral Blood Mononuclear Cells (PBMCs) from a healthy human donor. PBMCs based on available PPI evidence. NetREm is run using the older V11 PPIN and evaluated using the updated human PPI network (including STRINGv12 network and other resources) on TF-TF PPIs. There are TF-TF coordination scores including 0 for 1,029 TFs in respective gene expression data. The known (both) links are found in the older V11 and newer PPI networks. Removed links are found in the previous V11 but no longer are found in the newest PPI network (e.g. V12 and beyond). Unknown links are those that are not found in both networks. Valid discoveries (VDs) are not in V11 but uncovered in the newest network (PPI network version 12 and not in the earlier STRINGdb v11). The y-axis is the magnitude of cell-type TF-TF coordination scores $|\bar{B}|$.

C) Please note that this is the same as part **Fig. S11B**, except we collapse 2 TF-TF subgroups. Essentially, the V11 links could be known links (in V11 and newest network) or they could be removed. The other 2 (Unknown and Valid discoveries) are still focusing on links not in the older V11 network: still not in the newest network (if they are unknown) or have just been discovered in the newest network (if they are valid discoveries).

A)

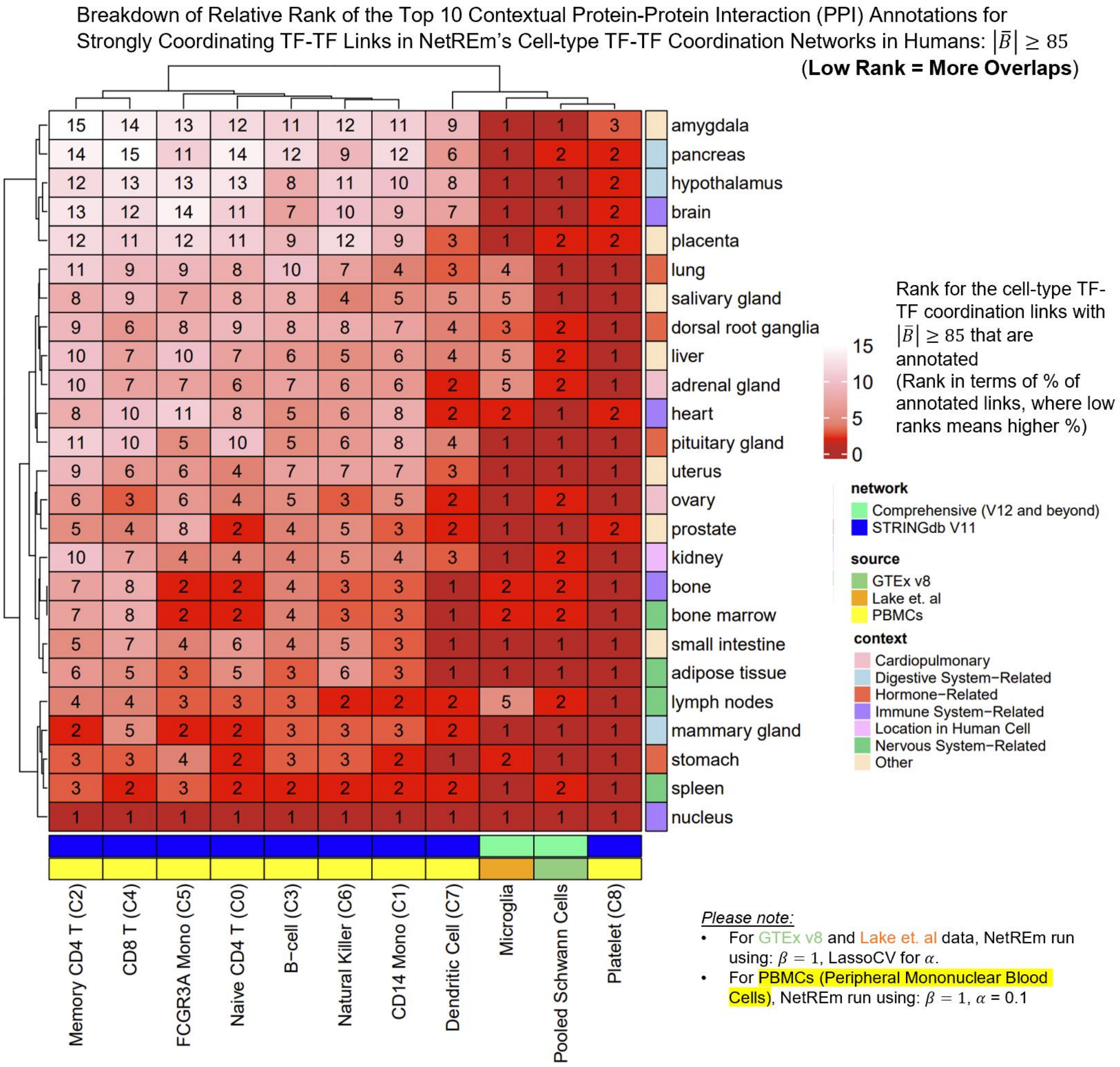
Breakdown of Relative Rank of the Top 10 Contextual Protein-Protein Interaction (PPI) Annotations for Strongly Coordinating TF-TF Links in NetREm's Cell-type TF-TF Coordination Networks in Humans: $|\bar{B}| \geq 85$
(**Low Rank = More Overlaps**)
amygdala 15 14 13 12 11 12 11 9 1 1 3
pancreas 14 15 11 14 12 9 12 6 1 2 2
hypothalamus 12 13 13 13 8 11 10 8 1 1 2
brain 13 12 14 11 7 10 9 7 1 1 2
placenta 12 11 12 11 9 12 9 3 1 2 2
lung 11 9 9 8 10 7 4 3 4 1 1
salivary gland 8 9 7 8 8 4 5 5 5 1 1
dorsal root ganglia 9 6 8 9 8 8 7 4 3 2 1
liver 10 7 10 7 6 5 6 4 5 2 1
adrenal gland 10 7 7 6 7 6 6 2 5 2 1
heart 8 10 11 8 5 6 8 2 2 1 2
pituitary gland 11 10 5 10 5 6 8 4 1 1 1
uterus 9 6 6 4 7 7 7 3 1 1 1
ovary 6 3 6 4 5 3 5 2 1 2 1
prostate 5 4 8 2 4 5 3 2 1 1 2
kidney 10 7 4 4 4 5 4 3 1 2 1
bone 7 8 2 2 4 3 3 1 2 2 1
bone marrow 7 8 2 2 4 3 3 1 2 2 1
small intestine 5 7 4 6 4 5 3 1 1 1 1
adipose tissue 6 5 3 5 3 6 3 1 1 1 1
lymph nodes 4 4 3 3 3 2 2 2 5 2 1
mammary gland 2 5 2 2 3 3 3 2 1 1 1
stomach 3 3 4 2 3 3 2 1 2 1 1
spleen 3 2 3 2 2 2 2 2 1 2 1
nucleus 1 1 1 1 1 1 1 1 1 1 1
Memory CD4 T (C2)
CD8 T (C4)
FCGR3A Mono (C5)
Naive CD4 T (C0)
B-cell (C3)
Natural Killer (C6)
CD14 Mono (C1)
Dendritic Cell (C7)
Microglia
Pooled Schwann Cells
Platelet (C8)
15
10
5
0
Rank for the cell-type TF-TF coordination links with $|\bar{B}| \geq 85$ that are annotated
(Rank in terms of % of annotated links, where low ranks means higher %)
network
Comprehensive (V12 and beyond)
STRINGdb V11
source
GTEx v8
Lake et. al
PBMCs
context
Cardiopulmonary
Digestive System−Related
Hormone−Related
Immune System−Related
Location in Human Cell
Nervous System−Related
Other
Please note:
• For GTEx v8 and Lake et. al data, NetREm run using: $\beta = 1$, LassoCV for α.
• For PBMCs (Peripheral Mononuclear Blood Cells), NetREm run using: $\beta = 1$, α = 0.1

B)

TF pair	B cell (C3)	CD14 Mono (C1)	CD8 T-cell (C4)	Dendritic Cell (C7)	FCGR3A Mono (C5)	Memory CD4 T-cell (C2)	Naive CD4 T-cell (C0)	Natural Killer (C6)	Platelet (C8)
EBF1−E2F5	85.09								
IRF7−BATF3	63.90	98.05		−89.01	−1.12	33.85			
IRF8−IRF1	70.08	94.78	71.61	−88.51	17.78	−29.42	31.04	89.25	
IRF8−IRF7	11.76	4.41	0.82	95.28	−4.41	92.89	80.46	−58.00	
JUNB−CXXC5	96.81	−34.36	−85.79	−1.27	−4.74	85.38	−67.61	−76.44	68.05
JUND−CEBPB	37.16	−95.37	−6.38	−87.28	3.49	66.57	85.77	−11.01	60.39
JUND−FOSB	42.67	95.29	−72.20	87.45	−93.53	−51.00	32.28	−0.89	34.10
KLF6−ELF1	75.57	−4.41	0.03	82.82	−88.86	−85.30	91.86	−3.26	76.11
RXRA−JUND	56.39	−64.53	69.20	−2.82	−34.43	37.96	−5.15	2.66	59.94
RXRA−KLF6	89.66	92.78	−57.55	38.68	−32.32	−1.50	95.62	−89.40	86.15
SPI1−ELF1	88.90	61.32	−56.61	−95.19	−68.90	−1.42	67.62	6.70	80.08
TBX21−GATA3	98.09	65.98	70.32		71.27	42.04	92.88	91.22	
TBX21−MYC	76.80	67.05	−0.45		70.39	30.86	99.18	1.51	
TCF4−IRF1	20.44	−0.63		−83.82	92.38	29.79	88.95	87.84	
TCF4−REL	49.66	50.60		−86.06	1.85	81.98	16.19	63.63	
TCF7−GATA3	41.84	81.24	90.02	63.40	−5.91	−3.85	89.34	77.51	
TCF7−LEF1	90.82	53.74	82.28		22.39	82.31	90.22	88.40	86.19
TFEC−OLIG1		79.19							
TSHZ2−GATA3	12.50	42.01		58.52	45.47	0.70	84.83		
TSHZ2−LEF1	22.98	96.99			60.37	77.25	88.18		
TSHZ2−SATB1	28.61	94.69		17.78	44.10	−18.53	98.00		
VDR−TFEC		81.13							
YBX3−POU2F2	−96.42	83.71	86.36	−71.29	−39.87	90.65	84.95	48.26	−32.91
ZEB2−BATF3	96.74	86.91		−82.49	0.80	24.55	51.17		
ZNF532−POU2AF1	52.42								
ZNF860−E2F5	86.26								
FOS_CXXC5	10.18	−61.56	83.08	6.12	−32.81	1.34	84.45	−93.81	73.01
RUNX1_NFIL3		59.76	65.49	63.85	70.75	24.24	55.33	99.79	
JUN_FOS	99.06	99.66	99.88	96.42	95.53	99.88	99.98	97.44	74.87
GATA3_CDC5L	79.04	75.30	0.85	64.11	76.54	87.79		92.85	
POU2AF1_IRF4	89.75		53.42	−0.07	69.08	67.78	55.42		
NFE2_CEBPA		86.82	20.17		−88.46		9.78	37.61	17.33
TBX21_NFE2		82.77	57.60		92.96		4.06	77.98	
BACH1_ATF1	78.73	88.61	82.45	28.64	94.82	65.31	69.69	77.02	
CEBPD_BATF3	82.54	81.41		10.74	92.35	23.11	65.70		
OLIG1_ELF1		57.43			−89.33				
OLIG1_CEBPD					90.61				
VENTX_AHR		74.82			97.83				
OLIG1_HIF1A					96.84				
ZEB2_VENTX					54.46				
OLIG1_MAFB					−88.22				
SPIB_SMAD4	84.47	18.20	21.49	−76.88	53.05	99.90	51.26		
TCF7_MYC	23.25	76.08	93.13	−33.62	87.21	−60.15		72.16	
NFATC3_KMT2A	31.16	85.37	39.23	14.35	88.62	89.43	3.16	81.15	
TSHZ2_MYC	56.58	71.67		61.10	71.14	92.43	79.05		
ZFP90_FOXP3			58.45			90.00	70.12		
TEF_FOXP3			2.04			16.11	5.35		
CEBPE_AHR		70.01			34.56		39.21		
TFEC_OLIG1		79.19							
VDR_TFEC		81.13							
SPI1_JUND	83.73	−99.50	92.15	85.10	−35.89	97.65	51.27	44.68	60.67
VENTX_ETS2		95.86			79.92				
ZNF230_HOXA5			13.82						
ZNF101_LEF1	85.82	71.75	98.76		86.26	28.40	33.07	2.49	
ZNF540_JUNB	−1.38	−76.17	87.80			−10.20	−78.51		
BCL6_BATF3	50.77	0.32		92.55	58.56	25.75	52.76		
IRF8_IRF4	−60.78	70.03	58.40	76.70	0.28	65.15	56.75	75.33	
TCF4_IRF8	−33.55	88.51		88.55	−57.47	77.26	59.55	58.88	
TCF4_SPIB	89.60	69.70		93.66	85.10	99.90	65.94		
ZNF789_USF2	87.98	88.30	93.69	85.17	32.17	25.70	81.73	95.72	

cell_type

Cell−Type TF−TF Coordination Score $\bar{B}$

100, 50, 0, −50, −100 — cooperative (positive), antagonistic (negative)

Cell-type that Both Coordinating TFs are Cell Markers For:

- AXL+SIGLEC6+ dendritic cell
- B cell
- CD141+CLEC9A+ dendritic cell
- CD8+ T cell
- Central memory CD4+ T cell
- Central memory T cell
- Dendritic cell
- Effector memory CD4+ T cell
- Memory B cell
- Monocyte
- Multilymphoid progenitor cell
- Myeloid cell
- Myeloid dendritic cell
- Naive CD4 or CD4+ T cell
- Naive CD8+ T cell
- Natural killer cell
- Plasma cell
- Plasmacytoid dendritic cell

9 PBMC Sub-types

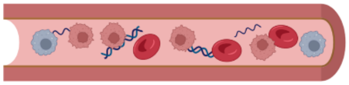

Supplementary Figure 12 – Evaluating the Context-Specificity of Annotations for Top TF-TF Coordination Links

A) Contextual PPI Integrated Interactions Database (IID) Annotations for Top TF-TF Coordination $\bar{B}$ Links overall: We note that the TF-TF coordination networks can prioritize cell-specific TF-TF coordination. Based on our revisions to our method, we now have cell-type TF-TF coordination scores that can fall between -100 and 100 (i.e. $|\bar{B}| \leq 100$). To show this, we do the following analysis for several cell-types in humans. For the given cell-type, we retrieve cell-type TF-TF links that are relatively strong $|\bar{B}| \geq 85$. We overlay those with annotated TF-TF links in the contextual Integrated Interactions Database (IID)(Kotlyar et. al 2022), computing the % of top TF-TF links enriched for each of the 240 different annotations. Then, we rank the results, where low ranks represent a greater %. We note all the top TF-TF links are predicted to localize in the nucleus, consistent with their functions as TFs. For instance, in Microglia and pooled Schwann cells (SCs), we find our strong TF-TF coordination links are more typically more enriched for nervous system-related terms (e.g. dorsal root ganglia, brain, hypothalamus, amygdala) than 9 immune sub-populations in PBMCs (immune-related cells) are. These PBMC cell-types tend to have strong TF-TF coordination links that are most strongly enriched for immune-related terms (e.g. spleen, bone marrow, lymph nodes, adipose tissue). We note how different PBMC sub-populations have more enrichment (lower rank values) for certain immune-related terms than others, possibly suggesting their localization and functions in the immune system. Given that we use Pooled GTEx SCs for this analysis (which are gathered from 5 tissues: esophagus mucosa/muscularis, heart, prostate, skeletal muscle), our top TF-TF coordination links for SCs tend to be highly enriched for relevant tissues (e.g. heart, prostate).

B) Heatmap of select top cell-type TF-TF coordination links $\bar{B}$ for the 9 different PBMC sub-types (where $-100 \leq \bar{B} \leq 100$). The columns correspond to the 9 different PBMC subtypes. The rows correspond to the different TF-TF coordination links. Cells in red are positive TF-TF coordination links (cooperative) and those in blue are negative links (antagonistic). The $TF_i - TF_j$ links are annotated based on available data from the CellMarker 2.0 database(Hu et al. 2023); that is, we annotate the links based on the presence of both TFs as cell-type markers. This database is a manually curated collection of experimentally supported genes that serve as markers of various cell-types across humans and mice. In particular, we aim to highlight the presence of strong PBMC subtype TF-TF coordination links for TFs that are markers of the relevant cell-type. For instance, the top row focuses on the EBF1-E2F5 TF-TF coordination link that is only present in B cells (and not in any of the other 8 PBMC subtypes) and cooperative ($\bar{B} = 85.09$); further, both EBF1 and E2F5 are cell-type markers for B-cells based on (Hu et al. 2023). The same is true ZNF860-E2F5, for example.

A)

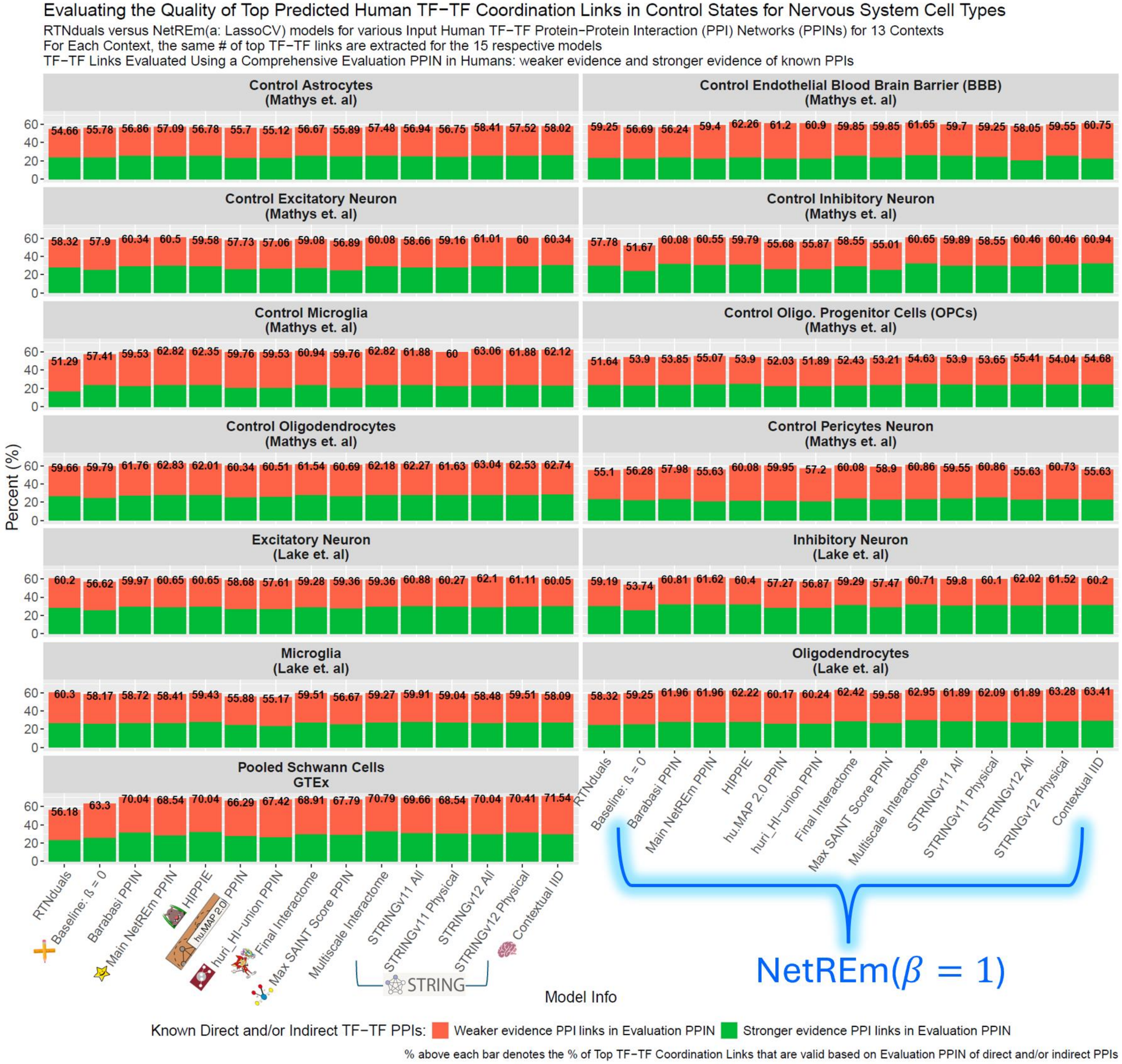

Evaluating the Quality of Top Predicted Human TF−TF Coordination Links in Control States for Nervous System Cell Types
RTNduals versus NetREm(a: LassoCV) models for various Input Human TF−TF Protein−Protein Interaction (PPI) Networks (PPINs) for 13 Contexts
For Each Context, the same # of top TF−TF links are extracted for the 15 respective models
TF−TF Links Evaluated Using a Comprehensive Evaluation PPIN in Humans: weaker evidence and stronger evidence of known PPIs
Control Astrocytes (Mathys et. al)
54.66 55.78 56.86 57.09 56.78 55.7 55.12 56.67 55.89 57.48 56.94 56.75 58.41 57.52 58.02
Control Endothelial Blood Brain Barrier (BBB) (Mathys et. al)
59.25 56.69 56.24 59.4 62.26 61.2 60.9 59.85 59.85 61.65 59.7 59.25 58.05 59.55 60.75
Control Excitatory Neuron (Mathys et. al)
58.32 57.9 60.34 60.5 59.58 57.73 57.06 59.08 56.89 60.08 58.66 59.16 61.01 60 60.34
Control Inhibitory Neuron (Mathys et. al)
57.78 51.67 60.08 60.55 59.79 55.68 55.87 58.55 55.01 60.65 59.89 58.55 60.46 60.46 60.94
Control Microglia (Mathys et. al)
51.29 57.41 59.53 62.82 62.35 59.76 59.53 60.94 59.76 62.82 61.88 60 63.06 61.88 62.12
Control Oligo. Progenitor Cells (OPCs) (Mathys et. al)
51.64 53.9 53.85 55.07 53.9 52.03 51.89 52.43 53.21 54.63 53.9 53.65 55.41 54.04 54.68
Control Oligodendrocytes (Mathys et. al)
59.66 59.79 61.76 62.83 62.01 60.34 60.51 61.54 60.69 62.18 62.27 61.63 63.04 62.53 62.74
Control Pericytes Neuron (Mathys et. al)
55.1 56.28 57.98 55.63 60.08 59.95 57.2 60.08 58.9 60.86 59.55 60.86 55.63 60.73 55.63
Excitatory Neuron (Lake et. al)
60.2 56.62 59.97 60.65 60.65 58.68 57.61 59.28 59.36 59.36 60.88 60.27 62.1 61.11 60.05
Inhibitory Neuron (Lake et. al)
59.19 53.74 60.81 61.62 60.4 57.27 56.87 59.29 57.47 60.71 59.8 60.1 62.02 61.52 60.2
Microglia (Lake et. al)
60.3 58.17 58.72 58.41 59.43 55.88 55.17 59.51 56.67 59.27 59.91 59.04 58.48 59.51 58.09
Oligodendrocytes (Lake et. al)
58.32 59.25 61.96 61.96 62.22 60.17 60.24 62.42 59.58 62.95 61.89 62.09 61.89 63.28 63.41
Pooled Schwann Cells GTEx
56.18 63.3 70.04 68.54 70.04 66.29 67.42 68.91 67.79 70.79 69.66 68.54 70.04 70.41 71.54
Percent (%)
0 20 40 60
RTNduals
Baseline: β = 0
Barabasi PPIN
Main NetREm PPIN
HIPPIE
hu.MAP 2.0 PPIN
huri_HI−union PPIN
Final Interactome
Max SAINT Score PPIN
Multiscale Interactome
STRINGv11 All
STRINGv11 Physical
STRINGv12 All
STRINGv12 Physical
Contextual IID
STRING
NetREm(β = 1)
Model Info
Known Direct and/or Indirect TF−TF PPIs: Weaker evidence PPI links in Evaluation PPIN Stronger evidence PPI links in Evaluation PPIN
% above each bar denotes the % of Top TF−TF Coordination Links that are valid based on Evaluation PPIN of direct and/or indirect PPIs

B)

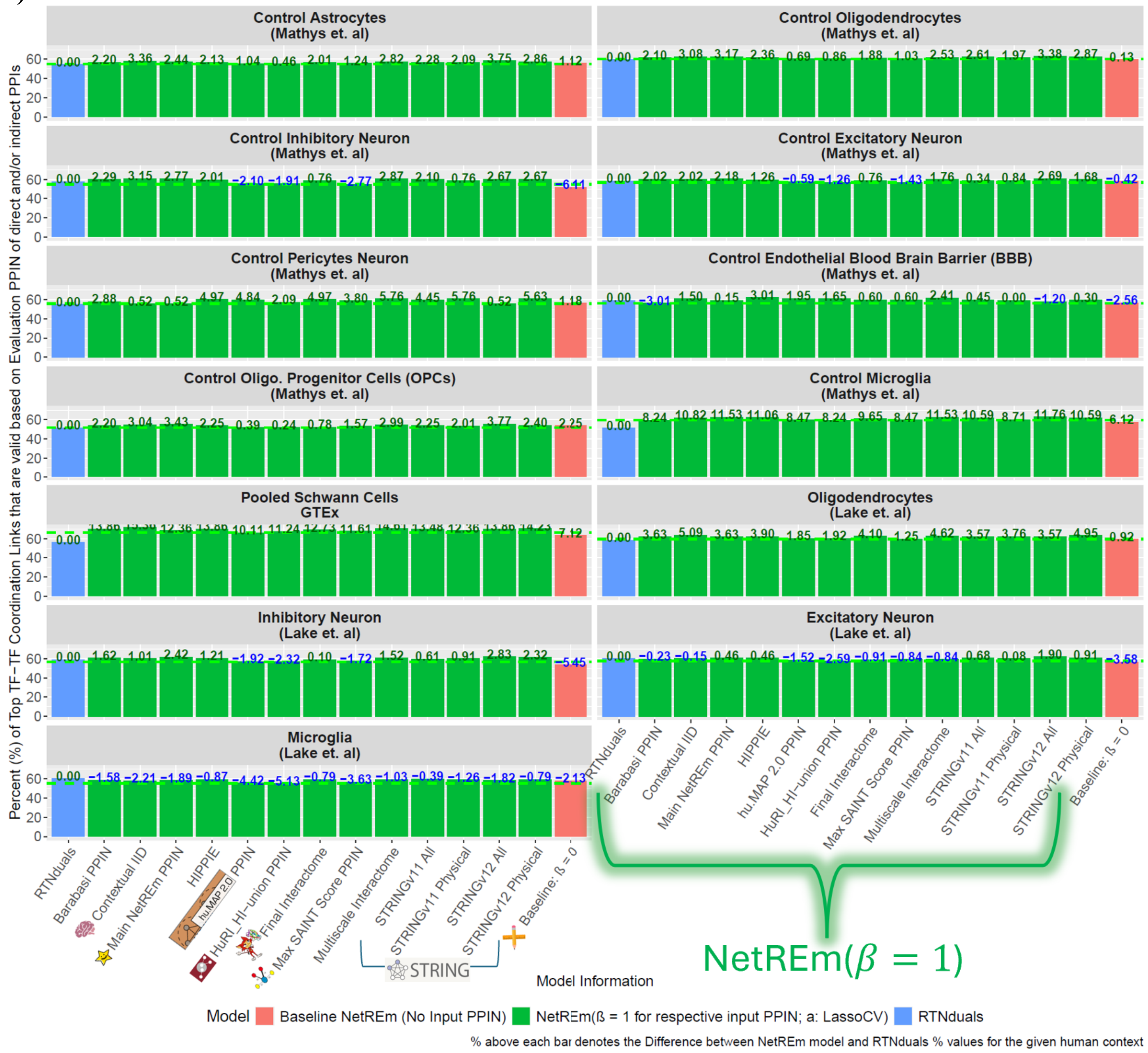

Supplementary Figure 13 – Benchmark: Compare predicted TF-TF Links for RTNduals versus NetREm for various input PPI Networks

Evaluating the quality of top predicted human TF-TF coordination network $\bar{B}$ links for 13 different contexts (control states for cell-types in the nervous system). We run 13 different NetREm models with $\beta = 1$ and α subsequently optimized by Cross-Validation (CV, default: 5-fold CV, using the LassoCV function). For each of these 13 NetREm$_{\beta = 1}$ models, we use a different input PPI network (PPIN) with weights $0.01 \leq w \leq 1$, where weights with $w = \eta = 0.01$ are added in as artificial weights with this minimum default edge weight to ensure that W is fully-connected. We have a baseline model (baseREm: NetREm run with positive $\beta \approx 0$ (i.e. $\beta = 1e - 100$) and α is subsequently optimized via LassoCV). That is, in baseREm, we essentially run NetREm with virtually no (or infinitely small) prior PPIN knowledge; baseREm helps us understand and gauge the role of prior PPIN knowledge in predicting how TFs coordinate to co-regulate TGs. That is, baseREm uses the NetREm pipeline in the absence of prior PPIN regularization; for some instances, we have required the use of our Truncated SVD approach. We run RTNduals for each of these 13 different human contexts. We retrieve a certain # of final TF-TF links by RTNduals(Chagas et al. 2019), say k. Then, we go to our predicted cell-type TF-TF coordination network (for our given NetREm$_{\beta=1}$ model and/or baseREm model) and extract the top k links in terms of

their magnitude of cell-type TF-TF coordination scores $|\bar{B}|$. So, we ultimately compare NetREm's top k links with RTNduals' final k links. We do this for each of our 13 $\text{NetREm}_{\beta=1}$ models and for baseREm.

Then, we compare our top TF-TF links (for each of the 13 $\text{NetREm}_{\beta=1}$ models and the baseREm model) against RTNduals using an Evaluation PPIN that we build using several resources. This Evaluation PPIN has annotated edges as stronger evidence links and weaker evidence links (middle evidence and/or weakest evidence links), which we explain in the *Supplementary Methods*. TF-TF coordination can be indirect or direct. Our Evaluation PPIN accounts for TF-TF coordination links that correspond to direct/indirect PPIs. Thus, for each of the 13 human nervous system-related contexts, for each of these 15 methods (13 $\text{NetREm}_{\beta=1}$ models, 1 baseREm model, RTNduals model) for TF-TF coordination, we utilize the same # of predicted TF-TF links derived from the same input list of context-specific TFs: k. This number of k is context-specific and depends on what RTNduals deems is significant and what it returns; we keep our # of rows consistent with RTNduals to facilitate an equal comparison. We then evaluate the % of these k links are found in the Evaluation PPIN. Then, for validated predictions, we see what the strength of evidence is for each of the predictions. For each of the 13 $\text{NetREm}_{\beta=1}$ models, the validated predictions may comprise True Positive links (that were already in the existing respective input PPI network) as well as novel discoveries (False Negatives: not in the input PPIN but found in the Evaluation PPIN). Some links in the input PPIN may be False Positives and removed in the Evaluation PPIN. Thus, we are also indirectly gauging how many of the top TF-TF links may be poor results (False Positives or still unknown: not in input PPIN and not in Evaluation PPIN either). Our 13 input PPINs are fairly diverse! We note that both figures A and B present different ways of visualizing and interpreting the breakdown of the % of top k TF-TF links that are validated.

A) The % of top k TF-TF links (of direct and/or indirect coordination for the context overall) that are validated. Each of these facets represents 1 of the 13 human contexts. The x-axis lists the 15 different models (with PPI Network logos given if applicable), where the first 2 are for comparisons: RTNduals and baseREm (baseline NetREm). These are stacked barplots where the bottom bar (green) refers to the % of the top k links that have stronger evidence (supporting their indirect and/or direct interaction) in the comprehensive Evaluation PPIN. The red bar on top represents % of the links with weaker evidence (middle or weakest level of evidence). The # on top of each bar refers to the height of the stacked bar and is the cumulative % of top links that are validated (with any degree of evidence) in the Evaluation PPIN.

B) Please note that this is a follow-up of the previous figure but focuses on direct comparison of each of the 13 $\text{NetREm}_{\beta = 1}$ models and baseREm model with RTNduals for each context. Here, we are comparing the difference in the % of top k TF-TF links that are validated in the Evaluation PPIN. The # on top of the bar represents the difference between the 14 different models (13 $\text{NetREm}_{\beta = 1}$ models and baseREm) and RTNduals; the # above the bar is negative and in blue if RTNduals is winning (a higher % of validated links) and is green if RTNduals is losing (a lower % of validated links). Please note that the green dashed line is the minimum value from any of the 13 $\text{NetREm}_{\beta=1}$ models, to assist with visual comparison. This highlights the benefit of our methodology that incorporates PPINs, regardless of the input PPIN. That is, network-regularized regression studies emphasize the value of incorporating prior knowledge of interactions among the predictors regardless of how accurate or complete such networks may be. Such prior networks are meaningful points of departure for the model.

A)

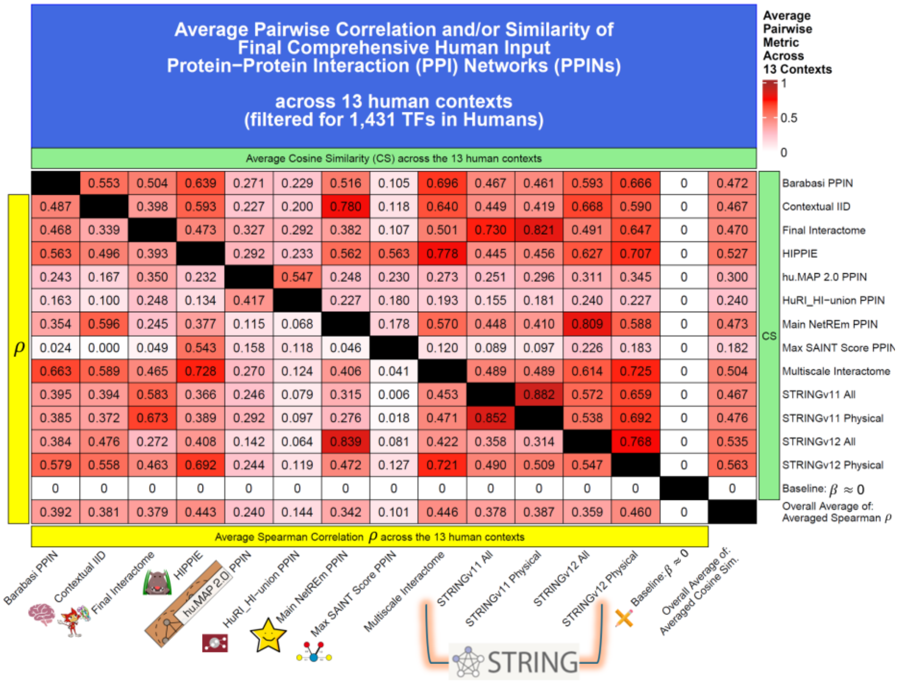

Here, these input PPINs exhibit a range of diversity, with average ρ ranging from 2.45e-5 to 0.85 (average 0.316) and average cosine similarity CS ranging from 0.0887 to 0.8824 (average 0.40).

B)

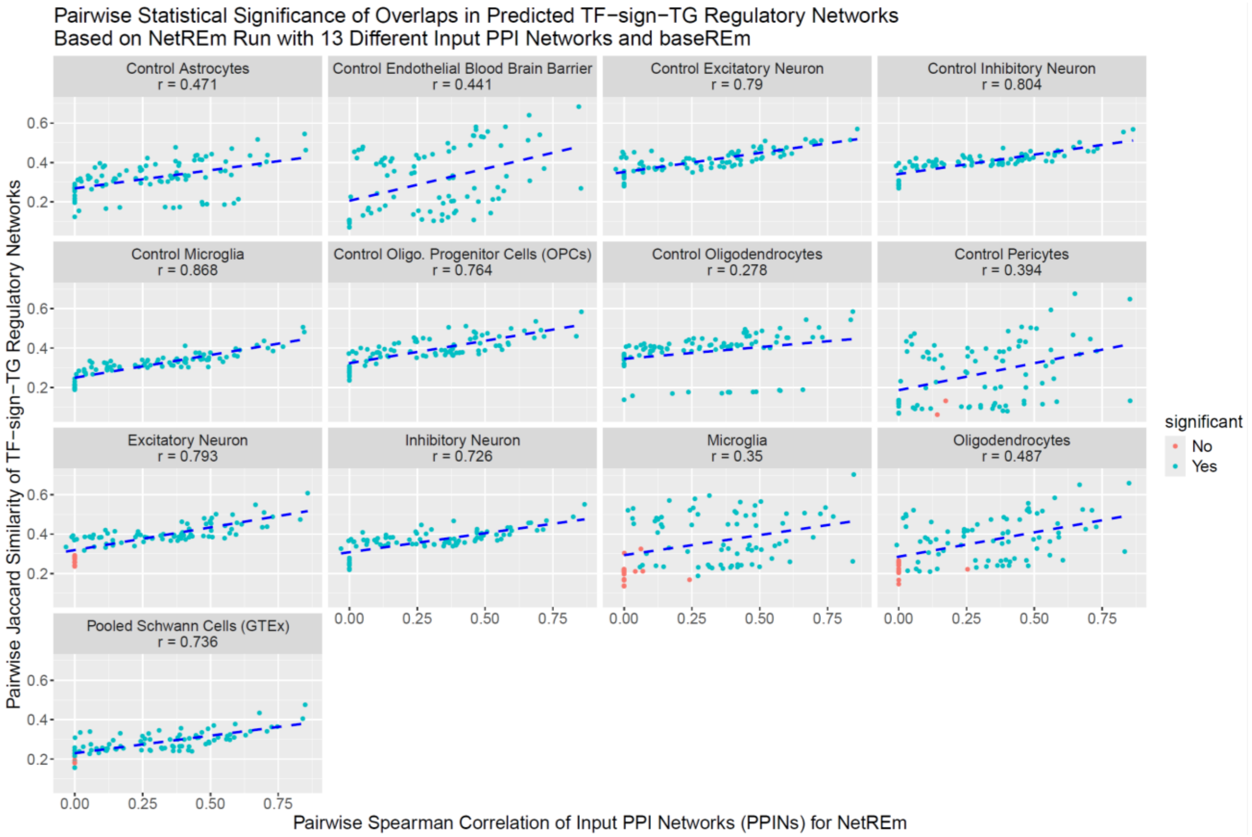

The strength of this trend depends on the context and goes from 0. We are showing the correlation between Pairwise Spearman Correlation of input PPI Networks and the Pairwise Jaccard Similarity of TF-sign-TG regulatory networks for each of the 13 human contexts. Here, the x-values are 0 for the 13 comparisons of baseREm model with NetREm models.

C)

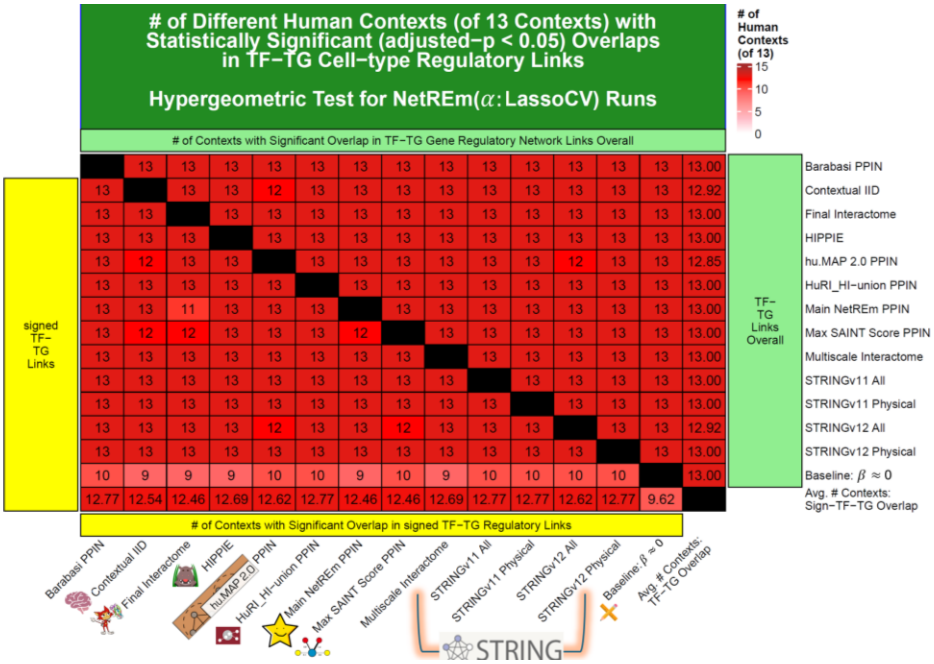

For each of the 91 pairwise comparisons, we perform a hypergeometric test of the statistical significance of overlaps in signed TF-TG links (bottom left of matrix) and of overlaps in the simple TF-TG GRN links overall (top right of matrix) across the 13 human contexts. In this statistical testing, we perform multiple testing correction, so we only count contexts where the adjusted p-value < 0.05. We find that almost all 14 models (13 NetREm-based and baseREm) have statistically significant overlaps when we consider the simple TF-TG GRN links overall, without analyzing the actual direction of the sign of the TFs (i.e. the TF roles as either an activator or a repressor). Thus, we perform a more granular analysis and consider the sign of the TF (i.e. coefficient c^* sign: activator (+) or repressor (-)) in the GRN link, hence evaluating the signed TF-TG links. We find that most of the 13 NetREm-based models with input PPINs tend to have statistically significant overlaps in signed TF-TG links with each other for at least 11 out of the 13 contexts (compared with baseREm that only has similarity in 9 to 10 contexts).

D)

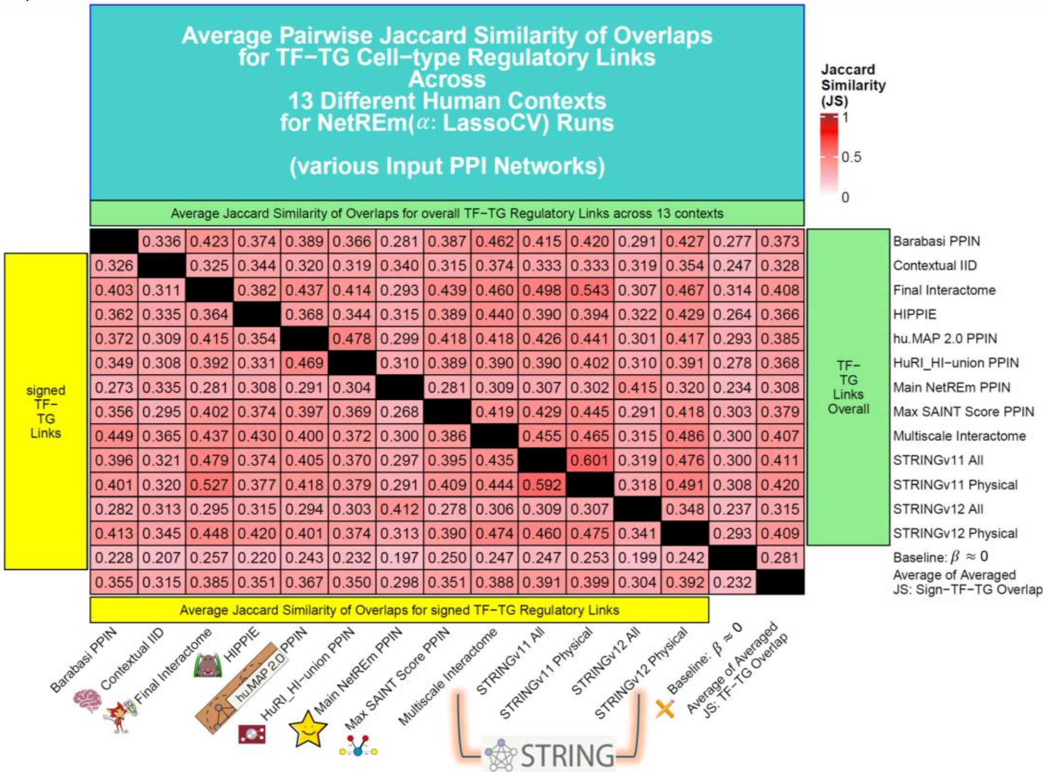

Average Pairwise Metric across 13 human contexts	Aggregate measure Applied to Pairwise Metric	78 pairwise comparisons (amongst 13 NetREm models)	13 pairwise comparisons of NetREm models with baseREm
Jaccard Similarity (JS) of TF-TG GRN Links	min	0.281269	0.233918
	max	0.600824	0.31446
	average	0.382948	0.280623
	median	0.38883	0.292618
JS of Signed TF-TG Links	min	0.268144	0.196661
	max	0.591531	0.257049
	average	0.367814	0.232382
	median	0.36931	0.241844

We compare how similar the final overall cell-type TF-TG regulatory networks are in terms of predicting TF-TG regulatory relationships (top right of the matrix) and how similar they are in terms of predicting signed TF-TG regulatory links (bottom left of the matrix). We aggregate the measures for the average Jaccard similarity of TF-TG regulatory links for the 78 comparisons among NetREm$_{\beta=1}$ models and find the following metrics: (min: 0.281269, max: 0.600824, average: 0.382948, median: 0.38883). We can compare this with the resulting aggregate measures for the average Jaccard similarity of TF-TG regulatory links for the 13 comparisons between NetREm$_{\beta=1}$ and baseREm models (min: 0.233918, max: 0.31446, average: 0.280623, median: 0.292618) and find that NetREm's pairwise results are higher than those for 13 comparisons. In addition, we aggregate the measures for the average Jaccard similarity of signed-TF-TG regulatory links for the 78 comparisons among NetREm$_{\beta=1}$ models and find the following metrics: (min: 0.268144, max: 0.591531, average: 0.367814, median: 0.36931). We can compare this with the resulting aggregate measures for the average Jaccard similarity of TF-TG regulatory links for the 13 comparisons between NetREm$_{\beta=1}$ and baseREm models (min: 0.196661, max: 0.257049, average: 0.232382, median: 0.241844) and find that NetREm's pairwise results are much higher than those for 13 comparisons.

E)

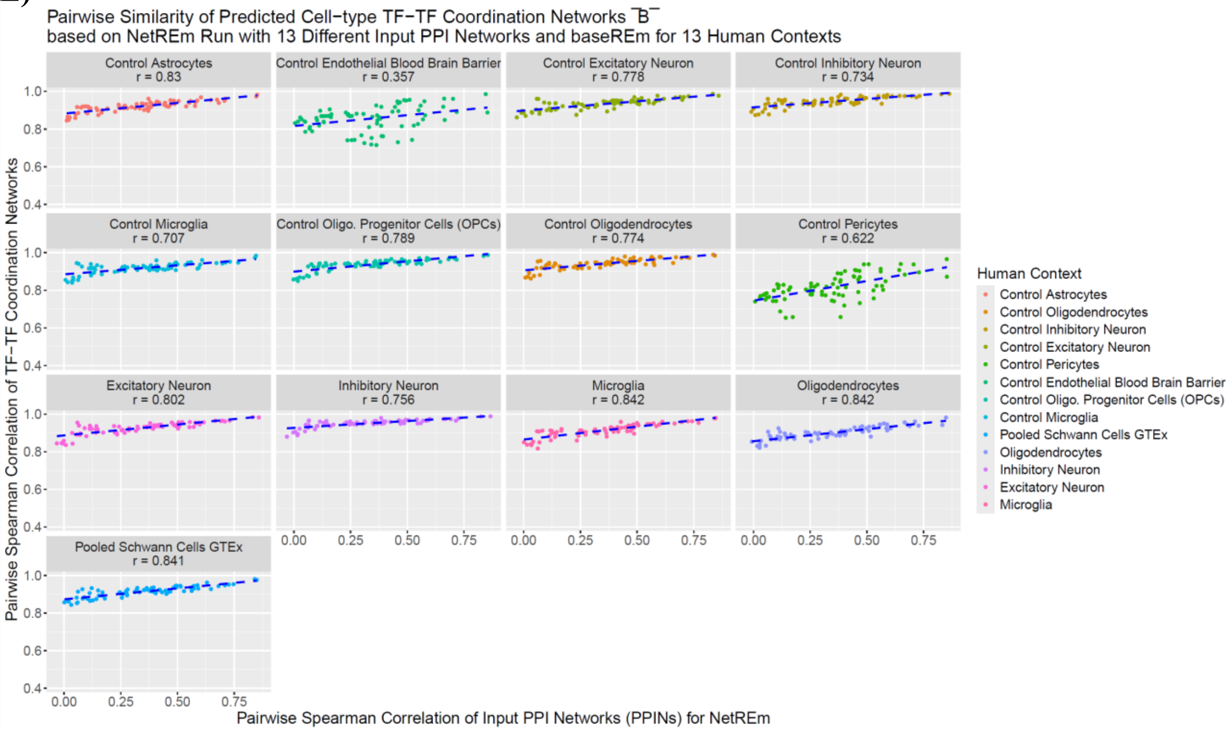

The strength of this trend depends on the context and goes from 0. We are showing the correlation between Pairwise Spearman Correlation of input PPI Networks and the Pairwise Spearman Correlation of TF-TF coordination networks for each of the 13 human contexts. Here, the x-values are 0 for the 13 comparisons of baseREm model with NetREm models.

F)

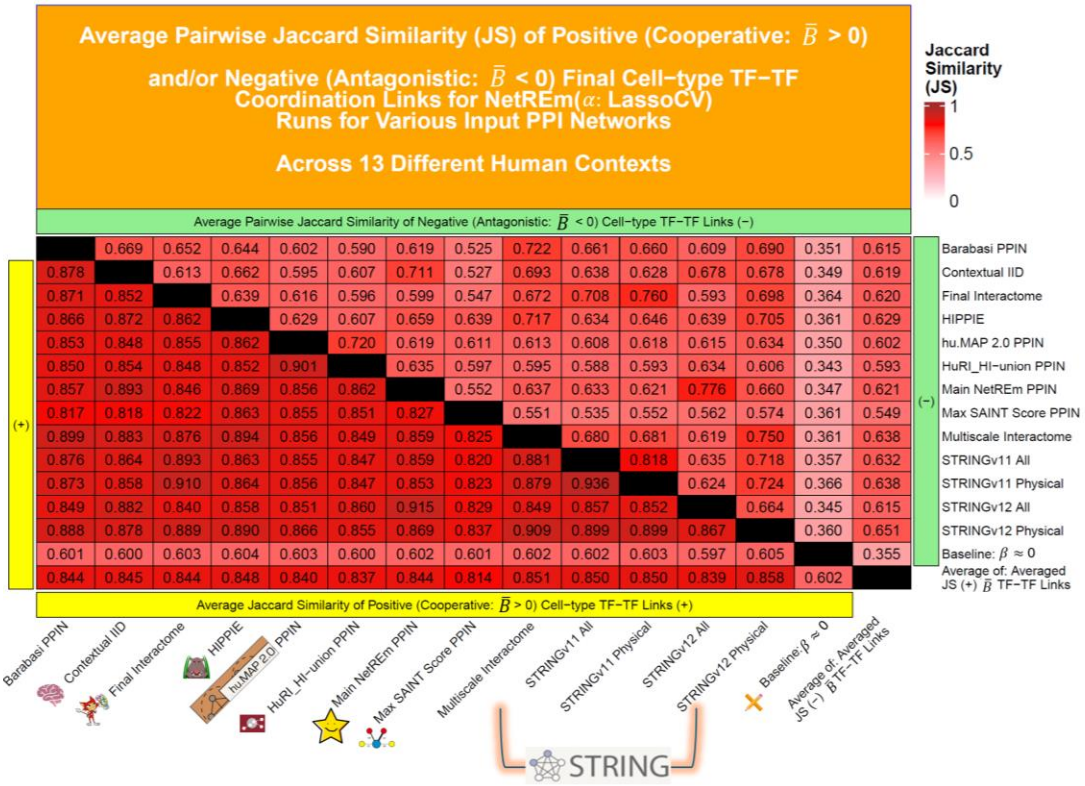

Average Pairwise Metric across 13 human contexts	Aggregate measure Applied to Pairwise Metric	78 pairwise comparisons (amongst 13 NetREm models)	13 pairwise comparisons of NetREm models with baseREm
JS of Cooperative Cell-type TF-TF Coordination Links	min	0.816641	0.596677
	max	0.935608	0.605095
	average	0.8634	0.601635
	median	0.859443	0.601685
JS of Antagonistic Cell-type TF-TF Coordination Links	min	0.525491	0.342509
	max	0.81817	0.366497
	average	0.638788	0.355182
	median	0.633855	0.357343

In this analysis, we compare how similar the final overall cell-type TF-TF coordination networks $-100 \leq \bar{B} \leq 100$ are in terms of assigning TF-TF links as cooperative ($\bar{B} > 0$) or antagonistic ($\bar{B} < 0$). To do this, we separate the analysis, where we focus on the similarity of the positive TF-TF coordination links on the bottom left and the similarity of the negative TF-TF coordination links on the top right. For each pair of models, we compute the Jaccard Similarity (JS) scores. Here, the bottom left matrix represents the average Jaccard Similarity (JS) scores of each of the 13 different models (13 $\text{NetREm}_{\beta=1}$ models and $baseREm_{\beta\approx0}$ model) across the 13 human contexts for the TF-TF coordination links that are predicted positive. Here, for each pair of these models, we compute these JS scores for the positive links across all 13 contexts and then average the JS score and report that value. We do this for all 78 of the pairwise model comparisons here. The top right is derived in the same fashion, except the focus is on the similarity among the TF-TF coordination links that are predicted negative by the respective pair of models. On average (based on averaging the 78 average pairwise Jaccard Similarity scores for the 13 human contexts), we find that 13 $\text{NetREm}_{\beta=1}$ models have a: Jaccard Similarity (JS) score for the negative TF-TF coordination links of 0.638788 with each other and a JS score of 0.8634 for the positive TF-TF coordination links. These average values are much higher than those average values of $\text{NetREm}_{\beta=1}$ models with baseREm: 0.355182 for negative TF-TF coordination links and 0.601635 for positive TF-TF coordination links. Nonetheless, we do recognize that despite the similar assignments of cooperative or antagonistic links for each pair of models, the magnitudes assigned to a given TF-TF link may vary. That is, we need to use additional metrics to quantify the similarity of the TF-TF coordination score magnitudes and values, which we do in **Fig. S13G**, where we compare the average Spearman correlations and Cosine Similarity values of the resulting TF-TF coordination networks across the 13 contexts for each pair of models.

G)

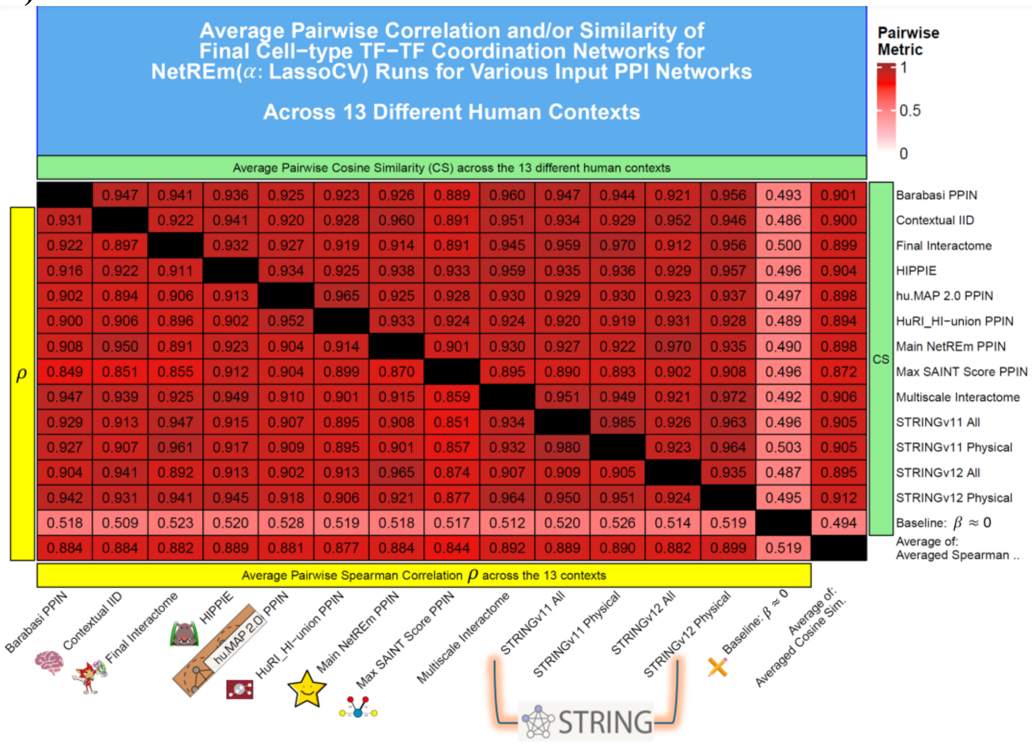

Average Pairwise Metric across 13 human contexts	Aggregate measure Applied to Pairwise Metric	78 pairwise comparisons (amongst 13 NetREm models)	13 pairwise comparisons of NetREm models with baseREm
Spearman Correlation of Cell-type TF-TF	min	0.849387	0.508715
	max	0.980214	0.528357
	average	0.913097	0.518692
	median	0.91156	0.518639
Cosine Similarity of Cell-type TF-TF	min	0.888851	0.486066
	max	0.984515	0.503156
	average	0.932854	0.493774
	median	0.930167	0.495408

Next, we aggregate the measures for the average Spearman correlations for the 78 comparisons among NetREm$_{\beta=1}$ models and find the following metrics: (min: 0.849387, max: 0.980214, average: 0.913097, median: 0.91156). We can compare this with the resulting aggregate measures for the average Spearman correlations for the 13 comparisons between NetREm$_{\beta=1}$ and baseREm models (min: 0.508715, max: 0.528357, average: 0.518692, median: 0.518639). Similarly, we aggregate the measures for the average Cosine Similarity (CS) values for the 78 comparisons among NetREm$_{\beta=1}$ models and find the following metrics: (min: 0.888851, max: 0.984515, average: 0.932854, median: 0.930167). We can compare this with the resulting aggregate measures for the average CS values for the 13 comparisons between NetREm$_{\beta=1}$ and baseREm models (min: 0.486066, max: 0.503156, average: 0.493774, median: 0.495408).

Supplementary Figure 14 – Evaluating the Robustness of NetREm's TF-TG Regulatory Networks and TF-TF Coordination Networks across 13 Human Contexts for Different Input PPI Networks (PPINs)

A) These 13 different input PPINs have various degrees of pairwise cosine similarity and Spearman correlation (ρ) with each other. Despite these variations, NetREm predictions generally remain relatively stable and are not highly sensitive to differences in the input PPIN. In **Fig. S14A-G** we evaluate the robustness of NetREm in terms of the input PPI network for the 13 different input PPINs and the baseREm case. This results in 91 pairwise comparisons (78 among the 13 $\text{NetREm}_{\beta=1}$ models, 13 between baseREm and each $\text{NetREm}_{\beta=1}$ model) of inputs and outputs across each of the 13 contexts. After running NetREm for these 13 NetREm-based models baseREm, we obtain TF-TG regulatory networks (**Fig. S14B-D**) and TF-TF coordination networks (**Fig. S14E-G**) for each of these contexts. As anticipated, models with more similar input PPINs (higher ρ) tend to produce similar outputs, displaying higher Jaccard Similarity (JS) in predicted signed TF-TG regulatory links and higher ρ in the TF-TF coordination networks. That is, across the 13 contexts, we find that input PPINs that are more similar (e.g. have higher ρ with each other) tend to have higher: Jaccard Similarity (JS) scores of overlaps in their predicted TF-sign-TG regulatory links (**Fig. S14B**) and positive ρ values of their predicted cell-type TF-TF coordination networks (**Fig. S14E**). Nonetheless, in general, the 13 $\text{NetREm}_{\beta=1}$ models have statistically significant overlaps in signed TF-TG links (hypergeometric p-adj < 0.05) with each other for at least 12 contexts (for all but 1 comparison) and with baseREm for only 9-10 contexts (**Fig. S14C**); this highlights how NetREm's signed TF-TG regulatory networks are typically robust to changes in the input PPIN, especially if there is an input PPIN.

Key takeaways: Across all 78 pairwise comparisons among the 13 $\text{NetREm}_{\beta=1}$ models, 91.03% (71 of 78) show statistically significant overlaps in signed TF-TG links (hypergeometric p-adj < 0.05) for all 13 contexts (**Fig. S14C**). The remaining 7 comparisons exhibit significant overlaps in ≥11contexts. Our comparative analyses show $\text{NetREm}_{\beta=1}$ models predict highly similar TF-TF coordination networks, with high mean ρ (0.85 to 0.98) values across all contexts, regardless of the degree of similarity in their respective input PPINs (**Fig. S14G**). In contrast, the 13 comparisons between $\text{NetREm}_{\beta=1}$ and baseREm models demonstrate fewer significant overlaps in signed TF-TG links (only in 9-10 contexts) and more divergent TF-TF coordination networks, with notably lower ranges of mean ρ (0.51-0.53) values.

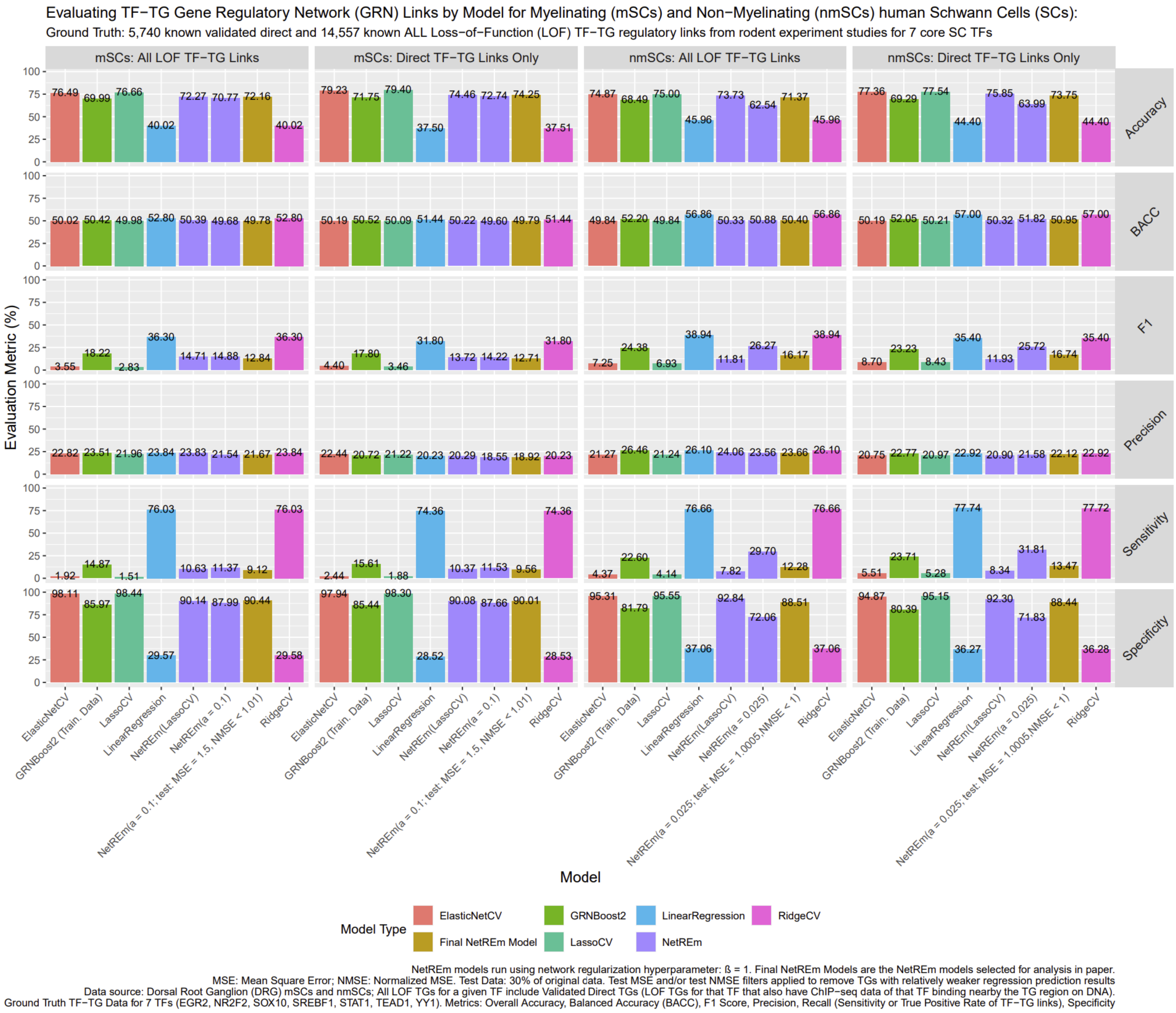

Supplementary Figure 15 – NetREm TF-TG regulatory link performance metrics for 7 core Schwann cell (SC) Transcription Factors (TFs)

NetREm TF-TG regulatory link performance metrics for 7 core Schwann cell (SC) Transcription Factors (TFs) in myelinating (mSCs) and non-myelinating (nmSCs) human SCs in the Dorsal Root Ganglion (DRG). This analysis is done for only the 7 TFs that we have background, experimental validation data for: EGR2, NR2F2, SOX10, SREBF1, STAT1, TEAD1, YY1. Here, RXRG is not included since we lack loss-of-function (LOF) validation data for this TF. For the LOF evaluation, there are 64,765 total possible LOF TF-TG regulatory links in SCs. We note that there are 14,557 are total positive links that appear in the ground truth GRN.

A)

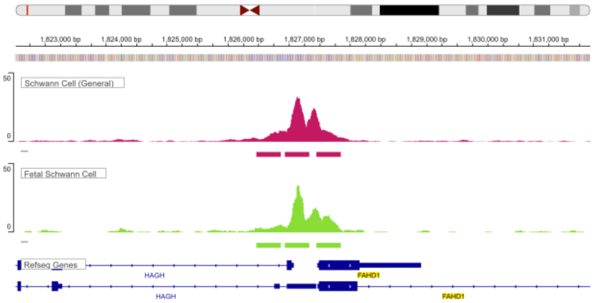

predicted SOX10 binding regions for *FAHD1*

TF	TG	chromosome	start	end	region	genome
SOX10	FAHD1	chr16	1826388	1827564	promoter	hg38

B)

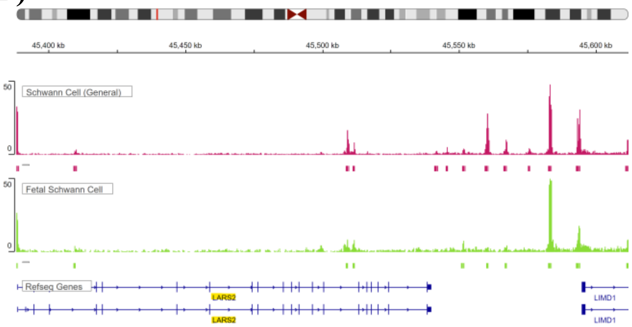

predicted SOX10 binding regions for *LARS2*

TF	TG	chromosome	start	end	region	genome
SOX10	LARS2	chr3	45388244	45388873	Distal (Enhancer)	hg38
SOX10	LARS2	chr3	45511534	45511853	Distal (Enhancer)	hg38
SOX10	LARS2	chr3	45582929	45583865	Distal (Enhancer)	hg38
SOX10	LARS2	chr3	45611555	45611991	Distal (Enhancer)	hg38

C)

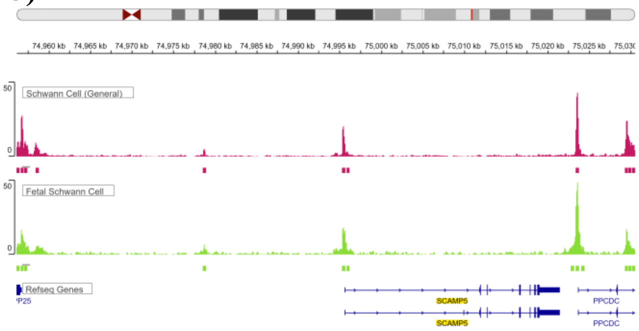

predicted SOX10 binding regions for *SCAMP5*

TF	TG	chromosome	start	end	region	genome
SOX10	SCAMP5	chr15	74956149	74957552	Distal (Enhancer)	hg38
SOX10	SCAMP5	chr15	75029296	75030505	Distal (Enhancer)	hg38

D)

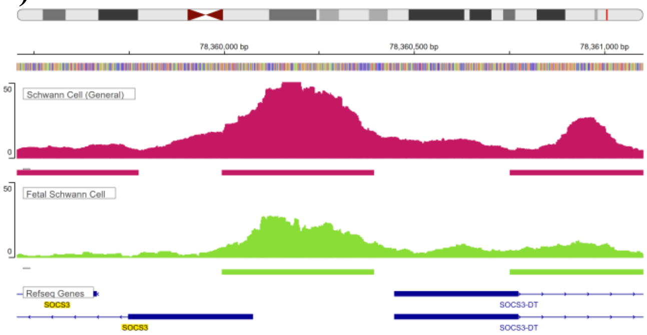

predicted SOX10 binding regions for *SOCS3*

TF	TG	chromosome	start	end	region	genome
SOX10	SOCS3	chr17	78359457	78361101	promoter	hg38

Supplementary Figure 16 – Open Chromatin in human SCs and SOX10 predicted binding regions for 4novel SOX10-predicted TGs in human mSCs

Binding peaks of open chromatin in humans (hg38 reference genome) based on (Zhang et al. 2021) for the corresponding 4 novel final TGs for SOX10 in SCs shown in **Figure 3D** (rat validation data). None of these TGs are LOF or direct TGs for SOX10. Left: epigenome tracks of accessible chromatin in humans. Top track (scarlet): adult general SCs, bottom track (green): fetal SCs. On the right is a table of predicted SOX10 binding regions for each of these TGs in hg38 in the input prior GRNs in adult mSCs derived from multiomics data (initial TF-RE-TG links). **A)** SOX10 may bind to a promoter region for *FAHD1*. **B)** SOX10 may bind to various potential enhancers for *LARS2*. **C)** SOX10 binds to various potential enhancer regions for *SCAMP5*. **D)** SOX10 may bind to a potential promoter for *SOCS3*.

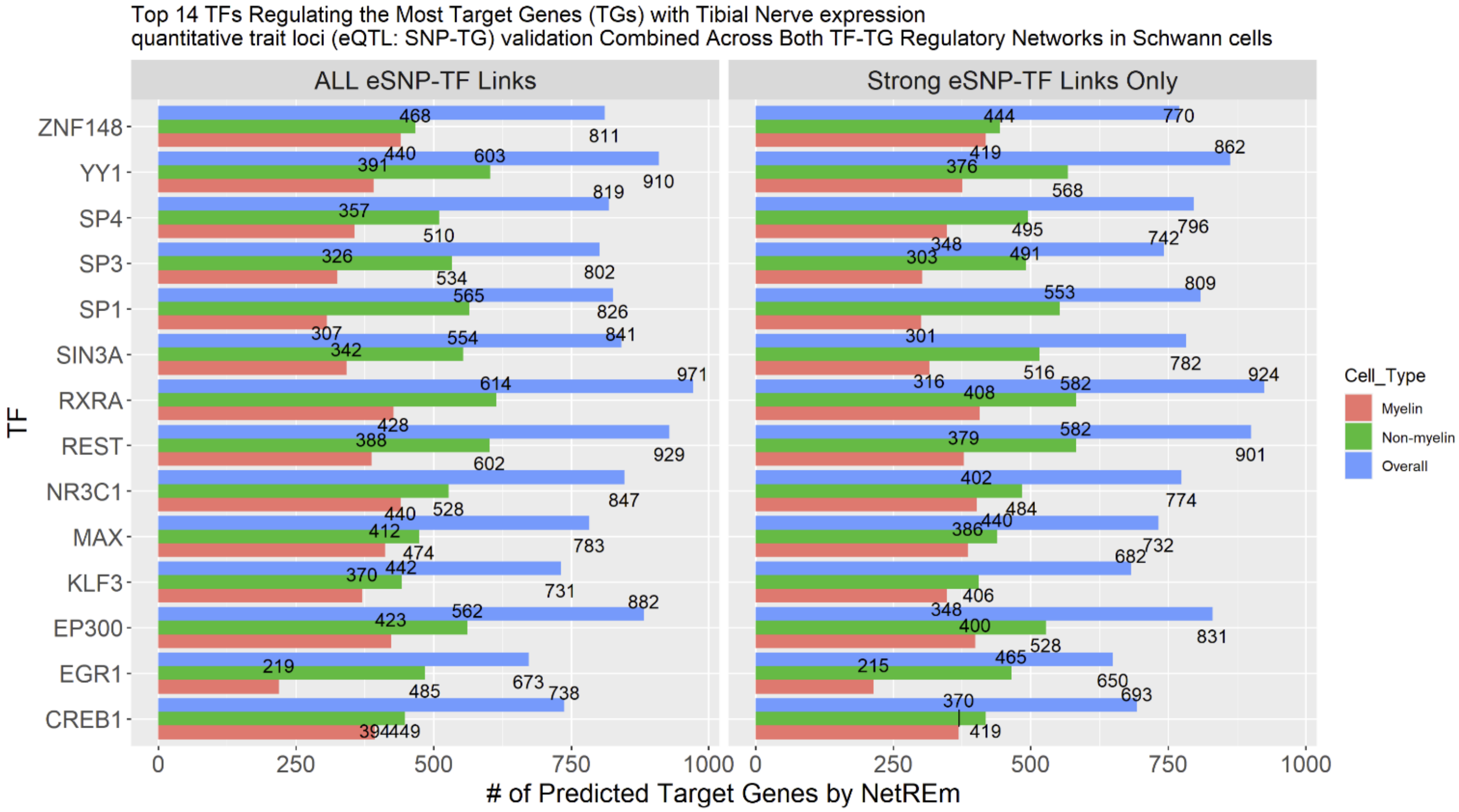

Supplementary Figure 17 – Comparative barplot showing the top 14 Transcription Factors (TFs) with the highest # of eQTL-validated Target Genes (TGs) overall across myelinating (mSCs) and non-myelinating (nmSCs) Schwann Cells (SCs).

This plot focuses on the top 14 TFs that altogether regulate the greatest number of unique TGs that have eQTL validation in Schwann Cells (SCs). To this end, we pool together the results from myelin (mSCs) and non-myelin (nmSCs). This comparative barplot shows the 14 TFs with the highest number of eQTL-validated TGs in both mSC and nmSC NetREm-predicted TF-TG regulatory networks (complementary GRNs). The panel on the left focuses on the TFs with this validation based on strong and/or weak predicted SNP-TF effects predicted by the motifbreakR (Coetzee et al. 2015) tool; the panel on the right focuses on the TFs with this validation based on only the strong predicted SNP-TF effects.

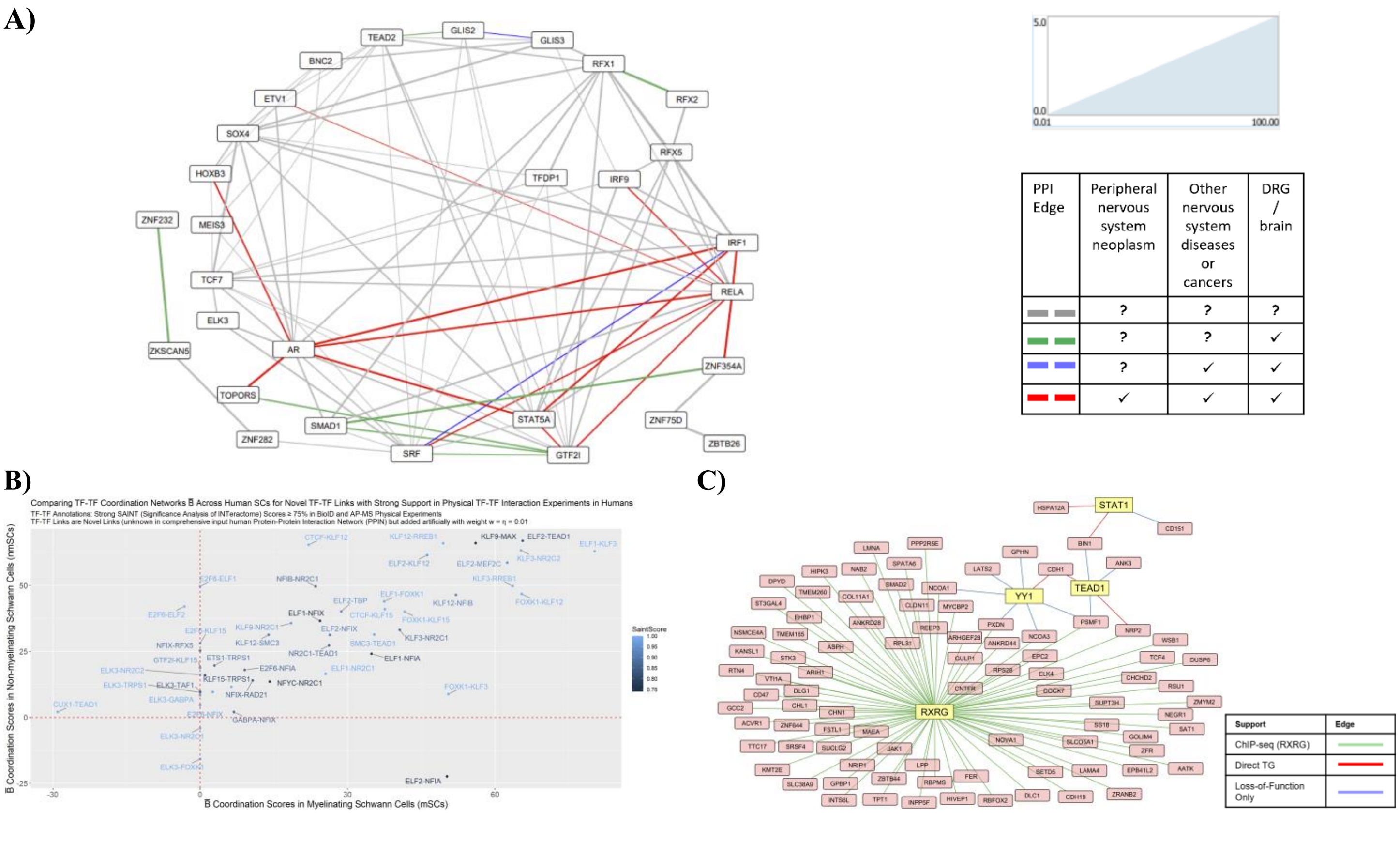

Supplementary Figure 18 – TF-TF Interactions and TF-TG Regulatory Networks in human nmSCs

A) TF-TF Interaction Network of 29 of 31 nmSC-specific TFs, with edge weights representing $\bar{B}$ coordination scores across the 5,164 TGs in the final nmSC NetREm TF-TG Regulatory Network. There are 93 TF-TF links. These links shown are subset to those that are known in input PPIN (i.e. those with evidence of known TF-TF interactions in the input PPI network). Higher cell-type coordination scores ($\bar{B}$) indicate stronger predicted TF-TF interactions. We annotate TF-TF interaction edges based on contextual Integrated Interactions Database (IID). Scores are predicted for 8,943 mSC TGs, all 5,207 nmSC TGs.

B) Comparative scatterplot of 48 novel TF-TF coordination links (unknown in comprehensive input human PPIN but added artificially to make input PPIN fully-connected, with $w = \eta = 0.01$) that are found in mSCs (37) and/or nmSCs (all 48). These novel TF_i-TF_j links comprise 30 different TFs discovered in (Göös et al. 2022) recent study on direct, physical human TF interaction networks where these TFs are predicted to physically bind in AP-MS and/or BioID experiments with very strong SAINT scores of interactions. Other novel TF-TF links may correspond to indirect TF-TF interactions that are not yet known (since we used the most comprehensive version). Overall, this shows NetREm's novel TF-TF coordination links may be biologically meaningful discoveries and also shows differences between the cell-type TF-TF coordination scores $\bar{B}$ between human mSCs and nmSCs.

C) Common TGs regulated by 4 Core Schwann Cell (SC) TFs (STAT1, YY1, RXRG, TEAD1) in nmSCs. All 4 TFs co-regulate 174 TGs in nmSCs. Here, this network visualization is filtered to only show the edges that have experimental validation support. We only have ChIP-seq data for RXRG and do not have knowledge of validated Direct TGs or LOF TGs. That is, RXRG only has ChIP-seq data (and no loss-of-function (LOF) information) available. So green edges correspond to TGs with RXRG ChIP-seq support. For instance, *CDH19* is 1 of the common TGs co-regulated by all 4 TFs and it is also a TG with nearby RXRG ChIP-seq peaks. Edges in blue (LOF only TGs) and/or red (direct TGs: LOF and ChIP-seq peaks for support) therefore correspond therefore to STAT1, TEAD1, and/or YY1 TGs.

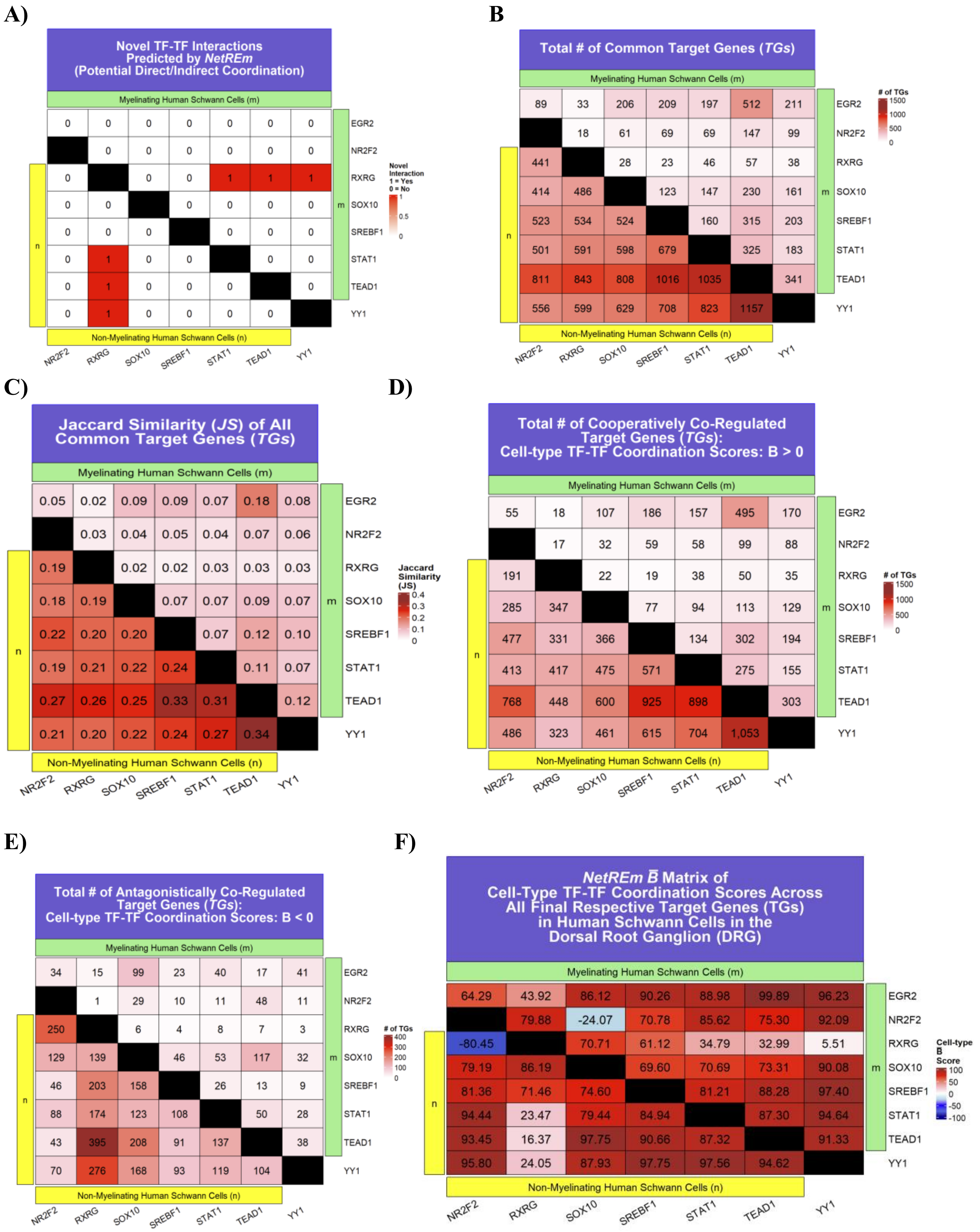
A)
Novel TF-TF Interactions Predicted by NetREm (Potential Direct/Indirect Coordination)
Myelinating Human Schwann Cells (m)
Non-Myelinating Human Schwann Cells (n)
Novel Interaction 1 = Yes 0 = No
B
Total # of Common Target Genes (TGs)
# of TGs
C)
Jaccard Similarity (JS) of All Common Target Genes (TGs)
Jaccard Similarity (JS)
D)
Total # of Cooperatively Co-Regulated Target Genes (TGs): Cell-type TF-TF Coordination Scores: B > 0
E)
Total # of Antagonistically Co-Regulated Target Genes (TGs): Cell-type TF-TF Coordination Scores: B < 0
F)
NetREm B̄ Matrix of Cell-Type TF-TF Coordination Scores Across All Final Respective Target Genes (TGs) in Human Schwann Cells in the Dorsal Root Ganglion (DRG)
Cell-type B Score
EGR2
NR2F2
RXRG
SOX10
SREBF1
STAT1
TEAD1
YY1

G)

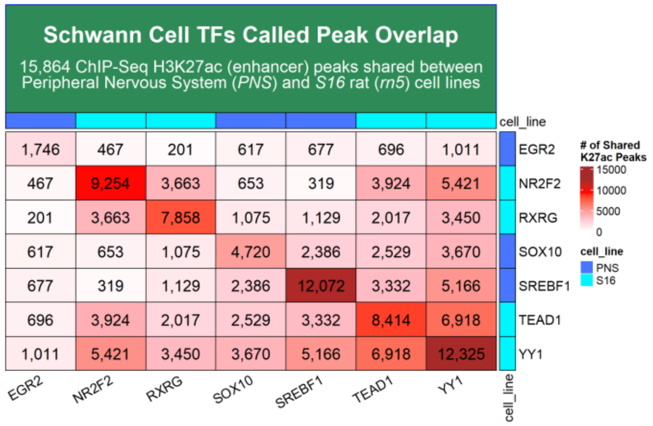

	EGR2	NR2F2	RXRG	SOX10	SREBF1	TEAD1	YY1
EGR2	1,746	467	201	617	677	696	1,011
NR2F2	467	9,254	3,663	653	319	3,924	5,421
RXRG	201	3,663	7,858	1,075	1,129	2,017	3,450
SOX10	617	653	1,075	4,720	2,386	2,529	3,670
SREBF1	677	319	1,129	2,386	12,072	3,332	5,166
TEAD1	696	3,924	2,017	2,529	3,332	8,414	6,918
YY1	1,011	5,421	3,450	3,670	5,166	6,918	12,325

H)

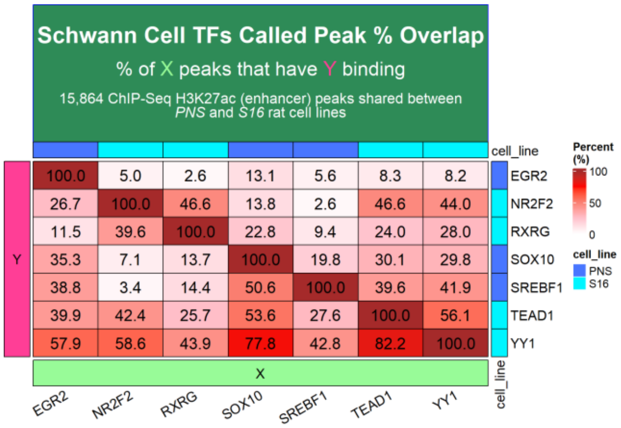

Y \ X	EGR2	NR2F2	RXRG	SOX10	SREBF1	TEAD1	YY1
EGR2	100.0	5.0	2.6	13.1	5.6	8.3	8.2
NR2F2	26.7	100.0	46.6	13.8	2.6	46.6	44.0
RXRG	11.5	39.6	100.0	22.8	9.4	24.0	28.0
SOX10	35.3	7.1	13.7	100.0	19.8	30.1	29.8
SREBF1	38.8	3.4	14.4	50.6	100.0	39.6	41.9
TEAD1	39.9	42.4	25.7	53.6	27.6	100.0	56.1
YY1	57.9	58.6	43.9	77.8	42.8	82.2	100.0

Supplementary Figure 19 – Analyzing NetREm TF-TF predictions and validations for 8 core TFs in SCs

We have experimental data for TFs: EGR2, NR2F2, RXRG, SOX10, SREBF1, STAT1, TEAD1, YY1. For some TFs we have more experimental data than for others. EGR2 is not in nmSCs. **Figures S19A-F** do not have EGR2 for columns; top diagonal (right) refers to mSCs, bottom diagonal (low left) refers to nmSCs. We use rat data in SC lines Peripheral Nervous System (PNS) and/or S16 to help validate our findings (**Figures S19G-H**).

A) NetREm predicts 3 novel TF-TF interactions (not known in input PPIN): RXRG-STAT1, RXRG-TEAD1, RXRG-YY1.

B) predicted # of co-regulated (common) TGs for the pair of TFs. Coordinating TFs tend to share a significantly larger # of TGs than expected by chance(Wang et al. 2009). **C)** Jaccard Similarity (JS) of shared TGs: # of shared TGs divided by total # of TGs predicted across both TFs. **D)** # of TGs that are predicted to be cooperatively co-regulated ($\bar{B} > 0$) by the pair of TFs (i.e. set of TGs they have similar signs of coefficients c^* for). That is, for each $TF_i - TF_j$ pair we count # of TGs where $c_i^* c_j^* > 0$.

E) # of TGs predicted to be antagonistically co-regulated ($\bar{B} < 0$) by the given pair of TFs (i.e. set of TGs they have opposite signs of c^* for). This is the # of co-regulated TGs where $c_i^* c_j^* < 0$.

F) Predicted Cell-type TF-TF coordination scores for core SC TFs based on coordination scores for 8,943 TGs in mSCs, 5,207 TGs in nmSCs. TFs with non-zero scores for a TG are predicted as final model TFs for TG.

G) SC TFs called peak overlap based on 15,864 ChIP-seq enhancer peaks (H3K27ac) shared between PNS and S16 rat cell lines. This is the only symmetric heatmap in **Fig. S19**.

H) Based on **Fig. S19G** but presents binding %. Column names refer to X axis, row names refer to Y axis. The plot is interpreted by going to X axis and selecting a TF. Then, we go up the row to the TF of interest, our Y. So, the 1st column (X = 1: EGR2), 2nd row from the top (Y = 2: NR2F2) refers to the % of shared SC peaks for TF EGR2 that have also have strong binding for TF NR2F2: ~26.7%. Conversely, the 2nd column (X = 2: NR2F2), 1st row from the top (Y = 1: EGR2) is 5%, which means that: 5% of peaks for NR2F2 have strong binding for EGR2 as well.

A)

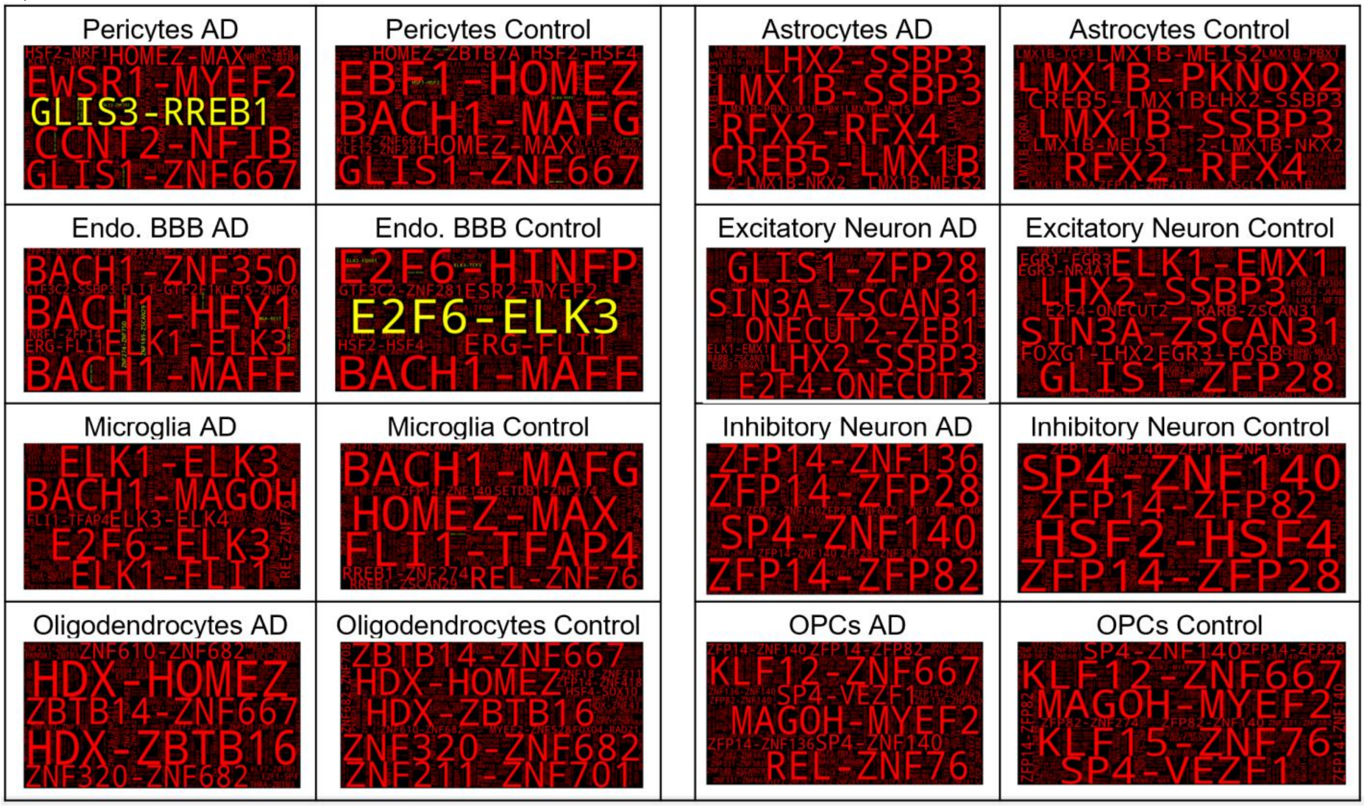

Antagonistic Cell-type Coordination: $\bar{B} < 0$ Cooperative Cell-type Coordination: $\bar{B} > 0$

B)

Comparing TF-TF Coordination Networks $\bar{B}$ Across 8 Glial/Neuronal Cells in Control versus Alzheimer's (AD) for 216 Novel TF-TF Links with Strong Support in Physical TF-TF Interaction Experiments in Humans

- TF-TF Annotations: Strong SAINT (Significance Analysis of INTeractome) Scores ≥ 75% in BioID and AP-MS Experiments
- TF-TF Links are Novel Links (unknown in comprehensive input human PPI Network (PPIN) but added artificially with $w = \eta = 0.01$)

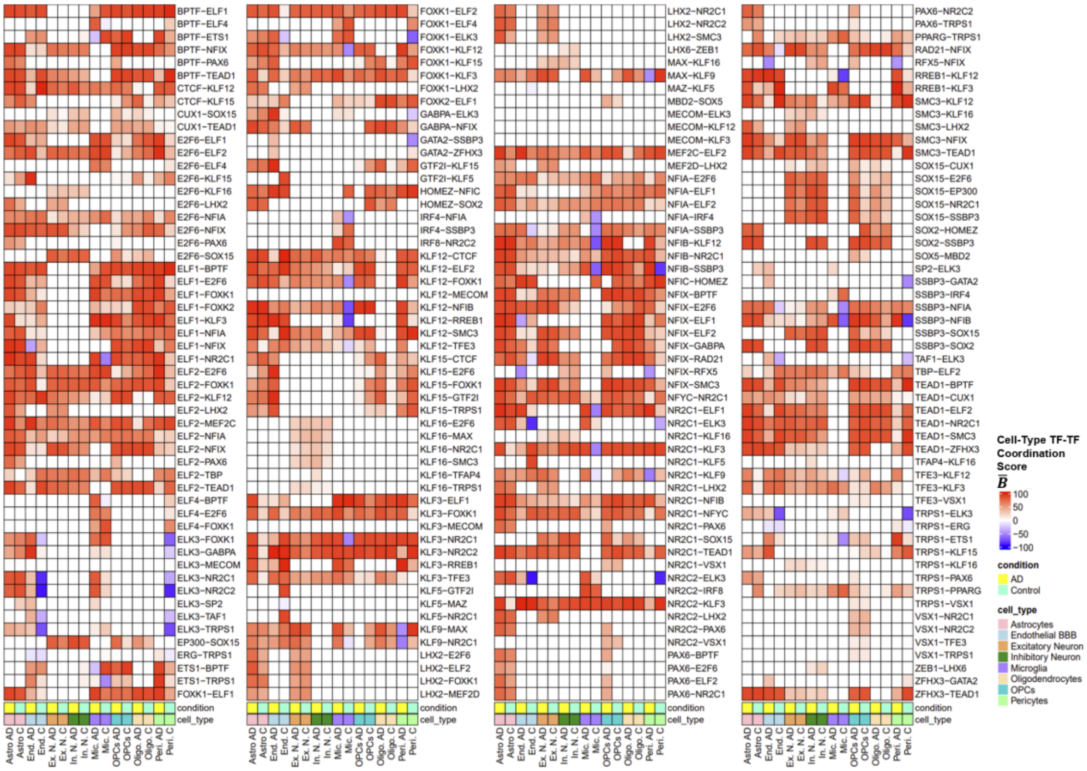

Supplementary Figure 20 – Comparing Coordination Scores Across Glial/Neuronal Cell-Types and Conditions: Information Content Word Cloud representations of Cell-type TF-TF Coordination Network Links $-100 \leq \bar{B} \leq 100$ and Comparison of Novel TF-TF Links with Physical Binding Support

A) We note there are larger word sizes for larger $|\bar{B}|$. Here, red words have: $\bar{B} > 0$ and yellow words have $\bar{B} < 0$. We show the core links here (i.e. those with relatively higher magnitudes of cell-type TF-TF coordination). Endo BBB is for Endothelial cells in the Blood Brain Barrier. OPCs represents Oligodendrocyte Progenitor (or Precursor) cells.
B) Comparative heatmap of 216 novel TF-TF coordination links (unknown in comprehensive input human PPIN but added artificially to make input PPIN fully-connected, with $w = \eta = 0.01$) that are found in at least 1 of the 16 different networks (8 cell-type networks, 2 conditions: Alzheimer's disease (AD) versus Controls). Here, these novel TF_i-TF_j links are discovered in (Göös et al. 2022) recent study on direct, physical human TF interaction networks where these TFs are predicted to physically bind in AP-MS and/or BioID experiments with very strong SAINT scores of interactions (above 75%). Please note that other novel TF-TF links could correspond to indirect TF-TF interactions that are not yet known (since we used the most comprehensive version). Overall, this shows NetREm's novel TF-TF coordination links may be biologically meaningful discoveries and also shows differences between the cell-type TF-TF coordination scores $\bar{B}$ between human cell-types and/or conditions.

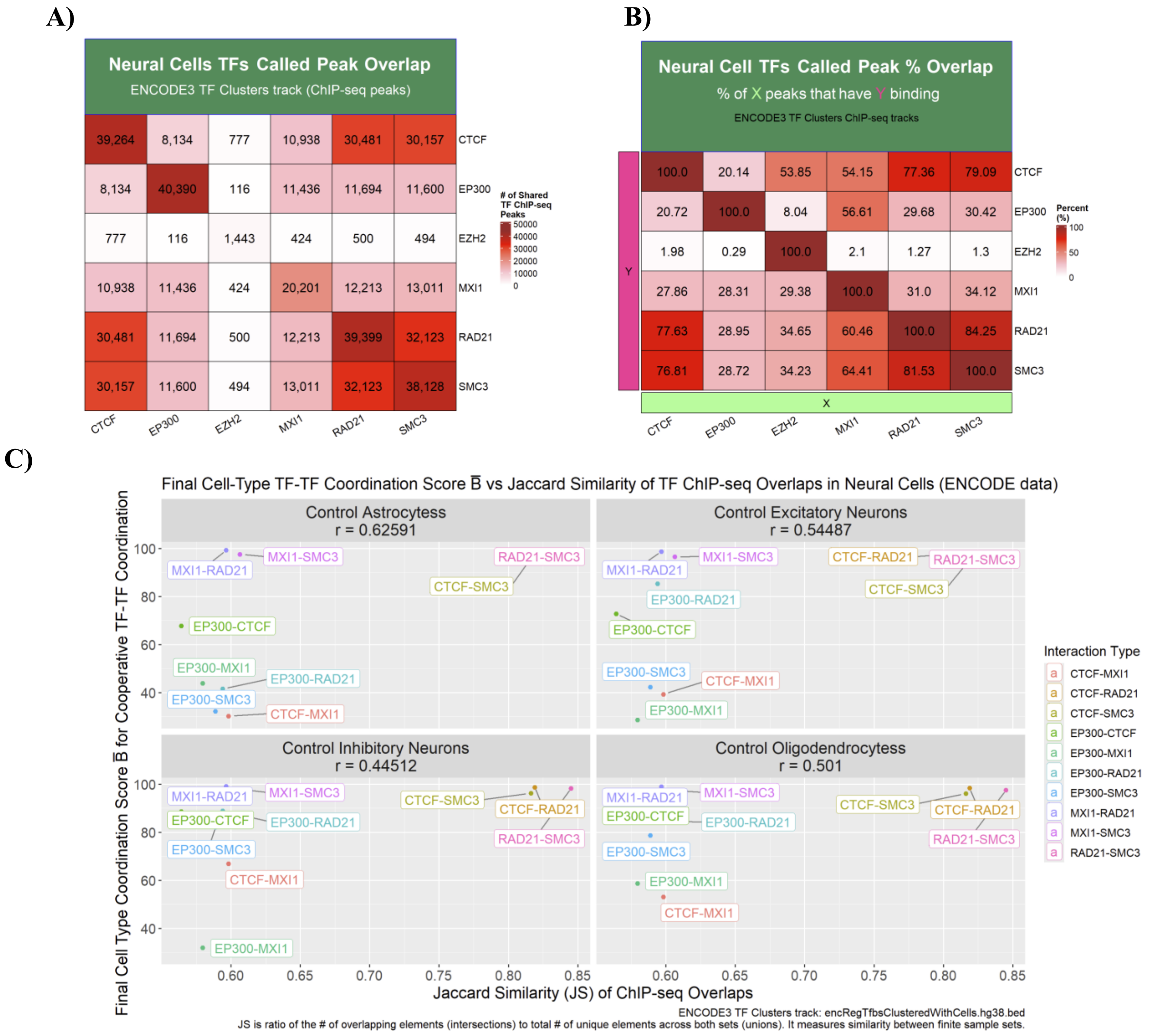

Supplementary Figure 21 – Analyzing NetREm TF-TF predictions and validations for Control Excitatory and Inhibitory Neurons based on Neural Cells for 5 TFs: CTCF, EP300, MXI1, RAD21, SMC3

We use raw data for the ENCODE3 TF clusters track (**encRegTfbsClusteredWithCells.hg38.bed.gz**) from UCSC Genome Browser. **A)** We see the peak overlap of TF Clusters (Based on ChIP-seq peaks) shared among these 6 TFs. This is a symmetric matrix. **B)** The column names refer to the X axis, and the row names refer to the Y axis. The plot is interpreted by going to the X axis and selecting a TF. Then, we go up the row to the TF of interest, which is our Y. For instance, (X = 3: EZH2, Y = 2: EP300) refers to % of shared neural cell TF clusters peaks for TF EZH2 that have also have strong binding for TF EP300, which is ~8.04%. Conversely, (X = 2: EP300, Y = 3: EZH2) shows ~0.29% of peaks for EP300 have strong binding for EZH2. We note that EZH2 is not found in our networks. **C)** We calculate the Jaccard Similarity (JS) of these overlaps among the TF-TF pairs for the 5 TFs. This JS(TF_i, TF_j) is based on: (# of common peaks for TF_i and TF_j) divided by the total # of unique peaks across TF_i and TF_j. So, TF pairs with a strong JS will have a high overlap and this gets 1 value for each TF-TF pair that quantifies similarity (i.e. a proxy for cooperativity metric: $\bar{B} > 0$ as both TFs are binding to overlapping regions, based on ChIP-seq peaks, versus aggressively competing for binding). This is on the x-axis. The y-axis has the cell-type TF-TF coordination score $\bar{B}$ and we note the correlations (r) across Control Astrocytes (r = 0.626), Excitatory Neurons (r = 0.544), Inhibitory Neurons (r = 0.45), Oligodendrocytes (r = 0.501). In fact, Astrocytes cells are derived from the same neural stem cells that give rise to neurons.

Supplementary Tables

Supplementary Table S1

Keeping the # of candidate Transcription Factors (TFs) constant (fixed) across all target genes (TGs): $N = \mathcal{N}$ for TGs that are not candidate TFs. For TGs that are also cell-type TFs, $N = \mathcal{N} - 1$ since we will remove that TF from the list of candidate TFs for that respective TG. For instance, in human embryonic stem cells (hESCs), we run NetREm 1,250 times (1 for each TG) with N = 206-207 TFs for each TG.

**Cell-type and conditions**	**# of TGs**	**# of candidate TFs $N = \mathcal{N}$** for $\mathcal{N}$ cell-type TFs<br>(If the TG is a TF, then $N = \mathcal{N} - 1$):<br>Maximum N = max(N))	**# of cells**<br>M<br>(if 100% of data is used for training, M = # of cells; else M = # of train cells)
Simulated Human Embryonic Stem Cells (hESCs)<br>Case 1: $M > N$	1,250	$\mathcal{N} = 207$ cell-type TFs<br>N = 206 to 207 cell-type TFs	(1,000; $M = 700$ train, 300 test)
Simulated Human Embryonic Stem Cells (hESCs)<br>Case 2: $M < N$	1,250	$\mathcal{N} = 207$ cell-type TFs<br>N = 206 to 207 cell-type TFs	(100; $M = 70$ train, 30 test)
Human Hematopoietic stem cells (HSCs)	10,588	$\mathcal{N} = 178$ cell-type TFs<br>N = 177 to 178 cell-type TFs	$M = 2{,}268$
Mouse Embryonic Stem cells (mESCs)	19,225	$\mathcal{N} = 195$ cell-type TFs<br>N = 194 to 195 cell-type TFs	$M = 1{,}080$
Mouse Dendritic cells (mDCs)	9,087	$\mathcal{N} = 93$ cell-type TFs<br>N = 92 to 93 cell-type TFs	$M = 1{,}211$
Human Pluripotent Stem Cells (hPSCs)	7,464	$\mathcal{N} = 170$ cell-type TFs<br>N = 169 to 170 cell-type TFs	$M = 5{,}520$
Budding Yeast Cells	5,355	$\mathcal{N} = 280$ cell-type TFs<br>N = 279 to 280 cell-type TFs	$M = 17{,}396$
PBMCs Naïve CD4-T cell (C0)	13,714	$\mathcal{N} = 1{,}029$ cell-type TFs<br>N = 1,028 to 1,029 cell-type TFs	$M = 709$
PBMCs CD14-Monocytes (C1)	13,714	$\mathcal{N} = 1{,}029$ cell-type TFs<br>N = 1,028 to 1,029 cell-type TFs	$M = 480$
PBMCs Memory CD4-T (C2)	13,714	$\mathcal{N} = 1{,}029$ cell-type TFs<br>N = 1,028 to 1,029 cell-type TFs	$M = 429$
PBMCs B cells (C3)	13,714	$\mathcal{N} = 1{,}029$ cell-type TFs<br>N = 1,028 to 1,029 cell-type TFs	$M = 342$
PBMCs CD8-T cell (C4)	13,714	$\mathcal{N} = 1{,}029$ cell-type TFs<br>N = 1,028 to 1,029 cell-type TFs	$M = 316$
PBMCs FCGR3A Monocyte (C5)	13,714	$\mathcal{N} = 1{,}029$ cell-type TFs<br>N = 1,028 to 1,029 cell-type TFs	$M = 162$
PBMCs Natural Killer cell (C6)	13,714	$\mathcal{N} = 1{,}029$ cell-type TFs<br>N = 1,028 to 1,029 cell-type TFs	$M = 154$
PBMCs Dendritic Cell (C7)	13,714	$\mathcal{N} = 1{,}029$ cell-type TFs<br>N = 1,028 to 1,029 cell-type TFs	$M = 32$
PBMCs Platelet (C8)	13,714	$\mathcal{N} = 1{,}029$ cell-type TFs<br>N = 1,028 to 1,029 cell-type TFs	$M = 14$

62 different immune cell sub-types	15	$\mathcal{N} = 125$ cell-type TFs<br>N = 124 to 125 cell-type TFs	Various values ranging from $M = 3$ for myeloid dendritic cell to $M = 35{,}204$ for macrophages
Comparison with RTNduals and baseREm:<br>13 Human Contexts<br>Filtering for TFs found in this study: (Göös et al. 2022) and in the gene expression dataset			
Schwann Cells GTEx Data (Eraslan et. al 2022) Pooled from 5 tissues	14,144	$\mathcal{N} = 88$ cell-type TFs<br>N = 87 to 88 cell-type TFs	$M = 1{,}430$
Excitatory Neuron (All Data) (Lake et al. 2018)	15,693	$\mathcal{N} = 76$ cell-type TFs<br>N = 75 to 76 cell-type TFs	$M = 13{,}709$
Inhibitory Neuron (All Data) (Lake et al. 2018)	15,693	$\mathcal{N} = 76$ cell-type TFs<br>N = 75 to 76 cell-type TFs	$M = 6{,}045$
Microglia (All Data) (Lake et al. 2018)	15,693	$\mathcal{N} = 97$ cell-type TFs<br>N = 96 to 97 cell-type TFs	$M = 317$
Oligodendrocytes (All Data) (Lake et al. 2018)	15,693	$\mathcal{N} = 80$ cell-type TFs<br>N = 79 to 80 cell-type TFs	$M = 2{,}657$
Control Excitatory Neurons (Training Data) (Mathys et. al 2019)	17,926	$\mathcal{N} = 77$ cell-type TFs<br>N = 76 to 77 cell-type TFs	$M = 11{,}969$
Control Inhibitory Neurons (Training Data) (Mathys et. al 2019)	17,926	$\mathcal{N} = 78$ cell-type TFs<br>N = 77 to 78 cell-type TFs	$M = 3{,}378$
Control Microglia (Training Data) (Mathys et. al 2019)	17,926	$\mathcal{N} = 97$ cell-type TFs<br>N = 96 to 97 cell-type TFs	$M = 676$
Control Pericytes (Training Data) (Mathys et. al 2019)	17,926	$\mathcal{N} = 98$ cell-type TFs<br>N = 97 to 98 cell-type TFs	$M = 64$
Control Astrocytes (Training Data) (Mathys et. al 2019)	17,926	$\mathcal{N} = 93$ cell-type TFs<br>N = 92 to 93 cell-type TFs	$M = 1{,}093$
Control Endothelial Blood Brain Barrier (BBB) (Training Data) (Mathys et. al 2019)	17,926	$\mathcal{N} = 93$ cell-type TFs<br>N = 92 to 93 cell-type TFs	$M = 43$
Control Oligodendrocyte Precursor Cells (OPCs) (Training Data) (Mathys et. al 2019)	17,926	$\mathcal{N} = 90$ cell-type TFs<br>N = 89 to 90 cell-type TFs	$M = 936$
Control Oligodendrocytes (Training Data) (Mathys et. al 2019)	17,926	$\mathcal{N} = 82$ cell-type TFs<br>N = 81 to 82 cell-type TFs	$M = 6{,}440$

Supplementary Table S2

Using multi-omics data and prior gene regulatory network (GRN) knowledge to customize the N candidate TF predictors for each target gene (TG).

**Cell-type and conditions**	**# of TGs**	**# of candidate TFs N:** (aggregate metrics across TGs: Average $N = \bar{N}$, Median $N = med(N)$, Minimum $N = \min(N)$, Maximum $N = \max(N)$)	**# of cells** (70% training = M, 30% testing)
Astrocytes (AD and Control)	13,804	AD: $\{\bar{N} = 191.81; med(N) = 203$; $\min(N) = 47$; $\max(N) = 259\}$<br><br>Control: $\{\bar{N} = 193.12$; $med(N) = 205$; $\min(N) = 47$; $\max(N) = 259\}$	AD: (1,830, M = 1,281 train, 549 test)<br><br>Control: (1,562, M = 1,093 train, 469 test)
Endothelial Blood Brain Barrier (BBB) (AD and Control)	12,619	AD: $\{\bar{N} = 182.07; med(N) = 191$; $\min(N) = 46$; $\max(N) = 267\}$<br><br>Control: $\{\bar{N} = 166.22; med(N) = 173$; $\min(N) = 49$; $\max(N) = 243\}$	AD: (59, M = 41 train, 18 test)<br><br>Control: (62, M = 19 train, 43 test)
Excitatory Neuron (AD and Control)	14,018	AD: $\{\bar{N} = 169.04$; $med(N) = 184$; $\min(N) = 41$; $\max(N) = 219\}$<br><br>Control: $\{\bar{N} = 168.29; med(N) = 184$; $\min(N) = 39$; $\max(N) = 221\}$	AD: (17,878, M = 12,515 train, 5,363 test)<br><br>Control: (17,098, M = 11,969 train, 5,129 test)
Inhibitory Neuron (AD and Control)	13,700	AD: $\{\bar{N} = 145.32; med(N) = 155$; $\min(N) = 33$; $\max(N) = 205\}$<br><br>Control: $\{\bar{N} = 148.92; med(N) = 159$; $\min(N) = 34$; $\max(N) = 209\}$	AD: (4,371, M = 3,060 train, 1,311 test)<br><br>Control: (4,825, M = 3,378 train, 1,447 test)
Microglia (AD and Control)	12,490	AD: $\{\bar{N} = 179.99$; $med(N) = 191$; $\min(N) = 49$; $\max(N) = 251\}$<br><br>Control: $\{\bar{N} = 182.62; med(N) = 195$; $\min(N) = 52$; $\max(N) = 252\}$	AD: (955, M = 668 train, 287 test)<br><br>Control: (965, M = 676 train, 289 test)
Oligodendrocytes (AD and Control)	11,859	AD: $\{\bar{N} = 177.17; med(N) = 189$; $\min(N) = 54$; $\max(N) = 241\}$<br><br>Control: $\{\bar{N} = 173.95; med(N) = 185$; $\min(N) = 52$; $\max(N) = 234\}$	AD: (9,035, M = 6,324 train, 2,711 test)<br><br>Control: (9,200, M = 6,440 train, 2,760 test)
Oligodendrocyte Precursor Cells (OPCs) (AD and Control)	12,641	AD: $\{\bar{N} = 172.45; med(N) = 184$; $\min(N) = 50$; $\max(N) = 234\}$<br><br>Control: $\{\bar{N} = 176.25; med(N) = 188$; $\min(N) = 50$; $\max(N) = 237\}$	AD: (1,290, M = 903 train, 387 test)<br><br>Control: (1,337, M = 936 train, 401 test)
Pericytes (AD and Control)	14,206	AD: $\{\bar{N} = 210.55; med(N) = 230$; $\min(N) = 62$; $\max(N) = 278\}$<br><br>Control: $\{\bar{N} = 206.92$; $med(N) = 227$; $\min(N) = 55$; $\max(N) = 272\}$	AD: (76, M = 64 train, 27 test)<br><br>Control: (91, M = 53 train, 23 test)
Myelinating Schwann Cells (mSCs)		$\{\bar{N} = 128.49$; $med(N) = 140$; $\min(N) = 28$; $\max(N) = 197\}$	(319, M = 223 train, 93 test)

Non-myelinating Schwann Cells (nmSCs)		$\{\bar{N} = 136.85;\ med(N) = 150$; $\min(N) = 27$; $\max(N) = 201$ }	(2,468, M = 1,727 train, 741 test)

Supplementary Table S3

Availability of Data and Materials:

**Data Resource**	**Access Information:**
NetREm open-source software package	https://github.com/SaniyaKhullar/NetREm<br>*implemented in Python*
Mapping of cis-Candidate Regulatory Elements (cCREs) for hg38 human reference genome coordinates	http://catlas.org/catlas_downloads/humantissues/cCRE_hg38.tsv.gz.
Activity by Contact (ABC) scores for interacting cis-candidate REs (cCREs)	http://catlas.org/catlas_downloads/humantissues/ABC_scores/
Raw single-cell scATAC-seq peaks	http://catlas.org/catlas_downloads/humantissues/Peaks/RAW/
Single-cell DRG expression data	GSE169301
Rat SOX10 PNS binding tracks	GSE64703 (Lopez-Anido et. al 2015)
UCSC Genome Browser Session for SOX10 peaks	genome.ucsc.edu/s/saniyaKhullar/updated_rn5
EGR2 data	GSE35132
Raw depositions for H3K27ac ChIP-seq data; Cut&Run data (YY1, RXRG, TEAD1)	GSE247955
STAT1 data	GSE211338
Expression data for 4 mESC samples	GSE108222
Ground truth signed TF-TG hESC network	File 5 in (Sharov et al. 2022)
(Zhang et al. 2023) HSC expression data	zenodo.org/records/7879228 (Buenrostro_Hematopoiesis.tar.gz)
(Zhang et al. 2023) has gold standard GRNs for HSCs and mESCs	zenodo.org/records/7879228 (scMTNI_sourcedata.tar.gz)
mDCs gene expression data	zenodo.org/records/5909090: normalized GSE48968 (expression_data.zip)
hPSCs gene expression data	zenodo.org/records/5909090: normalized GSE107552 (expression_data.zip)
Budding yeast gene expression data	zenodo.org/records/5909090: normalized GSE125162 (expression_data.zip)
gold standards for mDCs, hPSCs, budding yeast cells	zenodo.org/records/5909090: gold standards (gold_standard_datasets.zip)
Preprocessed gene expression data for 8 cell-types in AD versus Control stages	http://tiny.cc/ad_control_netrem
comparative cell-type-GRNs for mDCs, hPSCs, and bYCs	zenodo.org/records/5909090: comparative cell-type-GRNs (normalized_inferred_networks.zip).

SERGIO tool	github.com/PayamDiba/SERGIO
Integrated Interactions Database (IID)	iid.ophid.utoronto.ca/<br>Based on the study ((Kotlyar et. al 2022)): tissue-specific PPI networks across species. Version: 2021-05 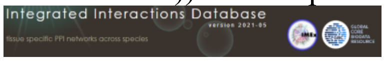
Older V11 STRINGdb PPIN	version-11-0.string-db.org/
Current PPIN includes V12 PPIs	string-db.org/
Tibial Nerve v8 eQTLs	gtexportal.org/home/downloads/adult-gtex/qtl
CNS eQTLs (Bryois et al. 2022)	zenodo.org/records/7276971
brainSCOPE resource	https://brainscope.gersteinlab.org/
scNET gene regulatory networks for AD and Control stages	https://zenodo.org/record/5829585
scGRNom tool	https://github.com/daifengwanglab/scGRNom/tree/master
Final Interactome PPIN	https://github.com/ku-cosbi/interactome-structural-coverage
Barabasi PPIN	https://github.com/Barabasi-Lab/COVID-19:<br><br>https://github.com/Barabasi-Lab/COVID-19/blob/main/data/DatasetS2.csv
Multiscale Interactome	https://github.com/snap-stanford/multiscale-interactome?tab=readme-ov-file ; http://snap.stanford.edu/multiscale-interactome/data/data.tar.gz
HIPPIE PPIN	https://cbdm-01.zdv.uni-mainz.de/~mschaefer/hippie/download.php v2.3 release (Human Integrated Protein-Protein Interaction rEference v2.3 release)
hu.MAP 2.0 PPIN	http://humap2.proteincomplexes.org/download: humap2_ppis_genename_20200821.txt
STRINGdb version 12 PPIN (most recent PPIN used by NetREm)	https://string-db.org/<br><br>organism id: humans (homo sapiens: 9606), mouse (mus musculus: 10090), yeast (Saccharomyces cerevisiae: 4932)<br><br>STRINGdbv12 all (indirect and/or direct PPIs) is obtained for each organism as follows: humans (**9606**.protein.links.v12.0.txt.gz), mouse (**10090**.protein.links.v12.0.txt.gz), yeast (**4932**.protein.links.v12.0.txt.gz)<br><br>Protein Annotations (mapping protein name to gene name):<br>**9606**.protein.info.v12.0.txt.gz<br>**10090**.protein.info.v12.0.txt.gz<br>**4932**.protein.info.v12.0.txt.gz
STRINGdb version 11 (older, archived version)	https://version-11-0.string-db.org/
Max SAINT Score PPIN	Supplementary Data obtained from (Göös et al. 2022)
Huri_HI-union PPIN	http://www.interactome-atlas.org/download: http://www.interactome-atlas.org/data/HI-union.tsv (64,006 interactions).
Lambert list of TFs	https://humantfs.ccbr.utoronto.ca/download/v_1.01/DatabaseExtract_v_1.01.csv<br>Current lists of human TFs and their motifs (v1.01) available from https://humantfs.ccbr.utoronto.ca/download.php

The Tabula Sapiens Atlas: Single-Cell Dataset	https://tabula-sapiens.sf.czbiohub.org/ → https://figshare.com/projects/Tabula_Sapiens/100973 → https://figshare.com/articles/dataset/Tabula_Sapiens_release_1_0/14267219
Paired yeast one-hybrid assays of antagonistic and cooperative TF-TF interactions in immune cell types (used for evaluating NetREm's TF-TF coordination links for specific TGs in the immune cell subtypes)	Supplementary Data (https://static-content.springer.com/esm/art%3A10.1038%2Fs41467-023-42445-6/MediaObjects/41467_2023_42445_MOESM5_ESM.xlsx).<br><br>Please use the data from Supplementary Tables 4 and 8 in the associated study to obtain the TG-specific TF-TF coordination links for evaluation. Their provided supplemental data is based on analysis for cytokine (immune-related) TGs.

Supplementary Table S4

Metrics for NetREm's TF-TG Gene Regulatory Networks (GRNs) and TF-TF Coordination Networks for Real-World Human Applications: Schwann cells and AD versus Controls

Cell-type	Condition	TF-TG Regulatory Network			TF-TF coordination network		
		# of TFs	***# of TGs***	***# of GRN links***	***# of TF-TF coordination links $\overline{B} \neq 0$***	***# of cooperative links: $\overline{B} > 0$***	***# of antagonistic links $\overline{B} < 0$***
Schwann Cells (SCs)	Myelinating (mSCs)	221	8,950	183,242	22,809	20,418	2,391
Schwann Cells (SCs)	Non-Myelinating (nmSCs)	228	5,207	277,541	24,795	21,852	2,943
Astrocytes	Alzheimer's disease (AD)	289	13,148	1,909,034	39,914	39,690	223
Endothelial Blood Brain Barrier (BBB) Cells		299	10,552	1,197,615	43,486	37,401	6,083
Excitatory Neuron (ExN)		240	8,758	1,286,725	27,891	27,890	0
Inhibitory Neuron (InN)		235	13,285	1,596,712	26,273	26,271	1
Microglia		276	12,033	1,165,661	36,810	34,770	2,039
Oligodendrocytes		264	8,510	1,215,594	33,510	33,509	0
Oligodendrocyte Precursor Cells (OPCs)		257	12,366	1,709,761	32,195	32,164	30
Pericytes		293	4,335	557,925	42,189	34,228	7,959
Astrocytes	Control	285	13,009	1,878,390	38,956	38,952	3
Endothelial BBB Cells		280	11,671	1,330,551	38,428	33,893	4,534
ExN		238	11,393	1,584,288	27,471	27,470	0
InN		238	13,368	1,512,851	26,699	26,698	0
Microglia		277	11,838	1,082,935	36,936	30,860	6,075
Oligodendrocytes		261	10,966	1,549,340	32,613	32,612	0
OPCs		263	12,201	1,744,456	33,707	33,515	191
Pericytes		288	11,419	1,599,600	40,576	35,246	5,328

NetREm with prior GRN knowledge input: initial TF-regulatory element-TG links → each TG has a customized list of $N \leq \mathcal{N}$ TFs. Please note our 2 real-world applications (# 11 and 12, respectively):

- 2 Schwann cell (SC) sub-types: myelinating (mSCs) vs. non-myelinating (nmSCs)
- Alzheimer's disease (AD) vs. Controls: 8 different neuronal/glial cell-types

Supplementary Table S5

The results of the 1-sided t-test comparison (assumption: equal variances = False) for raw coefficient values for the 5 Transcription Factor (TF) predictors (TF_1, TF_2, TF_3, TF_4, and TF_5) for the target gene (TG) y in the simulation study based on the following criteria: we run 1,000 simulations for $r(TF, TG)$ = [0.9, 0.5, 0.4, -0.3, -0.8] and 40% sparsity (0 values) for each variable in original training data, with Lasso model-type, $\alpha = 0.1$, and $\beta = 1$. In this case, we use all of the data for training (and the same exact data for testing) instead of holding anything out; thus, here our M = 10,000 cells. We standardize our data, so each variable has mean $\mu = 0$ and standard deviation of $\sigma = 1$. We retain the original simulated prior network from **Fig. 2A**. These results showcase NetREm's ability to statistically significantly increase the magnitudes of TF_2 (more positive) and TF_4 (more negative) and decrease the magnitude of TF_3 (making its value less positive), by incorporating the prior network as a constraint. This change in coefficient values for these 3 TFs does not appear to impact the coefficient values for major predictors TF_1 and TF_5. That is, in return, we retain our Null Hypothesis for TF_1 and TF_5 as the magnitude of their coefficients for TF_1 and TF_5 do not tend to decrease in any meaningful way.

1-Sided t Test Comparison: Coefficient Comparison for 1,000 Simulations

TF Predictor	Group 1	Group 2	Test statistic t	degrees of freedom (df)	p-value	Mean		Alternative Hypothesis H_A	Bounds	
						Group 1	Group 2		Low	Upper
TF_1	NetREm	LassoCV	-1,485.27	1,496.15	1	0.3772	0.6184	greater	-0.2414	Infinity
		Linear Regression	-1,484.88	1,495.73	1		0.6184		-0.2415	
		RidgeCV	-1,475.4	1,490.26	1		0.6181		-0.2411	
		ElasticNetCV	-1,483.75	1,498.90	1		0.6176		-0.2406	
TF_2		LassoCV	958.011	1,561.34	0	0.2475	0.0883		0.1589	
		Linear Regression	954.5762	1,561.05	0		0.0888		0.1584	
		RidgeCV	954.3345	1,561.25	0		0.0889		0.1583	
		ElasticNetCV	958.2369	1,562.95	0		0.0885		0.1587	
TF_3		LassoCV	-204.604	1,468.69	0	0.0316	0.0632	less	- Infinity	-0.0314
		Linear Regression	-208.372	1,468.36	0		0.0638			-0.0320
		RidgeCV	-208.61	1,467.92	0		0.0639			-0.0320
		ElasticNetCV	-205.703	1,469.94	0		0.0634			-0.0315
TF_4		LassoCV	-288.675	1,865.80	0	-0.0928	-0.0434			-0.0492
		Linear Regression	-284.77	1,865.46	0		-0.0440			-0.0485
		RidgeCV	-284.742	1,866.35	0		-0.0440			-0.0485
		ElasticNetCV	-288.374	1,867.02	0		-0.0435			-0.0491
TF_5		LassoCV	543.5864	1,716.19	1	-0.1884	-0.2874			0.0992
		Linear Regression	545.2875	1,715.89	1		-0.2877			0.0996

		RidgeCV	546.054	1,716.69	1		-0.2878			0.0997
		ElasticNetCV	545.9327	1,719.20	1		-0.2876			0.0995

Supplementary Table S6

Additional analysis of results for NetREm simulation study for conditions mentioned in **Table S1.** The results of the robustness evaluation for NetREm versus benchmark models trained on data for 5 Transcription Factor (TF) predictors (TF_1, TF_2, TF_3, TF_4, and TF_5) for the target gene (TG) with expression y in the simulation study. We use the following criteria: we run 1,000 simulations for $cor(TF, TG)$ = [0.9, 0.5, 0.4, -0.3, -0.8] and sparsity of 40% for gene expression data (i.e. ≈ 40% of the data for each of the 5 predictors in X and the response variable y are 0 values), with Lasso model-type and $\alpha = 0.1$ and $\beta = 1$. We simulate 10,000 cells. In each simulation, we simply vary the random seed used to generate the data (based on underlying correlations). Please note in this case we use all the data for training (as well as for testing) without holding any data out, so our $M = 10{,}000$ cells. We retain the original simulated prior network from **Fig. 2A**. Results illustrate how NetREm standard deviations (variance of models) tend to be lesser for each TF predictor relative to those from the other 4 benchmark models: ElasticNetCV, LassoCV, Linear Regression, and RidgeCV.

Standard Deviation of Coefficients in Demo Study after 1,000 simulations for $\beta = 1$					
Model	TF1	TF2	TF3	TF4	TF5
ElasticNetCV	0.004552	0.00459	0.004371	0.004308	0.004815
LassoCV	0.004567	0.004598	0.004378	0.004314	0.00483
LinearRegression	0.004569	0.004599	0.00438	0.004316	0.004831
NetREm	0.002353	0.002548	0.002184	0.003277	0.003137
RidgeCV	0.004598	0.004598	0.004382	0.004312	0.004827

Supplementary Table S7

The results for the NetREm simulation study when running 1,000 simulations for $cor(TF, TG)$ = [0.9, 0.5, 0.4, -0.3, -0.8] where the data is ≈40% sparse, M = 7,000 (since 70% of the 10,000 cells in the gene expression data are used for training). We used the Lasso model-type and $\alpha = 0.1$ and β varied across 13 values: 0.1, 0.25, 0.5, 0.75, 1, 2.5, 5, 7.5, 10, 25, 50, 75, and 100. The data has been standardized for X and y data based on the training data. In each simulation, the pairwise correlations among the TF predictors vary. We retain the original simulated prior network from **Fig. 2A**. Results illustrate how NetREm standard deviations (variance of the models) tend to be lesser up to $\beta = 5$. Then they teeter a bit till $\beta = 50$. After which they plummet. After this, the increased strength of network penalization tends to worsen the performance of NetREm, which struggles to meet the network constraint while optimizing model performance (measured by Mean Square Error (MSE)). This illustrates the bias-variance trade-off.

Standard Deviation Values Across 1,000 Simulations for Fixed α and Varied β						
β	TF_1	TF_2	TF_3	TF_4	TF_5	Total Standard Deviation
0.1	0.00330	0.00366	0.00371	0.00329	0.00381	0.00329
0.25	0.00275	0.00313	0.00326	0.00346	0.00335	0.00275
0.5	0.00248	0.00279	0.00279	0.00333	0.00317	0.00248
0.75	0.00239	0.00263	0.00245	0.00329	0.00314	0.00239
1	0.00235	0.00255	0.00218	0.00328	0.00314	0.00218
2.5	0.00231	0.00241	0.00136	0.00326	0.00318	0.00136
5	0.00233	0.00239	0.00091	0.00325	0.00320	0.00091
7.5	0.00236	0.00240	0.00076	0.00324	0.00320	0.00076
10	0.00241	0.00245	0.00070	0.00328	0.00325	0.00070
25	0.00254	0.00257	0.00062	0.00327	0.00326	0.00062

50	0.00090	0.00093	0.00017	0.00061	0.00063	0.00017
75	0.00059	0.00062	0	0	0	0
100	0.00051	0.00054	0	0	0	0

Supplementary Table S8

We vary $\beta \in \{0.5, 1, 5, 10\}$ and use LassoCV. As β increases, overall accuracy dips initially but improves to 76.7% at $\beta = 10$, with sensitivity peaking early before declining, and specificity exhibits an inverse pattern.

Metrics for Predicting TF-TG Regulatory Links for Human Hematopoietic Stem Cells (HSCs)

Goal:	?	↑	↑	↑	↓	↓	↓	↑	↑	↑	↑	↑	↑
Model	# of Rows (TF-TG)	True Positives	True Negatives	Total Correct (TP + TN)	False Negatives	False Positives	Total Errors (FN + FP)	Overall Accuracy	Sensitivity (recall)	Specificity	Balanced Accuracy (BACC)	Precision	F1 Score
NetREm (β = 1, α = 0.01)	1,133,617	181,624	629,438	811,062	121,432	951,993	1,073,425	43.04%	59.93%	39.80%	49.866%	16.02%	25.28%
NetREm (β = 0.5, LassoCV)	178,607	28,541	1,431,365	1,459,906	274,515	150,066	424,581	77.47%	9.42%	90.51%	49.964%	15.98%	11.85%
NetREm (β = 1, LassoCV)	187,230	30,379	1,424,580	1,454,959	272,677	156,851	429,528	77.21%	10.02%	90.08%	50.053%	16.23%	12.39%
NetREm (β = 5, LassoCV)	306,009	48,796	1,324,218	1,373,014	254,260	257,213	511,473	72.86%	16.10%	83.74%	49.918%	15.95%	16.02%
NetREm (β = 10, LassoCV)	178,607	21,288	1,424,112	1,445,400	281,768	157,319	439,087	76.70%	7.02%	90.05%	48.538%	11.92%	8.84%
GRNBoost2	741,189	133,696	973,938	1,107,634	169,360	607,493	776,853	58.78%	44.12%	61.59%	52.851%	18.04%	25.61%
ElasticNetCV	35,422	6,302	1,552,311	1,558,613	296,754	29,120	325,874	82.71%	2.08%	98.16%	50.119%	17.79%	3.72%
LassoCV	1,884,487	303,056	0	303,056	0	1,581,431	1,581,431	16.08%	100.00%	0.00%	50.000%	16.08%	27.71%
Linear Regression	1,884,487	303,056	0	303,056	0	1,581,431	1,581,431	16.08%	100.00%	0.00%	50.000%	16.08%	27.71%
RidgeCV	33,046	5,877	1,554,262	1,560,139	297,179	27,169	324,348	82.79%	1.94%	98.28%	50.111%	17.78%	3.50%
RTN (used by RTNduals)	152	16	1,581,295	1,581,311	303,040	136	303,176	83.91%	0.005%	1	49.998%	10.53%	0.0106%

Results where there are 1,884,487 total possible TF-TG regulatory links.
We note that RTNduals does not return any result for HSCs and instead returns an error stating: "no partial regulon has minimum ≥ 15".

Supplementary Table S9

Results where there are 3,748,681 total possible TF-TG regulatory links. NetREm's sensitivity is higher than that of ElasticNet and Lasso. NetREm's results are robust to changes in the input PPIN from old to new releases of PPI databases. To demonstrate this, we run NetREm(β = 1, α = 0.05) and compare results in mESCs for old STRINGdb version 11 (V11) versus updated V12 PPINs. Indeed, the mouse PPIN evolves from V11 to V12. For instance, 25 singleton TFs (of 195 mESC TFs) in V11 have known PPIs in V12. Nonetheless, our evaluation metrics results are similar.

Metrics for Predicting TF-TG Regulatory Links for Mouse Embryonic Stem Cells (mESCs)

Goal:	?	↑	↑	↑	↓	↓	↓	↑	↑	↑	↑	↑	↑
Model	# of Rows (TF-TG)	True Positives	True Negatives	Total Correct (TP + TN)	False Negatives	False Positives	Total Errors (FN + FP)	Overall Accuracy	Sensitivity (recall)	Specificity	Balanced Accuracy (BACC)	Precision	F1 Score
NetREm (β = 1, α = 0.05, old STRINGv11 PPI)	466,220	45,451	3,030,492	3,075,943	251,969	420,769	672,738	82.05%	15.28%	87.81%	51.5%	9.75%	11.9%

NetREm (β = 1, α = 0.05, new STRINGv12 PPI)	466,654	45,423	3,030,030	3,075,453	251,997	421,231	673,228	82.04%	15.27%	87.79%	51.5%	9.73%	11.9%
GRNBoost2	1,634,683	156,349	1,972,927	2,129,276	141,071	1,478,334	1,619,405	56.80%	52.57%	57.17%	54.9%	9.56%	16.2%
ElasticNetCV	232,211	23,021	3,242,071	3,265,092	274,399	209,190	483,589	87.10%	7.74%	93.94%	50.8%	9.91%	8.7%
LassoCV	213,819	21,275	3,258,717	3,279,992	276,145	192,544	468,689	87.50%	7.15%	94.42%	50.8%	9.95%	8.3%
Linear Regression	3,029,325	268,632	690,568	959,200	28,788	2,760,693	2,789,481	25.59%	90.32%	20.01%	55.2%	8.87%	16.1%
RidgeCV	2,982,912	266,479	734,828	1,001,307	30,941	2,716,433	2,747,374	26.71%	89.60%	21.29%	55.4%	8.93%	16.2%

Supplementary Table S10

In this comprehensive table for mDCs, we run NetREm with various hyperparameters and compare results for various runs against state-of-the-art cell-type GRNs and benchmark regression models (BRMs). Please note that **Fig. S6A** provides an accompanying visual of this Table.

We fix $\beta = 1$ and alter α for 11 values ranging from 0.01 to 0.2.

- We also vary β by magnitudes of 10 from 0.01 to 1,000 for 5 values and then optimize the selection of α using LassoCV.
- We fix $\alpha = 0.1$ and vary β for 4 different values: 0.1, 1, 10, and 100.

AUPR = Area Under Precision Recall Curve
AUC = Area Under Receiver Operator Characteristic Curve

**Metrics for Predicting TF-TG Regulatory Links for Normalized Mouse Dendritic Cells (mDCs)**													
Goal:	?	↑	↑	↓	↓	↑	↑	↑	↑	↑	↑	↑	↑
Model	# of Rows (TF-TG)	True Positives	True Negatives	False Negatives	False Positives	Overall Accuracy	Sensitivity (recall)	Specificity	Balanced Accuracy (BACC)	Precision	F1 Score	AUPR	AUC
NetREm (β = 0.01, LassoCV)	140,465	32,486	560,328	144,205	107,979	70.2%	18.4%	83.8%	51.1%	23.1%	20.5%	39.5%	9.9%
NetREm (β = 0.1, LassoCV)	140,484	32,513	560,336	144,178	107,971	70.2%	18.4%	83.8%	51.1%	23.1%	20.5%	39.5%	9.9%
NetREm (β = 1, LassoCV)	109,205	24,836	583,938	151,855	84,369	72.0%	14.1%	87.4%	50.7%	22.7%	17.4%	42.5%	7.6%
NetREm (β = 10, LassoCV)	59,230	13,846	622,923	162,845	45,384	75.4%	7.8%	93.2%	50.5%	23.4%	11.7%	49.6%	4.4%
NetREm (β = 100, LassoCV)	62,343	16,967	622,931	159,724	45,376	75.7%	9.6%	93.2%	51.4%	27.2%	14.2%	51.3%	5.5%
NetREm (β = 1000, LassoCV)	59,235	15,989	625,061	160,702	43,246	75.9%	9.0%	93.5%	51.3%	27.0%	13.6%	51.7%	5.2%
Scribe	427,042	170,986	412,251	5,705	256,056	69.0%	96.8%	61.7%	79.2%	40.0%	56.6%	47.4%	56.7%
Pearson	427,042	170,986	412,251	5,705	256,056	69.0%	96.8%	61.7%	79.2%	40.0%	56.6%	51.0%	39.8%
Pearson mean	314,028	89,366	443,645	87,325	224,662	63.1%	50.6%	66.4%	58.5%	28.5%	36.4%	34.4%	28.5%
PIDC mean	36,492	18,414	650,229	158,277	18,078	79.1%	10.4%	97.3%	53.9%	50.5%	17.3%	68.3%	5.1%
MERLIN	32,771	17,962	653,498	158,729	14,809	79.5%	10.2%	97.8%	54.0%	54.8%	17.2%	72.0%	5.5%
SCENIC	23,649	10,096	654,754	166,595	13,553	78.7%	5.7%	98.0%	51.8%	42.7%	10.1%	66.6%	3.1%
Inferelator	145,438	70,594	593,463	106,097	74,844	78.6%	40.0%	88.8%	64.4%	48.5%	43.8%	53.9%	19.0%
knnDREMI	258,009	66,184	476,482	110,507	191,825	64.2%	37.5%	71.3%	54.4%	25.7%	30.5%	31.4%	16.9%
LEAP mean	156,829	64,447	575,925	112,244	92,382	75.8%	36.5%	86.2%	61.3%	41.1%	38.6%	51.4%	21.6%
SCODE mean	156,829	64,447	575,925	112,244	92,382	75.8%	36.5%	86.2%	61.3%	41.1%	38.6%	46.7%	15.1%

SILGGM mean	155,399	38,604	551,512	138,087	116,795	69.8%	21.8%	82.5%	52.2%	24.8%	23.2%	39.2%	11.7%
ElasticNetCV	844,998	176,691	0	0	668,307	20.9%	100.0%	0.0%	50.0%	20.9%	34.6%	22.8%	52.9%
Linear Regression	844,998	176,691	0	0	668,307	20.9%	100.0%	0.0%	50.0%	20.9%	34.6%	22.8%	52.8%
RidgeCV	146,941	36,578	557,944	140,113	110,363	70.4%	20.7%	83.5%	52.1%	24.9%	22.6%	39.9%	11.1%
LassoCV	640,144	135,941	164,104	40,750	504,203	35.5%	76.9%	24.6%	50.7%	21.2%	33.3%	23.3%	40.5%
GRNBoost2	705,585	154,790	117,512	21,901	550,795	32.23%	87.60%	17.58%	52.59%	21.94%	35.09%	24.69%	48.04%
RTN (used by RTNduals)	39,944	8,044	636,407	168,647	31,900	76.27%	4.55%	95.23%	49.89%	20.14%	7.43%	51.41%	6.28%
NetREm (β = 1, α = 0.01)	398,202	88,625	358,730	88,066	309,577	52.9%	50.2%	53.7%	51.9%	22.3%	30.8%	27.1%	26.8%
NetREm (β = 1, α = 0.025)	175,392	44,009	536,924	132,682	131,383	68.7%	24.9%	80.3%	52.6%	25.1%	25.0%	37.9%	13.4%
NetREm (β = 1, α = 0.05)	79,676	22,753	611,384	153,938	56,923	75.0%	12.9%	91.5%	52.2%	28.6%	17.8%	49.4%	6.9%
NetREm (β = 1, α = 0.075)	38,148	12,162	642,321	164,529	25,986	77.5%	6.9%	96.1%	51.5%	31.9%	11.3%	57.7%	3.6%
NetREm (β = 1, α = 0.1)	20,413	6,943	654,837	169,748	13,470	78.3%	3.9%	98.0%	51.0%	34.0%	7.0%	62.2%	1.9%
NetREm (β = 1, α = 0.125)	12,235	4,145	660,217	172,546	8,090	78.6%	2.3%	98.8%	50.6%	33.9%	4.4%	64.0%	1.1%
NetREm (β = 1, α = 0.15)	7,951	2,648	663,004	174,043	5,303	78.8%	1.5%	99.2%	50.4%	33.3%	2.9%	64.7%	0.7%
NetREm (β = 1, α = 0.175)	5,414	1,698	664,591	174,993	3,716	78.9%	1.0%	99.4%	50.2%	31.4%	1.9%	64.3%	0.4%
NetREm (β = 1, α = 0.2)	30,509	9,523	647,321	167,168	20,986	77.7%	5.4%	96.9%	51.1%	31.2%	9.2%	58.7%	2.8%
NetREm (β = 0.1, α = 0.1)	53,587	17,428	632,148	159,263	36,159	76.9%	9.9%	94.6%	52.2%	32.5%	15.1%	55.4%	5.3%
NetREm (β = 10, α = 0.1)	59,778	19,451	627,980	157,240	40,327	76.6%	11.0%	94.0%	52.5%	32.5%	16.5%	54.5%	5.9%
NetREm (β = 100, α = 0.1)	185,328	88,185	571,164	88,506	97,143	78.0%	49.9%	85.5%	67.7%	47.6%	48.7%	56.2%	30.0%

Supplementary Table S11

Metrics for Predicting TF-TG Regulatory Links for Normalized Human Pluripotent Stem Cells (hPSCs)													
Goal:	?	↑	↑	↓	↓	↑	↑	↑	↑	↑	↑	↑	↑
Model	# of Rows (TF-TG)	True Positives	True Negatives	False Negatives	False Positives	Overall Accuracy	Sensitivity (recall)	Specificity	Balanced Accuracy (BACC)	Precision	F1 Score	AUPR	AUC
NetREm (β = 0.01, LassoCV)	227,849	44,773	856,732	184,129	183,076	71.1%	19.6%	82.4%	51.0%	19.7%	19.6%	34.3%	10.0%
NetREm (β = 0.1, LassoCV)	213,554	41,833	868,087	187,069	171,721	71.7%	18.3%	83.5%	50.9%	19.6%	18.9%	35.1%	9.4%
NetREm (β = 1, LassoCV)	122,412	24,019	941,415	204,883	98,393	76.1%	10.5%	90.5%	50.5%	19.6%	13.7%	42.3%	5.4%
NetREm (β = 10, LassoCV)	91,419	18,266	966,655	210,636	73,153	77.6%	8.0%	93.0%	50.5%	20.0%	11.4%	45.8%	4.2%
NetREm (β = 100, LassoCV)	181,614	37,234	895,428	191,668	144,380	73.5%	16.3%	86.1%	51.2%	20.5%	18.1%	38.0%	8.7%
NetREm (β = 1000, LassoCV)	186,968	38,035	890,875	190,867	148,933	73.2%	16.6%	85.7%	51.1%	20.3%	18.3%	37.5%	8.9%
Scribe	1,141,839	227,781	125,750	1,121	914,058	27.9%	99.5%	12.1%	55.8%	19.9%	33.2%	23.4%	54.9%
Pearson	1,141,839	227,781	125,750	1,121	914,058	27.9%	99.5%	12.1%	55.8%	19.9%	33.2%	21.4%	51.6%
Pearson mean	1,141,839	227,781	125,750	1,121	914,058	27.9%	99.5%	12.1%	55.8%	19.9%	33.2%	21.4%	51.6%
PIDC mean	1,141,839	227,781	125,750	1,121	914,058	27.9%	99.5%	12.1%	55.8%	19.9%	33.2%	23.2%	55.1%
MERLIN	180,983	38,179	897,004	190,723	142,804	73.7%	16.7%	86.3%	51.5%	21.1%	18.6%	38.0%	8.4%
SCENIC	246,267	56,778	850,319	172,124	189,489	71.5%	24.8%	81.8%	53.3%	23.1%	23.9%	35.0%	12.4%
Inferelator	43,991	9,580	1,005,397	219,322	34,411	80.0%	4.2%	96.7%	50.4%	21.8%	7.0%	53.0%	2.1%

knnDREMI	1,141,839	227,781	125,750	1,121	914,058	27.9%	99.5%	12.1%	55.8%	19.9%	33.2%	19.4%	50.4%
LEAP mean	1,141,839	227,781	125,750	1,121	914,058	27.9%	99.5%	12.1%	55.8%	19.9%	33.2%	19.7%	47.8%
SCODE mean	1,141,839	227,781	125,750	1,121	914,058	27.9%	99.5%	12.1%	55.8%	19.9%	33.2%	21.7%	53.4%
SILGGM mean	1,141,839	227,781	125,750	1,121	914,058	27.9%	99.5%	12.1%	55.8%	19.9%	33.2%	18.4%	46.4%
ElasticNetCV	272,884	54,265	821,189	174,637	218,619	69.0%	23.7%	79.0%	51.3%	19.9%	21.6%	32.0%	12.2%
Linear Regression	1,268,710	228,902	0	0	1,039,808	18.0%	100.0%	0.0%	50.0%	18.0%	30.6%	18.7%	51.1%
RidgeCV	1,268,710	228,902	0	0	1,039,808	18.0%	100.0%	0.0%	50.0%	18.0%	30.6%	18.7%	51.1%
LassoCV	266,294	53,032	826,546	175,870	213,262	69.3%	23.2%	79.5%	51.3%	19.9%	21.4%	32.3%	11.9%
GRNBoost2	874,285	170,774	336,297	58,128	703,511	40.0%	74.6%	32.3%	53.5%	19.5%	31.0%	21.3%	39.6%
RTN (used by RTNduals)	18,901	4,241	1,025,148	224,661	14,660	81.1%	1.9%	98.6%	50.2%	22.4%	3.4%	57.9%	13.5%

Supplementary Table S12

Metrics for Predicting TF-TG Regulatory Links for Normalized Budding Yeast Cells (YCs)													
Goal:	?	↑	↑	↓	↓	↑	↑	↑	↑	↑	↑	↑	↑
Model	# of Rows (TF-TG)	True Positives	True Negatives	False Negatives	False Positives	Overall Accuracy	Sensitivity (recall)	Specificity	Balanced Accuracy (BACC)	Precision	F1 Score	AUPR	AUC
NetREm (β = 0.01, LassoCV)	227,849	44,773	856,732	184,129	183,076	71.1%	19.6%	82.4%	51.0%	19.7%	19.6%	34.3%	10.0%
NetREm (β = 0.1, LassoCV)	213,554	41,833	868,087	187,069	171,721	71.7%	18.3%	83.5%	50.9%	19.6%	18.9%	35.1%	9.4%
NetREm (β = 1, LassoCV)	122,412	24,019	941,415	204,883	98,393	76.1%	10.5%	90.5%	50.5%	19.6%	13.7%	42.3%	5.4%
NetREm (β = 10, LassoCV)	91,419	18,266	966,655	210,636	73,153	77.6%	8.0%	93.0%	50.5%	20.0%	11.4%	45.8%	4.2%
NetREm (β = 100, LassoCV)	181,614	37,234	895,428	191,668	144,380	73.5%	16.3%	86.1%	51.2%	20.5%	18.1%	38.0%	8.7%
NetREm (β = 1000, LassoCV)	186,968	38,035	890,875	190,867	148,933	73.2%	16.6%	85.7%	51.1%	20.3%	18.3%	37.5%	8.9%
Scribe	1,141,839	227,781	125,750	1,121	914,058	27.9%	99.5%	12.1%	55.8%	19.9%	33.2%	23.4%	54.9%
Pearson	1,141,839	227,781	125,750	1,121	914,058	27.9%	99.5%	12.1%	55.8%	19.9%	33.2%	21.4%	51.6%
Pearson mean	1,141,839	227,781	125,750	1,121	914,058	27.9%	99.5%	12.1%	55.8%	19.9%	33.2%	21.4%	51.6%
PIDC mean	1,141,839	227,781	125,750	1,121	914,058	27.9%	99.5%	12.1%	55.8%	19.9%	33.2%	23.2%	55.1%
MERLIN	180,983	38,179	897,004	190,723	142,804	73.7%	16.7%	86.3%	51.5%	21.1%	18.6%	38.0%	8.4%
SCENIC	246,267	56,778	850,319	172,124	189,489	71.5%	24.8%	81.8%	53.3%	23.1%	23.9%	35.0%	12.4%
Inferelator	43,991	9,580	1,005,397	219,322	34,411	80.0%	4.2%	96.7%	50.4%	21.8%	7.0%	53.0%	2.1%
knnDREMI	1,141,839	227,781	125,750	1,121	914,058	27.9%	99.5%	12.1%	55.8%	19.9%	33.2%	19.4%	50.4%
LEAP mean	1,141,839	227,781	125,750	1,121	914,058	27.9%	99.5%	12.1%	55.8%	19.9%	33.2%	19.7%	47.8%
SCODE mean	1,141,839	227,781	125,750	1,121	914,058	27.9%	99.5%	12.1%	55.8%	19.9%	33.2%	21.7%	53.4%
SILGGM mean	1,141,839	227,781	125,750	1,121	914,058	27.9%	99.5%	12.1%	55.8%	19.9%	33.2%	18.4%	46.4%
ElasticNetCV	272,884	54,265	821,189	174,637	218,619	69.0%	23.7%	79.0%	51.3%	19.9%	21.6%	32.0%	12.2%
Linear Regression	1,268,710	228,902	0	0	1,039,808	18.0%	100.0%	0.0%	50.0%	18.0%	30.6%	18.7%	51.1%
RidgeCV	1,268,710	228,902	0	0	1,039,808	18.0%	100.0%	0.0%	50.0%	18.0%	30.6%	18.7%	51.1%
LassoCV	266,294	53,032	826,546	175,870	213,262	69.3%	23.2%	79.5%	51.3%	19.9%	21.4%	32.3%	11.9%
GRNBoost2	874,285	170,774	336,297	58,128	703,511	40.0%	74.6%	32.3%	53.5%	19.5%	31.0%	21.3%	39.6%
RTN (used by RTNduals)	45,327	2,769	1,407,408	46,389	42,558	94.1%	5.6%	97.1%	51.3%	6.1%	5.9%	36.6%	46.0%

Supplementary Table S13

Comparing the % of Predictable TFs and Perint (% of the Intersection) Across Datasets (Organisms: hPSCs, mDCs, YCs) for Various Models that Predict TF-TG Regulatory Networks						
**Model**	**% of Predictable TFs**			**Perint (% Intersection)**		
	**hPSCs**	**mDCs**	**Yeast**	**hPSCs**	**mDCs**	**Yeast**
ElasticNetCV	32.353	54.839	36.786	12.126	13.154	4.063
GRNBoost2	21.176	40.86	20.357	18.315	21.277	3.577
Inferelator	11.765	8.511	5.535	3.638	5.307	2.17
knnDREMI	0	0	0	19.929	28.065	3.37
LassoCV	32.353	53.763	40	11.994	12.743	4.069
LEAP mean	0	0	0	19.929	17.96	3.37
LinearRegression	0	0	0	18.042	20.91	3.279
MERLIN	26.144	11.111	16.236	10.271	9.454	3.733
NetREm (β= 0.01, α determined by LassoCV)	34.706	45.161	41.786	10.868	11.412	4.472
NetREm (β = 0.1, α determined by LassoCV)	34.706	46.237	41.786	10.442	11.422	4.487
NetREm (β = 1, α determined by LassoCV)	40.588	17.204	34.286	7.339	9.514	3.634
NetREm (β = 10, α determined by LassoCV)	36.471	9.677	16.071	6.047	6.235	3.162
NetREm (β = 100, α determined by LassoCV)	34.118	9.677	16.429	9.975	7.64	4.24
NetREm (β = 1000, α determined by LassoCV)	31.765	6.452	16.429	10.067	7.27	4.188
Pearson	0	0	0	19.929	39.512	3.37
Pearson mean	0	0	0	19.929	32.204	3.37
PIDC mean	0	0	0	19.929	22.266	3.37
RidgeCV	0	0	0	18.042	20.91	3.279
SCENIC	24.837	63.889	23.247	13.571	9.38	3.809
SCODE mean	0	0	0	19.929	23.951	3.37
Scribe	0	0	0	19.929	39.512	0
SILGGM mean	0	0	0	19.929	23.951	3.37

Supplementary Table S14

Please note that this is a simplification of **Figure 3A**, that is focused particularly on NetREm models relative to other models. ✎ We run NetREm for 5 different hyperparameter values for the prior network regularization: $\beta \in \{0.01, 0.1, 1, 10, 100, 1000\}$ and use LassoCV to subsequently optimize selection of α. Here, the models with lower rank have a more optimal performance for the given metric; for instance, rank 1 refers to the model that is in 1st place. We compare our 5 NetREm models against our 4 Benchmark Regression Models (BRMs) and these state-of-the-art cell-type GRN models: Inferelator; knnDREMI; mean: LEAP, Pearson, PIDC, SILGGM, SCODE; MERLIN; Pearson; SCENIC; Scribe, RTN, GRNBoost2. The yellow "Max NetREm Rank" column refers to rank of the NetREm model with the worst performance (i.e. the NetREm model in 'last place') across all 5 NetREm models. The blue "max rank across all models" column is the worst position # for that metric for that dataset. This is helpful since some metrics have ties among the models. For instance, for % of predictable TFs, several competitor models obtain 0. If there are ties, we assign the same rank to those tied models for that metric in that dataset. Hence, some metrics have a maximum ranking that is far less than the total # of models being compared. For example, the % of predictable TFs in hPSCs and YCs has a maximum rank of 12 for both; this reflects 12 distinct (unique) values for this metric in both datasets. The orange "NetREm improvement in rank is at least" column is the difference between the previous 2 columns and basically shows us that even NetREm's worst-performing model (for a given metric and dataset) still confers benefits and improvements since this improvement value is at least 1 across the metrics and datasets. That is, there is at least 1 competitor (non-NetREm) method that performs worse than any of our NetREm models do. Further, the optimal NetREm model would provide far greater improvement. The following 2 columns mention which NetREm model (or models) performs the best (low rank) for that

metric and dataset and which NetREm model (or models) performs the worst (this model has the rank shown in the corresponding entry in the yellow "Max NetREm rank" column).

Performance Evaluation of Relative Ranking of TF-TG Regulatory Networks Against Gold Standards: Comparing NetREm and Other Models															
Rank (Low # = Optimal Metric)	Max NetREm Rank	Max Rank Across All Models	NetREm improvement in rank is at least:	optimal NetREm (low #) β	worse NetREm (high #) β	Max NetREm Rank	Max Rank Across All Models	NetREm improvement in rank is at least:	optimal NetREm (low #) β	worse NetREm (high #) β	Max NetREm Rank	Max Rank Across All Models	NetREm improvement in rank is at least:	optimal NetREm (low #) β	worse NetREm (high #) β
	human pluripotent stem cells (hPSCs)					*budding yeast cells (YCs)*					*mouse dendritic cells (mDCs)*				
Specificity	9	15	6	10	0.01	9	15	6	10	0.01	13	20	7	1000	0.01
Balanced Accuracy (BACC)	12	15	3	100	10	11	15	4	0.01	10	18	20	2	100	10
Precision	13	15	2	100	0.1	7	15	8	10	0.01	17	20	3	100	1
Recall	13	15	2	0.01	10	13	15	2	0.01	10	18	20	2	0.1	10
F1 Score	13	15	2	0.01	10	13	15	2	0.1	10	18	20	2	0.1	10
Accuracy	10	15	5	10	0.01	9	15	6	10	0.01	14	20	6	1000	0.01
Area Under Precision Recall Curve (AUPR)	10	22	12	10	0.01	9	21	12	10	0.01	17	23	6	0.01	10
Area Under Receiver Operator Characteristic (ROC) Curve (AUC)	21	22	1	0.01	10	20	21	1	0.01	10	22	23	1	0.01	10
% Predictable TFs	6	12	6	1	1000	10	12	2	0.01; 0.1	10	12	13	1	0.1	1000
Perint: % of the Intersection	13	15	2	0.01	10	13	15	2	0.1	10	18	20	2	0.1	10
False Positive	9	15	6	10	0.01	9	15	6	10	0.01	13	20	7	1000	0.01
False Negative	13	15	2	0.01	10	13	15	2	0.01	10	18	20	2	0.1	10

For example, for the metric Area Under the Precision Recall Curve (AUPR) in hPSCs and YCs, NetREm's worst models (here, NetREm with $\beta = 10$ for both) still provides an improvement in at least 12 for the rank; we note that the ranks for this worst NetREm model is 10 and 9 in hPSCs and YCs, respectively, out of 22 and 21 possible ranks, respectively.

In all 3 applications (hPSCs, YCs, mDCs), NetREm models have a comparable performance to other 12 SOTA cell-type GRNs and 4 baseline regression models (BRMs). Here, models are ranked based on their relative performance where smaller rank values denote more optimal metrics. Network regularization hyperparameter β can be tuned to yield different results for the evaluation criteria. Nonetheless, in particular, all 5 NetREm models have relatively strong performance for Specificity, Accuracy, AUPR, and low False Positives relative to the other 16 models. AUPR has been used as a primary and preferred metric to measure the accuracy of the global GRN structure, especially given the imbalanced nature of the class distribution (abundance of negatives than positives)(McCalla et al. 2023; Pratapa et. al 2020; Skok Gibbs et al. 2024; Alawad et al. 2023; Seçilmiş et al. 2022; Sha et al. 2024; Drew et al. 2021). In hPSCs, NetREm($\beta = 1, \alpha$: LassoCV) model has the highest % of predictable TFs, a more sensitive metric that highlights the strengths of network inference methods (McCalla et al. 2023). In YCs, NetREm models have the highest BACC, Perint (% of intersection), F1 score, % of predictable TFs. In fact, (McCalla et al. 2023) evaluated the inferred networks using F1 score, AUPR, % of predictable TFs and found no single SOTA GRN outperformed the others across all datasets and gold standards. All 5 NetREm models have ranks that are within the range of ranks for all models, denoting that NetREm is a comparable cell-type GRN inference tool. In fact, (McCalla et al. 2023) evaluated the inferred networks using F1 score, AUPR, % of predictable TFs and found no single SOTA GRN outperformed the others across all datasets and gold standards. Moreover, (McCalla et al. 2023) found that methods like Inferelator and MERLIN, which use prior knowledge, tend to be wiser and outperform other GRN methods that solely use expression data to infer GRNs. Every method has its own strengths and weaknesses. Please note that this table is a simpler version of **Figure 3A**, which is truncated to focus on the 5 NetREm-based models.

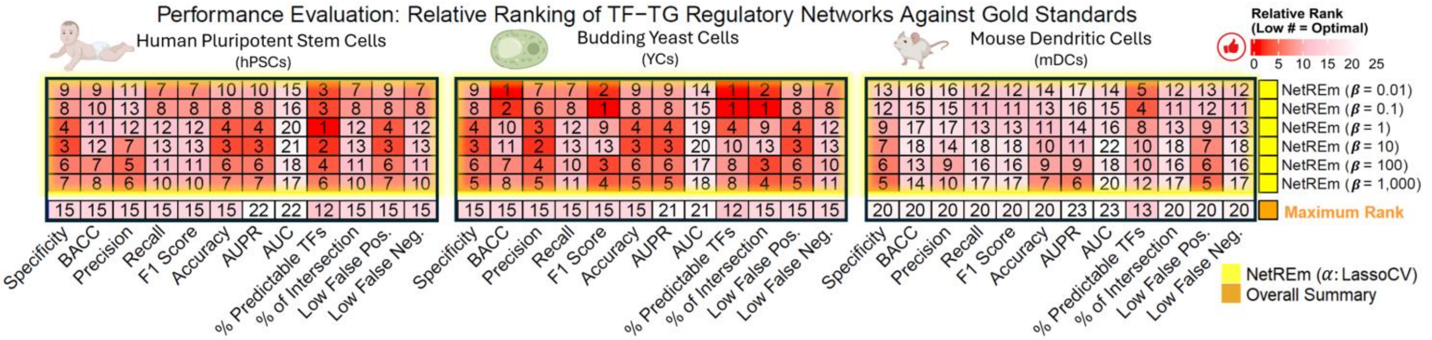

Supplementary Table S15

The mouse STRINGdb version 11 (v11) refers to the protein-protein interaction (PPI) network for mice as of January 19, 2019 to October 17, 2020. There are a couple STRINGdb networks in between that were released; nonetheless, we focus on versions 11 and 12. The later release of this PPI (and current version as of the timepoint this manuscript was written) is version 12 (v12), which includes data since July 26, 2023.

Reference Table:<br>Magnitude of TF-TF Coordination Score $|\bar{B}|$ Group Comparisons in Mouse Embryonic Stem Cells (mESCs)					
**Goal**	**Group 1**	**Group 2**	**Goal #**	# of TF-TF Coordination Links in Group 1	# of TF-TF Coordination Links in Group 2
known TF-TF links (in v11) versus links that are artificial (in v11)	PPI links in v11	PPI links not in v11	1	10,088	27,742
known links (in v11): comparing TF-TF links that are retained to those that are removed (in v12)	PPI links in v11 and v12	PPI links in v11 that are not in v12	2	8,590	1,498
artificial links (not in v11): comparing valid discovery links (found in v12) with those still not found in v12	PPI links not in v11 but found in v12	PPI links found in 0 networks (not in v11 and not in 12)	3	5,122	22,620
comparing valid discovery links (not in v11 but in v12) with removed links (False Positives; in v11 but not in v12)	PPI links not in v11 but found in v12	PPI links in v11 that are not in v12	4	5,122	1,498
comparing known links (any links in v11) with valid discovery links (not in v11 but in v12)	all PPI links found in v11	PPI links not in v11 but found in v12	5	10,088	5,122
comparing known and retained links (in v11 and v12) with valid discovery links (not in v11 but in v12)	PPI links in v11 and v12	PPI links not in v11 but found in v12	6	8,590	5,122
comparing unknown links (not in any network) with removed links (from v11 to v12)	PPI links found in 0 networks (not in v11 and not in 12)	PPI links in v11 that are not in v12	7 and 8	22,620	1,498

For other applications, please focus on just the first 4 columns: Goal, Group 1, Group 2, Goal #.

Supplementary Table S16

Alt. Hypothesis is the Alternative Hypothesis H_A and refers to Group 1 versus Group 2.

1-Sided t Test Comparison: TF-TF Coordination Scores in Mouse Embryonic Stem Cells (mESCs)												
Goal #	Metric	Test statistic t	degrees of freedom (df)	p-value	p-adj	significance (p)		Mean		Alt. Hypothesis	95% Confidence Bounds	
						p	p-adj	Group 1	Group 2		Low	Upper
1	Percentile (Average Coordination Score)	22.106	17,806.89	3.79E-107	6.07E-106	Yes	Yes	54.061	46.46	greater	7.04	Inf
2		11.311	2,103.66	3.85E-29	5.00E-28	Yes	Yes	55.408	46.34	greater	7.75	Inf
3		10.905	7,409.43	8.85E-28	9.74E-27	Yes	Yes	50.606	45.52	greater	4.322	Inf
4		5.0258	2,577.99	2.68E-07	1.61E-06	Yes	Yes	50.606	46.34	greater	2.87	Inf
5		6.6829	10,070.31	1.23E-11	9.87E-11	Yes	Yes	54.061	50.61	greater	2.605	Inf
6		9.0348	10,553.28	9.65E-20	9.65E-19	Yes	Yes	55.408	50.61	greater	3.928	Inf
7		-1.082	1,712.14	8.60E-01	1.00E+00	No	No	45.516	46.34	greater	-2.075	Inf
8		-1.082	1,712.14	1.40E-01	5.58E-01	No	No	45.516	46.34	less	-Inf	0.428
1	|Average Coordination Score B|	17.878	14,312.13	5.14E-71	7.72E-70	Yes	Yes	0.0104	0.007	greater	0.003	Inf
2		12.236	2,835.46	6.88E-34	9.63E-33	Yes	Yes	0.0111	0.006	greater	0.004	Inf
3		10.937	6,604.75	6.63E-28	7.96E-27	Yes	Yes	0.009	0.006	greater	0.002	Inf
4		6.5928	3,107.11	2.53E-11	1.77E-10	Yes	Yes	0.009	0.006	greater	0.002	Inf
5		4.7551	11,355.06	1.00E-06	5.02E-06	Yes	Yes	0.0104	0.009	greater	9E-04	Inf
6		6.8049	12,034.56	5.30E-12	4.77E-11	Yes	Yes	0.0111	0.009	greater	0.002	Inf
7		-0.118	1,706.17	5.47E-01	1.00E+00	No	No	0.0062	0.006	greater	-6E-04	Inf
8		-0.118	1,706.17	4.53E-01	1.00E+00	No	No	0.0062	0.006	less	-Inf	5E-04

- Novel discoveries (not in V11, in V12) than TF-TF links that are removed (in V11 but not in V12 PPIN). → NetREm prioritizes True Positives to False Positives and may be predictive of true meaningful future TF-TF PPIs.
- Novel discoveries than in TF-TF links that remain unknown (in neither network)
- in V11 networks than links that are not in V11 network → NetREm network prioritizes known links
- in both networks than novel discoveries à NetREm still prioritizes known links.
- in V11 network (may be removed or retained in V12) than novel discoveries → NetREm prioritizes known links.
- In both networks than links that are removed → NetREm prioritizes potential True Positives to False Positives

Supplementary Table S17

1-Sided t-Test Significance Results (P-adj < 0.05) of magnitude of TF-TF coordination scores $|\overline{B}|$ for Mouse Dendritic Cells Based on Input Mouse STRINGdb V11 Protein-Protein Interaction (PPI) Network (Older)

Varying α for the NetREm model (for β = 1)	Goal # (1 = significant, 0 = not significant)							Total # of Tests (Goals) the Model is Significant For
	**1**	**2**	**3**	**4**	**5**	**6**	**8**	
0.01	1	0	1	0	1	1	1	5
0.025	1	1	1	0	1	1	1	6
0.05	1	1	1	0	1	1	1	6
0.075	1	1	1	0	1	1	1	6
0.1	1	1	1	1	1	1	1	7
0.125	1	1	1	1	0	1	0	5
0.15	1	1	1	1	0	1	0	5
CV	1	1	1	0	1	1	1	6
# of models that are significant for goal	**8**	**7**	**8**	**3**	**6**	**8**	**6**	**46**

P-adj (i.e. adjusted p-value) is taken internally for each model. Altering the α for mDCs leads to additional statistically significant findings. Across these different NetREm models, we still find $|\bar{B}|$ is significantly higher for TF-TF links that are known (in V11) versus those that are not. Similarly, we find $|\bar{B}|$ is significantly higher for TF-TF links that are known and retained in both networks than for valid discoveries, which again shows how NetREm prioritizes known links. Nonetheless, we do find that NetREm's $|\bar{B}|$ is significantly higher for TF-TF links that are eventually discovered in V12 (valid discoveries) than those that still remain unknown in V12. This is a strong sign, showing NetREm's ability to prioritize future valid discoveries (with higher magnitude of coordination scores) to links that may be false positive (or at least not discovered yet). As we alter α, we do find significant results for additional 1-sided tests among the groups. This illustrates how NetREm may utilize an older PPI network and single-cell gene expression relationships to potentially uncover and predict novel relationships. There is a form of transfer learning.

Supplementary Table S18

1-Sided t Test Comparison: Magnitude of TF-TF Coordination Scores in Mouse Dendritic Cells (mDCs)

Goal #	α	Test statistic t	degrees of freedom (df)	p-value	p-adj	significance (p)		Mean		Alt. Hypothesis	95% Confidence Bounds	
						p	p-adj	Group 1	Group 2		Low	Upper
1	LassoCV	19.08	2,741.03	1.35E-76	1.08E-75	Yes	Yes	61.36	47.13	greater	13.002	Inf (Infinity)
2		2.85	309.69	0.00233	0.006989	Yes	Yes	62.06	56.73	greater	2.244	Inf
3		3.55	1,046.25	0.000199	0.000798	Yes	Yes	50.61	46.64	greater	2.1274	Inf
4		-3.03	412.36	0.998687	1	No	No	50.61	56.73	greater	-9.464	Inf
5		8.63	1,510.57	7.36E-18	4.42E-17	Yes	Yes	61.36	50.61	greater	8.7014	Inf
6		8.99	1,591.57	3.32E-19	2.32E-18	Yes	Yes	62.06	50.61	greater	9.3583	Inf
7		-5.72	247.6	1	1	No	No	46.64	56.73	greater	-13.01	Inf
8		-5.72	247.6	1.56E-08	7.80E-08	Yes	Yes	46.64	56.73	less	-Inf	-7.18
1	0.01	19.64	2,715.33	1.03E-80	8.24E-80	Yes	Yes	61.76	47.03	greater	13.498	Inf
2		1.81	318.36	0.035805	0.107415	Yes	No	62.2	58.9	greater	0.288	Inf
3		2.37	1,046.99	0.008903	0.035612	Yes	Yes	49.34	46.7	greater	0.8078	Inf
4		-4.84	423.23	0.999999	1	No	No	49.34	58.9	greater	-12.82	Inf

5		9.98	1,527.44	4.38E-23	2.63E-22	Yes	Yes	61.76	49.34	greater	10.372	Inf
6		10.08	1,618.12	1.67E-23	1.17E-22	Yes	Yes	62.2	49.34	greater	10.755	Inf
7		-7.12	248.86	1	1	No	No	46.7	58.9	greater	-15.02	Inf
8		-7.12	248.86	5.66E-12	2.83E-11	Yes	Yes	46.7	58.9	less	-Inf	-9.37
1	0.025	18.37	2,729.93	1.86E-71	1.49E-70	Yes	Yes	60.99	47.22	greater	12.529	Inf
2		2.65	314.65	0.004276	0.012827	Yes	Yes	61.63	56.75	greater	1.8359	Inf
3		3.67	1,055.59	0.00013	0.000518	Yes	Yes	50.76	46.73	greater	2.2192	Inf
4		-3.02	412.62	0.998642	1	No	No	50.76	56.75	greater	-9.272	Inf
5		8.31	1,540.92	1.08E-16	6.46E-16	Yes	Yes	60.99	50.76	greater	8.2004	Inf
6		8.61	1,627.77	8.20E-18	5.74E-17	Yes	Yes	61.63	50.76	greater	8.7941	Inf
7		-5.78	248.53	1	1	No	No	46.73	56.75	greater	-12.89	Inf
8		-5.78	248.53	1.13E-08	5.64E-08	Yes	Yes	46.73	56.75	less	-Inf	-7.16
1	0.05	17.6	2,753.82	4.16E-66	3.32E-65	Yes	Yes	60.47	47.35	greater	11.895	Inf
2		3.08	315.83	0.00114	0.003421	Yes	Yes	61.21	55.62	greater	2.5954	Inf
3		5.12	1,055.27	1.84E-07	9.04E-07	Yes	Yes	52.3	46.66	greater	3.8269	Inf
4		-1.68	419.31	0.953342	1	No	No	52.3	55.62	greater	-6.562	Inf
5		6.63	1,526.78	2.26E-11	1.36E-10	Yes	Yes	60.47	52.3	greater	6.1442	Inf
6		7.06	1,613.31	1.23E-12	8.58E-12	Yes	Yes	61.21	52.3	greater	6.8339	Inf
7		-5.23	249.26	1	1	No	No	46.66	55.62	greater	-11.78	Inf
8		-5.23	249.26	1.81E-07	9.04E-07	Yes	Yes	46.66	55.62	less	-Inf	-6.13
1	0.075	16	2,706.86	2.06E-55	1.65E-54	Yes	Yes	59.66	47.54	greater	10.871	Inf
2		2.83	310.55	0.002498	0.007495	Yes	Yes	60.37	55	greater	2.2351	Inf
3		5.6	1,044.45	1.40E-08	8.40E-08	Yes	Yes	53.05	46.77	greater	4.427	Inf
4		-0.96	408.54	0.830053	1	No	No	53.05	55	greater	-5.334	Inf
5		5.27	1,529.02	7.95E-08	3.97E-07	Yes	Yes	59.66	53.05	greater	4.5474	Inf
6		5.7	1,611.50	7.26E-09	5.08E-08	Yes	Yes	60.37	53.05	greater	5.2076	Inf
7		-4.6	247.02	0.999997	1	No	No	46.77	55	greater	-11.19	Inf
8		-4.6	247.02	3.43E-06	1.37E-05	Yes	Yes	46.77	55	less	-Inf	-5.27
1	0.1	16.91	2,689.28	2.34E-61	1.87E-60	Yes	Yes	55.17	41.12	greater	12.68	Inf
2		5.67	299.46	1.66E-08	9.99E-08	Yes	Yes	56.8	44.46	greater	8.7477	Inf
3		9.42	1,064.39	1.36E-20	9.53E-20	Yes	Yes	50.84	39.76	greater	9.1412	Inf
4		2.76	373.41	0.003023	0.009068	Yes	Yes	50.84	44.46	greater	2.5692	Inf
5		3.25	1,597.97	0.000587	0.00235	Yes	Yes	55.17	50.84	greater	2.1369	Inf
6		4.39	1,659.70	6.10E-06	3.05E-05	Yes	Yes	56.8	50.84	greater	3.723	Inf
7		-2.27	244.77	0.988081	0.988081	No	No	39.76	44.46	greater	-8.11	Inf
8		-2.27	244.77	0.011919	0.023838	Yes	Yes	39.76	44.46	less	-Inf	-1.29
1	0.125	18.65	2,425.85	6.32E-73	5.05E-72	Yes	Yes	39.78	22.43	greater	15.823	Inf
2		8.02	325.16	9.46E-15	5.68E-14	Yes	Yes	42.16	24.16	greater	14.295	Inf
3		13.61	1,005.01	4.06E-39	2.84E-38	Yes	Yes	37.75	20.28	greater	15.358	Inf
4		5.7	405.04	1.18E-08	5.92E-08	Yes	Yes	37.75	24.16	greater	9.6571	Inf
5		1.36	1,654.28	0.087545	0.17509	No	No	39.78	37.75	greater	-0.432	Inf
6		2.88	1,731.16	0.002038	0.008152	Yes	Yes	42.16	37.75	greater	1.8837	Inf
7		-1.86	242.98	0.968227	0.968227	No	No	20.28	24.16	greater	-7.319	Inf
8		-1.86	242.98	0.031773	0.09532	Yes	No	20.28	24.16	less	-Inf	-0.44

1	0.15	17	2,217.54	2.59E-61	2.07E-60	Yes	Yes	26.68	11.82	greater	13.418	Inf
2		9.29	381.76	5.81E-19	3.48E-18	Yes	Yes	28.95	11.72	greater	14.173	Inf
3		11.74	956.82	3.99E-30	2.79E-29	Yes	Yes	23.93	10.12	greater	11.873	Inf
4		6.16	474	7.58E-10	3.79E-09	Yes	Yes	23.93	11.72	greater	8.9483	Inf
5		1.95	1,708.62	0.025401	0.076202	Yes	No	26.68	23.93	greater	0.433	Inf
6		3.46	1,810.92	0.000274	0.001096	Yes	Yes	28.95	23.93	greater	2.6322	Inf
7		-0.97	242.78	0.832868	0.832868	No	No	10.12	11.72	greater	-4.32	Inf
8		-0.97	242.78	0.167132	0.334263	No	No	10.12	11.72	less	-Inf	1.13

Supplementary Table S19

1-Sided Welch t Test Comparison: Magnitude of TF-TF Coordination Scores in Human Peripheral Blood Mononuclear Cells (PBMCs) for Results that are Significant (P-adj < 0.05)

Goal #	Cell-type	Test statistic t	degrees of freedom (df)	p-adj	# of Values		Mean		Alt. Hypothesis	95% Confidence Bounds	
					Group 1	Group 2	Group 1	Group 2		Low	Upper
1	Naive CD4 T cell (C0)	83.9	61,975.6	0	54,880	875,380	57.49	46.31	greater	10.96	Inf
5		56.8	117,656.1	0	54,880	65,914	57.49	47.46	greater	9.75	Inf
6		47.5	69,934.5	0	34,766	65,914	57.26	47.46	greater	9.46	Inf
8		-57.5	21,262.0	0	809,466	20,114	46.21	57.90	less	-Inf	-11.35
1	CD14 Mono cell (C1)	145.9	60,949.1	0	50,930	749,200	65.35	48.72	greater	16.45	Inf
5		86.4	109,860.2	0	50,930	59,210	65.35	51.11	greater	13.97	Inf
6		83.1	78,500.9	0	33,044	59,210	66.58	51.11	greater	15.16	Inf
8		-82.8	19,362.9	0	689,990	17,886	48.51	63.09	less	-Inf	-14.29
1	Memory CD4 T cell (C2)	103.4	59,280.3	0	51,094	796,226	61.68	49.19	greater	12.29	Inf
2		2.7	41,491.6	9.8E-03	32,414	18,680	61.92	61.27	greater	0.25	Inf
5		68.5	112,358.1	0	51,094	62,244	61.68	50.37	greater	11.04	Inf
6		60.6	70,252.4	0	32,414	62,244	61.92	50.37	greater	11.23	Inf
8		-65.5	19,967.3	0	733,982	18,680	49.09	61.27	less	-Inf	-11.88
1	B cell (C3)	137.8	53,771.0	0	45,802	750,754	65.54	49.03	greater	16.31	Inf
5		92.2	104,436.5	0	45,802	59,366	65.54	50.17	greater	15.09	Inf
6		85.8	67,922.6	0	29,678	59,366	66.43	50.17	greater	15.95	Inf
8		-79.8	17,282.1	0	691,388	16,124	48.94	63.89	less	-Inf	-14.65
1	CD8 T cell (C4)	126.1	53,680.6	0	45,680	720,820	64.45	49.08	greater	15.17	Inf
5		86.8	102,360.0	0	45,680	57,028	64.45	49.68	greater	14.49	Inf
6		80.2	66,818.3	0	29,312	57,028	65.23	49.68	greater	15.23	Inf
8		-73.9	17,567.7	0	663,792	16,368	49.03	63.04	less	-Inf	-13.70
1	FCGR3A Mono cell (C5)	72.0	50,422.4	0	44,170	599,836	59.81	49.28	greater	10.29	Inf
5		48.3	92,535.7	0	44,170	49,816	59.81	50.46	greater	9.03	Inf
8		-56.6	16,107.7	0	550,020	15,146	49.17	61.69	less	-Inf	-12.15
1	Natural Killer cell (C6)	101.0	48,949.9	0	42,448	595,154	63.34	49.05	greater	14.06	Inf
5		69.7	89,625.6	0	42,448	47,786	63.34	50.02	greater	13.01	Inf
6		56.4	55,629.4	0	27,160	47,786	62.67	50.02	greater	12.28	Inf
8		-75.4	16,417.7	0	547,368	15,288	48.96	64.55	less	-Inf	-15.24

4		3.4	2,148.2	2.9E-03	2,938	1,134	50.87	47.68	greater	1.64	Inf
7	Platelet cell (C8)	3.1	1,297.8	6.5E-03	19,670	1,134	50.23	47.68	greater	1.21	Inf

Supplementary Table S20

1-Sided t-Test Significance Results (P-adj < 0.05) of magnitude of TF-TF coordination scores $\lvert\bar{B}\rvert$ for Human Peripheral Blood Mononuclear Cells Based on Input Human STRINGdb V11 PPI Network (Older)								
**NetREm run with b = 1, a from LassoCV**	**Goal #** (1 = significant, 0 = not significant)							Total # of Tests (Goals) the Cell-Type is Significant For
**Cell-type**	**1**	**2**	**4**	**5**	**6**	**7**	**8**	
B_C3	1			1	1		1	4
CD14_Mono_C1	1			1	1		1	4
CD8_T_C4	1			1	1		1	4
FCGR3A_Mono_C5	1			1			1	3
Memory_CD4_T_C2	1	1		1	1		1	5
Naive_CD4_T_C0	1			1	1		1	4
NaturalKiller_C6	1			1	1		1	4
Platelet_C8			1			1		2
# of cell-types that are significant for goal	**7**	**1**	**1**	**7**	**6**	**1**	**7**	**30**

Supplementary Table S21

Explanation	Metric	Barabasi PPIN	Final Interactome	HIPPIE	HuMAP2	HuRI HI-union	Max SAINT	Multiscale Interactome	Main NetREm PPIN	STRING v11 All	STRING v11 Physical	STRING v12 All	STRING v12 Physical	Contextual IID	Baseline NetREm (No Input PPIN)	
**Evaluating Overall Cell-type TF-TF Coordination Networks: Comparing the Performance of NetREm Run with the Different Input Protein-Protein Interaction (PPI) Networks (PPINs) Across 13 Different Human Contexts**																
Input PPIN	Jaccard Similarity with Evaluation Net	5.60%	2.47%	17.34%	2.24%	0.34%	11.40%	8.97%	90.80%	6.13%	4.86%	64.57%	14.90%	19.59%		
	Weighted (**w**) or dummy (binary: **d**)	dummy	dummy	w	w	dummy	w	dummy	w	w	w	w	w	dummy		
# of human contexts (of 13 Contexts) with **Performance Better than that of RTNduals**: Higher for Good Metrics, Lower for Bad Metrics	ALL Incorrect Links (Not in Eval. PPIN)	10	11	12	8	8	8	11	12	12	11	11	12	11	4	
	ALL Valid Links (Evaluation PPIN)	10	11	12	8	8	8	11	12	12	11	11	12	11	7	
	middle and/or stronger evidence links	12	10	12	3	3	6	13	10	13	11	9	13	10	4	
	middle evidence links (remainder: 0.5 <= score <= 0.7)	9	5	7	6	7	5	7	11	7	6	11	9	8	5	
	stronger evidence links	12	10	12	3	3	6	12	11	13	11	9	13	11	3	
	weaker evidence links	9	9	9	10	9	9	8	11	8	8	12	10	9	7	
	weakest evidence links (remainder: score < 0.5)	8	9	9	10	9	9	7	10	8	9	12	10	9	8	
# of human contexts (of 13 Contexts) with Tied (Equal) Performance to RTNduals	ALL Incorrect Links (Not in Eval. PPIN)	0	0	0	0	0	0	0	0	0	1	0	0	0	0	
	ALL Valid Links (Evaluation PPIN)	0	0	0	0	0	0	0	0	0	1	0	0	0	0	
	middle and/or stronger evidence links	0	0	0	0	0	1	0	0	0	1	0	0	1	0	
	middle evidence links (remainder: 0.5 <= score <= 0.7)	1	3	2	3	2	2	3	0	2	2	0	2	3	0	
	stronger evidence links	0	0	0	0	0	0	0	0	0	0	0	0	0	0	
	weaker evidence links	0	0	1	1	0	0	0	0	0	0	0	0	0	0	
	weakest evidence links (remainder: score < 0.5)	1	0	0	0	0	0	2	0	0	0	0	0	0	1	
# of human contexts (of 13 Contexts) with **Performance Worse than that for RTNduals**: Higher for Bad Metrics, Lower for Good Metrics	ALL Incorrect Links (Not in Eval. PPIN)	3	2	1	5	5	5	2	1	1	1	2	1	2	9	
	ALL Valid Links (Evaluation PPIN)	3	2	1	5	5	5	2	1	1	1	2	1	2	6	
	middle and/or stronger evidence links	1	3	1	10	10	6	0	3	0	1	4	0	2	9	
	middle evidence links (remainder: 0.5 <= score <= 0.7)	3	5	4	4	4	6	3	2	4	5	2	2	2	8	
	stronger evidence links	1	3	1	10	10	7	1	2	0	2	4	0	2	10	
	weaker evidence links	4	4	3	2	4	4	5	2	5	5	1	3	4	6	
	weakest evidence links (remainder: score < 0.5)	4	4	4	3	4	4	4	3	5	4	1	3	4	4	
Explanation	Metric	Barabasi PPIN	Final Interactome	HIPPIE	HuMAP2	huri_HI-union	Max SAINT	Multiscale Interactome	Main NetREm PPIN	STRING v11 All	STRING v11 Physical	STRING v12 All	STRING v12 Physical	Contextual IID	Baseline NetREm (No Input PPIN)	RTNduals
Average Percents (%) across 13 human contexts	ALL Incorrect Links (Not in Eval. PPIN)	40.144	40.112	39.270	41.485	41.893	41.455	38.966	39.610	39.622	40.009	39.262	39.036	39.347	50.660	42.931
	ALL Valid Links (Evaluation PPIN)	59.856	59.888	60.730	58.515	58.107	58.545	61.034	60.390	60.378	59.991	60.738	60.964	60.653	56.961	57.069
	Found by RTNduals	62.664	61.431	62.093	62.520	63.177	62.179	62.151	63.653	62.329	61.818	63.571	62.275	63.022	43.039	
	Input PPI Links	9.792	4.456	15.793	0.966	0.242	6.229	10.747	59.146	9.261	7.253	54.898	14.689	22.853		
	Links Not In Input PPI	90.208	95.544	84.207	99.034	99.758	93.771	89.253	40.854	90.739	92.747	45.102	85.311	77.147		
	middle evidence links (remainder: 0.5 <= score <= 0.7)	2.256	2.057	2.256	2.185	2.180	2.060	2.214	2.314	2.116	2.125	2.338	2.274	2.222	1.755	2.069
	Novel (middle evidence links (remainder: 0.5 <= score <= 0.7))	2.079	2.057	1.950	2.185	2.180	2.060	1.941		1.799	1.927		1.322	1.599		
	Novel (stronger evidence links)	19.299	22.933	13.324	24.105	24.649	19.438	18.347	1.244	20.241	21.266	5.840	15.488	8.995		
	Novel (weaker evidence links)	31.186	32.499	31.632	33.445	33.215	32.878	32.001		31.137	31.700		30.787	29.475		
	Novel (weakest evidence links (remainder: score < 0.5))	29.108	30.442	29.682	31.260	31.035	30.818	30.060		29.338	29.774		29.465	27.876		
	Novel Discoveries (False Negatives)	50.485	55.432	44.955	57.550	57.865	52.316	50.348	1.244	51.378	52.966	5.840	46.275	38.470		
	stronger evidence links	27.687	27.389	27.791	25.071	24.891	25.667	28.292	27.083	27.759	27.404	26.888	27.904	27.796	24.830	25.492
	True Positives	9.370	4.456	15.775	0.966	0.242	6.229	10.686	59.146	9.001	7.025	54.898	14.689	22.183		
	Still Unknown	39.723	40.112	39.252	41.485	41.893	41.455	38.906	39.610	39.361	39.781	39.262	39.036	38.677		
	weaker evidence links	32.168	32.499	32.939	33.445	33.215	32.878	32.742	33.307	32.620	32.587	33.850	33.060	32.857	32.131	31.577
	weakest evidence links (remainder: score < 0.5)	29.912	30.442	30.683	31.260	31.035	30.818	30.528	30.993	30.504	30.462	31.512	30.786	30.636	30.376	29.508

Supplementary Table S22

Relative Percentiles of TF activity for core Schwann cell TFs in terms of # of TGs they regulate. Metrics for the 8 core Schwann Cell (SC) TFs that we have additional experimental data for. Percentile (%) is between 0 and 1 and represents how many other SC-subtype TFs that given core TF is ahead of in terms of the # of TGs it is predicted to regulate. For instance, EGR2 is the 90.5% percentile, roughly, as NetREm's final model predicts it regulates 1,522 TGs in myelinating (mSCs) human Schwann cells (SCs); on average, the 221 TFs in mSCs tend to regulate around 829.15 TGs.

TF	mSC TGs	nmSC TGs	percentile (mSC)	percentile (nmSC)
EGR2	1,522	0	0.904977	0
NR2F2	508	1,239	0.285068	0.484649
RXRG	193	1,556	0.144796	0.649123
SOX10	1,003	1,472	0.613122	0.587719
SREBF1	1,004	1,610	0.61991	0.679825
STAT1	1,337	1,895	0.832579	0.842105
TEAD1	1,878	2,527	0.995475	0.969298
YY1	1,329	2,015	0.81448	0.907895

Global Metrics across all TFs in the Human Schwann Cell (SCs)		
	mSC TGs	nmSC TGs
# of TFs	221	228
Average	829.15	1,217.28
Median	839	1,279
Min	1	1
Max	2,126	3,188

We note that these core SC TFs tend to have relatively meaningful percentiles (in terms of the # of TGs they are predicted to regulate). In particular, TEAD1 is at the 99th and 97th percentile in mSCs and nmSCs, respectively. The lowest percentile is for RXRG in mSCs (14.5%), but we do show RXRG has stronger behavior in nmSCs (of 65th percentile). This may be attributed to RXRG's preferential expression in nmSCs over mSCs based on findings from single-cell data in both rodents and humans (Gerber et. al 2021). We note similar results for NR2F2.

Supplementary Table S23

The results of the 1-sided t-test comparison for testing if the Mean Square Error (MSE) Values for Schwann Cells (SCs) are less than those predicted by the other 4 benchmark models (RidgeCV, Linear Regression, ElasticNetCV, LassoCV) at the 5% level. We utilize the original target genes (TGs) predicted by the models without performing any filtering based on test Mean Square Errors (MSEs). Please note that for each pairwise Welch test, we use the R software $t.test$ function default of $var.equal = False$.

1-Sided t Test Comparison: Test Mean Square Error (MSE) Values for Schwann Cells SCs (Before Filtering)											
Alternative Hypothesis: Group 1 has a significantly lesser Test MSE than Group 2 does.											
SCs Type	Group 1	Group 2	Test statistic *t*	Degrees of freedom (df)	p-value	Mean		95% Confidence Bounds		# of Target Genes (ALL)	
						Group 1	Group 2	Low	Upper	Group 1	Group 2
mSC	NetREm	RidgeCV	-50.01	24,762.28	0	1.1761	2.211	-inf	-1.00055	12,396	12405
		Linear Regression	-81.59	23,389.55	0	1.1761	3.0820	-inf	-1.86747		12409
		ElasticNetCV	7.8038	15,954.37	1	1.1761	1.0469	-inf	0.15646		3614
		LassoCV	7.462	15,345.50	1	1.1761	1.0513	-inf	0.152293		3252
nmSC		RidgeCV	-7.242	26,904.00	2.27E-13	1.0957	1.1936	-inf	-0.07571	13,454	13452

		Linear Regression	-7.338	26,903.00	1.12E-13	1.0957	1.1949	-inf	-0.07701		13451
		ElasticNetCV	8.3306	16,382.44	1	1.0957	1.0113	-inf	0.101038		6152
		LassoCV	8.485	16,288.37	1	1.0957	1.0099	-inf	0.102424		5984

SCs Type	Group 1	Group 2	Test statistic *t*	Degrees of freedom (df)	p-value	Mean		95% Confidence Bounds		# of Target Genes (Common)	
						Group 1	Group 2	Low	Upper	Group 1	Group 2
mSC	NetREm	RidgeCV	-75.556	6,007.54	0	1.0432	2.1253	-inf	-1.05854	3,248	
		Linear Regression	-83.124	4,184.81	0	1.0432	3.0289	-inf	-1.94636		
		ElasticNetCV	-0.439	6,493.84	0.330495	1.0432	1.0485	-inf	0.014652		
		LassoCV	-0.655	6,493.83	0.256247	1.0432	1.0512	-inf	0.012025		
nmSC		RidgeCV	-24.617	11,954.53	0	1.0218	1.1366	-inf	-0.10714	5,980	
		Linear Regression	-24.911	11,954.44	0	1.0218	1.1380	-inf	-0.10853		
		ElasticNetCV	2.613	11,957.94	0.995513	1.0218	1.0097	-inf	0.019714		
		LassoCV	2.578	11,957.94	0.99502	1.0218	1.0099	-inf	0.019548		

Supplementary Table S24

Several previous studies (e.g., (Chen et. al 2023; Langfelder and Horvath 2008)) evaluate the degree exponent (γ) and coefficient of determination (R^2) to determine how closely a constructed network aligns with the properties of real biological networks. In this context, NetREm and scNET (Gupta et al. 2022) are applied to the same preprocessed gene expression data across control and Alzheimer's disease (AD) stages for 4 primary central nervous system (CNS) cell types in Application #12. Notably, scNET employs the scGRNom pipeline (Jin et al. 2021) to construct transcription factor (TF)-regulatory element-target gene (TG) networks using an ElasticNetCV framework, in contrast to the PPI-network-regularized regression framework used by NetREm. Consequently, we compare and assess the TF-TG regulatory network outputs from both NetREm and scNET. Since most real biological networks are known to exhibit scale-free properties (Barabási and Oltvai et. al 2004), this comparison highlights how NetREm's predicted gene regulatory networks (GRNs) potentially capture these characteristics better than scNET's respective GRNs do. This is evident in the rightmost columns of the table, labeled "improvement in scale-free topology by NetREm," which display the differences in γ and R^2 values between NetREm and scNET.

A network is generally classified as scale-free if the proportion of nodes with a given degree follows a power-law distribution $k^{-\gamma}$, with γ greater than 1. Here, k represents the "degree" of a node in the network. Specifically, it refers to the number of edges or connections that a node has to other nodes within the network. When analyzing the scale-free topology of biological networks, the degree distribution describes how many nodes have a certain degree k, and the distribution follows a power-law pattern, $k^{-\gamma}$, where γ is the degree exponent. In a scale-free network, a small number of nodes (often called "hubs") have a very high degree, while most nodes have a low degree. Some interpretations of the "scale-free hypothesis" impose stricter conditions, such as requiring $2 < \gamma < 3$ (Broido and Clauset 2019).

In all 8 contexts studied, NetREm consistently demonstrates a superior scale-free topology, characterized by a degree exponent (γ) closer to the ideal range of 2–3 and a coefficient of determination (R^2) nearer to 1. Improvements over

scNET range from increases of 0.343 to 1.032 in γ and 0.031 to 0.421 in R^2, indicating that NetREm more effectively captures the underlying properties of biological networks. Please see the metrics from the comparative analysis, below:

Comparing Properties of TF-TG Regulatory Networks between scNET and NetREm for Common Contexts: AD versus Control stages across 4 Brain-Related Cell-types

Gene Expression Data from Application #11		**# of TF-TG GRN Links**		**# of Transcription Factors (TFs)**		**# of Target Genes (TGs)**		**Degree Exponent: γ**		**Coefficient of Determination: R^2**		**Improvement in Scale-Free Topology by NetREm**	
**cell-type**	**condition**	NetREm	scNET	NetREm	scNET	NetREm	scNET	NetREm	scNET	NetREm	scNET	γ	R^2
Excitatory Neuron	AD	1,286,725	132,734	240	322	8,757	1,574	0.957	0.614	0.279	0.248	0.343	0.031
Inhibitory Neuron	AD	1,596,712	127,160	235	268	13,285	1,717	1.352	0.767	0.416	0.339	0.584	0.077
Microglia	AD	1,165,661	80,421	276	200	12,033	1,369	1.498	0.538	0.552	0.157	0.961	0.395
Oligodend.	AD	1,215,594	79,078	264	278	8,510	997	1.084	0.411	0.368	0.140	0.673	0.228
Excitatory Neuron	Control	1,584,288	135,700	238	329	11,393	1,575	1.148	0.646	0.340	0.275	0.502	0.065
Inhibitory Neuron	Control	1,512,851	132,261	238	278	13,368	1,722	1.444	0.712	0.469	0.302	0.733	0.167
Microglia	Control	1,082,935	80,185	277	203	11,838	1,360	1.523	0.491	0.552	0.132	1.032	0.421
Oligodend.	Control	1,549,340	78,754	261	278	10,966	996	1.168	0.411	0.374	0.139	0.757	0.235

Supplementary Table S25

TF-sign-TG Regulatory Network Evaluation: Comparison of NetREm and scNET with BrainSCOPE Resource (Reference Ground Truth GRN)

**Method for GRN Inference**	**Control cell-type**	**Metrics for Filtered TF-TG regulatory Networks (used for comparisons)**			**Network Overlap with BrainSCOPE**		
					TF-sign-TG links		
		common TFs	*common TGs*	*Total TF-sign-TG links*	*# of Common Links*	*Jaccard Similarity (JS) Score*	Average Area Under Precision Recall Curve (AUPR)
NetREm	Microglia	141	1,088	80,641	6,661	0.082601	0.686737
scNET	Microglia	158	986	65,606	3,407	0.051931	0.644920
NetREm	Oligo.	114	846	78,140	4,106	0.052547	0.712676
scNET	Oligo.	157	734	52,589	2,270	0.043165	0.648071

To assess biological relevance of our inferred TF-TG regulatory networks, we use the comprehensive and recently developed brainSCOPE resource (Emani et al. 2024; brainSCOPE Resource 2024), which provides a high-resolution atlas of cell-type-specific GRNs with detailed transcriptional profiles, as a proxy for signed ground truth networks in Controls. NetREm outperforms scNET, achieving a higher Jaccard similarity (JS) score of 0.083 in Mic, a 59.6% improvement over scNET's 0.052, and 0.053 in Oli, a 23.3% increase over scNET's 0.043. We also compute weighted signed average AUPR metrics on these filtered networks, ensuring that AUPR calculations focus on valid and comparable transcriptional

interactions across all models. Overall, NetREm outperforms scNET in predicting transcriptional regulatory interactions when compared to the brainSCOPE baseline in both Control Mic and Control Oli. In Mic, NetREm achieves an average AUPR of 0.687, compared to 0.645 for scNET, while in Oli, NetREm similarly performs better with an average AUPR of 0.713 versus 0.648 for scNET.

Supplementary Table S26

Overlaps in the # of top 500 genes based on random forest (RF) classifier models for predicting neurodegenerative disease or not based on gene-level TF-TF coordination score B changes for genes from Control to Alzheimer's disease (AD) Stages for the respective cell-type. This is a balanced classification task (undersampling class 0: True Negatives and/or False Negatives to equal class 1 links) based on the contextual Integrated Interactions Database (IID) (Kotlyar et. al 2022)

	**Pericytes**	**OPCs**	**Oligo**	**Microglia**	**In. Neuron**	**Ex. Neuron**	**Endo. BBB**	**Astrocytes**
**Pericytes**		22	25	25	27	23	33	28
**OPCs**	22		26	21	35	26	25	25
**Oligo**	25	26		29	28	22	19	24
**Microglia**	25	21	29		26	16	29	21
**In. Neuron**	27	35	28	26		28	19	27
**Ex. Neuron**	23	26	22	16	28		19	27
**Endo. BBB**	33	25	19	29	19	19		15
**Astrocytes**	28	25	24	21	27	27	15	

For instance: Astrocytes (Astro) share 24 TGs with Oligodendrocytes (Oligo); both cross-talk (via cell-cell contact, cytokines, signal molecules) is core for neuroinflammation, glial development, neuron regeneration (Nutma et al. 2020).

Supplementary Table S27

We present potential applications of NetREm to solve various problems in the field of bioinformatics and beyond. NetREm creates interpretable models that can be used to guide regression solutions, incorporate prior knowledge, and uncover relationships among the predictors. In addition to gene expression regression, NetREm can be extended to other emerging single-cell omics such as scATAC-seq to identify TF and chromatin interactions on open accessible regions at the cell-type level. By defining these components (predictors, response, input network, and goal), network-regularized regression can be effectively tailored to address specific bioinformatics challenges, leveraging prior knowledge to enhance predictive accuracy and biological interpretability. NetREm can be used for problems with continuous predictors (X) and response variable (y) where predictors (i.e. features) interact in functional subnetworks (that can be provided as input prior knowledge). It predicts which predictor groups impact y and how they associate and coordinate in networks to influence the target y.

**Application**	**Predictors (X)**	**Response Variable (y)**	**Input Network among Predictors**	**Goal**
GRN Inference	Gene expression levels of transcription factors (TFs)	Expression levels of a target gene (TG)	Protein-protein interaction network (PPIN) or known co-regulatory interactions among TFs	Predict TG expression based on TF expression, identifying key TFs and their coordination.
Protein Function Prediction	Gene expression levels or protein abundances	Functional annotations or scores (e.g., enzymatic activity)	Protein-protein interaction network (PPIN)	Predict functions of uncharacterized proteins based on known interactions.
Disease Gene Identification	Genetic/genomic data (e.g., gene expression levels, SNP genotypes)	Disease phenotype presence/absence or severity score	Protein-protein interaction network (PPIN) or gene-disease association networks	Identify genes associated with diseases and how genetic variations influence disease risk.

Pathway Analysis	Gene expression levels or protein abundances	Pathway activation scores or pathway-specific gene expression levels	Pathway interaction network or gene-gene interaction network within pathways	Predict activation status of biological pathways and discover/refine pathway interactions.
Single-Cell Analysis	Single-cell RNA-seq gene expression levels	Cell type-specific gene expression profiles or differentiation states	PPI network (PPIN) or similarity network from single-cell regulatory network embeddings	Infer cell type-specific regulatory networks and model dynamic changes during differentiation.
Drug Response Prediction	Gene expression levels, protein abundances, or genetic variants	Drug response measures (e.g., sensitivity, resistance)	Drug-target interaction network or gene-drug interaction network	Predict individual responses to drugs based on molecular data for personalized medicine.
Epigenomics	Chromatin state features, histone modification levels, or DNA methylation	Gene expression levels or chromatin state activation scores	Chromatin interaction network or histone modification interaction network	Predict impact of chromatin states and histone modifications on gene regulation.
Integration of Multi-Omics Data	Combined features from multiple omics layers	Phenotypic traits, disease states, or molecular signatures	Integrated multi-omics network combining interactions from various omics layers	Build comprehensive models leveraging multi-omics data for complex traits and diseases.
Non-Coding SNP Impact Analysis	Genotype data for non-coding SNPs	Gene expression levels, chromatin accessibility, or regulatory activity	Regulatory network connecting SNPs to regulatory regions and target genes	Predict impact of non-coding SNPs on gene expression and regulatory activity.
Epistasis Analysis	Genotype data for pairs or groups of SNPs	Phenotypic traits or disease states	Epistatic interaction network encoding interactions between genetic variants	Identify and quantify significant epistatic interactions influencing complex traits/disease.
Polygenic Risk Score Calculation	Genotype data for multiple genetic variants	Disease phenotype or quantitative trait measurement	Network capturing interactions among genetic variants (SNP-SNP or gene-gene interaction network)	Calculate individual polygenic risk scores considering genetic variant interactions.

Supplementary Methods

Section §1: Mathematical Methods Work for NetREm:

Pre-processing gene expression data:

§1.1 Integrating multimodal data & networks in NetREm workflow

Pre-processing gene expression data:

For the M cell samples, $X^{(0)} \in \mathbb{R}^{M \times N}$ matrix contains expression data for N predictor TFs, while $y^{(0)} \in \mathbb{R}^{M}$ is the expression vector for TG. $X^{(0)}$ dimensions are TG-specific based on N TFs. Expression data can be in different units (Zhao et al. 2021) and may undergo pre-processing using toolkits (e.g. Scanpy(Wolf et al. 2018), Seurat(Satija et. al 2015)). Nonetheless, we standardize $X^{(0)}$ to obtain X with $X_{ij} = \frac{X_{ij}^{(0)} - \overline{\mu_j^{(0)}}}{\sigma_j^{(0)}}$ where $\overline{\mu_j^{(0)}}$ is the mean and $\sigma_j^{(0)} > 0$ is standard deviation of TF_j in $X^{(0)}$. Each TF in X has $\bar{\mu}_j \approx 0$ and $\sigma_j \approx 1$ and any previous units are removed. Since r is invariant to scaling, original pairwise r are preserved. Similarly, we standardize $y^{(0)}$ to obtain y with $\mu_y \approx 0$, $\sigma_y \approx 1$. X and y are our final unitless, standardized expression input data.

§1.2 Step 1: Network regularized regression (Problem Definition)

Transforming NetREm to ElasticNet Problem:

Our objective function $f(c) = \frac{1}{2M}\left|\left|y - Xc\right|\right|^2 + \alpha\left|\left|c\right|\right|_1 + \beta c^T Ac$ employs both L1 (Lasso) and L2 (Ridge) penalties to mitigate issues of collinearity. Even when the problem is transformed into a Lasso problem $\tilde{f}(c) = \frac{1}{2N}\left|\left|\tilde{y} - \tilde{X}c\right|\right|^2 + \alpha\left|\left|c\right|\right|_1$, the objective function's value $\tilde{f}(c)$ remains equivalent to $f(c)$ (subject to a constant), hence still addressing collinearity concerns. In the special case where $A = I$, the identity matrix (indicating a fully disconnected network prior), our objective transforms into the Elastic Net $f(c) = \frac{1}{2M}\left|\left|y - Xc\right|\right|^2 + \alpha\left|\left|c\right|\right|_1 + \beta\left|\left|c\right|\right|^2$. Given that we typically make our TG-specific input PPI network W fully-connected with artificial edge weight η for missing edges, we may set $w = \eta$ =1e-8 or some very small number so W is disjoint and reflects a scaled version of the Identity Matrix.

§1.3 Step 2: Gene expression embeddings from network regression

Matrix definitions:

A, D, W and $V \in \mathbb{R}^{N \times N}$ and all are symmetric matrices (i.e. $A = A^T, D = D^T, W = W^T, V = V^T$).

- Degree-based matrix D where $d_i = \sum_{j=1,\ j \neq i}^{N} w_{ij}$

$$D = \begin{bmatrix} \frac{1}{\sqrt{d_1}} & 0 & \cdots & 0 \\ 0 & \frac{1}{\sqrt{d_2}} & \cdots & 0 \\ \vdots & \vdots & \ddots & \vdots \\ 0 & 0 & \cdots & \frac{1}{\sqrt{d_N}} \end{bmatrix}_{N \times N} = D^T$$

- Input Protein-Protein Interaction Network weight matrix W where $w_{ij} = w_{ji} > 0$:

$$W = \begin{bmatrix} \frac{\sum_{j=2,\ i \neq j}^{N} w_{1j}}{N-1} & w_{12} & \cdots & w_{1N} \\ w_{21} & \frac{\sum_{j=1,\ i \neq j}^{N} w_{2j}}{N-1} & \cdots & w_{2N} \\ \vdots & \vdots & \ddots & \vdots \\ w_{N1} & w_{N2} & \cdots & \frac{\sum_{j=1, i \neq j}^{N} w_{Nj}}{N-1} \end{bmatrix}_{N \times N} = \begin{bmatrix} \frac{d_1}{N-1} & w_{12} & \cdots & w_{1N} \\ w_{21} & \frac{d_2}{N-1} & \cdots & w_{2N} \\ \vdots & \vdots & \ddots & \vdots \\ w_{N1} & w_{N2} & \cdots & \frac{d_N}{N-1} \end{bmatrix}_{N \times N}$$

We do not consider self-loops; instead $W_{ii} = \frac{d_i}{N-1}$ where TF_i's degree with $N - 1$ other TFs is $d_i = \sum_{k \neq i} w_{ik} > 0$.

- V matrix: $V = N \cdot I - 11^T = \begin{bmatrix} N-1 & -1 & \cdots & -1 \\ -1 & N-1 & \cdots & -1 \\ \vdots & \vdots & \ddots & \vdots \\ -1 & -1 & \cdots & N-1 \end{bmatrix}_{N \times N}$
- Intermediate matrix $W \odot V$: element-wise or Hadamard product of the W and V matrices.

$$W \odot V = \begin{bmatrix} d_1 & -w_{12} & \cdots & -w_{1N} \\ -w_{21} & d_2 & \cdots & -w_{2N} \\ \vdots & \vdots & \ddots & \vdots \\ -w_{N1} & -w_{N2} & \cdots & d_N \end{bmatrix}_{N \times N}$$

- Network-regularization based matrix A:

$$A = D^T (W \odot V) D = \begin{bmatrix} 1 & -w_{12}/\sqrt{d_1 d_2} & \cdots & -w_{1N}/\sqrt{d_1 d_N} \\ -w_{21}/\sqrt{d_1 d_2} & 1 & \cdots & -w_{2N}/\sqrt{d_2 d_N} \\ \vdots & \vdots & \ddots & \vdots \\ -w_{N1}/\sqrt{d_1 d_N} & -w_{N2}/\sqrt{d_2 d_N} & \cdots & 1 \end{bmatrix}_{N \times N}$$

Proof of Equivalence between Summation and Matrix Form:

The original network-based regularization term in the optimization problem is given by:

$$\frac{\beta}{2} \sum_{i=1}^{N} \sum_{j=i}^{N} w_{ij} \left(\frac{c_i}{\sqrt{d_i}} - \frac{c_j}{\sqrt{d_j}} \right)^2 = \frac{\beta}{2} c^T A c$$

We need to show that this sum is equivalent to the form $\frac{\beta}{2} c^T A c$ where the symmetric matrix $A \in \mathbb{R}^{N \times N}$ is defined as:

$$A = D^T (W \odot V) D$$

Here, D is a diagonal matrix where $D_{ii} = 1/\sqrt{d_{ii}}$ and $W \odot V$ represents element-wise product of matrices W and V.

Expanding the Matrix Expression: We start by expanding the quadratic form: $c^T A c = c^T D^T (W \odot V) D c = (Dc)^T (W \odot V)(Dc)$. Please let $u = Dc$, such that $u_i = c_i / \sqrt{d_i}$. Thus, the expression becomes:

$$u^T (W \odot V) u = \sum_{i=1}^{N} \sum_{j=i}^{N} u_i (W \odot V)_{ij} u_j$$

That is, the expression for $c^T A c$ transforms to $\sum_{i=1}^{N} \sum_{j=i}^{N} u_i (W \odot V)_{ij} u_j$.

Examining $W \odot V$ Since matrix V is defined to modify the diagonal entries of W, we have $V = N \cdot I - 11^T$. Here, 1 is a vector of all ones. The Hadamard (element-wise) product $W \odot V$ adjusts W based on V, particularly affecting the diagonal elements. This matrix helps translates the interactions defined in W into the regularization context, taking into account the adjustments from V.

Proof of Equivalence: The goal is to show that $\sum_{i=1}^{N} \sum_{j=i}^{N} u_i (W \odot V)_{ij} u_j = 2 \sum_{i=1}^{N} \sum_{j=i}^{N} w_{ij} (u_i - u_j)^2$

Since W is symmetric and without self-loops, and V modifies the diagonal entries to reflect subtracting the sum across rows, the off-diagonal terms of $W \odot V$ essentially remain w_{ij} for $i \neq j$, and:

$$(W \odot V)_{ij} = \begin{cases} d_i & \text{if } i = j \\ -w_{ij} & \text{if } i \neq j \end{cases}$$

Now, please note that the quadratic form expands as: $\sum_{i=1}^{N} \sum_{j=i}^{N} u_i (W \odot V)_{ij} u_j = \sum_{i=1}^{N} u_i^2 d_i - \sum_{i=1}^{N} \sum_{j \neq i} u_i w_{ij} u_j$

Which, for $i < j$, can be rearranged into: $\sum_{i=1}^{N} \sum_{j=i}^{N} 2 w_{ij} (u_i - u_j)^2$

Conclusion: Hence, the term $c^T A c$ accounts for each pair (i, j) with $i \neq j$ twice, as needed. Therefore, to match the original regularization term, we need the ½ factor: $\frac{\beta}{2} \sum_{i=1}^{N} \sum_{j=i}^{N} w_{ij} \left(\frac{c_i}{\sqrt{d_i}} - \frac{c_j}{\sqrt{d_j}} \right)^2 = \frac{\beta}{2} c^T A c$

Details regarding SVD on E matrix:

$\Sigma^{\frac{1}{2}}$ and $\Sigma^{-\frac{1}{2}}$ are diagonal matrices with entries $\sqrt{S}$ and $1/\sqrt{S}$, respectively. The condition # of E with respect to a particular norm, $\kappa(E)$, measures how close E is to being singular. For the Euclidean norm, $\kappa(E) = \frac{s_{\max}}{s_{\min}}$. If $\kappa(E)$ is very large (e.g. 1e6), E may be nearly singular and ill-conditioned and results may be numerically unstable as inverting E (i.e. compute

E^{-1}) can be difficult; in these unfortunate cases, ≥1 eigenvalue of E is ≈0, which is associated with increased variance of c^*. Small changes in inputs can cause large changes in c^*. In high-throughput data, there may be high multicollinearity, especially when $M \ll N$, so regression models may face problems related to a severely ill-conditioned Gram matrix X^TX (Lê Cao et al. 2011; Hastie 2020). Adding βA to the Gram matrix to yield E may help eliminate potential issues arising from numerical instability in inverting X^TX, by ensuring E is well-conditioned (increasing smallest singular values of X^TX to reduce $\kappa(E)$) and invertible. Nonetheless, our sparsity prior α on $\tilde{X}$ in $Eq(4)$, is an effective regularization penalty, helping tackle cases of an ill-conditioned E, stabilizing and guiding results, performing feature selection (Lê Cao et al. 2011; Hastie 2020; Kelner et al. 2021); by shrinking c^*, Lasso regression mitigates c^*'s sensitivity to errors/changes in inputs. Nonetheless, we endeavor to preempt any risk of an ill-conditioned E (i.e. $\kappa(E) \geq 1 \times 10^6$) via pseudo-inverses with an adaptive minimum threshold $t = \frac{s_{\max}}{1\times10^6}$. Singular value $s_i \in S$ is problematic (corresponding to noise or redundant info) if $s_i \leq t$. In such cases, we set $\left[\Sigma^{\frac{1}{2}}\right]_i = 0$ (instead of $\sqrt{s_i}$) and $\left[\Sigma^{-\frac{1}{2}}\right]_i = 0$ (instead of $1/\sqrt{s_i}$). Our modification ignores directions (deemed unstable or insignificant in terms of data variance) like s_i, concentrating on strong singular values (most pertinent features), zeroing out roles of smaller ones in subsequent computations. This remedy enhances NetREm's interpretability, strengthening E's robustness against noise, addressing potential challenges related to M to N ratio (e.g. E being rank deficient).

Proof for the $A = D^T(W \odot V)D$ matrix:

$A,\ D,\ W$ and $V \in \mathbb{R}^{N\times N}$ and all are symmetric matrices (i.e. $A = A^T, D = D^T, W = W^T, V = V^T$). $W \circ V$ multiplies $w_{ii} = \frac{\sum_{j=1,\ i\neq j}^{N} w_{ij}}{N-1}$ by $N-1$ to yield $d_i = \sum_{j=1,\ i\neq j}^{N} w_{ij}$ along the main diagonal and multiplies off-diagonal w_{ij} by -1 for $i \neq j$. Thus, $(W\odot V)_{ii} = d_i$ and $(W\odot V)_{ij} = -w_{ij}$ for $i \neq j$ where $(W\odot V) \in \mathbb{R}^{N\times N}$. Since D is symmetric, $D = D^T$. Since $D_{ii} = 1/\sqrt{d_i}$ and $D_{ij} = 0$ for $i \neq j$, then $D^T(W\odot V)$ will be:

$$\begin{bmatrix} 1/\sqrt{d_1} & 0 & \cdots & 0 \\ 0 & 1/\sqrt{d_2} & \cdots & 0 \\ \vdots & \vdots & \ddots & \vdots \\ 0 & 0 & \cdots & 1/\sqrt{d_N} \end{bmatrix}_{N\times N} \begin{bmatrix} d_1 & -w_{12} & \cdots & -w_{1N} \\ -w_{21} & d_2 & \cdots & -w_{2N} \\ \vdots & \vdots & \ddots & \vdots \\ -w_{N1} & -w_{N2} & \cdots & d_N \end{bmatrix}_{N\times N} =$$

$$\begin{bmatrix} \sqrt{d_1} & -w_{12}/\sqrt{d_1} & \cdots & -w_{1N}/\sqrt{d_1} \\ -w_{21}/\sqrt{d_2} & \sqrt{d_2} & \cdots & -w_{2N}/\sqrt{d_2} \\ \vdots & \vdots & \ddots & \vdots \\ -w_{N1}/\sqrt{d_N} & -w_{N2}/\sqrt{d_N} & \cdots & \sqrt{d_N} \end{bmatrix}_{N\times N}$$

.That is, $D^T(W\odot V) \in \mathbb{R}^{N\times N}$ where the main diagonal terms are $\sqrt{d_i}$ for $i = 1, \dots, N$ and the off-diagonal elements are scaled versions of the original weights w_{ij} divided by $\sqrt{d_i}$ based on the row i, for $i \neq j$. Hence, $A = D^T(W\odot V)D$ evaluates to:

$$\begin{bmatrix} \sqrt{d_1} & -w_{12}/\sqrt{d_1} & \cdots & -w_{1N}/\sqrt{d_1} \\ -w_{21}/\sqrt{d_2} & \sqrt{d_2} & \cdots & -w_{2N}/\sqrt{d_2} \\ \vdots & \vdots & \ddots & \vdots \\ -w_{N1}/\sqrt{d_N} & -w_{N2}/\sqrt{d_N} & \cdots & \sqrt{d_N} \end{bmatrix}_{N\times N} \begin{bmatrix} 1/\sqrt{d_1} & 0 & \cdots & 0 \\ 0 & 1/\sqrt{d_2} & \cdots & 0 \\ \vdots & \vdots & \ddots & \vdots \\ 0 & 0 & \cdots & 1/\sqrt{d_N} \end{bmatrix}_{N\times N} =$$

$$\begin{bmatrix} 1 & -w_{12}/\sqrt{d_1 d_2} & \cdots & -w_{1N}/\sqrt{d_1 d_N} \\ -w_{21}/\sqrt{d_1 d_2} & 1 & \cdots & -w_{2N}/\sqrt{d_2 d_N} \\ \vdots & \vdots & \ddots & \vdots \\ -w_{N1}/\sqrt{d_1 d_N} & -w_{N2}/\sqrt{d_2 d_N} & \cdots & 1 \end{bmatrix}_{N\times N}$$

. Thus, A has a main diagonal where $A_{ii} = 1$ for $i = 1, \dots, N$ and it has off-diagonal terms that are $A_{ij} = -w_{ij}/\sqrt{d_i d_j}$ where $i \neq j$.

The importance of βA for potentially reducing rank deficiency:

Here, $E = X^TX + \beta A$. Overall, adding βA to yield the E matrix effectively improves numerical stability by addressing both rank deficiency and ill-conditioning. Rank-deficient matrices are often ill-conditioned because the presence of linear dependencies among rows or columns means that some singular values are ≈0, inflating $\kappa(E)$. The rank of a matrix signifies the maximum number of linearly independent columns or rows within the matrix. For instance, the maximum

rank X can have is $\min(M, N)$. X is rank deficient if $\text{rank}(X) < \min(M, N)$, indicating there are redundant rows (if $N < M$, not all TFs are linearly independent) or columns (if $M > N$, then not all samples are linearly independent across TFs) in X, which do not add any new information or dimension to the space spanned by X. Here, $X^T X$ can have a maximum rank: $\min\big(\text{rank}(X), (\text{rank}(X^T X)\big) = \min(M, N)$. Since A behaves similarly to a graph Laplacian L, it has $N - 1$ linearly independent rows and columns (i.e. rank of $N - 1$ and rank deficient by 1). According to spectral graph theory properties(Klee and Stamps 2022), for a fully-connected graph, L has exactly one 0 eigenvalue (corresponding eigenvector is a constant vector of 1's representing the direction along which all TF node potentials are equal) and $N - 1$ positive eigenvalues. The second term (βA) has rank $N - 1$.

Theoretically, when $M < N - 1$, the first data term ($\frac{1}{M} X^T X$) in $Eq(3a)$, indeed is rank deficient with rank up to M since $\text{rank}(X^T X) = \min(M, N) = M$. And, $\text{rank}(A)$ is always $N - 1$ since it is a modified Laplacian matrix. When we add 2 semi-definite matrices (i.e. $X^T X$ and A), the rank is at least the minimum of the ranks of both matrices. That is, $\text{rank}(E) \geq \min\big(\text{rank}(X^T X), \text{rank}(A)\big) = \min\big(\min(M, N), \text{rank}(A)\big) = \min(\min(M, N), N - 1) = \min(M, N - 1) = M$. Hence, $\text{rank}(E) \geq M$. Then, the combined matrix terms in the embedding matrix $\frac{1}{N} \tilde{X}^T \tilde{X}$ (that is based on Singular Value Decomposition results of E) may have a rank that is at least M, showing how network regularization may alleviate potential rank deficiency problems. In ideal cases with more samples than features ($N < M$), there is a reduced risk of overfitting and X may have full column rank of N; then the primary result of adding βA is a modified eigenstructure (i.e. structure and spectral properties) of $\frac{1}{M} X^T X$.In the case of full rank, we cannot guarantee that the condition number will be improved compared with $X^T X$, unless A is an identity matrix.

Proving Gene expression embedding for the target gene expression $\tilde{y}$:

Please note that here we verify $\frac{1}{N} \tilde{y}^T \tilde{X} = \frac{1}{M} y^T X$ $\qquad Eq(3b)$

Given that $\tilde{X} = \sqrt{N} \Sigma^{\frac{1}{2}} U^T$ and $\tilde{y} = \frac{\sqrt{N}}{M} \Sigma^{-\frac{1}{2}} U^T X^T y$. Then, $\tilde{y}^T = \frac{\sqrt{N}}{M} \left(\Sigma^{-\frac{1}{2}} U^T X^T y \right)^T$

$$\tilde{y}^T = \frac{\sqrt{N}}{M} \left(\Sigma^{-\frac{1}{2}} U^T X^T y \right)^T = \frac{\sqrt{N}}{M} (y)^T (X^T)^T (U^T)^T \left(\Sigma^{-\frac{1}{2}} \right)^T = \frac{\sqrt{N}}{M} y^T X U \left(\Sigma^{-\frac{1}{2}} \right)^T$$

Here, $\Sigma \in \mathbb{R}^{N \times N}$ is a diagonal matrix of the singular values $\{s_1, s_2, \dots, s_N\}$ of E

$$\Sigma^{-\frac{1}{2}} = \begin{bmatrix} s_1^{-1/2} & 0 & 0 & \cdots & 0 \\ 0 & s_2^{-1/2} & 0 & \cdots & 0 \\ 0 & 0 & s_3^{-1/2} & \cdots & 0 \\ \vdots & \vdots & \vdots & \ddots & \vdots \\ 0 & 0 & 0 & \cdots & s_N^{-1/2} \end{bmatrix}_{N \times N} = \begin{bmatrix} 1/\sqrt{s_1} & 0 & 0 & \cdots & 0 \\ 0 & 1/\sqrt{s_2} & 0 & \cdots & 0 \\ 0 & 0 & 1/\sqrt{s_3} & \cdots & 0 \\ \vdots & \vdots & \vdots & \ddots & \vdots \\ 0 & 0 & 0 & \cdots & 1/\sqrt{s_N} \end{bmatrix}_{N \times N} = \left(\Sigma^{-\frac{1}{2}} \right)^T$$

Hence, $\left(\Sigma^{-\frac{1}{2}} \right)^T = \Sigma^{-\frac{1}{2}}$

$\tilde{y}^T = \frac{\sqrt{N}}{M} \left(\Sigma^{-\frac{1}{2}} U^T X^T y \right)^T = \frac{\sqrt{N}}{M} (y)^T (X^T)^T (U^T)^T \left(\Sigma^{-\frac{1}{2}} \right)^T = \frac{\sqrt{N}}{M} y^T X U \, \Sigma^{-\frac{1}{2}}$. Then, we note that: $\tilde{y} = (\tilde{y}^T)^T$

$$\tilde{y} = \left(\frac{\sqrt{N}}{M} y^T X U \, \Sigma^{-\frac{1}{2}} \right)^T = \frac{\sqrt{N}}{M} \left(y^T X U \, \Sigma^{-\frac{1}{2}} \right)^T$$

$$\tilde{y} = \frac{\sqrt{N}}{M} \left(\Sigma^{-\frac{1}{2}} \right)^T U^T X^T (y^T)^T \rightarrow \tilde{y} = \frac{\sqrt{N}}{M} \Sigma^{-\frac{1}{2}} U^T X^T y$$

Since $\beta > 0$ is imposed, it is mandatory to have a PPIN among TFs so $\tilde{X}$ encodes not only principal components of X but also the PPIN of relations among TFs.

§1.4 Output 1: Identification of novel cell-type TFs for regulating TG

Metrics to evaluate the regression performance of Target Gene (TG)

For each target gene (TG) in a cell-type (and context), we evaluate the performance of NetREm's regression model c^* (used to define our TF-TG regulatory links) in train and test expression data. That is, we compare predicted values $\hat{\tilde{y}} \in \mathbb{R}^{N \times 1}$ to actual embeddings $\tilde{y}$ by relating signal (ground truth $\tilde{y}$) to noise (errors between

$\widehat{\tilde{y}}$ and $\tilde{y}$) in training and testing gene expression data. NetREm can unearth novel, cell-type-specific TFs involved in TG regulation. These N^* regulatory links likely capture truer biological interactions among TFs.

- Mean Square Error (averages the squared differences between predicted and actual expression values for TG): $MSE = \frac{1}{N}\sum_{v=1}^{N}(\widehat{\tilde{y}_v} - \tilde{y})^2 = P_{noise}$
- normalized MSE (help understand the quality or purity of a signal by normalizing the MSE): $NMSE = \frac{P_{noise}}{P_{signal}}$
- signal-to-noise ratio (measures signal strength relative to background noise): $SNR = 10 \times \log_{10}\left(\frac{P_{signal}}{P_{noise}}\right)$
- peak SNR ($PSNR = 10\log_{10}\left(\frac{MAX_I^2}{P_{noise}}\right)$).

Here, $P_{signal} = \frac{1}{N}\sum_{v=1}^{N}\tilde{y}^2$ is the mean squared value of the reference array $\tilde{y}$ and MAX_I^2 is the maximum possible intensity of the signal. Unless otherwise specified MAX_I^2 defaults to $\max|\tilde{y}|$ as the maximum agreed-upon absolute value for the predictions. Generally, ideal regression solutions have low MSE and NMSE values, high SNR and PSNR values.

Incorporating Prior Gene Regulatory Networks to derive TF-RE-TG regulatory networks

We identify regulatory elements (RE) for a given TG. Then, we determine TFs likely to bind directly to or associate indirectly with TF Binding Sites (TFBSs) on these REs. We input N TG-specific candidate TFs to NetREm for TG. Then, we overlay NetREm's N^* TF-TG regulatory links for TG with this prior GRN (initial TF–RE –TG links for N TFs for TG). Then, we can uncover the highly confident final TF-RE-TG links for our final set of N^* TFs for TG. These final TF-RE-TG correspond to the N^* TFs that are selected for regulating the expression of the TG. These TFs may bind directly or indirectly associate with these REs. Thus, the prior GRN links can help annotate our final regulatory links. Please note that this is subjective and is based on steps we detail to obtain a prior GRN.

Mapping Single-Nucleotide Polymorphisms (SNPs) onto NetREm's TF-TG or TF-RE-TG regulatory networks

To annotate/validate these inferred relationships, we may map SNPs on top of these networks. We use motifbreakR (Coetzee et al. 2015), a tool designed to predict the impact of SNPs on TF binding to their binding sites (TFBSs). We use not only strong effects but also weak effects for motifbreakR. We primarily use strong effects for TFs that are predicted to directly associate to the Regulatory Element (RE) to regulate the TG (i.e. TFs from the motif-based GRN that are retained in the pruned TF list); for simplicity, we do not consider the TFs that may indirectly associate with these REs. We use PWMs from motifbreakR as well as our own comprehensive PWMs. We use all 3 methods "default", "ic", "log" for computing SNP-TF effects. In this way, we may predict pathways from non-coding SNPs to disease phenotypes, via TFs with altered TF binding to TFBSs, impacting TF interactions with each other and ultimately leading to changes in expression of the TG.

- When alleleDiff > 0: SNP increases TF binding (boosts its affinity) to its TFBS on regulatory element (RE).
- When alleleDiff < 0: SNP decreases TF binding (disrupts its motif recognition and binding) to its TFBS on RE.

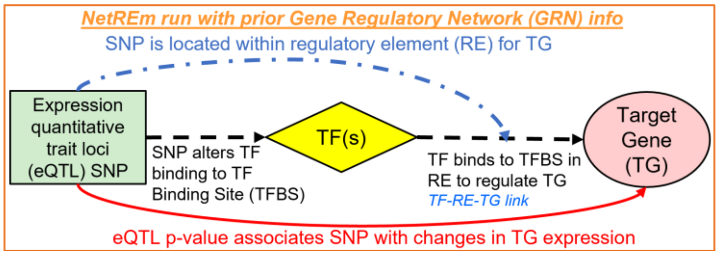

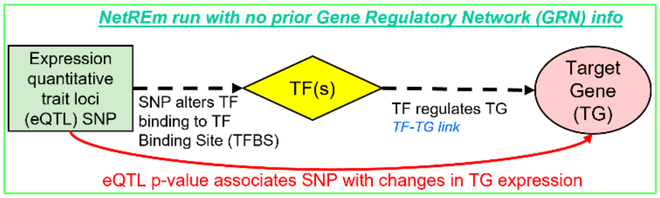

We can then map these SNP-TF links on top of these along with the expression quantitative trait loci (eQTL: eSNP-eTG links) links on these networks. In eQTLs, we have eSNP-eTG that means this expression quantitative trait SNP (eSNP) is linked to expression quantitative trait changes in the TG (eTG).

Left figure: If we use prior (reference) gene regulatory network (GRN) knowledge info, then we may have final TF-RE-TG links from NetREm. We check that the SNP impacts TF binding (either increasing or decreasing TF binding), that this TF binds to a regulatory element (RE) for the TG to regulate the TG (based on final TF-RE-TG links), this SNP falls within this same RE, this SNP associates with changes in TG expression (based on eQTL links).

Right figure: If we do not use any prior gene regulatory network (GRN) knowledge info, then we have final TF-TG links from NetREm. We check the SNP impacts TF binding (either increasing or decreasing TF binding), that this TF regulates

the TG (based on final TF-TG links), this SNP associates with changes in TG expression (based on eQTL links). These networks are not as confident as those from the left figure given that the criteria is not as strong as it is for left figure.

§1.5 Output 2: Cell-type TF-TF coordination (direct/indirect interactions)

Cell-type TF-TF coordination $\bar{B}$:

Functionally coordinated TFs help regulate sets of TGs at a given time point (Nie et al. 2011) through direct and/or indirect interactions with one another. In reality, TF_i and TF_j may only co-regulate a subset of the G TGs in the cell-type, if at all. Nonetheless, their coordination behavior is considered across all G TGs to implicitly adjust for this; that is, the learned cell-type TF-TF coordination score $\bar{B}_{ij}$ will reflect better their coregulatory behavior for the cell-type.

To create a weighted and signed cell-type-specific coordination network among $\mathcal{N}$ TFs, $\bar{B}$, we first aggregate individual B across G TGs. Each TG (i.e. TG$_k$) has expression (y_k), N_k candidate TFs (where $N_k \leq \mathcal{N}$), and coordination score matrix $B^k \in \mathbb{R}^{N_k \times N_k}$, where $k = 1, \dots, G$ and $-100 \leq B^k \leq 100$. Next, to compute aggregate cell-type values from $\{B^k\}_{k=1}^{G}$, we 0 pad $B^k \in \mathbb{R}^{N_k \times N_k}$ to $\hat{B}_k \in \mathbb{R}^{\mathcal{N} \times \mathcal{N}}$ where $\hat{B}_{ij}^k = B_{ij}^k$ if both TF_i and TF_j are N_k^* final TFs (i.e. $c_i^*, c_j^* \neq 0$) selected for TG$_k$ and $\hat{B}_{ij}^k = 0$ otherwise. Then, we compute the mean of these TG-specific undirected TF-TF coordination matrices $\{\hat{B}^k\}_{k=1}^{G}$ as $\mathcal{B}^{(0)} = \frac{\sum_{k=1}^{G} \hat{B}^k}{G}$ where $\mathcal{B}^{(0)} \in \mathbb{R}^{\mathcal{N} \times \mathcal{N}}$. We note there are $\ell_{\mathcal{N}} = 0.5\mathcal{N}(\mathcal{N} - 1)$ unique TF-TF links (i.e. $TF_i - TF_j$ link is the same as $TF_j - TF_i$ and hence counted once). We flatten $\mathcal{B}^{(0)}$ to a vector $\mathcal{B} \in \mathbb{R}^{\ell_{\mathcal{N}}}$ of corresponding values for the corresponding $\ell_{\mathcal{N}}$ unique TF-TF links. For each $TF_i - TF_j$ link, we convert $|\mathcal{B}_{ij}|$ to a percentile $0 \leq P_{ij} \leq 100$, where higher values indicate greater relative coordination among $\ell_{\mathcal{N}}$ links and $P \in \mathbb{R}^{\ell_{\mathcal{N}}}$; this enables a direct comparison of coordination strengths by normalizing $|\mathcal{B}|$'s distribution to a uniform 0 to 100 scale. We sort $|\mathcal{B}|$ in ascending order (small to large) and determine relative position, R_{ij}, of $|\mathcal{B}_{ij}|$ so $P_{ij} = 100\left(\frac{R_{ij}-1}{\ell_{\mathcal{N}}}\right)$% and higher P_{ij} reflects proportionally larger $|\mathcal{B}_{ij}|$. A pair of TFs may exhibit cooperative behavior to regulate some TGs and antagonistic behavior to regulate others. Our goal is to amalgamate learned coordination info across multiple TGs to reach a conclusion about the net nature of cell-type coordination (i.e. signed percentiles), in a manner akin to information content measures. We note that sign($\mathcal{B}$) is 1 if $\mathcal{B}_{ij} > 0$, -1 if $\mathcal{B}_{ij} < 0$, 0 otherwise. This informs the overall net cell-type coordination behavior between TF_i and TF_j.

The final cell-type TF-TF coordination network $\bar{B}$ is defined by: $\bar{B} = \text{sign}(\mathcal{B}) \cdot P$, where $\bar{B}, \mathcal{B}, P \in \mathbb{R}^{\ell_{\mathcal{N}}}$ and -100 $\leq \bar{B} \leq 100$, is the same range as TG-specific coordination scores. Each non-zero score in $\bar{B}$ is unique. $|\overline{B_{ij}}|$ denotes relative strength of $TF_i - TF_j$ coordination and cell-type co-regulation (i.e. normalized interaction significance); the sign encapsulates their net observed behavior across G TGs: $-100 \leq \overline{B_{ij}} < 0$: both TFs are antagonistic overall, $0 < \overline{B_{ij}} \leq 100$: both TFs cooperate overall. TF-TG regulatory networks provide more insights. If $\overline{B_{ij}} \approx 0$ and both TFs co-regulate TGs, we cannot deduce any meaningful info regarding the nature of their cell-type-specific interaction.

Thus, NetREm predicts how TFs coordinate with each other in networks to co-regulate TGs. For each of the G TGs in the cell-type, it outputs a TG-specific TF-TF coordination network: $-100 \leq B \leq 100$, where B_{ij} is the $TF_i - TF_j$ coordination to co-regulate TG. Here, $\bar{B}$ is a function f of $\{B^k\}_{k=1}^{G}$ across G TGs and is an overall cell-type-specific TF-TF coordination network: $-100 \leq \bar{B} \leq 100$, where $\bar{B}_{ij}$ is the net $TF_i - TF_j$ coordination behavior across all G TGs. The G TG-specific coordination networks $\{B^k\}_{k=1}^{G}$ and the overall cell-type coordination network $\bar{B}$ contain scores between -100 and 100, where negative scores suggest potential antagonistic co-regulatory relations and positive scores assign potential cooperative relations among the TFs, via direct and/or indirect mechanisms.

In fact, a recent study (Berenson et al. 2023)found that cooperative TF pairs are supported by strong in vivo evidence of co-binding in ChIP-seq (Chromatin Immunoprecipitation followed by sequencing) experiments. This technique is used to observe where transcription factors bind to DNA across the genome, providing real-world data on TF interactions. These cooperative pairs often consist of one TF that is expressed in many tissues (ubiquitously expressed) and another that is specific to a certain tissue type. This combination allows for fine-tuned regulation of gene expression in particular tissues, leveraging the tissue-specific TF to guide the general activity of the ubiquitously expressed TF. On the other hand, antagonistic pairs usually involve two transcription factors that are both ubiquitously expressed. These pairs tend to work against each other, potentially competing for binding sites on DNA or exerting opposite effects on gene expression. The balance between such antagonistic TFs can be crucial for maintaining proper gene regulation across different cell types and conditions. That previous study highlights the dynamic nature of TF interactions, where cooperative and antagonistic relationships can significantly impact how genes are regulated in different tissues or

conditions. This study suggested that cooperativity and antagonism among TFs to coordinate TG regulation may be mechanisms by which tissue- and cell type-specific TFs modulate the function of more ubiquitous TFs.

- Cell-type TF-TF coordination network $\bar{B}$ for $\mathcal{N}$ cell-type TFs:
  - Run NetREm for each of the G TGs to obtain: $\{B^k\}_{k=1}^{G}$ where $-100 \leq B^k \leq 100$, $B^k \in \mathbb{R}^{N_k \times N_k}$ is TG_k-specific TF-TF coordination where candidate TFs $N_k \leq \mathcal{N}$.
  - Steps to deriving cell-type TF-TF coordination scores $\bar{B}$ from $\{B^k\}_{k=1}^{G}$:

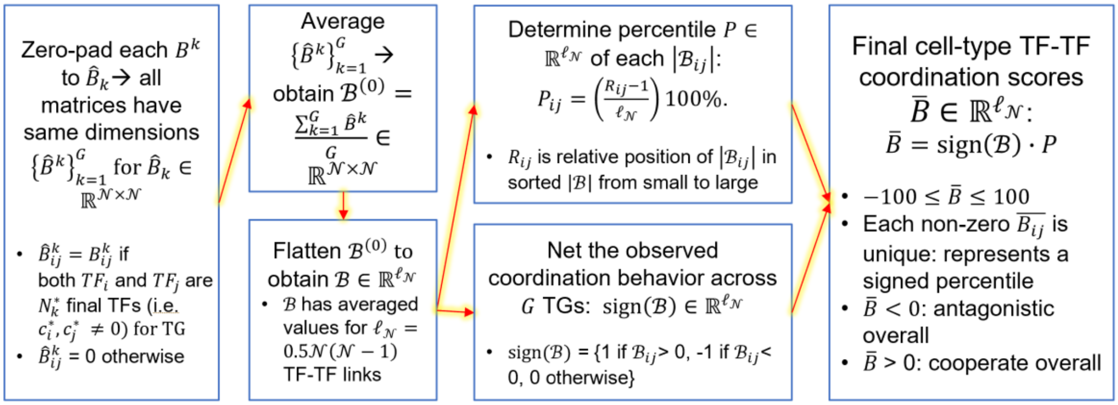

Section §2: Simulation Study:

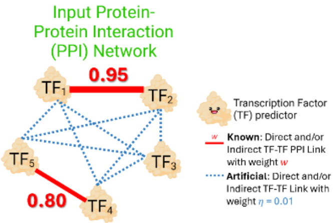

Utilizing prior biological knowledge of network interactions among TF predictors, NetREm can precisely identify and allocate influences of each TF, providing reliable and nuanced insights into individual contributions, despite the presence of existing intercorrelations. We illustrate this capability by running 2 larger simulation analyses in §2.4 and §2.5, each involving 1,000 simulations for the same underlying data. In each simulation, the random seed is changed. Relative pairwise correlations among TFs are nearly identical between training and testing data. For each of the two simulations below, we maintain the original simulated prior protein-protein interaction (PPI) network ($TF_1 - TF_2$ edge weight of 0.8, $TF_4 - TF_5$ edge weight of 0.95, default edge weights of 0.1) and $cor(TF, TG)$ = [0.9, 0.5, 0.4, -0.3, -0.8], as in **Fig. 2A**. We do not fit any y-intercept. Across simulations, our NetREm models continue to standardize X and standardize y. The results of these additional simulation analyses underscore the necessity for careful tuning and adjustments of α and β to help NetREm achieve optimal complexity and dependable predictiveness.

§2.1 Biological Interpretation of Main Simulation in Figure 2:

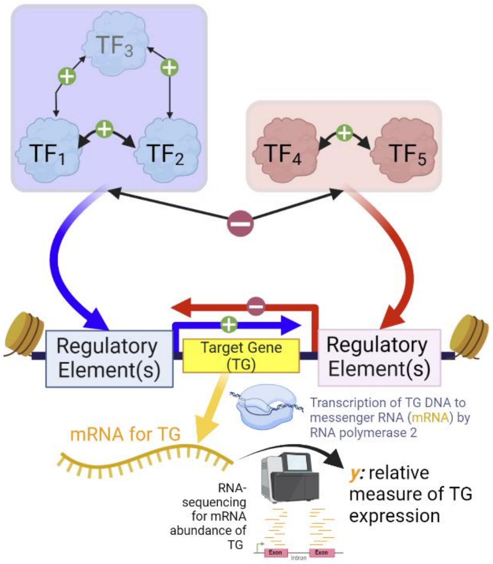

Activators: positive NetREm coefficient $c^* > 0$. Repressors: negative NetREm coefficient $c^* < 0$. Both outputs of NetREm: cell-type TF-TG Regulatory Network (given by coefficients c^* and the TG-specific TF-TF interactions (given by B values) for the cell-type to illustrate how both can help determine TF-TF behaviors involved in gene regulation of TG. Please note that this figure on the left of the following page shows how we may interpret NetREm's main results from **Figure 2**. It is an illustration of cell-type-specific TF-TF coordination interactions that are involved in gene regulation of TG with relative gene expression y.

The TF-TG regulatory network shows that TF_1, TF_2, and TF_3 are activators of TG (work to increase TG expression) since they have positive coefficients: $c^* > 0$. Given their negative coefficients for regulating TG (i.e. $c^* < 0$), we assign TF_4 and TF_5 as repressors of TG (work to decrease TG expression levels y).
The final NetREm model predicted the main actions of TFs 1, 2, 4, and 5 in gene regulation of target gene TG. TF_3 is weakly involved. It is likely that from the B matrix of TF-TF interactions we may predict: Cooperative behavior (+): TF1 and TF2 (and weakly with TF3); TF4 and TF5. That is, cooperative behavior ($B > 0$) is among co-activators and co-repressors since they have the same goals for TG: co-activators want to increase transcription and subsequent expression of TG; co-repressors want to prevent or reduce transcription of TG to messenger RNA (mRNA). Antagonistic behavior ($B < 0$): TF_1 and TF_4; TF_1 and TF_5; TF_2 and TF_4; TF_2 and TF_5; TF_3 and TF_4; TF_3 and TF_5. NetREm's model predicts that there is antagonism between an activator and repressor because both have opposing behavior to regulate TG. We see there are these activator modules and repressor modules and these form transcriptional regulatory modules (TRMs) together. While the figure shows that they may bind to different Regulatory Elements (REs), they can all bind to the same ones as well. Nonetheless, NetREm can be applied to many applications where an inherent network structure exists among the predictors (e.g. TFs) to influence the response variable y (the expression levels of our TG that may be regulated by some of the N candidate TFs). The figure on the right shows how cell-type TF-TF coordination networks $\bar{B}$ may differ. Different cell-type TFs may interact with each other differently across cell-types to regulate the TG.

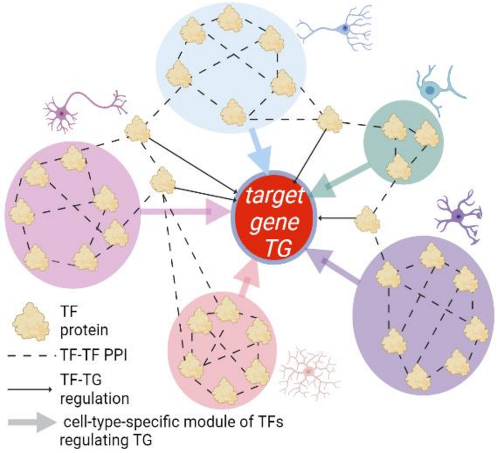

§2.2 Simulation with various sparsity levels:

To tackle the issue of gene expression sparsity, notably pertinent to transcription factor expression in single-cell analyses, we performed a simulation study with $M = 100$ and $N = 500$ across different sparsity levels (0.70, 0.85, 0.90). In this context, 70%, 85%, and 90% of the gene expressions are zeros, respectively. We evaluated the method's performance using mean squared error (MSE) and noted an improvement in performance with increasing sparsity levels, as outlined in the table below that affirms our method's adeptness at managing data sparsity effectively:

Sparsity	0.70	0.85	0.90
MSE	0.0239	0.0183	0.00903

§2.3 Simulation with various M and N

In our manuscript, we discuss enhancing stability by setting small singular values (e.g., $\sigma_i < 10^{-6}\sigma_{max}$) to zero, resulting a truncated Σ_{trunc}. Similarly, we adjust the inverse $\Sigma_{trunc}{}^{-1}$ so that the inverse elements corresponding to small singular values are set to zero. To illustrate the necessity of this approach, we simulated various cases where $M < N, M = N$, and $M > N$, computing the conditional numbers of $E = \frac{X^T X}{M} + \beta A$ for various values of β as shown in the table below:

β	M	N	Conditional Number $\boldsymbol{\kappa(E)}$
10^{-6}	100	500	1.39×10^{7}
10^{-6}	300	300	6.28×10^{6}
10^{-6}	500	100	3.94×10^{6}
1	100	500	1.83
1	300	300	8.84
1	500	100	7.32
10^{6}	100	500	1.14×10^{6}
10^{6}	300	300	1.56×10^{6}
10^{6}	500	100	1.69×10^{6}

This method demonstrates how the conditional numbers vary across different configurations, revealing that the condition number can be significantly large ($> 10^6$), thus showing the necessity of employing truncated SVD. The analysis also shows that when β is small, $E = \frac{X^TX}{M} + \beta A$ primarily depends on the data term $\frac{X^TX}{M}$, where the conditional number decreases as the number of measurements increases (from $M < N$ to $M > N$). When β is large, the conditional number $E = \frac{X^TX}{M} + \beta A$ mainly depends on the network prior term βA which is determined by the PPIN and is independent of M.

§2.4 Testing Robustness

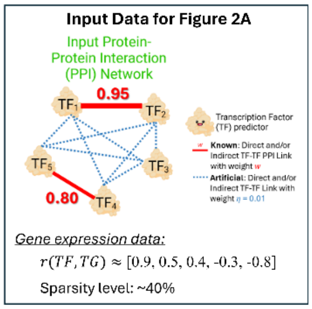

This analysis tests the robustness of NetREm (measured by the standard deviation) compared with that of the 4 benchmark regression models (LassoCV, LinearRegression, ElasticNetCV, RidgeCV). We use the same setup from **Figure 2A**: pairwise correlations of TF predictors with TG expression y, sparsity levels, and same underlying input PPI Network. All 4 models are fit with defaults and no y-intercept. We run benchmarks and NetREm (with LassoCV) on 1,000 simulations of the respective random generated data (changing random seed); this setup allows for variations in pairwise correlations among TFs in each simulation. Please note that for each pairwise Welch test, we use the R software t.test function default of var.equal = False. We compare the coefficients predicted by NetREm for each of the 5 predictors with those predicted by each of the 4 respective benchmark regression models (BRMs: RidgeCV, Linear Regression, ElasticNetCV, LassoCV) where we test the following alternative hypotheses (H_A) based on the sample means $\widehat{\mu_{TF}}$ for each respective TF predictor (assuming that the Null Hypothesis H_o asserts that there is NO statistically significant difference in sample mean values for the coefficients for that given TF between NetREm and that benchmark model): for i = 1 to 5

$$H_A \text{ for } TF_i\text{: } \mu_{\widehat{Netrem,\ TF_i}} > \mu_{\widehat{Benchmark,\ TF_i}}$$

We guide these 1-sided t-tests (at the 5% level: p-value < 0.05 to reject H_o for statistical significance) based on what we strive to illustrate in this demo (i.e. indicated by change in signs < or > for H_A). The results are in **Table S1**. Our results are statistically significant for TFs 2 to 4 compared to BRMs. Comparative robustness checks across 1,000 simulations show NetREm has less variability and more stability for all c^* (**Table S2, Fig. S2A**).

§2.5 Benchmarking β

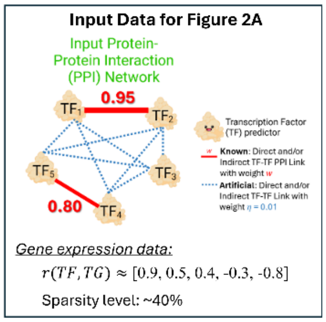

The 2nd analysis focuses on the impact of network-constrained prior hyperparameter β on NetREm coefficients, standard deviation, and test performance (measured by the mean square error (MSE)). We use the same setup from **Figure 2A**: pairwise correlations of TF predictors with TG expression y, sparsity levels, and same underlying input PPI Network. Here, we benchmark β by re-running 1,000 simulations to investigate the impacts of altering β (across 7 test values: 0.1, 0.5, 1, 5, 10, 50, 100) on NetREm's performance, holding all else constant; thus, we fix α at α^* and use Lasso; we compare NetREm models with one another based on varying the network constrained hyperparameter β and holding the lasso hyperparameter term α constant at $\alpha = 9e - 4$. This procedure elucidates NetREm's adeptness at managing variance (e.g. std) by incorporating a manageable level of bias (MSE), which is key for optimizing the bias-variance tradeoff, preventing overfitting, enhancing generalization to unseen data; nonetheless, there is a critical point beyond which excessive network regularization penalty can be detrimental,

leading to models overly constrained by the predefined network structure. We illustrate how bias (e.g. test MSE) increases monotonically as β increases: 0.1, 0.25, 0.5, 0.75, 1, 2.5, 5, 7.5, 10, 25, 50, 75, 100. Our network regularization term scaled by β can aid with managing variance (e.g. standard deviation (std) of coefficients based on changes in data) of our model, even if it introduces some level of bias. This key adjustment helps models generalize better to unseen data by preventing overfitting, a situation often characterized by low bias but high variance. This procedure elucidates NetREm's adeptness at managing variance by incorporating a manageable level of bias, which is key for optimizing the bias-variance tradeoff, preventing overfitting, enhancing generalization to unseen data. Coefficients for TF_2 (0.101 to 0.283) and TF_4 (-0.037 to –0.107) increase in magnitude and standard deviation of all 5 coefficients decrease as β increases to 5. In any case, β = 7.5 is still alright in terms of standard deviation values. However, there is a critical point beyond which excessive network regularization penalty can be detrimental, leading to models overly constrained by the predefined network structure. Elevated β values, specifically at 50, 75, and 100, cause NetREm to struggle as it strictly adheres to network relations, leading to increased bias and variance. This deteriorates not only predictive performance but also variance, observed by suboptimal coefficients, increased std. Thus, meticulously tuning both α and β can secure a balanced model complexity and maintain robust, reliable predictive performance. 1,000 simulations for 13 β values (**Table S3, Fig. S2B-C**), illustrate bias-variance trade-offs where excess β over-constrains models, causing NetREm to struggle and predictions to suffer.

Section §3: Introducing the 12 main applications of NetREm

§3.1 Diving into the 12 main applications of NetREm

We apply NetREm to 12 different real-world applications, where an application refers to a unique data resource from a given study. That is, we apply NetREm to single-cell gene expression data spanning 12 different studies. Some of these applications include multiple cell-types.

Application (#)	Cell-type	Organism
NetREm with no prior GRN knowledge input: all TGs have same set of $\mathcal{N}$ potential cell-type TFs		
Benchmarking (Gold Standard GRN is available)	6 Simulated Embryonic Stem Cells (hESCs) datasets (#1)	Humans
	Hematopoietic Stem Cells (HSCs) (#2)	
	Human Pluripotent Stem Cells (hPSCs) (#5)	
	Mouse (mESCs) ESCs (#3)	Mice
	Mouse Dendritic Cells (mDCs) (#4)	
	Budding Yeast Cells (YCs) (#6)	Yeast
Benchmarking	9 Peripheral Blood Mononuclear cell (PBMC) types (#7)	Humans
	Pooled Schwann cells from 5 human tissues (#8)	
Benchmarking 4 Central Nervous System (CNS) cell types (#9)	Excitatory Neurons (ExNs)	
	Inhibitory Neurons (InNs)	
	Oligodendrocytes (Oligo)	
	Microglia (Mic)	
Benchmarking TG-specific TF-TF coordination links (#10)	62 different immune cell subtypes available from the Tabula Sapiens Atlas. Filtered for only 15 TGs and 125 TFs with available coordination experimental results by another study.	
NetREm with prior GRN knowledge input: initial TF-regulatory element-TG links: each TG has a customized list of $N \leq \mathcal{N}$ TFs		
Human Schwann Cells (SCs) (#11)	Myelinating SCs: mSCs	5 human donors
	Non-myelinating SCs: nmSCs	
Alzheimer's Disease (AD) versus Control Stage (C) in Humans (#12)	Astrocytes	AD: 24 humans; C: 24 humans
	Endothelial Blood Brain Barrier (Endo BBB)	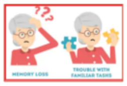
	Excitatory Neurons	
	Inhibitory Neurons	
	Microglia	
	Oligodendrocytes (Oligo)	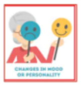
	Oligo Progenitor Cells (OPCs)	
	Pericytes	

Please note that applications 1 to 10 span humans, mice, and yeast, and are used for benchmarking NetREm in the absence of prior gene regulatory network (GRN) knowledge. For these 10 applications, the NetREm pipeline is therefore run for the same set of potential N candidate TFs for each TG, where $N = \mathcal{N}$. Here, $\mathcal{N}$ is the set of potential cell-type TFs. If the TG is one of the candidate TFs, we remove that TF for the TG and then run NetREm with $N = \mathcal{N} - 1$ TFs.

In applications 11 and 12, we apply NetREm in real-world scenarios in the presence of prior information, namely prior GRN knowledge. We obtain this prior GRN knowledge by integrating various multiomics data to link candidate TFs to regulatory elements (e.g. promoters, enhancers) they may bind to or associate with to regulate their potential TG. Ultimately, we have a custom, personalized list of $N \leq \mathcal{N}$ candidate TFs for each TG, where N may differ across TGs.

Here, β is the network-constrained hyperparameter and comes beforehand and is used to define network regression gene embeddings $\tilde{X}$ and $\tilde{y}$; α is the sparsity prior hyperparameter (and optimizes prediction of $\tilde{y}$ from $\tilde{X}$). Thus, we often select β first and then may use LassoCV solvers to optimize α based on $\tilde{X}$ and $\tilde{y}$.

Please note that for all our applications (i.e. 1 to 12), we use our preprocessed respective TF-TF PPI network (PPIN) with positive weights as a network regression constraint; we do not fit a y-intercept for our NetREm or our baseline models. We run NetREm each time (1 by 1) for each target gene (TG) in the cell-type (and/or context) to yield TG-specific TF-TF coordination network B and N^* final TFs for the TG (where N^* can vary across TGs even for fixed N candidate TFs since N^* TFs are those with $c^* \neq 0$). We then aggregate our results to yield our cell-type TF-TG regulatory network (comprising individual TF-TG links) and our cell-type TF-TF coordination network $\bar{B}$.

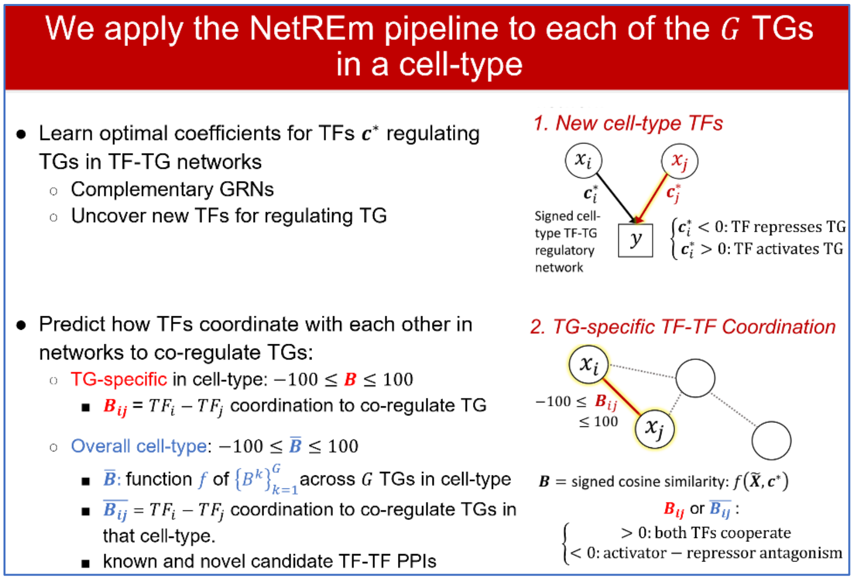

§3.2 Example outputs by NetREm for a case-control (treatment versus control application)

For instance, suppose there are gene expression datasets of treatment versus controls for a given condition in a context (e.g. specific cell-type or tissue-type). We can apply NetREm to build 2 context-specific TF-TG regulatory networks (coefficients c^*), 2 overall context-specific TF-TF coordination networks $\bar{B}$, and individual TF-TF coordination networks for the given TGs B.

That is, for each TG in a given context and situation (treatment or control), NetREm will predict TF-TF coordination for regulating that TG. We can stitch together those predictions:

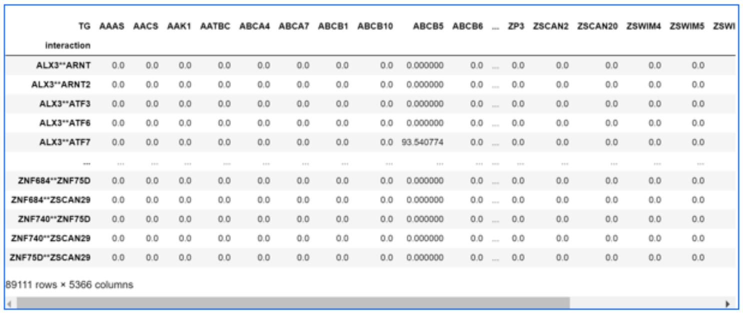

TG / interaction	AAAS	AACS	AAK1	AATBC	ABCA4	ABCA7	ABCB1	ABCB10	ABCB5	ABCB6	...	ZP3	ZSCAN2	ZSCAN20	ZSWIM4	ZSWIM5	ZSWI
ALX3**ARNT	0.0	0.0	0.0	0.0	0.0	0.0	0.0	0.0	0.000000	0.0	...	0.0	0.0	0.0	0.0	0.0	
ALX3**ARNT2	0.0	0.0	0.0	0.0	0.0	0.0	0.0	0.0	0.000000	0.0	...	0.0	0.0	0.0	0.0	0.0	
ALX3**ATF3	0.0	0.0	0.0	0.0	0.0	0.0	0.0	0.0	0.000000	0.0	...	0.0	0.0	0.0	0.0	0.0	
ALX3**ATF6	0.0	0.0	0.0	0.0	0.0	0.0	0.0	0.0	0.000000	0.0	...	0.0	0.0	0.0	0.0	0.0	
ALX3**ATF7	0.0	0.0	0.0	0.0	0.0	0.0	0.0	0.0	93.540774	0.0	...	0.0	0.0	0.0	0.0	0.0	
...	...	...	...	...	...	...	...	...	...	...	...	...	...	...	...	...	
ZNF684**ZNF75D	0.0	0.0	0.0	0.0	0.0	0.0	0.0	0.0	0.000000	0.0	...	0.0	0.0	0.0	0.0	0.0	
ZNF684**ZSCAN29	0.0	0.0	0.0	0.0	0.0	0.0	0.0	0.0	0.000000	0.0	...	0.0	0.0	0.0	0.0	0.0	
ZNF740**ZNF75D	0.0	0.0	0.0	0.0	0.0	0.0	0.0	0.0	0.000000	0.0	...	0.0	0.0	0.0	0.0	0.0	
ZNF740**ZSCAN29	0.0	0.0	0.0	0.0	0.0	0.0	0.0	0.0	0.000000	0.0	...	0.0	0.0	0.0	0.0	0.0	
ZNF75D**ZSCAN29	0.0	0.0	0.0	0.0	0.0	0.0	0.0	0.0	0.000000	0.0	...	0.0	0.0	0.0	0.0	0.0	

89111 rows × 5366 columns

- TG-specific TF-TF coordination in Control samples

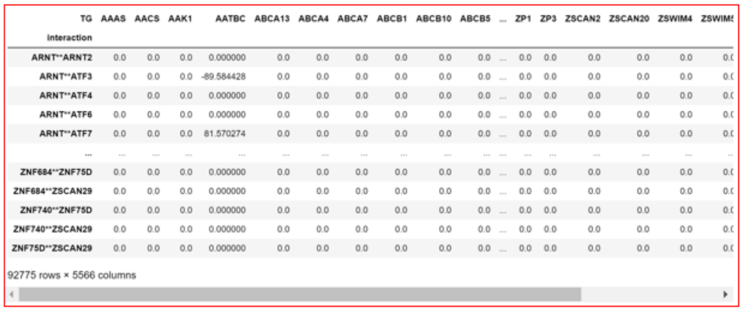

TG / interaction	AAAS	AACS	AAK1	AATBC	ABCA13	ABCA4	ABCA7	ABCB1	ABCB10	ABCB5	...	ZP1	ZP3	ZSCAN2	ZSCAN20	ZSWIM4	ZSWIM[illegible]
ARNT**ARNT2	0.0	0.0	0.0	0.000000	0.0	0.0	0.0	0.0	0.0	0.0	...	0.0	0.0	0.0	0.0	0.0	0.[illegible]
ARNT**ATF3	0.0	0.0	0.0	-89.584428	0.0	0.0	0.0	0.0	0.0	0.0	...	0.0	0.0	0.0	0.0	0.0	0.[illegible]
ARNT**ATF4	0.0	0.0	0.0	0.000000	0.0	0.0	0.0	0.0	0.0	0.0	...	0.0	0.0	0.0	0.0	0.0	0.[illegible]
ARNT**ATF6	0.0	0.0	0.0	0.000000	0.0	0.0	0.0	0.0	0.0	0.0	...	0.0	0.0	0.0	0.0	0.0	0.[illegible]
ARNT**ATF7	0.0	0.0	0.0	81.570274	0.0	0.0	0.0	0.0	0.0	0.0	...	0.0	0.0	0.0	0.0	0.0	0.[illegible]
...	...	...	...	...	...	...	...	...	...	...	...	...	...	...	...	...	...
ZNF684**ZNF75D	0.0	0.0	0.0	0.000000	0.0	0.0	0.0	0.0	0.0	0.0	...	0.0	0.0	0.0	0.0	0.0	0.[illegible]
ZNF684**ZSCAN29	0.0	0.0	0.0	0.000000	0.0	0.0	0.0	0.0	0.0	0.0	...	0.0	0.0	0.0	0.0	0.0	0.[illegible]
ZNF740**ZNF75D	0.0	0.0	0.0	0.000000	0.0	0.0	0.0	0.0	0.0	0.0	...	0.0	0.0	0.0	0.0	0.0	0.[illegible]
ZNF740**ZSCAN29	0.0	0.0	0.0	0.000000	0.0	0.0	0.0	0.0	0.0	0.0	...	0.0	0.0	0.0	0.0	0.0	0.[illegible]
ZNF75D**ZSCAN29	0.0	0.0	0.0	0.000000	0.0	0.0	0.0	0.0	0.0	0.0	...	0.0	0.0	0.0	0.0	0.0	0.[illegible]

92775 rows × 5566 columns

- TG-specific TF-TF coordination in Treatment samples

We note how for the control situation we have individual TF-TF coordination networks for each of the final 5,366 TGs (i.e. 5,366 respective TG-specific TF-TF coordination networks B) and how for the treatment we have 5,566 respective TG-specific TF-TF coordination networks B.

These individual networks can be stitched together and aggregated to yield 2 final respective networks: overall control-specific context TF-TF coordination network $\bar{B}$ and overall treatment-specific context TF-TF coordination network $\bar{B}$.

treated

	A	B	C	D	E	F	G	H	I	J	K	L	M	N
1	interaction	TF1	TF2	Final # TGs	# of TGs with TF-TF coordination	# of nonzero links	condition	Final Coordination Score	Net Behavior	Cooperative TGs	Antagonistic TGs	Total Final Co-Regulated TGs	Direct and/or indirect PPI score	Known PPI Link
2	RXRA**JUNB	RXRA	JUNB	7,334	5,564	92,775	treated	100.000	Cooperative	2,052	0	2,052	0.166166166	yes
3	RXRA**JUND	RXRA	JUND	7,334	5,564	92,775	treated	99.999	Cooperative	2,012	0	2,012	0.309309309	yes
4	JUND**JUNB	JUND	JUNB	7,334	5,564	92,775	treated	99.998	Cooperative	1,928	0	1,928	1	yes
5	RXRA**STAT1	RXRA	STAT1	7,334	5,564	92,775	treated	99.997	Cooperative	1,778	0	1,778	1	yes
6	RARA**RXRA	RARA	RXRA	7,334	5,564	92,775	treated	99.996	Cooperative	1,727	0	1,727	1	yes
7	VDR**RXRA	VDR	RXRA	7,334	5,564	92,775	treated	99.995	Cooperative	1,657	0	1,657	1	yes
8	JUNB**STAT1	JUNB	STAT1	7,334	5,564	92,775	treated	99.994	Cooperative	1,634	0	1,634	1	yes
9	SREBF1**FOS	SREBF1	FOS	7,334	5,564	92,775	treated	99.992	Cooperative	1,614	0	1,614	0.422422422	yes
10	STAT1**JUND	STAT1	JUND	7,334	5,564	92,775	treated	99.991	Cooperative	1,613	0	1,613	1	yes
11	RARA**JUNB	RARA	JUNB	7,334	5,564	92,775	treated	99.990	Cooperative	1,595	0	1,595	0.249249249	yes
12	E2F6**RXRA	E2F6	RXRA	7,334	5,564	92,775	treated	-99.989	Antagonistic	0	1,549	1,549	0.16016016	yes
13	JUND**RARA	JUND	RARA	7,334	5,564	92,775	treated	99.988	Cooperative	1,528	0	1,528	0.336336336	yes
14	RARA**ETS1	RARA	ETS1	7,334	5,564	92,775	treated	99.987	Cooperative	1,470	0	1,470	0.307307307	yes
15	JUNB**E2F6	JUNB	E2F6	7,334	5,564	92,775	treated	-99.986	Antagonistic	0	1,441	1,441	0	no
16	E2F6**RARA	E2F6	RARA	7,334	5,564	92,775	treated	-99.985	Antagonistic	0	1,370	1,370	1	yes
17	IRF1**RXRA	IRF1	RXRA	7,334	5,564	92,775	treated	99.984	Cooperative	1,389	0	1,389	0.255255255	yes
18	JUND**E2F6	JUND	E2F6	7,334	5,564	92,775	treated	-99.983	Antagonistic	0	1,376	1,376	0.364364364	yes
19	VDR**JUNB	VDR	JUNB	7,334	5,564	92,775	treated	99.982	Cooperative	1,415	0	1,415	1	yes
20	VDR**JUND	VDR	JUND	7,334	5,564	92,775	treated	99.981	Cooperative	1,371	0	1,371	0	no
21	RXRA**MAF	RXRA	MAF	7,334	5,564	92,775	treated	99.980	Cooperative	1,306	0	1,306	0.326326326	yes
22	STAT1**RARA	STAT1	RARA	7,334	5,564	92,775	treated	99.978	Cooperative	1,353	0	1,353	1	yes
23	IRF1**JUNB	IRF1	JUNB	7,334	5,564	92,775	treated	99.977	Cooperative	1,298	0	1,298	0.441441441	yes
24	JUND**IRF1	JUND	IRF1	7,334	5,564	92,775	treated	99.976	Cooperative	1,293	0	1,293	0.424424424	yes
25	GATA2**FOSL2	GATA2	FOSL2	7,334	5,564	92,775	treated	99.975	Cooperative	1,292	0	1,292	0.329329329	yes
26	RXRA**ETS1	RXRA	ETS1	7,334	5,564	92,775	treated	99.974	Cooperative	1,321	0	1,321	0.254254254	yes
27	RXRA**POU3F3	RXRA	POU3F3	7,334	5,564	92,775	treated	99.973	Cooperative	1,265	0	1,265	0	no
28	MZF1**ISX	MZF1	ISX	7,334	5,564	92,775	treated	99.972	Cooperative	1,267	0	1,267	0	no
29	STAT1**VDR	STAT1	VDR	7,334	5,564	92,775	treated	99.971	Cooperative	1,273	0	1,273	1	yes
30	STAT1**E2F6	STAT1	E2F6	7,334	5,564	92,775	treated	-99.970	Antagonistic	0	1,244	1,244	0.402402402	yes
31	MAF**JUNB	MAF	JUNB	7,334	5,564	92,775	treated	99.969	Cooperative	1,263	0	1,263	0.741741742	yes

controls

	A	B	C	D	E	F	G	H	I	J	K	L	M	N
1	interaction	TF1	TF2	Final # TGs	# of TGs with TF-TF coordination	# of nonzero links	condition	Final Coordinati on Score	Net Behavior	Cooperative TGs	Antagonistic TGs	Total Final Co-Regulated TGs	Direct and/or indirect PPI score	Known PPI Link
2	JUND**RXRA	JUND	RXRA	7,075	5,362	89,110	control	100.000	Cooperative	1,827	0	1,827	0.30930931	yes
3	RXRA**JUND	RXRA	JUND	7,075	5,362	89,110	control	100.000	Cooperative	1,827	0	1,827	0.30930931	yes
4	RXRA**ELF3	RXRA	ELF3	7,075	5,362	89,110	control	99.999	Cooperative	1,588	0	1,588	0.23423423	yes
5	ELF3**RXRA	ELF3	RXRA	7,075	5,362	89,110	control	99.999	Cooperative	1,588	0	1,588	0.23423423	yes
6	RARA**RXRA	RARA	RXRA	7,075	5,362	89,110	control	99.998	Cooperative	1,587	0	1,587	1	yes
7	RXRA**RARA	RXRA	RARA	7,075	5,362	89,110	control	99.998	Cooperative	1,587	0	1,587	1	yes
8	RXRA**FOSL1	RXRA	FOSL1	7,075	5,362	89,110	control	-99.997	Antagonistic	0	1,481	1,481	0.1971972	yes
9	FOSL1**RXRA	FOSL1	RXRA	7,075	5,362	89,110	control	-99.997	Antagonistic	0	1,481	1,481	0.1971972	yes
10	RXRA**STAT1	RXRA	STAT1	7,075	5,362	89,110	control	-99.996	Antagonistic	0	1,389	1,389	1	yes
11	STAT1**RXRA	STAT1	RXRA	7,075	5,362	89,110	control	-99.996	Antagonistic	0	1,389	1,389	1	yes
12	JUN**FOSL2	JUN	FOSL2	7,075	5,362	89,110	control	99.994	Cooperative	1,383	0	1,383	1	yes
13	FOSL2**JUN	FOSL2	JUN	7,075	5,362	89,110	control	99.994	Cooperative	1,383	0	1,383	1	yes
14	JUND**FOSL1	JUND	FOSL1	7,075	5,362	89,110	control	-99.993	Antagonistic	0	1,307	1,307	1	yes
15	FOSL1**JUND	FOSL1	JUND	7,075	5,362	89,110	control	-99.993	Antagonistic	0	1,307	1,307	1	yes
16	NFYB**SREBF1	NFYB	SREBF1	7,075	5,362	89,110	control	-99.992	Antagonistic	0	1,305	1,305	0.56856857	yes
17	SREBF1**NFYB	SREBF1	NFYB	7,075	5,362	89,110	control	-99.992	Antagonistic	0	1,305	1,305	0.56856857	yes
18	ELF3**JUND	ELF3	JUND	7,075	5,362	89,110	control	99.991	Cooperative	1,346	0	1,346	0.26626627	yes
19	JUND**ELF3	JUND	ELF3	7,075	5,362	89,110	control	99.991	Cooperative	1,346	0	1,346	0.26626627	yes
20	STAT1**RARA	STAT1	RARA	7,075	5,362	89,110	control	-99.990	Antagonistic	0	1,304	1,304	1	yes
21	RARA**STAT1	RARA	STAT1	7,075	5,362	89,110	control	-99.990	Antagonistic	0	1,304	1,304	1	yes
22	SREBF1**RARA	SREBF1	RARA	7,075	5,362	89,110	control	99.989	Cooperative	1,366	0	1,366	1	yes
23	RARA**SREBF1	RARA	SREBF1	7,075	5,362	89,110	control	99.989	Cooperative	1,366	0	1,366	1	yes
24	RARA**ELF3	RARA	ELF3	7,075	5,362	89,110	control	99.988	Cooperative	1,315	0	1,315	0.17117117	yes
25	ELF3**RARA	ELF3	RARA	7,075	5,362	89,110	control	99.988	Cooperative	1,315	0	1,315	0.17117117	yes
26	JUND**RARA	JUND	RARA	7,075	5,362	89,110	control	99.987	Cooperative	1,312	0	1,312	0.33633634	yes
27	RARA**JUND	RARA	JUND	7,075	5,362	89,110	control	99.987	Cooperative	1,312	0	1,312	0.33633634	yes
28	GATA2**JUNB	GATA2	JUNB	7,075	5,362	89,110	control	99.985	Cooperative	1,284	0	1,284	0.57857858	yes
29	JUNB**GATA2	JUNB	GATA2	7,075	5,362	89,110	control	99.985	Cooperative	1,284	0	1,284	0.57857858	yes
30	SREBF1**ETS1	SREBF1	ETS1	7,075	5,362	89,110	control	-99.984	Antagonistic	0	1,248	1,248	0.17917918	yes
31	ETS1**SREBF1	ETS1	SREBF1	7,075	5,362	89,110	control	-99.984	Antagonistic	0	1,248	1,248	0.17917918	yes
32	JUN**EGR3	JUN	EGR3	7,075	5,362	89,110	control	-99.983	Antagonistic	0	1,222	1,222	0.5965966	yes
33	EGR3**JUN	EGR3	JUN	7,075	5,362	89,110	control	-99.983	Antagonistic	0	1,222	1,222	0.5965966	yes
34	JUN**NR6A1	JUN	NR6A1	7,075	5,362	89,110	control	-99.982	Antagonistic	0	1,268	1,268	0	no
35	NR6A1**JUN	NR6A1	JUN	7,075	5,362	89,110	control	-99.982	Antagonistic	0	1,268	1,268	0	no
36	MZF1**IRF9	MZF1	IRF9	7,075	5,362	89,110	control	99.981	Cooperative	1,241	0	1,241	0	no
37	IRF9**MZF1	IRF9	MZF1	7,075	5,362	89,110	control	99.981	Cooperative	1,241	0	1,241	0	no
38	JUND**STAT1	JUND	STAT1	7,075	5,362	89,110	control	-99.980	Antagonistic	0	1,193	1,193	1	yes
39	STAT1**JUND	STAT1	JUND	7,075	5,362	89,110	control	-99.980	Antagonistic	0	1,193	1,193	1	yes

Section §4: Single-Cell Gene Expression Data for 11 Main Applications (Apps)

By default, NetREm reserves 30% of samples for testing and utilizes the remaining 70% for training. This is a partitioning used by other methods(Jin et al. 2021). We use this partitioning for applications 1, 10, and 11; thus, so the # of samples: M = # of train cells. For the remaining applications, we use all the gene expression data for training and for testing, so M = # of original cells in the final pre-processed data. We normalize and scale data with Seurat in R (Satija et. al 2015). In applications 4-6, we use (McCalla et al. 2023) for the normalized preprocessed gene expression data on the original data. While dataset §4.1 is simulated, the remaining datasets are from real-world applications and studies.

§4.1: human Embryonic Stem Cells (hESCs): simulated

We obtain a weighted and signed (+: activates; -: represses) atlas of ground truth TF-TG regulatory information in hESCs, originating from TF induction analysis on gene expression and from ChIP-seq studies (Sharov et al. 2022). We randomly select 1,250 TGs and their TFs from the ground truth GRN, which results in $N^* = 207$ TFs (15 that are also TGs, 192 that are master regulators) and 5,050 signed TF-TG links that we input to the state-of-the-art SERGIO (Single-cell ExpRession of Genes In silicO) tool. SERGIO (Dibaeinia and Sinha 2020; Dibaeinia 2024) then simulates realistic single-cell gene expression data for 1,000 cells (training: M = 700, testing: 300) and 1,442 genes based on this underlying, true GRN subset. We vary SERGIO's noise parameter (30%, 60%, 90%) and ultimately retrieve 3 different synthetic single-cell gene expression datasets for hESCs. We repeat this for 100 cells (M = 70).

§4.2: human Hematopoietic Stem Cells (HSCs)

We utilize Seurat (Satija et. al 2015). to normalize and scale single-cell gene expression data for 12,558 genes in HSCs (Buenrostro et al. 2018). We remove genes not expressed in at least 3 cells in the total data, resulting in 12,223 genes for 2,268 cells in HSCs. We focus on running NetREm for TGs that with prior ground truth GRN validation data (Zhang et al. 2023). Hence, the final data has 10,588 genes for M = 2,268 cells in HSCs. We use all data for training and for testing. We also note there are $\mathcal{N}$ = 178 TFs found in both the ground truth GRN and the gene expression data, which are used as the candidate TFs for each TG. For each TG, N = 177 (if the TG is also a TF) or 178 TFs (if the TG is not a TF).

§4.3: mouse Embryonic Stem Cells (mESCs)

We use data (GSE108222) from a study (Tran et. al 2019) that reprograms mouse embryonic fibroblasts (MEFs) to an embryonic-like induced pluripotent stem cell (iPSC) state (i.e. "turning back the clock" on the cellular identity) by reversing cellular differentiation end epigenetic markers of aging. Since they pass all pluripotency tests, iPSCs have a pluripotent state functionally equivalent to that of mESCs. We follow Zhang et. al (Zhang et al. 2023) and concatenate the expression of 2 mice replicates at each time point. We use Seurat (Satija et. al 2015) to normalize and scale the gene expression data. Then, we filter the 24,421 genes to include only TGs and TFs found in ground truth data (Zhang et al. 2023). Our final data has 19,225 genes for M = 1,080 cells in mESCs. We also note there are $\mathcal{N}$ = 195 TFs found in both the ground truth GRN and the gene expression data, which are used as the candidate TFs for each TG. Final data has 19,225 genes for M = 1,080 cells. N = 194-195 candidate TFs for each TG.

§4.4: mouse Dendritic Cells (mDCs)

We use normalized data (McCalla et al. 2023) originating from study GSE48968 (Shalek et. al 2014) on single-cell RNA-seq libraries for over 1,700 primary DCs derived from bone marrow in mice. Ultimately, we find $\mathcal{N}$ = 93 TFs in both ground truth GRN and expression data, which are candidate TFs for each of the 9,087 TGs. This expression data has M = 1,211 cells. N = 92-93 candidate TFs for each of 9,087 TGs for M = 1,211 cells.

§4.5: human pluripotent stem cells (hPSCs)

We use normalized data (McCalla et al. 2023) originating from study GSE107552 (Han et al. 2018), which consists of high-throughput single-cell RNA sequencing (scRNA-seq) data mapping early differentiation pathways of hPSCs. As hPSCs have the unique ability to differentiate into nearly any cell type, this dataset captures a comprehensive cellular-state landscape, spanning lineages like neural, muscle, endothelial, stromal, liver, and epithelial cells. The gene expression profiles and developmental trajectories provided by this dataset are crucial for understanding the processes of early human development, enabling deeper insights into cellular-state transitions and the regulation of lineage specification—important for applications in stem cell research and regenerative medicine. We filter this single-cell expression data for genes that are in the ground truth GRN. N = 169-170 candidate TFs for each of 7,464 TGs for M = 5,520 cells.

§4.6: budding yeast cells (YCs)

Yeast is a single-celled fungus that belongs to the eukaryotic microorganisms. It is commonly used in baking, brewing, and scientific research. In particular, budding yeast, is a model organism in biology due to its simplicity, fast growth, and genetic tractability, making it ideal for studying fundamental cellular processes, including gene regulation, metabolism, and cell division. Yeast shares many essential biological pathways with higher organisms, providing valuable insights that are broadly applicable across species.

We use normalized scRNA-seq data (McCalla et al. 2023) from a study (GSE125162) (Jackson et al. 2020) of budding yeast (*Saccharomyces cerevisiae*). This original study employed Chromium droplet-based single-cell encapsulation (10x Genomics) to pool and sequence 38,285 individual yeast cells under 11 different environmental conditions. These cells represent 72 uniquely barcoded strains, encompassing 12 genotypes: a wild-type control and 11 strains with individual transcription factor deletions, each with six biological replicates. By analyzing the gene expression state across diverse conditions, the authors highlighted the significant influence of environmental factors on gene expression in yeast. For our work, we use a refined and normalized version of this dataset (provided by (McCalla et al. 2023)), where the original 38,285 cells were filtered down to 17,396 cells.

This dataset provides a rich source for constructing GRNs and enables the assessment of our methods against known interactions and transcription factor binding motifs in this model eukaryotic organism. Yeast is particularly well-suited for GRN testing and benchmarking due to several key features. First, yeast has a wealth of high-quality information on gene interactions, regulatory pathways, and transcription factor binding motifs, making it possible to benchmark GRN inference methods against well-established data. As a simple eukaryotic model, yeast shares many core biological processes with higher organisms, enabling insights that are broadly applicable while being experimentally manageable. Additionally, the genetic tractability of yeast allows for the creation of diverse genotypes, such as TF deletions or other perturbations, facilitating studies on regulatory dynamics across varied conditions. The robust gene expression changes in yeast cells in response to different environmental conditions are also valuable for testing a GRN method's ability to capture context-specific regulatory interactions. Moreover, the availability of large-scale, single-cell datasets provides a platform to test the robustness, scalability, and accuracy of GRN inference methods in a controlled, experimentally

accessible system. It has been proven a strong, model organism (e.g. test tube or environmental condition) to uncover interactions between human proteins as well (Laval et al. 2023). Overall, yeast's combination of rich data resources, genetic flexibility, and well-characterized networks makes it an ideal organism for evaluating and validating GRN inference methodologies. The final pre-processed gene expression data we use has M = 17,396 cells, 5,736 genes, and N = 279 to 280 cell-type TFs.

§4.7: human Peripheral Blood Mononuclear cells (PBMCs)

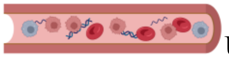
Using publicly available 10X Genomics data on 2,700 PBMCs from a healthy donor, we follow a tutorial (Satija et. al. 2024) to retrieve 9 immune cell subpopulations (denoted by Cluster C and the #) with the same 13,714 TGs each: Naïve CD4 T cells (C0), CD14 Monocytes (C1), Memory CD4 T cells (C2), B cells (C3), CD8 T cells (C4), FCGR3A Monocytes (C5), Natural Killer cells (C6), Dendritic cells (C7), Platelet cells (C8). These are any peripheral blood cells that have a round nucleus and they are located in the peripheral blood. These are crucial immune cells that help protect the body against harmful substances (e.g. pathogens) and help the body defend itself and fight off infections. We use a list of 1,639 human TFs (Lambert et al. 2018) provided by http://humantfs.ccbr.utoronto.ca/download.php. We find 1,029 TFs in this list are also in the gene expression data. These 9 PBMC-based sub cell-type applications are cases where $N \gg M$, so the input gene expression data for TFs, X, is high-dimensional.

§4.8: Genotype-Tissue Expression (GTEx) Data for Schwann Cells

We utilize this dataset as a validation dataset to benchmark NetREm in human Schwann cells and to also evaluate various results for Application 10. This GTEx data is from (Eraslan et. al 2022) for SCs pooled from 5 tissues (esophagus mucosa/muscularis, heart, prostate, skeletal muscle). After applying Seurat (Satija et. al. 2024) and MAGIC normalization, we obtained our final dataset that contains 14,144 genes across 1,430 SCs.

§4.9: Gene Expression data for 4 Neuronal/Glial Cells

We use this dataset to benchmark NetREm in 4 Central Nervous System (CNS) cell types in the absence of prior GRN information and to also evaluate various results for Application 10. Study GSE97942 (Lake et al. 2018) provides data for 4 Central Nervous System (CNS) cell types in Controls: Excitatory (Glutamatergic) Neurons, Inhibitory Neurons (InNs), Microglia (Mic), and Oligodendrocytes (Oligo). This data represents the unique molecular identifier (UMI) counts of individual cells of these 4 primary CNS cell-types; these cell-type gene expression datasets are derived from 1 postmortem tissue of the human frontal cortex. This undergoes preprocessing according to methods outlined (Jin et al. 2021), which includes removing genes expressed in <100 cells, normalization and scaling via (Satija et. al. 2024), imputing single-cell expression using the MAGIC algorithm. Please note that we followed (Jin et al. 2021) for more guidance on pre-processing this data. The final data comprises 15,692 genes across 13,709 ExNs, 6,045 InNs, 317 Mic, and 2,657 Oligo cells.

§4.10: Human Immune Cell Sub-types

We utilize single-cell gene expression data from The Tabula Sapiens Atlas (The Tabula Sapiens Consortium 2022) for 264,824 human cells in the immune system. These immune cells are broken apart into 62 immune cell subtypes and span 25 different organ tissues in the human body [Liver, Trachea, Blood, Lymph Node, Salivary Gland, Spleen, Tongue, Mammary, Uterus, Eye, Fat, Skin, Bone Marrow, Heart, Pancreas, Prostate, Muscle, Thymus, Bladder, Large Intestine, Lung, Small Intestine, Vasculature, Kidney]. All of the data is used for training as well as for testing.

We use the scanpy package (Wolf et al. 2018) in Python to load in the processed data that is in 10X Genomics format. We use this data to motivate our predictions of TG-specific TF-TF coordination B. To do this evaluation, we utilize results from (Berenson et al. 2023) that employs paired yeast one-hybrid assays to uncover TF interactions during direct DNA binding in immune-related contexts in humans. Thus, our focus is on the 125 TFs found in the Tabula Sapiens atlas as well as (Berenson et al. 2023) and these cytokine genes that were tested by (Berenson et al. 2023): *CCL15, CCL2, CCL5, CSF2, CXCL8, IFNB1, IL10, IL12B, IL18, IL1B, IL23A, IL32, IL6, TNF, TNFSF15*. Please note metrics on the # of cells (M) are shown in this plot below; bars are shaded based on the # of organ tissues that are pooled together for that given cell sub-type.

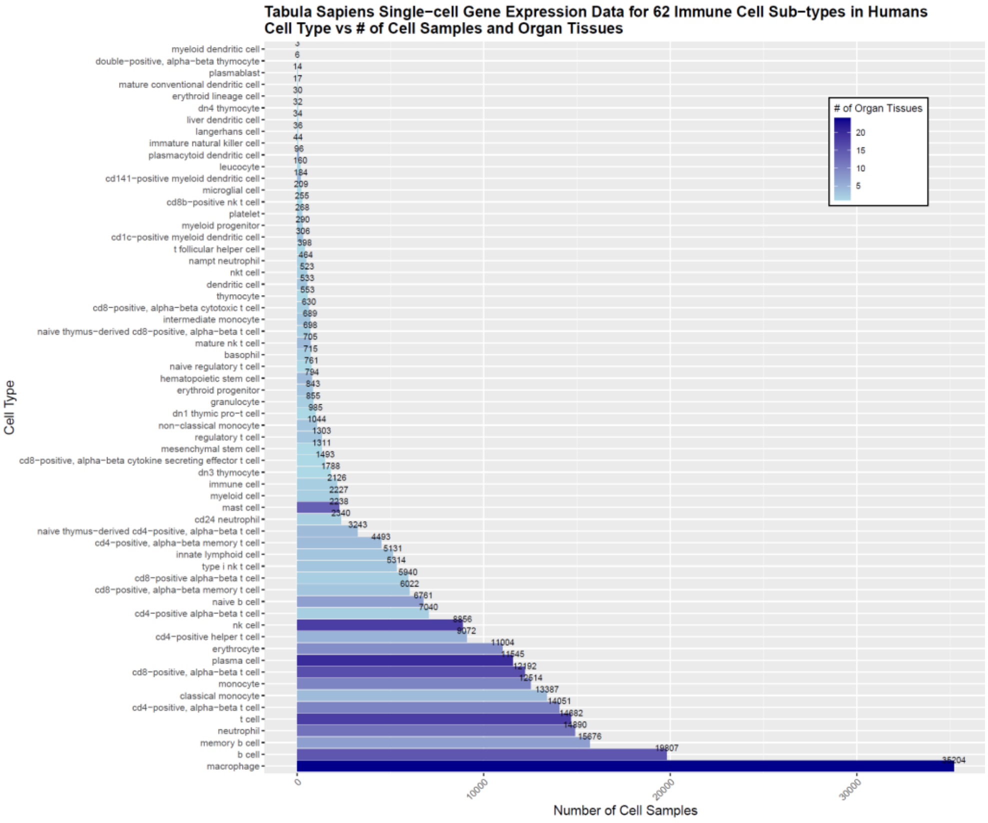

§4.11: Human myelinating (mSCs) and non-myelinating (nmSCs) Schwann cells (SCs)

We utilize Seurat (Satija et. al. 2024) to normalize and scale gene expression datasets for 13,886 genes in mSCs and nmSCs in the Dorsal Root Ganglion (DRG) L4,5 regions for 5 human donors (Avraham et al. 2022). We include only protein-coding genes and remove genes not expressed in at least 3 cells in the total data (comprised of other cell types). Our final data has 17,049 genes for 319 cells (training: M = 223, testing: 96) in mSCs and 2,468 cells (training: M = 1,727, testing: 741) in nmSCs.

§4.12: Alzheimer's disease (AD) vs. Controls in 8 Central Nervous System (CNS) Cell-Types

Astrocytes (Astro), Oligodendrocytes (Oligo), OPCs (Oligodendrocyte Precursor or Progenitor Cells), Microglia (Mic), Inhibitory GABA-ergic Neurons (InNs), Excitatory Glutamatergic Neurons (ExNs), Pericytes (Peri), Endothelial Blood Brain Barrier (BBB) cells (Endo. BBB). We use the processed data for 24 individuals with AD pathology and 24 healthy controls provided by Gupta et. al (Gupta et al. 2022) for all 8 neuronal/glial cell types. This data is from the original AD study (Mathys et. al 2019) of 80,660 droplet-based single-nucleus transcriptomes (i.e. the cells) for 17,926 genes from the prefrontal cortex for 8 neuronal/glial cell types. In AD, we have the following number of cells: Astro (1,830, $M = 1{,}281$ train, 549 test), Oligo (9,035, $M = 6{,}324$ train, 2,711 test), OPCs (1,290, $M = 903$ train, 387 test), Mic (955, $M = 668$ train, 287 test), InNs (4,371, $M = 3{,}060$ train, 1,311 test), ExNs (17,878, $M = 12{,}515$ train, 5,363 test), Pericytes (76, $M = 64$ train, 27 test), Endo. BBB (59, $M = 41$ train, 18 test). These are the respective counts in controls: Astro (1,562, $M = 1{,}093$ train, 469 test), Oligo (9,200, $M = 6{,}440$ train, 2,760 test), OPCs (1,337, $M = 936$ train, 401 test), Mic (965, $M = 676$ train, 289 test), InNs (4,825, $M = 3{,}378$ train, 1,447 test), ExNs (17,098, $M = 11{,}969$ train, 5,129 test), Pericytes (91, $M = 53$ train, 23 test), Endo. BBB (62, $M = 19$ train, 43 test).

Section §5: Overview of Building out Final Comprehensive Protein-Protein Interaction Networks (PPINs): $\mathbb{W}$

We assume users typically have a PPIN available for their specific needs that they can input to NetREm along with their respective gene expression data (and optional prior GRN knowledge).

The proteins are represented by their HGNC (HUGO Gene Nomenclature Committee) gene symbols. PPI network edges are undirected (i.e. only 1 edge weight for each TF pair) with non-negative weights (i.e. weights $w > 0$). For humans, mice, and yeast, we utilize the STRING PPI database(Szklarczyk et al. 2023). STRING has average combined scores (across several evidence types), which we scale to be between 0 and 1. This STRINGdb database focuses on proteins that have a function association and specific partnership. These proteins are physically associated with each other or are indirectly associated (e.g. work together in a common metabolic or signaling pathway, regulate each other through intermediaries, contribute to a common biological cause)(Szklarczyk et al. 2023). That is, according to BioGRID(Oughtred et al. 2021) (one of the PPI resources used by STRING) the definition of interaction among 2 proteins can include: direct physical binding of 2 proteins, co-existence in a stable complex, and/or genetic interaction. Thus, this STRING PPI database has edges that represent protein-protein associations among proteins that jointly contribute to a shared, specific, and meaningful function, regardless of whether the proteins physically bind to each other. Currently, the latest STRINGdb release is version 12 (v12), which includes data since July 26, 2023.

Our final comprehensive PPI networks $\mathbb{W}$ for each organism contain PPI links with edge weight $w > 0.01$ and are thereby typically partly-connected networks. These links encompass various interactions. There are no self-loops in our network. Then, we filter these networks to get our TF-TF PPI networks. For a given TG in a context, we will subset this network $\mathbb{W}$ for just its N candidate TFs, to get a TF-TF PPIN. Here, the maximum weight for any TF_i- TF_j interaction is 1 (that is, $0.01 < w_{ij} \leq 1$), where $TF_i \neq TF_j$. We add artificial links ($\eta = 0.01$) to this network to make it connected pairwise for all N candidate TFs to yield W, our TG-specific TF-TF PPI network: $0.01 \leq w_{ij} \leq 1$, where $TF_i \neq TF_j$ and all N candidate TFs have pairwise edges with the other $N - 1$ edges. We do not consider self-loops (i.e. we do not consider TF_i- TF_i interactions of the same TF with itself). Instead, we set $W_{ii} = \frac{d_i}{N-1}$ where TF_i's degree (connectivity) with $N - 1$ other TFs is $d_i = \sum_{k \neq i} w_{ik} > 0$.

Please note that while we choose $\eta = 0.01$ as our minimum default weight for all edges in our PPIN, the user may choose any weight they would like. Further, we do recommend weights be capped at 1, but this is not a requirement and other weight scales can be used (e.g. weights between 1 and 100).

For our main applications, we utilize more reliable and updated networks, which are organism-specific given the limitations of available data. Although we focus on organism-specific PPINs, NetREm is adaptable and can be applied using context-specific PPINs if available to the user. While the contextual Integrated Interactions Database (IID) serves as a valuable resource, it is primarily tissue-specific and lack annotations for cell types, limiting its use for constructing the main PPINs. In addition, the Contextual IID does not provide any weights. Instead, we use the IID to validate the context-specificity of our predicted networks given that it is primarily tissue-specific and to retrieve any knowledge regarding any TFs that form complexes. In any case, we do use the contextual IID as 1 of the input networks for our benchmarking analyses across 13 human contexts.

Further, we do tend to filter the input PPINs for TFs that are relevant to the cell-type and application. Thus, the input PPINs may reflect direct and/or indirect PPIs among cell-type TFs. These input PPINs may thus inherently capture cell-type PPIs.

Below, we explain the processes for obtaining these giant, partly-connected, PPI networks $\mathbb{W}$ in humans (homo sapiens), mice (mus musculus), and yeast (Saccharomyces Cerevisiae). Please note that to build our $\mathbb{W}$, we will remove scores with $w < 0.01$ since we will later add minimum default edge weights of 0.01 to any missing edges in $\mathbb{W}$ to make the input TF-TF input PPIN fully connected. In the next section, (Section §**6**), we explain the process for deriving the fully-connected comprehensive TF-TF input PPINs.

§5.1 Final Comprehensive PPI Networks (PPINs) in Humans

Organism: Humans (Homo Sapiens)

§5.1.1 Max SAINT Score PPIN

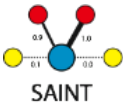

We used TF-TF human PPIN links from a recent study (Göös et al. 2022) investigating human TF protein interaction networks via rigorous physical experimental analysis of 109 different proteins (1,431 TFs). Please note that we will also use these 1,431 TFs to filter the other human PPINs. That study recognizes interactions with a SAINT (Significance Analysis of INTeractome) score above 0.74, as assessed by SAINTexpress software version 3.1.0[108], as being significant and highly-confident. It uncovers 6,503 and 1,336 strong direct TF-TF interactions through BioID and AP-MS analysis respectively, with 200 common interactions, many of which are novel and absent in input PPI. That is, it returns TF-TF direct interactions with high-confidence based on BioID (proximity-dependent biotin identification) and/or AP-MS (Affinity Purification-Mass Spectroscopy) experiments with strong SAINT scores. There are 8,034 unique edges in the PPIN with weights corresponding to SAINT scores. Some edges are found by BioID, some by AP-MS, others by both experimental techniques. In cases where a TF-TF edge is found by both experiments, the maximum SAINT score is used; hence, we refer to this input PPIN as the Max Saint Score PPIN. That is, (Göös et al. 2022) returns TF-TF direct interactions with high-confidence based on BioID and/or AP-MS experiments with strong SAINT scores: Highly Confident Novel Human TF-TF Protein Interaction Networks based on BioID and/or AP-MS physical experiments. This weighted PPIN has weights between 0.74 and 1, corresponding to SAINT scores.

§5.1.2 Barabasi (Human Interactome) PPIN

We utilize a human interactome assembled from 21 public databases that contain experimentally derived. This PPIN is provided by a recent study on network medicine frameworks for identifying drug-repurposing opportunities for Covid-19 (Morselli Gysi et al. 2021). In that study, the authors constructed a human interactome based on PPIs from the following resources of interactions that include: 1) binary PPIs identified from high-throughput yeast two-hybrid experiments and 3-dimensional protein structures; 2) interactions found through affinity purification followed by mass spectrometry; 3) kinase-substrate interactions; 4) signaling interactions; and 5) regulatory interactions. The final interactome encompasses 18,505 proteins and 327,924 connections between them. Additionally, interactions between SARS-CoV-2 and human proteins reported by Gordon et al. were incorporated, along with drug–target information sourced from the DrugBank database. We call this the Barabasi PPIN since the PPIN is available under the Covid-19 repository on the GitHub site under the Barabasi-Lab: https://github.com/Barabasi-Lab/COVID-19 (DatasetS2.csv). We load in the original dataset of 327,924 rows. These proteins are given in the form of their gene Entrez IDs. We convert the Entrez ID to HGNC gene symbols using the `mygene` package([CSL STYLE ERROR: reference with no printed form.]) in Python. We drop self-loops and remove duplicates to then obtain 322,162 unique edges. This is a binary, dummy network, so we assign a score of 1 to these edges.

§5.1.3 Final Interactome PPIN

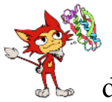

Our final interactome PPIN contains 117,897 unique protein-protein interactions provided by a recent study (Kosoglu et. al 2024) that forms a reference human interactome. This final interactome PPIN is a binary, dummy network: there are no scores provided. Hence, for edges in the network, we provide a weight of 1. To make the network fully connected, we add a minimum default edge weight of 0.01 for missing edges.

This study mapped all known experimental protein structures to the reference human proteome and analyzed structural coverage improvements using homology modeling and deep learning (AlphaFold). The research compiled interactions from scientific literature and databases, forming a reference human interactome consisting of 117,897 unique protein-protein interactions (PPIs). The interactome was constructed by reviewing databases like HuRI, STRING, BioPlex, BioGRID, HIPPIE, IID, APID, and PICKLE. The interactions from HuRI, STRING, and HIPPIE were explored further. For STRING, the dataset '9606.protein.physical.links.detailed.v11.5.txt' was downloaded and rescored using a provided Python script, with models having combined experimental and database scores greater than 0.7 being retained. The HuRI dataset was obtained as a TSV file from the 2020 publication. For HIPPIE, the v2.3 release was filtered to include only binary PPIs detected by two-hybrid, atomic force microscopy, or fluorescence resonance energy transfer methods, and a high-quality threshold of 0.73 was applied. All datasets were standardized using UniProt identifiers, and redundant interactions were removed. We mapped the UniProt protein IDs to gene names using the mapping database provided by https://rest.uniprot.org/uniprotkb. Ultimately, we uncovered 116,650 unique PPI edges.

§5.1.4 HIPPIE (Human Integrated Protein-Protein Interaction rEference v2.3 release)

HIPPIE provides a weighted PPI Network with weights based on the experimental resources(Alanis-Lobato et. al 2017). In the original PPI network, we find there are 831,833 rows with scores between 0 and 1 based on experiment sources. Of those, 826,805 rows have scores above 0.01. We remove any rows with missing values and any self-loops and we remove any duplicated rows. Ultimately, our HIPPIE network is filtered to 765,220 unique PPI edges. This is a weighted PPI network with scores greater than 0.01 and less than or equal to 1.

§5.1.5 Hu.MAP 2.0

We retrieve the Hu.MAP 2.0 PPI Network (Drew et al. 2021) that is a weighted protein-protein interaction network with probability scores. This network provides all protein pair predictions with the corresponding Support Vector Machine (SVM) probability score. This network has 17,526,289 rows. We filter out self-loops and rows with weight less than or equal to 0.01. Ultimately, we uncover 36,059 unique PPIs. This is a weighted PPI network with scores greater than 0.01 and less than or equal to 1.

§5.1.6 Huri_HI-union PPIN: Human Reference Interactome (HuRI)

This PPIN is based on data released by The Human Reference Protein Interactome Mapping Project. We obtain the Huri_HI-union PPIN from http://www.interactome-atlas.org (HI-union dataset) that details 64,006 interactions(Luck et al. 2020). We map Ensembl Gene IDs to gene names using the mygene package in Python. We then uncover 63,242 rows. We remove self-loops (i.e. where the PPI corresponds to the same protein interacting with itself). Ultimately, our network corresponds to 63,185 unique edges and is a binary, dummy network. Hence, we assign a score of 1 to all edges in this network.

§5.1.7 Multiscale Interactome

We obtained the multiscale interactome from a previous study (Ruiz et al. 2021) that built this human interactome to help identify disease treatment mechanisms. This interactome is available at https://github.com/snap-stanford/multiscale-interactome. All of the data is available here: http://snap.stanford.edu/multiscale-interactome/data/data.tar.gz. After removing missing rows, we find 385,692 unique edges in this binary, dummy network. We assign scores of 1 to all these edges.

§5.1.8 STRINGv11 database

- STRINGv11 All: direct and/or indirect protein-protein interactions

The human STRINGdb version 11 (v11 or V11) network is archived and refers to the PPIN with updates from January 19, 2019, to October 17, 2020. We navigate to https://version-11-0.string-db.org/ and go to the Download section, where we enter 9606 (homo sapiens: human) for the organism.

We obtain the protein network data (interaction types for protein links) as 9606.protein.actions.v11.0.txt.gz. This data file annotates protein-protein interactions in humans as: unknown, reaction, catalysis, binding, activation, inhibition, ptmod, and expression. In this case, we will use all the interactions that are provided and will not filter for any criteria.

9606.protein.actions.v11.0.txt

	item_id_a	item_id_b	mode	action	is_directional	a_is_acting	score
0	9606.ENSP0000000233	9606.ENSP0000216366	binding	NaN	f	f	165
1	9606.ENSP0000000233	9606.ENSP0000216366	reaction	NaN	f	f	165
2	9606.ENSP0000000233	9606.ENSP0000216366	reaction	NaN	t	f	165
3	9606.ENSP0000000233	9606.ENSP0000216366	reaction	NaN	t	t	165
4	9606.ENSP0000000233	9606.ENSP0000222547	binding	NaN	f	f	913
...	...	...	...	...	...	...	...
3470901	9606.ENSP00000485678	9606.ENSP00000409581	inhibition	inhibition	f	f	600
3470902	9606.ENSP00000485678	9606.ENSP00000409581	ptmod	NaN	f	f	600
3470903	9606.ENSP00000485678	9606.ENSP00000438346	activation	activation	t	f	900
3470904	9606.ENSP00000485678	9606.ENSP00000481878	activation	activation	f	f	600
3470905	9606.ENSP00000485678	9606.ENSP00000481878	binding	NaN	f	f	900

3470906 rows × 7 columns

9606.protein.info.v11.0.txt

	protein_external_id	preferred_name	protein_size	annotation
0	9606.ENSP00000000233	ARF5	180	ADP-ribosylation factor 5; GTP-binding protein...
1	9606.ENSP00000000412	M6PR	277	Cation-dependent mannose-6-phosphate receptor;...
2	9606.ENSP00000001008	FKBP4	459	Peptidyl-prolyl cis-trans isomerase FKBP4; Imm...
3	9606.ENSP00000001146	CYP26B1	512	Cytochrome P450 26B1; Involved in the metaboli...
4	9606.ENSP00000002125	NDUFAF7	441	Protein arginine methyltransferase NDUFAF7, mi...
...	...	...	...	...
19561	9606.ENSP00000485671	ENSG00000280273	120	HCG1991042
19562	9606.ENSP00000485672	ENSG00000279458	86	annotation not available
19563	9606.ENSP00000485673	ENSG00000279988	243	annotation not available
19564	9606.ENSP00000485675	ENSG00000280116	84	annotation not available
19565	9606.ENSP00000485678	OR6Q1	317	Olfactory receptor 6Q1; Odorant receptor; Olfa...

19566 rows × 4 columns

We map the proteins (please see columns “item_id_a” and “item_id_b” in the leftmost snapshot above) from their Ensembl protein IDs (e.g. ENSP IDs) to their the updated gene names using the file 9606.protein.info.v11.0.txt.gz that

details annotations for 19,566 proteins (“preferred_name” in the rightmost snapshot above). This yields this updated dataset below, where edges are annotated with gene names.

	item_id_a	item_id_b	mode	action	is_directional	a_is_acting	combined_score	gene_1	gene_2	gene1_gene2	
**0**	9606.ENSP00000000233	9606.ENSP00000216366	binding	NaN	f	f	165.0	ARF5	AP4S1	ARF5**AP4S1	STRINGdb_960
**1**	9606.ENSP00000000233	9606.ENSP00000216366	reaction	NaN	f	f	165.0	ARF5	AP4S1	ARF5**AP4S1	STRINGdb_960
**2**	9606.ENSP00000000233	9606.ENSP00000216366	reaction	NaN	t	f	165.0	ARF5	AP4S1	ARF5**AP4S1	STRINGdb_960
**3**	9606.ENSP00000000233	9606.ENSP00000216366	reaction	NaN	t	t	165.0	ARF5	AP4S1	ARF5**AP4S1	STRINGdb_960
**4**	9606.ENSP00000000233	9606.ENSP00000222547	binding	NaN	f	f	913.0	ARF5	BET1	ARF5**BET1	STRINGdb_960
**...**	...	...	...	...	...	...	...	...	...	...	
**3470901**	9606.ENSP00000485678	9606.ENSP00000409581	inhibition	inhibition	f	f	600.0	OR6Q1	ARRB1	OR6Q1**ARRB1	STRINGdb_960
**3470902**	9606.ENSP00000485678	9606.ENSP00000409581	ptmod	NaN	f	f	600.0	OR6Q1	ARRB1	OR6Q1**ARRB1	STRINGdb_960
**3470903**	9606.ENSP00000485678	9606.ENSP00000438346	activation	activation	t	f	900.0	OR6Q1	REEP1	OR6Q1**REEP1	STRINGdb_960
**3470904**	9606.ENSP00000485678	9606.ENSP00000481878	activation	activation	f	f	600.0	OR6Q1	GNB1	OR6Q1**GNB1	STRINGdb_960
**3470905**	9606.ENSP00000485678	9606.ENSP00000481878	binding	NaN	f	f	900.0	OR6Q1	GNB1	OR6Q1**GNB1	STRINGdb_960

3470906 rows × 11 columns

We average the scores across the same human gene pairs (in case there are duplicate pairs with different scores) to obtain combined scores. We find the maximum score in the dataset, which we use to scale all scores. That is, we divide all combined score values in this dataset by this maximum value to obtain scores greater than 0 but less than or equal to 1. Then, we filter out rows with scaled scores less than or equal to 0.01. Ultimately, we uncover 544,436 unique PPI edges for our STRINGdb V11 network, which represent any and all potential interaction betwixt the proteins, whether direct or indirect. This is a weighted network with edge weights between 0.01 and 1.

- STRINGv11 Physical only STRING

Here, we follow the same steps as above to obtain our input data files (actions file: 9606.protein.actions.v11.0.txt.gz, info file: 9606.protein.info.v11.0.txt.gz). In this case, we filter the actions data for only actions that involve binding as that implies direct interactions between the proteins (e.g. physical binding to form complexes to regulate TGs). That is, we only retain rows where the action = “binding”. We average the scores across the same human gene pairs (in case there are duplicate pairs with different scores) to obtain combined scores. We find the maximum score in the dataset, which we use to scale all scores. That is, we divide all combined score values in this dataset by this maximum value to obtain scores greater than 0 but less than or equal to 1. Then, we filter out rows with scaled scores less than or equal to 0.01. Ultimately, we uncover 325,467 unique PPI edges for our STRINGdb V11 network, which represent any direct, physical binding interaction betwixt the proteins. This is a weighted network with edge weights between 0.01 and 1.

§5.1.9 STRINGv12 database

- STRINGv12 All STRING

The human STRINGdb version 12 (v12 or V12) network refers to most recent PPIN (as of this publication time point). We navigate to https://string-db.org// and go to the Download section, where we enter 9606 (homo sapiens: human) for the organism. We obtain the protein network data (full network, scored links between proteins) as **9606**.protein.links.v12.0.txt.gz. We use all these comprehensive interactions that are provided; these interactions include indirect associations as well as direct interactions. We will map the proteins (please see columns “protein1” and “protein2” in the leftmost snapshot below) from their Ensembl protein IDs (e.g. ENSP IDs) to their the updated gene names using the file **9606**.protein.info.v12.0.txt.gz that details annotations for 19,699 proteins (“preferred_name” in the rightmost snapshot below).

9606.protein.links.v12.0.txt

	protein1	protein2	combined_score
0	9606.ENSP00000000233	9606.ENSP00000356607	173
1	9606.ENSP00000000233	9606.ENSP00000427567	154
2	9606.ENSP00000000233	9606.ENSP00000253413	151
3	9606.ENSP00000000233	9606.ENSP00000493357	471
4	9606.ENSP00000000233	9606.ENSP00000324127	201
...	...	...	...
13715399	9606.ENSP00000501317	9606.ENSP00000475489	195
13715400	9606.ENSP00000501317	9606.ENSP00000370447	158
13715401	9606.ENSP00000501317	9606.ENSP00000312272	226
13715402	9606.ENSP00000501317	9606.ENSP00000402092	169
13715403	9606.ENSP00000501317	9606.ENSP00000404074	251

13715404 rows × 3 columns

9606.protein.info.v12.0.txt

	#string_protein_id	preferred_name	protein_size	annotation
0	9606.ENSP00000000233	ARF5	180	ADP-ribosylation factor 5; GTP-binding protein...
1	9606.ENSP00000000412	M6PR	277	Cation-dependent mannose-6-phosphate receptor;...
2	9606.ENSP00000001008	FKBP4	459	Peptidyl-prolyl cis-trans isomerase FKBP4, N-t...
3	9606.ENSP00000001146	CYP26B1	512	Cytochrome P450 26B1; Involved in the metaboli...
4	9606.ENSP00000002125	NDUFAF7	441	Protein arginine methyltransferase NDUFAF7, mi...
...	...	...	...	...
19694	9606.ENSP00000501254	ALDH3B2	385	Aldehyde dehydrogenase family 3 member B2; Oxi...
19695	9606.ENSP00000501259	FAM163B	166	Protein FAM163B; Family with sequence similari...
19696	9606.ENSP00000501265	NWD1	1432	NACHT domain- and WD repeat-containing protein...
19697	9606.ENSP00000501277	LDB1	411	LIM domain-binding protein 1; Binds to the LIM...
19698	9606.ENSP00000501317	RFX7	1460	DNA-binding protein RFX7; Regulatory factor X7...

19699 rows × 4 columns

This yields the updated dataset below, where edges are annotated with gene names.

	protein1	protein2	combined_score	gene_1	gene_2
0	9606.ENSP00000000233	9606.ENSP00000356607	173	ARF5	RALGPS2
1	9606.ENSP00000000233	9606.ENSP00000427567	154	ARF5	FHDC1
2	9606.ENSP00000000233	9606.ENSP00000253413	151	ARF5	ATP6V1E1
3	9606.ENSP00000000233	9606.ENSP00000493357	471	ARF5	CYTH2
4	9606.ENSP00000000233	9606.ENSP00000324127	201	ARF5	PSD3
...	...	...	...	...	...
13715399	9606.ENSP00000501317	9606.ENSP00000475489	195	RFX7	MPHOSPH9
13715400	9606.ENSP00000501317	9606.ENSP00000370447	158	RFX7	VCX
13715401	9606.ENSP00000501317	9606.ENSP00000312272	226	RFX7	YPEL2
13715402	9606.ENSP00000501317	9606.ENSP00000402092	169	RFX7	SAMD3
13715403	9606.ENSP00000501317	9606.ENSP00000404074	251	RFX7	ZSCAN9

13715404 rows × 5 columns

	TF1	TF2	score
0	A1BG	A2M	0.692693
1	A1BG	A2ML1	0.256256
2	A1BG	A4GNT	0.154154
3	A1BG	AADAC	0.175175
4	A1BG	AADACL2	0.177177
...	...	...	...
13715399	hCG_2039566	ZFAND4	0.198198
13715400	hCG_2039566	ZNF248	0.166166
13715401	hCG_2039566	ZNF711	0.165165
13715402	hCG_2039566	ZNHIT1	0.234234
13715403	hCG_2039566	ZNRD1	0.189189

→ 13715404 rows × 3 columns

In the process, we obtain our v12 network that has [human gene 1, human gene 2, combined score]. We average combined scores across the same human gene pairs (in case there are duplicate pairs with different scores) to obtain average combined scores. We find the maximum average combined score in the dataset, which we use to scale all average combined scores. That is, we divide all average combined score values in this dataset by this maximum value to obtain scaled scores greater than 0 but less than or equal to 1. Then, we filter out rows with scaled scores less than or equal to 0.01. Ultimately, the final comprehensive PPI v12 network of direct and/or indirect interactions for humans has 13,715,404 rows corresponding to 6,857,702 unique undirected edges among proteins. This human PPI network $\mathbb{W}$ reflects the updated knowledge regarding PPIs among the proteins in humans. This is a weighted network with edge weights between 0.01 and 1: $0.01 < w \leq 1$ and we consider these links as known PPIs.

- STRINGv12 Physical only STRING

9606.protein.physical.links.v12.0.txt

	protein1	protein2	combined_score
0	9606.ENSP00000000233	9606.ENSP00000257770	311
1	9606.ENSP00000000233	9606.ENSP00000226004	161
2	9606.ENSP00000000233	9606.ENSP00000434442	499
3	9606.ENSP00000000233	9606.ENSP00000262455	531
4	9606.ENSP00000000233	9606.ENSP00000303145	499
...	...	...	...
1477605	9606.ENSP00000501317	9606.ENSP00000444357	292
1477606	9606.ENSP00000501317	9606.ENSP00000296785	962
1477607	9606.ENSP00000501317	9606.ENSP00000361930	195
1477608	9606.ENSP00000501317	9606.ENSP00000370745	188
1477609	9606.ENSP00000501317	9606.ENSP00000290524	618

1477610 rows × 3 columns

We navigate to https://string-db.org// (displays most recent STRINGdb v12 network) and go to the Download section, where we enter 9606 (homo sapiens: human) for the organism. We obtain the protein network data (physical subnetwork, including subscores per channel) as 9606.protein.physical.links.v12.0.txt.gz. These interactions are a subnetwork of the original larger network and comprise direct physical interactions between the proteins. There are 1,477,610 rows in this data resource.

We will map the proteins (please see columns "protein1" and "protein2" in the snapshot on the left) from their Ensembl protein IDs (e.g. ENSP IDs) to their the updated gene names using the file 9606.protein.info.v12.0.txt.gz that details annotations for 19,699 proteins.

In the process, we obtain our v12 physical network that has [human gene 1, human gene 2, combined score]. We average combined scores across the same human gene pairs (in case there are duplicate pairs with different scores) to obtain average combined scores. We find the maximum average combined score in the dataset, which we use to scale all average combined scores. That is, we divide all average combined score values in this dataset by this maximum value to obtain scaled scores greater than 0 but less than or equal to 1. Then, we filter out rows with scaled scores less than or equal to 0.01 (that is, we remove edges with $w \leq 0.01$). Ultimately, the final comprehensive PPI v12 network of direct, physical interactions for humans has 1,477,610 rows corresponding to 738,805 unique undirected edges among proteins. This human PPI network 𝕎 reflects the updated knowledge regarding direct, physical PPIs among the proteins in humans. This is a weighted network with edge weights between 0.01 and 1.

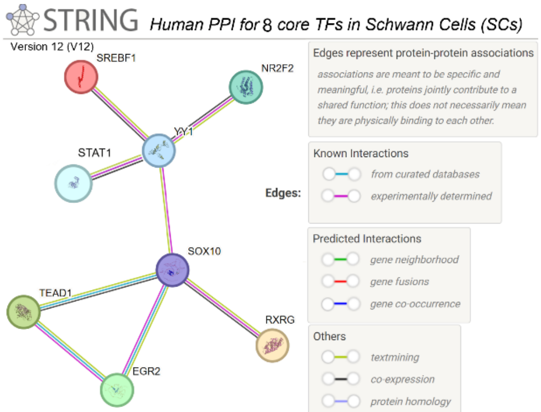

§5.1.10 PBMC STRINGdb V11 Network

For our applications in human PBMCs, we obtain our version 11 STRINGdb network using another approach. We navigate to the archived website: https://version-11-0.string-db.org/. We use the following input data files: PPI links file (9606.protein.links.v11.0.txt.gz) and annotations info file (9606.protein.info.v12.0.txt.gz). Our PPI links file has 11,759,454 rows of [Protein1 ID, Protein2 ID, combined score]. We map these protein IDs to gene symbol names using the more updated STRINGdb v12 protein information file instead. In the process, we obtain our v11 network that has [human gene 1, human gene 2, combined score]. We average combined scores across the same human gene pairs (in case there are duplicate pairs with different scores) to obtain average combined scores. Ultimately, the final comprehensive PPI v11 network for humans has 9,521,964 rows corresponding to 4,760,982 unique undirected edges among proteins. This human PPI network 𝕎 reflects the older (potentially outdated) knowledge regarding PPIs among the proteins in humans and will be a baseline.

§5.1.11 Main NetREm PPI Network (Comprehensive Network used in several applications)

We construct a comprehensive human PPI network of associated interactions, with weights $w > 0$, from multiple sources. STRINGdb v12 (Szklarczyk et al. 2023) provides 6,857,702 undirected edges for humans (id: 9606); we use the preprocessing steps mentioned above for STRINGv12 All. Hu.MAP 2.0 (Drew et al. 2021) contributes ≈8.75 million edges with probability weights (protein pairs with the corresponding svm probability score), which we use as weights. Other data, including protein complexes and PPIs, comes from MaayanLab (e.g. BioGRID, DIP, HPRD, HubProteins, NURSA, CORUM) (Giurgiu et al. 2019), Human Reference Interactions (HuRI) (Luck et al. 2020), TF complexes in: Position Weighted Networks (PWMs), contextual Integrated Interactions Database (IID) (Kotlyar et. al 2022), KEGG Pathway Networks (Kanehisa and Goto 2000) that we web-scraped using the BeautifulSoup package (Richardson) in Python. These sources are integrated to build our final PPIN. A weight of 1 is assigned to any TFs found to physically interact in complexes with each other in any source. For PPI links found in multiple sources, we use the highest weight for that link.

We filter this network to only include links with edge $w > 0.01$. Ultimately, we have 8,701,636 undirected PPI edges in our final comprehensive PPIN 𝕎, our ground truth for humans. All weights are between 0 (missing link) and 1 as this net work is partly-connected. Here, links may correspond to direct physical interactions and/or indirect TF-TF interactions with evidence of functional association in humans. We may refer to this network as NewNet or our Main NetREm PPI Net work and will use this for many applications in this paper. For instance, we use this main NetREm PPI network for our my elinating versus non-myelinating Schwann cell application (Application #10) and our Alzheimer's disease versus controls application for the 8 cell types (Application #11).

§5.1.12 Contextual Integrated Interactions Database (IID) PPIN

We obtain our contextual integrated interactions database (IID) PPIN from https://iid.ophid.utoronto.ca/ based on a study (Kotlyar et. al 2022) that annotates existing PPI networks using various contextual terms (e.g. disease affiliation, tissue). We retain 112 terms that have annotations and/or tissues/regions that are specific to the nervous system. Please note that we do this since we will be using this PPIN as 1 of 13 possible PPINs in our benchmarking analysis across 13 different human contexts (that are for datasets in the nervous system).

1. Brain Regions and Structures

- Lobes: Frontal lobe; Parietal lobe; Temporal lobe; Occipital lobe; Limbic lobe
- **Cerebral Structures:** Cerebral cortex; Telencephalon; Basal ganglia; Striatum; Caudate nucleus; Body of the cau date nucleus; Head of the caudate nucleus; Cerebral nuclei
- **Hippocampal Formation and Related Structures:** Hippocampal formation; Subiculum; CA1 field; CA3 field; CA4 field; Dentate gyrus; Parahippocampal gyrus; Parahippocampal gyrus, left
- **Gyrus Structures:** Superior frontal gyrus; Middle frontal gyrus; Cingulate gyrus; Cingulate gyrus, frontal part; P ostcentral gyrus; Inferior temporal gyrus; Fusiform gyrus; Lingual gyrus; Gyrus rectus; Precuneus; Superior pariet al lobule; Superior temporal gyrus; Claustrum

2. Laterality-Specific Brain Regions

- **Left-Sided Regions:** Putamen, left; Superior frontal gyrus, left; Middle frontal gyrus, left; Cingulate gyrus, fronta l part, left; Parahippocampal gyrus, left; Claustrum, left; Subiculum, left; CA1 field, left; CA3 field, left; Dentate gyrus, left; Inferior temporal gyrus, left; Lingual gyrus, left; Fusiform gyrus, left; Body of caudate nucleus, left; H ead of caudate nucleus, left; Superior parietal lobule, left; Precuneus, left; Middle temporal gyrus, left

3. Specific Subregions within Gyri

- **Superior Frontal Gyrus:Superior frontal gyrus, left, medial bank of gyrus; Superior frontal gyrus, left, late ral bank of gyrus**
- **Middle Temporal Gyrus:** Middle temporal gyrus, left, superior bank of gyrus; Middle temporal gyrus, left, inferi or bank of gyrus
- **Cingulate Gyrus:** Cingulate gyrus, frontal part, left, superior bank of gyrus; Cingulate gyrus, frontal part, left, inf erior bank of gyrus
- **Inferior Temporal Gyrus:** Inferior temporal gyrus, left, lateral bank of gyrus; Inferior temporal gyrus, left, bank of mts; Inferior temporal gyrus, left, bank of the its
- **Lingual Gyrus:** Lingual gyrus, left, striate; Lingual gyrus, left, peristriate
- **Precuneus:** Precuneus, left, superior lateral bank of gyrus; Precuneus, left, inferior lateral bank of gyrus

4. Basal Ganglia and Thalamic Structures

- **Basal Ganglia: Putamen; Basal ganglia**
- **Thalamic Structures:** Thalamus; Dorsal thalamus; Lateral group of nuclei; Lateral group of nuclei, ventral divisi on; Diencephalon

5. Brainstem and Associated Nuclei

- **Pons and Related Structures:** Pons; Basal part of pons; Pontine nuclei, left; Pontine nuclei; Metencephalon
- **Spinal and Brainstem Nuclei:** Spinal trigeminal nucleus; Spinal trigeminal nucleus, left; Dorsal root ganglia; Inf erior olivary complex, left; Substantia nigra; Substantia nigra, pars compacta, left; Midbrain tegmentum; Mesence phalon; Myelencephalon

6. Conditions and Diseases

- **Nervous System and Neurodegenerative Diseases:** Nervous system disease; neurodegenerative disease; Central nervous system disease; Astrocytoma; Tauopathy

7. General Terms: Brain, Amygdala, Telencephalon

§5.2 Final Comprehensive PPI Networks (PPINs) in Mice

Organism: Mouse (Mus Musculus)

We utilize 2 different versions of the STRING PPI database for mice (id: 10090): version 11 (v11) and v12.

§5.2.1 STRINGdb V11 PPIN in mice (Older PPIN)

The mouse STRINGdb v11 PPIN has updates from January 19, 2019, to October 17, 2020: 11,944,806 rows of [Protein1 ID, Protein2 ID, combined score]. We map protein IDs to gene symbol names using the more updated v12 protein info file for 21,840 proteins in mice. In the process, we retain 8,600,490 rows for our v11 network that has [mouse gene 1, mouse gene 2, combined score]. Then, we average combined scores across the same mouse gene pairs (in case there are duplicate pairs with different scores) to obtain average combined scores. Ultimately, the final comprehensive v11 PPIN $\mathbb{W}$ for mice has 8,600,490 rows corresponding to 4,300,245 unique undirected edges among proteins. This mouse PPIN reflects the older (potentially outdated) knowledge regarding PPIs among the proteins in the newer v12 PPIN and will be a baseline.

§5.2.2 STRINGdb V12 PPIN in mice (New PPIN)

We perform the same data pre-processing steps as we did for the v11 network for the mouse STRINGdb v12 PPI network (PPIN) that has 12,684,354 rows. Then, we use motif complexes from the JASPAR2022 (Castro-Mondragon et al. 2022) database for mice to adjust the PPI scores to be 1 (our maximum) for 11 TF pairs known to be in a complex (i.e. AHR::ARNT, HAND1::TCF3, GATA1::TAL1, POU5F1::SOX2, PPARG::RXRA, FOS::JUN, ARNT::HIF1A, NR1H3::RXRA, STAT5A::STAT5B, BACH1::MAFK, ZIC1::ZIC2). Ultimately, the final mouse comprehensive v12 PPIN $\mathbb{W}$ has 12,684,354 rows corresponding to 6,342,177 unique undirected edges among the proteins. This is the newer PPIN for mice, which reflects the current knowledge regarding PPIs among the proteins.

§5.3 Final Comprehensive PPI Networks (PPINs) in Yeast

For our application in yeast, we use the STRINGdb version 12 for all interactions (indirect and/or direct). We use the following input data files for organism (4932): PPI links file (4932.protein.links.v12.0.txt.gz) and annotations info file (4932.protein.info.v12.0.txt.gz). Our PPI links file has rows of [Protein1 ID, Protein2 ID, combined score]. We map these protein IDs to gene symbol names using the protein information file instead. In the process, we obtain our v12 network that has [yeast gene 1, yeast gene 2, combined score] to obtain 2,824,842 rows. We average combined scores across the same yeast gene pairs (in case there are duplicate pairs with different scores) to obtain average combined scores for 1,412,421. When running NetREm in budding yeast, we utilize this input PPI network. However, many of the 280 candidate TFs are not found in the initial STRINGdb V12 network with their original symbols. Hence, we used g:Profiler's g:Convert function(Reimand et al. 2007) to obtain gene ID conversions to obtain the proper nomenclature to locate the TFs in the network. Ultimately, 278 TFs had existing, known PPI links in the database. This yeast PPI network input TF-TF network $\mathbb{W}$ has 27,798 rows, with weight (i.e. scores) above 0.01 for the 280 candidate TFs for the TGs in budding yeast.

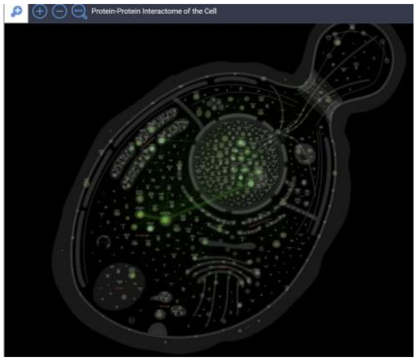

On the right is a visualization of the yeast interactome provided by http://www.yeast-interactome.org/ from a study on the social and structural architecture of the yeast interactome (Michaelis et al. 2023)

§5.4 Rules for handling weighted versus binary (dummy) PPI Networks

For these binary PPINs we assign a score of 1 to all links described in the network; the missing pairwise links will be assigned a default edge weight of 0.01. We note that all of our networks are filtered so that all network weights in $\mathbb{W}$ have edge weights $0.01 < w \leq 1$. These links represent potential 'known' links. This network is likely a partly-connected network as not all proteins need interact with one another. (We note that in the next section, we will make this network fully-connected by adding in minimum edge weights of 0.01 to any missing pairwise edges).

§5.5 Overview of Input PPI Network Used for Different Analyses

**Input PPI Network**	**Organism**	**Explanation of network and scores**	**PPI types**	**Analyses that input this network for NetREm (Application #s are listed)**
Max SAINT Score PPIN	Human	Weights between 0.01 and 1	Only direct, physical PPIs	
Barabasi (Human Interactome) PPIN	Human	Binary: 1 if present	Only direct, physical PPIs	

Final Interactome		Human	Binary: 1 if present	Only direct, physical PPIs	Comparison for 13 human contexts in the absence of prior GRN information
HIPPIE (Human Integrated Protein-Protein Interaction rEference v2.3 release)		Human	Weights between 0.01 and 1		
Hu.MAP 2.0 PPIN		Human	Weights between 0.01 and 1	All: Direct and/or Indirect TF-TF PPIs	
Huri_HI-union PPIN		Human	Binary: 1 if present		
Multiscale Interactome		Human	Binary: 1 if present		
Contextual Integrated Interactions Database (IID) PPIN<br>Filtered for relevant terms → Context-specific IID		Human	Binary: 1 if present	Only direct, physical PPIs	
STRINGdb	STRINGv11 All	Human	Weights between 0.01 and 1	All: Direct and/or Indirect TF-TF PPIs	
	STRINGv11 Physical	Human		Only Direct TF-TF PPIs	
	STRINGv12 All	Human		All: Direct and/or Indirect TF-TF PPIs	
	STRINGv12 Physical	Human		Only Direct TF-TF PPIs	
	Main NetREm PPIN:	Human		All: Direct and/or Indirect TF-TF PPIs	• Comparison for 13 human contexts<br>• hESCs (App # 1)<br>• HSCs (App # 2)<br>• hPSCs (App # 5)

	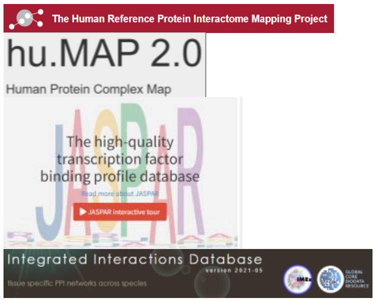				• Pooled SCs (App #8)<br>• 4 CNS cell types (App # 9)<br>• 62 immune cell subtypes for 15 TGs (App # 10)<br>• mSCs and nmSCs (App # 11)<br>• AD versus Control for 8 glial/neuronal cell-types (App # 12)
	PBMC STRINGdb V11 Network	Human		All: Direct and/or Indirect TF-TF PPIs	9 PBMC cell-types (App # 7)
	STRINGv11 All	Mouse		All: Direct and/or Indirect TF-TF PPIs	• mESCs (App # 3)<br>• mDCs (App # 4)
	STRINGv12 All	Mouse		All: Direct and/or Indirect TF-TF PPIs	• mESCs (App # 3)<br>• mDCs (App # 4)
	STRINGv12 All	Yeast		All: Direct and/or Indirect TF-TF PPIs	Budding yeast cells (App # 6)

Section §6: Overview of Building out Fully-Connected Comprehensive TF-TF Input PPINs for NetREm: W

Transcription Factors (TFs) are types of proteins. Thus TF-TF interactions are a subset of the PPIs that are measured. Hence, in our real-world applications, we will filter the final comprehensive PPIN for the respective organism to include only PPI links among the relevant TFs. That is, the comprehensive TF-TF PPINs are subnetworks of final comprehensive

PPINs, and they refer to direct and/or indirect TF-TF interactions with existing prior knowledge predictions to support them (i.e. $w > 0.01$).

When we run NetREm, we assign a default edge weight of $\eta = 0.01$ to any TF-TF edges that are missing in the TF-TF PPIN $W^{(0)}$ for the organism (i.e. every TF has a weighted pairwise link to every TF whether that link is known or potentially artificial); we add these artificial TF-TF links to help ensure a fully-connected PPI network for NetREm's regularized regression (for numerical stability and more accurate results) and guide the discovery of novel TF-TF coordination links. That is, we run NetREm on our final fully-connected comprehensive TF-TF PPIN where all TFs have pairwise links to each other with weight $\eta = 0.01 \leq w \leq 1$. Further, we note that some of the novel links may not have been in the original input PPIN (i.e. are completely missing), and other novel links may have been in the original PPIN but were filtered out as they had a score below our desired minimum weight $\eta = 0.01$.

§6.1 Fully-Connected Comprehensive TF-TF PPI Networks (PPINs) in Humans

Organism: Humans (Homo sapiens)

We apply the final comprehensive fully-connected human TF-TF PPI networks to predict TF-TF coordination networks and TF-TG regulatory networks for our human applications (#1, 2, 5, 7-11). In all of these applications, we use our Main NetREm PPIN . For the Schwann cell and Alzheimer's disease (AD) applications (#11 and 12, respectively), we incorporate prior GRN knowledge from multiomics data, such that the number and set of N candidate TFs may differ across TGs in each cell-type.

Thus, we only provide main metrics related to fully-connected TF-TF networks for our hESCs, HSCs and hPSCs applications (#1 and 2 and 7, respectively), which we run NetREm for in the absence of prior knowledge. In hESCs, we have the same $N = \mathcal{N} = 207$ (or $N = 206$ if TF is a TG) candidate TFs fixed for each TG. For 1,235 of 1,250 TGs where the TG is not a TF (i.e. $N = 207$), we have $\frac{(207)(206)}{2} = 21{,}321$ TF-TF edges (corresponding to 42,642 rows) in our fully-connected TF-TF PPI network, comprising 10,777 known and 10,544 artificial edges. Similarly, in HSCs, when the TG is not a TF (i.e. $N = \mathcal{N} = 178$), we will have 15,753 TF-TF edges in our fully-connected TF-TF PPI network, comprising 8,298 known and 7,455 artificial edges.

13 human contexts: We note that there are some input PPINs that are binary (dummy) with no weights. For these binary PPINs we assign a score of 1 to all links described in the network; the missing pairwise links will be assigned a default edge weight of 0.01.

Human PPIN Data Source	Network Type	Initial Input TF-TF PPIN (links considered to be known: $w > 1$): $W^{(0)}$	Final Preprocessed, Fully-connected PPIN: W
Barabasi PPIN	dummy (binary)	known links all have weights with $w = 1$	• 2 weight values: [0.01 and 1].<br>• Known links: weights with $w = 1$.<br>• Unknown links: weights with $w = 0.01$
Contextual IID			
Final Interactome			
HuRI_HI Union			
Multiscale Interactome			
HIPPIE	continuous	known links all have weights $0.01 < w \leq 1$	• Weight values w between 0.01 and 1.<br>• Known links: weights with $0.01 < w \leq 1$.<br>• Unknown links: weights with $w = 0.01$
HuMAP2			
Max SAINT Score PPIN			
Main NetREm PPIN			
STRINGv11 All			

STRING v11 Physical			
STRINGv12 All			
STRING v12 Physical			

§6.2 Fully-Connected Comprehensive TF-TF PPI Networks (PPINs) in Mouse

Organism: Mouse (Mus Musculus)

We apply the final comprehensive fully-connected mouse TF-TF PPI networks to predict TF-TF coordination networks and TF-TG regulatory networks in mouse ESCs, where we have the same $N = \mathcal{N} = 195$ (or $N = 194$ if the TF is a TG) candidate TFs fixed for each TG. In the cases where the TG is not a TF (i.e. $N = 195$), we will have 18,915 TF-TF edges (corresponding to $(\mathcal{N})(\mathcal{N} - 1) = 37{,}830$ rows) in our fully-connected TF-TF PPI network, comprising known (v11: 5,044; v12: 6,856) and artificial (v11: 13,871; v12: 12,059) TF-TF edges. Both networks lack any known interactions for 2 orphaned and singleton TFs (AES and STRA13) with other TFs. There are 749 out of 5,044 edges in the v11 network that are not found in the v12 release and may have been removed as potential False Positives. The removal of edges from v11 to v12 networks may be due to many factors. As new experimental data becomes available and existing data is re-evaluated, previously identified PPIs might be reclassified. Some interactions may have been based on data that, upon further scrutiny, is now found to be inaccurate or not reproducible, leading to their removal; moreover, PPI databases may update their criteria for including PPIs to reflect the latest in bioinformatics research and protein interaction validation methodologies. This might involve higher thresholds for confidence scores, leading to the exclusion of lower-confidence interactions that were included in previous versions. Similarly, 2,561 out of the 6,856 edges in the v12 network are relatively recent discoveries as they are not in the v11 network. In fact, there are 25 singleton TFs in the v11 network that have known interactions in the v12 network.

In mDCs, we have $N = N^* = 93$ (or $N = 92$ if the TF is a TG) candidate TFs fixed for each TG. In the cases where the TG is not a TF (i.e. $N = 93$), we will have 4,278 TF-TF edges (corresponding to 8,556 rows) in our fully-connected TF-TF PPI network, comprising known (v11: 863; v12: 3,110) and artificial (v11: 3,415; v12: 1,168) TF-TF edges. We run NetREm using the older v11 TF-TF PPI network to predict TF-TF coordination scores and gauge how effective the predictions are in terms of predicting future knowledge (i.e. links known in the v12 TF-TF PPI network).

§6.3 Fully-Connected Comprehensive TF-TF PPI Networks (PPINs) in Yeast

In yeast, we have $N = N^* = 280$ (or $N = 279$ if the TF is a TG) candidate TFs fixed for each TG. In the cases where the TG is not a TF (i.e. $N = 280$), we will have 39,340 TF-TF edges (corresponding to rows) in our fully-connected TF-TF PPI network, comprising known (v12 network: 14,177) and artificial (v12 network: 25,161) TF-TF edges.

Section §7: Prior Knowledge on Gene Regulation in Applications 10 and 11

Please note that we utilize R Statistical Software and Bioconductor packages to derive several databases and tools that are used in this analysis.

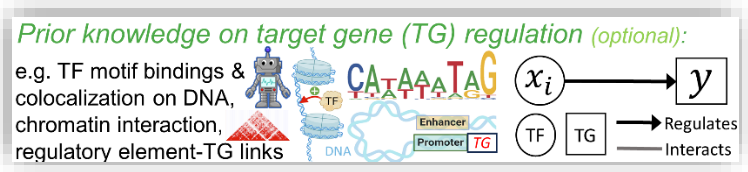

Prior gene regulatory network (GRN) knowledge on gene regulation (overview)

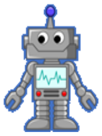

In human applications 11 (Schwann cells (SCs)) and 12 (Alzheimer's disease (AD)), we build and use prior reference GRN knowledge that subsets N biologically-promising candidate TFs for each TG in the cell-type (N can differ across TGs). Recent data enables the construction of prior GRNs for various cell-types in the human body (e.g. (Zhang et al. 2021) uncovers regions of open chromatin in 222 distinct human cell-types). To

construct prior candidate TF-Regulatory Element (RE)-TG regulatory links (prior reference GRNs), we use a multi-step approach based on available multi-omics data (e.g. TF binding predictions, chromatin accessibility). Incorporating multi-omics data can lead to more biologically meaningful and potentially truer cell-type TF-TG regulatory networks(Badia-i-Mompel et al. 2023). Such sources help determine N candidate TFs for each TG, whose expression levels define our input X when running NetREm for that TG. We provide step-by-step details and explanations for these 4 steps as a potential guide:

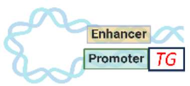

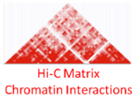

__1. Mapping cell-type REs to TGs they may be associated with:__ We identify raw scATAC-seq peaks (regions of open and accessible DNA chromatin) in adult cell-types (Zhang et al. 2021) relevant to our study. We obtain various annotated databases of RE to TG links. Next, we map these non-coding scATAC-seq peaks to TGs by overlapping them with annotated RE-TG links. Thus, we obtain peak-to-TG predicted links (annotated scATAC-seq regions mapped to respective TGs as potential cell-type REs for those TGs), our RE–TG links. By focusing on cell-type chromatin accessibility, we consider TGs open to transcription in that cell-type as well as regulatory regions where TFs may potentially bind to regulate them, which is important when predicting TF-TG links (Badia-i-Mompel et al. 2023).

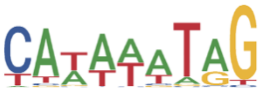

__2. Motif-based GRN identifies potential TFs binding to REs to regulate their TGs:__ We predict TF binding using motif matcher algorithm tools (motifmatchr (Schep et. al 2023) and monaLisa (Machlab et al. 2022) along with various Position Weight Matrix (PWM) databases) to scan along the RE regions. In general, we assume a given motif binds to a single TF motif at a time. The only exception to this is when we have dimeric transcription factors (e.g. AP-1, JUN/FOS), which can be heterodimers of 2 different TFs. We predict TFs in this motif-based GRN recognize and bind to their sequence-specific motifs on TFBSs of various REs (e.g. promoters, enhancers, eQTL-based regions), thereby regulating expression of their respective TGs. This results in a list of motif-GRN TFs for each TG. This may reveal paralogs and TFs recognizing the same motif, competing for TFBSs (Berenson et al. 2023).

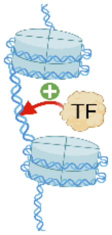

__3. Pruning Motif-based GRN TFs:__ We chop motif-GRN TFs with relatively lower motif-matching scores (based on output from motif matcher algorithm tools) and remove TFs with relatively low expression in training expression data (and/or external data for same cell-type).

__4. TF Augmentation Step (Consider Potential Colocalization among TFs):__ Nonetheless, approaches that solely rely on PWMs to model motifs and predict candidate TFs for TGs suffer from drawbacks and limitations (e.g. assume independence of positions within a binding motif (Siddharthan 2010); many TF binding motifs have low specificity (Badia-i-Mompel et al. 2023) as many TFs share similar binding motifs (Ali et al. 2022)) to represent TFBSs such that most ChIP-seq peaks for TFs miss relevant PWM hits for corresponding TFs (Tsukanov et al. 2022). Observations of a TF binding to a TG may not always imply the TF regulates the TG as TFs cooperate with other molecules like TFs to regulate the TG (Badia-i-Mompel et al. 2023). TF regulatory mechanisms involve other factors besides the accessibility of TF binding motifs (Kamimoto et al. 2023) (e.g. pioneer TFs). Hence, we implement an augmentation step, adding TFs based on various criteria (e.g. potential colocalization with our pruned TFs, TFs forming complexes with each other and/or exhibiting strong molecular functional similarity), checking these TF pairs have known PPI links (and are not artificial). Ultimately, this results in N customized, candidate TFs for a given TG.

Goal: Deriving Prior Gene Regulatory Network (GRN) for each Target Gene (TG)

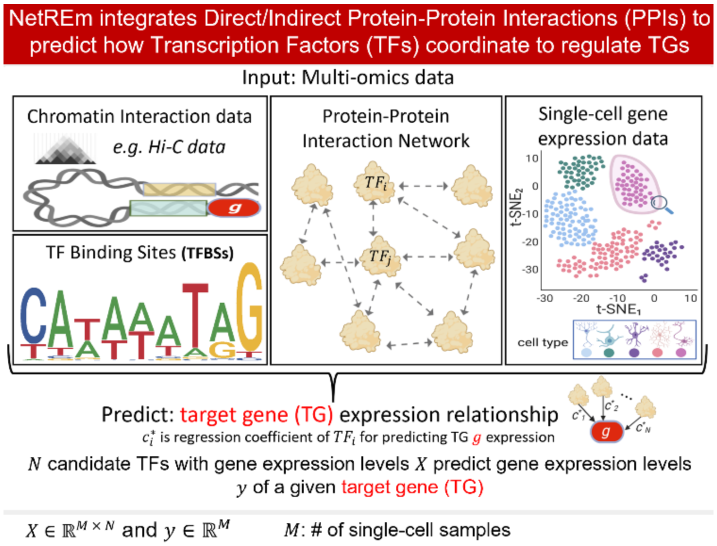

Please note that the approaches mentioned below are examples of how to derive a prior reference GRN for the input step for NetREm. Performing this step is optional but this initial feature selection may lead to more biologically relevant results. In addition, the steps taken below may be adapted and adjusted based on personal preferences or application needs. In short, there are many ways and various existing tools that can be utilized to find a list of N candidate Transcription Factor (TF) # for each target gene (TG). Each TG may have a different final number of candidate TFs, so N may differ across TGs.

§7.0 Organizing Input Data

§7.0A Raw single-cell ATAC-seq regions

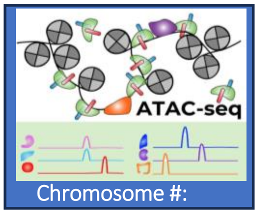

In this study, we obtain raw single-cell ATAC-seq peaks for 222 cell types from (Zhang et al. 2021), which correspond to regions of open and accessible DNA chromatin (euchromatin) in non-coding DNA. We focus on adult cell-types that are relevant to our study.

Please note that the figure on the left is adapted from the original figure made by Dr. Ryan Corces.

§7.0B Determining cell-type-specific single-cell ATAC-seq regions

The following details how we determine cell-type-specific single-cell ATAC-seq peaks (regions of open and accessible DNA chromatin) based on the raw scATAC-seq data from **§7.0A**. Below, we explain our process for Schwann cells (SCs), which we applied for the other adult cell-types for our Alzheimer's disease (AD) application. We note that this process can be applied to other cell-types of interest, based on the data that is available.

Schwann cells (SCs)

We focus on the raw Schwann cell (SC) ATAC-seq file, which has 88,175 rows corresponding to 73,114 unique peaks (chrom #: start position - stop position). Our goal was to find ATAC-seq peaks that are mainly specific to Schwann cells and/or potentially limited to up to these 6 others closely-related cell-types: fetal Schwann cells, fetal enteric glia, fetal Oligo Progenitor 2, adult melanocytes, adult oligodendrocytes, adult oligodendrocyte progenitor cells (OPCs).

To determine any and all overlaps between Schwann cell ATAC-seq peaks and the peaks of each of the other 221 cell types, we employed the GRanges (Lawrence et. al 2013) package in R. This allowed for efficient identification of the shared genomic regions between the Schwann cell peaks and the peaks of the respective cell types. Thus, for each of the 221 cell types, we overlapped their respective raw ATAC-seq peaks with those of the general SC raw ATAC-seq peaks.

For a given cell type, we use the 2-sided Fisher's exact test (via fisher.exact in R) to assess the statistical significance of each overlap identified between a SC ATAC-seq peak and that cell type's ATAC-seq peak. This was preferable to defining a strict overlap cutoff number of bases since raw ATAC-seq peaks had differing widths. By comparing the observed overlap with the expected overlap under independence (if the overlap is not statistically significant), Fisher's exact test provided a p-value indicating the likelihood of obtaining such an overlap by chance (i.e. is the observed overlap higher or lower than expected). To account for multiple testing, a Benjamini-Hochberg multiple test correction was applied. This correction method adjusted the p-values for each overlap to control the false discovery rate. Only overlaps with an adjusted p-value (p_adj) < 0.05 are considered statistically significant between SCs and the respective cell type.

Those significant peaks were then annotated as overlaps between SCs and that cell type and the non-significant overlaps (p_adj > 5%) were discarded. We repeated this process for all 221 cell types.

Ultimately, SC-type-specific scATAC-seq peaks would only be found in the raw SCs and did not have any statistically significant overlaps with any of the ATAC-seq peaks for the other 221 cell types. We found 1,736 such peaks. Nonetheless, there are strong shared biological signals between Schwann cells and those 6 cell types mentioned above. Hence, we expand our scope and loosened our criteria (to uncover more Schwann cell ATAC-seq peaks) to consider Schwann cell-type-specific scATAC-seq peaks as those that may significantly overlap with any of those 6 cell-types only and do not overlap with any of the other 215 cell types. That adds 1,052 peaks. Finally, we uncover 2,788 such Schwann cell ATAC-seq peaks.

We perform this analysis for the 8 other cell-types for Alzheimer's disease (AD):

- For Endothelial (Endo.) Blood Brain Barrier (BBB) cells, we consider similar cell-types: Endo. exocrine, endo. general 1-3, endo. myocardial, fetal endo. general 1 – 3, fetal endo. hepatic 1-2, fetal endo. placental.
- For Inhibitory Neurons, we consider similar cell-types as fetal Inhibitory neurons 1 to 5. For excitatory neurons, we consider similar cell-types as fetal excitatory neurons 1 to 12.
- For Oligodendrocyte Precursor Cells (OPCs), we consider fetal oligo progenitor 2 as a similar cell-type.
- Oligodendrocytes: we consider Fetal Oligo progenitor 2, Oligo Precursor, fetal Schwann general, Schwann general.
- For Astrocytes, we consider fetal Astrocytes 1 to 5.
- For Microglia, we consider fetal Macrophage general 1 to 4, fetal macrophage hepatic 1 to 3, fetal Macrophage placental, Macrophage general or alveolar, Macrophage general.
- For Pericytes, we consider: Pericyte muscularis and cardiac pericyte 1 to 4.

§7.0C Obtaining peak-to-TG predicted links

To construct prior candidate TF-Regulatory Element- (RE)-TG regulatory links (prior reference GRNs), we use a multi-step approach based on the available multi-omics data. We start by identifying raw scATAC-seq peaks from §7.0A. We map these peaks to TGs by overlapping them with annotated RE to TG links. This process entails using various approaches to obtain these peak-to-TG predicted links, which we explain below as a potential guide.

1. We gather a database of cis-Candidate REs (cCREs) (Zhang et al. 2021) that are already mapped to TGs based on genomic distance limits(Badia-i-Mompel et al. 2023) and previously filtered based on their Activity by Contact (ABC) scores. We use all interacting cCREs for that cell-type (e.g. promoter-promoter, promoter proximal – promoter, distal (enhancer) – promoter) that are present in adult tissues. These various types of chromatin interactions may illuminate different aspects of coordination among TFs; for instance, studies observe that since promoter-promoter interactions may cluster several TGs, they can provide unparalleled information on TF coordination, especially among TGs that may be context-specific (e.g. tissue- or cell-type- specific) or involved in cellular housekeeping processes (Li et al. 2012). We thus learn the following potential regulatory elements for each TG: gene promoters, cCREs (promoter, promoter-proximal, and/or distal).

Please note that this figure below is adapted from (Badia-i-Mompel et al. 2023) and Dr. Ryan Corces.

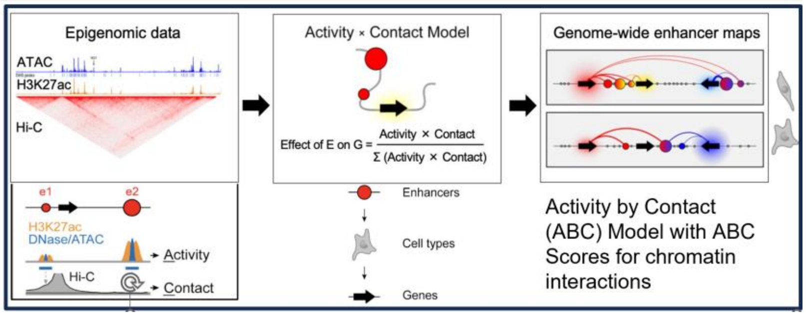

2. We utilize TxDb.Hsapiens.UCSC.hg38.knownGene (Team BC, Maintainer BP, et. al 2019; Zhou et. al 2018 2018) to define default promoter-only regions and then overlapping those with the scATAC-seq peaks.
3. For Oligo, Mic, and Neurons (ExNs and/or InNs), we also utilize Plaq-seq data(Nott et al. 2019) on promoter-enhancer interactomes and enhancer regions in hg19. We run scGRNom(Jin et al. 2021) Step 1 (scGRNom_interaction) on this epigenomic data to predict all possible interactions between enhancers and promoters and annotate them with the associated TGs. We use rtracklayer (Lawrence et al. 2009) and the hg19tohg38.over.chain file from UCSC (Karolchik et al. 2009) to perform liftover(2021) to map the associated enhancer and promoter regions to hg38 human genomic coordinates. We use these annotated interaction enhancer and promoter regions as Plaq-seq-based regulatory regions for the respective cell-types.

We overlap our raw scATAC-seq peaks with these annotated regulatory elements to TG links to associate the scATAC-seq peaks with their potential TGs.

4. Then, we look at the remaining scATAC-seq peaks that did not map to any regulatory elements and attempt to characterize them via expression quantitative trait (eQTL) data that links single nucleotide polymorphisms (SNPs) to TGs whose expression levels they impact. We use GTEx v8 Tibial Nerve eQTL data (the left peripheral tibial nerve from the gastrocnemius region) for SCs (THE GTEX CONSORTIUM 2020) and eQTL data from Byrois et al. for brain cells for our AD application (Bryois et al. 2022).

§7.0D Position Weighted Matrices (PWMs) of TF Binding Motifs

Different TF binding motif databases have different coverages of TFs(Badia-i-Mompel et al. 2023) and diverse annotated binding sites(Skok Gibbs et al. 2022). We obtain PWMs for humans from R Bioconductor (Gentleman et al. 2004) databases. Each motif is specific to 1 TF and a TF may have many motifs. When running motifmatchr (Schep et. al 2023) to search for motif matches for TFs, we use JASPAR2022 (Castro-Mondragon et al. 2022) for humans (species = 9606) and convert Position Frequency Matrices (PFMs) to PWMs using TFBSTools (toPWM() function, type = "prob"(Tan and Lenhard 2016)). We have 949 PWMs corresponding to 680 TFs. To run monaLisa (Machlab et al. 2022) for motif analysis and TF binding prediction, we use a comprehensive database of 8,903 PWMs (last update: March 4, 2022). We use MotifDb (Shannon and Richards 2023) for "hsapiens" to obtain 6,086 PWMs and motifbreakR's motifbreakR_motif data (Coetzee et al. 2015) to add 2,817 PWMs. We remove redundant motifs (found in both sources) for TFs when running monaLisa for our analysis.

Since DNA strands are antiparallel, they run in opposite directions where one strand runs 5' to 3' while the complementary strand runs 3' to 5'. A palindrome in DNA refers to a sequence of base pairs that reads the same in the 5' to 3' direction on one strand as it does in the 5' to 3' direction on the complementary strand when both strands are aligned. In regulatory DNA, palindromes are inverted sequence repeats (read the same from 5' to 3' on complementary DNA strands). These cis-regulatory palindromic motifs can be bound, cooperatively, by homodimers (same TF: $TF_i :: TF_i$) or by heterodimers (different TFs: $TF_i :: TF_j$) to mediate cell type-specific combinatorial gene regulation. That is, a sequence-specific TF can bind to the palindromic motif on opposing DNA strands as a homodimer, where 1 TF binds with its DNA-binding domain to the left and another copy of the TF binds to the right half-site of the motif(Datta and Rister 2022). Thus, if TFs bind as homodimers, then the motif in PWMs will reflect this behavior. For instance, JUN-family TFs have a palindromic AP-1 motif (TGAG/CTCA), which enables them to form homodimers with other JUN TFs or form heterodimers with FOS family TFs (Datta and Rister 2022). Our current approach looks at coordination among different TFs. To incorporate homodimers in our analysis, we need robust prior knowledge on homodimeric TF-TF interactions and may need to adjust NetREm to handle self-interactions.

§7.1 Constructing Motif-based Gene Regulatory Networks (GRNs)

Essentially, we predict Transcription Factor (TF) Binding Sites (TFBSs) for TFs. We utilize the peak-to-TG predicted links for each target gene (TG) from §7.0C. Then, we predict candidate TFs that may bind to these regulatory regions for the TG based on their sequence-specific TFBS motifs. To this end, we utilize 2 different tools to construct these motif-based GRNs: monaLisa and scGRNom.

§7.1A Utilizing monaLisa

To predict strong TF binding to the regulatory regions for TGs, we run monaLisa with a very stringent minimum motif matching score of 8 with the "matchPWM" method. We use our comprehensive Position Weighted Matrix (PWM) of 8,903 PWMs. For TF binding to regulatory regions, we use method = "matchPWM" and min.score of 8 to identify more TFs binding to the regulatory element (RE) for each of the cell-type-specific REs that we determined. We use our more comprehensive input Position Weighted Matrix (PWM). The monaLisa tool returns TFs predicted to strongly bind to each given regulatory region along with their scores and predicted positions in the regulatory region. The monaLisa tool returns TFs predicted to strongly bind to each given regulatory region along with their scores and predicted positions in the regulatory region. This is our monaLisa-motif-based GRN that links TFs to REs (and specific positions within them) to the target genes (TGs). For instance, these regulatory elements have chromosome #: start position – end position.

§7.1B Adapting scGRNom

We use the JASPAR2022 database (Castro-Mondragon et al. 2022) and hg38 human reference genome. We adapt the scGRNom(Jin et al. 2021) pipeline for building cell-type gene regulatory networks (GRNs) such that we run scGRNom_getTF (Step 2) on all the regulatory regions for the TG, including promoters, eQTL-based regions, proximal promoters, distal (enhancers). We use all the defaults mentioned by scGRNom tool to predict TFs regulating the TG based on scGRNom. We output a reference scGRNom-motif-based GRN that links TFs to regulatory elements to TGs.

§7.1C Combining the motif-based GRNs

Thus, for a given Target Gene (TG), we run monaLisa in §7.1A, to identify a pool of TFs that are predicted to regulate this TG based on our monaLisa binding prediction (using the comprehensive Position Weighted Matrix (PWM)) run on all regulatory elements we have mapped to this TG.
We also find the list of TFs in §7.1B predicted by scGRNom Step 2 (on JASPAR2022 database (Castro-Mondragon et al. 2022)) to regulate this TG based on the regulatory elements that have been mapped to this TG.

Then, we look at all the TFs that are identified in §7.1A and/or §7.1B, and we add in any TFs that they may be found to form a protein complex with (i.e. score in input Protein-Protein Interaction (PPI) network of 1). For instance, if FOS is identified as one of the potential TFs for the TG (based on §7.1A and/or §7.1B), then we ensure that JUN is also added to the list of TFs (if it is not already in the list of TFs), since FOS and JUN are known to form a complex FOS::JUN.

Thus, §7.1C results in a pooled list of TFs from §7.1A and/or §7.1B and the associated TFs that they form known TF complexes with. Going forward, please note we explain this process for a given target gene (TG) in the cell-type (or tissue-type) for the particular context (e.g. control, disease). This process will be applied for each TG in the cell-type.

Please note that this figure below is adapted from (Jin et al. 2021)

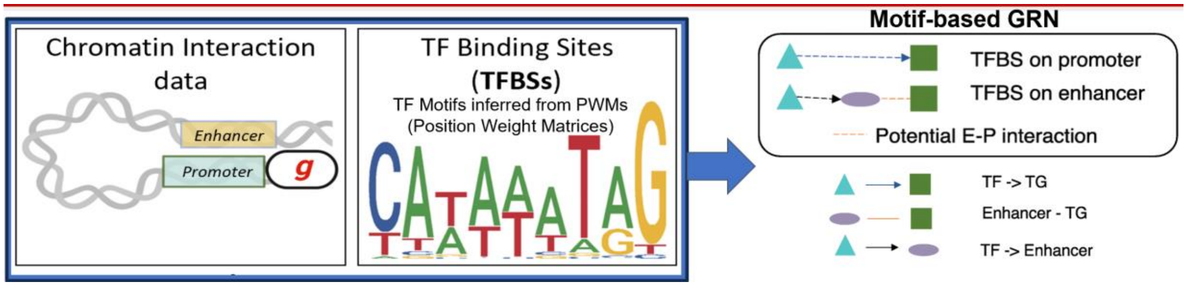

For instance, please note this is the motif-based GRN for human Schwann cells:

:) motif-based Gene Regulatory Network (GRN) for human Schwann cells (SCs)

	chrom	start	end	final RE: raw scATAC-seq_peak	TF	TG	source	cell_type	TF_TG	Region	original cis-candidate cCRE	seqNum
**0**	chrY	2934936	2936248	chrY:2934936-2936248	RARA	ZFY	scgrnom	Schwann_Cells	RARA_ZFY	TxDB_hg38_promoter	chrY:2933781-2935780	73068
**1**	chrY	2934936	2936248	chrY:2934936-2936248	RXRA	ZFY	scgrnom	Schwann_Cells	RXRA_ZFY	TxDB_hg38_promoter	chrY:2933781-2935780	73068
**2**	chrY	2934936	2936248	chrY:2934936-2936248	EGR1	ZFY	scgrnom	Schwann_Cells	EGR1_ZFY	TxDB_hg38_promoter	chrY:2933781-2935780	73068
**3**	chrY	2934936	2936248	chrY:2934936-2936248	MEF2A	ZFY	scgrnom	Schwann_Cells	MEF2A_ZFY	TxDB_hg38_promoter	chrY:2933781-2935780	73068
**4**	chrY	2934936	2936248	chrY:2934936-2936248	YY1	ZFY	scgrnom	Schwann_Cells	YY1_ZFY	TxDB_hg38_promoter	chrY:2933781-2935780	73068
**...**	...	...	...	...	...	...	...	...	...	...	...	...
**10976661**	chr10	3067852	3069214	chr10:3067852-3069214	SIN3A	PFKP	monalisa	Schwann_Cells	SIN3A_PFKP	cCRE Promoter Proximal	chr10:3067963-3068363	7087
**10976662**	chr10	3067852	3069214	chr10:3067852-3069214	SIN3A	PFKP	monalisa	Schwann_Cells	SIN3A_PFKP	cCRE Promoter	chr10:3068411-3068811	7087
**10976663**	chr10	3067852	3069214	chr10:3067852-3069214	SIN3A	PFKP	monalisa	Schwann_Cells	SIN3A_PFKP	cCRE Promoter Proximal	chr10:3068940-3069340	7087
**10976664**	chr10	3067852	3069214	chr10:3067852-3069214	SIN3A	PFKP	monalisa	Schwann_Cells	SIN3A_PFKP	Gene Promoter	chr10:3067626-3069626	7087
**10976665**	chr10	3067852	3069214	chr10:3067852-3069214	SIN3A	PFKP	monalisa	Schwann_Cells	SIN3A_PFKP	Gene Promoter	chr10:3066547-3068547	7087

12321042 rows × 13 columns

§7.2 Pruning TFs down based on expression levels

This is the reduction stage, where we will prune down the list of TFs that we retrieve in §7.1C, for the TG, based on their relative expression gene expression levels. That is, key drivers of cell-type-specific gene regulation ought to have relatively meaningful expression levels in the cell (§7.2A-§7.2B).

§7.2A Removing TFs based on global criteria for all genes in the dataset

We note that we will remove Transcription Factors (TFs) from §7.1C that have a relatively lower level of gene expression in the cell-type (or tissue-type) compared with other genes.

Otherwise, we will use our training gene expression dataset for this part (not our testing data). If we have another gene expression dataset available for a similar context (e.g. cell-type or tissue-type), we can use that external dataset as well.

That is, we will look at that (training) gene expression data and prune out TFs based on their expression levels relative to the expression levels of all other genes in the dataset. Below, we detail how we do this for Schwann cells (where we do have external gene expression data we can use) and for Alzheimer's disease (AD) versus controls. For the AD application, since we only have external data (Lake et. al) for 4 out of the 8 cell-types, we opt not to use this external dataset, to help keep the process consistent for the 8 cell-types.

Schwann Cells (Application 11)

Please note we also utilize raw RNA-seq data for mSCs and nmSCs in the Sciatic Nerves (SN) of 4 mice from GSE137947 (Gerber et. al 2021), which comprises the SN Atlas (SNAT). We use g:Profiler(Reimand et al. 2007) orthologous functionality to help map the mouse (mus musculus) gene IDs to 16,954 human (homo sapiens) gene names.

- **Step 1:** We identify the 40th percentile for all 17,048 genes in the training gene expression dataset in the Dorsal Root Ganglion (DRG).
  - In human mSCs, this 40% threshold is: 2.9e-2. Here, 6,819 genes are below this threshold.
  - In human nmSCs, this 40% threshold is: 2.44e-2. Here, 6,819 genes are below this threshold.
- **Step 2:** We identify the 30th percentile for all 16,954 genes in the respective SNAT dataset for the mouse (i.e. 4 mSCs for mSCs application or 4 nmSCs for the nmSCs application).
  - In mouse mSCs, this 30% threshold is: 14.75. Here, 5113 genes are below this threshold (3,105 of these are also lowly-expressed in corresponding human data).
  - In mouse nmSCs, this 30% threshold is: 25.25. Here, 5,096 genes are below this threshold (3,167 of these are also lowly-expressed in corresponding human data).
- **Step 3:** We remove any genes found below the threshold in Step 1 and/or Step 2 from our list of TFs (but will still retain those genes as TGs). This filtering is meant to remove TFs that have low gene expression levels in the training

data and/or mouse data from the pool of potential candidate TFs (to guide us in our feature selection of TFs for the TG). Please note that we do not filter the list of TGs based on expression levels.

- In mSCs, we flag 8,827 genes with low expression. Any TFs in this list of lowly-expressed genes will be removed.
- In nmSCs, we flag 8,748 genes with low expression. Any TFs in this list of lowly-expressed genes will be removed.

Alzheimer's disease (AD) (Application 12):

Please note that we adapt our steps to be closely-related to those for Schwann cells (SCs).

- **Step 1:** We identify the 40th percentile for all 17,926 genes in the training gene expression dataset for that cell-type and/or condition (AD versus Controls).
- **Step 2:** We will remove any genes found below the threshold in Step 1 from our list of TFs (but will still retain those genes as TGs). This filtering is meant to remove TFs that have low expression levels in the training data and/or mouse data from the pool of potential candidate TFs (to guide us in our feature selection of TFs for the TG). Please note that we do not filter the list of TGs based on expression levels.

§7.2B Removing TFs based on relative gene expression levels for remaining TFs

After we perform **§7.2A**, we essentially have a reduced list of TFs from **§7.1C**, which meet a minimum percentile of relative gene expression levels in the cell-type. The list of TFs to remove based on the criteria in **§7.2A** remains the same for each TG for the cell-type (since that filtering is at a global level for all genes in the (training) dataset).

Then, we perform an additional filtering step to remove TFs in **§7.2A** that have lower expression levels relative to the other TFs in **§7.2A**. In this way, this is a more tailored filter applied based on the specific TFs that remain after **§7.2A**.

We utilize the training gene expression data for all pooled TFs in **§7.2A** and determine the TFs that have relative gene expression above the 40th percentile (compared with the other pooled TFs). Thus, we determine the top 60th percentile of TFs (out of the pooled) that we should keep, filtering out the TFs from **§7.2A** to only include these TFs with relatively high expression**.**

§7.3 Pruning TFs down based on motif-score criteria

We also will use motif-score criteria to help us prune down the list of TFs for the TG.

§7.3A Identifying common TFs in both motif-based GRNs

We keep all the TFs found in common in **§7.1A** (scGRNom-based) and **§7.1B** (filtered monaLisa) as those are highly-confident TFs. That is, these TFs were found as viable candidate TFs for the TG using 2 separate methods; this instills confidence that these TFs could be key to consider.

§7.3B Filtering based on metrics for the monaLisa motif-based GRN

We will filter the list of TFs found in **§7.2B** based on additional criteria, which we will mention in these upcoming steps. Since our monaLisa motif-based GRN (**§7.1A**) tends to be more extensive, we will apply filters to the TFs in **§7.2B** based on their relative performance for various motif-based metrics in **§7.1A.**

In **§7.0B**, we identified a list of raw scATAC-seq peaks that are cell-type-specific. Then, in **§7.0C**, we identified peak-to-TG links (i.e. raw scATAC-seq peaks mapped to the TG via regulatory element relationships). Both approaches are different ways to annotate and glean information about the raw scATAC-seq peaks for the cell-type. For the given TG, there may be overlaps between the peaks in **§7.0B** with those in **§7.0C.** Here, we determine if the TG contains any peaks (in **§7.0C**) that are also cell-type-specific (**§7.0B**). Then, we will retain TFs based on various criteria.

- *If we found that the TG has at least 1 cell-type-specific scATAC-seq peak:* If the TG does have scATAC-seq peaks that are cell-type specific (i.e. we identified cell-type-specific peaks for the TG), then we will adjust our criteria to focus more on TFs that bind to the cell-type-specific scATAC-seq regulatory peaks as those TFs may help drive cell-type-specific gene expression levels. The minimum percentile for motif-matching scores that we use to filter out the TFs predicted by monaLisa is: *min_monaLisa_percentile* = 80% if the TG has cell-type-specific peaks.

- *If we found NO cell-type-specific scATAC-seq peak for the TG:* The minimum percentile for motif-matching scores that we use to filter out the TFs predicted by monaLisa is: *min_monaLisa_percentile* = 75% if we could not identify any cell-type-specific peaks for the TG.

All TFs need to fall in groups A, B, and C, which are global for the TG.

 - **A**: We find which TFs are bound to at least the *min_monaLisa_percentile* # of all regulatory regions (whether cell-type-specific or not) for that TG
 - **B**: Which TFs have a median motif-matching score that is at least *min_monaLisa_percentile* of that for the other TFs
 - **C**: The same as B, but focused on the average motif matching score

If the TG has cell-type specific peaks, we have additional criteria, for the genes to fall into (so they need to fall in groups A, B, C, D, E, and F, and we have a stricter *min_monaLisa_percentile*). The groups D to F are more tailored for the cell-type-specific regulatory regions for the TG.

 - **D**: We find which TFs are bound to at least the *min_monaLisa_percentile* # of only the cell-type-specific regulatory regions for that TG
 - **E**: Which TFs have a median motif-matching score for cell-type-specific regulatory regions for TG that is at least *min_monaLisa_percentile* of that for other TFs that bind to cell-type-specific regulatory regions for TG.
 - **F**: The same as E, but focused on the average motif matching score

Thus, **§7.3B** results in a set of TFs from monaLisa filtered down from that in **§7.2B.**

§7.3C Strong list of candidate TFs based on binding information

At this point, the TFs we identify for the TG thus are found in **§7.3A** (common TF in both scGRNom-based GRN and monaLisa-based GRN) and/or **§7.3B** (strong metrics in monaLisa-based GRN relative to other TFs in monaLisa-based GRN for the TG). We pool together the list of TFs from **§7.3A** and/or **§7.3B** as a strong list of potential candidate TFs based on binding information.

These steps so far represent our improvements in determining potential candidate TFs for the TG based on TF binding profiles. Our enhancements include:

- Predict TF binding for more regulatory interactions besides Distal (Enhancer) – Promoter – Gene
- Include potential "promoter-only" TGs that may lack data on regulatory interactions
- Use more comprehensive PWMs
- Integrate more tools to infer TF binding from PWMs
- Utilize more flexible pruning criteria based on motif binding scores
- Retain TFs with a minimum average gene expression level in training data

§7.4 TF Augmentation Step:

This is our expansion step, where we build on the list of TFs that are found by **§7.3C**. For each of these TFs in **§7.3C**, we will additional TFs based on various TF similarity approaches and colocalization/cobinding measures that we have derived. For each TF, we prioritize adding TFs that not only have high scores for the corresponding metric but also contain known links in the PPI (if the TF is found in the PPI); if the TF forms a known complex with other TFs, we especially focus only on the TFs that it forms a complex with and see which of those TFs have high values for that given metric. We focus on adding in TFs that are not found in **§7.3C**, so that unique TFs can be added in this step; nonetheless, it is possible that a given TF may be added in multiple times in this Step. We use these 3 approaches to identify highly-similar TFs to add to the list of TFs we have from our final motif-based GRNs.

 - **§7.4A** *Colocalization Approach 1*: we add TFs with the highest colocalization score based on monaLisa-based derivations (high Jaccard Similarity values).
 - **§7.4B** *Colocalization Approach 2:* we add TFs with the highest colocalization score based on scGRNom-based derivations (high Jaccard Similarity values).

- §7.4C *Gene Ontology Molecular Function Similarity*: add TFs with the highest similarity with that TF that are also found in the PPI.

§7.4A Determining Colocalization Approach 1:

In approach 1, we will focus on cell-type-specific scATAC-seq peaks. We utilize these cell-type-specific raw scATAC-seq peaks (from §7.0**)** for our *Colocalization among TFs* analysis approach 1). Thus, we identify scATAC-seq peaks unique for that cell-type, ensuring no significant overlap with peaks from other cell-types (Zhang et al. 2021). Then, we run the monaLisa tool to analyze the colocalization among TFs. We use method = "matchPWM" and min.score of 7.5 to identify more TFs binding to the regulatory region for each of the cell-type-specific scATAC-seq regions that we determined. We use our more comprehensive input Position Weighted Matrix (PWM) of 8,903 PWMs.

Using monaLisa(Machlab et al. 2022), we predict TF binding within these specific peaks, noting the TFs and their binding locations. The monaLisa(Machlab et al. 2022) tool returns TFs predicted to strongly bind to each given regulatory region along with their scores and predicted positions within the given regulatory region. To determine potential colocalization among a pair of TFs for each region, we determine if there is any configuration of binding positions where the 2 TFs may not overlap (or at least bind 1 base pair apart from each other). We assign a score of 1 if there exists such a configuration between those TFs for that regulatory region, else a score of 0. Then, we aggregate the number of regulatory regions for which these 2 TFs have score of 1 to determine the number of regions with colocalization among the TFs.

We note TFs that may overlap could form complexes. Nonetheless, the TF augmentation step already adds TFs that form known complexes with each other to the list of proteins. Hence, for this approach, we assume TFs that are overlapping across all possible binding configurations for the regulatory region may compete for binding to that region and likely do not exhibit cooperative behavior at that region; with that prediction, both TFs receive a 0 score for that region.

Thus, for each TF pair, a score of 1 is assigned per peak if they have non-overlapping potential TFBSs, suggesting cooperativity. Aggregate scores for all TF pairs are calculated across the peaks. Finally, we scale these pairs using a Jaccard Similarity (JS) index, with higher scores indicating greater colocalization. Here, colocalization is 2 TFs that bind within the same enhancer but not to the same motif. $JS(TF_i, TF_j) = \frac{\#\text{ of peaks with potential non-overlap in binding for } TF_i \text{ and } TF_j}{\#\text{ of peaks bound by } TF_i \frac{\text{and}}{\text{or}} TF_j}$

For each TF in §7.3C, we add TFs with highest colocalization score based on monaLisa derivations (high JS values).

§7.4B Determining Colocalization Approach 2:

In Approach 2, we will focus on all the raw scATAC-seq peaks (including the cell-type-specific, mapped, and unmapped peaks). For analyzing colocalization among TFs (Approach 2 in the main paper for *Colocalization among TFs*), we run scGRNom(Jin et al. 2021) Step 2 (with our adaptations) on all the raw single-cell ATAC-seq regions for that given cell-type to predict TFs with strong binding. We run motifmatchr (Schep et. al 2023) on all raw scATAC-seq peaks and identify TFs with strong binding for each peak. Then, we determine the JS of binding among TFs by analyzing the number of common regulatory regions (out of all raw regions in the cell-type) they share strong binding for. Next, we calculate JS between any 2 TFs as: $JS(TF_i, TF_j) = \frac{\#\text{ of peaks bound by } TF_i \text{ and } TF_j}{\#\text{ of peaks bound by } TF_i \frac{\text{and}}{\text{or}} TF_j}$

Then, we consider TFs with a high JS score as being very similar to each other (e.g. potentially sharing the same TF family of motifs) based on this derived predicted TF-DNA-binding data. For each TF in §7.3C, we add TFs with the highest colocalization score based on scGRNom-based derivations (high Jaccard Similarity values).

§7.4C Determining Colocalization Approach 4:

We employ clusterProfiler (Wu et al. 2021) to determine TF similarity based on Molecular Function (MF). Using the godata() function on the org.Hs.eg.db database (Carlson et. al 2019), we generate semantic similarity data for Gene Ontology (GO) Molecular Function (GOMF). We then apply the mgeneSim() function with default settings, utilizing the Wang measure and BMA (Best-Match Average) approach. This method helps infer genetic interactions and PPIs, as closely-related TFs often exhibit similar behavior (Yu). For each TF in §7.3C, we add TFs with the highest similarity with that TF that are also found in the PPI network.

§7.4D Combining TFs from 3 Approaches:

For each TF in **§7.3C**, we add a list of TFs based on **§7.4A-C**. We do this for all TFs in **§7.3C**. Ultimately, this results in new TFs (some may overlap with those in **§7.3C**), which we add to list of TFs in **§7.3C** to retrieve our updated list of TFs.

§7.4E Adding TF complexes:

Here, we add in TFs that are found to form physical complexes with any of the TFs in **§7.4D**. Then, we use data on known TF complexes (i.e. TF pairs with weight 1 in comprehensive input PPI network). While we had done this in **§7.1C**, we recognize that some of these TFs may have been eliminated during the pruning steps in **§7.2** and **§7.3**. This is our final list of N candidate TFs for the given TG.

Section §8 NetREm Parameters for Applications

For all of our runs of NetREm, we choose not to fit or include any y-intercept term.

§8.1 NetREm in benchmarking applications with no prior GRN information

For our benchmarking applications, please note the following NetREm hyperparameters with more details in **Table S24** regarding the # of TGs, # of cells M, fixed set of N candidate TFs for TG (held constant across all TGs for the $\mathcal{N}$ potential cel-type TFs; if the TG is also one of the $\mathcal{N}$ TFs, we just remove that self-TF (corresponding to the same TG) from the list of candidate TFs so there is no overlap between the candidate TFs and the TG → $N = \mathcal{N} - 1$ candidate TFs for the TG).

Human (hESCs) Embryonic Stem Cells (ESCs)

Data from Application 1

We use $\beta = 1$ and then vary α for each of our 6 SERGIO-simulated gene expression datasets (varying noise parameter: {30%, 60%, 90%}, varying training cells M: {70; 700}). We may use LassoCV to optimize α based on 5-fold cross validation (CV). We also try out 5 fixed values of α: 0.01, 0.025, 0.05, 0.075, 0.1. Thus, the following 6 NetREm runs (for each of the 1,250 TGs in each of the 6 gene expression datasets) are: NetREm (β = 1, α = LassoCV), NetREm (β = 1, α = 0.01), NetREm (β = 1, α = 0.025), NetREm (β = 1, α = 0.05), NetREm (β = 1, α = 0.075), NetREm (β = 1, α = 0.1). We use the comprehensive human Main NetREm PPIN that has STRINGdb V12 human PPIs and beyond (from various sources detailed in **§5.1.11**) as our input PPIN.

Human Hematopoietic Stem Cells (HSCs)

Data from Application 2

We have 4 different NetREm runs, which we do on Main NetREm PPIN (comprehensive input human PPIN) detailed from **§5.1.11**: NetREm (β = 1, α = 0.01), NetREm (β = 0.5, α = 0.01), NetREm (β = 1, α from LassoCV), NetREm (β = 10, α from LassoCV). We apply this pipeline and these 4 different runs to each of the 10,588 TGs in HSCs.

Mouse (mESCs) ESCs

Data from Application 3

We use a fixed $\beta = 1$ and α = 0.05. We mainly alter the input STRINGdb PPIN that is used for our network constraint and run NetREm with the older Version 11 network (updated between January 2019 and October 2020) and the current newest V12 PPIN (updates since July 2023). That is, we have 2 runs of NetREm for each of the 19,225 TGs: NetREm (β = 1, α = 0.05) with the older V11 STRING PPI network (**§5.2.1**) and NetREm (β = 1, α = 0.05) with the newer V12 STRING PPI network (**§5.2.2**) for the mouse organism (mus musculus).

Mouse Dendritic cells (mDCs)

Data from Application 4

We vary α for various values using 2 different versions of the STRINGdb input PPIN for mice. We apply NetREm for each of the 9,087 TGs in mDCs. Thus, we use the older PPIN V11 (**§5.2.1**) and for newer PPIN V12 (**§5.2.2**) from STRINGdb.

- Please note that we run NetREm using STRINGdb V11 for a fixed $\beta = 1$ and 8 different values of $\alpha \in$ {0.01, 0.025, 0.05, 0.075, 0.1, 0.125, 0.15, LassoCV}. We note that the α: LassoCV means that we obtain $\tilde{X}$ and $\tilde{y}$ based on $\beta = 1$ and then subsequently determine the optimal α (via Cross-Validation (CV); default: 5-fold

CV) to minimize this objective equation (*Equation 4* in the main manuscript): $c^* = \underset{c}{\operatorname{argmin}} \tilde{f}(c) = \frac{1}{2N}||\tilde{y} - \tilde{X}c||^2 + \alpha||c||_1$. Thus, there are these 8 different runs of NetREm with STRINGdb V11: NetREm ($\beta = 1, \alpha = 0.01$), NetREm ($\beta = 1, \alpha = 0.025$), NetREm ($\beta = 1, \alpha = 0.05$), NetREm ($\beta = 1, \alpha = 0.075$), NetREm ($\beta = 1, \alpha = 0.1$), NetREm ($\beta = 1, \alpha = 0.125$), NetREm ($\beta = 1, \alpha = 0.15$), NetREm ($\beta = 1, \alpha =$ from LassoCV).

- Please note we run NetREM using STRINGdb V12 for these 8 previous runs (of $\beta = 1$ and of $\alpha \in \{0.01, 0.025, 0.05, 0.075, 0.1, 0.125, 0.15, \text{LassoCV}\}$.
- We perform additional runs for NetREm using STRINGdb V12: NetREm (β = 0.01, LassoCV), NetREm (β = 0.1, LassoCV), NetREm (β = 10, LassoCV), NetREm (β = 100, LassoCV), NetREm (β = 1000, LassoCV), NetREm (β = 1, α = 0.175), NetREm (β = 1, α = 0.2), NetREm (β = 0.1, α = 0.1), NetREm (β = 10, α = 0.1), NetREm (β = 100, α = 0.1).

Human Pluripotent Stem cells (hPSCs)

Data from Application 5

We run NetREm for these 5 different approaches where we tune hyperparameters $\beta \in$ {0.01, 0.1, 1, 10, 100 and 1,000} and subsequently optimize α using LassoCV. Our input PPIN is the Main NetREm PPIN (§5.1.11) for humans. Thus, these are the 5 NetREm runs for each of the 7,464 TGs: NetREm (β = 0.01, α: LassoCV), NetREm (β = 0. 1, α: LassoCV), NetREm (β = 1, α: LassoCV), NetREm (β = 10, α: LassoCV), NetREm (β = 100, α: LassoCV), (β = 1,000, α: LassoCV).

Budding Yeast cells (YCs)

Data from Application 6

We run NetREm for these 5 different approaches where we tune hyperparameters $\beta \in$ {0.01, 0.1, 1, 10, 100 and 1,000} and subsequently optimize α using LassoCV. Our input PPIN is the STRINGdb V12 PPIN in yeast (§5.3). We have these 5 NetREm runs for each of the 5,355 TGs: NetREm (β = 0.01, α: LassoCV), NetREm (β = 0. 1, α: LassoCV), NetREm (β = 1, α: LassoCV), NetREm (β = 10, α: LassoCV), NetREm (β = 100, α: LassoCV), (β = 1,000, α: LassoCV).

Human Peripheral Blood Mononuclear Cells (PBMCs)

Data from Application 7

We run NetREm (for each of the 13,714 TGs) with $\beta = 1$ and $\alpha = 0.1$ for each of the 9 immune cell sub-types: Naïve CD4 T (Cluster 0), CD14 Monocytes (Cluster 1), Memory CD4 T (Cluster 2), B-cells (Cluster 3), CD8 T (Cluster 4), FCGR3A Monocytes (Cluster 5), Natural Killer (Cluster 6), Dendritic Cell (Cluster 7), Platelet (Cluster 8). Each of these 9 cell-types has 13,714 TGs. So, there are 18 different runs (2 for each cell sub-type based on the input PPIN used). Please note these following 2 different input PPINs that we use for each of the 9 PBMC gene expression datasets:

- We do this for the older STRINGdb V11 human PPIN detailed in §5.1.10.
- We do this for the comprehensive human Main NetREm PPIN (§5.1.11).

NetREm comparisons with RTNduals across 13 different human contexts (related to the Nervous System)

Data from Applications 8, 9, 12

We note that the RTNduals tool (contemporary tool used for benchmarking) may fail to yield any outputs for some contexts. In this analysis, we thus focus on 13 different human contexts where RTNduals (Chagas et al. 2019) returns outputs for TF-TF coordination links (please see §10). 8 of these contexts come from the 8 Control gene expression datasets (Mathys et. al 2019) for neurons/glial cells (also used in AD application #12, but for RTNduals comparison we fix the set of N candidate cell-type TFs for each TG and use all of the 17,926 TGs): Endothelial Blood Brain Barrier (BBB) cells, Oligodendrocytes, Oligodendrocyte Precursor Cells (OPCs), Excitatory Neurons, Inhibitory Neurons, Pericytes, Astrocytes, Microglia. 4 of these contexts come from Application #9 ((Lake et al. 2018) that has control gene expression for 15,693 TGs in 4 cell types: Microglia, Inhibitory Neurons, Excitatory Neurons, Oligodendrocytes). Lastly, we also use the GTEx version 8 Schwann cell (SC) pooled data (Application # 8) that has 14,144 TGs. For all 13 contexts, we run NetREm using the fixed # of candidate TFs for each TG (unless the TG is also a TF, in which case we just remove

the TF from the list of candidate TFs). Hence, these are application runs of NetREm in the absence of prior GRN information.

For this analysis, we also utilize 13 different input PPI networks in humans: **§5.1.1-5.1.9** and **5.1.11-5.1.12.** These are referred to as our 13 NetREm$_{\beta=1}$ models because we fit NetREm using $\beta = 1$ and use LassoCV to subsequently optimize α. Thus, we have 13 different runs of NetREm with an input PPIN across 13 different context → 169 different results.

We also run a baseline version of NetREm (baseREm: NetREm with $\beta = 1e - 100 \approx 0$; α based on LassoCV), which is essentially NetREm with virtually no prior knowledge, for each of the 13 contexts. For this, we use the Main NetREm PPIN for humans. We note any other PPIN could be applied since the prior input PPIN will not be used. This helps motivate the importance of prior knowledge on TF-TF interactions because we show how baseREm performs worse than any of the methods that use input PPINs.

62 Immune cell subtypes based on The Tabula Sapiens Atlas

Data from Application 10

Please note that we run NetREm($\beta = 100, \alpha = 0.1$) using the human Main NetREm PPIN (**§5.1.11**) for 15 cytokine, immune-related TGs (*CCL15, CCL2, CCL5, CSF2, CXCL8, IFNB1, IL10, IL12B, IL18, IL1B, IL23A, IL32, IL6, TNF, TNFSF15*) across the 62 different Immune cell subtypes provided by the Tabula Sapiens Atlas. This is run in the absence of any prior GRN knowledge, so each TG has 125 candidate TFs (unless the TG is a TF, in which case it has 124 candidate TFs), which we obtain from (Berenson et al. 2023).

§8.2 NetREm with prior GRN information for TG-specific TFs

Data from Applications 11-12 as well as multiomics data

Please note that we refer to Applications 11-12, where we run NetREm with prior GRN information for TG-specific TFs. **Table S25** provides metrics on the # of target genes (TGs), # of cells for training NetREm models (M), and metrics on values of N, the # of candidate TF predictors. The value of N may vary across the TGs because of the prior reference gene regulatory network (GRN) of TF-RE-TG links (derived from multi-omics data, which we explain in §7) where RE are Regulatory Elements (REs) linked to the TG by peak-to-TG links.

For our applications in Schwann cells (SCs) and Alzheimer's disease (AD) versus controls, we use $\beta = 100$. We use the comprehensive preprocessed human PPI network (PPIN) (the Main NetREm input PPIN **§5.1.11**) as our network regression constraint.

Application 11:

For myelinating (mSCs) SCs, we use a stricter sparsity hyperparameter of $\alpha = 0.1$. In non-myelinating (nmSCs) SCs, we used a relatively more relaxed hyperparameter of $\alpha = 0.025$. Further, we filter predictors based on goodness of fit, retaining only TGs with test NMSE values less than 1.01 in nmSCs. We also filter TGs based on test MSE values where we impose a harsher filter on nmSCs due to the relaxed sparsity during model training. That is, we retain mSC TGs where test MSE values are less than 1.5 and retain nmSC TGs where test MSE values are less than 1.0005.

Application 12:

For AD applications we fix $\beta = 100$, exclude the y-intercept. For the 8 neuronal/glial cell-types in AD and/or controls, we use Lasso with α = 0.25. For the ease of comparison across 18 TF-TG regulatory networks and respective TF-TF interaction networks, we apply no additional filters.

Section §9: General Overview of Benchmarking NetREm's Outputs

To ensure a robust evaluation of various methods in computational biology and bioinformatics, it is essential to have a large and diverse collection of datasets. These datasets should encompass either complete or partial ground truth information, as this is vital for accurately assessing the performance of different techniques. Having a rich dataset allows researchers to evaluate how well methods generalize to real-world scenarios and edge cases, enhancing the reliability of the findings.

In addition, multiple objective evaluation criteria are crucial for effectively measuring and comparing the performance of these methods. Such metrics might include accuracy, precision, recall, F1 score, and area under the ROC curve, which provide insights into the strengths and weaknesses of each approach. Depending on the specific biological application or research question, additional context-specific metrics may be necessary to capture unique characteristics, such as sensitivity to changes in gene expression or robustness to noise.
Furthermore, the evaluation process often involves running and comparing a substantial number of methods. This complexity is compounded by the need for careful statistical analysis to ensure that comparisons are valid and meaningful. Techniques such as cross-validation, significance testing, and the use of ensemble methods can help consolidate results and provide a clearer picture of method performance.

Motivation for Benchmarking

Benchmarking is a critical process in computational biology and bioinformatics for several reasons. First, it establishes a standardized framework for evaluating different algorithms and methods, allowing researchers to understand how new approaches perform relative to existing ones. This comparison can drive innovation and improvement, as researchers can identify areas where their methods excel or fall short.

Second, benchmarking fosters transparency and reproducibility in research. By publicly sharing datasets, evaluation criteria, and results, researchers enable others in the field to replicate studies and validate findings. This openness enhances the overall quality of research and builds trust in the results produced by various methods. Finally, effective benchmarking can accelerate the translation of research findings into practical applications. By clearly demonstrating the performance of various methods in a controlled environment, researchers can better identify the most promising techniques for further development and potential clinical application.

Testing NetREm's Networks

In this context, there is a particular need to test NetREm's cell-type transcription factor (TF)-target gene (TG) regulatory networks. This involves examining the interactions between TFs and their corresponding TGs to understand how specific regulatory mechanisms operate within different cell types. Additionally, investigating TG-specific cell-type TF-TF coordination links is crucial. These links reveal how transcription factors coordinate with one another to regulate gene expression uniquely in each cell type, providing insights into the underlying regulatory networks.

Moreover, exploring the overall cell-type TF-TF coordination networks is essential for understanding the broader regulatory landscape. By analyzing how transcription factors interact within these networks, researchers can uncover key regulatory hubs and pathways that drive cell-type-specific functions. This comprehensive approach will enhance our understanding of cellular regulation and aid in the development of targeted therapies and interventions in various biological contexts.

§9.1: Cell-type-specific TF-TG regulatory networks (output 1)

Please note that we benchmark our TF-TG regulatory network links using various approaches.

§9.1.1: Benchmark Regression Models (BRMs)

4 baseline regression models (BRMs: ElasticNetCV, LassoCV, Linear Regression, RidgeCV).

- Simulation Study (Figure 2 and beyond): We compare results for NetREm-based models with those of the 4 BRMs. We compare regression metrics such as Mean Square Error (MSE) values. We see the robustness of predicted coefficients to perturbations in the input PPI network and/or underlying gene expression data. We do other adjustments to the main simulation study and compare outputs for NetREm with those for the 4 BRMs. We also highlight how the RTN GRN-inference tool cannot infer TF-TF coordination for the TG and how it does not prioritize lowly-correlated TFs with TG, even if those TFs may be involved in regulating the TG through more complicated avenues.
- We also present comparisons of MSEs across BRMs and NetREm models in mSCs and nmSCs in humans.

§9.1.2: Ground truth gene regulatory networks for validation

We utilize various ground truth GRN datasets to evaluate NetREm's performance against the performance of other GRN inference tools. Moreover, we illustrate how NetREm's outputs may alter based on tuning the hyperparameters: network-regularization $\beta > 0$ and sparsity $\alpha \geq 0$.

- In hESCs, we compare NetREm's performance using a ground truth GRN that is signed (i.e. has coefficient signs c^* for TF predictors): TF-sign-TG links where sign is + (if $c^* > 0$, for activators) and – (if $c^* < 0$, for repressor).
- In HSCs, mESCs, mDCs, hPSCs, and YCs, our ground truth GRN simply has TF-TG regulatory links (with no sign).
- In HSCs, we compare NetREm against the 4 BRMs, GRNBoost2, and RTN GRN-inference tools.
- In mESCs, we compare NetREm against the 4 BRMs and GRNBoost2.
- For mDCs, hPSCs, and YCs, we benchmark NetREm against several state-of-the-art (SOTA) GRN inference tools.
- We have experimental data for 8 core TFs (EGR2, NR2F2, RXRG, SOX10, SREBF1, STAT1, TEAD1, YY1) in SCs. In mSCs and nmSCs, we utilize a ground truth GRN for only 7 core SC TFs (EGR2, NR2F2, SOX10, SREBF1, STAT1, TEAD1, YY1). that we have validation data for in rats/mice: both Loss-of-function (LOF) experiments and direct TF binding information (based on ChIP-seq peaks for these TFs). We also have ChIP-seq data for RXRG. In particular, we focus on the TF-TG links that are uncovered for these 7 TFs where direct TGs are those with support from both LOF and ChIP-seq data and LOF TGs solely have LOF data for support.
- We also have a ground truth cell-type GRN (from the BrainSCOPE resource) for Control Microglia and Control Oligodendrocytes, which we utilize to benchmark NetREm's performance (with multiomics input data) against scNET (which applies the scGRNom tool on multiomics data) for the same gene expression datasets in Application #12.

§9.1.3: Showcasing NetREm's grouped variable selection property

NetREm's grouped variable selection property prioritizes subnetworks of functionally coordinating TFs for co-regulating TGs. We show some examples from human HSCs: fixed N = 177 to 178 candidate TFs for each TG. Our examples show that Novel cell-type TFs may be involved in biologically-relevant enriched PPIs with other substantiated TFs. To illustrate this, we identify TGs with TFs that are verified (in the ground truth) as well as novel TFs that are predicted by NetREm. We compare findings for these TGs across BRMs. And, we determine enrichments of PPIs (using the Metascape(Zhou et al. 2019) tool).

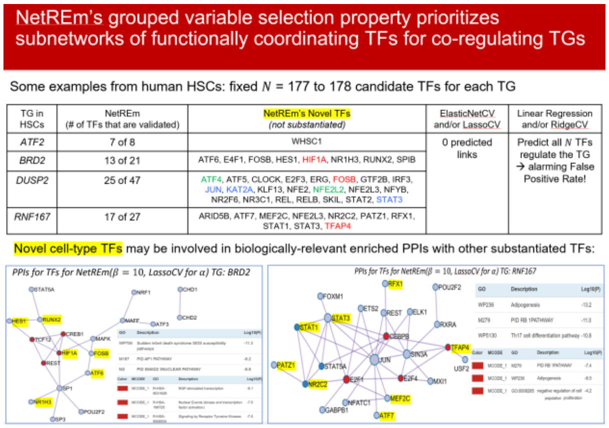

Using the Metascape PPI enrichment tools helps to validate that novel TFs may interact with validated TFs (i.e. substantiated TFs for the TG in the ground truth GRN) in biologically-meaningful PPIs. Further, it illustrates how TFs coordinate with each other in established PPIs to drive various biological processes. And, it presents examples of this grouped selection property of NetREm (and other network-regularized regression models) that use the prior knowledge on interactions among the predictors to drive the regression to prioritize certain outputs.

In the process, we show how ElasticNetCV and LassoCV may struggle to predict results for these TGs and how RidgeCV and Linear Regression may have an alarming potential for False Positives (by predicting all candidate TFs regulate the TG).

Novel TFs may be involved in meaningful PPIs with TFs that have been substantiated. And we note that these ground truth GRNs may be inadequate and partial (so NetREm's novel TFs could be relevant).

§9.1.4: Using Downstream analyses to validate TF-TG links

We do eQTL-based validation of our TGs, especially for situations where we can predict TF-Regulatory Element (RE)-TG links. We see if the SNP is predicted to alter binding of the TF, if the SNP is linked to changes in the TG expression

(based on the eQTL data), if the TF is predicted to regulate the TG. If we have regulatory element information, we can then see if the SNP is in the Regulatory Element and if the TF is predicted to regulate the TG via the regulatory element. We use SNPs related to disease/traits in GWAS and/or to changes in expression via eQTL analysis. These SNPs are mapped onto our TF-TG regulatory networks (comprise TF-RE-TG links) using impacted TFBSs and REs for TGs.

Further, we perform gene enrichment analysis of TGs and relative gene percentile analysis. That is, we do gene set enrichment analysis to see if the predicted TGs of TFs are indeed enriched for biological terms. We also see if TGs of core cell-type TFs have statistically significantly higher expression in the relevant cell-type compared with other cell-types.

§9.1.5: Evaluating scale-free characteristics of predicted TF-TG regulatory networks

We perform comparison of NetREm with scNET to show that NetREm exhibits better scale-free properties and improved evaluation metrics (e.g. signed Jaccard Similarity and signed Area Under Precision Recall Curve) using the brainSCOPE resource as a proxy for a ground truth GRN.

§9.2: TG-specific TF-TF Coordination networks for the cell-type (output 2)

TG-specific evaluation not only validates that these TGs play a role in co-regulating the TG (directly and/or indirectly) but that they share a certain relationship with one another (i.e. act in a coordination fashion, whether antagonistic or cooperative). Further, we evaluate NetREm's TG-specific TF-TF coordination networks in various contexts using a recent study that performed paired yeast one-hybrid experimental assays to uncover TF interactions during direct DNA binding in immune-related contexts. To this end, we run NetREm for 15 TGs across 62 immune cell subtypes and evaluate its predicted cooperative ($B > 0$) and antagonistic ($B < 0$) TF-TF coordination links.

§9.3: Overall cell-type-specific TF-TF coordination networks (output 2)

- We use a contextual Integrated Interactions Database (IID) with annotated TF-TF links (Kotlyar et. al 2022) associated with various diseases/traits/tissues. This Contextual IID helps us determine if we do prioritize meaningful TF-TF links (e.g. context-specific) based on various evaluation criteria.
- We use the CellMarkers 2.0 database to validate the cell-type specificty of various TFs in top TF-TF links for cell-types.
- We compare NetREm models run with $\beta = 1$ to a baseREm model (NetREm run with virtually no prior input PPIN knowledge) across 13 different human contexts for 13 different input PPI networks. This motivates the importance of prior PPIN knowledge. Further, we evaluate NetREm against RTNduals for these 13 contexts for these 13 input PPI networks. That is, for each context, RTNduals outputs its top k TF-TF links and the corresponding number of top k coordination links (high $|\bar{B}|$) are extracted from each of the NetREm(α: LassoCV) models. We analyze the % of links that are valid based on an Evaluation PPIN. We fit a baseline model of NetREm run in the absence of an input PPIN (i.e. baseREm: $\beta \approx 0$); this helps motivate the value of PPIN-based regularization. We also present the results for 13 NetREm(β = 1) models (i.e. NetREm$_{\beta=1}$ models) for different input PPI networks. 10 of 13 NetREm$_{\beta=1}$ models predict TF-TF coordination links that have a statistically significantly higher % of valid links across the 13 contexts when compared with RTNduals. We do not observe that the baseREm model has statistical significance.
- We perform 1-sided t-tests of statistical significance to compare the magnitude of cell-type TF-TF coordination scores $\bar{B}$ among True Positives, False Positives, False Negatives. This shows that NetREm can uncover biological truths and nominate promising candidate PPIs for further investigation.
- We use experimental data such as ChIP-seq peaks and Cut&Run data for TFs in Schwann cells and neural cells to validate potential cooperativity among TFs in the corresponding applications.

§9.4: Robustness of NetREm's aggregate context-specific outputs to changes in the input PPIN networks

We evaluate the performance of NetREm for predicting TF-TF coordination networks and TF-TG regulatory networks across 13 human contexts when we vary these input PPINs. We fix β = 1 for these 13 NetREm-based models (that we call NetREm$_{\beta=1}$). For reference, we run a baseline NetREm model (that we call baseREm), where β = 1e-100, which is so small that it is virtually 0; in this case, baseREm is not incorporating any prior PPIN knowledge.

In this analysis, we evaluate the similarity of NetREm's final output networks (based on these different respective input PPINs) using various statistical measures such as: significant overlap, cosine similarity, spearman correlation (ρ), Jaccard Similarity. These 13 input PPINs exhibit a range of diversity, with average Spearman correlation ρ ranging from 2.45e-5 to 0.85 (average 0.316) and average cosine similarity CS ranging from 0.0887 to 0.8824 (average 0.40). As anticipated, models with more similar input PPINs (higher ρ) tend to produce similar outputs, displaying higher Jaccard Similarity (JS) in predicted signed TF-TG regulatory links and higher ρ in the TF-TF coordination networks. Nonetheless, our results suggest that for a fixed β, NetREm's signed TF-TG regulatory and $\bar{B}$ networks are robust to variations in the PPIN that is input.

Section §10: NetREm analysis for 13 different human contexts (in the Nervous System)

§10.1 Building the Evaluation PPI Network

Please note that we combined PPIs from the following 4 input PPINs: Main NetREm PPIN, STRINGv12 all (direct and/or indirect PPIs), Final Interactome, and Max SAINT Score PPIN. We filtered these PPIs for only the proteins that comprise the 1,431 TFs in the Max SAINT Score PPIN. We also added identified TF-TF interactions by a recent study ((Berenson et al. 2023)) that identified 350 unique PPIs in humans. This resulted in 140,928 total rows, corresponding to 70,464 unique PPI rows.

Then, we annotated the links in this Evaluation PPIN as stronger evidence or weaker evidence based on the strength of the evidence and the source of the PPIN. In the Final Interactome, the authors tend to select PPIs with combined scores (e.g. experimental and database channels) above 0.7 for STRINGdb and above 0.73 for HIPPIE. Similarly, we opt to annotate our Evaluation PPI Network in the following way, with the following categories:

- Stronger evidence links are PPIs found in at least 1 of these following PPINs:
    - Final interactome PPIN
    - Max SAINT Score PPIN
    - STRINGv12 PPIN with combined score > 0.7
    - Main NetREm PPIN with combined score > 0.7
    - List of TF-TF human interactions identified by a recent study (Berenson et al. 2023)
- Weaker evidence links are the remaining PPIs in the Evaluation PPIN and are broken into the following 2 sub-groups. For a given PPI we see if it can be a middle evidence link based on the STRINGv12 PPIN or Main NetREm PPIN. If not, then it will be assigned as a weakest evidence link.
    - Middle evidence links:
        - STRINGv12 PPIN with: 0.5 ≤ combined score ≤ 0.7
        - Main NetREm PPIN with: 0.5 ≤ combined score ≤ 0.7
    - Weakest evidence links:
        - STRINGv12 PPIN with: combined score < 0.5
        - Main NetREm PPIN with: combined score < 0.5

The Evaluation PPIN is not weighted and has no scores. It just has the links annotated as either stronger evidence links or weaker evidence links; for weaker evidence links, there are 2 sub-groups: middle evidence links (remainder: 0.5 ≤ combined score ≤ 0.7) and weakest evidence links (remainder: 0.5 ≤ combined score ≤ 0.7). Ultimately, our Evaluation PPIN has the following breakdown of 70,464 unique rows:

- Stronger evidence PPIs: 41,437
- Weaker evidence PPIs: 29,027 in total
    - Middle evidence PPIs: 1,693
    - Weakest evidence PPIs: 27,334

We use this Evaluation PPIN to evaluate NetREm versus RTNduals.

§10.2 Gathering Input PPI Networks

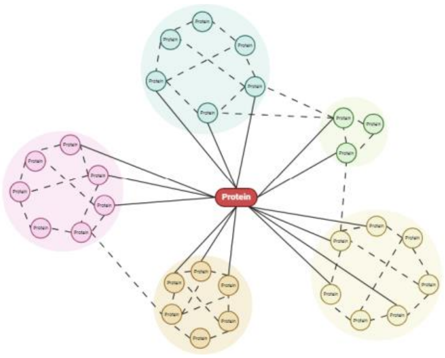

We obtain 13 different Input PPI Networks for humans. These networks are explained in more detail in §5.1.1-§5.1.9, §5.1.11-§5.1.12. We filter these PPINs to only retain proteins that are among the list of 1,431 TFs tested by (Göös et al. 2022), the study that gave us our Max SAINT Score PPIN.

Please note that in the table below, we provide some properties of these filtered PPINs $W^{(0)}$ based on their known PPI links (i.e. weight $w > 0.01$). We evaluate these PPINs based on the Evaluation PPIN that encompasses various sources. For each TG in each context, we will filter these networks further to only focus on the N candidate respective TFs for the TG. Ultimately, NetREm will add minimum default edge weight of 0.01 (i.e. $w = \eta = 0.01$) to missing edges in the input PPINs $W^{(0)}$ to make our networks fully-connected. The results below illustrate stark differences in the input PPINs and their similarities with the Evaluation PPIN. Some PPINs like HIPPIE have a relatively high # of False Positive links. Nonetheless, studies stress the importance of utilizing prior biological network knowledge, as a point of departure, even if that knowledge may be incomplete or contain false links.

Comparing Properties of Known Links for the Human Input TF-TF PPI Networks Based on the Evaluation Network: $W^{(0)}$

**Human Input PPI Network**	**# of TFs (of 1,431 TFs)**	# of Total Known Links for TFs in Filtered PPIN	**Common Known PPI Links with Evaluation Network**					**Known PPI Links (w > 0.01) Not in Evaluation Network**	
			% of Known Evaluation Network Links (out of 70,464 PPIs)	Total Links in Filtered PPIN	# of PPIs that are Strong Evidence Links	# of PPIs that are Middle Evidence Links	# of PPIs that are Weakest Evidence Links	# of False Positives	% of Known PPI Links that are False Positives
Optimal Direction:	↑	↑	↑	↑	↑	↑	↑	↓	↓
Barabasi	742	4,584	5.64%	3,977	3,562	64	351	607	13.24%
Main NetREm Input PPIN	781	64,190	90.94%	64,079	35,049	1,693	27,337	111	0.17%
HIPPIE	1,364	41,611	23.50%	16,558	14,905	166	1,487	25,053	60.21%
HuMAP2	496	1,691	2.24%	1,580	1,238	78	264	111	6.56%
Huri HI-union PPIN	199	239	0.34%	239	239	0	0	0	0.00%
Final Interactome	553	1,739	2.47%	1,739	1,739	0	0	0	0.00%
Max SAINT Score PPIN	1,431	8,031	11.40%	8,031	8,031	0	0	0	0.00%
Multiscale Interactome	732	6,430	8.98%	6,328	5,783	64	481	102	1.59%
STRINGv11 ALL (direct and/or indirect PPIs)	635	4,698	6.16%	4,340	3,164	370	806	358	7.62%

STRINGv11 Physical (direct interactions)	605	3,721	4.87%	3,435	2,670	250	515	286	7.69%
STRINGv12 ALL (direct and/or indirect PPIs)	768	45,497	64.57%	45,497	16,563	1,691	27,243	0	0.00%
STRINGv12 Physical (direct interactions)	766	10,497	14.90%	10,497	7,837	690	1,970	0	0.00%
Contextual (Annotated) Interactions Database	764	15,021	93.23%	14,004	12,291	237	1,476	1,017	6.77%

For instance, the Barabasi PPIN has 742 of the 1,431 TFs. This network has 4,854 known PPI links based on its criteria, which have $w > 0.01$; since the Barabasi PPIN is binary (dummy), these known PPI links are actually given weight $w = 1$ (so sadly, some information is lost regarding edge weight strengths). Of these 4,854 links, 3,977 are also found in the Evaluation PPIN [3,562 are considered strong evidence links, 64 middle evidence links, 351 as weakest evidence links]. Overall, the Barabasi PPIN has prior knowledge on approximately 5.64% (= 3,977/70,464) of the known PPI links in the Evaluation PPIN. The remaining 607 (= 4,584 – 3,977) known links that are not in the Evaluation PPIN are considered as False Positives as of now (since those did not meet the recent stringent criteria to be considered PPIs). Thus, approximately 13.24% (= 607/4,584) known PPI links in the Barabasi PPIN may be termed as False Positives.

Thus, we will have the following buckets for evaluating TF-TF coordination scores:

- Known Protein-Protein Interactions
  - In Both Input PPI Network and the Evaluation PPI Network
  - Novel Discoveries: not in respective input PPI network but are in Evaluation PPIN.
- Poor results:
  - Still unknown (not in input PPI network and not in Evaluate PPIN) → may be True Negatives (TNs) or False Negatives (FNs).
  - Removed (were in input PPI network but are not in Evaluation PPIN) → potential False Positives (FPs)

We note that these 13 different input PPI networks have their own strengths and weaknesses. Some are weighted, others are binary (dummy: no weights). In general, weighted PPI networks are preferable to binary since the weights help with the prioritization of TF-TF links and provide an additional layer of information and wisdom. Nonetheless, there is the Max SAINT Score PPIN, which is a "Goldilocks network" (between weighted and dummy PPINs) that includes weights but mainly for strong links with scores (i.e. weights) between 0.74 and 1. Further, some of these networks are outdated (with some false information), while others are more recent. In addition, some PPI networks focus on only on direct interactions (e.g. STRING physical, Max SAINT Score PPIN), while others focus on direct as well as indirect interactions (e.g. STRING all). There's the contextual IID that we hone for contextual interactions related to the nervous system in the organism; nonetheless, the other PPINs are global and organism-specific and lack context-specificity (e.g. tissue-specificity, cell-type-specificity, condition-specificity). In addition, these 13 PPINs are based on various resources and some input PPINs are subsets of others. Some PPIs in some resources are defined by strong evidence while others are defined based on strong predictions. Furthermore, some PPINs have outdated information (e.g. potentially false positives or missing links) and others are more recent; this is important, especially with the advent of the recent AlphaFold tools by Google's DeepMind company that has been used to predict protein shapes and PPIs at an unprecedented level. All in all, these input PPINs are constructed with their own rigor and own criteria for defining valid PPI links. Thus, these input PPINs are diverse and offer a vast perspective on the state of current knowledge in PPIs.

§10.3 Defining Similarity for the Input PPI Networks

Please note that by default we use 0 for the pairwise similarity of baseREm with any of these 13 NetREm-based models. Here, we define the average Input protein-protein interaction (PPI) network (PPIN) similarity among the different sources across the 13 different contexts where we note that the PPINs may differ based on the specific candidate cell-type TFs used (that are fixed for all TGs in a context).

We explain the metrics and procedures for comparing the similarities among the 13 Input PPI Networks for a fixed context. That is, we compare the similarity among all known ($w > 0.01$) and novel ($w = 0.01$) links.

The analysis aims to compare PPI networks across different sources or experimental conditions, focusing on their similarity in terms of interaction strengths and network structure. To achieve this, several key metrics are calculated for each pair of PPI networks, providing insight into how much they overlap and how closely their interaction profiles align.

1. Input PPI Networks

The PPI networks being compared come from different datasets or experimental platforms (e.g., STRING, Barabasi, HuMAP2). These networks represent interactions between TFs and target proteins, with scores reflecting the confidence or strength of each interaction. For each pair of networks, the following metrics are calculated to quantify their similarity:

2. Metrics Used for Comparison

a. Spearman Correlation

This measures the rank correlation between the interaction scores in the two networks. Spearman correlation evaluates whether the relative ranking of interaction strengths between pairs of proteins is preserved across the two networks. A high Spearman correlation indicates that the networks assign similar relative importance to the same interactions, even if the absolute scores differ.

b. Cosine Similarity

Cosine similarity measures the angle between two vectors of interaction scores, reflecting how similar the overall interaction patterns are between the two networks. A cosine similarity close to 1 indicates that the networks have highly similar interaction profiles, while a value closer to 0 suggests distinct profiles.

c. Pearson Correlation

Pearson correlation measures the linear relationship between the interaction scores of the two networks. A high Pearson correlation indicates that the scores in one network are linearly related to the scores in the other, meaning that increases (or decreases) in one network's interaction strengths are mirrored in the other.

d. Mean Squared Error (MSE)

MSE quantifies the average squared difference between the interaction scores of the two networks. A low MSE suggests that the scores in both networks are numerically similar, whereas a high MSE indicates larger deviations in the interaction strengths across networks.

e. Jaccard Similarity

Jaccard similarity is used to measure the overlap of interactions between the two networks. Here, interactions with scores above a certain threshold (0.5) are considered "present." The Jaccard index is calculated as the ratio of the number of shared interactions to the total number of unique interactions across both networks. A high Jaccard similarity means that the two networks share many of the same interactions, whereas a low value indicates minimal overlap.

3. Assessment of Known PPIs

In addition to comparing the overall interaction profiles, the analysis also focuses on known protein-protein interactions by filtering out interactions below a minimum score threshold (e.g., 0.01). This provides a more focused comparison of high-confidence, biologically relevant interactions, as follows:

- **Known PPI Network 1 Links**: The number of known interactions (i.e., interactions above the minimum score threshold) in the first network.
- **Known PPI Network 2 Links**: The number of known interactions in the second network.
- **Common Known PPI Links**: The number of known interactions shared between the two networks, providing insight into how much biological information is consistently captured by both.

4. Results of the Comparison

The results of the PPI network comparison are intended to provide the following insights:

- **Overall Similarity of Networks**: The calculated metrics (Spearman, Cosine, Pearson, MSE) provide a detailed assessment of how similar the two networks are in terms of interaction strengths. High correlations and low MSE suggest strong agreement between the networks, whereas low correlations and high MSE highlight significant differences.

- **Overlap of Known Interactions**: Jaccard similarity and the counts of common known links help assess the extent to which the networks capture the same interactions. This is crucial for validating whether different PPI datasets are biologically consistent or complementary.
- **Biological Interpretation**: Networks with high overlap and correlation are likely representing similar underlying biological processes, while those with distinct patterns might capture different biological phenomena or be subject to experimental bias.

Conclusion

The pairwise comparison of PPI networks, using metrics such as correlation coefficients, cosine similarity, MSE, and Jaccard index, provides a comprehensive view of how similar the networks are in terms of interaction strengths and known biological links. These metrics enable the identification of consistent interaction patterns, validation of biologically meaningful data, and exploration of differences across network sources or conditions.

§10.4 Datasets used from the following studies/applications for the 13 Human Contexts

We use data from 13 different human contexts for controls, spanning 3 different studies (or applications). Please note that these applications are:

- Application #8: pooled Schwann cells (SCs)
- Application #9: 4 primary cell-types in the Central Nervous System (CNS)
- Application #12: 8 main cell-types in the CNS for the Control Stages only.

Please note that we run NetREm for all these 13 contexts with no prior GRN information. Hence, each TG (for a given context) has the same fixed $N = \mathcal{N}$ candidate cell-type TFs (unless the TG is also one of the TFs, in which case we simply remove the TF from the list so $N = \mathcal{N} - 1$). Below, please note possible metrics for these TF-TG regulatory networks (complementary GRNs) and overall context-specific TF-TF coordination networks $\bar{B}$.

We note that these 13 contexts contain cell-types that are from or similar to Applications 11 (mSCs vs. nmSCs) and 12 (AD versus Control for 8 CNS cell-types). Hence, we can use the respective prior GRN information for all TGs that we derive to help us obtain a list of cell-type TFs: $\mathcal{N}$. To clarify, we simply use the prior GRN information to obtain a fixed list of cell-type TFs that we hold as constant for all TGs in the cell-type (as their N candidate TFs); that is, we do not vary the TFs for individual TGs (unless the TG is also a TF). We do this to reduce noise and hone in on specific cell-type-related TFs for the TGs.

For example, in pooled SCs, we obtain our $\mathcal{N}$ = 88 cell-type TFs based on all of the following 3 criteria being met:

- TF is found in the prior GRNs for any TGs in mSCs and/or nmSCs
- And is also found in the gene expression dataset for pooled SCs
- And is one of the 1,431 TFs based on the Max SAINT Score PPIN

cell-type	information	source	# of TFs (out of 1,431)	# of Target Genes (TGs)	# of TFs that are TGs	# of TF-TG GRN links that are possible 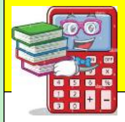 (((# of TFs) x (# of TGs)) - (# of TFs that are TGs))		TF-TF interactions with each other (direct and/or indirect) ((# of TFs) x (# of TFs - 1)) x 0.5 # of Unique TF-TF PPI Links (Known and Novel)	# of Possible Overall TF-TF Coordination Links
Astrocytes	Control	Mathys et. al (Study #12)	93	17,926	93	(93 x 17926) - 93 =	1,667,025	(93 x 92) x 0.5 =	4,278
Oligodendrocytes	Control		82	17,926	82	(82 x 17926) - 82 =	1,469,850	(82 x 81) x 0.5 =	3,321
Inhibitory Neurons	Control		78	17,926	78	(78 x 17926) - 78 =	1,398,150	(78 x 77) x 0.5 =	3,003
Excitatory Neurons	Control		77	17,926	77	(77 x 17926) - 77 =	1,380,225	(77 x 76) x 0.5 =	2,926
Pericytes	Control		98	17,926	98	(98 x 17926) - 98 =	1,756,650	(98 x 97) x 0.5 =	4,753
Endothelial Blood Brain Barrier (BBB) cells	Control		93	17,926	93	(93 x 17926) - 93 =	1,667,025	(93 x 92) x 0.5 =	4,278
Oligodendrocyte Precursor Cells (OPCs)	Control		90	17,926	90	(90 x 17926) - 90 =	1,613,250	(90 x 89) x 0.5 =	4,005
Microglia	Control		97	17,926	97	(97 x 17926) - 97 =	1,738,725	(97 x 96) x 0.5 =	4,656
Schwann_GTEx	GTEx	GTEx (Study # 8)	88	14,144	88	(88 x 14144) - 88 =	1,244,584	(88 x 87) x 0.5 =	3,828
Oligodendrocytes	Lake	Lake et. al (Study # 9)	80	15,693	80	(80 x 15693) - 80 =	1,255,360	(80 x 79) x 0.5 =	3,160
Inhibitory Neurons	Lake		76	15,693	76	(76 x 15693) - 76 =	1,192,592	(76 x 75) x 0.5 =	2,850
Excitatory Neurons	Lake		76	15,693	76	(76 x 15693) - 76 =	1,192,592	(76 x 75) x 0.5 =	2,850
Microglia	Lake		92	15,693	92	(92 x 15693) - 92 =	1,443,664	(92 x 91) x 0.5 =	4,186

Please note additional metrics for these input PPI Networks across the 13 human different contexts in the Nervous System.

# of Unique Known PPI Links														
13 Human Contexts:		HuMAP2	Barabasi PPIN	Context IID	Final Interactome	HIPPIE	HuRI_HI Union	Max SAINT Score PPIN	Multiscale Interactome	Main NetREm PPIN	STRING v11 (all)	STRING v11 (physical)	STRING v12 (all)	STRING v12 (physical)
Astrocytes	Control	27	331	800	125	589	13	244	334	2,366	273	198	2,212	503
Oligodendrocytes	Control	23	307	720	113	476	9	195	283	2,370	253	183	2,215	435
Inhibitory Neurons	Control	18	214	508	90	414	5	161	224	1,608	198	148	1,481	332
Excitatory Neurons	Control	18	223	536	85	402	6	164	236	1,634	178	134	1,500	344
Pericytes	Control	31	372	918	137	515	7	154	346	2,542	282	202	2,362	498
Endothelial Blood Brain Barrier (BBB) cells	Control	24	308	727	120	524	11	202	302	2,161	255	188	1,990	458
Oligodendrocyte Precursor Cells (OPCs)	Control	20	262	649	108	456	9	167	280	1,906	238	171	1,773	413
Microglia	Control	30	352	832	122	532	10	210	328	2,565	271	199	2,391	497
Schwann_GTEx	GTEx	27	341	762	123	505	12	171	323	2,168	250	182	2,023	470
Oligodendrocytes	Lake	18	212	504	89	409	5	160	221	1,560	197	147	1,433	327
Inhibitory Neurons	Lake	18	217	494	83	364	6	141	210	1,520	174	132	1,386	308
Excitatory Neurons	Lake	28	343	825	124	481	7	142	325	2,252	258	187	2,082	460
Microglia	Lake	20	254	602	106	415	9	143	252	1,783	232	169	1,650	374

of Unique PPIs that are Novel (added to the PPIN data source with weight w = 0.01)

13 Human Contexts:		HuMAP2	Barabasi PPIN	Context IID	Final Interactome	HIPPIE	HuRI_HI Union	Max SAINT Score PPIN	Multiscale Interactome	Main NetREm PPIN	STRING v11 (all)	STRING v11 (physical)	STRING v12 (all)	STRING v12 (physical)
Astrocytes	Control	4,251	3,947	3,478	4,153	3,689	4,265	4,034	3,944	1,912	4,005	4,080	2,066	3,775
Oligodendrocytes	Control	4,255	3,971	3,558	4,165	3,802	4,269	4,083	3,995	1,908	4,025	4,095	2,063	3,843
Inhibitory Neurons	Control	2,908	2,712	2,418	2,836	2,512	2,921	2,765	2,702	1,318	2,728	2,778	1,445	2,594
Excitatory Neurons	Control	2,985	2,780	2,467	2,918	2,601	2,997	2,839	2,767	1,369	2,825	2,869	1,503	2,659
Pericytes	Control	4,625	4,284	3,738	4,519	4,141	4,649	4,502	4,310	2,114	4,374	4,454	2,294	4,158
Endothelial Blood Brain Barrier (BBB) cells	Control	3,981	3,697	3,278	3,885	3,481	3,994	3,803	3,703	1,844	3,750	3,817	2,015	3,547
Oligodendrocyte Precursor Cells (OPCs)	Control	3,301	3,059	2,672	3,213	2,865	3,312	3,154	3,041	1,415	3,083	3,150	1,548	2,908
Microglia	Control	4,723	4,401	3,921	4,631	4,221	4,743	4,543	4,425	2,188	4,482	4,554	2,362	4,256
Schwann_GTEx	GTEx	3,801	3,487	3,066	3,705	3,323	3,816	3,657	3,505	1,660	3,578	3,646	1,805	3,358
Oligodendrocytes	Lake	2,832	2,638	2,346	2,761	2,441	2,845	2,690	2,629	1,290	2,653	2,703	1,417	2,523
Inhibitory Neurons	Lake	2,832	2,633	2,356	2,767	2,486	2,844	2,709	2,640	1,330	2,676	2,718	1,464	2,542
Excitatory Neurons	Lake	4,158	3,843	3,361	4,062	3,705	4,179	4,044	3,861	1,934	3,928	3,999	2,104	3,726
Microglia	Lake	3,140	2,906	2,558	3,054	2,745	3,151	3,017	2,908	1,377	2,928	2,991	1,510	2,786

of TFs that have Any Known PPIs (0.01 < w < 1) in the Respective Input PPIN

13 Human Contexts:		HuMAP2	Barabasi PPIN	Context IID	Final Interactome	HIPPIE	HuRI_HI Union	Max SAINT Score PPIN	Multiscale Interactome	Main NetREm PPIN	STRING v11 (all)	STRING v11 (physical)	STRING v12 (all)	STRING v12 (physical)
Astrocytes	Control	33	84	92	61	91	16	77	84	93	79	74	93	91
Oligodendrocytes	Control	29	85	92	60	91	12	73	81	93	74	69	93	89
Inhibitory Neurons	Control	27	64	74	50	76	8	56	65	77	65	59	77	74
Excitatory Neurons	Control	30	71	77	52	77	10	61	73	78	62	57	78	76
Pericytes	Control	36	93	96	65	96	10	70	92	97	78	71	97	94
Endothelial Blood Brain Barrier (BBB) cells	Control	30	77	89	58	90	14	73	78	90	74	68	90	87
Oligodendrocyte Precursor Cells (OPCs)	Control	28	74	80	56	80	11	66	73	82	73	66	82	80
Microglia	Control	33	90	95	62	96	13	78	87	98	79	72	98	94
Schwann_GTEx	GTEx	33	82	87	61	87	15	66	81	88	76	69	88	87
Oligodendrocytes	Lake	27	63	73	48	75	8	55	64	76	64	58	76	73
Inhibitory Neurons	Lake	30	69	74	48	75	10	56	69	76	60	55	76	73
Excitatory Neurons	Lake	35	87	91	60	91	10	66	86	92	73	65	92	88
Microglia	Lake	28	72	78	52	78	11	61	69	80	71	64	80	77

% of Total TFs that have known PPIs in the Respective Input PPI Network														
13 Human Contexts:		HuMAP2	Barabasi PPIN	Context IID	Final Interactome	HIPPIE	HuRI_HI Union	Max SAINT Score PPIN	Multiscale Interactome	Main NetREm PPIN	STRING v11 (all)	STRING v11 (physical)	STRING v12 (all)	STRING v12 (physical)
Astrocytes	Control	35.48	90.32	98.92	65.59	97.85	17.20	82.80	90.32	100.00	84.95	79.57	100.00	97.85
Oligodendrocytes	Control	31.18	91.40	98.92	64.52	97.85	12.90	78.49	87.10	100.00	79.57	74.19	100.00	95.70
Inhibitory Neurons	Control	35.06	83.12	96.10	64.94	98.70	10.39	72.73	84.42	100.00	84.42	76.62	100.00	96.10
Excitatory Neurons	Control	38.46	91.03	98.72	66.67	98.72	12.82	78.21	93.59	100.00	79.49	73.08	100.00	97.44
Pericytes	Control	37.11	95.88	98.97	67.01	98.97	10.31	72.16	94.85	100.00	80.41	73.20	100.00	96.91
Endothelial Blood Brain Barrier (BBB) cells	Control	33.33	85.56	98.89	64.44	100.00	15.56	81.11	86.67	100.00	82.22	75.56	100.00	96.67
Oligodendrocyte Precursor Cells (OPCs)	Control	34.15	90.24	97.56	68.29	97.56	13.41	80.49	89.02	100.00	89.02	80.49	100.00	97.56
Microglia	Control	33.67	91.84	96.94	63.27	97.96	13.27	79.59	88.78	100.00	80.61	73.47	100.00	95.92
Schwann_GTEx	GTEx	37.50	93.18	98.86	69.32	98.86	17.05	75.00	92.05	100.00	86.36	78.41	100.00	98.86
Oligodendrocytes	Lake	35.53	82.89	96.05	63.16	98.68	10.53	72.37	84.21	100.00	84.21	76.32	100.00	96.05
Inhibitory Neurons	Lake	39.47	90.79	97.37	63.16	98.68	13.16	73.68	90.79	100.00	78.95	72.37	100.00	96.05
Excitatory Neurons	Lake	38.04	94.57	98.91	65.22	98.91	10.87	71.74	93.48	100.00	79.35	70.65	100.00	95.65
Microglia	Lake	35.00	90.00	97.50	65.00	97.50	13.75	76.25	86.25	100.00	88.75	80.00	100.00	96.25

% of PPIs in the Fully-connected Input PPIN that are Novel (i.e. not known)														
13 Human Contexts:		HuMAP2	Barabasi PPIN	Context IID	Final Interactome	HIPPIE	HuRI_HI Union	Max SAINT Score PPIN	Multiscale Interactome	Main NetREm PPIN	STRING v11 (all)	STRING v11 (physical)	STRING v12 (all)	STRING v12 (physical)
Astrocytes	Control	99.369	92.263	81.300	97.078	86.232	99.696	94.296	92.193	44.694	93.619	95.372	48.294	88.242
Oligodendrocytes	Control	99.462	92.824	83.170	97.359	88.873	99.790	95.442	93.385	44.600	94.086	95.722	48.223	89.832
Inhibitory Neurons	Control	99.385	92.686	82.638	96.924	85.851	99.829	94.498	92.344	45.044	93.233	94.942	49.385	88.653
Excitatory Neurons	Control	99.401	92.574	82.151	97.169	86.613	99.800	94.539	92.141	45.588	94.073	95.538	50.050	88.545
Pericytes	Control	99.334	92.010	80.284	97.058	88.939	99.850	96.692	92.569	45.404	93.943	95.662	49.270	89.304
Endothelial Blood Brain Barrier (BBB) cells	Control	99.401	92.310	81.848	97.004	86.916	99.725	94.956	92.459	46.042	93.633	95.306	50.312	88.564
Oligodendrocyte Precursor Cells (OPCs)	Control	99.398	92.111	80.458	96.748	86.269	99.729	94.971	91.569	42.608	92.833	94.851	46.612	87.564
Microglia	Control	99.369	92.594	82.495	97.433	88.807	99.790	95.582	93.099	46.034	94.298	95.813	49.695	89.543
Schwann_GTEx	GTEx	99.295	91.092	80.094	96.787	86.808	99.687	95.533	91.562	43.365	93.469	95.246	47.153	87.722
Oligodendrocytes	Lake	99.368	92.561	82.316	96.877	85.649	99.825	94.386	92.246	45.263	93.088	94.842	49.719	88.526
Inhibitory Neurons	Lake	99.368	92.386	82.667	97.088	87.228	99.789	95.053	92.632	46.667	93.895	95.368	51.368	89.193
Excitatory Neurons	Lake	99.331	91.806	80.291	97.038	88.509	99.833	96.608	92.236	46.202	93.837	95.533	50.263	89.011
Microglia	Lake	99.367	91.962	80.949	96.646	86.867	99.715	95.475	92.025	43.576	92.658	94.652	47.785	88.165

# of Known PPIs in the Input PPIN that are Valid (Are Also Found in the Evaluation PPIN)														
13 Human Contexts:		HuMAP2	Barabasi PPIN	Context IID	Final Interactome	HIPPIE	HuRI_HI Union	Max SAINT Score PPIN	Multiscale Interactome	Main NetREm PPIN	STRING v11 (all)	STRING v11 (physical)	STRING v12 (all)	STRING v12 (physical)
Astrocytes	Control	27	307	768	125	589	13	244	331	2,366	266	193	2,212	503
Oligodendrocytes	Control	23	291	680	113	475	9	195	281	2,370	245	177	2,215	435
Inhibitory Neurons	Control	18	204	492	90	413	5	161	223	1,608	190	143	1,481	332
Excitatory Neurons	Control	18	212	517	85	401	6	164	234	1,634	172	130	1,500	344
Pericytes	Control	31	356	876	137	515	7	154	346	2,542	274	197	2,362	498
Endothelial Blood Brain Barrier (BBB) cells	Control	24	294	698	120	523	11	202	300	2,161	248	183	1,990	458
Oligodendrocyte Precursor Cells (OPCs)	Control	20	252	625	108	456	9	167	278	1,906	230	166	1,773	413
Microglia	Control	30	335	798	122	532	10	210	326	2,565	262	192	2,391	497
Schwann_GTEx	GTEx	27	323	747	123	505	12	171	321	2,168	246	178	2,023	470
Oligodendrocytes	Lake	18	202	488	89	408	5	160	220	1,560	189	142	1,433	327
Inhibitory Neurons	Lake	18	206	478	83	363	6	141	208	1,520	168	128	1,386	308
Excitatory Neurons	Lake	28	327	789	124	481	7	142	325	2,252	251	182	2,082	460
Microglia	Lake	20	244	581	106	415	9	143	250	1,783	224	164	1,650	374

§10.5 Parameters used for 13 NetREm Models: NetREm$_{\beta=1}$

We ran NetREm in the absence of any prior Gene Regulatory Network (GRN) information. Thus, for each context, we have a list of $\mathcal{N}$ context-specific TFs (please see **Table S1** for details); for each TG in a given context, we run NetREm using these $\mathcal{N}$ respective TFs as its N candidate TFs. In the cases where the TG is also one of the $\mathcal{N}$ TFs, we remove the TG from that list of TFs for the TG, so $N = \mathcal{N} - 1$.

Our X gene expression data (single-cell gene expression or bulk-level gene expression for the N candidate predictors for the TG) will thus be $N = \mathcal{N}$ (i.e. $X \in \mathbb{R}^{M \times \mathcal{N}}$ for M samples and $N = \mathcal{N}$ candidate predictors) if the TG is not a TF. Otherwise, $X \in \mathbb{R}^{M \times (\mathcal{N}-1)}$ where the respective TG is removed from its list of candidate TFs (since we ensure that a TG does not have its own self as one of its candidate TFs).

We run all 13 different NetREm models with $\beta = 1$ and α subsequently optimized by Cross-Validation (CV, default: 5-fold CV, using the LassoCV function). For each of these NetREm$_{\beta=1}$ models, we use a different input PPI network (PPIN) with weights $0.01 \le w \le 1$, where weights with $w = \eta = 0.01$ are added in as artificial weights with this minimum default edge weight to ensure that our PPIN used for each TG, our W, is fully-connected. We do not fit any y-intercept term.

§10.6 Baseline model used (baseREm)

Please note that we use a baseline NetREm model (a baseREm model), which is essentially applying the NetREm framework (i.e. the steps of the pipeline) in the absence of network regularization. That is, the main method of NetREm is run using $\beta \approx 0$. We note a requirement of NetREm is that $\beta > 0$, so there is some form of PPIN regularization. Nonetheless, by fixing $\beta = 1e - 100$, we are essentially reducing and diluting any potential role of prior network regularization.

Here, we similarly run baseREm using the $X \in \mathbb{R}^{M \times \mathcal{N}}$ gene expression data for predictors for all TGs that are not in the list of $\mathcal{N}$ context-specific TFs. If the TG is a TF as well, we have $X \in \mathbb{R}^{M \times (\mathcal{N}-1)}$ for the remaining $\mathcal{N} - 1$ TGs. We do not fit any y-intercept term.

Thus, baseREm essentially runs NetREm with virtually no (or infinitely small) prior PPIN knowledge; baseREm helps us understand and gauge the role of prior PPIN knowledge for predicting how TFs coordinate to co-regulate TGs. That is, baseREm uses the NetREm pipeline in the absence of prior PPIN regularization.

Hence, we refer to baseREm as: NetREm$_{\beta=0}$, where: NetREm is run with positive $\beta \approx 0$ and α subsequently optimized via LassoCV). We can use any of the input PPI networks or a dummy network. The input PPIN will not matter since NetREm will apply SVD on this E matrix: $E = \frac{X^T X}{M} + \beta A \in \mathbb{R}^{N \times N}$ and for $\beta \approx 0$, then $\beta A \rightarrow 0$, and $E \approx \frac{X^T X}{M} + 0 \approx \frac{X^T X}{M}$. For some contexts, we have required the use of our Truncated SVD approach due to issues with the condition number of our resulting E matrix when running baseREm. In this case, the predictions are made solely based on the gene expression data.

We note that we set up baseREm as NetREm(positive $\beta \approx 0$, α optimized by LassoCV). This is roughly equivalent to us running NetREm with a constant, fully-connected network, where all of the pairwise edges among the given N candidate TFs (for the TG) are a fixed weight (e.g. assigning 0.01 to all pairwise edge weights in the PPIN). In this case, there is virtually no benefit from any PPIN knowledge since all edges are the same weight. An analogy is highlighting every word in a document or making every word **bold**; in doing so, virtually nothing will be gained and there is no benefit in terms of differentiating the importance or value (pun intended) of anything.

Please note that in terms of TF-TG regulatory network prediction, this is akin to using the simple LassoCV benchmark regression model (BRM).

By using baseREm, we can further motivate the role of prior protein-protein interaction (PPI) network (PPIN) data in our network-regularized regression model. We can illustrate how any prior wisdom on PPIs, on any potential interactions among our TF predictors, is helpful even if some of the PPIN knowledge is wrong (False Positive links of unreliable PPIs that have been added) or incomplete (False Negative links that are missing).

§10.7 Comparison with RTNduals

We consider applications where RTNduals returns its top k TF-TF links as it may fail to return links in some contexts. For the sake of comparison, we similarly consider this k (for each context) fixed.

For each context, we then extract the top k TF-TF links from each of the 13 NetREm$_{\beta = 1}$ models (i.e. their context-specific $|\bar{B}|$ networks:) and the baseREm model. We identify which of these top k links are found in the Evaluation PPIN. Those TF-TF links are considered valid. We compare these results across the 13 NetREm$_{\beta = 1}$ models, baseREm model, and RTNduals for the given context. We repeat this for the remaining 12 human contexts.

We perform this comparative analysis between NetREm and RTNduals for these 13 different NetREm models and for baseREm as well. Overall, we find that on average, across the 13 contexts, our 13 NetREm$_{\beta=1}$ models still perform better than RTNduals and baseREm, predicting a higher % of valid links on average (based on the Evaluation PPIN). This highlights the benefit of our methodology that incorporates PPINs, regardless of the input PPIN. That is, network-regularized regression studies emphasize the value of incorporating prior knowledge of interactions among the predictors regardless of how accurate or complete such networks may be. Such prior networks are meaningful points of departure for the model to learn.

§10.8 Pairwise comparisons for the Input PPI networks

Please note that for NetREm(β = 1, α subsequently derived using LassoCV), we use the following input PPI Networks in humans: Barabasi PPIN, Final Interactome, HIPPIE, HuMAP 2.0 PPIN, HuRI_HI-union PPIN, Max SAINT PPIN, Multiscale Interactome, Main NetREm PPIN, STRINGv11 All, STRINGv11 physical, STRINGv12 all, STRINGv12 Physical, Contextual IID PPIN.

For the baseREm case: NetREm($\beta \approx 0$, α subsequently derived using LassoCV), we can use any input PPI network since $\beta < 1e - 100$ is so small that the effects of any PPIN regularization will be negligible at best.

Thus, there are 14 possible PPIN settings (13 involving actual PPINs and 1 that is baseREm): Barabasi PPIN, Final Interactome, HIPPIE, HuMAP 2.0 PPIN, HuRI_HI-union PPIN, Max SAINT PPIN, Multiscale Interactome, Main NetREm PPIN, STRINGv11 All, STRINGv11 physical, STRINGv12 all, STRINGv12 Physical, Contextual IID PPIN, baseREm. Hence, we have (14)(13)/2 = 182/2 = 91 unique pairwise comparisons that are possible.

78 of these pairwise comparisons are among the 13 $\text{NetREm}_{\beta = 1}$ models and the remaining 13 are between a given $\text{NetREm}_{\beta = 1}$ model and baseREm.

For a given context: we note that the 13 $\text{NetREm}_{\beta = 1}$ models and the baseREm model return TG-specific undirected TF-TF coordination networks B for the context, an overall context-specific undirected TF-TF coordination network $\bar{B}$, and an overall context-specific TF-TG regulatory network (our complementary GRN).

We thus endeavor to quantify properties of these different context-specific TF-TF coordination networks and TF-TG regulatory networks. This analysis is done using various statistical measures of similarity, which we explain in the following sub-sections.

The goal is to quantify NetREm's robustness to changes in the input PPI Network (PPIN) that is provided. We achieve this analysis by the 78 pairwise comparisons across the 13 contexts. For our baseline comparison, we use baseREm. This helps provide a range of comparison since baseREm does not use an input PPIN (as the role of the prior PPIN is virtually none). Further, we note that these input PPINs exhibit a vast diversity in their properties and links. Some input PPINs focus on strong, direct physical links only. Others incorporate indirect interactions. Some provide only strong weights (e.g. Max SAINT Score PPIN only has weights above 0.74), some other others include a spectrum of weights between 0.01 and 1 (after pre-processing), and then there are those that are binary (dummy) with no weights. For these binary PPINs we assign a score of 1 to all links described in the network; the missing pairwise links will be assigned a default edge weight of 0.01.

§10.9 Analyzing the Robustness of NetREm's TF-TG Regulatory Networks

Please note the following analysis for each pairwise set of TF-TG regulatory networks. Here, 2 TF-TG regulatory networks are compared to assess their similarity using a combination of network statistics and statistical tests. Below is an outline of the general methods applied in the comparison, suitable for inclusion in a supplemental section:

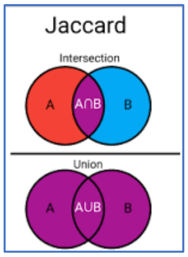

1. Data Setup and Similarity Metrics

The analysis involves comparing multiple networks, each representing regulatory relationships between TFs and TGs. For each pair of networks, several key metrics are calculated:

- **Common Links**: The # of regulatory interactions (links) that are shared between the 2 networks.
- **Total Links in Network 1 and Network 2**: The total number of links in each of the 2 networks being compared. These values represent the overall size and complexity of the networks.
- **Jaccard Similarity (JS)**: A measure of similarity between two sets, calculated as the ratio of common links to the union of links in both networks:

$$Jaccard\ Similarity = \frac{Common\ Links}{(Total\ Links\ in\ Network\ 1) + (Total\ Links\ in\ Network\ 2) - (Common\ Links)}$$

- This metric provides an intuitive measure of how much overlap exists between the two networks.

2. Hypergeometric Test for Statistical Significance

To assess whether the overlap (i.e., the number of common links) between two networks is statistically significant, a **hypergeometric test** is employed. This test determines if the observed number of common links between the networks is greater than what would be expected by random chance.

- **Hypergeometric Distribution**: This distribution models the probability of obtaining a specific number of common links (successes) in a sample drawn without replacement, making it well-suited for evaluating network similarities where each link can only be present once.

- **Parameters of the Test**:
  - K: The total number of possible links, which represents the number of potential links across both networks.
  - z: The total number of links in Network 1.
  - Z: The total number of links in Network 2.
  - q: The number of common links between the two networks.
- **P-value Calculation**: The hypergeometric survival function is used to calculate the p-value, which represents the probability of observing at least as many common links as are found in the actual data, given the total number of possible links. The formula used is:

$$p - \text{value} = P(Q \geq q) = \text{hypergeom.sf}(q - 1, K, z, Z)$$

This p-value indicates whether the overlap is statistically significant or likely to occur by chance.

3. Multiple Testing Correction

Since multiple pairwise comparisons between different TF-TG networks are performed, the p-values are adjusted for multiple testing. We do this using the **Benjamini-Hochberg (BH) correction**, which controls the false discovery rate (FDR). This adjustment reduces the likelihood of false positives, which could arise from performing numerous statistical tests.

4. Final Output and Interpretation

The final analysis yields:

- **P-values**: These indicate the statistical significance of the observed overlap between two networks. A low p-value suggests a significant overlap, meaning the networks share more common links than expected by random chance.
- **Adjusted P-values**: After adjusting for multiple comparisons, these provide a more conservative estimate of statistical significance.
- **Jaccard Similarity**: This metric offers an intuitive measure of the degree of overlap between networks, complementing the statistical significance tests.

By combining the Jaccard Similarity (which quantifies the extent of overlap) with the hypergeometric test (which assesses the significance of that overlap), the method provides a robust framework for comparing TF-TG regulatory networks.

General Workflow Summary

1. **Quantify Overlap**: Count the number of common links and calculate the Jaccard similarity for each pair of networks.
2. **Assess Significance**: Apply the hypergeometric test to determine whether the observed overlap is statistically significant.
3. **Adjust for Multiple Testing**: Use p-value adjustments to account for the number of comparisons made.
4. **Report Metrics**: Output p-values, adjusted p-values, and Jaccard similarities to summarize the degree of network similarity.

This methodology ensures a rigorous assessment of TF-TG network similarity while controlling for false positives due to multiple comparisons.

§10.10 Analyzing the Robustness of NetREm's Context-Specific TF-TF Coordination Networks

To compare the TF-TF coordination networks output by NetREm for different input PPI Networks, we perform quantitative assessments using various statistical measures. For each of the 13 human contexts, we calculate multiple similarity metrics to assess how closely the coordination patterns of 2 TF-TF coordination networks (derived from different input PPIN networks) match.

In short, we use Net1 to represent the TF-TF coordination network of NetREm(β = 1, α: LassoCV) for a given input PPI network (or NetREm($\beta \approx 0$, α: LassoCV) for the baseline model (baseREm) case). And, Net2 represents the TF-TF coordination network of NetREm applied for another input PPI network (or for baseREm)). Here, Net1 and Net2 are representing different input PPI network knowledge provided to NetREm (where baseREm represents essentially no input PPI network knowledge as β is extremely small and virtually negligible positive #, essentially 0).

Please note that these are the following metrics that we use to analyze the robustness and statistical similarity of these undirected, overall, cell-type TF-TF coordination networks $\bar{B}$.

1. **Pearson Correlation (TF Coordination)**
   - **Description**: The Pearson correlation coefficient measures the linear correlation between the coordination scores of the two networks. It ranges from -1 to 1, where:
     - 1 indicates a perfect positive linear relationship.
     - -1 indicates a perfect negative linear relationship.
     - 0 indicates no linear relationship.
   - **Calculation**: $r = \frac{\sum(x_i - \bar{x})(y_i - \bar{y})}{\sqrt{\sum(x_i - \bar{x})^2}\sqrt{\sum(y_i - \bar{y})^2}}$ where x_i and y_i are the coordination scores of Net1 and Net2, respectively, and $\bar{x}$, $\bar{y}$ are their means.
   - **Relevance**: It helps to identify if the variations in one network's coordination scores are mirrored in the other network, indicating overall similarities in how TF pairs are regulated.
2. **Spearman Correlation (TF Coordination)**
   - **Description**: The Spearman correlation coefficient assesses monotonic relationships by ranking the coordination scores before comparison. It also ranges from -1 to 1.
   - **Calculation**:
     - Rank all scores in both networks.
     - Compute Pearson correlation on these ranks.
   - **Relevance**: This metric is less sensitive to outliers and is used to identify if the ranks of the coordination scores are preserved between the two networks, indicating consistent ordering of TF pair interactions.
3. **Mean Absolute Error (MAE) (TF Coordination)**
   - **Description**: MAE measures the average magnitude of the differences between corresponding coordination scores in the two networks.
   - **Calculation**: $MAE = \frac{1}{n}\sum_{i=1}^{n}|x_i - y_i|$
   - where x_i and y_i are coordination scores for a TF pair from Net1 and Net2, respectively.
   - **Relevance**: MAE provides an overall sense of how close the values are between the two networks, with a lower MAE indicating more similarity.
4. **Root Mean Squared Error (RMSE) (TF Coordination)**
   - **Description**: RMSE provides a measure of the standard deviation of residuals or prediction errors.
   - **Calculation**: $RMSE = \sqrt{\frac{1}{n}\sum_{i=1}^{n}(x_i - y_i)^2}$
   - **Relevance**: RMSE penalizes larger differences between coordination scores more heavily than MAE and thus highlights more significant deviations in similarity between the networks.
5. **Cosine Similarity (TF Coordination)**
   - **Description**: Cosine similarity measures the cosine of the angle between two vectors of coordination scores. It ranges from -1 to 1, where:
     - 1 indicates identical direction (high similarity).
     - 0 indicates orthogonal vectors (no similarity).
     - -1 indicates opposite direction.
   - **Calculation**: Cosine Similarity $= \frac{\sum x_i y_i}{\sqrt{\sum x_i^2}\sqrt{\sum y_i^2}}$
   - **Relevance**: A higher cosine similarity indicates that the direction of change in coordination scores between the networks is highly similar, even if their magnitudes differ.
6. **Common Positive and Negative Links (TF Coordination)**
   - **Description**: These metrics count the number of TF pairs that have a coordination score greater than 0 (positive links) or less than 0 (negative links) in both networks.
   - **Calculation**:
     - Count pairs where both networks have scores > 0 for positive overlap.
     - Count pairs where both networks have scores < 0 for negative overlap.
   - **Relevance**: These counts indicate how many TF pairs are consistently positive or negative in both networks, providing insight into their alignment in terms of coordination behavior.
7. **Jaccard Similarity for Positive and Negative Links**
   - **Description**: The Jaccard similarity index measures the proportion of shared positive or negative links between the networks relative to the total number of unique links.
   - **Calculation**:

$$Jaccard_{positive} = \frac{Common\ Positive\ Links}{\text{Total Positive Links: (Net1 + Net2 − Common Positive)}}$$

$$Jaccard_{negative} = \frac{Common\ Negative\ Links}{\text{Total Negative Links: (Net1 + Net2 − Common Negative)}}$$

- **Relevance**: A higher Jaccard similarity indicates a greater overlap in the positive or negative coordination between the networks.

8. **Hypergeometric Test for Positive and Negative Overlap**
   - **Description**: The hypergeometric test is used to calculate the probability of observing a certain overlap (or greater) of positive or negative links by chance, given the total number of links.
   - **Calculation**:
     - For positive overlap:

$P(at\ least\ k) = 1 - \sum_{i=0}^{k=1} \frac{\binom{J}{i}\binom{T-J}{t-i}}{\binom{T}{t}}$ where T is the total number of TF pairs, J is the number of positive links in Net1, t is the number of positive links in Net2, and k is the observed overlap.

     - Please note that a similar test is conducted for negative overlap.
   - **Relevance**: This test provides a p-value indicating whether the overlap of positive or negative links between the networks is statistically significant or could have occurred by chance.
9. **Counts of Positive and Negative Links in Each Network**
   - **Description**: These metrics count the number of positive and negative coordination scores within each network separately.
   - **Calculation**: Count scores > 0 as positive links and scores < 0 as negative links for both networks.
   - **Relevance**: Knowing the distribution of positive and negative links in each network is crucial to understand their individual characteristics and provides context for overlap comparisons.
10. **Total Number of Links (TF Pairs)**

- **Description**: This is the total number of TF pairs considered in the comparison.
- **Relevance**: total count provides context for interpreting the results of overlap metrics and statistical tests.

Conclusion: These similarity metrics collectively provide a detailed and comprehensive understanding of how closely two TF coordination networks align. The correlations (Pearson, Spearman), error measures (MAE, RMSE), and similarity metrics (Cosine Similarity, Jaccard Similarity Index) assess the strength and directionality of coordination, while overlap counts and statistical tests (hypergeometric) determine the significance of shared regulatory patterns between the networks.

Section §11: Benchmarking for NetREm with No Prior GRN Knowledge

In this section, we have evaluated how effectively NetREm predicts meaningful TF-TG and TF-TF coordination networks in real-world cases, even without any prior GRN info (i.e. all TGs have same N candidate TFs). For each TG, $N = \mathcal{N}$, where $\mathcal{N}$ is global set of TFs; TGs that are TFs have $N = \mathcal{N} - 1$ TFs.

We anticipate our inferred TF-TG regulatory and TF-TF coordination networks in our upcoming SC and AD applications (apps) will be improved, given that we incorporate prior GRNs. We use multiomics data to derive initial TF-RE-TG links; these prior GRNs perform initial feature selection, resulting in a small, customized list of N promising and biologically-relevant TFs for a given TG as input to NetREm. N may vary across TGs.

Preliminary notes:

NetREm enhances robustness through PPIN regularization, prioritizing cell-type-specific TF-TF coordination. This is advantageous in high-dimensional $N > M$ data, where overfitting and multicollinearity can lead to spurious r among variables (Campos et al. 2019; Hoefsloot et al. 2008). Single-cell expression data, prone to high sparsity (≈70% of entries ≈ 0), often fails to accurately represent expression distributions (Nguyen et al. 2020). Further, expression data often

contains noise of both direct and indirect TF-TG interactions (Escorcia-Rodríguez et al. 2023). The inherent complexity of gene regulation in eukaryotes (e.g. humans, mice) can also obscure TF-TF correlations, complicating the identification of functionally coordinated TFs (Nie et al. 2011). TFs co-regulating common TGs typically exhibit high r due to similar expression profiles, suggesting potential co-association and cooperativity (Roy et. al 2020; Nie et al. 2011; Wang et al. 2016). This r can be problematic when predicting TF-TG regulatory links using expression data, as many correlated TFs may be causally related to TG expression (Ahsendorf et al. 2017; Ouyang et al. 2009). Even uncorrelated TF_i-TG r can indicate broader coordination among many TFs including TF_i (Zaborowski and Walther 2020). BRMs struggle with highly correlated features, often selecting independent TFs or omitting true co-regulating TFs, compromising the integrity of their TF-TG regulatory network(Roy et. al 2020; Nicodemus and Malley 2009). Our optimization incorporates PPIN structures among TFs to encourage similarity among strongly connected TFs, thereby accounting for noise due to TF coordination (Parab et al. 2022). NetREm discerns and assigns each TF's influences with superior generalizability and consistency, capturing intricate interactions that may be oversimplified by BRMs. NetREm's balanced approach to predictive accuracy and structural interpretability makes it a computationally sound method. This ability to incorporate comprehensive prior data, such as PPINs, into GRN predictions from expression data is a significant advantage of NetREm over traditional methods. This integration not only enhances the accuracy of the GRN but also ensures that essential but less prominent TFs are recognized in the regulatory network. Such an approach is vital for understanding the full spectrum of TF activities in specific cell types, demonstrating the importance of using extensive prior info in GRN predictions, a capability effectively implemented by NetREm (Dibaeinia and Sinha 2020).

§11.1: Cell-type-specific TF-TG regulatory networks (output 1)

To evaluate our networks in terms of predicting regulatory TF-TG links as well as accurate TF roles (based on signed c^*: +/-), we compare NetREm with BRMs for predicting signed-TF-TG links. We use SERGIO (Dibaeinia and Sinha 2020) to input a signed hESC GRN and generate 6 realistic datasets (1,250 TGs, N = 207), varying M and noise %. Overall, NetREm has the highest precision, indicating its superior reliability in correctly identifying true TF-TG links, assigning activator/repressor roles, minimizing FPs (**Fig. S5**).

We benchmark our inferred TF-TG links using single-cell expression data for human HSCs and mice (mESCs, mDCs) (**Figs. S6-7**, **Tables S8-14**) against gold standard GRNs (McCalla et al. 2023; Zhang et al. 2023). Due to its use of biological information (Shojaie and Michailidis 2009), NetREm demonstrates higher sensitivity in identifying relevant biomarkers, though it shows lower specificity compared to Lasso and ElasticNet (Li and Li 2008). By adjusting β and α, we can fine-tune NetREm's behavior; increasing α might decrease sensitivity but enhance specificity. Our findings are consistent across updates in input PPINs, comparing well between mouse STRINGdb versions 11 (V11) and 12 in mESCs.

NetREm features a unique grouped variable selection mechanism that leverages PPIN structures to prioritize tightly-linked TF groups involved in known PPIs. This optimizes the selection of cell-type TFs that co-regulate TGs, enhancing NetREm's ability to identify complex TF-TF relations. We highlight this in HSCs. ElasticNet and Lasso fail to identify critical TF-TG links, for instance, missing regulation of *ATF2*, a key HSC TF (Ju et al. 2023). Conversely, Linear Regression and Ridge predict that *ATF2* is regulated by all N = 177 TFs, showing their alarming potential for FPs. However, NetREm(β = 10) identifies 8 TFs for *ATF2*, with 7 confirmed by gold standards. This pattern extends to other TGs like *BRD2, RNF167, DUSP2*; NetREm identifies groups of both validated and novel TFs involved in biologically relevant PPIs for these TGs and more. For instance, 1 of 10 novel TFs for *RNF167* is TFAP4, which forms PPIs for adipogenesis and cell population proliferation regulation alongside 3 of 17 verified final TFs. NetREm effectively uses prior TF-TF PPIN knowledge to identify genuine TF-TF coordination for TG regulation, navigating through data noise and avoiding false correlations (r). It integrates r and PPIs among TFs, to achieve superior generalizability and consistency in identifying intricate TF-TF relationships that BRMs may struggle with.

Our TF-TG networks complement SOTA cell-type GRN tools. NetREm has comparable F1 Score, sensitivity, specificity, and accuracy with 10 tools benchmarked in mDCs (McCalla et al. 2023). No method outperforms others. However, NetREm uniquely infers B and cell-type $\bar{B}$, capabilities that other tools lack. We summarize our core benchmarking results for NetREm's TF-TG regulatory networks in this figure below:

§11.2: Cell-type-specific TF-TF coordination scores $\bar{B}$ (output 2)

We adopt innovative approaches to benchmark NetREm's $\bar{B}$ for TF-TF pairs for TG regulation. Specifically, we use Welch 1-sided tests (p-adj < 0.05) to analyze its performance with public STRINGdb Version 11 (V11) as the input PPIN, a proxy for outdated knowledge. V12 represents future insights. TF-TF pairs are categorized into 4 sub-groups: retained in

both PPINs (TPs), removed in V12 (FPs), absent in V11 but present in V12 (FNs, future discoveries), and absent in both versions. NetREm's $|\bar{B}|$ values across these sub-groups are analyzed to see if high $|\bar{B}|$ values for novel TF-TF links (not known in V11 but artificially added with $w = 0.01$) indicate valid FNs and if NetREm effectively prioritizes TPs over FPs (results for mESCs, mDCs, PBMCs: **Tables: S15-20; Figures: S9A-D, S10A-B, S11A-C**). For instance, in mESCs, $|\bar{B}|$ is significantly higher for V11 than non-V11 links and for TPs than for FNs, indicating that high $|\bar{B}|$ often reflects known PPIs. $|\bar{B}|$ is also higher for FNs than for FPs, showing NetREm's ability to flag FP links for removal; this may assist efforts to filter out unreliable PPIs in existing databases (Peng et al. 2017). In terms of novel links, $|\bar{B}|$ is higher for FNs than it is for those whose status remains unknown, suggesting that $|\bar{B}|$ can uncover biological truths and effectively nominate promising candidate PPIs for further investigation. Thus, NetREm prioritizes known TPs and flags actual future TF-TF PPIs that are yet unknown.

To validate NetREm's capability to prioritize biologically relevant links, we leverage a contextual Integrated Interactions Database (IID) (Kotlyar et. al 2022) that annotates PPIs with >243 terms but lacks cell-type specificity (**Fig. S12A-B**). By showing that our top links are indeed enriched for context-specific terms (e.g. NS-related in Microglia (Mic) and pooled SCs, immune-related in PBMCs), we suggest a potential extrapolation to cell-type specificity. This highlights NetREm's pioneering potential to discover cell-type TF-TF PPIs, addressing a crucial gap in existing global PPINs.

Further, we benchmark NetREm against the RTNduals (Chagas et al. 2019) tool using 13 different input PPI Networks. 10 of our 13 PPI Networks identify a statistically significantly higher % of verified PPIs in the Evaluation PPIN. 12 of 13 human contexts (**Fig. S13A-B**), our top k links using 10 of 13 input PPINs outperform RTNduals, with a higher % that are verified PPIs in our Evaluation PPIN. This underscores NetREm's efficacy in identifying TPs and leveraging historical PPINs to predict previously unknown PPIs; this is encouraging as only a small number of ~130-650k estimated human PPIs are currently known (Sevimoglu and Arga 2014; Venkatesan et al. 2009; Yu et al. 2020).

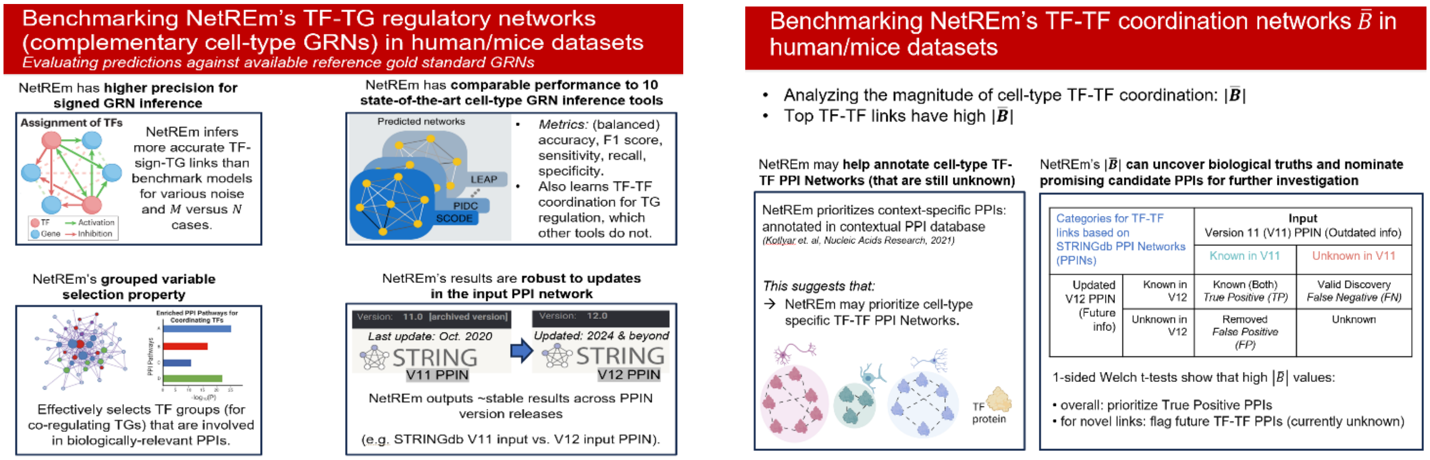

[§11.3: SCENIC applied to human myelinating (mSCs) and non-myelinating (nmSCs) Schwann cells (SCs) in Dorsal Root Ganglion (DRG):]

While state-of-the-art (SOTA) tools like SCENIC (Aibar et al. 2017) may provide indirect insights into TF-TF interactions (e.g. TFs co-regulating many common TGs), they sadly focus on TFs with strong binding and do not incorporate other prior info. To demonstrate, we perform comparative analysis of SCENIC in SCs. After running GRNBoost2 with 1,839 TFs to detect gene modules showing co-expression alongside TFs, we find 16,109 and 14,482 TGs for 1,572 and 1,432 TFs (including core SC TF TEAD1) in nmSCs and mSCs. SCENIC's cis-regulatory motif analysis retains modules with significant enrichment for the appropriate upstream regulator, eliminating indirect TGs lacking motif support. SCENIC detects 640 TFs and 9,241 TGs in nmSCs and 522 TFs and 9,622 TGs in mSCs but fails to identify TEAD1 in both SCs, detecting 3 TEAD family TFs instead. By integrating PPIs, NetREm detects true GRN relations for core TFs with relatively weak binding info (e.g. TEAD1) whose signals may be drowned out. Hence, it is important to integrate additional omics data (Dibaeinia and Sinha 2020), like PPINs as prior info, into GRN prediction from expression regression, which is precisely what NetREm accomplishes.

Section §12: Simulated human embryonic stem cells (hESCs)

In this section, we provide details about the methodology used to obtain the dataset for application §4.1. We randomly select 70% of data for training so M = # of train cells. We randomly select 1,250 TGs and corresponding TFs from the weighted and signed (+: activates; -: represses) ground truth GRN atlas from TF induction analysis on expression and ChIP-seq studies (Sharov et al. 2022). This results in $\mathcal{N}$ = 207 TFs (192 master regulators) and 5,050 signed GRN ground truth links we input to SERGIO (Dibaeinia and Sinha 2020; Dibaeinia 2024) to simulate realistic single-cell data for 1,000 cells (M = 700) and 1,442 genes. We vary noise (30%, 60%, 90%), retrieving 3 different synthetic expression datasets. We repeat this for 100 cells (M = 70).

§12.1: Motivation for using the SERGIO tool

We use the SERGIO tool to evaluate and benchmark NetREm's performance on gold standard data for human embryonic stem cells (hESCs). We want to measure and benchmark the performance of our TF-TG regulatory networks holistically; this includes evaluating the predicted sign (c^*: + or -) because we use them to assign the TF regulatory roles. Here: $c^* > 0$ (+: activator) and $c^* < 0$ (-: repressor) for the TFs. NetREm also uses these to infer the nature of potential coordination among TFs for regulating the TG: ($B < 0$: antagonistic relation) and ($B > 0$: cooperative relation) for the TFs.

Nonetheless, it is difficult to find this signed ground truth gene regulatory network (GRN) to evaluate: c^*-TF-TG regulatory networks. Other real-world applications with gold standards may not have sign information and instead provide TF-TG regulatory links. Or, the gold standards may be measured in different experiments with different assumptions and may not always be reliable for comparing the coefficients signs. Overall, we wanted to evaluate not only our TF-TG regulatory links but also the signs that are inferred. We also wanted to test out different sample sizes M (# of cells) and noise ratios to see our performance. Please note that this is an extension of our underlying simulation studies, but for realistic real-world single-cell data.

We note that this single-cell simulator tool, SERGIO (Dibaeinia and Sinha 2020; Dibaeinia 2024), can help us generate realistic gene expression datasets. In fact, the SERGIO (SERGIO (Single-cell ExpRession of Genes In silicO) tool incorporates TF-TG regulatory interactions (e.g. stochastic nature of transcription, regulation of TFs by many TFs) that are found in the true data. Data simulated by SERGIO is shown to be comparable to experimental data generated by Illumina HiSeq2000, Drop-seq, Illumina 10X chromium, and Smart-seq.

Hence, we use SERGIO to obtain single-cell gene expression data for our human ESC ground truth GRN, and we can then run NetREm and the other benchmark regression models (BRMs: LassoCV, RidgeCV, ElasticNetCV, and Linear Regression) to compare their accuracy, sensitivity, and specificity. SERGIO generates datasets that are statistically comparable to experimental data. Essentially, SERGIO can model the stochastic nature of gene regulation and the role of many TFs in regulating TGs. This tool enables us to reverse engineer by building and inputting a ground truth GRN, and then returning a realistic gene expression dataset based on that. SERGIO has been utilized by other studies to help evaluate and benchmark the performance of various GRNs. In this way, we can ensure that the single-cell gene expression dataset is constructed to align, in a realistic manner, specifically with the ground truth GRN; this enables us to control for other sources of variability and focus only on the relative performance of different tools for GRN inference.

§12.2: Signed ground truth gene regulatory network (GRN) for hESCs:

We obtain an atlas of regulated TGs of TFs in hESCs (Sharov et al. 2022), and analyze results provided in Additional File 5 of the study (Table of all regulated targets of TFs): 94,395 TF-TG regulatory links in hESCs learned after combining 2 data sources. 1: TF binding data (e.g. ChIP-seq data). 2: data on changes in the expression levels of genes (i.e. increase or decrease and by how much) after the given TF is induced so its expression level and presence in the cell is boosted (i.e. how does the TF expression impact expression levels of other genes). To control the False Positive (FP) rate (predicted TF-TG regulatory links that may be false), the study utilizes the Expected Proportion of False Positives (EPFP) to guarantee the FP rate is below a specified threshold. We note that the table provides the direction of TG expression change after TF induction and the corresponding Log10-ratio of TG expression change:

$\log_{10}\left(\frac{TG \text{ expression before TF induction}}{TG \text{ expression after TF induction}}\right)$ where direction $= \begin{cases} down \text{ if } \log_{10}\left(\frac{TG \text{ expression before TF induction}}{TG \text{ expression after TF induction}}\right) < 0 \\ up \text{ if } \log_{10}\left(\frac{TG \text{ expression before TF induction}}{TG \text{ expression after TF induction}}\right) > 0 \end{cases}$. For

simplicity, the table focuses on the dominant TG expression change direction, where it predicts that when direction is down, the TF represses the TG (i.e. $\log_{10}$(ratio) < 0 → TF decreases TG expression → coefficient can be assumed negative) and when direction is up, the TF activates the TG (i.e. $\log_{10}$(ratio) > 0 → TF increases TG expression, coefficient can be assumed positive) (Sharov et al. 2022).

There are other columns of data such as the Direct-EPFP, which is N/A if there is no ChIP-seq data. We want to retain highly-confident TF-TG regulatory links that have ChIP-seq support. Thus, we remove rows where there was no ChIP-seq data, resulting in 57,272 rows of highly-confident TF-TG regulatory links comprising 14,079 genes: 14,044 TGs, 266 TFs (of which, 35 TFs are master regulators and hence are not TGs).

We did not have any underlying gene expression data for steady-state human ESCs, but we could utilize this provided table as a ground truth GRN, where edges are directed from TF and TG and are weighted and signed. Here, the weight is given by the $\log_{10}$(ratio) of TG expression change after TF induction.

Out of the 14,044 TGs, we randomly selected 1,250 unique TGs. Then, we select their corresponding TFs based on the ground truth GRN. Based on our randomly selected TGs, we ultimately select 1,442 genes in total, comprising 207 TFs. Since we subset 1,250 TGs and are only adding their direct TFs, we miss other regulatory TF-TG interactions and hence 192 of those 207 TFs are considered master regulators in our subset GRN (although some of them may be TGs for other TFs that are not in the list of 1,442 genes).

§12.3: Using the SERGIO Tool to Generate hESC gene expression data based on input ground truth GRN:

We utilize the SERGIO (Single-cell ExpRession of Genes In silicO) tool to simulate single-cell gene expression data guided by our underlying ground truth GRN.

A key advantage of SERGIO is that the user can alter and tune different parameters to customize the resulting gene expression data set. We can adjust various parameters for SERGIO, such as the number of genes (we set number_genes = 1442), number of cells (total number of cells per cell-type to be simulated; we set number_sc = 1000), noise type (The type stochastic noise for genes; we set: dpd for Dual Production Decay), sampling state (length of simulations in stationary region; we set: 15), number of cell types (total number of distinct cell types to be simulated; we set number_bins = 1), decays (decay parameter for genes in steady-state simulations; we set: 0.2). For our GRN structure, we input the log10 values for our maximum interaction strength where negative values are for repressive TF-TG interactions and positive values are for activating interactions. We assume that all 192 master regulator TFs have the same basal production rate in the cell-type, so we give a value of 1 for each. We use shared_coop_state = 2, where this value is recommended to be between 1 and 3 and is the value for all hill coefficients in simulations. We retain all other defaults but vary the noise_params to generate 3 different gene expression datasets. That is, we adjust the noise parameter (noise_params) to be 0.3, 0.6, and 0.9, where noise_params controls the noise amplitude parameter q for the genes in our steady-state simulations. SERGIO finds that the accuracy of GRN network inference is impacted greatly by technical noise(Dibaeinia and Sinha 2020), and we want to evaluate NetREm's performance and robustness to these real-world problems. This first setting outputs 3 gene expression datasets for 1,442 genes and 1,000 cells (single-cell samples). Since we will hold out 30% of our gene expression data for testing and use the remaining 70% for training, we will have $M = 700$ cells. In this case, we have $N = 207 < M = 700$.

We also want to explore scenarios where $M < N$, to benchmark NetREm's performance in a scenario where there are more predictors and fewer samples. Hence, we adjust SERGIO so that we also create gene expression datasets for 100 samples (training: 70 cell samples, testing: 30 cell samples). Thus, we retain the previous settings but set number_sc = 100. We still vary the noise parameters (30%, 60%, 90%). Hence, SERGIO returns 6 gene expression datasets for hESCs:

- $M > N$: 1,000 cells: $M = 700$, N = 207 TFs (206 if the TG is also a TF). Noise parameter: 30%
- $M > N$: 1,000 cells: $M = 700$, N = 207 TFs (206 if the TG is also a TF). Noise parameter: 60%
- $M > N$: 1,000 cells: $M = 700$, N = 207 TFs (206 if the TG is also a TF). Noise parameter: 90%
- $M < N$: 100 cells: $M = 70$, N = 207 TFs (206 if the TG is also a TF). Noise parameter: 30%
- $M < N$: 100 cells: $M = 70$, N = 207 TFs (206 if the TG is also a TF). Noise parameter: 60%

- $M < N$: 100 cells: $M = 70$, N = 207 TFs (206 if the TG is also a TF). Noise parameter: 90%

Theoretically, 258,735 TF-TG regulatory links are possible and only 5,050 of these links will be true. Thus, we adjust the noise parameter (we set `noise_params` = 0.3, 0.6, 0.9) and the # of cells to have these 6 datasets. We will be able to also evaluate the coefficients c^* for the TF predictions (where $c^* > 0$ is for activators and $c^* < 0$ is for repressors) since our input GRN is weighted and signed (and is used to generate the respective gene expression data).

§12.4: Corresponding human input protein-protein interaction (PPI) network (PPIN):

To estimate the # of true negatives (TN) using the # of transcription factors (TFs) and target genes (TGs), we make some assumptions about the interactions within this ground truth GRN. The total possible interactions can be calculated if we assume that every TF can potentially regulate every TG. However, the presence of overlaps between the TFs and TGs introduces a slight complexity, which we will address. So, out of 1,250 TGs and N = 207 TFs (of which 15 are also TGs), we find there are 258,735 total possible TF-TG links (where TF ≠ TG). We benchmark NetREm's performance for constructing TF-TG regulatory links for the 1,250 TGs in hESCs across each of the 6 SERGIO-simulated datasets. We input the comprehensive human PPI network of 21,321 edges comprising 10,777 known links and 10,544 artificial links (default edge weight of 0.01) for these 207 TFs. For each of the 15 TFs that are also TGs, we remove the respective TF and its associated edges from the input PPI network and node that $N = 206$ for those TFs; otherwise, $N = N^*$ = 207 for the remaining 1,235 TGs, since we do not incorporate other GRN knowledge to select candidate TFs.

#	1,250 Randomly Selected Target Genes in Human Embryonic Stem Cells (hESCs)										207 corresponding TFs for those TGs (15 in common)	
1	AACS	BTN2A1	CRYBG1	FCAR	IQGAP2	MAP1B	OAZ2	RASA4CP	SLC4A8	TRHDE-AS1	ASCL1	OTX2
2	AAMP	C11orf95	CRYBG3	FGD4	IRX2	MAP1LC3A	OGG1	RASD1	SLC6A12	TRIB3	ATF2	PATZ1
3	ABCA2	C12orf29	CSF1R	FGFR2	ISCA2P1	MAP1LC3B	OIP5	RASGEF1C	SLC6A9	TRIM17	ATF3	PAX5
4	ABCC1	C12orf49	CSK	FITM1	ISCU	MAP2K3	OLA1	RASL10B	SLC7A1	TRIM2	ATF4	PAX6
5	ABCC9	C12orf57	CSKMT	FLNB-AS1	ISG20	MAP2K4	OPA1	RASL12	SLC8A1-AS1	TRIM65	ATOH1	PAX8
6	ABCG1	C14orf93	CSPP1	FLT1	ISPD	MAP3K6	ORAOV1	RASSF1	SLC9A7P1	TRIM9	BACH1	PDX1
7	ABHD4	C15orf40	CTBP2P1	FLVCR2	ITCH	MAP4K4	ORC2	RBM19	SLCO3A1	TRIP13	BHLHE40	PHF8
8	ABRACL	C15orf59	CTGF	FOXD2	ITGB8	MARCH6_TG	OSBPL1A	RBM24	SLCO4A1	TRIQK	BMI1	PITX3
9	ABTB1	C18orf21	CTNNBIP1	FOXH1	ITPK1	MAVS	OSBPL9	RBM42	SLX1A	TRMT10C	CBX3	POLR3A
10	ACADS	C1GALT1C1	CTSH	FPGS	JAK3	MAZ	OSGIN2	RBMS2P1	SMAD6	TRNP1	CBX8	POU3F2
11	ACBD5	C1orf210	CTSL	FREM2	JHY	MBD5	OSMR	RBP1	SMARCB1	TRPA1	CDK9	PPARG
12	ACSL1	C1orf228	CTSV	FRMD6	JMJD1C-AS1	MBTPS1	OSTM1	RCCD1	SMC6	TRPM6	CDX2	PRDM1
13	ACTL6A	C1orf52	CUTA	FRMD6-AS1	JPT1	MCCC1	OTP	RECQL	SNHG18	TSFM	CEBPA	RAD21
14	ACTR1A	C1QTNF1	CXCL5	FSTL4	JPT2	MCCC2	P3H2	RELT	SNHG21	TSKU	CEBPB	RARA
15	ACTR2	C1QTNF12	CYP1B1-AS1	FUBP3	JUN	MCHR1	PAGR1	REPIN1	SNHG9	TSPEAR-AS2	CEBPD	REST
16	ACY3	C2	DAG1	FUCA1	KANK2	MCM3AP	PAK6	RETSAT	SNORA18	TTC39A	CHD1	RFX2
17	ADAMTS2	C21orf91	DAGLB	FUCA2	KANSL1L	MCRIP1	PALB2	REXO2	SNORD139	TTI1	CREB1	RFX5
18	ADAMTS7	C22orf42	DAND5	FUT10	KAT5	MCUR1	PALM2-AKAP2	RFC4	SNRPF	TTLL11	CRY1	RNF2
19	ADAMTS9	C22orf46	DANT1	FUT4	KCNB1	MDM2	PAOX	RFX4	SNTB2	TTN-AS1	CTCF	RUNX1
20	ADCK5	C2orf68	DCAF10	FXYD1	KCNC1	MED13L	PAPOLA	RGL2	SNX10	TUBA4A	CTCFL	RUNX3
21	ADCY5	C4orf46	DCAF8	FXYD3	KCNF1	MED22	PAPSS2	RHBDD3	SNX2	TUBBP6	CTNNB1	RXRA
22	ADCYAP1R1	C5orf30	DCDC2	FZD2	KCNG3	MEF2A	PARP2	RHBDL3	SOBP	TXNDC16	CUX1	SALL1
23	ADM	C6orf132	DCTPP1	FZD6	KCNH3	MEIS3P1	PATL1	RHOB	SOWAHD	TXNDC17	DMRT1	SALL4
24	ADPRM	C6orf141	DDI2	GAA	KCNN2	MEMO1	PAX5	RHOC	SOX1	TYSND1	DUX4	SAP30
25	ADRA2A	C7orf50	DDR1	GAB1	KCTD15	MEMO1P1	PAXIP1-AS1	RHOH	SOX21	TYW3	E2F1	SETDB1
26	AGFG2	C8orf37	DDX11	GABRE	KDELR3	MESP1	PBX1	RIC8A	SOX4	UBA2	E2F4	SIN3A
27	AGPAT5	C9orf135	DDX18	GABRR3	KDM4C	METTL1	PBX3	RIMBP2	SP110	UBAP1	E2F6	SIRT6
28	AGTRAP	C9orf72	DDX56	GADD45B	KDR	METTL2B	PBXIP1	RIPPLY3	SP140	UBAP2L	E2F7	SIX1
29	AHCY	CACNA1H	DEF8	GALNT11	KEAP1	METTL8	PCDH1	RLN2	SP6	UBE2B	EGR1	SLBP
30	AK2	CADPS	DEGS2	GAPVD1	KHDC1	MEX3D	PCDH11Y	RMND1	SPAST	UBE2J2	EGR2	SMAD1
31	AKAP5	CALCA	DENND1B	GAREM2	KHDRBS3	MFAP2	PCMT1	RNASEH1	SPATA2	UBE2Q1	EHF	SMAD2
32	AKAP6	CALCB	DEPDC4	GAS8	KIAA1211	MFN2	PCOLCE	RND3	SPATA6L	UBL4A	ELF1	SMAD3
33	ALDH1B1	CALN1	DERA	GCNA	KIAA1211L	MGLL	PCSK4	RNF121	SPEG	UBOX5	ELF5	SMAD4
34	ALDH1L2	CAMTA1	DFFB	GEMIN8P4	KIAA1522	MIA2	PCSK5	RNF152	SPINK2	UBXN2A	ELK1	SMARCA4
35	ALDH3A2	CAP1	DGAT1	GIPR	KIAA1549	MIA3	PCYOX1L	RNF19A	SPOCD1	UCN2	ELL2	SMC3
36	ALG13	CAP2	DGKH	GLYATL1	KIAA1841	MIATNB	PDE3A	RNF215	SPOCK2	UCP1	EOMES	SNAI2
37	ALG5	CAPN10	DHPS	GM2A	KIF11	MIGA2	PDE7A	RNFT2	SPRR1B	UGT3A1	ERG	SNAPC4
38	ALPK3	CAPN2	DHRS7B	GNA13	KIF21A	MIR302C	PDK3	ROPN1	SPRYD3	ULK2	ESR1	SOX10
39	AMER1	CAPZA2	DKC1	GNAL	KIF5A	MIR4664	DC2P-NPIPB	RPL21P28	SQOR	UNC45B	ESRRA	SOX11
40	AMIGO2	CARD10	DLEU1	GNB2	KLF10	MIR621	PEAK1	RPUSD1	SRM	UNC93B1	ETS1	SOX15
41	AMOTL2	CARD8	DLG5-AS1	GNG8	KLF2	MIR663AHG	PEPD	RRBP1	SRSF9P1	UNK	ETV1	SOX17
42	ANGPT4	CARS-AS1	DLGAP1-AS1	GNL1	KLF7	MIR7-3HG	PFDN5	RRN3	SRXN1	UPK1B	ETV4	SOX2
43	ANGPTL4	CASKIN1	DLX4	GON7	KLHL5	MISP	PGA3	RRP7A	SS18L1	UQCC3	ETV6	SOX5
44	ANKHD1-EIF4EBP3	CASQ1	DM1-AS	GOSR2	KLK14	MKL1	PGAM2	RRP9	SSFA2	UQCR10	FEV	SOX6
45	ANKRD1	CASQ2	DNAAF1	GOT2	KLK4	MKRN1	PGAP2	RSL24D1	SSTR2	USP12	FLI1	SPI1
46	ANKRD12	CASS4	DNAH17	GP1BB	KLK8	MMAB	PGF	RTEL1	STAG1	USP16	FOS	SPIB
47	ANKRD17	CBFA2T2	DNAI1	GPC1	KMT2D	MMP12	PGLS	RUBCNL	STBD1	USP24	FOSL1	SREBF2
48	ANKRD22	CBX7	DNAJB5	GPD1L	KPTN	MMS19	PGPEP1	RUNDC3A-AS1	STC2	USP32P1	FOSL2	STAG1
49	ANKRD23	CCDC106	DNAJB8	GPR107	KRT17P7	MND1	PHF1	S100A3	STK36	USP33	FOXA1	STAT3
50	ANKRD28	CCDC114	DNAJC19P5	GPR155	KRT86	MNDA	PHF14	SALL1	STK38	USP42	FOXA2	STAT5A
51	ANKRD9	CCDC138	DNAL4	GPR78	KRT8P48	MNX1	PHF2	SAR1A	STOX1	USP43	FOXC1	SUZ12
52	ANP32A	CCDC184	DNASE1	GPRIN3	KRTAP4-7	MOB1B	PHF23	SATB1	STOX2	USP8	FOXD3	T
53	ANXA9	CCDC28B	DNHD1	GPSM2	L3MBTL4	MOB2	PHLPP2	SAXO2	STRA6	USPL1	FOXH1	TAF7
54	AP2B1	CCDC6	DNTTIP1	GPX1	LAMC1	MORC3	OSPHO2-KLH	SBDSP1	STRN	UXS1	FOXM1	TAL1
55	AP3M2	CCDC84	DOCK7	GPX3	LAMTOR1	MPND	PI4KA	SBK3	STRN4	VEGFC	FOXO3	TBX3
56	AP4B1	CCDC91	DOK1	GRIK3	LARGE1	MRPL30	PI4KAP1	SCAMP1	STUM	VKORC1L1	FOXP1	TBX5
57	APLP1	CCM2	DPAGT1	GRK3	LARP4	MRPL35	PIP5K1A	SCHIP1	SUMO3	VMAC	GABPA	TCF12
58	APOA1	CCZ1	DPF1	GSN	LBX2-AS1	MRPL51	PJA2	SCLT1	SURF4	VMO1	GATA1	TCF4
59	APOBEC3F	CD2AP	DPPA2P2	GSTA4	LDB3	MRPS2	KD1P6-NPIPP	SCMH1	SYCE1L	VN1R51P	GATA2	TCF7L2
60	APOC1	CD63	DPYD	GSTT2B	LDHAP3	MRTO4	PKMYT1	SCML4	SYCE2	VPS13B	GATA3	TEAD4
61	APP	CD7	DRD4	GTPBP3	LENG1	MSH2	PKNOX1	SCN3A	SYMPK	VPS37D	GATA6	TFAP2A
62	APPL1	CDC25B	DSN1	GUCY2C	LENG8	MSR1	PKP3	SCN7A	SYN3	VPS54	GLIS2	TFAP2B
63	AQP7P3	CDC27P1	DTX1	HAL	LEUTX	MTG1	PLA2G15	SCN8A	SYNE1	VPS9D1-AS1	GRHL2	TFAP2C
64	ARF4	CDC37	DUSP28	HAT1	LHX3	MTIF3	PLA2G6	SCNN1D	SYT4	WASHC3	GTF2B	TFAP4
65	ARF5	CDC42EP5	DYNC1H1	HCFC2	LIN52	MTMR3	PLCD3	SCP2	TAB2	WDFY3-AS2	GTF2F1	TFE3
66	ARFGAP3	CDC42P3	E2F2	HCN3	LINC00239	MTMR4	PLCH2	SDCBPP3	TADA2B	WDR73	HDAC6	THAP1
67	ARG2	CDC7	EBF3	HCST	LINC00479	MTND6P4	PLEC	SEC24C	TAPT1	WEE1	HES1	THAP11
68	ARGLU1	CDH24	ECHDC1	HDGF	LINC00632	MUT	PLEKHA3	SEC31A	TATDN3	WIPI1	HEY1	TP53
69	ARHGAP19-SLIT1	CDK5RAP2	ECHDC3	HEATR1	LINC00891	MVB12B	PLEKHB2	SELENOI	TBCC	WNK4	HHEX	TP73
70	ARHGAP24	CDK5RAP3	ECSIT	HECTD4	LINC00960	MVK	PLIN3	SELENOM	TBCCD1	WNT2B	HNF1A	TSHZ1
71	ARHGAP39	CDKN2C	EDIL3	HEXA	LINC00973	MXI1	PLK2	SEMA4G	TBRG1	WNT6	HNF4A	UBTF
72	ARHGEF26	CDKN3	EFCAB1	HEY1	LINC01089	MYH14	PLOD1	SEMA6A	TBX21	WRNIP1	HOXA7	USF1
73	ARHGEF4	CDO1	EFCAB13	HHIP	LINC01091	MYH3	PLSCR1	SENP8	TCAF2	XKR8	HOXA9	WRNIP1
74	ARID1B	CDRT4	EFCAB14	HINT1	LINC01108	MYHAS	PLXNA3	SEPT2_TG	TCEA1	XPC	HSF1	WT1
75	ARID2	CDT1	EGFL6	HIPK3	LINC01124	MYL10	PMS1	SEPT5-GP1BB	TCEAL1	XPO7	IKZF1	YY1
76	ARID3A	CEMP1	EIF2A	HIST1H2AI	LINC01234	MYL12A	PNOC	SERP2	TCF12	XPOTP1	IRF1	ZBED4
77	ARL9	CENPB	EIF2B1	HIST1H2BJ	LINC01260	MYO1E	PNPLA8	SERPINB7	TCF4	XRCC2	IRF2	ZBTB7A
78	ARMC9	CENPS	EIF2B2	HIST2H2AA4	LINC01410	MYRIP	PODN	SERPINF2	TCIM	YBX1	IRF3	ZKSCAN1
79	ARRDC4	CEP104	EIF5A2	HLCS	LINC01569	MZT1	POLD2	SFMBT1	TDRKH-AS1	YIPF2	IRF4	ZNF263
80	ARV1	CEP162	ELOVL2	HLX	LINC01664	NAALAD2	POLR1C	SFXN4	TEAD4	YPEL1	ISL1	ZNF274
81	ARVCF	CEP170	ELP5	HM13	LINC01686	NABP2	POLR2G	SGSM2	TEX9	ZBTB14	JARID2	ZNF281
82	ASAP3	CERS2	EMC2	HMGA1	LINC01881	NADSYN1	POLRMT	SGTB	TFCP2L1	ZBTB32	JUN	ZSCAN4
83	ASB5	CES4A	EMILIN1	HMOX1	LINC02200	NANOGP4	POM121L1P	SH3BGRL3	TFDP2	ZBTB43	JUNB	
84	ASB6	CFAP69	ENTPD2	HOMER1	LINC02418	NAPRT	POPDC3	SH3BP1	TFPI	ZCWPW2	JUND	
85	ASB7	CGB7	EPHA3	HOXC13	LINC02482	NARF	POT1	SH3D19	TGM1	ZDHHC16	KLF1	
86	ASF1A	CHAC1	EPM2A	HPF1	LIPH	NCCRP1	PP7080	SH3GL2	THNSL2	ZDHHC2	KLF12	
87	ASMTL	CHAMP1	EPPK1	HPS1	LIPT1	NCK1	PPIF	SH3RF3	TIAM1	ZFAND2A	KLF14	
88	ATAD2B	CHCHD10	EPS8L1	HRC	LNX1	NCKAP5L	PPL	SHC4	TK1	ZFAND2B	KLF15	
89	ATG4D	CHCHD5	ERLEC1	HS3ST2	LNX2	NCLN	PPM1M	SHH	TLCD1	ZFAT	KLF4	
90	ATL3	CHD5	ERP29	HS3ST3B1	LOC100129083	NCMAP	PPP1R12B	SHISA6	TLR6	ZFPM1	KLF5	
91	ATP10A	CHDH	ERP44	HS3ST5	LOC100506082	NDC1	PPP1R14A	SHQ1	TM9SF3	ZFYVE26	KLF6	
92	ATP11C	CHGA	ERRFI1	HS6ST1	LOC100506083	NDP	PPP1R16A	SHROOM4	TMEM106B	ZKSCAN5	KLF8	
93	ATP1B2	CHGB	ETFA	HSBP1	LOC100506178	NEAT1	PPP1R17	SIGMAR1	TMEM132C	ZMYM2	KLF9	
94	ATP6V0A2	CHKB	EVA1B	HSPB1	LOC100507351	NEK11	PPP1R3E	SIK1	TMEM147-AS1	ZMYND19	L3MBTL2	
95	ATP6V0B	CHPT1	EXOC6B	HSPB1P1	LOC100652758	NEURL1B	PPP2R3B	SIL1	TMEM161B-AS1	ZNF213	LHX2	
96	ATP6V1D	CHSY3	EXOSC3	HSPB2	LOC101926943	NF2	PPP2R5B	SLA	TMEM184A	ZNF233	MAFK	
97	ATP8B3	CIDEA	FA2H	HTATIP2	LOC101927919	NFIB	PRAC1	SLC12A2	TMEM185A	ZNF277	MAX	
98	AUNIP	CIRBP-AS1	FABP5	HTATSF1P2	LOC389895	NFIX	PRF1	SLC13A3	TMEM198	ZNF280B	MBD3	
99	AVPI1	CIT	FABP5P7	HTR1B	LOC643072	NINJ1	PRIMA1	SLC13A4	TMEM201	ZNF285	MEF2A	
100	AXIN2	CKS2	FADS2	IAH1	LOC647859	NINL	PRKAG1	SLC16A11	TMEM203	ZNF286A	MEF2C	
101	AXL	CLCN5	FAM109B	IARS2	LOC730101	NIP7	PRKCH	SLC16A12	TMEM206	ZNF318	MEIS1	
102	AZGP1	CLDN5	FAM111A	IER2	LONRF3	NKAIN1	PRKCQ-AS1	SLC16A7	TMEM254	ZNF321P	MSC	
103	B3GALT1	CLIC1	FAM114A1	IFIH1	LPCAT1	NLK	PROCA1	SLC1A3	TMEM51-AS1	ZNF333	MXI1	
104	B3GALT4	CLINT1	FAM131A	IFITM10	LRRC37A17P	NLRC5	PRR15	SLC22A18	TMEM63B	ZNF362	MYBL2	
105	B4GALNT1	CLSTN1	FAM149A	IFRD2	LRRC3B	NOM1	PRR3	SLC24A1	TMSB15A	ZNF398	MYC	
106	B4GALNT4	CMC1	FAM151A	IFT172	LRRC6	NPAS2	PRR36	SLC25A25-AS1	TMSB4Y	ZNF414	MYF5	
107	B4GALT7	CMKLR1	FAM151B	IGF2R	LRRC8C	NPHP3	PRR7	SLC25A29	TNFRSF11A	ZNF425	MYOD1	
108	BABAM2	CNDP2	FAM155B	IGFBP3	LSR	NPRL3	PRSS36	SLC25A34	TNFRSF11B	ZNF445	NANOG	
109	BAG5	COL3A1	FAM189A2	IGSF21	LTA4H	NPTXR	PSMF1	SLC25A39	TNFRSF12A	ZNF528-AS1	NELFE	
110	BAGE4	COL5A2	FAM199X	IGSF3	LTBP1	NRAV	PSMG4	SLC25A45	TNFRSF1B	ZNF532	NEUROD1	
111	BARX2	COLEC12	FAM19A2	IL10RA	LTBP4	NRCAM	PTGES	SLC26A11	TNFRSF8	ZNF552	NEUROG2	
112	BBS12	COMP	FAM210B	IL18	LTBR	NREP	PTGIS	SLC2A12	TNFSF12	ZNF568	NEUROG3	
113	BCL11B	COMT	FAM221A	IL1A	LY6E	NRIR	PTPRH	SLC2A13	TNPO1	ZNF595	NFATC1	
114	BCL6	COPB2	FAM241A	IL1RN	LYPD5	NRK	PTPRVP	SLC2A14	TOP2A	ZNF614	NFE2L1	
115	BCRP3	COQ2	FAM46B	IL6R	LYPD6B	NSUN7	PUS7	SLC2A3	TOP2B	ZNF620	NFIA	
116	BEND5	COX14	FAM53B	IMPA2	LYPLAL1	NT5DC3	PYCR2	SLC2A4	TOX	ZNF667-AS1	NFIB	
117	BICRA	COX17	FAM83H	IMPDH1P10	LYRM7	NTF3	PYCR3	SLC2A5	TP73	ZNF672	NFIC	
118	BIRC6	COX5B	FAM86EP	ING2	LZTFL1	NTHL1	PYGM	SLC2A8	TPBG	ZNF763	NFIL3	
119	BLCAP	CPT1A	FAM86HP	ING4	MAB21L1	NTNG1	PYROXD2	SLC30A3	TPCN2	ZNF784	NKX2-1	
120	BLMH	CPT2	FANCI	INMT-MINDY4	MAB21L3	NTNG2	RAB11FIP4	SLC35G1	TPGS2	ZNF805	NKX2-5	
121	BMP4	CREB1	FASTKD1	INPP4B	MAGI3	NUB1	RAB14	SLC38A3	TPRKB	ZNF816-ZNF321P	NR2F1	
122	BRAF	CREG1	FBP1	INTS6L	MAL	NUCKS1	RAB21	SLC39A9	TRAF3IP2-AS1	ZNHIT6	NR2F2	
123	BROX	CRISP3	FBXL13	IPMK	MAML1	NUMA1	RAB30-AS1	SLC45A3	TRAIP	ZPLD1	NR3C1	
124	BTBD1	CRLF1	FBXO33	IPP	MANEA	NXN	RAD9B	SLC4A11	TRAM2	ZSCAN20	NRF1	
125	BTBD8	CRLF3	FBXO7	IQCH	MANEAL	OAS2	RAMP2	SLC4A7	TRAPPC2L	ZSCAN5B	OLIG2	

Section §13: Overview of PBMC Data

We note that the Peripheral Blood Mononuclear Cells (PBMC) data is used in §4.7. Here, we explain the cell classes and groupings for these 9 different PBMC subtypes (based on the respective clusters that we uncover for them).

§13.1: Cell classes in Cell Marker 2.0

We note the following relevant cell classes with cell markers provided by the Cell Marker 2.0 database:

1. **B Cell**: A major type of lymphocyte involved in antibody production. It corresponds directly to the "B cells (C3)" category in PBMCs, encompassing naïve and memory B cells.
2. **Dendritic Cell**: Antigen-presenting cells that play a critical role in initiating and regulating immune responses. This corresponds to "Dendritic cells (C7)" in the PBMC subtypes, covering multiple dendritic cell (DC) types, including myeloid and plasmacytoid DCs.
3. **Monocyte**: A type of white blood cell that can differentiate into macrophages and dendritic cells. It is represented by "CD14 Monocytes (C1)" and "FCGR3A Monocytes (C5)" in the PBMC subtypes. CD14 monocytes are classical monocytes, while FCGR3A monocytes are also known as non-classical or intermediate monocytes.
4. **Naive CD4 or CD4+ T Cell**: A subset of T cells that have not yet encountered antigens. These cells are part of "Naïve CD4 T cells (C0)" in the PBMC group.
5. **Myeloid Dendritic Cell**: A subtype of dendritic cells derived from myeloid lineage, distinct from plasmacytoid dendritic cells, often involved in antigen presentation and T-cell activation. These are encompassed in the "Dendritic cells (C7)" category.
6. **Natural Killer (NK) Cell**: Lymphocytes that play a crucial role in the innate immune response, capable of killing virus-infected cells and tumor cells without prior sensitization. They correspond directly to "Natural Killer cells (C6)" in PBMCs.
7. **Plasma Cell**: Differentiated B cells responsible for producing antibodies. While they are derived from B cells, they are generally less represented in standard PBMC profiles but are part of the broader "B cells (C3)" lineage.
8. **Myeloid Cell**: This is a broad category encompassing cells derived from the myeloid lineage, including monocytes, granulocytes, and myeloid dendritic cells. "CD14 Monocytes (C1)" and "FCGR3A Monocytes (C5)" fall under this myeloid lineage.
9. **Memory B Cell**: A subset of B cells that have encountered antigen previously and can respond more rapidly upon re-exposure. They are a part of the "B cells (C3)" grouping in PBMCs.
10. **Central Memory CD4+ T Cell**: Memory T cells that have a high capacity for proliferation and can differentiate into effector cells. They would be part of the "Memory CD4 T cells (C2)" category.
11. **Central Memory T Cell**: This can encompass both CD4+ and CD8+ memory T cells. CD4+ versions are part of "Memory CD4 T cells (C2)," while CD8+ central memory cells would align with "CD8 T cells (C4)" in the PBMC subsets.
12. **Effector Memory CD4+ T Cell**: These are memory T cells that have a quick ability to produce effector functions like cytokine production. They fall under "Memory CD4 T cells (C2)" but are distinguished from central memory T cells by their immediate effector capabilities.
13. **Multilymphoid Progenitor Cell**: A progenitor cell capable of giving rise to various lymphoid cell types, including B cells, T cells, and NK cells. These progenitor cells are generally not differentiated in typical PBMC datasets but give rise to many cells in these categories.
14. **Naive CD8+ T Cell**: These are T cells that have not encountered antigens and are ready to respond to new infections. They are part of the broader "CD8 T cells (C4)" category in PBMCs.
15. **CD8+ T Cell**: This is a general category of cytotoxic T cells, including naïve, effector, and memory subsets. They correspond to "CD8 T cells (C4)" in the PBMC subtypes.
16. **CD141+CLEC9A+ Dendritic Cell**: A specialized type of dendritic cell, often referred to as a cross-presenting DC due to its ability to present antigens to CD8+ T cells. It would fall under the "Dendritic cells (C7)" category but is a more specialized subset not typically distinguished in PBMC subsets.
17. **Plasmacytoid Dendritic Cell**: A type of dendritic cell known for producing large amounts of interferon in response to viral infections. This is also part of "Dendritic cells (C7)" in PBMCs.
18. **AXL+SIGLEC6+ Dendritic Cell**: Another subset of dendritic cells with specific markers (AXL and SIGLEC6), which are involved in immune regulation. This subset is part of the "Dendritic cells (C7)" grouping in PBMCs but is not often specifically identified in standard profiling.

We note that these cell classes correspond to our 9 PBMC subtypes in the following way. That is, this is a summary of the grouping relationships to PBMC subtypes:

- **Naïve CD4 T cells (C0)**: Corresponds to naïve CD4+ T cells.
- **CD14 Monocytes (C1)**: Classical monocytes.
- **Memory CD4 T cells (C2)**: Memory CD4+ T cells, including central and effector memory subsets.
- **B cells (C3)**: Includes both naïve and memory B cells.
- **CD8 T cells (C4)**: Includes naïve, central memory, and effector CD8+ T cells.
- **FCGR3A Monocytes (C5)**: Intermediate or non-classical monocytes.
- **Natural Killer cells (C6)**: NK cells.
- **Dendritic cells (C7)**: A broad category covering various dendritic cell subtypes, including myeloid, plasmacytoid, CD141+CLEC9A+, and AXL+SIGLEC6+ dendritic cells.
- **Platelet cells (C8)**: Not directly related to the immune cell categories listed above but represents platelets in circulation.

§13.2: PBMC Sub-cell-type groupings:

The following groupings help us analyze the PBMC data at a higher level based on shared lineage, function, activation status, or immune roles. We note that there are many different possible ways to group these 9 PBMC sub-cell types.

**Category**	**Groupings**	**PBMC Sub-types**	**Explanation**
1 **Lineage-Based Grouping**	Lymphoid Lineage	*Naïve CD4 T cells (C0), Memory CD4 T cells (C2), CD8 T cells (C4), B cells (C3), Natural Killer cells (C6)*	These cells originate from the common lymphoid progenitor and are primarily involved in adaptive immunity (T and B cells) or innate immunity (NK cells).
	Myeloid Lineage	*CD14 Monocytes (C1), FCGR3A Monocytes (C5), Dendritic cells (C7), Platelet cells (C8)*	These cells originate from the common myeloid progenitor and are associated with innate immune responses, phagocytosis, antigen presentation, and inflammation.
2 **Functional Grouping**	T Lymphocytes (T Cells)	*Naïve CD4 T cells (C0), Memory CD4 T cells (C2), CD8 T cells (C4)*	All T cell types that differ in their roles: naive for adaptive immunity initiation, memory for secondary immune response, and CD8 T cells for cytotoxic activity.
	Antigen-Presenting Cells (APCs)	*CD14 Monocytes (C1), FCGR3A Monocytes (C5), Dendritic cells (C7), B cells (C3)*	Cells involved in presenting antigens to T cells and initiating immune responses.
	Effector/Phagocytic Cells	*Monocytes (C1, C5), Natural Killer cells (C6), Platelet cells (C8)*	Cells that actively respond to infection or injury through phagocytosis, cytotoxic activity, or wound healing.
3 **Innate vs. Adaptive Immune Cells**	Innate Immune Cells	*CD14 Monocytes (C1), FCGR3A Monocytes (C5), Natural Killer cells (C6), Dendritic cells (C7), Platelet cells (C8)*	These cells are part of the rapid, non-specific immune response.

	Adaptive Immune Cells	*Naïve CD4 T cells (C0), Memory CD4 T cells (C2), CD8 T cells (C4), B cells (C3)*	These cells provide a specific and long-lasting immune response through antigen recognition and memory formation.
4 **Activation and Maturation Status**	Naïve vs. Activated/Memory Cells	○ *Naïve Cells: Naïve CD4 T cells (C0)* - Immature cells that haven't encountered an antigen.<br>○ *Memory/Activated Cells: Memory CD4 T cells (C2), CD8 T cells (C4)* - Previously activated T cells that have encountered an antigen and differentiated into effector or memory cells.	
	Resting vs. Activated Cells:	○ *Resting/Quiescent Cells: Naïve CD4 T cells (C0), B cells (C3)*<br>○ *Activated Cells: Memory CD4 T cells (C2), CD8 T cells (C4), Monocytes (C1, C5), NK cells (C6), Dendritic cells (C7)*	
5 **Cytotoxic vs. Helper/Regulatory Cells**	Cytotoxic Cells	*CD8 T cells (C4), Natural Killer cells (C6)*	Cells that kill infected or cancerous cells directly through cytolytic activity.
	Helper/Regulatory Cells	*Naïve CD4 T cells (C0), Memory CD4 T cells (C2)*	Helper T cells that assist in coordinating immune responses by secreting cytokines.
6 **Monocyte Subtypes**	Classical vs. Non-Classical Monocytes	*Classical Monocytes (C1): CD14 Monocytes*	Highly phagocytic cells involved in inflammation and pathogen clearance.
		Non-Classical Monocytes (C5): FCGR3A Monocytes	Less phagocytic, involved in patrolling and tissue repair.
7 **Effector Functions**	Cytokine Secreting Cells	*Memory CD4 T cells (C2), CD8 T cells (C4), Natural Killer cells (C6), Monocytes (C1, C5)*	Cells that secrete cytokines to modulate the immune response.
	Phagocytic Cells	*Monocytes (C1, C5), Dendritic cells (C7)*	Cells that ingest pathogens or debris as part of immune defense or tissue homeostasis.
8 **Blood-Related vs. Tissue/Immune-Related**	Blood Cells	*Platelet cells (C8), B cells (C3), Monocytes (C1, C5)*	Primarily involved in hemostasis, immunity, and circulating in the blood.
	Immune Effector Cells	*T cells (C0, C2, C4), Natural Killer cells (C6), Dendritic cells (C7)*	Cells involved in immune surveillance, response, and tissue immunity.

Section §14: Evaluating PPI Predictions

§14.1 Validate TF-TF Coordination Link Prediction Scores for True Signals based on new PPINs

We benchmark the performance of our predicted cell-type TF-TF coordination scores $\bar{B}$. We run NetREm with an older STRINGdb v11 network as a proxy for older information. We use the newer V12 (and beyond) network as current, updated information to gauge NetREm's ability to detect future links). Indeed, these PPINs evolve from v11 to v12. For

instance, in mESCs, our fully-connected TF-TF PPIN comprises known (v11: 5,044; v12: 6,856) and artificial (v11: 13,871; v12: 12,059) TF-TF edges for 195 candidate TFs. In fact, 25 singleton TFs in v11 have known PPIs in v12.
Valid discoveries (False Negatives (FNs)) are found only in the newer, updated STRING db version 12 (v12), and are unknown in the older STRING v11 (input for NetREm); such discoveries show NetREm's ability to learn future PPI links of indirect/direct TF-TF interactions that may be unknown at the current time.
Unknown links are those that have not been mentioned in both networks (i.e. STRING v11 and STRING v12 do not mention them); such links can nonetheless be future discoveries (*False Negatives (FNs)*) or truly *True Negatives* (TNs: no meaningful direct/indirect interaction truly exists between them in biology).
V11 links are found in STRING v11; these links may still be in V12 networks: *Known (both)* or may have been *False Positives (FPs)* if they are no longer found in the newer STRING v12 network: *Removed*).
Non-V11 links are those that are not in STRING v11. They are artificially added with the default edge weight (typically $w = \eta = 0.01$) and could be FNs (discovered in V12 or beyond) or TN (truly unknown).
We have these groupings for the magnitude of cell-type TF-TF coordination scores $|\bar{B}|$. We perform 1-sided Welch statistical tests (please see **Table S15**) to determine if there are any statistically significant coordination scores across the key groups of interest. Please note these 8 goals that we do (where all groups in a) category are tested to see if they are statistically significantly higher than those in groups b) for goals 1 to 7. In goal 8, it is the reverse where we test if group a) is less than group b). The TF-TF links we are comparing are $|\bar{B}|$.

1. Comparing known TF-TF links (v11) with those that are not found in that PPIN. Are those that are Known in v11 higher than those that are unknown in V11 network?
    a. Known in v11
    b. Unknown in v11
2. Comparing Known and Retained TF-TF links with Known and Removed TF-TF links. Are those links that are Known and Retained having higher magnitude of TF-TF scores $|\bar{B}|$?
    a. Known in v11 and kept in v12
    b. Known in v11 and removed in v12
3. Comparing Valid discovery TF-TF links (not found in v11 but in v12) higher than links that are still unknown in v12? Goal: Issue of positive unlabeled learning. Can we still identify some novel TF-TF links that had yet to be discovered?
    a. Not in v11 but found in v12
    b. Not in v11 and not in v12
4. Comparing Valid discovery TF-TF links (not found in v11 but in v12) higher than links that are removed (in v11 but not in v12)?
    a. Not in v11 but found in v12
    b. In v11 but not in v12
5. Comparing known TF-TF links (v11) with those that are valid discoveries. Another way to see the impact of PPI network weights on coordination scores.
    a. In v11
    b. Not in v11 but found in v12
6. Comparing known and retained TF-TF links (v11) with those that are valid discoveries.
    a. In v11 and in v12
    b. Not in v11 but found in v12
7. Comparing Unknown TF-TF links (not found in v11 or in v12) higher than links that are removed (in v11 but not in v12)? Goal: see if NetREm coordination scores can predict links that may be removed in future releases. That is, removing False Positives
    a. Not in v11 and not in v12
    b. In v11 but not in v12
8. Comparing Unknown TF-TF links (not found in v11 or in v12) lesser than links that are removed (in v11 but not in v12)? Goal: see if NetREm coordination scores can predict links that may be removed in future releases. That is, removing False Positives
    a. Not in v11 and not in v12
    b. In v11 but not in v12

Key Findings:
Our findings underscore NetREm's capacity to discern between different types of TF-TF links, demonstrating a nuanced understanding of PPI network evolution:

- *Prioritization of True Positives:* $\bar{B}$ is significantly higher for TF-TF links identified as novel discoveries in v12 compared to those removed (indicative of FPs) in the transition from v11 to v12. This suggests NetREm's effectiveness in predicting meaningful future TF-TF interactions.
- *Recognition of Known Links:* Across multiple scenarios, including mESCs and mDCs with varying sparsity parameters α, β fixed at 1), $\bar{B}$ consistently favored known links over unknown or newly discovered ones, highlighting NetREm's reliance on established knowledge.
- *Future Discovery Potential:* Despite its preference for known interactions, NetREm also demonstrates a significant capability to predict valid, yet previously unknown TF-TF links, as evidenced by higher $\bar{B}$ scores for discoveries in v12 compared to persistently unknown links. Thus there is some transfer learning that takes place.

Our detailed exploration and benchmarking of NetREm against evolving PPI databases reveal its sophisticated ability to navigate and predict the complex landscape of TF-TF interactions, affirming its value as a powerful tool for understanding cellular mechanisms and their evolution over time.

§14.2 Comparing NetREm Predictions with RTNduals and baseREm across 13 human contexts using an Evaluation PPIN

- Please note that we explain this process in Section §10.7.

§14.3 Integrated Interactions Database (IID) for Contextual PPI Annotations

We employ an integrated Contextual PPID (Kotlyar et. al 2022) of dummy (0 = absent, 1 = present) values, annotated for various diseases/tissues/traits/conditions, as a validation tool for TF-TF links. That is, this IID has annotated PPI links to help assess the validity of predicted TF-TF links. We note that this contextual IID has tissue specific PPI networks across species. We use the current version 2021-05 (https://iid.ophid.utoronto.ca/) for annotated PPI networks for humans; this IID database has been considered as a global core biodata resource. It annotates PPIs with comprehensive information about: conditions that they may occur in (e.g., developmental stages, tissues), conservation across species, directionality, potential as drug targets, duration, membership in protein complexes comprising more than 2 proteins. There are over 243 context-specific terms. This data resource integrates PPIs that have been experimentally detected from 10 curated databases: BCI, BIND, BioGRID, DIP, HPRD, I2D, InnateDB, IntAct, MatrixDB, MINT. Currently, this is the only database that has context-specific PPI networks for common model organisms and domesticated species.

This is another example of positive unlabeled learning; to adjust for this, we undersample majority class 0. For machine learning analysis tasks, we use B scores of TF-TF links for each TG in a matrix. We have a matrix of TF-TF link coordination scores (i.e. embeddings) as rows, for respective TG as columns. Overall, NetREm provides a holistic approach to understanding cell-type-specific GRN mechanisms and enriches our understanding of core cell-type interactions (direct/indirect) among TFs involved in these processes. We assigned 0 to any links that are missing from the contextual PPI; thus, links with 0 value may be a True Negative (TN) or currently unknown.

Schwann cells: We focus on Contextual PPI links annotated for Peripheral Nervous System (PNS) neoplasms, and other nervous system-related diseases or disorders.

Alzheimer's disease (AD): We use this Contextual PPI database, which categorizes these links as either associated with AD or currently unassociated. In addition, we employ machine learning to identify TGs predictive of neurodegenerative diseases across eight cell types. For this task, we opt to focus on neurodegenerative diseases at large, rather than AD specifically, to expand the pool of positive links.

Next, for each cell-type, we obtain the B matrix of coordination scores representing TF-TF links for each TG in the AD stage: $\widetilde{B_{AD}}$. We also obtain $\widetilde{B_{Control}}$, which refers to the B matrix of TF-TF coordination scores for each TG in the Control stage for that cell-type. We note that values in $\widetilde{B_{AD}}$ and $\widetilde{B_{Control}}$ will be between -100 and 100 based on our definition of coordination scores. Thus, we do not perform any scaling on these coordination scores and note that both matrices should be on a comparable scale of values. Then, we perform 0 padding to ensure that both $\widetilde{B_{AD}}$ and $\widetilde{B_{Control}}$ matrices have the same dimensions corresponding to the same TF-TF links and TGs, where TF-TF links are rows and TGs are columns. We

then compute $\widetilde{B_{Final}} = \widetilde{B_{AD}} - \widetilde{B_{Control}}$, to obtain our final matrix of net changes in TF-TF coordination scores for the cell-type TGs. That is, these differences in B values between AD and controls across TGs are used to create $\widetilde{B_{Final}}$. Using the Contextual PPI database (Kotlyar et. al 2022), we label these TF-TF links as either related to neurodegenerative diseases (class 1) or not (class 0). Due to class imbalance and potential for false negatives (FNs) in class 0, we undersample majority class 0 to create a balanced dataset for each cell type (via imblearn.undersampling RandomUnderSampler(Lemaître et al. 2017)). We train various machine learning models based on this balanced data.

We train a Random Forest Classifier model using all defaults (e.g. 100 trees, gini estimator for split). We also train XGBoost classifier models (via sklearn XGBClassifier (Pedregosa et al. 2011)) with the logloss evaluation metric and all other defaults. Further, we train Naïve Bayes, Support Vector Machine (SVM), and Logistic Regression models using the respective defaults. Model performance is evaluated using stratified 5-fold Cross Validation (CV), with metrics for each fold, including: accuracy, Receiver Operator Characteristic (ROC) curves, and Precision-Recall curves. We report averaged values across all 5 folds for: area under the ROC curve (AUC), area under Precision-Recall curve (AUPR), and accuracy. Our final classifiers are trained on the entire dataset for each cell type, and we filter the genes to select the top 500 most influential TGs for each cell type (based on highest feature importance gain values), which may serve as potential biomarkers for neurodegenerative diseases, warranting further investigation. We conduct Gene Enrichment Analysis to ensure these identified TGs are biologically meaningful and relevant.

In addition, we use the Contextual IID to determine any biologically-meaningful annotations for top cell-type TF-TF coordination links $\bar{B}$. Currently, this Contextual IID does not have cell-type PPI annotations. Nonetheless, our findings may show NetREm may typically prioritize cell-type and/or context-specific TF-TF coordination links. That is, this is a proxy for determining how cell-type-specific our TF-TF coordination networks can be (i.e. potential cell-type TF-TF PPINs). For the given cell-type, we retrieve cell-type TF-TF links that are relatively strong $|\bar{B}| \geq 85$. We overlay those with annotated Contextual IID links, computing the % of top TF-TF links enriched for each of the 243 different annotations. We rank the results, where low ranks represent a greater %.

Please note that we also use this Contextual IID as an input PPIN (section **§5.1.12**) for benchmarking NetREm against baseREm and RTNduals.

§14.4 Using Cut&Run data and ChIP-seq data to predict TF-TF colocalization and coordination

We use experimental Cut&Run data and ChIP-seq data for TF binding to help validate our predictions of TFs binding on shared regulatory elements to regulate their TGs. These rae genome-wide experimental methods that profile a given TF and determine DNA binding predictions. Analyzing the TF binding profiles can be used to determine cooperativity among TF pairs and identify new relationships that may be undetected in existing PPI networks. We use this data for 8 core TFs in Schwann cells (in Application #11) as well as for 5 TFs in neural cells (in Application #12).

Section §15: Alzheimer's disease versus Controls: Additional Information for Application 12

§15.1 Comparing NetREm with scNET

We wanted to do comparison analysis of NetREm's TF-TG regulatory networks for an example of NetREm applied with prior reference GRN info derived from multi-omics data. Here, we note that NetREm and scNET (Gupta et al. 2022) are applied to the same preprocessed gene expression data across control and Alzheimer's disease (AD) stages for 4 primary central nervous system (CNS) cell types in Application #11. Notably, scNET employs the scGRNom pipeline (Jin et al. 2021) to construct TF-regulatory element (RE)-TG networks using an ElasticNetCV framework, in contrast to the PPI-network-regularized regression framework used by NetREm. Consequently, we compare and assess the TF-TG regulatory network outputs from both NetREm and scNET. Indeed, most real biological networks are known to exhibit scale-free properties (Barabási and Oltvai et. al 2004). So, our new comparison will highlight how NetREm's predicted GRNs potentially capture these characteristics better than scNET's respective GRNs do; we also show how NetREm's TF-TG regulatory networks are closer to brainSCOPE GRNs (proxy for ground truth GRNs) than scNET's GRNs are.

§15.2 Evaluating if Predicted TF-TG Regulatory Networks Exhibit Scale-Free Topology

To evaluate whether NetREm's predicted GRNs exhibit scale-free topology, we follow a procedure similar to those used in previous studies that focus on assessing degree exponent (γ) and coefficient of determination (R^2) values. Here's a step-by-step guide we use in our evaluation process:

1. Construct Degree Distribution:
- First, for each predicted GRN, we construct the degree distribution by counting the # of TFs or TGs that have a particular degree (i.e., number of connections). We plot this distribution on a log-log scale, where the x-axis represents the degree and the y-axis represents the frequency of nodes with that degree.

2. Fit the Degree Distribution to a Power Law:
- Fit the degree distribution data to a power-law model, which is characterized by the probability $P(k)$ a node has degree k: $P(k) \sim k^{-\gamma}$. Here, γ is the degree exponent, which indicates how rapidly the degree distribution decays.

3. Estimate the Degree Exponent (γ):
- Using statistical tools or software packages like the powerlaw Python package, estimate the degree exponent γ from your fitted model.
- Ensure the fit is robust by analyzing the goodness of fit.

4. Calculate the Coefficient of Determination (R^2):
- To quantify how well the power-law model fits the observed data, calculate the coefficient of determination R^2.
- R^2 values close to 1 indicate a strong linear relationship on the log-log scale, suggesting that the network might follow a scale-free distribution.

5. Interpret the Results:
- Compare the obtained γ and R^2 values with those from known biological networks or other similar studies to assess the similarity.
- Typical values for γ in real biological networks often range between 2 and 3. If the networks exhibit similar values, this suggests that the GRNs might be capturing the scale-free nature observed in biological systems.

This evaluation helps quantify the scale-free topology of NetREm's predicted GRNs, providing insights into how well the predicted networks mirror the properties of real biological networks. We endeavor to evaluate the γ and R^2 values of NetREm's predicted GRNs to quantify their scale-free topology.

§15.3 Utilizing the brainSCOPE resource as a ground truth GRN in Control Microglia and Oligodendrocytes

In this analysis, the goal is to compare two different gene regulatory network (GRN) inference methods—NetREm and scNET—to a "ground truth" GRN provided by the brainSCOPE resource. That is, the brainSCOPE resource is used as a proxy for a ground truth GRN given its vast resources. The comparison focuses on specific cell types, namely Microglia and Oligodendrocytes, which are common to all three networks. Both NetREm and scNET are applied to the same gene expression dataset to infer GRNs 3 However, brainSCOPE, which acts as the ground truth, has been constructed using more comprehensive data, providing a broader and likely more accurate regulatory map of the gene networks for these cell types in the brain.

Please note that we utilize these filtered brainSCOPE, NetREm, and scNET networks for our comparisons of Jaccard Similarity and our signed AUPR values of signed TF-TG regulatory networks between scNET and NetREm in Microglia and Oligodendrocytes. Please see the 2 subsequent, consecutive sections for more details.

§15.4 Comparing Jaccard Similarity of Signed TF-TG Regulatory Links between NetREm and scNET

The comparison involves examining how well the networks inferred by NetREm and scNET align with the brainSCOPE GRN, and this is done using signed Jaccard similarity on TF and TG interactions.

Methods Involved in the Comparison

1. Defining the Components of the GRN

Each network (NetREm, scNET, brainSCOPE) consists of regulatory interactions between transcription factors (TFs) and their target genes (TGs). Each interaction also includes a **sign**, which indicates the direction of the regulation:

- **Positive sign (+)**: The TF activates the expression of the TG.
- **Negative sign (-)**: The TF represses the expression of the TG.

For each network, we create a unique identifier that encapsulates this regulatory relationship:
TF-sign-TG where Sign is (+) for activating relationships and (-) for repressing relationships. This uniquely represents each interaction, taking into account both the specific TF-TG pair and the regulatory direction (sign).

2. Filtering for Common Genes: Please note that we use the filtered networks described below

Here, we describe the process of filtering the 3 networks (for each of the 2 contexts: Control Microglia and Control Oligodendrocytes (Oligo)) to help us evaluate the performance of NetREm versus scNET against the corresponding filtered network from brainSCOPE.

For each of the 2 contexts, please note that we do the following:
- Step 1: *Controlling for common genes across all 3 networks*
  - 1a: Pool together all the genes (TFs and/or TGs) for each of the 3 networks. Then, find the common genes across all 3 networks.

	Control Microglia	Control Oligodendrocytes
# of common genes	1,203	944

  - 1b: Next, we filter the brainSCOPE TF-TG regulatory network (i.e. GRN) so that TFs and TGs belong to the respective group of common genes across all 3 networks. This is for ease of comparison. We also filter the NetREm and scNET networks for these common genes.

That is, given that the datasets (NetREm, scNET, and brainSCOPE) may contain different sets of genes, we first filter for common genes across the 3 networks for each cell type (Microglia, Oligodendrocytes). This ensures a fair comparison by limiting the analysis to shared TFs and TGs:
- NetREm Genes: The union of all TFs and TGs in NetREm.
- scNET Genes: The union of all TFs and TGs in scNET.
- brainSCOPE Genes: The union of all TFs and TGs in brainSCOPE.

The intersection of these gene lists is used to filter each dataset so that only interactions involving common genes are retained.
- Step 2: *Further filtering scNET and NetREm networks based on respective filtered brainSCOPE network*. We further filter the scNET and NetREm networks for only
  - TFs that are found as TFs in brainSCOPE's filtered GRN (from Step 1).
  - TGs that are found as TGs in brainSCOPE's filtered GRN (from Step 1).

Thus, we obtain our filtered networks for comparison. Please note that this table below delineates the counts and metrics for each of those steps.

Filtering the 3 TF-TG Regulatory Networks for Each Context for Comparison									
**Method for GRN Inference**	**Control cell-type**	**Original**		**After Step 1 Filtering**		**After Step 2 Filtering (Final Filtered Networks)**			
		TFs	*TGs*	*TFs*	*TGs*	*TFs*	*TGs*	*# of TFs as TGs*	*# of TF-sign-TG regulatory links*
BrainSCOPE		346	8,694	173	1,094	173	1,094	137	15,582
NetREm		277	11,838	169	1,196	141	1,088	114	71,720
scNET	Microglia	203	1,360	182	1,059	158	986	30	53,431
BrainSCOPE	Oligo.	366	8,634	170	855	170	855	130	10,175

NetREm		261	10,966	136	933	114	846	94	72,071
scNET		278	996	190	782	157	734	21	44,684

All 3 networks return signs for the predicted regulation activity. That is, TFs have coefficients where negative coefficients correspond to potential repressor roles and positive coefficients for potential activator roles in regulating the given TG.

3. Signed Jaccard Similarity Calculation
The Jaccard similarity is used to quantify how similar the regulatory networks are between brainSCOPE and NetREm/scNET for each cell type. The signed version takes into account the directionality of the regulatory interactions. Here's the process for calculating the similarity:

- Intersection (and):
  - The intersection between two sets (NetREm and brainSCOPE, or scNET and brainSCOPE) consists of all the regulatory interactions (TF-sign-TG) that are present in both networks, including the direction of regulation. For example, both networks must agree that TF1 positively regulates TG1 for the interaction to be included in the intersection.
- Union (or):
  - The union consists of all unique regulatory interactions from both networks, considering both shared and distinct interactions. For instance, if TF1 regulates TG1 in one network but not the other, it will still contribute to the union.

The Jaccard Similarity (JS) is calculated as the ratio of the intersection to the union:

$$JS(S_1, S_2) = \frac{|S_1 \text{ and } S_2|}{|S_1 \text{ or } S_2|}$$

Where S_1 and S_2 are the sets of TF-sign-TG interactions for a pair of networks (e.g., brainSCOPE vs. NetREm or brainSCOPE vs. scNET).

- Numerator: The size of the intersection—how many TF-TG-sign interactions are shared between the two networks.
- Denominator: The size of the union—all unique interactions across both networks.

This results in a score between 0 and 1:

- 1: Perfect overlap, meaning all interactions are the same between the two networks.
- 0: No overlap, meaning no shared interactions.

The comparison is conducted separately for NetREm and scNET against brainSCOPE.

4. Analyzing Results for Microglia and Oligodendrocytes
The analysis is carried out individually for each cell type:

- Microglia: A set of GRNs from NetREm, scNET, and brainSCOPE is constructed for this cell type. Jaccard similarity is calculated for both NetREm vs. brainSCOPE and scNET vs. brainSCOPE.
- Oligodendrocytes: Similarly, the networks are generated and compared using Jaccard similarity.

This gives two distinct sets of comparisons:

1. NetREm vs. brainSCOPE (Microglia and Oligodendrocytes).
2. scNET vs. brainSCOPE (Microglia and Oligodendrocytes).

Additional Metrics for Evaluation
Apart from Jaccard similarity, other aspects of the networks are also compared:

1. Number of TFs and TGs: The number of unique transcription factors and target genes in each dataset is compared before and after filtering for common genes. This helps ensure that the comparisons are being made on a consistent set of genes.
2. TFs acting as TGs: In some cases, transcription factors can also act as target genes. The number of such instances is tracked across the datasets, providing insight into more complex regulatory roles.
3. Shared TFs and TGs: Beyond Jaccard similarity, the number of common TFs and TGs between the datasets is also reported to understand overlap at the gene level, separate from their regulatory interactions.

Conclusion and Insights

By comparing **NetREm** and **scNET** to the **brainSCOPE** ground truth for Microglia and Oligodendrocytes, we aim to determine which method yields GRNs more similar to the ground truth, with a focus on both the regulatory pairs and the direction of regulation. The **signed Jaccard similarity** provides a quantitative measure of this alignment.

- **Higher Jaccard similarity** indicates that the inferred network captures both the correct regulatory interactions and the correct signs (activation vs. repression).
- This method allows for a direct comparison of how well each inference method performs for the given cell types using the same input data.

The result of this analysis will inform us about the relative accuracy of NetREm and scNET in modeling cell-type-specific regulatory networks, potentially guiding future GRN inference methods or validation efforts using additional datasets.

§15.5 Signed Weighted AUPR Calculation for Regulatory Networks

Data Sources and Preparation
We utilized 2 predictive regulatory datasets, **NetREm** and **scNET**, and compared them against the **brainSCOPE** dataset, which served as the ground truth. The NetREm and scNET datasets contained TF to target gene (TG) regulatory edges, each with coefficients representing the strength of the interaction. The brainSCOPE dataset provided signed correlation values for TF-TG pairs, which we used as a baseline for comparison. The primary goal was to evaluate which predictive model, NetREm or scNET, more accurately predicted the TF-TG regulatory edges when compared to brainSCOPE using a signed Area under the Precision-Recall Curve (AUPR) approach.

Focus on Control Microglia and Control Oligodendrocytes
We conducted this analysis on two specific cell types, **Control Microglia** and **Control Oligodendrocytes**, since these were the common cell types for which predicted networks were available from all three sources: NetREm, scNET, and brainSCOPE. While regulatory elements predicted by each model may differ due to the integration of multi-omics data, we focused solely on comparing the final **gene expression-derived networks** from NetREm and scNET, as both models were applied to the same underlying gene expression data
.
Filtering for Common Transcription Factors and Target Genes
To ensure valid comparisons between the datasets, we first identified the common transcription factors (TFs) and target genes (TGs) shared between the 2 networks. That is, for (NetREm, brainSCOPE) AUPR calculations, we focus on the common TF-TG pairs (ignoring sign) between NetREm and brainSCOPE for that context. Similarly, for (scNET, brainSCOPE) AUPR calculations, we focus on the common TF_TG pairs (ignoring signs) between scNET and brainSCOPE. Thus, TF-TG pairs that are not present in both datasets are excluded from the analysis; this is done to recognize that the networks have been constructed using different assumptions and multiomics data.

Signed AUPR Calculation
For each filtered TF-TG pair, we categorized the edges into positive and negative edges based on the sign of the correlation in the brainSCOPE dataset. If the ground truth correlation for a TF-TG pair was positive, it was classified as a positive edge; if negative, it was classified as a negative edge.
The coefficients from the NetREm and scNET datasets were used as predicted scores for the corresponding TF-TG pairs. For each pair, the following steps were performed:

1. *Positive Edge AUPR:* For TF-TG pairs with positive correlations in brainSCOPE, we calculated the AUPR using the positive coefficients from the NetREm and scNET datasets as the prediction scores. The Precision-Recall curve was generated, and the area under the curve (AUPR) was calculated using the scikit-learn precision_recall_curve function.
2. *Negative Edge AUPR:* For TF-TG pairs with negative correlations in brainSCOPE, the AUPR was calculated using the corresponding negative coefficients from NetREm and scNET.
3. *Averaging AUPR:* The final AUPR was computed by averaging the AUPR values for both positive and negative edges, providing a balanced measure of the performance of the predicted regulatory networks in capturing both activating and repressing TF-TG interactions.

Handling Edge Cases
We incorporated checks to ensure the robustness of the calculations. If either the positive or negative edge sets were empty or lacked sufficient variation in the y_true labels (i.e., no positive or negative edges), the AUPR for that category was set to zero. The final AUPR was computed only if valid values were available for both positive and negative edges. If no valid TF-TG pairs were found after filtering, the method returned zero for the signed AUPR.

Summary of Steps:

1. *Data Filtering:* Filter TF-TG pairs to include only those present in both datasets (NetREm, scNET, and brainSCOPE).
2. *Edge Classification:* Classify edges as positive or negative based on the sign of the correlation in the brainSCOPE dataset.
3. *AUPR Calculation:* Calculate AUPR separately for positive and negative edges using coefficients from NetREm and scNET as predicted scores.
4. *Averaging:* Compute the final AUPR by averaging the results from both positive and negative edge sets.

Comparison Between NetREm and scNET

The final goal was to compare the AUPR values from **NetREm** and **scNET** against brainSCOPE in both Control Microglia and Control Oligodendrocytes. By focusing on AUPR, we aimed to identify which model, NetREm or scNET, provided more accurate predictions of TF-TG regulatory interactions. This methodology allows for a robust evaluation of how well these models capture transcriptional regulation, independent of the specific regulatory elements predicted, as both were applied to the same underlying gene expression data.

§15.6 Neural Cell TF-clusters ChIP-seq tracks

We use raw data for the ENCODE3 TF clusters track (**encRegTfbsClusteredWithCells.hg38.bed.gz**) from UCSC Genome Browser (Transcription Factor ChIP-seq Data Standards and Processing Pipeline – ENCODE (encodeproject.org)). We analyze our results for cell-type TF-TF cooperativity in Control Excitatory Neurons and Control Inhibitory Neurons (from our Application 11) for these 6 TFs with available data in neural cells (CTCF: EP300, EZH2, MXI1, RAD21, SMC3). We determine the Jaccard Similarity of overlaps between each pair of TFs. We consider even 1 base pair overlap as important and do this overlap analysis using GenomicRanges (Lawrence et. al 2013) in R.

$$JS(TF_i, TF_j) = \frac{\text{\# of ChIP} - \text{seq peaks with overlap in binding for } TF_i \text{ and } TF_j}{\text{\# of peaks bound by } TF_i \frac{\text{and}}{\text{or}} TF_j}$$

Overlap is very important to consider as 2 ChIP-seq experiments by 2 different TF proteins can be analyzed to see if they have a large fraction of peaks with overlap; if so, these TFs may be cooperative in terms of their regulation (Yu et al. 2015).

Neural cells, primarily known as neural stem cells or progenitor cells, can differentiate into various cell types within the nervous system. Their development depends on the signals they receive and their environment, leading to specific lineages. The main cell types neural cells can become include:

1. **Neurons**: These are the primary signaling cells of the nervous system, responsible for transmitting information via electrical and chemical signals. Neurons can be further classified into various types, such as excitatory or inhibitory, based on their function and neurotransmitters.
2. **Glial Cells**: These support and protect neurons and perform various essential functions. The main types of glial cells include:
    - **Astrocytes**: Maintain the extracellular environment, regulate blood flow, provide nutrients to neurons, and are involved in the blood-brain barrier.
    - **Oligodendrocytes**: Produce myelin in the central nervous system (CNS), which wraps around axons to facilitate rapid signal conduction.
    - **Schwann Cells (SCs)**: These are the myelin-producing cells of the peripheral nervous system (PNS).

For our analysis, we consider Controls in Astrocytes, Oligodendrocytes, Inhibitory Neurons, and Excitatory Neurons from our Application 12.

Section §16: Gold Standard TG-Specific TF-TF Coordination

In this section, we explain the validation data that is used for the Application #10 (based on expression data explained in §4.10) for the immune-related subtypes in humans.

§16.1: Ground truth experimental data on TF-TF coordination for TG regulation

Please note that we showcase NetREm's unprecedented ability to identify TG-specific TF-TF coordination links B for diverse contexts. To do this, we utilize experimental findings from a recent study (Berenson et al. 2023) that employed Paired Yeast One-Hybrid (pY1H) experimental Assays to detect DNA-binding cooperativity and antagonism across human TFs.

We navigate to the Supplementary Data and pool data from Supplementary Tables 4 and 8 of this study to yield our resource of TF-TF relationships when regulating the bait gene (i.e. the Target Gene (TG)). This study focused especially on the immune system in humans and on a few cytokine TGs. In fact, they analyze relationships among TFs to regulate these cytokine TGs: *CCL15, CCL2, CCL5, CSF2, CXCL8, IFNB1, IL10, IL12B, IL18, IL32, IL6, TNF, TNFSF15, IL1B*. They provide data on event type, which we organize as follows: cooperative TF-TF interactions (event type: cooperativity) and antagonistic TF-TF interactions (event type: {TF1 antagonized, TF2 antagonized, or mutual antagonism}).

They find antagonistic and cooperative relationships among 137 different human TFs. There are 476 $(\mathrm{TF}_i - \mathrm{TF}_j) - \mathrm{TG}$ pairs (antagonistic: 280, cooperative: 196) in this ground truth data, comprising the 15 TGs and 194 unique TF-TF relationships.

We note that this data lacks cell-type specificity. We utilize the TFs that are found in the ground truth and the TGs that are tested to filter our gene expression data.

§16.2: Running NetREm on 62 filtered gene expression datasets for 62 immune cell subtypes

Given that the ground truth data focused, primarily, on immune-related contexts, we utilize one of the most recent and comprehensive data resources available for the immune system cells: The Tabula Sapiens Atlas (The Tabula Sapiens Consortium 2022) for humans. There are over 260k different cells spanning 62 different cell subtypes of the immune system. Please note that this is our Application #10. Also, please note that these datasets are pooled across various tissues and that for sheer simplicity, we did not break apart the single-cell datasets by the tissue type.

We filter these 62 single-cell gene expression datasets for these 15 TGs as well as for the 137 TFs. We uncover 125 TFs (out of the 137) in the gene expression datasets, which is our $\mathcal{N}$. Thus, we run NetREm (using our main NetREm PPIN) for each of these 15 TGs using the fixed set of $N = \mathcal{N}$ candidate TFs if the TG is not one of the TFs; if the TG is a TF, we simply remove that TF from the list and run NetREm with N = $\mathcal{N} - 1$ candidate TFs.

Please note that for each of our NetREm runs (for each TG across each of the 62 datasets), we utilize the Main NetREm PPIN (§5.1.11) and run NetREm with $\beta = 100$ and $\alpha = 0.1$. We then obtain our TG-specific TF-TF coordination B links for each of the TGs. Inherently, these TF-TF links are non-zero for the TG only if both TFs are final predictors for the TG. Thus, for each $(\mathrm{TF}_i - \mathrm{TF}_j) - \mathrm{TG}$ pair, both TFs are coordinating to co-regulate the TG.

When we are evaluating our TG-specific TF-TF coordination networks B, we are analyzing our outputs on a deeper level. We are evaluating the success of that prediction that both TFs are involved in regulating TG either directly or indirectly. Then, we are going a step further to evaluate our predicted nature of $(\mathrm{TF}_i - \mathrm{TF}_j)$ coordination: do we predict correctly that both TFs cooperate (if it is a cooperative role) or that both TFs are antagonistic (if it is an antagonistic role). Thus, we focus mainly on our success at predicting correct B links: $(\mathrm{TF}_i - \mathrm{TF}_j) - \mathrm{TG}$ links where we accurately predict that both TFs are involved in co-regulating TG as well as the nature of the TF-TF interaction with one another.

We note that TG-specific TF-TF coordination may differ across cell-types (i.e. $(\mathrm{TF}_i - \mathrm{TF}_j) - \mathrm{TG}$ is cell-type-specific), but that we lack this data in our ground truth data. We also understand that $(\mathrm{TF}_i - \mathrm{TF}_j)$ interactions are not only cell-type-specific but also TG-specific and we endeavor to validate NetREm's ability to detect these nuances at the TG-level. We also provide metrics on the predicted cooperation and/or antagonism between the pair of TFs at the immune cell subtype level.

Overall, NetREm's TG-specific TF-TF coordination networks are unprecedented and this analysis helps motivate that the findings may be biologically meaningful. We also note that our results can be enhanced greatly by the incorporation of prior knowledge on GRNs (i.e. TG-specific candidate TFs for the TG), which we did not incorporate in this analysis.

In the future, we anticipate the availability of more studies that analyze TF-TF interactions to co-regulate TGs for additional TGs and contexts. We also hope for such evaluation data to become available at the cell-type and the cell sub-type level. We mainly focus on presenting examples where NetREm's predictions for TF-TF coordination style and TF-TF co-regulation match the ground truth.

Section §17: Gold Standard Gene Regulatory Networks for the Main Applications

We evaluate the TF-TG regulatory network predictions inferred by various models: NetREm (with hyperparameters β and α altered as needed) as well as other models.

Please note that in all our gold standard GRNs (and in all GRNs in general), we removed any links where the TF is the same as the TG. We also ensured that the GRN links contain TGs and TFs that are found in the original gene expression data.

Further, when we run NetREm (and other models), we ensure that we only consider the TFs and TGs that were considered by the gold standard networks, for ease of reference; this is since we cannot corroborate the reason for any missing TFs or TGs in the gold standard networks (so to be safe and not make any assumptions and to also enable ease of comparison, we keep the TFs and TGs as those found in the gold standards (if there are any) in our benchmarking applications). Please note that these current gold standard reference GRNs are work-in-progress (i.e. are partial); nonetheless, they are helpful tools for gauging the effectiveness of the predictions. We are hopeful that in coming years, there will be a surge in available experimental datasets for validation.

We also note that our ground truth GRNs for HSCs, mESCs, mDCs, hPSCs, and YCs simply note TF-TG links and do not provide more information regarding the type of regulatory relationship (e.g. TF represses TG or TF activates TG). To build those ground truth GRNs, we pooled together the validation data files provided for the respective dataset. That is, we use the ground truth GRN data provided by the same study that we use for our final gene expression data.

Nonetheless, benchmarking studies (e.g. (McCalla et al. 2023)) highlight how gold standards for regulatory network inference from scRNAseq datasets are woefully inadequate and suffer from drawbacks.

§17.1: human Embryonic Stem Cells (hESCs)

We have the 5,050 TF-TG signed ground truth GRN links subset from the original atlas of regulatory links(Sharov et al. 2022) for the 1,250 randomly-selected TGs. In addition, we have the sign (+: activate, -: repress), which we can use to evaluate the c^* sign (+ or -) for predicted TF-TG regulatory links. We can use this ground truth GRN for validation as it is the underlying input data for SERGIO to generate gene expression data. In particular, we designed this application so that we would have an exact ground truth GRN to use for our validation.

For hESCs, we have information on the sign of the TF-TG regulatory link; that is, we have positive (+) signs for activator TFs and negative (-) signs for repressor TFs. In hESCs, we refer to True Positive (TP) predictions (correct TF-TG regulatory links predicted) as TF-TG regulatory links that are not only found in the ground truth validation atlas but also have the correct coefficient $c^*_{TF_i}$ where $c^*_{TF_i} > 0$ if TF_i activates TG and $c^*_{TF_i} < 0$ if TF_i represses TG. That is, in hESCs, we can also focus on TF-sign-TG regulatory links where sign can be (+ or -).

Please note that the underlying gene expression data is detailed in §4.1 for Application #1.

§17.2: human Hematopoietic Stem Cells (HSCs)

We utilize the pooled gold standard GRN networks from (Zhang et al. 2023) that mined and curated from experimentally derived networks of regulatory interactions from literature and from existing scientific databases. These are the following gold standard networks that we pool for HSCs: UniBind.gs_network, Cus_ChIP_2, Cus_KO_0.01_Cus_ChIP_2_intersect, Cus_KO_0.01, and Cus_KO_0.01_union_intersect.

We filter the ground truth GRN for only TFs and TGs in the gene expression data. Ultimately, we uncover 10,588 TGs, 178 TFs (177 TFs are also TGs and 1 is a master regulator). There are 303,056 TF-TG links in this ground truth network. Theoretically, 1,884,487 TF-TG links could be possible (where no self-links exist; that is, each TF-TG link contains a TF that is different from the TG).
Please note that the underlying gene expression data is detailed in §4.2 for Application #2.

§17.3: mouse Embryonic Stem Cells (mESCs)

For mESCs, we gather the gold standard networks from mESC_KDUnion, mESC_chipunion, and mESC_chipunion_KDUnion_intersect gold standard GRN networks provided by (Zhang et al. 2023).
Please note that the underlying gene expression data is detailed in §4.3 for Application #3.

§17.4: mouse Dendritic Cells (mDCs)

We gather the gold standard networks (available on zenodo: https://zenodo.org/records/5909090 gold_standard_datasets.zip) provided by (McCalla et al. 2023) for: mDC_chipunion, mDC_chipunion_KDUnion_intersect, mDC_KDUnion. We filter the ground truth GRN for only TFs and TGs in the gene expression data. Ultimately, we uncover 9,087 TGs, 93 TFs (all TFs are also TGs). There are 176,691 TF-TG links in this ground truth network. Theoretically, 844,998 TF-TG links could be possible.
Please note that the underlying gene expression data is detailed in §4.4 for Application #4.

§17.5: human pluripotent stem cells (hPSCs)

For hPSCs, we gather the gold standard networks (available on zenodo: https://zenodo.org/records/5909090 gold_standard_datasets.zip) for hESCs for the same benchmarking study resource (McCalla et al. 2023) that was used to provide input gene expression data and validation data. Here, we use the provided TF-TG data for hESC_chipunion, hESC_chipunion_KDUnion_intersect, hESC_encodechip, hESC_escapechip, hESC_KDUnion, hESC_lit, hESC_logof, hESC_nishiyama. We pool these provided resources together.

We filter the ground truth GRN for only TFs and TGs in the gene expression data. Ultimately, we uncover 7,464 TGs, 170 TFs (all TFs are also TGs). There are 228,902 TF-TG links in this ground truth network. Theoretically, 1,268,710 TF-TG links could be possible.
Please note that the underlying gene expression data is detailed in §4.5 for Application #5.

§17.6: budding yeast cells (YCs)

We gather the gold standard networks (available on zenodo: https://zenodo.org/records/5909090 gold_standard_datasets.zip) provided by (McCalla et al. 2023) for: hu, mac2, yeast_chipunion, yeast_chipunion_KDUnion_intersect, yeast_KDUnion, yeastract_c3, yeastract_t2.
We filter the ground truth GRN for only TFs and TGs in the gene expression data. Ultimately, we uncover 5,355 TGs, 280 TFs (276 TFs that are also TGs and the remaining 4 that are master regulators). There are 49,158 TF-TG links in this ground truth network. Theoretically, 1,499,124 TF-TG links could be possible.
Please note that the underlying gene expression data is detailed in §4.6 for Application #6.

§17.7: Human myelinating (mSCs) and non-myelinating (nmSCs) Schwann cells (SCs)

We have ground truth GRN for only 7 TFs in Schwann cells based on data in rodents (e.g. mice, rats). This is our proprietary GRN that we have made using public data as well as other resources that we have deposited on Gene Expression Omnibus. We use our results for these 7 SC TFs as a proof-of-concept for our other predictions related to mSCs and nmSCs. We simply focus on the loss-of-function (LOF) TGs and the Direct TGs (have LOF data as well as ChIP-seq binding for that TF nearby the TG).
Please note that the underlying gene expression data is detailed in §4.11 for Application #11.

§17.8: Alzheimer's disease (AD) vs. Controls in Central Nervous System (CNS) Cell-Types

We use the recent brainSCOPE resource (available: https://brainscope.gersteinlab.org/) from a recent study (Emani et al. 2024) that has comprehensive TF-TG regulatory links in several cell-type GRNs based on integrating single cell-resolution, multiomics data for 388 adult samples in the human dorsolateral prefrontal cortex (DLPFC) regions. The cell-type GRNs that are returned are based on TF-regulatory element-TG links where TFs are given signs (+ for activator and – for repressor) as well as coefficients. We use this resource to obtain our "ground truth GRN" for validating our TF-TG regulatory predictions in Control Microglia and Control Oligodendrocytes. We primarily focus on the signed TF-TG regulatory links since the regulatory elements (e.g. promoters and enhancers) are defined using different input omics data. Please note that the underlying gene expression data is detailed in §4.12 for Application #12.

Section §18: Training Comparative Methods for TF-TG Regulation (i.e. GRN Inference)

§18.1: The 4 Benchmark Regression Models (BRMs)

We run benchmark regression models (BRMs) to infer TF-TG regulatory links for all of our applications. In fact, in our NetREm software package, we provide implementations for these baseline functions for comparison. Model selection, regularization techniques, and ensemble methods are commonly used to navigate the bias-variance tradeoff, aiming to find a model with an optimal balance between bias and variance. To assess the performance of our NetREm model, we employ a comparative approach involving 4 benchmark regression models (BRMs) as a baseline: ElasticNetCV(Pedregosa et al. 2011), LassoCV(Pedregosa et al. 2011), Linear Regression(Pedregosa et al. 2011), and RidgeCV(Pedregosa et al. 2011), all implemented using Python's scikit-learn (Pedregosa et al. 2011) package. For each target gene (TG) within a specific cell type, we fit NetREm and these benchmark models for the same N candidate Transcription Factor (TF) predictors (where N may vary in number and specific TFs selected across TGs if prior knowledge is incorporated and/or the TG is also a cell-type TF). All 5 models fit a y-intercept. These 4 BRMs enable us to gain a better understanding of NetREm's performance. All models, except for Linear Regression, undergo 5-fold cross-validation (CV) to optimize hyperparameters. These models are applied on the same training data and tested on the same testing data to infer TF-TG regulatory networks for transcription. We can compare them against NetREm using the traditional evaluation metrics (when gold standard reference GRNs are available). In addition, since these are regression methods, we can use evaluate their performance against NetREm using regression-based metrics (e.g. Mean Square Error (MSE)). Statistical significance of performance differences between NetREm and benchmarks is assessed using 1-sided t-tests of whether NetREm consistently yields lower test MSEs.

§18.2: GRNBoost2

We apply the GRNBoost2 machine learning algorithm (Moerman et al. 2019) used to infer gene regulatory networks (GRNs) from gene expression data, particularly in single-cell RNA sequencing (scRNA-seq). It is also part of the SCENIC pipeline and is based on a gradient boosting framework (specifically, LightGBM). GRNBoost2 identifies potential regulatory relationships between TFs and target genes by modeling co-expression patterns across genes. It outputs feature importances, which we use as our scores. We note that GRNboost2 does not provide any signs (i.e. activation vs. repression information is not given) for the TF-TG regulatory links.

§18.3: RTN (used by RTNduals)

The RTN tool stands for Reconstruction of Transcriptional Networks (Fletcher et al. 2013) and it has been designed for bulk RNA-seq dataset but can be adapted to the newer scRNA-seq datasets. Please note that we apply the RTN package to predict a regulatory element based on gene expression data and a list of TFs. That is, RTN uses only gene expression data (`gexpr`) and a list of TFs (`tf_list`: $\mathcal{N}$ cell-type TFs) for the context. It does not incorporate any TG-specific inputs or any additional prior knowledge.

Please note the following commands (in bullets) that we run in R (using the Bioconductor package for RTNduals and/or RTN).

- `rtni <- tni.constructor(gexp, regulatoryElements = tf_list)`

RTNduals checks for regulatory elements. It then performs permutation analysis for the regulons (i.e. TFs), using 1,000 permutations with pValueCutoff of 0.05 (for the adjusted p-value). That is,

- `rtni <- tni.permutation(rtni, nPermutations=1000, pValueCutoff=0.05)`

This outputs a regulatory network based on the RTN package. This regulatory network is inferred by mutual information analysis followed by multiple hypothesis testing corrections.

- `rtni <- tni.bootstrap(rtni, nBootstrap=100)`

Then, it performs bootstrap analysis to assess the stability of the regulatory network.

- `regulons <- tni.get(rtni, what = "regulons.and.mode")`

We obtain the list of TF-TG links (i.e. our RTN-based regulatory network) through this regulons variable. This outputs our TF-TG regulatory network. Please note that we explain (in a later section) how we use the RTN-inferred network to output TF-TF links when we run RTNduals.

§18.4: SCENIC (for mSCs and nmSCs)

SCENIC is designed to infer GRNs from scRNA-seq data. Its main goal is to identify TFs and their TGs, which control gene expression in specific cell types or states. Scenic achieves this by integrating gene expression data with information about transcription factor binding motifs and co-expression patterns, helping to reveal the underlying regulatory mechanisms that drive cellular identity and behavior. SCENIC operates in 3 key steps. First, it uses GRNBoost2 (or GENIE3) to identify potential regulatory links between transcription factors and their target genes based on co-expression relationships. Next, it refines these interactions using RcisTarget, which incorporates motif enrichment analysis to ensure that the predicted target genes contain binding motifs for the respective transcription factors. Finally, the AUCell step assesses the activity of these regulatory networks in individual cells, identifying which transcription factors are active in each cell by scoring gene sets. The result is a detailed map of regulatory interactions that helps researchers understand how transcription factors control cell-type-specific gene expression patterns.

We run the full SCENIC pipeline for the gene expression data in mSCs and for the gene expression data in nmSCs in humans.

§18.5: Output State-of-the-art (SOTA) cell-type GRNs from a benchmarking study

Please note that we use a recent benchmarking study (McCalla et al. 2023) that evaluated various SOTA cell-type GRNs in different organisms and datasets, such as mouse dendritic cells (mDCs), human pluripotent stem cells (hPSCs), and budding yeast cells (YCs). We focus on these 3 applications (mDCs, hPSCs, YCs). This study provides the normalized corresponding scRNA-seq gene expression data for these 3 applications, the validation GRN (pooling different ground truth experimental data), as well as GRNs inferred by several SOTA cell-type GRNs.

These SOTA GRNs return their predicted TF-TG regulatory links by employing various techniques on single-cell data. The benchmarking study noted that adding prior information and estimating the activity of TFs helps improve the performance of cell-type GRN methods like Inferelator and MERLIN, that most of the cell-type GRN methods had a modest recovery of experimentally-derived interactions based on AUPR curve metrics, and that these methods tend to be comparable to one another.

Please note these SOTA cell-type GRNs are trained on normalized gene expression data (for mDCs, hPSCs, or YCs) and are output. These are the following SOTA cell-type GRNs that we obtain from (McCalla et al. 2023); the image on the left is from a main figure from (McCalla et al. 2023) and we highlight the SOTA GRNs that have output GRNs available for any (or all) of the 3 datasets. For these 3 datasets (from the respective studies), we utilize the GRN methods that were provided in the supplementary data for the given dataset. For instance, there was no Scribe model provided for YCs.

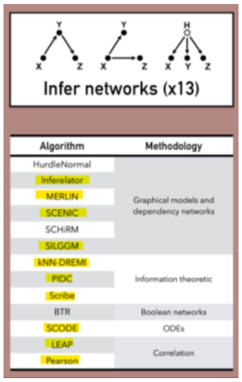

We note that Inferelator and MERLIN were developed for bulk RNA-seq datasets, while the other methods are specifically designed for applications in scRNA-seq datasets. Both methods leverage dependency network learning frameworks with different focuses: MERLIN integrates modularity, while Inferelator emphasizes regularized regression with stability selection to ensure robustness in the inferred network.

Summary:
That is, we utilize input (normalized gene expression data), validation (ground truth GRN for evaluating accuracy of the inferred GRNs), and output (predicted state-of-the-art (SOTA) cell-type GRNs) data provided by a previous study (McCalla et al. 2023) that benchmarked the performance of these SOTA GRN inference tools in mouse dendritic cells (mDCs), human pluripotent stem cells (hPSCs), budding yeast cells (YCs): Inferelator (Greenfield et. al 2013), knnDREMI (van Dijk et. al 2018), LEAP (Specht and Li 2017) mean, MERLIN (Roy et al. 2013), Pearson mean (baseline network: undirected fully connected network, where edges are weighted by correlation between each pair of genes over all cells), PIDC (Chan et al. 2017) mean, SCENIC (Aibar et. al 2017), SCODE (Matsumoto et al. 2017) mean, Scribe (Qiu et al. 2020), SILGGM (Zhang et al. 2018) mean. These SOTA GRN inference models utilize the following methods for network inference from scRNA-seq data: graphical models and dependency networks (Inferelator, MERLIN, SCENIC, SILGGM), information theoretic (kNN-DREMI, PIDC, Scribe), ordinary differential equations (SCODE), correlation (LEAP, Pearson) (McCalla et al. 2023).

§18.5.1: Graphical models and dependency network

- Inferelator (Greenfield et. al 2013)

Inferelator is also built on a dependency network learning framework but focuses on using regularized regression techniques for inferring networks. In this case, the **BBSR (Bayesian Best Subset Regression)** algorithm was employed within Inferelator. The method's internal stability selection was utilized to determine edge confidence in the inferred regulatory network.

- MERLIN (Roy et al. 2013)

MERLIN uses a dependency network learning framework combined with a probabilistic graphical model to incorporate a modularity prior into the network inference. It emphasizes modular structures within gene regulatory networks. In this method, stability selection was applied, and default settings were used for parameters related to sparsity ($p = -5$), modularity ($r = -5$), and clustering ($h = 0.6$) to guide the inference process.

- SCENIC (Aibar et. al 2017)

SCENIC (Single-cell Regulatory Network Inference and Clustering) builds on the GENIE3 algorithm, which infers regulatory networks using ensemble regression trees. SCENIC adds two additional steps: (1) **RcisTarget**, which identifies enriched regulatory motifs in target genes, and (2) **AUCell**, which calculates the activity of transcription factor (TF) target genes across individual cells. For certain applications, only the GENIE3 network outputs are used.

- SILGGM (Zhang et al. 2018)

SILGGM is based on a Gaussian graphical model (GGM) representation of gene regulatory networks. It estimates conditional dependencies between genes using methods like nodewise scaled Lasso and de-sparsified graphical Lasso. SILGGM includes a false discovery rate (FDR) control and was applied using the de-sparsified nodewise scaled Lasso approach in this case.

§18.5.2: Information theory

- knnDREMI (van Dijk et. al 2018)

kNN-DREMI is an adaptation of the DREMI algorithm for recovering gene-gene relationships from scRNA-seq data. DREMI originally estimates mutual information between pairs of genes using a heat-diffusion-based kernel density estimator. kNN-DREMI replaces this heat-diffusion kernel with a k-nearest neighbor (kNN) graph-based density estimator, which is better suited for handling the high dimensionality and sparsity of single-cell RNA-sequencing (scRNA-seq) data.

- PIDC (Chan et al. 2017)

PIDC (Partial Information Decomposition and Context) is based on an information-theoretic framework. It analyzes the relationships between three random variables (genes) by calculating the "unique," "synergistic," and "redundant" information contributions. It then infers gene regulatory networks by using the ratio of unique information to mutual information, establishing edges between genes based on this ratio.

- Scribe (Qiu et al. 2020)

Scribe infers gene regulatory relationships using an information-theoretic measure called Restricted Directed Information. It accounts for pseudotime and measures mutual information between a gene's current state and a regulator's previous state, conditioned on the target gene's previous state. Scribe uses bias correction to emphasize transitioning cells, enhancing the detection of regulatory interactions.

§18.5.3: Ordinary differential equations (ODEs)

- SCODE (Matsumoto et al. 2017))

SCODE formulates gene regulatory network inference as an ordinary differential equation (ODE) problem. It requires pseudotime and gene expression data. SCODE estimates the network by deriving a lower-dimensional representation of key expression dynamics and uses a linear transformation matrix to estimate gene expressions. The network is inferred by solving linear regressions based on this model.

§18.5.4: Correlation *r*

- LEAP (Specht and Li 2017)

LEAP (Learning Embeddings Along Pseudotime) assumes cells are ordered along a pseudotime trajectory and computes pairwise gene correlations at various time lags. The method selects a window size and computes Pearson correlation coefficients for gene expression across different time points and lags. The regulatory score between genes is the maximum of these correlation coefficients.

- Pearson

Pearson networks serve as a baseline for comparison. They represent a fully connected, undirected network where the edges between genes are weighted by the Pearson correlation coefficients calculated across all cells.

§18.6: scNET (application of the scGRNom tool)

Please note that scNET (Gupta et al. 2022) applied the scGRNom tool (Jin et al. 2021) on the same processed gene expression data used in Application 12 (Alzheimer's disease versus controls) for 4 of the 8 cell-types: Inhibitory Neurons, Excitatory Neurons, Microglia, and Oligodendrocytes. This tool also integrates multiomics data (e.g. open chromatin regions, TF motif binding data) to define a prior knowledge gene regulatory network (GRN) of candidate TFs for each TG. However, scGRNom instead uses an ElasticNet objective function and it does not utilize prior knowledge on TF-TF interactions (i.e. it does not utilize a prior protein-protein interaction (PPI) network). Instead, NetREm utilizes a PPIN-aware ElasticNet approach and also infers TF-TF coordination for TG regulation. Further, the steps we utilize to define candidate TFs for the TG from multiomics data (explained in §7 are more wholesome and adapt upon approaches used by tools like scGRNom). Here, we compare the performance of scNET with NetREm in AD versus Control cell-types for Inhibitory Neurons, Excitatory Neurons, Microglia, and Oligodendrocytes.

§18.7: Overview of different types of models utilized

Please note that we will elaborate on the details for obtaining these comparative methods in section: §#.#.
For now, please see this table below with more details.

		Simulated data	Simulated data	Real data (benchmarking)	Real data	Real data	Real data	Real data	Real data	Real data
		Simulation Study	hESCs	mESCs	HSCs	mDCs	hPSCs	bYCs	mSCs and nmSCs	AD vs Control for 8 cell-types
Baseline Regression Model	Linear Regression	✓	✓	✓	✓	✓	✓	✓	✓	✓
	LassoCV	✓	✓	✓	✓	✓	✓	✓	✓	✓
	ElasticNetCV	✓	✓	✓	✓	✓	✓	✓	✓	✓
	RidgeCV	✓	✓	✓	✓	✓	✓	✓	✓	✓
State-of-the-art cell-type GRN inference tools	RTN (used by RTNduals)	✓	✓		✓	✓	✓	✓		
	GRNboost2		✓	✓	✓	✓	✓	✓		
	Pearson					✓	✓	✓		
	Pearson (mean)					✓	✓	✓		
	PIDC mean					✓	✓	✓		
	MERLIN					✓	✓	✓		
	SCENIC					✓	✓	✓	✓	
	Inferelator					✓	✓	✓		
	knnDREMI					✓	✓	✓		
	LEAP mean					✓	✓	✓		
	SCODE mean					✓	✓	✓		
	SILGGM mean					✓	✓	✓		
	Scribe mean					✓	✓			
multiomics	scNET									✓

Section §19: RTNduals: Comparative Method for TF-TF Coordination

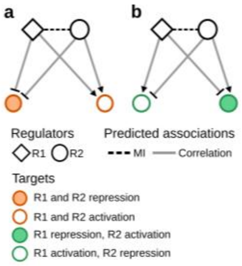

Please note that the image on the left is from RTNduals (Chagas et al. 2019), which performs an analysis of co-regulation and inference of dual regulons from gene expression data. It is a tool that is derived from the RTN (Reconstruction of Transcriptional Networks) package. Regulons include TFs, miRNA, mRNA.

We evaluate and compare the potential reliability and accuracy of predicted TF-TF interactions by NetREm in the context of other tools, such as RTNduals. Like NetREm, RTNduals reconstructs Transcription Networks (via its parent package RTN(Groeneveld et al. 2023) that infers a TF-TG regulatory network) and assesses whether regulons (i.e. TFs) might cooperate or compete within the regulatory frameworks to form possible co-regulatory loops (i.e. forming dual regulons). RTNduals analyzes co-regulatory associations (i.e. loops) among regulons, uses the Data Processing Inequality (DPI) algorithm (Meyer et. al 2008) to remove the weakest interaction between 2 regulators and a common target gene (TG), and ultimately RTNduals infers statistically significant dual regulons from input gene expression data and a list of potential TFs. Both tools (RTNduals and NetREm) recognize that each TG in each TF-TG regulatory network may be linked to multiple TF regulators based on direct and/or indirect interactions among TFs.

Please note this following analysis is performed for each of the 13 NetREm-based models that use input PPI networks. (And the same is applied for baseREm, which is basically the NetREm pipeline with virtually no prior PPI knowledge).

§19.1: Comparative Analysis Approach

We intend to discern whether integrating a PPI network as a prior knowledge network constraint enhances NetREm's predictive accuracy for TF-TF links (direct and/or indirect) over RTNduals, using identical input gene expression data and TFs list. We assume that this PPI ground truth network is static across various cell types and conditions, eliminating the need for users to curate additional PPI data, unless desired. For a balanced comparison, both RTNduals and NetREm are run on the same input training gene expression data and a fixed list of TFs. We then compare their predicted TF-TF links, focusing on the same top number of interactions.

In the table below, we see the top k TF-TF links that are yielded by RTNduals across various applications. This number of links it returns (if at all) are based on its own methodology and mathematical analyses of significant overlap in the list of regulons (i.e. the set of predicted target genes for a given TF regulator). We also note the # of TFs that are input to the NetREm model and to RTNduals as candidate TF predictors for the TG: $N = \mathcal{N}$. (In NetREm, if the given TG is also a candidate TF, we remove that TF from the list of candidate TFs for the TG: $N = \mathcal{N} - 1$).

	**# of k Top TF-TF Links Returned by RTNduals for various single-cell RNA-seq dataset applications**				
Count: # of links returned $\boldsymbol{k}$	**# of TFs in input TF list: $\mathcal{N}$**	**Cell type**	**Source**	**More information**	organism
2,587	93	Astrocytes		Control Astrocytes	Human
2,335	82	Oligodendrocytes		Control Oligodendrocytes	Human
1,047	78	Inhibitory Neuron		Control Inhibitory Neurons	Human
1,190	77	Excitatory Neuron		Control Excitatory Neurons	Human
764	98	Pericytes		Control Pericytes	Human
665	93	EndoBBB		Control Endothelial Blood Brain Barrier (BBB) Cells	Human
2,041	90	OPCs		Control Oligodendrocyte Precursor Cells (OPCs)	Human
425	97	Microglia	Mathys et. al	Control Microglia	Human
267	88	Pooled Schwann cells	GTEx	Pooled Schwann cells across 5 tissues	Human
1,514	80	Oligodendrocytes		Oligodendrocytes	Human
990	76	Inhibitory Neuron		Inhibitory Neurons	Human
1,314	76	Excitatory Neuron		Excitatory Neurons	Human
1,267	92	Microglia	Lake et. al	Microglia	Human
4,076	280	YCs	Jackson et. al	Budding Yeast Cells (YCs)	Yeast
0 ☹	178	HSCs	Buenrostro et. al	Hematopoietic Stem Cells	Human
0 ☹	1,029	Each of the 9 different PBMC sub-types		Naïve CD4 T cells, CD14 Monocytes, Memory CD4 T cells, B cells, CD8 T cells, FCGR3A Monocytes, Natural Killer cells, Dendritic cells, and Platelet cells	Human
81	170	hPSCs	Han et. al	Human Pluripotent Stem Cells	Human

133	93	mDCs	Shalek et. al	Mouse Dendritic cells	Mouse

§19.2: RTNduals method

We provide gene expression data (genes and samples) and a list of TFs into RTNduals. We run the method with defaults. That is, we use 5% adjusted p-value, 1,000 permutations, 100 bootstraps, epsilon eps = NA: estimate the threshold from the empirical null distribution computed in the permutation and bootstrap steps to compute the DPI-filtered regulatory network). Please note more details on the process: https://www.bioconductor.org/packages/release/bioc/vignettes/RTNduals/inst/doc/RTNduals.html.

We run the RTN package to output a predicted TF-TG regulatory network solely using gene expression data (`gexp`) and a list of TFs (`tf_list`). RTNduals does not incorporate any TG-specific inputs or any additional prior knowledge.

- `rtni <- tni.constructor(gexp, regulatoryElements = tf_list)`
- `rtni <- tni.permutation(rtni, nPermutations=1000, pValueCutoff=0.05)`
- `rtni <- tni.bootstrap(rtni, nBootstrap=100)`

This outputs a regulatory network based on the RTN package. This regulatory network is inferred by mutual information analysis followed by multiple hypothesis testing corrections. Then, it computes a DPI-filtered regulatory network where DPI is the Data Processing Inequality (DPI) algorithm. We set the epsilon = NA in order to estimate the threshold from the empirical null distribution that was found in the permutation and bootstrap steps. To do this, we run the tni2mbrPreprocess function to construct an mbr object and apply the DPI algorithm.

- `rmbr <- tni2mbrPreprocess(rtni)`

Next, RTNduals tests the associations for dual regulons.

- `rmbr <- mbrAssociation(rmbr)`

This process involves examining regulons (i.e., TFs) and their associated regulatory elements. It identifies inferred regulatory connections and maps network triplets involving regulons, evaluating the overlap between regulons and analyzing pairwise correlations. The mbrAssociation method takes the transcriptional network (i.e TF-TG regulatory network) constructed in previous steps and identifies all triplets consisting of two regulators and a shared target gene (TG). It calculates the mutual information between the regulators and evaluates the consistency of predicted downstream effects using correlation analysis. A Fisher's exact test is applied to determine if the number of shared TGs is significantly greater than would occur by chance. It compares shared targets in order to infer 'dual regulons', testing whether regulators can co-operate or compete in influencing TGs. Please note that we run the following to yield results:

- `overlap <- mbrGet(rmbr, what="dualsOverlap")`
- `correlation <- mbrGet(rmbr, what="dualsCorrelation")`

§19.3: RTNduals may fail to yield results

There are applications where RTNduals may fail to predict any results. This is even if the inferred GRN (based on the RTN package) makes strong predictions. = For instance, the table above shows how RTNduals fails to yield any TF-TF links for applications such as HSCs and for any of the 9 PBMC sub-types (i.e. the # of links it returns is 0!); in fact, it returns an error when the mbrAssociation() function is run to check regulons and regulatory elements, where it states: "Error in mbrAssociation(rmbr): NOTE: no partial regulon has minimum ≥ 15". Further, RTNduals is unable to yield isolated predictions of TF-TF coordination for regulating individual TGs (i.e. it cannot predict TG-specific TF-TF coordination B). For instance, for HSCs, we get the following error:

```
> rmbr <- mbrAssociation(rmbr)
-Checking regulons and regulatory elements...
Error in mbrAssociation(rmbr) :
  NOTE: no partial regulon has minimum >= 15
> mbrGet(rmbr, what="summary")
$MBR
$MBR$Duals
      Tested Predicted
Duals     NA        NA
```

Please note that this is a weakness of the RTNduals method. Thus, we will focus on the situations where RTNduals returns predicted TF-TF links.

§19.4: Applying NetREm and RTNduals for each context

For each of our NetREm models in a given context:

We run NetREm for the Target Genes (TGs) in the cell-type for the fixed # of candidate TFs $N = \mathcal{N}$; if the TG is also a TF, we just remove that TF from the list of candidate TFs for that TG, so that TG has $N = \mathcal{N} - 1$ candidate TFs (**Table S1**). That is, we do not use any other prior GRN knowledge.

If the model is 1 of the 13 NetREm$_{\beta=1}$ models, we fit NetREm with $\beta = 1$, no y-intercept, and use LassoCV to optimize α using 5-fold Cross Validation (CV) to minimize the CV Mean Square Error. Otherwise, if this is the baseREm model, we run NetREm with $\beta \approx 0 > 0$; that is, $\beta \approx 1e - 100$.

In any case, we note that NetREm may incorporate TG-specific knowledge, which RTNduals may be unable to do. Nonetheless, we want to strictly compare the performance of RTNduals with that of NetREm in the absence of additional TG-specific knowledge for the context. Thus, we use the same TFs for each TG for the sake of comparison with RTNduals. We also note a limitation of RTNduals is that it may not return any significant results for some of the single-cell expression data; RTNduals is not adept at single-cell data, unlike NetREm that can handle both bulk and single-cell expression (sparse!) data.

For each TG, we learn the TG-specific TF-TF coordination network B. Then, we aggregate the results across all TGs to learn our final $\bar{B}$ matrix of cell-type coordination scores. Again, please note that that we are using the same TFs for each TG for the sake of comparison with RTNduals. We note our $\bar{B}$ matrix always returns more predicted TF-TF links than RTNduals does, which could be up to: $(\mathcal{N})(\mathcal{N} - 1)(0.5)$ for NetREm. We run NetREm using the given input PPI network.

Overall, NetREm can input TG-specific information (e.g. prior GRN knowledge on potential transcriptional regulators, like TFs, for the TG: TG-specific candidate TFs) and prior information on interactions among TF predictors; ultimately, NetREm can also output TG-specific TF-TF coordination links, which RTNduals cannot do.

Our goal is to determine if RTNduals can pick up on true TF-TF relations with PPI support (based on an Evaluation PPIN) and compare results against NetREm, which incorporates various types of input PPINs (i.e. 13 different types of input PPINs, with their own strengths and weaknesses).

Evaluation and Benchmarking:

We assess the number k and quality of significant TF-TF links predicted by RTNduals and subsequently align our $|\bar{B}|$ matrix (magnitude of cell-type TF-TF coordination scores, where higher values in $|\bar{B}|$ denote stronger antagonism and/or cooperativity among TFs). Thus, we include the top k TF-TF links (based on TF-TF coordination scores for $|\bar{B}|$) for a harmonized comparison. The total number and respective percentages of TF-TF links, annotated based on an Evaluation PPIN, are compared between RTNduals and NetREm. This comparison enables us to critically assess and benchmark the efficacy of NetREm's predicted TF-TF coordination network against RTNduals, based on identical input data. This comparative analysis is poised to contribute valuable insights into the reliability of the top TF-TF coordination links predicted by NetREm.

Section §20: TF-TG Regulatory Network Evaluation using Gold Standard Networks

§20.1: Confusion matrix:

All TF-TG regulatory network predictions can be classified into these 4 categories, which form a confusion matrix.

- *True Positives (TP):* the # of instances where the model correctly predicts the positive class. In other words, it is the # of correct predictions that an instance is positive.

- *False Positives (FP):* the # of instances where the model incorrectly predicts the positive class. That is, it is the # of times the model predicts an instance as positive when it is actually negative.
- *False Negatives (FN):* the # of instances where the model incorrectly predicts the negative class. It means the # of times the model predicts an instance as negative when it is actually positive.
- *True Negatives (TN):* the # of instances where the model correctly predicts the negative class. It is the # of correct predictions that an instance is negative.

TF-TG regulatory links (and/or TF-sign-TG regulatory links) that are not predicted by a model are predicted negatives (as they are missing links in the model's inferred TF-TG regulatory network). These missing links may be FNs (if they are true links in the ground truth network that should have been predicted) or TNs (not found in the ground truth network and correctly not predicted as a meaningful link by the model). Regulatory links are predicted by a model are predicted positive (and may comprise TPs if they are correct links found in the ground truth network or FPs if they are predicted links that are actually not found in the ground truth network). To calculate the total # of TF-TG regulatory links that are theoretically possible for each scenario, we utilize the following formula. Total # of predictions = Total TF-TG regulatory links possible = TPs + FPs + TNs + FNs = $\left((Total\ \#\ of\ TFs) \times (Total\ \#\ of\ TGs)\right) - (\#\ of\ TFs\ that\ are\ TGs)$

Then, for instance, in hESCs (1,250 TGs and 207 TFs, of which 15 TFs are also TGs), we have: (207)(1,250) – 15 = 258,735 potential TF-TG regulatory links that can be predicted. Thus, TP + FP + FN + TN = 258,735. Likewise, in mESCs (19,225 TGs and 195 TFs, of which 194 TFs are also TGs), we have 3,748,681 potential TF-TG regulatory links that can be predicted. Further, in mDCs (9,087 TGs and 93 TFs, where all TFs are also TGs), we have 844,998 potential TF-TG regulatory links that can be predicted. TFs that are not TGs in the gene expression data sets may be considered potential master regulators. Similarly in HSCs (10,588 TGs and 178 TFs, of which 177 TFs are also TGs and 1 TF is a master regulator), we have: (178)(10,588) – (177) = 1,884,664 – 177 = 1,884,487 potential TF-TG regulatory links that are possible in the universe.

§20.2: Evaluation metrics:

There are various metrics we can use to evaluate the model predictions of TF-sign-TG regulatory links (in hESCs) and TF-TG regulatory links (in hESCs, HSCs, mDCs, YCs, hPSCs, and mESCs).

- Precision measures the proportion of predicted positives (True Positives (TPs) + False Positives (FPs)) that are correct (i.e. TPs) out of all of the regulatory links, how many have been validated. Precision = $\frac{TP}{TP + FP} = \frac{TP}{Total\ Predicted\ Positives}$ = True Positive Rate (TPR)
- Specificity measures the proportion of actual negatives (True Negatives (TNs) + False Positives (FPs)) that are correctly identified. Specificity = $\frac{TN}{FP + TN} = \frac{TN}{Total\ Actual\ Negatives}$
- Recall measures the proportion of actual positives (TPs + FNs) that are correctly identified. That is, it is the # of correct positive predictions (i.e. TPs) divided by all the relevant samples (i.e. all samples that are truly positive). Recall = $\frac{TP}{TP + FN} = \frac{TP}{Total\ Actual\ Positives}$ = Sensitivity
- F1 Score is the harmonic mean of precision and recall. $F1 = (2)\left(\frac{(\text{precision}) \times (\text{recall})}{(\text{precision}) + (\text{recall})}\right)$
- Balanced Accuracy (BACC) is great for skewed datasets. $BACC = \frac{(\text{sensitivity}) + (\text{specificity})}{2}$
- Total correct predictions = TPs + TNs. Total errors = FPs + FNs
- Overall accuracy is the ratio of total correct predictions over the total number of predictions. And the total # of predictions is the total # of possible regulatory links. $Overall\ Accuracy = \frac{TP+TN}{TP+TN+FP+FN}$
- Perint is the percentage of intersection and it is given as: $Perint = \frac{TP}{TP+FP+FN}$

§20.3: Area Under the Receiver Operating Characteristic Curve (AUC) and the Area Under the Precision-Recall Curve (AUPR) when Ground Truth Network has no weights:

Please note that this sub-section describes the approach for computing the AUC and AUPR metrics of different methods when the gold standard GRN is a binary, dummy network with no weights. That is, in this scenario, the gold standard GRN simply denotes the TF-TG regulatory edges and does not provide any weight or sign for the regulatory interactions.

To evaluate the accuracy of our transcription factor-target gene (TF-TG) regulatory network predictions, we used two primary metrics: the Area Under the Receiver Operating Characteristic Curve (AUC) and the Area Under the Precision-Recall Curve (AUPR). These metrics provide complementary perspectives on the model's performance, particularly in distinguishing true positive TF-TG regulatory relationships from false positives. We note that at random performance, the baseline AUPR is equal to the fraction of Positives. The baseline AUC from random guessing is 0.5.

Methodology Overview

1. **Data Preparation:**
    - We generated a dataset comprising predicted TF-TG regulatory interactions (i.e. GRN links), with associated weights from the model. These weights or confidence scores can be represented by the absolute value of the coefficients of the TFs (e.g. magnitudes of coefficients $|c^*|$ in the NetREm model), importances of the edges, and other metrics based on the model. That is, these scores quantify the predicted strength or likelihood of the regulatory relationship.
    - A separate ground truth dataset containing known TF-TG interactions was compiled, serving as the "gold standard" for evaluation.
    - We merged the predicted and ground truth datasets to construct an evaluation data frame that aligned predicted GRN links with their corresponding ground truth status. Each TF-TG pair was labeled as 1 if it appeared in the ground truth and 0 otherwise.
2. **Scaling of Prediction Scores:**
    - To facilitate a comparative analysis across different thresholds, the confidence scores from our model were scaled between 0 and 1 by dividing each score by the maximum score in the prediction set. This normalization step ensures that the prediction scores are on a consistent scale for evaluation.
3. **Calculation of AUC (Area Under the ROC Curve):**
    - We computed the Receiver Operating Characteristic (ROC) curve, which plots the True Positive Rate (TPR, also known as sensitivity or recall) against the False Positive Rate (FPR) at various threshold levels of the prediction score.
        - **TPR (Recall)**: The proportion of true TF-TG interactions correctly predicted by the model out of all true interactions.
        - **FPR**: The proportion of false TF-TG interactions (i.e., predicted links not present in the ground truth) out of all interactions that are not in the ground truth.
    - The ROC curve provides an overall view of the trade-off between sensitivity and specificity (1 - FPR) at different score thresholds.
    - The AUC is calculated as the integral of the ROC curve, providing a scalar value between 0 and 1 that summarizes the model's discriminative ability across all thresholds. A higher AUC indicates better model performance in distinguishing between true and false TF-TG interactions.
4. **Calculation of AUPR (Area Under the Precision-Recall Curve):**
    - We also computed the Precision-Recall (PR) curve, which is particularly useful for imbalanced datasets where true interactions are much fewer compared to false interactions.
        - **Precision**: The proportion of predicted TF-TG interactions that are correct (true positives out of all predicted positives).
        - **Recall**: The proportion of true interactions that are correctly predicted (equivalent to TPR).
    - The PR curve plots precision against recall at different threshold levels. Since it focuses on the performance of the model concerning the true positives, the PR curve is especially sensitive to the correct prediction of true regulatory relationships in cases of class imbalance.
    - The AUPR is the area under this curve, providing a single value that captures the model's precision-recall trade-off across all thresholds. A higher AUPR indicates a better balance between precision and recall, and thus better performance in identifying true positives while minimizing false positives.

5. **Threshold-independent Evaluation:**
   - Both AUC and AUPR are threshold-independent metrics, meaning they evaluate model performance over a range of possible thresholds. This characteristic is beneficial as it avoids potential biases associated with choosing a single threshold for classification, providing a holistic assessment of model accuracy.
6. **Interpretation of AUC and AUPR:**
   - **AUC (ROC Curve)** provides insight into the overall ability of the model to distinguish between true and false TF-TG links, offering a balanced perspective on both false positives and false negatives.
   - **AUPR (PR Curve)** focuses on the precision-recall trade-off, which is particularly informative for datasets with a high class imbalance, as it emphasizes the model's capacity to correctly identify true TF-TG interactions (true positives) with minimal false positives.

By utilizing both AUC and AUPR, we gain a comprehensive understanding of model performance across varying thresholds and contexts, ensuring robust evaluation of the predicted TF-TG regulatory network. These metrics together confirm the model's effectiveness in capturing biologically meaningful regulatory relationships.

§20.4: Calculating the % of Predictable TFs

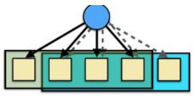

Please note that in this section, we explain how we calculate the % of predictable TFs. We derive this metric based on a previous study (McCalla et al. 2023) that implemented this metric. We identify predictable TFs through Target Overlap Analysis. Our approach is adapted from the approach described in this previous study and the image on the left is from that same study.

Overview

To assess the performance of TF regulatory network inference, we implement a granular approach focused on measuring the ability of predicted networks to recover true TF-target (TF-TG) relationships. The goal is to determine whether the predicted set of TGs for each TF shows a statistically significant overlap with a true target set, as defined by an experimentally validated gold standard. This approach evaluates the predictive accuracy of inferred networks at the level of individual TFs, providing insights into how well the model identifies true regulatory targets.

Data Preparation

The analysis requires two datasets: the predicted TF-TG network and a validation network containing experimentally confirmed TF-TG links. The predicted network includes TFs and their corresponding TGs as inferred by a computational method, while the validation network serves as the "ground truth," containing known TF-TG relationships from biological experiments. Both networks are combined to establish a comprehensive set of possible TGs, creating a "universe" of TGs for statistical testing.

Statistical Testing of Target Overlap

For each TF in the predicted network, we quantify the significance of overlap between its predicted TGs and the corresponding validated TGs. This is achieved using a hypergeometric test, which calculates the probability of observing a given number of overlapping TGs under the assumption of a random distribution. The hypergeometric test considers the total population of TGs, the number of true targets for each TF, the number of predicted targets, and the size of the overlap between these sets. The result is a p-value for each TF, representing the likelihood that the observed overlap occurred by chance. A lower p-value suggests that the overlap is unlikely to be due to random variation, indicating accurate prediction of the TF's targets. Given that multiple statistical tests are conducted across all TFs, there is a risk of inflating the type I error rate (false positives).

Multiple Hypothesis Correction

To address the issue of multiple comparisons, we apply the Benjamini-Hochberg procedure to control the false discovery rate (FDR) and correct for multiple hypothesis testing. This correction adjusts individual p-values, reducing the likelihood of incorrectly identifying a TF as predictable when the significant overlap is due to chance. A corrected p-value threshold (commonly 0.05) is used to classify a TF as "predictable," meaning the overlap between predicted and validated targets is statistically significant after accounting for multiple testing.

Quantifying Network Performance

The number of predictable TFs is then quantified to provide a performance measure for network inference. The percentage of predictable TFs out of the total number of TFs in the final model serves as an indicator of how well the inferred network captures true TF-TG relationships. A higher proportion of predictable TFs suggests greater accuracy in identifying true regulatory interactions.

This approach enables focused evaluation of network performance at the level of individual TFs, offering insights into the overall quality of the network inference and predictive power for specific regulatory factors. By measuring the statistical significance of target overlap and applying multiple hypothesis correction, the evaluation of predicted TF-TG interactions is rigorous. This method serves as a valuable metric for comparing various network inference tools and assessing their biological relevance in capturing transcriptional regulation, helping to identify areas of strength and potential improvement.

Section §21: Additional Biological Resources for TF-TG Regulatory Network Evaluation and Downstream Analysis

§21.1 Expression quantitative trait loci (eQTL)-based validation

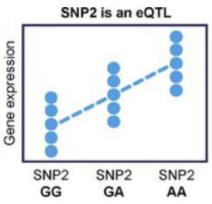

We use expression cell-type quantitative trait loci (eQTL) data, which links single nucleotide polymorphisms (SNPs) to changes in the expression of various TGs. Integrating eQTL data may help us verify some of the TF-Regulatory Element-TG links inferred by NetREm. For AD we incorporate additional eQTLs for 4 of our cell-types from Zeng et al. (Zeng et al. 2022); we did not use this source when defining eQTL-based regulatory regions. For Schwann Cells (SCs), we recognize that the Tibial Nerve eQTL dataset is limited in terms of its associations as it only has sequencing for 532 human samples, and studies observe that relatively small sample sizes for eQTL data may likely result in relatively low statistically significant SNP-TG associations(Ndungu et al. 2020); moreover, this data (i.e. left peripheral tibial nerve from the gastrocnemius region) is a proxy for SCs, given the current unavailability of a SC-specific cell-type eQTL dataset. Hence, for eQTL validation for SCs, we allowed weak effects (along with strong effects) for motifbreakR (Coetzee et al. 2015) predictions of SNP-TF links. For AD applications, we mandate that SNP-TF links ought to have strong effects.

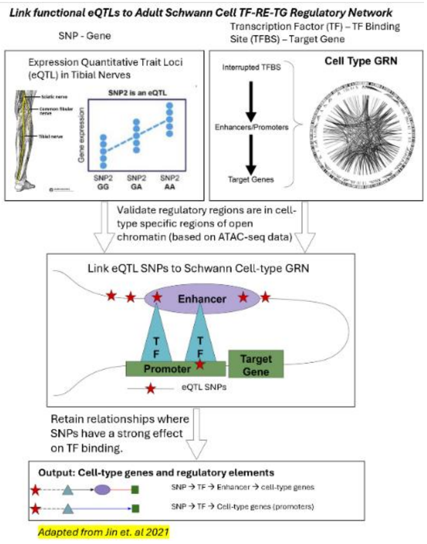

To understand the relationship between SNPs, TFs, and TGs, we employ a computational pipeline that begins with NetREm for the initial inference of TF-Regulatory Element (RE)-TG links. That is, for our TGs, we may overlay our final cell-type TF-TG regulatory networks (predicted by NetREm) with the TF-RE-TG links (based on the prior GRNs); this is since the prior GRNs do initial feature selection to identify N candidate TFs for the given TG out of the $\mathcal{N}$ potential cell-type TFs overall (where some of these N candidate TFs have TF-RE-TG links based on the motif-based GRN and other candidate TFs are augmented with indirect association with these TFs). Then, NetREm selects $N^* \leq N$ optimal TFs with $c^* \neq 0$ for the TG; thus we may see which of these N^* TFs has TF-RE-TG links for the TG and use that information to retrieve our TF-RE-TG links (for other TFs that lack strong binding support, we tend to assume they may have more indirect binding mechanisms and do not focus on those). To validate these inferred relationships, we use motifbreakR tool designed to predict the impact of SNPs on TF binding to their binding sites (TFBSs). This allows us to assess whether SNPs within the REs can explain the observed cell-type-specific eQTL data. Specifically, we examine SNPs that not only fall within the regulatory elements of TGs but also affect TF binding and correlate with changes in TG expression. Please note that the image on the right is adapted from (Jin et al. 2021).

SNPs mapped on original TF-TG regulatory networks

Nonetheless, in the absence of prior reference GRNs for the TGs, we may still use NetREm's final TF-TG regulatory networks where for a given TF-TG regulatory link, we can see if: the eSNP-TF exists (based on motifbreakR) and the eSNP-eTG link exists (based on expression quantitative trait loci (eQTL) eSNP-eTG links). This may yield more results (than overlaying with TF-RE-TG links can).

§21.2 GWAS-eQTL colocalization analysis

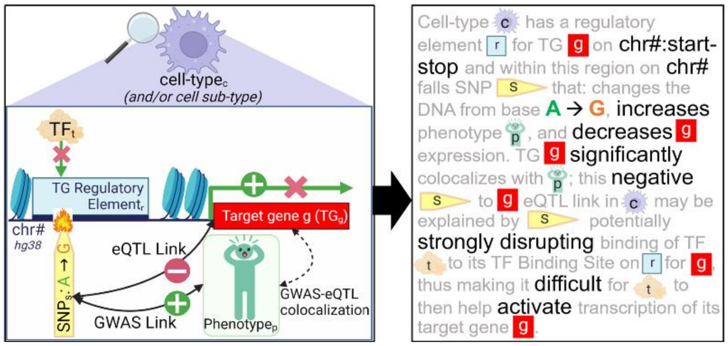

We incorporate Genome-Wide Association Studies (GWAS) to further enrich our analysis. GWAS data associates SNPs and various traits, including diseases. In this way, we may predict pathways from non-coding SNPs to disease phenotypes, via TFs with altered TF binding to TFBSs, impacting TF interactions with each other and ultimately cascading to changes in TG expression. We specifically investigate SNPs that are part of our eQTL-based validation and associated with GWAS traits. To assess the statistical significance of links that influence both TG expression and GWAS traits, we perform GWAS-eQTL colocalization analyses using the coloc (Wallace 2020) tool for a given shared eQTL and GWAS SNP. We set posterior probability (PP4) threshold > 50% for statistical significance. Here, PP4 is the probability that the given SNP is associated and affects both traits: GWAS trait and TG expression trait (i.e. eQTL) (Giambartolomei et. al, 2014). We want to assess whether any statistically significant links exist that influence both TG expression and the given GWAS trait. Specifically, we consider PP4 ≥ 70% as a threshold for statistical significance.

Adding Linkage Disequilibrium (LD) Analysis

To deepen our understanding of SNP co-inheritance patterns within the population (and their non-random association with each other), we conduct Linkage Disequilibrium (LD) analyses via LDlink (Machiela et. al 2015). This helps us correlate a pair of SNPs (on the same chromosome). That is, SNPs that are in LD are significantly correlated with each other.

§21.3 Gene Enrichment Analysis

We use Gene Enrichment Analysis tools (e.g. MaayanLab (Rouillard et al. 2016), clusterProfiler (Wu et al. 2021), BaderLab (https://baderlab.org/), Metascape (Zhou et al. 2019), and g:Profiler (Reimand et al. 2007) with $-\log_{10}(q)$ to validate biological relevance of our terms for our input lists of TGs (where q refers to a p-value (p) adjusted for the False Discovery Rate due to multiple hypothesis testing).

Section §22: Myelinating vs. Non-myelinating Human Schwann cells (Application 10): Additional details

§22.1 Experimental validation data for Schwann cells (SCs)

We gathered as well as performed experimental analysis for TFs that play key roles in Schwann cells, such as: EGR2, TEAD1, SOX10, RXRG, SREBF1, STAT1, YY1, and NR2F2. Thus, we primarily analyze NetREm's predicted TF-TG regulatory links and TF-TF interactions with respect to these TFs. SOX10 has been shown to play key roles in mSCs and nmSCs (Balakrishnan et al. 2021) and EGR2 (may serve as either an activator or repressor of TG expression [18]) is important for mSCs (Svaren and Meijer 2008). We used biomaRt(Durinck et al. 2009) and g:Profiler(Reimand et al. 2007) to map rat/mouse gene names to the orthologous gene names in humans. We consider *loss-of-function (LOF) TGs* as those that are impacted directly by the TF being knocked out. Then, we consider validated direct TGs as *LOF TGs* that have a ChIP-seq binding peak for that respective TF that is within 100 kilobases on the DNA; thus, *validated direct TGs* not only have knockout support (TG expression is associated with the presence or absence of that TF) but also strong evidence of TF binding nearby. Hence, we are more confident about the *validated direct TGs* but some of the *LOF TGs* could be indirectly regulated by a given TF. This additional data serves to both validate our predicted TF-TG regulatory links and to assess the strength of potential TF-TF interactions.

Target Genes (TGs) for core SC TFs Predicted by TF-TG regulatory links	Validation Data for Schwann Cells (SCs)	
	Mice/Rat Knockout	***Rodent ChIP-seq binding peak***
	TG expression affected by knockout of TF	TG has peak for that TF within 100 kilobases on DNA
**Loss-of-Function TGs**	✓	**?**

**Direct TGs (Verified)**	✓	✓
**Other Candidate TGs**	**?**	**?**

Data utilized:

We have ChIP-seq (Chromatin Immunoprecipitation Sequencing) binding data from sciatic nerve of P15 rats (rn5 genome) for the following TFs: EGR2, SOX10 (Lopez-Anido et al. 2015; Srinivasan et al. 2012), and SREBF1 (manuscript in preparation). EGR2 and SOX10 are expressed exclusively in Schwann cells of peripheral nerve, and ChIP-seq raw data have been deposited in NCBI (Lopez-Anido et. al 2015). We obtain ChIP-seq data for STAT1 in differentiating rat SCs from NCBI GSE211337 for the rn6 rat genome assembly (Xu et al. 2023). For analysis of binding to known enhancers, we have employed H3K27ac ChIP-seq data from rat peripheral nerve (Lopez-Anido et al. 2015; Hung et al. 2015). We utilize ChIPseeker (Yu et al. 2015) to annotate peaks with TF binding to the nearest gene, intergenic region, promoter, Transcription Start Site (TSS), Transposable Elements (TEs), exon, or intron. We also use rtracklayer (Lawrence et al. 2009) and the rn5tohg38.over.chain (Karolchik et al. 2009) file from UCSC (Karolchik et al. 2009) to perform liftover (2021) from rn5 to hg38 genomic coordinates for the ChIP-seq tracks (we similarly use rn6torn5.over.chain to map STAT1 TF ChIP-seq files to rn5, which may explain its relatively lower overlaps). For YY1, RXRG, NR2F2, TEAD1, we perform Cut&Run (Cleavage Under Targets & Release Using Nuclease) assays in the rat S16 Schwann cell (SC) line (GSE247955).

For loss-of-function (LOF) data, we have obtained expression profiling data for sciatic nerve of knockout mice for EGR2(Le et. al 2005), and Schwann cell-specific knockout of YY1 (He et. al, 2010). For TEAD1, we employ RNA-seq data from analysis of a Schwann cell-specific knockout of YAP/TAZ coactivators of TEAD TFs (Poitelon et. al 2016). For SOX10, we use RNA-seq data from a derivative of the S16 Schwann cell line in which CRISPR gene editing is used to eliminate the *Sox10* gene(Fogarty et. al 2020). In this knockdown data, the TF is downregulated to determine which genes had significant changes in their expression. We retrieve additional lists of knockdown data for SOX10 and EGR2 from (Srinivasan et al. 2012) and LOF data for SREBF1 from (Cermenati et al. 2015). We utilize various resources, such as Ensembl biomaRt(Durinck et al. 2009), g:Profiler(Reimand et al. 2007), and HGNC multi-symbol checker(Oh et al. 2022) to adjust gene names from mouse/rat to human names and retrieve updated gene names. Fold changes (FCs) < 0 imply that the TF may activate the TG, while FC > 0 may suggest that the TF represses the TG. For STAT1, we obtain data on comparative gene expression profiling analysis of RNA-seq data for differentiating STAT1 knockdown SCs and control SCs from NCBI GSE211336 (Xu et al. 2023). We consider LOF genes for the TF as those where $|\log_2(\mathrm{FC})| > 0.5$ and the adjusted p-value < 0.05. Unfortunately, LOF data is missing for RXRG. LOF genes with nearby annotated ChIP-seq peaks for the TF are defined as validated direct TGs.

We validate novel SOX10 predicted TGs using SOX10 ChIP-seq (Lopez-Anido et. al 2015) epigenomic data of rats in the Peripheral Nervous System (PNS), including: SOX10 PNS Peaks, SOX10 Sciatic Nerve (SN) reads: Run 204, SOX10 PNS Filtered DBChIP Peaks (found a SOX10 site differentially expressed in Schwann cells), Homer Filtered PNS K27ac, and H3K27Ac (marker for active enhancers (Zhang et al. 2020)) Immunoprecipitation Sorted Tag Directory; we have a visual session in UCSC genome browser available (Karolchik et al. 2009). Peaks files show called reads.

Cut&Run Data Processing:

Cut&Run FastQ files are mapped to rat reference genome rn5 using Bowtie2 (Langmead et. al 2012; Langmead et al. 2009) to produce Binary Alignment Map (BAM) files. BAM files are filtered for mapped reads using BamTools(Quinlan and Hall 2010) and sorted into called peaks using MACS2(Zhang et al. 2008; Liu 2014). BedTools bamCoverage generates bedgraphs of Cut&Run and ChIP-seq samples. Bedgraph files show the distribution of reads. Heat maps are created via EAseq(Lerdrup et. al 2016). Data processing is performed in a cloud-based manner through GalaxyBiostars(Afgan et al. 2018). Cut&Run and ChIP-seq tracks are visualized using the University of California, Santa Cruz Genome Browser(Kent et al. 2002).

Co-binding / colocalization analysis among pairwise TFs:

We perform co-binding analyses among all pairwise TFs for the 7 TFs above based on the rn5 rat ChIP-seq tracks for the TFs. For each $TF_i - TF_j$ TF-TF coordination pair, we determine the percentage of ChIP-seq peaks for TF_i that have

binding overlaps with any given peaks for TF_j based on 15,864 H3K27ac peaks (histone epigenetic mark of active enhancer regions) shared between the PNS and S16 tracks. Similarly, the $TF_j - TF_i$ TF-TF coordination pair, determines the % of ChIP-seq peaks for TF_j that have binding overlaps with any given peaks for TF_i based on those shared H3K27ac peaks. We note that the value for $TF_i - TF_j$ may differ from that for $TF_j - TF_i$ based on the denominator.

In addition, we determine the Jaccard similarity among the TFs based on TF-TG regulatory network links for the TGs. We identify common TGs. Further, we overlay the regulatory regions in our TF-TG regulatory network (in hg38 human genomic coordinates) with the ChIP-seq peaks (after liftover from rat rn5 to hg38 coordinates) and determine TF-TG regulatory links for our core SC TFs that bind to regulatory regions with ChIP-seq support. We overlap the resulting TGs among TFs. In addition, we overlap the rn5 ChIP-seq peaks with the rn5 H3K27ac enhancers to determine enhancer-based ChIP-seq peaks and overlay those with our TF-TG regulatory networks to uncover how many shared predicted TGs have support based on ChIP-seq data for TFs in regions of active enhancers in rats. If there is a relatively large overlap in the fraction of peaks between 2 different proteins from 2 respective ChIP-seq experiments, it may suggest that both proteins are cooperative in their gene regulatory behavior (Yu et al. 2015).

Ground truth gene regulatory network (GRN) in Schwann cells:

We note the following pooled results for SCs based on 17,049 TGs that are also found in the Dorsal Root Ganglion (DRG) SC gene expression data. This corresponds to 10,790 total unique TGs (including 4,708 unique TGs that are considered direct for at least 1 TF; nonetheless, they may be LOF TGs and/or direct TGs for other TFs). RXRG is missing from the analysis since we did not have any LOF data for RXRG. This original GRN has 16,804 total TF-TG links, of which 6,393 links are TF-TG for validated direct TGs. In this original GRN, 75,523 links would have been possible among the 7 TFs and all LOF TGs (pooled LOF only TGs and validated direct TGs) and 32,950 links are possible among the 7 TFs and all validated direct TGs.

**Ground Truth Gene Regulatory Network in Schwann Cells** (Based on 17,049 Target Genes (TGs) found in Dorsal Root Ganglion (DRG) Schwann Cell Gene Expression Data) 16,804 total TF-TG links			
Transcription Factor (TF)	# LOF Only TGs	# Direct TGs	All LOF TGS<br>**Total**
SOX10	5,404	2,278	7,682
YY1	2,838	1,273	4,111
STAT1	216	786	1,002
TEAD1	663	735	1,398
NR2F2	250	719	969
SREBF1	133	387	520
EGR2	907	215	1,122

Based on our input multi-omics analysis, we uncover results for 13,886 TGs in mSCs and the same 13,886 TGs in nmSCs. Now, instead of 16,804 total TF-TG links, we retain only 14,557 total TF-TG links in the ground truth GRN for both SCs, of which 5,740 links are TF-TG for validated direct TGs. This corresponds to 9,253 unique TGs (including 4,185 unique TGs that are considered direct for at least 1 TF; nonetheless, they may be LOF TGs and/or direct TGs for other TFs). Now, 64,765 links are possible among the 7 TFs and all LOF TGs (pooled LOF only TGs and validated direct TGs) and 29,290 links are possible among the 7 TFs and all validated direct TGs.

Results of TF-TG regulatory networks: **mSCs:** 236149 rows originally in TF-TG regulatory network. Corresponding to 12,396 TGs and 221 TFs. After filtering: 183,242 rows now corresponding to 8,950 TGs and 221 TFs **nmSCs:** 657,013 rows originally in TF-TG regulatory network. Corresponding to 13,454 TGs and 233 TFs. After filtering (rounding test mse to 4 decimal places): 277,541 rows now corresponding to 5,207 TGs and 228 TFs.

Final Ground Truth Gene Regulatory Network (GRN) in Schwann Cells (Based on 13,886 Target Genes (TGs) found in Dorsal Root Ganglion (DRG) Schwann Cell Gene Expression Data and Multi-omics Data-Derived Reference GRNs for SCs)			
Transcription Factor (TF)	# LOF Only TGs	# Direct TGs	All LOF TGS
			Total
SOX10	4,916	2,102	7,018
YY1	2,122	1,079	3,201
STAT1	198	713	911
TEAD1	526	652	1,178
NR2F2	228	660	888
SREBF1	102	327	429
EGR2	725	207	932

§22.2 Relative gene expression percentile analysis

Schwann cells (SCs)

We assess the relative gene expression levels of all predicted TGs for all of the TFs in mSCs and/or nmSCs in SCs using GTEx gene expression data across various tissues (Eraslan et. al 2022) We compare these results with the corresponding respective levels using expression data for 4 CNS cell types sourced from Lake et al. (Lake et al. 2018). While we perform this analysis for all TFs in mSCs and nmSCs, we are primarily interested in ascertaining whether the TGs for core SC TFs (e.g. EGR2, NR2F2, SOX10, SREBF1, TEAD1, YY1) exhibit cell-type specificity.

To refine our exploration, we delve into comparative analyses of relative gene expression percentiles across 5 unique cell types in the nervous system, leveraging gene expression data from the aforementioned validation datasets. Recognizing variations in expression values and the spectrum of genes between datasets from (Lake et al. 2018) and (Eraslan et. al 2022), relative gene expression percentiles are employed as a common ground for comparison. Every gene within a given cell type and dataset is ranked by its expression level relative to its peers in that cell type, compiling these ranks for all TGs per TF in SCs, mindful of potential absences of some TGs from the datasets. This procedure is mirrored for 4 other CNS cell types, with an acknowledgment of possible missing TGs. To ensure the robustness of our findings, we utilize 1-sided paired t-tests to compare the relative gene expression percentiles of TGs in SCs against the 4 CNS cell types, with non-TGs for the given TF as a baseline control. We retrieve p-values for all TFs in nmSCs (for their respective nmSC TGs) and for all TFs in mSCs (for their respective mSC TGs) and perform multiple-testing correction using the Benjamin-Hochberg (BH) procedure. We retain significant comparisons as those where the adjusted p-value is less than 5%. Results are deemed meaningful if relative gene expression percentiles for our TGs are not only statistically higher in SCs compared to other CNS cell types but also exhibit specificity to this group of TGs (i.e., significance is not observed in control genes).

§22.3 Hypergeometric test of TF-TG regulatory network overlaps with known TGs for TFs in Schwann Cells (SCs)

In myelinating (mSCs) and non-myelinating (nmSCs), we perform hypergeometric testing of overlaps of predicted TGs with ground truth TGs to see if the predicted results are statistically significant. We do this for all Loss-of-Function (LOF) TGs and for the verified Direct TGs for the 7 SC TFs that we have experimental validation data for in rodents (e.g. rats). Please see section "Experimental data for Schwann cells" for more details. As a reminder, all LOF TGs comprise Direct TGs as well as LOF only TGs. Since we are missing corresponding LOF knockout data for RXRG, we cannot do this analysis for RXRG. We have 9,253 LOF TGs across 7 core SC TFs: EGR2, SOX10, NR2F2, STAT1, TEAD1, YY1, SREBF1. And, we have 7,018 LOF TGs for SOX10, 3,201 LOF TGs for YY1, 911 LOF TGs for STAT1, 1,178 LOF TFs for TEAD1, 888 LOF TGs for NR2F2, 429 LOF TGs for SREBF1, and 932 LOF TGs for EGR2. We also find 4,185 validated Direct TGs across these 7 core SC TFs, where we have: 2,102 for SOX10, 1,079 for YY1, 713 for STAT1, 652 for TEAD1, 660 for NR2F2, 327 for SREBF1, 207 for EGR2.

We run NetREm for myelinating (mSCs) and non-myelinating (nmSCs) SCs and find how many total TGs we have for each of these 7 core SC TFs. Then, we overlap these total TGs with LOF TGs for the TF for the LOF analysis (and overlap total TGs with validated Direct TGs for the TF for validated direct TG analysis).

For instance, for SOX10 LOF TGs in mSCs, we use the hypergeometric test to determine whether the overlap between the SOX10 predicted mSC TGs and the SOX10 LOF TGs is greater than what would be expected by random chance.
The Null Hypothesis of the Hypergeometric Test is: H_O: there is NO statistically significant association between the predicted SOX10 TGs and the LOF TGs for SOX10. Any overlap observed is due to random chance.
The Alternative Hypothesis of the Hypergeometric Test is: H_A: there is A statistically significant association between the predicted SOX10 TGs and the LOF TGs for SOX10. Any overlap observed is not due to random chance.

Suppose there are k common TGs between SOX10 mSC TGs and all LOF TGs for SOX10. This is the overlap. The p-value from the hypergeometric test represents the probability of observing at least k common TGs (or a more extreme overlap) between the predicted SOX10 TGs in mSCs and the SOX10 LOF TGs, assuming that the Null Hypothesis is true. We use the p-value to determine if the overlaps are statistically significant. This will help us determine if the predicted TGs in SOX10 in mSCs are over-enriched (for NetREm's TF-TG regulatory networks in mSCs).

For the hypergeometric test, there is P, the total population (total # of items in the population, which here is the total # of unique LOF TGs among the 7 SC TFs: 9,253 TGs). So $P = 9{,}253$. We also see the # of SOX10 LOF TGs in the ground truth network, which is $m = 7{,}018$. We look at the # of SOX10 mSC TGs that are also SOX10 LOF TGs (i.e. the overlap k), which in our case is $k = 564$ TGs. We also look at the LOF TGs that are found as core for any SC TF, which is the sample size n: 709 TGs. We then run the hypergeometric test with # of successes $k = 564$ TGs, sample size $n = 709$ TGs, # of successes in the population $m = 7{,}018$ TGs, population size $P = 9{,}253$ TGs. Statistically significant results will show that our set of SOX10 TGs in mSCs is overenriched SOX10 LOF TGs, which will be a good sign. In fact, we get "over enriched 1.05 fold compared to expectations", with a p-value of 0.00855 that is statistically significant over-enrichment. We do this for all 7 core SC TFs in mSCs and in nmSCs, for LOF TGs and for validated Direct TGs.

§22.4: Validation data: Sciatic Nerve Atlas Data for Schwann Cells in Mice

We utilize raw RNA-seq data for mSCs and nmSCs in the Sciatic Nerves (SN) of 4 mice from GSE137947 (Gerber et. al 2021), which comprises the SN Atlas (SNAT). We use g:Profiler(Reimand et al. 2007) orthologous functionality to help map the mouse (mus musculus) gene IDs to 16,954 human (homo sapiens) gene names. The gene expression data has data for 17,048 genes in myelinating (mSCs) Schwann cells (SCs) and in non-myelinating (nmSCs) SCs. We use this dataset to help prune down our list of TFs in mSCs and nmSCS, when we define our prior gene regulatory knowledge.

Section §23: Software Implementation of NetREm

Please note implement NetREm on GitHub as an open-source software package in Python: https://github.com/SaniyaKhullar/NetREm/tree/main.

§23.1: Handling input gene expression data

By default, NetREm uses 70% of the gene expression data for training the model and the remaining 30% for testing. Other default settings for NetREm include standardizing the X variable and standardizing the y variable. NetREm employs StandardScaler from the sklearn.preprocessing package(Pedregosa et al. 2011) to standardize X by fitting the StandardScaler (i.e. computing the mean and standard deviation of each feature) on X_{train}. Then, these calculated parameters are used to perform the transformation, i.e., subtracting the mean and dividing by the standard deviation, on both X_{train} and X_{test} sets. Similarly, to standardize y, NetRem fits the StandardScaler (i.e. computing the mean and standard deviation of each feature) on y_{train}. Then, these calculated parameters are used to perform the transformation, i.e., subtracting the mean and dividing by the standard deviation, on both y_{train} and y_{test} sets. These operations help convert $X^{(0)}$ and $y^{(0)}$, which are typically gene expression data and in measurements like FPKM, to unitless quantities X and y, respectively.

Standardizing a variable involves subtracting the mean and dividing by the standard deviation (which is positive number). For these M cell samples (rows), our $X^{(0)} \in \mathbb{R}^{M \times N}$ matrix contains single-cell gene expression data for these N TFs

(columns), while $y^{(0)} \in \mathbb{R}^M$ is the expression vector for our given TG. Again, the dimensions of $X^{(0)}$ may vary across TGs as the number of candidate TFs, N, may differ for TGs if we incorporate prior knowledge on cell-type gene regulation for initial feature selection. We note that this single-cell gene expression data may have been subject to batch correction and other pre-processing using toolkits like Seurat (e.g. normalizing molecular counts to consider impact of variance in sequencing depth across cells that may be of different cell-types. For instance, Seurat's LogNormalize method divides the counts for each gene in a cell by the total counts for that cell (that are multiplied by a scaling factor, e.g. 10,000) to yield a value; then $\log_e(\text{value} + 1)$ is calculated for each gene to yield the normalized counts (e.g. log-transformed counts per 10,000 total counts). In addition, Seurat's scaleData method scales each gene to have a mean of 0 and variance of 1 across cells; nonetheless, we do not make assumptions regarding the prior pre-processing or units of our original gene expression data $X^{(0)}$ and $y^{(0)}$. When we run NetREm, we randomly partition our M samples into mutually exclusive training and testing samples and select the corresponding $(X^{(0)}, y^{(0)})$ gene expression data for our respective training $(X_{train}^{(0)}, y_{train}^{(0)})$ and testing data $(X_{test}^{(0)}, y_{test}^{(0)})$. We will standardize our datasets utilizing the training data as a guide to ensure robust model generalization to unseen data by preventing data leakage from the test set during the preprocessing stage.

The user can opt to center the response y variable ($y - \bar{y}$, where $\bar{y}$ is the mean of y), which is False by default. If the user opts to center the y variable, then NetREm will compute $\overline{y_{train}}$ and subtract that from the response variables in both y_{train} and y_{test} sets to center y. This implementation thus utilizes the training data as a guide to ensure robust model generalization to unseen data by preventing data leakage from the test set during the preprocessing stage.

§23.2: Running netrem and netremCV functions

NetREm utilizes 2 hyperparameters: β for network constraint and α for sparsity. These can either be optimized dataset-specifically through cross-validation (CV) or manually set by the user. The chosen values strike a balance between the constraints imposed by the hyperparameters and the fit to the data. Lasso regression is performed on $\tilde{X}$ and $\tilde{y}$ using Python's scikit-learn package (Pedregosa et al. 2011). We provide 2 distinct functions: *netrem* and *netremCV*, each with its own strategy for hyperparameter selection. Generally, the choice of β influences the optimal value for α, given that α depends on the embeddings $\tilde{X}$ and $\tilde{y}$, which are scaled by β.

That is β is the network-constrained hyperparameter and it defines network regression gene embeddings $\tilde{X}$ and $\tilde{y}$. It does this by impacting the weight of the prior network knowledge (i.e. A that is based on input PPI network (PPIN)) in determining $E = \frac{X^T X}{M} + \beta A \in \mathbb{R}^{N \times N}$, upon which Singular Value Decomposition (SVD) is performed to then help us eventually obtain our network regression gene embeddings: $\tilde{X}$ and $\tilde{y}$. Thus, we often select β first and then may use LassoCV solvers to optimize α based on $\tilde{X}$ and $\tilde{y}$.

Traditional Lasso solvers in Python's sci-kit learn (Pedregosa et al. 2011) use α to denote the sparsity prior for Lasso $\mathcal{L}_1$ regularization. NetREm inherits from sklearn.base *RegressorMixin* and *BaseEstimator* classes and is a Sci-kit Learn estimator itself. Hence, for consistency and to avoid confusion, we retain α for our sparsity prior. Nonetheless, β is the 1st hyperparameter selected and α is conditioned to operate based on embeddings generated for the given β. Hence, contrary to the Greek alphabet, our order is sequential for our hyperparameters: $\beta \rightarrow \alpha$.

We ensure that the list of N candidate TFs for the TG does not contain the TG as a TF (that is, we ensure there are no overlaps in variables in X and y).

<u>Function: *netrem*</u>

The *netrem* function offers multiple approaches for determining β and α and is more flexible. Users have the option of employing GridSearchCV (Pedregosa et al. 2011) or Bayesian optimization via the Scikit-Optimize (skopt) library with Gaussian process minimization Alternatively, users can manually input β (or use the default) and decide on α. If α is either provided or set to default, NetREm employs Lasso for model fitting; else, NetREm uses LassoCV to automatically optimize α via CV.

<u>Function: *netremCV*</u>

Contrastingly, *netremCV* employs CV to automatically optimize both β and α, although at a higher computational cost. Nonetheless, there are cases in which *netrem* may outperform *netremCV* or vice-versa; please assume that unless otherwise mentioned, *netrem* is used for the applications in this paper.

§23.3: ElasticNetREm implementation: elasticnetrem function

We also have an ElasticNetREm implementation of NetREm, which changes the objective function based on $\tilde{X}$ and $\tilde{y}$ to be solved via ElasticNet or ElasticNetCV instead of Lasso or LassoCV. In addition, there are other transformations that may utilize the updated $\tilde{X}$ and $\tilde{y}$ latent gene embedding representations, in a manner that combines the penalties of Lasso ($\mathcal{L}_1$norm) and Ridge ($\mathcal{L}_2$ norm) regularizations. For example, NetREm may be also solved via ElasticNet regression, with the following equation that is adapted from $Eq(2)$.

$$c^* = \underset{c}{\operatorname{argmin}}\, \tilde{f}(c) = \frac{1}{2N}\left|\left|\tilde{y} - \tilde{X}c\right|\right|^2 + \alpha\rho\left|\left|c\right|\right|_1 + \frac{\alpha(1-\rho)}{2}\left|\left|c\right|\right|_2^2$$

This originates from the corresponding network regularization optimization formula, which we denote as ElasticNetREm:

$$c^* = \underset{c}{\operatorname{argmin}}\, f(c) = \frac{1}{2M}\left|\left|y - Xc\right|\right|^2 + \alpha\rho\left|\left|c\right|\right|_1 + \frac{\alpha(1-\rho)}{2}\left|\left|c\right|\right|_2^2 + \frac{\beta}{2}\sum_{i=1}^{N}\sum_{j=1}^{N} w_{ij}\left(\frac{c_i}{\sqrt{d_i}} - \frac{c_j}{\sqrt{d_j}}\right)^2$$

Here ρ is the parameter that controls the balance between $\mathcal{L}_1$ and $\mathcal{L}_2$ penalties (i.e. $\mathcal{L}_1$ ratio), α is the overall strength of the regularization, $\left|\left|c\right|\right|_1$ is the $\mathcal{L}_1$ norm (i.e. $\sum_{i=1}^{N}|c_i|$), and $\left|\left|c\right|\right|_2^2$ is the $\mathcal{L}_2$ norm squared of the coefficients vector c (i.e. $\sum_{i=1}^{N} c_i^2$). We note that $0 \le \rho \le 1$, where $\rho = 1$ for Lasso ($\mathcal{L}_1$ penalty only) and $\rho = 0$ for Ridge ($\mathcal{L}_2$ penalty only).

§23.4: Handling directed input prior networks

Further, NetREm is most effective when applied using positive-weighted, undirected prior networks as constraints. To assist users in converting directed networks into undirected similarity networks among nodes, we implement a weighted Node2Vec network embedding algorithm (Cohen 2023; Grover and Leskovec 2016). This algorithm conducts random walks in the directed network, inputs these walks into a skipgram model (neural-network that predicts the likelihood of the content nodes given the current nodes) to learn node embeddings, and then calculates the cosine similarity among the nodes based on these embeddings.

§23.5: Running NetREm for a cell-type (many target genes)

Simprem function (no prior GRN knowledge)

We recognize that in the absence of prior gene regulatory network (GRN) knowledge, we will have a fixed set and # of candidate TFs for each target gene (TG): $N = \mathcal{N}$, where $\mathcal{N}$ is the set of potential cell-type TFs. In the cases where the TG is also one of the TFs in $\mathcal{N}$, we will just remove that TF from the list of TFs for that TG, so that TG will have $N = \mathcal{N} - 1$ candidate TFs. When we run NetREm for each TG (step-by-step in an iterative loop), we can optimize its performance by realizing that the underlying input TF-TF PPIN network would be the same for a majority of cases (where TGs are not TFs), so we can reuse that network each time (instead of spending time preprocessing and recreating the same TF-TF PPIN network (W) and deriving V, D, A) and only make changes to the TF-TF PPIN network for those cases where the TG is also a TF. Thus, we build out the simprem function to keep things simpler. The *simprem* function helps us run NetREm faster in the absence of prior knowledge.

netremany function (with prior GRN knowledge)

This is a portmanteau of “NetREm” and “many” and is a capsule function that allows us to run NetREm when there may be prior GRN knowledge for some TGs, where we have some tailored candidate TFs for them. It enables the user to run NetREm for dozens or thousands of TGs in the cell-type in 1 function. Naturally, it will be slower than simprem because it is recreating the TF-TF PPIN for each TG in the cell-type, which takes time.

Section §24: References

Afgan E, Baker D, Batut B, van den Beek M, Bouvier D, Čech M, Chilton J, Clements D, Coraor N, Grüning BA, et al. 2018. The Galaxy platform for accessible, reproducible and collaborative biomedical analyses: 2018 update. *Nucleic Acids Res* **46**: W537–W544.

Ahsendorf T, Müller F-J, Topkar V, Gunawardena J, Eils R. 2017. Transcription factors, coregulators, and epigenetic marks are linearly correlated and highly redundant. *PLOS ONE* **12**: e0186324.

Aibar et. al 2017. SCENIC: single-cell regulatory network inference and clustering | Nature Methods. https://www.nature.com/articles/nmeth.4463 (Accessed March 27, 2023).

Aibar S, González-Blas CB, Moerman T, Huynh-Thu VA, Imrichova H, Hulselmans G, Rambow F, Marine J-C, Geurts P, Aerts J, et al. 2017. SCENIC: Single-cell regulatory network inference and clustering. *Nat Methods* **14**: 1083–1086.

Alanis-Lobato et. al 2017. HIPPIE v2.0: enhancing meaningfulness and reliability of protein–protein interaction networks | Nucleic Acids Research | Oxford Academic. https://academic.oup.com/nar/article/45/D1/D408/2290937 (Accessed October 5, 2024).

Alawad DM, Katebi A, Kabir MWU, Hoque MT. 2023. AGRN: accurate gene regulatory network inference using ensemble machine learning methods. *Bioinforma Adv* **3**: vbad032.

Ali O, Farooq A, Yang M, Jin VX, Bjørås M, Wang J. 2022. abc4pwm: affinity based clustering for position weight matrices in applications of DNA sequence analysis. *BMC Bioinformatics* **23**: 83.

Avraham O, Chamessian A, Feng R, Yang L, Halevi AE, Moore AM, Gereau RWI, Cavalli V. 2022. Profiling the molecular signature of satellite glial cells at the single cell level reveals high similarities between rodents and humans. *PAIN* **163**: 2348.

Badia-i-Mompel P, Wessels L, Müller-Dott S, Trimbour R, Ramirez Flores RO, Argelaguet R, Saez-Rodriguez J. 2023. Gene regulatory network inference in the era of single-cell multi-omics. *Nat Rev Genet* **24**: 739–754.

Balakrishnan A, Belfiore L, Chu T-H, Fleming T, Midha R, Biernaskie J, Schuurmans C. 2021. Insights Into the Role and Potential of Schwann Cells for Peripheral Nerve Repair From Studies of Development and Injury. *Front Mol Neurosci* **13**: 608442.

Barabási and Oltvai et. al 2004. Network biology: understanding the cell's functional organization | Nature Reviews Genetics. https://www.nature.com/articles/nrg1272 (Accessed October 1, 2024).

Berenson A, Lane R, Soto-Ugaldi LF, Patel M, Ciausu C, Li Z, Chen Y, Shah S, Santoso C, Liu X, et al. 2023. Paired yeast one-hybrid assays to detect DNA-binding cooperativity and antagonism across transcription factors. *Nat Commun* **14**: 6570.

brainSCOPE Resource 2024. brainSCOPE Resource. *Brain Single-Cell Omics PsychEncode Resour*. https://brainscope.gersteinlab.org/ (Accessed September 16, 2024).

Broido AD, Clauset A. 2019. Scale-free networks are rare. *Nat Commun* **10**: 1017.

Bryois J, Calini D, Macnair W, Foo L, Urich E, Ortmann W, Iglesias VA, Selvaraj S, Nutma E, Marzin M, et al. 2022. Cell-type-specific cis-eQTLs in eight human brain cell types identify novel risk genes for psychiatric and neurological disorders. *Nat Neurosci* **25**: 1104–1112.

Buenrostro JD, Corces MR, Lareau CA, Wu B, Schep AN, Aryee MJ, Majeti R, Chang HY, Greenleaf WJ. 2018. Integrated Single-Cell Analysis Maps the Continuous Regulatory Landscape of Human Hematopoietic Differentiation. *Cell* **173**: 1535-1548.e16.

Campos LM de, Cano A, Castellano JG, Moral S. 2019. Combining gene expression data and prior knowledge for inferring gene regulatory networks via Bayesian networks using structural restrictions. *Stat Appl Genet Mol Biol* **18**. https://www.degruyter.com/document/doi/10.1515/sagmb-2018-0042/html (Accessed November 17, 2023).

Carlson et. al 2019. org.Hs.eg.db. *Bioconductor*. http://bioconductor.org/packages/org.Hs.eg.db/ (Accessed September 26, 2023).

Castro-Mondragon JA, Riudavets-Puig R, Rauluseviciute I, Berhanu Lemma R, Turchi L, Blanc-Mathieu R, Lucas J, Boddie P, Khan A, Manosalva Pérez N, et al. 2022. JASPAR 2022: the 9th release of the open-access database of transcription factor binding profiles. *Nucleic Acids Res* **50**: D165–D173.

Cermenati G, Audano M, Giatti S, Carozzi V, Porretta-Serapiglia C, Pettinato E, Ferri C, D'Antonio M, De Fabiani E, Crestani M, et al. 2015. Lack of Sterol Regulatory Element Binding Factor-1c Imposes Glial Fatty Acid Utilization Leading to Peripheral Neuropathy. *Cell Metab* **21**: 571–583.

Chagas VS, Groeneveld CS, Oliveira KG, Trefflich S, de Almeida RC, Ponder BAJ, Meyer KB, Jones SJM, Robertson AG, Castro MAA. 2019. RTNduals: an R/Bioconductor package for analysis of co-regulation and inference of dual regulons. *Bioinformatics* **35**: 5357–5358.

Chan TE, Stumpf MPH, Babtie AC. 2017. Gene Regulatory Network Inference from Single-Cell Data Using Multivariate Information Measures. *Cell Syst* **5**: 251-267.e3.

Chen et. al 2023. SWEET: a single-sample network inference method for deciphering individual features in disease | Briefings in Bioinformatics | Oxford Academic. https://academic.oup.com/bib/article/24/2/bbad032/7017366 (Accessed September 20, 2024).

Chen J, Xiao L, Rao JN, Zou T, Liu L, Bellavance E, Gorospe M, Wang J-Y. 2008. JunD Represses Transcription and Translation of the Tight Junction Protein Zona Occludens-1 Modulating Intestinal Epithelial Barrier Function. *Mol Biol Cell* **19**: 3701–3712.

Coetzee SG, Coetzee GA, Hazelett DJ. 2015. motifbreakR: an R/Bioconductor package for predicting variant effects at transcription factor binding sites. *Bioinformatics* **31**: 3847–3849.

Cohen E. 2023. Node2Vec. https://github.com/eliorc/node2vec (Accessed September 3, 2023).

Datta RR, Rister J. 2022. The power of the (imperfect) palindrome: Sequence-specific roles of palindromic motifs in gene regulation. *BioEssays* **44**: 2100191.

Dibaeinia P. 2024. PayamDiba/SERGIO. https://github.com/PayamDiba/SERGIO (Accessed February 17, 2024).

Dibaeinia P, Sinha S. 2020. SERGIO: A Single-Cell Expression Simulator Guided by Gene Regulatory Networks. *Cell Syst* **11**: 252-271.e11.

Drew K, Wallingford JB, Marcotte EM. 2021. hu.MAP 2.0: integration of over 15,000 proteomic experiments builds a global compendium of human multiprotein assemblies. *Mol Syst Biol* **17**: e10016.

Durinck S, Spellman PT, Birney E, Huber W. 2009. Mapping identifiers for the integration of genomic datasets with the R/Bioconductor package biomaRt. *Nat Protoc* **4**: 1184–1191.

Emani PS, Liu JJ, Clarke D, Jensen M, Warrell J, Gupta C, Meng R, Lee CY, Xu S, Dursun C, et al. 2024. Single-cell genomics and regulatory networks for 388 human brains. *Science* **384**: eadi5199.

Eraslan et. al 2022. Single-nucleus cross-tissue molecular reference maps toward understanding disease gene function | Science. https://www.science.org/doi/10.1126/science.abl4290 (Accessed April 5, 2024).

Escorcia-Rodríguez JM, Gaytan-Nuñez E, Hernandez-Benitez EM, Zorro-Aranda A, Tello-Palencia MA, Freyre-González JA. 2023. Improving gene regulatory network inference and assessment: The importance of using network structure. *Front Genet* **14**. https://www.frontiersin.org/articles/10.3389/fgene.2023.1143382 (Accessed November 24, 2023).

Fletcher MNC, Castro MAA, Wang X, de Santiago I, O'Reilly M, Chin S-F, Rueda OM, Caldas C, Ponder BAJ, Markowetz F, et al. 2013. Master regulators of FGFR2 signalling and breast cancer risk. *Nat Commun* **4**: 2464.

Fogarty et. al 2020. SOX10-regulated promoter use defines isoform-specific gene expression in Schwann cells | BMC Genomics | Full Text. https://bmcgenomics.biomedcentral.com/articles/10.1186/s12864-020-06963-7 (Accessed September 7, 2023).

Gentleman RC, Carey VJ, Bates DM, Bolstad B, Dettling M, Dudoit S, Ellis B, Gautier L, Ge Y, Gentry J, et al. 2004. Bioconductor: open software development for computational biology and bioinformatics. *Genome Biol* **5**: R80.

Gerber et. al 2021. GEO Accession viewer. https://www.ncbi.nlm.nih.gov/geo/query/acc.cgi?acc=GSE137870 (Accessed September 29, 2023a).

Gerber et. al 2021. Transcriptional profiling of mouse peripheral nerves to the single-cell level to build a sciatic nerve ATlas (SNAT) | eLife. https://elifesciences.org/articles/58591 (Accessed September 29, 2023b).

Giambartolomei et. al, 2014. Bayesian Test for Colocalisation between Pairs of Genetic Association Studies Using Summary Statistics | PLOS Genetics. https://journals.plos.org/plosgenetics/article?id=10.1371/journal.pgen.1004383 (Accessed November 16, 2023).

Giurgiu M, Reinhard J, Brauner B, Dunger-Kaltenbach I, Fobo G, Frishman G, Montrone C, Ruepp A. 2019. CORUM: the comprehensive resource of mammalian protein complexes—2019. *Nucleic Acids Res* **47**: D559–D563.

Göös H, Kinnunen M, Salokas K, Tan Z, Liu X, Yadav L, Zhang Q, Wei G-H, Varjosalo M. 2022. Human transcription factor protein interaction networks. *Nat Commun* **13**: 766.

Greenfield et. al 2013. Robust data-driven incorporation of prior knowledge into the inference of dynamic regulatory networks | Bioinformatics | Oxford Academic. https://academic.oup.com/bioinformatics/article/29/8/1060/232957 (Accessed August 23, 2024).

Groeneveld C, Robertson G, Wang X, Fletcher M, Markowetz F, Meyer K, Castro M. 2023. RTN: RTN: Reconstruction of Transcriptional regulatory Networks and analysis of regulons. https://bioconductor.org/packages/RTN/ (Accessed November 7, 2023).

Grover A, Leskovec J. 2016. node2vec: Scalable Feature Learning for Networks. http://arxiv.org/abs/1607.00653 (Accessed September 3, 2023).

Gupta C, Xu J, Jin T, Khullar S, Liu X, Alatkar S, Cheng F, Wang D. 2022. Single-cell network biology characterizes cell type gene regulation for drug repurposing and phenotype prediction in Alzheimer's disease. *PLOS Comput Biol* **18**: e1010287.

Han X, Chen H, Huang D, Chen H, Fei L, Cheng C, Huang H, Yuan G-C, Guo G. 2018. Mapping human pluripotent stem cell differentiation pathways using high throughput single-cell RNA-sequencing. *Genome Biol* **19**: 47.

Hastie T. 2020. Ridge Regularization: An Essential Concept in Data Science. *Technometrics J Stat Phys Chem Eng Sci* **62**: 426–433.

He et. al, 2010. Yy1 as a molecular link between neuregulin and transcriptional modulation of peripheral myelination | Nature Neuroscience. https://www.nature.com/articles/nn.2686 (Accessed September 7, 2023).

Hoefsloot HCJ, Smit S, Smilde AK. 2008. A Classification Model for the Leiden Proteomics Competition. *Stat Appl Genet Mol Biol* **7**. https://www.degruyter.com/document/doi/10.2202/1544-6115.1351/html (Accessed November 17, 2023).

Hu C, Li T, Xu Y, Zhang X, Li F, Bai J, Chen J, Jiang W, Yang K, Ou Q, et al. 2023. CellMarker 2.0: an updated database of manually curated cell markers in human/mouse and web tools based on scRNA-seq data. *Nucleic Acids Res* **51**: D870–D876.

Hung HA, Sun G, Keles S, Svaren J. 2015. Dynamic Regulation of Schwann Cell Enhancers after Peripheral Nerve Injury *. *J Biol Chem* **290**: 6937–6950.

Jackson CA, Castro DM, Saldi G-A, Bonneau R, Gresham D. 2020. Gene regulatory network reconstruction using single-cell RNA sequencing of barcoded genotypes in diverse environments eds. N. Barkai and D. Weigel. *eLife* **9**: e51254.

Jin T, Rehani P, Ying M, Huang J, Liu S, Roussos P, Wang D. 2021. scGRNom: a computational pipeline of integrative multi-omics analyses for predicting cell-type disease genes and regulatory networks. *Genome Med* **13**: 95.

Ju H, Yun H, Kim Y, Nam YJ, Lee S, Lee J, Jeong SM, Heo J, Kwon H, Cho YS, et al. 2023. Activating transcription factor-2 supports the antioxidant capacity and ability of human mesenchymal stem cells to prevent asthmatic airway inflammation. *Exp Mol Med* **55**: 413–425.

Kamimoto K, Stringa B, Hoffmann CM, Jindal K, Solnica-Krezel L, Morris SA. 2023. Dissecting cell identity via network inference and in silico gene perturbation. *Nature* **614**: 742–751.

Kanehisa M, Goto S. 2000. KEGG: Kyoto Encyclopedia of Genes and Genomes. *Nucleic Acids Res* **28**: 27–30.

Karolchik D, Hinrichs AS, Kent WJ. 2009. The UCSC Genome Browser. *Curr Protoc Bioinforma Ed Board Andreas Baxevanis Al* **CHAPTER**: Unit1.4.

Kelner J, Koehler F, Meka R, Rohatgi D. 2021. On the Power of Preconditioning in Sparse Linear Regression. http://arxiv.org/abs/2106.09207 (Accessed March 9, 2024).

Kent WJ, Sugnet CW, Furey TS, Roskin KM, Pringle TH, Zahler AM, Haussler and D. 2002. The Human Genome Browser at UCSC. *Genome Res* **12**: 996–1006.

Klee S, Stamps MT. 2022. Eigenvalues of Graph Laplacians Via Rank-One Perturbations. *Q J Math* **73**: 609–616.

Kosoglu et. al 2024. Structural coverage of the human interactome | Briefings in Bioinformatics | Oxford Academic. https://academic.oup.com/bib/article/25/1/bbad496/7510974 (Accessed September 29, 2024).

Kotlyar et. al 2022. IID 2021: towards context-specific protein interaction analyses by increased coverage, enhanced annotation and enrichment analysis | Nucleic Acids Research | Oxford Academic. https://academic.oup.com/nar/article/50/D1/D640/6424757 (Accessed July 14, 2023).

Lake BB, Chen S, Sos BC, Fan J, Kaeser GE, Yung YC, Duong TE, Gao D, Chun J, Kharchenko PV, et al. 2018. Integrative single-cell analysis of transcriptional and epigenetic states in the human adult brain. *Nat Biotechnol* **36**: 70–80.

Lambert SA, Jolma A, Campitelli LF, Das PK, Yin Y, Albu M, Chen X, Taipale J, Hughes TR, Weirauch MT. 2018. The Human Transcription Factors. *Cell* **172**: 650–665.

Langfelder P, Horvath S. 2008. WGCNA: an R package for weighted correlation network analysis. *BMC Bioinformatics* **9**: 559.

Langmead B, Trapnell C, Pop M, Salzberg SL. 2009. Ultrafast and memory-efficient alignment of short DNA sequences to the human genome. *Genome Biol* **10**: R25.

Langmead et. al 2012. Fast gapped-read alignment with Bowtie 2 | Nature Methods. https://www.nature.com/articles/nmeth.1923 (Accessed September 26, 2023).

Laval F, Coppin G, Twizere J-C, Vidal M. 2023. Homo cerevisiae—Leveraging Yeast for Investigating Protein–Protein Interactions and Their Role in Human Disease. *Int J Mol Sci* **24**: 9179.

Lawrence et. al 2013. Software for Computing and Annotating Genomic Ranges | PLOS Computational Biology. https://journals.plos.org/ploscompbiol/article?id=10.1371/journal.pcbi.1003118 (Accessed March 12, 2024).

Lawrence M, Gentleman R, Carey V. 2009. rtracklayer: an R package for interfacing with genome browsers. *Bioinformatics* **25**: 1841–1842.

Lê Cao K-A, Boitard S, Besse P. 2011. Sparse PLS discriminant analysis: biologically relevant feature selection and graphical displays for multiclass problems. *BMC Bioinformatics* **12**: 253.

Le et. al 2005. Nab proteins are essential for peripheral nervous system myelination | Nature Neuroscience. https://www.nature.com/articles/nn1490 (Accessed September 7, 2023).

Lemaître G, Nogueira F, Aridas CK. 2017. Imbalanced-learn: A Python Toolbox to Tackle the Curse of Imbalanced Datasets in Machine Learning. *J Mach Learn Res* **18**: 1–5.

Lerdrup et. al 2016. An interactive environment for agile analysis and visualization of ChIP-sequencing data | Nature Structural & Molecular Biology. https://www.nature.com/articles/nsmb.3180 (Accessed September 26, 2023).

Li and Li 2008. Network-constrained regularization and variable selection for analysis of genomic data | Bioinformatics | Oxford Academic. https://academic.oup.com/bioinformatics/article/24/9/1175/206444 (Accessed March 27, 2023).

Li G, Ruan X, Auerbach RK, Sandhu KS, Zheng M, Wang P, Poh HM, Goh Y, Lim J, Zhang J, et al. 2012. Extensive Promoter-centered Chromatin Interactions Provide a Topological Basis for Transcription Regulation. *Cell* **148**: 84–98.

Liu T. 2014. Use model-based Analysis of ChIP-Seq (MACS) to analyze short reads generated by sequencing protein-DNA interactions in embryonic stem cells. *Methods Mol Biol Clifton NJ* **1150**: 81–95.

Liu T, Xia L, Yao Y, Yan C, Fan Y, Gajendran B, Yang J, Li Y-J, Chen J, Filmus J, et al. 2019. Identification of diterpenoid compounds that interfere with Fli-1 DNA binding to suppress leukemogenesis. *Cell Death Dis* **10**: 1–11.

Lopez-Anido C, Sun G, Koenning M, Srinivasan R, Hung HA, Emery B, Keles S, Svaren J. 2015. Differential Sox10 genomic occupancy in myelinating glia. *Glia* **63**: 1897–1914.

Lopez-Anido et. al 2015. GEO Accession viewer. https://www.ncbi.nlm.nih.gov/geo/query/acc.cgi?acc=GSE64703 (Accessed July 14, 2023).

Luck K, Kim D-K, Lambourne L, Spirohn K, Begg BE, Bian W, Brignall R, Cafarelli T, Campos-Laborie FJ, Charloteaux B, et al. 2020. A reference map of the human binary protein interactome. *Nature* **580**: 402–408.

Machiela et. al. 2015. LDLink: Web-based applications to interrogate linkage disequilibrium in populations - NCI. https://dceg.cancer.gov/tools/analysis/ldlink (Accessed August 27, 2023).

Machlab D, Burger L, Soneson C, Rijli FM, Schübeler D, Stadler MB. 2022. monaLisa: an R/Bioconductor package for identifying regulatory motifs. *Bioinformatics* **38**: 2624–2625.

Mathys et. al 2019. Single-cell transcriptomic analysis of Alzheimer's disease | Nature. https://www.nature.com/articles/s41586-019-1195-2 (Accessed March 27, 2023).

Matsumoto H, Kiryu H, Furusawa C, Ko MSH, Ko SBH, Gouda N, Hayashi T, Nikaido I. 2017. SCODE: an efficient regulatory network inference algorithm from single-cell RNA-Seq during differentiation. *Bioinformatics* **33**: 2314–2321.

McCalla SG, Fotuhi Siahpirani A, Li J, Pyne S, Stone M, Periyasamy V, Shin J, Roy S. 2023. Identifying strengths and weaknesses of methods for computational network inference from single-cell RNA-seq data. *G3 GenesGenomesGenetics* **13**: jkad004.

Meyer et. al 2008. minet: A R/Bioconductor Package for Inferring Large Transcriptional Networks Using Mutual Information | BMC Bioinformatics | Full Text. https://bmcbioinformatics.biomedcentral.com/articles/10.1186/1471-2105-9-461 (Accessed January 28, 2024).

Michaelis AC, Brunner A-D, Zwiebel M, Meier F, Strauss MT, Bludau I, Mann M. 2023. The social and structural architecture of the yeast protein interactome. *Nature* **624**: 192–200.

Moerman T, Aibar Santos S, Bravo González-Blas C, Simm J, Moreau Y, Aerts J, Aerts S. 2019. GRNBoost2 and Arboreto: efficient and scalable inference of gene regulatory networks. *Bioinformatics* **35**: 2159–2161.

Morselli Gysi D, do Valle Í, Zitnik M, Ameli A, Gan X, Varol O, Ghiassian SD, Patten JJ, Davey RA, Loscalzo J, et al. 2021. Network medicine framework for identifying drug-repurposing opportunities for COVID-19. *Proc Natl Acad Sci* **118**: e2025581118.

Ndungu A, Payne A, Torres JM, Bunt M van de, McCarthy MI. 2020. A Multi-tissue Transcriptome Analysis of Human Metabolites Guides Interpretability of Associations Based on Multi-SNP Models for Gene Expression. *Am J Hum Genet* **106**: 188–201.

Nguyen H, Tran D, Tran B, Pehlivan B, Nguyen T. 2020. A comprehensive survey of regulatory network inference methods using single cell RNA sequencing data. *Brief Bioinform* **22**: bbaa190.

Nicodemus KK, Malley JD. 2009. Predictor correlation impacts machine learning algorithms: implications for genomic studies. *Bioinformatics* **25**: 1884–1890.

Nie J, Stewart R, Zhang H, Thomson JA, Ruan F, Cui X, Wei H. 2011. TF-Cluster: A pipeline for identifying functionally coordinated transcription factors via network decomposition of the shared coexpression connectivity matrix (SCCM). *BMC Syst Biol* **5**: 53.

Nott A, Holtman IR, Coufal NG, Schlachetzki JCM, Yu M, Hu R, Han CZ, Pena M, Xiao J, Wu Y, et al. 2019. Brain cell type–specific enhancer–promoter interactome maps and disease-risk association. *Science* **366**: 1134–1139.

Nutma E, van Gent D, Amor S, Peferoen LAN. 2020. Astrocyte and Oligodendrocyte Cross-Talk in the Central Nervous System. *Cells* **9**: 600.

Oh S, Abdelnabi J, Al-Dulaimi R, Aggarwal A, Ramos M, Davis S, Riester M, Waldron L. 2022. HGNChelper: identification and correction of invalid gene symbols for human and mouse. *F1000Research* **9**: 1493.

Oughtred R, Rust J, Chang C, Breitkreutz B, Stark C, Willems A, Boucher L, Leung G, Kolas N, Zhang F, et al. 2021. The BioGRID database: A comprehensive biomedical resource of curated protein, genetic, and chemical interactions. *Protein Sci Publ Protein Soc* **30**: 187–200.

Ouyang Z, Zhou Q, Wong WH. 2009. ChIP-Seq of transcription factors predicts absolute and differential gene expression in embryonic stem cells. *Proc Natl Acad Sci* **106**: 21521–21526.

Parab L, Pal S, Dhar R. 2022. Transcription factor binding process is the primary driver of noise in gene expression. *PLOS Genet* **18**: e1010535.

Pedregosa F, Varoquaux G, Gramfort A, Michel V, Thirion B, Grisel O, Blondel M, Prettenhofer P, Weiss R, Dubourg V, et al. 2011. Scikit-learn: Machine Learning in Python. *J Mach Learn Res* **12**: 2825–2830.

Peng X, Wang J, Peng W, Wu F-X, Pan Y. 2017. Protein–protein interactions: detection, reliability assessment and applications. *Brief Bioinform* **18**: 798–819.

Poitelon et. al 2016. YAP and TAZ control peripheral myelination and the expression of laminin receptors in Schwann cells | Nature Neuroscience. https://www.nature.com/articles/nn.4316 (Accessed September 7, 2023).

Pratapa et. al 2020. Benchmarking algorithms for gene regulatory network inference from single-cell transcriptomic data | Nature Methods. https://www.nature.com/articles/s41592-019-0690-6 (Accessed March 27, 2023).

Qiu X, Rahimzamani A, Wang L, Ren B, Mao Q, Durham T, McFaline-Figueroa JL, Saunders L, Trapnell C, Kannan S. 2020. Inferring Causal Gene Regulatory Networks from Coupled Single-Cell Expression Dynamics Using Scribe. *Cell Syst* **10**: 265-274.e11.

Quinlan AR, Hall IM. 2010. BEDTools: a flexible suite of utilities for comparing genomic features. *Bioinformatics* **26**: 841–842.

Reimand J, Kull M, Peterson H, Hansen J, Vilo J. 2007. g:Profiler—a web-based toolset for functional profiling of gene lists from large-scale experiments. *Nucleic Acids Res* **35**: W193–W200.

Richardson L. beautifulsoup4: Screen-scraping library. https://www.crummy.com/software/BeautifulSoup/bs4/ (Accessed August 25, 2023).

Rouillard AD, Gundersen GW, Fernandez NF, Wang Z, Monteiro CD, McDermott MG, Ma'ayan A. 2016. The harmonizome: a collection of processed datasets gathered to serve and mine knowledge about genes and proteins. *Database* **2016**: baw100.

Roy et. al 2020. PoLoBag: Polynomial Lasso Bagging for signed gene regulatory network inference from expression data | Bioinformatics | Oxford Academic. https://academic.oup.com/bioinformatics/article/36/21/5187/5875056 (Accessed November 17, 2023).

Roy S, Lagree S, Hou Z, Thomson JA, Stewart R, Gasch AP. 2013. Integrated Module and Gene-Specific Regulatory Inference Implicates Upstream Signaling Networks. *PLOS Comput Biol* **9**: e1003252.

Ruiz C, Zitnik M, Leskovec J. 2021. Identification of disease treatment mechanisms through the multiscale interactome. *Nat Commun* **12**: 1796.

Satija et. al 2015. Spatial reconstruction of single-cell gene expression data | Nature Biotechnology. https://www.nature.com/articles/nbt.3192 (Accessed April 10, 2024).

Satija et. al. 2024. Analysis, visualization, and integration of spatial datasets with Seurat. https://satijalab.org/seurat/articles/spatial_vignette (Accessed February 4, 2024).

Schep et. al 2023. motifmatchr.knit. https://bioconductor.org/packages/release/bioc/vignettes/motifmatchr/inst/doc/motifmatchr.html (Accessed August 25, 2023).

Seçilmiş D, Hillerton T, Tjärnberg A, Nelander S, Nordling TEM, Sonnhammer ELL. 2022. Knowledge of the perturbation design is essential for accurate gene regulatory network inference. *Sci Rep* **12**: 16531.

Sha Y, Qiu Y, Zhou P, Nie Q. 2024. Reconstructing growth and dynamic trajectories from single-cell transcriptomics data. *Nat Mach Intell* **6**: 25–39.

Shalek et. al 2014. Single-cell RNA-seq reveals dynamic paracrine control of cellular variation | Nature. https://www.nature.com/articles/nature13437 (Accessed April 4, 2024).

Shannon P, Richards M. 2023. MotifDb: An Annotated Collection of Protein-DNA Binding Sequence Motifs. https://bioconductor.org/packages/MotifDb/ (Accessed August 25, 2023).

Sharov AA, Nakatake Y, Wang W. 2022. Atlas of regulated target genes of transcription factors (ART-TF) in human ES cells. *BMC Bioinformatics* **23**: 377.

Shojaie A, Michailidis G. 2009. Analysis of Gene Sets Based on the Underlying Regulatory Network. *J Comput Biol* **16**: 407–426.

Siddharthan R. 2010. Dinucleotide Weight Matrices for Predicting Transcription Factor Binding Sites: Generalizing the Position Weight Matrix. *PLoS ONE* **5**: e9722.

Skok Gibbs C, Jackson CA, Saldi G-A, Tjärnberg A, Shah A, Watters A, De Veaux N, Tchourine K, Yi R, Hamamsy T, et al. 2022. High-performance single-cell gene regulatory network inference at scale: the Inferelator 3.0. *Bioinformatics* **38**: 2519–2528.

Skok Gibbs C, Mahmood O, Bonneau R, Cho K. 2024. PMF-GRN: a variational inference approach to single-cell gene regulatory network inference using probabilistic matrix factorization. *Genome Biol* **25**: 88.

Specht AT, Li J. 2017. LEAP: constructing gene co-expression networks for single-cell RNA-sequencing data using pseudotime ordering. *Bioinformatics* **33**: 764–766.

Srinivasan R, Sun G, Keles S, Jones EA, Jang S-W, Krueger C, Moran JJ, Svaren J. 2012. Genome-wide analysis of EGR2/SOX10 binding in myelinating peripheral nerve. *Nucleic Acids Res* **40**: 6449–6460.

Svaren J, Meijer D. 2008. The molecular machinery of myelin gene transcription in Schwann cells. *Glia* **56**: 1541–1551.

Szklarczyk D, Kirsch R, Koutrouli M, Nastou K, Mehryary F, Hachilif R, Gable AL, Fang T, Doncheva NT, Pyysalo S, et al. 2023. The STRING database in 2023: protein–protein association networks and functional enrichment analyses for any sequenced genome of interest. *Nucleic Acids Res* **51**: D638–D646.

Tan G, Lenhard B. 2016. TFBSTools: an R/bioconductor package for transcription factor binding site analysis. *Bioinformatics* **32**: 1555–1556.

Team BC, Maintainer BP, et. al 2019. TxDb.Hsapiens.UCSC.hg38.knownGene. *Bioconductor*. http://bioconductor.org/packages/TxDb.Hsapiens.UCSC.hg38.knownGene/ (Accessed August 25, 2023).

THE GTEX CONSORTIUM. 2020. The GTEx Consortium atlas of genetic regulatory effects across human tissues. *Science* **369**: 1318–1330.

The Tabula Sapiens Consortium. 2022. The Tabula Sapiens: A multiple-organ, single-cell transcriptomic atlas of humans. *Science* **376**: eabl4896.

Tran et. al 2019. Defining Reprogramming Checkpoints from Single-Cell Analyses of Induced Pluripotency - ScienceDirect. https://www.sciencedirect.com/science/article/pii/S2211124719305297 (Accessed March 9, 2024).

Tsukanov AV, Mironova VV, Levitsky VG. 2022. Motif models proposing independent and interdependent impacts of nucleotides are related to high and low affinity transcription factor binding sites in Arabidopsis. *Front Plant Sci* **13**: 938545.

van Dijk et. al 2018. Recovering Gene Interactions from Single-Cell Data Using Data Diffusion: Cell. https://www.cell.com/cell/fulltext/S0092-8674(18)30724-4?_returnURL=https%3A%2F%2Flinkinghub.elsevier.com%2Fretrieve%2Fpii%2FS0092867418307244%3Fshowall%3Dtrue (Accessed August 23, 2024).

Vecchiarelli-Federico LM, Liu T, Yao Y, Gao Y, Li Y, Li Y-J, Ben-David Y. 2017. Fli-1 overexpression in erythroleukemic cells promotes erythroid de-differentiation while Spi-1/PU.1 exerts the opposite effect. *Int J Oncol* **51**: 456–466.

Wallace C. 2020. Eliciting priors and relaxing the single causal variant assumption in colocalisation analyses. *PLOS Genet* **16**: e1008720.

Wang J, Liu Q, Sun J, Shyr Y. 2016. Disrupted cooperation between transcription factors across diverse cancer types. *BMC Genomics* **17**: 560.

Wang Y, Zhang X-S, Xia Y. 2009. Predicting eukaryotic transcriptional cooperativity by Bayesian network integration of genome-wide data. *Nucleic Acids Res* **37**: 5943–5958.

Wolf FA, Angerer P, Theis FJ. 2018. SCANPY: large-scale single-cell gene expression data analysis. *Genome Biol* **19**: 15.

Wu T, Hu E, Xu S, Chen M, Guo P, Dai Z, Feng T, Zhou L, Tang W, Zhan L, et al. 2021. clusterProfiler 4.0: A universal enrichment tool for interpreting omics data. *The Innovation* **2**. https://www.cell.com/the-innovation/abstract/S2666-6758(21)00066-7 (Accessed August 25, 2023).

Xu J, Zhang B, Cai J, Peng Q, Hu J, Askar P, Shangguan J, Su W, Zhu C, Sun H, et al. 2023. The transcription factor Stat-1 is essential for Schwann cell differentiation, myelination and myelin sheath regeneration. *Mol Med* **29**: 79.

Yu G. *Chapter 1 Overview of semantic similarity analysis | Biomedical Knowledge Mining using GOSemSim and clusterProfiler*. https://yulab-smu.top/biomedical-knowledge-mining-book/semantic-similarity-overview.html (Accessed August 25, 2023).

Yu G, Wang L-G, He Q-Y. 2015. ChIPseeker: an R/Bioconductor package for ChIP peak annotation, comparison and visualization. *Bioinformatics* **31**: 2382–2383.

Zaborowski AB, Walther D. 2020. Determinants of correlated expression of transcription factors and their target genes. *Nucleic Acids Res* **48**: 11347–11369.

Zeng B, Bendl J, Kosoy R, Fullard JF, Hoffman GE, Roussos P. 2022. Multi-ancestry eQTL meta-analysis of human brain identifies candidate causal variants for brain-related traits. *Nat Genet* **54**: 161–169.

Zhang K, Hocker JD, Miller M, Hou X, Chiou J, Poirion OB, Qiu Y, Li YE, Gaulton KJ, Wang A, et al. 2021. A single-cell atlas of chromatin accessibility in the human genome. *Cell* **184**: 5985-6001.e19.

Zhang R, Ren Z, Chen W. 2018. SILGGM: An extensive R package for efficient statistical inference in large-scale gene networks. *PLOS Comput Biol* **14**: e1006369.

Zhang S, Pyne S, Pietrzak S, Halberg S, McCalla SG, Siahpirani AF, Sridharan R, Roy S. 2023. Inference of cell type-specific gene regulatory networks on cell lineages from single cell omic datasets. *Nat Commun* **14**: 3064.

Zhang T, Zhang Z, Dong Q, Xiong J, Zhu B. 2020. Histone H3K27 acetylation is dispensable for enhancer activity in mouse embryonic stem cells. *Genome Biol* **21**: 45.

Zhang Y, Liu T, Meyer CA, Eeckhoute J, Johnson DS, Bernstein BE, Nusbaum C, Myers RM, Brown M, Li W, et al. 2008. Model-based analysis of ChIP-Seq (MACS). *Genome Biol* **9**: R137.

Zhao Y, Li M-C, Konaté MM, Chen L, Das B, Karlovich C, Williams PM, Evrard YA, Doroshow JH, McShane LM. 2021. TPM, FPKM, or Normalized Counts? A Comparative Study of Quantification Measures for the Analysis of RNA-seq Data from the NCI Patient-Derived Models Repository. *J Transl Med* **19**: 269.

Zhou et. al 2018. 2018. How to extract promoters positions. https://hzhou.scholar.harvard.edu/blog/how-extract-promoters-positions (Accessed August 25, 2023).

Zhou Y, Zhou B, Pache L, Chang M, Khodabakhshi AH, Tanaseichuk O, Benner C, Chanda SK. 2019. Metascape provides a biologist-oriented resource for the analysis of systems-level datasets. *Nat Commun* **10**: 1523.

Activity-by-contact model of enhancer–promoter regulation from thousands of CRISPR perturbations | Nature Genetics. https://www.nature.com/articles/s41588-019-0538-0 (Accessed September 29, 2024a).

CS109A - Advanced Section 2: Regularization. https://harvard-iacs.github.io/2019-CS109A/a-section/a-section2/ (Accessed March 9, 2024b).

Identifying ChIP-seq enrichment using MACS | Nature Protocols. https://www.nature.com/articles/nprot.2012.101 (Accessed September 24, 2023c).

Index of /gbdb/hg19/liftOver. https://hgdownload.soe.ucsc.edu/gbdb/hg19/liftOver/ (Accessed September 27, 2023d).

Index of /goldenPath/rn5/liftOver. https://hgdownload.soe.ucsc.edu/goldenPath/rn5/liftOver/ (Accessed August 25, 2023e).

2021. *Liftover to Map Genomic Coordinates Between Genome Builds with Rtracklayer Bioconductor Package*. https://www.youtube.com/watch?v=942WLiy3eb4 (Accessed August 25, 2023).

mygene: Python Client for MyGene.Info services. https://github.com/biothings/mygene.py (Accessed September 29, 2024f).

Structure-primed embedding on the transcription factor manifold enables transparent model architectures for gene regulatory network and latent activity inference | Genome Biology | Full Text. https://genomebiology.biomedcentral.com/articles/10.1186/s13059-023-03134-1 (Accessed October 2, 2024g).
